# Deprotective Alkylation: A Platform for Alcohol‐to‐Amine Interconversion Using Nonafluoromesitylenesulfonamides

**DOI:** 10.1002/anie.3520541

**Published:** 2026-09-08

**Authors:** Jakub Brześkiewicz, Kunj Kapur, Boris Maryasin, Nuno Maulide

**Affiliations:** ^1^ Institute of Organic Chemistry University of Vienna Vienna Austria

**Keywords:** alcohols, amines, deprotective functionalization, protecting group, stereochemistry

## Abstract

The direct conversion of alcohols to amines remains a long‐standing challenge in organic synthesis. In this study, we report a practical approach, based on the concept of deprotective functionalization, for the stereospecific synthesis of secondary and tertiary amines in high yields directly from alcohol precursors and Nms‐amides. Our approach exploits the distinct reactivity of Nms‐amides and has the potential to reduce step count in synthetic sequences. The versatility of the method is showcased through applications in late‐stage functionalization and the efficient preparation of isotopically labeled compounds.

## Graphical abstract

The straightforward conversion of alcohols to tertiary or secondary amines employing Nms‐amides in a single operation is described. The transformation features broad functional group tolerance and high stereospecificity, underscoring its utility in the synthesis of isotope‐labeled pharmaceuticals. In‐depth mechanistic studies reveal the distinct reactivity profile of Nms‐amides compared to other benchmark sulfonamide‐based protecting groups.

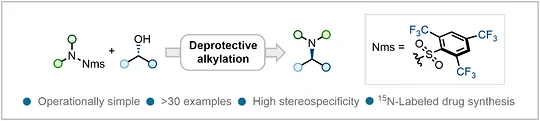

Amines are privileged structures in chemistry [1, 2, 3]. Access to alkylated amines is possible through a range of widely used transformations, such as alkylation with organohalides, reductive amination, amide reduction, hydroamination, or hydroaminoalkylation [4, 5, 6, 7]. Despite numerous advances in these strategies, they do not leverage the pronounced availability and cost efficiency of alcohols as starting materials [8, 9]. While direct conversion of an OH group into an amine moiety is restricted by the poor leaving group ability of hydroxide, one well‐established solution is hydrogen auto‐transfer (colloquially referred to as “borrowing hydrogen catalysis”) [10, 11, 12]. This strategy proceeds through a formally redox‐neutral process, in situ modulating oxidation states of the alcohol precursor and ultimately circumventing the need for preactivation of the hydroxy group. Although successfully developed over the last decade and providing efficient access to medicinally relevant building blocks (including optically pure products) [13], the reliance of “hydrogen borrowing” on transition metal catalysts and relatively harsh reaction conditions—often requiring high operating temperatures (80°C–200°C)—limits its applicability in late‐stage functionalization (Scheme 1A, top) [14].

**SCHEME 1:**
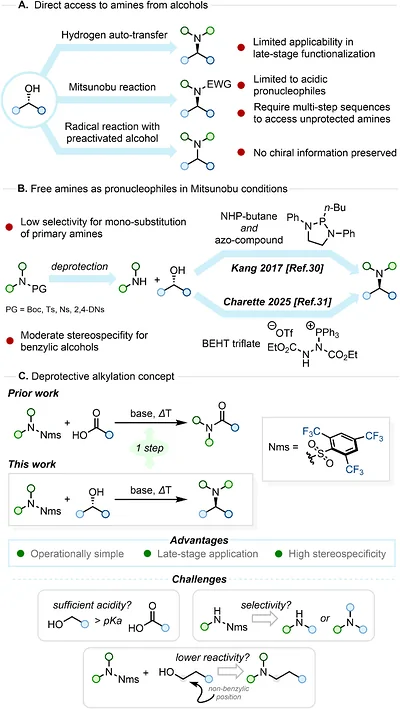
(A) Overview of direct amine synthesis from alcohols. (B) Current methods employing free amines in Mitsunobu reactions. (C) This work: deprotective alkylation approach enabling amine synthesis from Nms‐amides. EWG—electron‐withdrawing group; PG—protecting group; NHP—N‐heterocyclic phosphine, BEHT triflate—(1,2‐bis(ethoxycarbonyl)hydrazineyl)triphenylphosphonium trifluoromethanesulfonate; Nms—nonafluoromesitylenesulfonyl.

While the OH group readily lends itself to protonation into a good leaving group, the productive and broadly applicable substitution of alcohols with nitrogen‐based nucleophiles is commonly accepted to reside under the domain of Mitsunobu and related P(III)/P(V) reaction manifolds [15, 16, 17]. Albeit routinely employed as the method of choice for the alcohol‐to‐amine interconversion, the venerable Mitsunobu process carries its own intrinsic limitations, such as stoichiometric use of hazardous reagents and low atom economy. Despite considerable recent advancements, including the development of catalytic approaches that address safety concerns [18, 19, 20, 21, 22, 23, 24], the introduction of unprotected primary or secondary amines remains a persistent challenge. The basicity of the amine nitrogen renders unfunctionalized amines unsuitable as pronucleophiles, thereby restricting C–N bond formation in the context of the Mitsunobu reaction to acidic amine derivatives or precursors (p*K*a ≤ 11; sulfonamides [25], azides [26], and phthalimides [27]). This requirement mandates further multistep derivatization to access the target secondary or tertiary amines, devoid of the originally introduced p*K*a‐lowering functionality (Scheme 1A, middle). Recently, the direct transformation of alcohols to amines has also been achieved through radical processes [28, 29]. While valuable for access to oxidant‐sensitive tertiary amines, the inevitable loss of stereochemical information limits the practical utility of such approaches (Scheme 1A, bottom).

An elegant advancement in Mitsunobu chemistry, in which the use of an N‐heterocyclic phosphine (NHP‐butane) enables the direct deoxygenative coupling of unprotected amines with alcohols, was recently disclosed by Kang [30]. Despite avoiding the need for additional protection and deprotection sequences of the amine moiety, this method suffers from low selectivity for mono‐substitution when primary amines are employed. A similar, more broadly applicable method utilizing BEHT triflate was subsequently introduced by Charette (Scheme 1B) [31]. Although this approach exhibits milder reaction conditions and broader functional group tolerance, it generally struggles with lower stereoselectivity when applied to substrates prone to competing carbocationic pathways, which can override the classical S_N_2‐type displacement—with benzyl alcohols being the simplest examples [32].

Our group has recently introduced the Nms‐amide (Nms—nonafluoromesitylenesulfonyl) as a valuable protecting group, distinguished by its ease of handling, exceptional stability, and efficient deprotection profile [33]. Further investigation of the properties of Nms‐amides revealed their aptitude to undergo what we termed “deprotective functionalization” processes—transformations characterized by concurrent removal of the protecting group and functionalization of the liberated amine in a single operational step (as opposed to a one‐pot combination of deprotection and functionalization) [34, 35, 36]. Building on the efficient and direct synthesis of carboxamides from Nms‐amides and carboxylic acids [34], we speculated that the use of alkoxides could potentially afford secondary or tertiary amines through deoxygenative substitution (Scheme 1C). However, challenges related to the significantly lower acidity of alcohols compared to carboxylic acids, the considerably higher activation barrier of alkylations when compared to acylations, particularly for primary, non‐benzylic alcohols, and the risk of competitive overalkylation of amines make the implementation of such a concept particularly demanding.

In this study, we present a novel strategy, termed deprotective alkylation, to access secondary and tertiary amines directly from alcohols and Nms‐amides.

Drawing on prior work from our laboratory [34], we hypothesized that a secondary benzylic alcohol could be suitable for initial studies, due to both a slightly higher acidity of the OH group relative to aliphatic alcohols as well as its suitability for probing the stereospecific nature of the reaction. Preliminary investigations employing sodium hydride as the base, 1‐phenylethanol and tertiary Nms‐amide **1a** afforded the desired tertiary amine product **2s** in 29% yield (Scheme 2A, entry 1). Encouraged by these results, we evaluated a series of alternative bases (Cs_2_CO_3_, *t*‐BuOK, and *t*‐BuOLi; entries 3–5), among which lithium *tert*‐butoxide proved to be the most effective, enabling efficient alcohol deprotonation under comparatively mild conditions.

**SCHEME 2:**
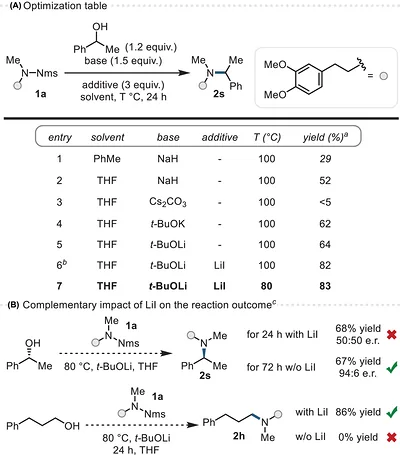
(A) Optimization table of deprotective alkylation. (B) The complementary impact of LiI on reaction efficiency and stereoselectivity. ^a^NMR yield determined by ^1^H NMR with CH_2_Br_2_ as an internal standard. ^b^1.5 equiv. of LiI. ^c^Yields correspond to isolated products. THF—tetrahydrofuran; w/o—without.

A significant improvement was achieved upon addition of lithium iodide to the system, presumably enabling an in situ Finkelstein‐type exchange (vide infra) [37]. Finally, after reducing the reaction temperature to 80°C, amine **2s** was obtained in 83% yield (Scheme 2A, entries 6 and 7). Importantly, high substrate conversion was achieved using only a small excess of the starting alcohol. This stands in stark contrast to hydrogen auto‐transfer reactions, which typically require a substantial excess of one of the reactants [38]. Whereas the addition of lithium iodide proved essential, racemization of the stereogenic center was observed when optically pure 1‐phenylethanol was employed (Scheme 2B, top). In contrast, in the absence of LiI, benzylic alcohols undergo the transformation with excellent levels of stereoinversion, albeit entailing longer reaction times. The complementary impact of LiI is particularly evident in the case of primary alcohols, which only afford the desired product in the presence of lithium iodide (Scheme 2B, bottom).

With suitable conditions established, we next explored the scope of this transformation with respect to the alcohol component (Scheme 3). Given the fact that N‐methylamines are a highly prevalent structural motif in medicinal chemistry [39, 40], it is noteworthy that only a stoichiometric amount of methanol is needed to effect deprotective methylation of Nms‐amide **1a**. In general, ethanol and benzyl alcohol yielded the corresponding amines (**2b** and **2c**) in high yields. Moreover, substituted benzyl (**2d**–**f**), allyl (**2g**), and alkyl (**2h**) alcohols furnished the desired amines in good to high yields. The protocol exhibited broad functional group tolerance, accommodating a free indole (**2i**), primary *tert*‐butyldimethylsilyl (TBS) ether (**2j**), aryl ether (**2k**) as well as an aldehyde (**2f**), manifesting orthogonality of our methodology to reductive amination for the generation of amines. In addition, an intramolecular variant of the reaction was achieved: conducting the transformation in the absence of lithium iodide (thereby presumably suppressing competing intermolecular pathways) enabled cyclization to the isoindoline (**2l**) in an excellent yield of 85%. To further demonstrate the utility of our protocol for the functionalization of structurally complex substrates, a series of pharmaceutically relevant alcohols (derived from Oxaprozin, Biotin, Sulindac, Febuxostat, and Ataluren) were subjected to the reaction conditions in the presence of Nms‐amide **1a**, affording the corresponding products (**2m**–**q**) in good to high yields. In the case of a Lithocholic acid‐derived substrate, preferential functionalization was observed at the primary alcohol over the secondary alcohol, providing **2r** selectively in 67% yield. Following this, a series of secondary alcohols was investigated. In general, activated secondary alcohols afforded moderate yields. However, in all cases where the enantiopure alcohol was employed as a starting material, a high enantiomeric ratio was observed (**2s**–**w**), indicating that the reaction proceeds with minimal erosion of stereochemical integrity. In addition, racemic mandelic acid *t*‐butyl ester and Lumefantrine (an antimalarial drug) afforded the amino ester **2x** and the diamine **2y** in 52% and 53% yield, respectively.

**SCHEME 3:**
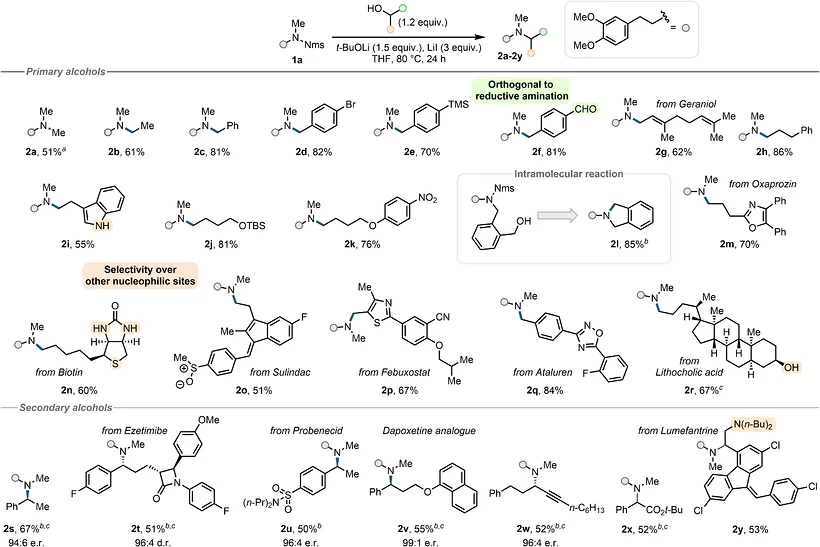
Scope of alcohols. Reaction conditions: sulfonamide (0.15 mmol), alcohol (0.18 mmol), *t*‐BuOLi (0.225 mmol), LiI (0.45 mmol), THF (1.5 mL), 80°C, 24 h, air atmosphere. ^a^Reaction performed with 20 mol% of LiI. ^b^Reaction performed without LiI. ^c^Reaction performed for 72 h. Yields correspond to isolated products. TMS—trimethylsilyl; TBS—*tert*‐butyldimethylsilyl.

We next turned our attention to the scope of sulfonamides, beginning with secondary sulfonamides (Scheme 4). Importantly, secondary Nms‐amides proved particularly challenging substrates due to competitive deprotonation of the sulfonamide (rather than the alcohol), resulting in reduced conversion of starting material. Following reoptimization of the reaction conditions (see the Supporting Information for details), we found that the use of a slightly weaker base, potassium trimethylsilanolate (TMSOK), improved the outcome of the reaction. The protocol displayed tolerance toward secondary Nms‐amides such as **3a** and **3b** bearing a secondary carbamate (NHBoc), and **3c**, possessing thiophene substituent. To illustrate the applicability of our method to late‐stage functionalization, a series of Nms‐amides derived from pharmaceutically relevant compounds was investigated. Notably, Menthylamine, Sitagliptin, Alogliptin and Primaquine analogues afforded the corresponding products (**3d**–**g**) in good to high yields. Furthermore, the synthesis of a Primaquine‐derived amine (**3g**) was successfully scaled up to deliver over 1 g of product in a single batch. Following the study of secondary Nms‐amides, we commenced the investigation of the tertiary sulfonamide scope by testing an Nms‐protected *N*‐Me‐*p*‐anisidine. Gratifyingly, even an aniline derivative afforded the product **3h** in an excellent yield of 84%. Furthermore, Boc‐protected piperazine delivered the amine **3i** in 76% yield. In the case of **3j**, which contains a free alcohol group, a slightly higher excess of *t‐*BuOLi was required to ensure efficient deprotonation and drive the reaction toward completion, affording the desired product in 84% yield. Finally, Nms‐amides derived from pharmaceuticals such as Paroxetine, Fasudil, Desloratadine, and Vonoprazan were readily transformed to the desired tertiary amines with high efficiency (**3k**–**n**).

**SCHEME 4:**
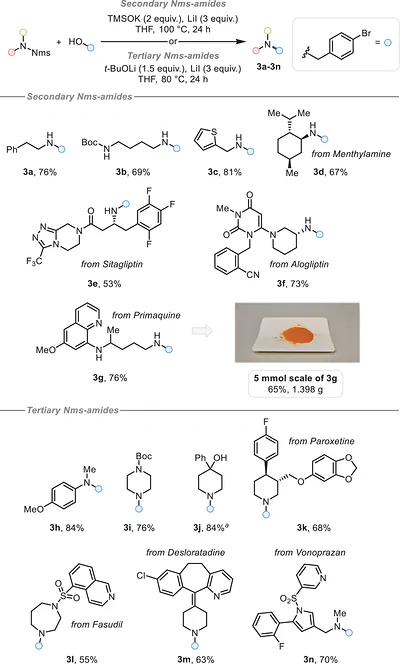
Scope of Nms‐amides. Yields correspond to isolated products. Reaction conditions: sulfonamide (0.15 mmol), alcohol (0.30 mmol), TMSOK (0.30 mmol), LiI (0.45 mmol), THF (1.5 mL), 100°C, 24 h, air atmosphere. Tertiary Nms‐amides reaction conditions: sulfonamide (0.15 mmol), alcohol (0.18 mmol), *t*‐BuOLi (0.225 mmol), LiI (0.45 mmol), THF (1.5 mL), 80°C, 24 h, air atmosphere. ^a^Reaction performed with 2.5 equiv. of *t*‐BuOLi. Boc—*tert*‐butyloxycarbonyl.

To further demonstrate the synthetic utility of the deprotective alkylation reaction, we first applied it to the synthesis of the antipsychotic agent Penfluridol (Scheme 5A). Our approach offers a remarkable reduction in step count compared to the method reported by Knochel and coworkers [41], leading to the target scaffold in only three synthetic steps (longest linear sequence). Notably, the implementation of the Nms protecting group streamlines the preparation of compound **5**, since the Grignard reagent addition step does not require an additional protectinggroup swap.

Importantly, the deprotective alkylation concept could be successfully extended to the incorporation of CD_3_ groups (Scheme 5B). Starting from inexpensive methanol‐*d*_4_, the described reactivity enables access to medicinally important deuterium‐labeled N‐methylamines [42], offering a practical and cost‐effective alternative to the use of expensive iodomethane‐*d*_3_. Keen to explore the stereochemical potential of our methodology, we compared the performance of deprotective alkylation with the recently developed protocol employing BEHT triflate [31]. Gratifyingly, the stereoinvertive synthesis of **2s** proceeded exceptionally better when deprotective functionalization of an Nms‐amide was employed, furnishing the product both in better yield and with a higher enantiomeric ratio (67%, e.r. 94:6, Scheme 5C). Finally, we sought to apply our approach to the synthesis of ^15^N‐labeled dimethylamine‐containing pharmaceuticals.

**SCHEME 5:**
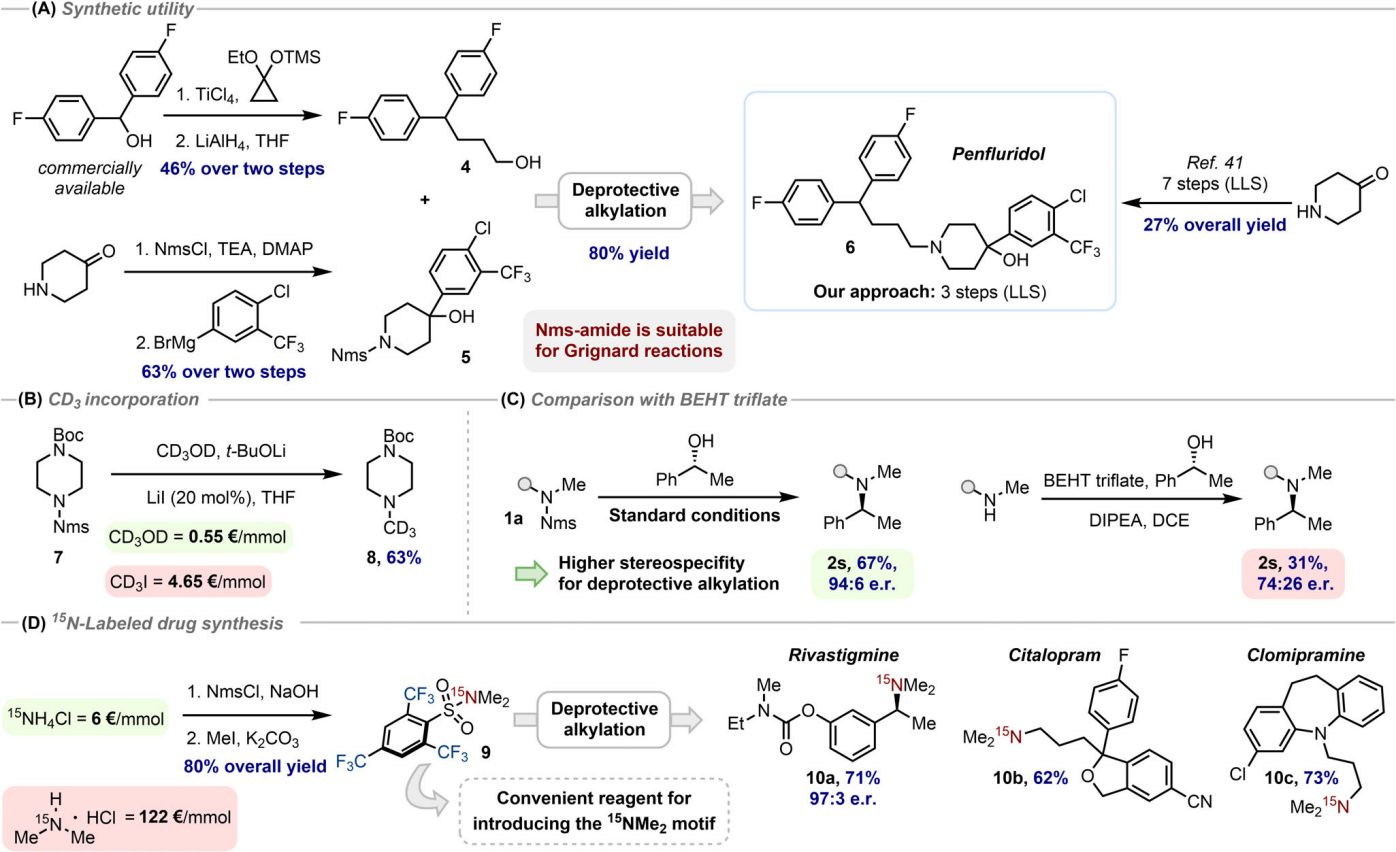
Application and performance comparison of the developed method. Price comparison based on data retrieved from www.sigmaaldrich.com on 26.04.2026. TEA—triethylamine; DMAP—4‐dimethylaminopyridine; LLS—longest linear sequence; DIPEA—diisopropylethylamine; DCE—1,2‐dichloroethane.

The dimethylamine pharmacophore is present in a broad range of FDA‐approved drugs, underscoring its importance in medicinal chemistry [42]. Moreover, the synthesis of ^15^N‐labeled medicinal compounds plays a crucial role in probing physicochemical activities of pharmaceuticals at a molecular level [43]. Using the cost‐effective ^15^N‐source ^15^NH_4_Cl, we synthesized dimethyl‐Nms‐amide **9** in excellent yield over two steps, circumventing the need for highly expensive ^15^ *N*‐dimethylamine hydrochloride. Furthermore, compound **9** was successfully implemented in the synthesis of three labeled drugs: Rivastigmine, Citalopram, and Clomipramine (**10a**–**c**), highlighting the potential of our strategy for future applications in medicinal chemistry (Scheme 5D). To give insight into the mechanism, several experiments were conducted. In the absence of LiI under standard conditions, treatment of Nms‐amide **1a** with 3‐phenylpropanol, afforded the aryl ether **11** in 40% yield (Scheme 6A). Subsequently, in an independent experiment, compound **11** was subjected to Finkelstein‐type conditions, ultimately providing (3‐iodopropyl)benzene in a yield of 85%. Based on these results, we wondered whether other commonly used sulfonamide protecting groups, such as Ns (4‐nitrobenzenesulfonyl) and 2,4‐DNs (2,4‐dinitrobenzenesulfonyl), exhibited identical reactivity. Strikingly, the use of **13** did not lead to the formation of the tertiary amine, even in trace amounts, despite the observation of aryl ether (**15**) formation in 24%. In contrast, for compound **14,** the desired amine **2h** was detected in minor quantities, while the corresponding aryl ether **16** constituted the predominant component of the reaction mixture (Scheme 6B). Collectively, these observations indicate that the conversion of the aryl ether intermediate to the corresponding alkyl iodide is a key step, proceeding with high efficiency only in the case of Nms‐amides.

**SCHEME 6:**
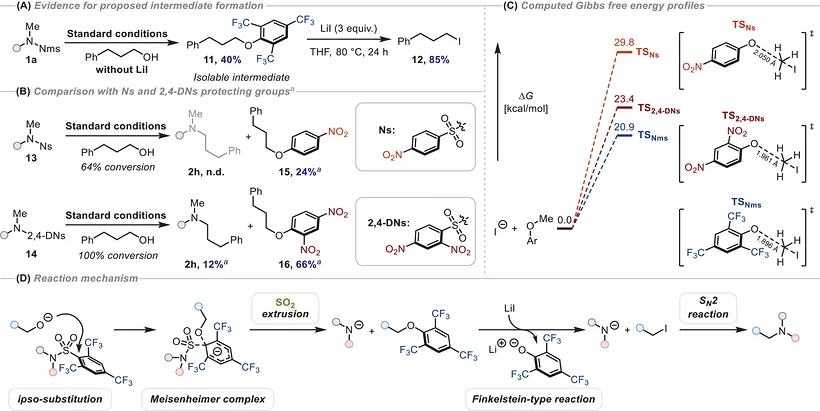
Mechanistic investigation and computed Gibbs free energy reaction barriers (Δ*G* ^‡^_353, THF_, kcal mol^−1^). Level of theory: B3LYP‐D3(BJ)/def2‐TZVP, SMD‐18(THF)//B3LYP‐D3(BJ)/def2‐SVP. The separated reactants (iodide anion and the ether) are taken as the reference (0.0 kcal mol^−1^). (a) NMR yield determined by ^1^H NMR with CH_2_Br_2_ as an internal standard. n.d.—not detected; TS—transition state.

To comprehensively elucidate the mechanism of this process and to clarify the differences in reactivity among sulfonamide‐based protecting groups, quantum chemical calculations were performed using density functional theory (DFT) at the B3LYP‐D3(BJ)/def2‐TZVP, SMD‐18(THF)//B3LYP‐D3(BJ)/def2‐SVP level of theory [44, 45, 46, 47, 48, 49] (Scheme 6C). Gibbs free energy barriers (Δ*G* ^‡^_353, THF_, kcal mol^−1^) were computed for the Finkelstein‐type step using separated reactants (iodide anion and methoxyarene) as the reference (0.0 kcal/mol); the methyl group was employed to simplify the model. The calculated barriers differ markedly among the protecting groups: 20.9 kcal mol^−1^ for Nms, consistent with the high experimental yield (86%) of product **2h**; 23.4 kcal mol^−1^ for 2,4‐DNs, corresponding to a lower yield (12%); and 29.8 kcal mol^−1^ for Ns, in agreement with the absence of the expected product **2h** and the predominance of aryl ether **15**.

Crucially, the formation of aryl ethers **11**, **15**, and **16** (for Nms, Ns, and 2,4‐DNs, respectively) provides strong evidence for the intermediacy of the Meisenheimer complex. Notably, the O–C bond lengths in the corresponding transition states follow a Hammond‐type trend, whereby higher activation barriers are associated with later, more product‐like transition states. Specifically, **TS_Nms** is the earliest (r(O─C) = 1.896 Å), **TS_2,4‐DNs** is intermediate (1.961 Å), and **TS_Ns** is the latest (2.050 Å). The observed effect can be rationalized by electronic factors. The strongly electron‐withdrawing CF_3_‐substituents in the Nms group stabilize the negatively charged transition state of the Finkelstein‐type step, in which charge buildup originates from the iodide nucleophile. In contrast, the less electron‐withdrawing 2,4‐DNs and Ns groups provide reduced stabilization, leading to higher barriers and diminished reactivity. Combined experimental and computational data support a mechanism initiated by nucleophilic attack of the alkoxide anion at the aryl‐sulfonamide resulting in formation of a Meisenheimer complex. Subsequent collapse of the complex, followed by the extrusion of SO_2_, affords a highly electron‐deficient aryl ether which undergoes Finkelstein‐type reactivity in which the phenolate is displaced by an iodide anion. Ultimately, the alkyl iodide and amide react through an S_N_2 pathway, furnishing the amine as the final product (Scheme 6D).

In summary, we have developed a novel method for the direct coupling of alcohols and Nms‐protected sulfonamides to the corresponding secondary or tertiary amines with high stereospecificity. The presented protocol enables the efficient synthesis of a broad range of amines, demonstrating its applicability in late‐stage functionalization of structurally complex drug derivatives. Importantly, the deprotective alkylation approach shortens the synthesis of biologically important pharmaceuticals such as Penfluridol and is a practical tool for introducing isotopically‐labeled motifs into medicinally relevant compounds. The unique reactivity of Nms‐amides is further substantiated by DFT calculations, highlighting the mechanistic intricacies of the developed strategy.

## Author Contributions

**Jakub Brześkiewicz**: investigation, methodology, and writing – original draft. **Kunj Kapur**: investigation, methodology, and writing – review and editing. **Boris Maryasin**: investigation, methodology, and writing – original draft. **Nuno Maulide**: conceptualization, supervision, writing – review and editing, and resources.

## Conflicts of Interest

The authors declare no conflicts of interest.

## Supporting information



The authors have cited additional references within the Supporting Information [50, 51, 52, 53, 54, 55, 56, 57, 58, 59, 60, 61, 62, 63, 64, 65, 66, 67, 68, 69, 70, 71, 72, 73, 74, 75, 76]. **Supporting File**: anie74563‐sup‐0001‐SuppMat.pdf.

## Data Availability

The data that supports the findings of this study are available in the Supporting Information of this article.
