# Supplementary material for: Deprotective Alkylation: A Platform for Alcohol‐to‐Amine Interconversion Using Nonafluoromesitylenesulfonamides
Source: Angew Chem Int Ed Engl. 2026 Sep 8;65(43):e3520541. doi: 10.1002/anie.3520541 (PMC13676391; doi:10.1002/anie.3520541)
Supplement: Supplementary file 1 [file ANIE-65-e3520541-s001.pdf]

Deprotective Alkylation: A Platform for Alcohol-to-Amine Interconversion using Nonafluoromesitylenesulfonamides

Jakub Brześkiewicz[a], Kunj Kapur[a], Boris Maryasin[a] and Nuno Maulide*[a]

[a] Institute of Organic Chemistry, University of Vienna, Währinger Straße 38, 1090 Vienna (Austria)
E-Mail: nuno.maulide@univie.ac.at, Homepage: http://maulide.univie.ac.at

Table of Contents

1. General Information ... 2
2. Optimization ... 3
   2.1 Solvents ... 3
   2.2 Bases ... 3
   2.3 Additives ... 4
   2.4 Enantiomeric excess ... 4
   2.5 Primary alcohols ... 5
   2.6 *N*-Methylation ... 6
   2.7 Secondary Nms-amides ... 6
3. Experimental procedures and characterization data ... 8
   3.1 Alcohol synthesis ... 8
   3.2 Nms-amide synthesis ... 20
   3.3 Amine synthesis through deprotective alkylation ... 27
   3.4 Experimental evidence for stereoinversion ... 53
   3.5 Scale-up procedure ... 56
   3.6 Unsuccessful and low-yielding examples ... 58
   3.7 Penfluridol synthesis ... 59
   3.8 Comparison with BEHT triflate ... 63
   3.9 Synthesis of isotope labelled amines ... 64
4. Mechanistic studies ... 68
   4.1 Intermediate isolation ... 68
   4.2 Comparison with other protecting groups ... 70
   4.3 DFT calculations ... 72
5. NMR spectra ... 75
6. References ... 170

1. General Information

Unless otherwise stated, all glassware was flame-dried before use and all reactions were performed under an atmosphere of argon. All solvents were distilled from appropriate drying agents prior to use or directly taken from commercial sealed bottles under an atmosphere of argon. All reagents were used as received from commercial suppliers unless otherwise stated. Lithium *tert*-butoxide (*t*-BuOLi) was purchased from abcr GmbH and stored in a glovebox under an atmosphere of argon. Reaction progress was monitored by thin layer chromatography (TLC) performed on aluminium plates coated with silica gel F254 with 0.2 mm thickness. Chromatograms were visualized by fluorescence quenching with UV light at 254 nm or by staining using potassium permanganate, followed by heating. Microwave (MW) irradiation experiments were carried out with a CEM Discover SP in a 10 mL vessel with 1.5 mL of the corresponding solvent. Flash column chromatography was performed using silica gel 60 (230-400 mesh, 0.040-0.063 mm Merck and co.) and silica gel RS SiOH (15-40 µm). Neat infrared spectra were recorded using a Perkin-Elmer Spectrum 100 FT-IR spectrometer. Wavenumbers (ν_{max}) are reported in cm^{-1}. Mass spectra were obtained on a Bruker maXis UHR-TOF (QQ-TOF) spectrometer, using electrospray ionization (ESI) or an Agilent 7200B GC/Q-TOF spectrometer, using electron ionization (EI). Optical rotations were measured on a Perkin Elmer 341 polarimeter using a 50 mm path-length cell at 589 nm (c given in g/100 mL). Chiral HPLC was performed using AGILENT Infinity 1260 with Chiralcel OD-H, Lux-Cellulose-1 and Lux-Cellulose-3 columns. Details on chromatographic conditions are indicated under each compound. All ^{1}H NMR, ^{13}C NMR, ^{31}P NMR and ^{19}F NMR spectra were recorded using Bruker AV-400, AV-500, AV-600 or AV-700 spectrometers at 298 K, unless stated otherwise. Chemical shifts (δ) are given in parts per million (ppm) and referenced to the given solvent peak as described in literature.[50] Coupling constants are quoted in Hz (*J*). ^{1}H NMR splitting patterns are designated as singlet (s), doublet (d), triplet (t), quartet (q), pentet (p), heptet (hept). ^{1}H NMR splitting patterns are reported as they appeared in the spectrum. If the appearance of a signal differs from the expected splitting pattern, the observed pattern is designated as apparent (app). Splitting patterns that could not be interpreted or easily visualized are designated as multiplet (m) or broad (br).

2. Optimization

2.1 Solvents

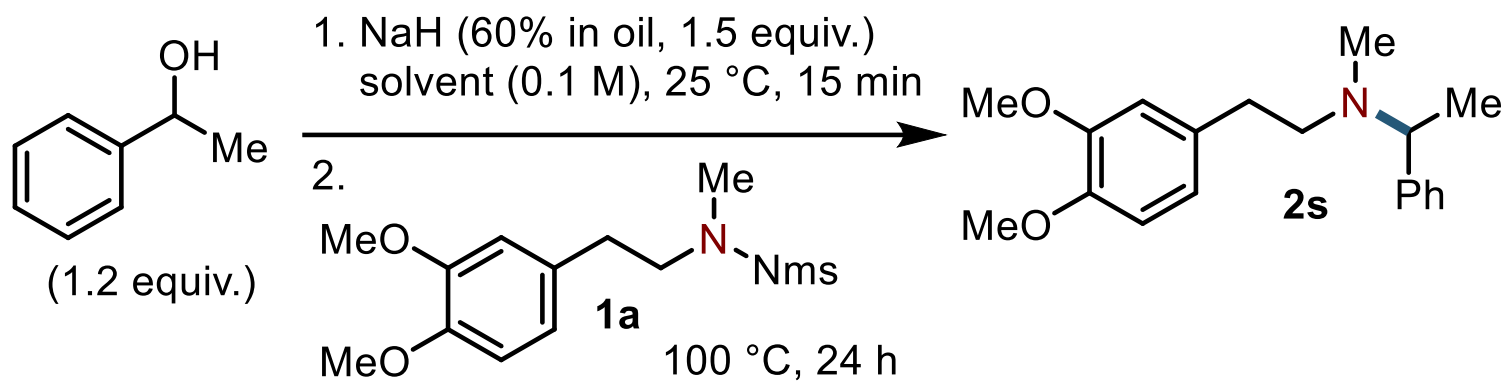

entry	solvent	NMR yield (%)
**1**	**THF**	**52**
2	PhMe	29
3	1,4-dioxane	39
4	DME	32
5	CPME	50
6	(*n*-Bu)$_2$O	34
7[a]	*t*-BuOH	-

Table S1. Solvent optimization. [a] *t*-BuOLi was used as a base. NMR yields were determined using CH_2Br_2 as an internal standard.

2.2 Bases

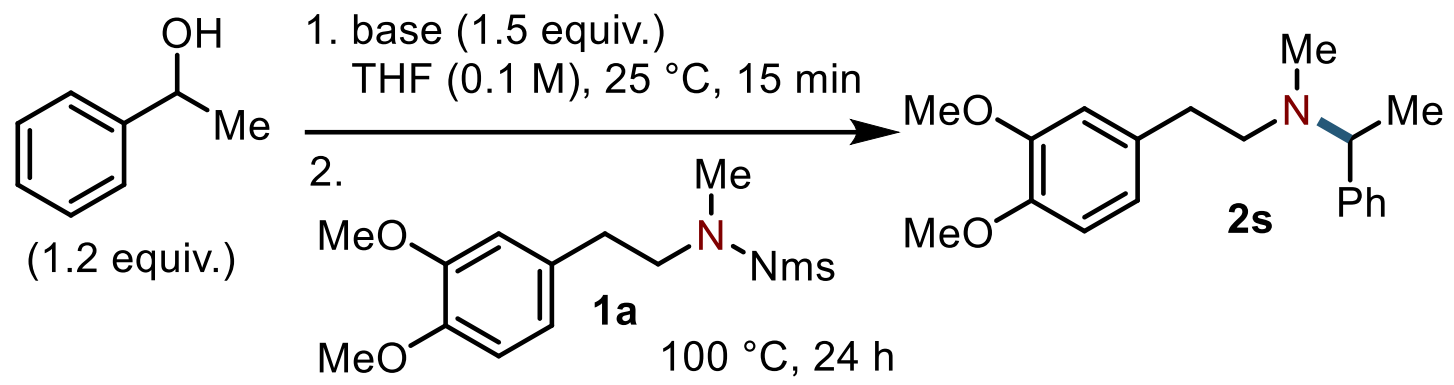

entry	base	NMR yield (%)
1	KH	-
2	DBU	-
3	Cs_2CO_3	traces
4	*t*-BuOK	62
5	NaHMDS	40
6[a]	*t*-BuOK	43
7	*t*-BuONa	42
**8**	***t*-BuOLi**	**64**

Table S2. Base optimization. [a] 1 equiv. of H_2O was added. NMR yields were determined using CH_2Br_2 as an internal standard.

2.3 Additives

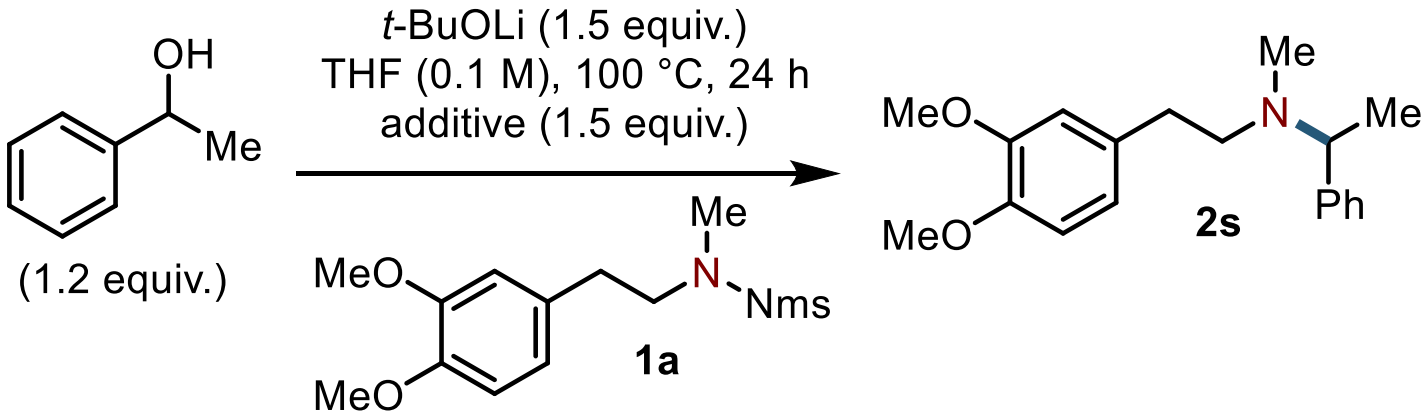

entry	additive	NMR yield (%)
**1**	**LiI**	**82**
2	LiBr	34
3	TBAB	78
4	CsI	59
5	CsF	71
6	MgI_2	-
7	12-crown-4	62

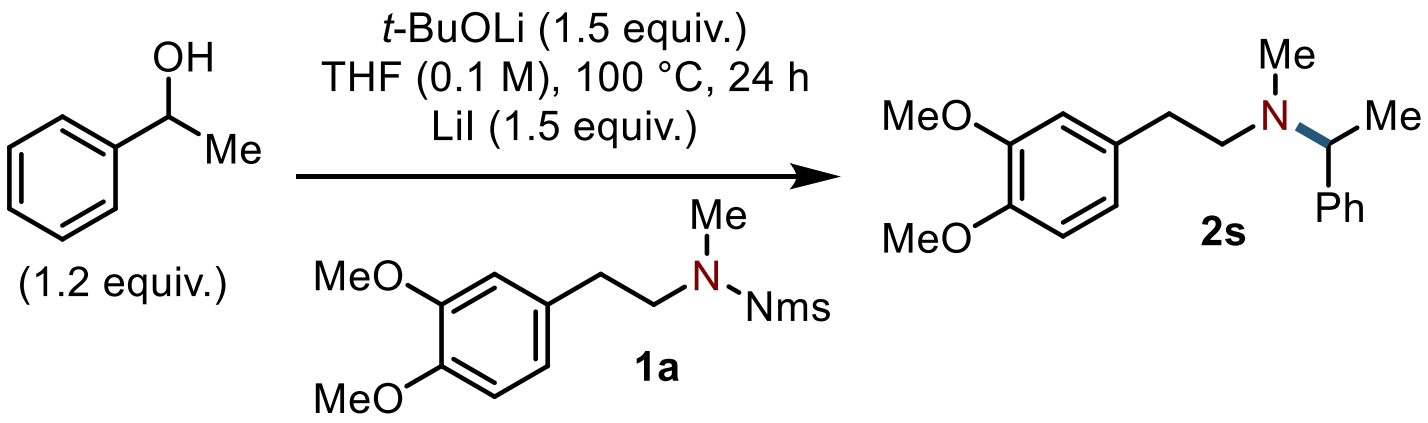

entry	deviation	NMR yield (%)
8	temp. 120 °C	41
9	3 h in MW reactor	57
10	LiI (1.5 equiv.), 1 h in MW reactor (100 °C)	44
11	LiI (1.5 equiv.), concentration 0.2 M	75
12	LiI (1.5 equiv.), concentration 0.05 M	76

Table S3. Additive optimization. NMR yields were determined using CH_2Br_2 as an internal standard.

2.4 Enantiomeric excess

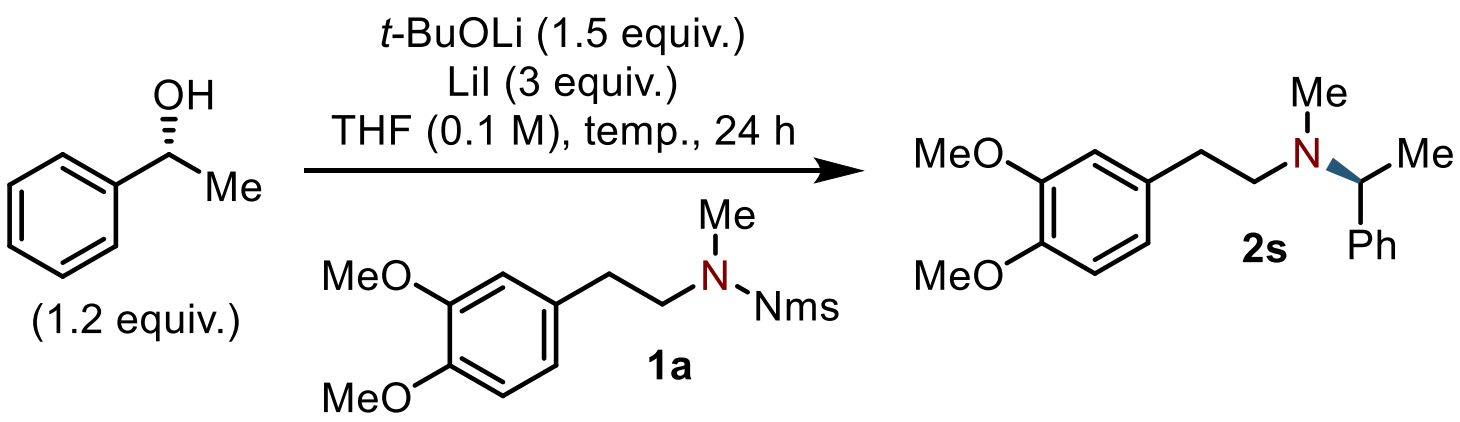

entry	temperature (°C)	NMR yield (%)	*e.r.*
1	110	85	50:50
2	90	82	50:50
**3**	**80**	**83 (68)[a]**	50:50
4	70	80	50:50

Table S4.1. Enantiomeric ratio optimization with LiI. [a] Isolated yield. NMR yields were determined using CH_2Br_2 as an internal standard.

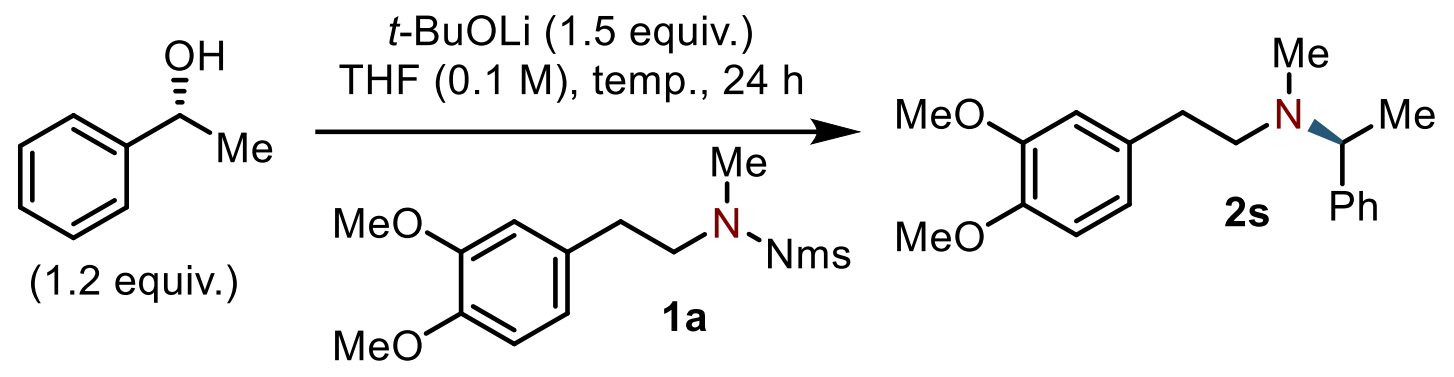

entry	temperature (°C)	NMR yield (%)	*e.r.*
1	100	58	92:8
2	90	59	94:6
**3**	**80**	**55 (47)[a]**	**96:4**
4	70	40	98:2

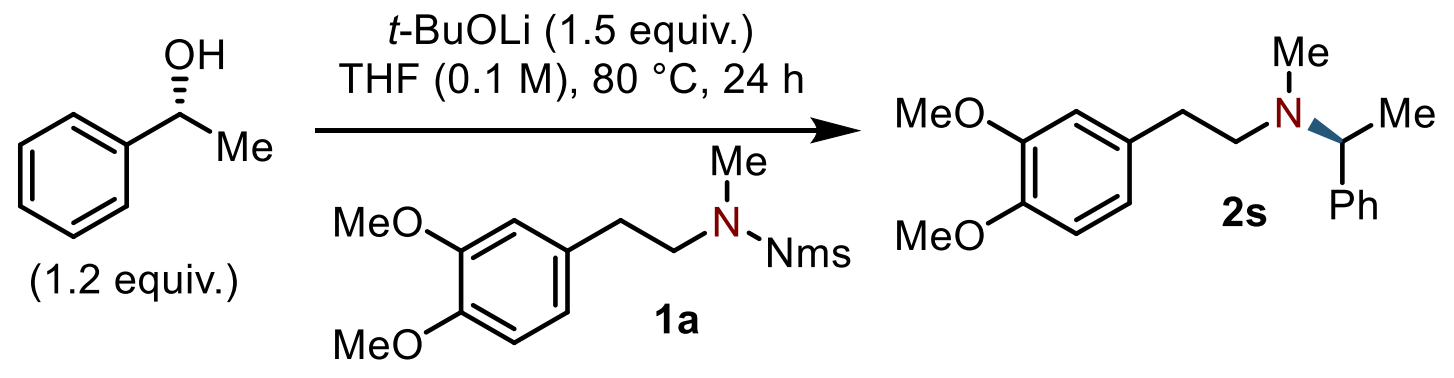

entry	deviation	NMR yield (%)	*e.r.*
5	0.05 M THF conc., 48 h reaction time	54	95:5
6	48 h reaction time	58	94:6
**7**	**72 h reaction time**	**73 (67)[a]**	**94:6**
8	72 h reaction time, 2 equiv. of alcohol	64	94:6

Table S4.2. Enantiomeric ratio optimization without LiI. [a] Isolated yield. NMR yields were determined using CH_2Br_2 as an internal standard.

2.5 Primary alcohols

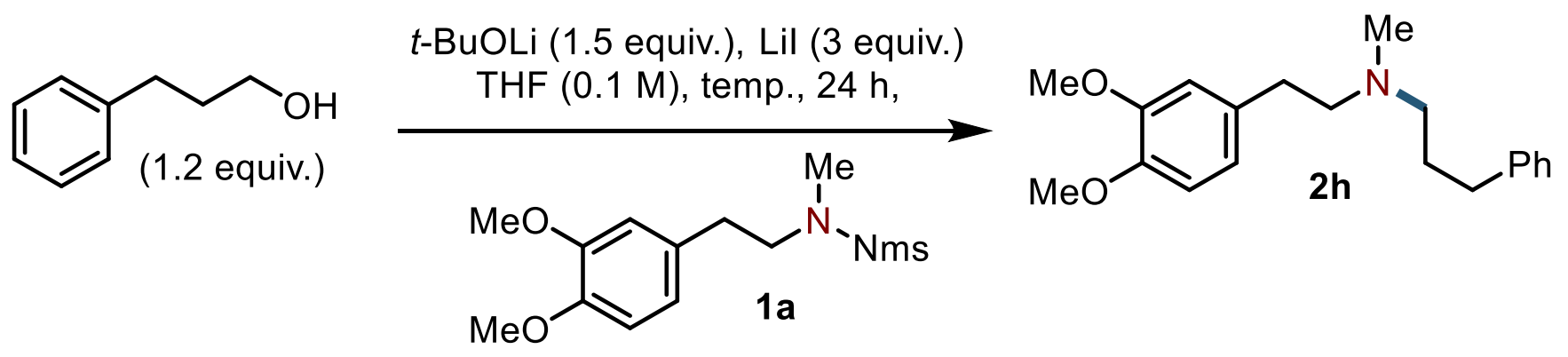

entry	temperature (°C)	NMR yield (%)
1	120	43
2	110	53
3	100	62
4	90	74
**5**	**80**	**83 (86)[a]**
6	80	67[b]
7	80	28[c]
8	80	36[c,d]
9	70	82
10	60	65

Table S5. Primary alcohol optimization. [a] Isolated yield. [b] 1.5 equiv. of LiI was used. [c] Reaction performed with 20 mol% of LiI. [d] Reaction performed for 72 h. NMR yields were determined using CH_2Br_2 as an internal standard.

2.6 *N*-Methylation

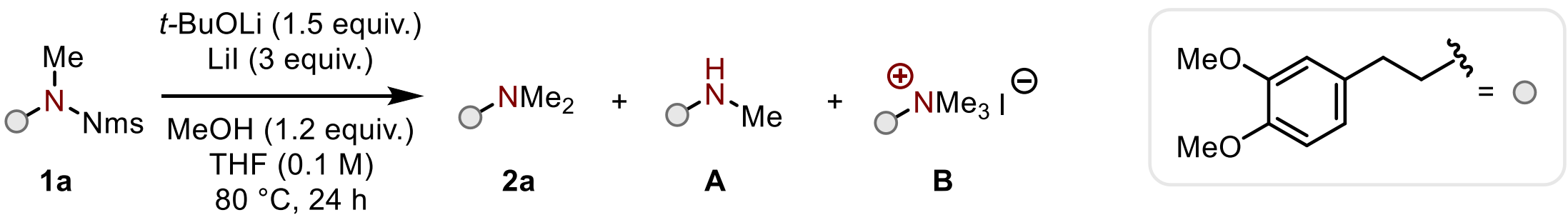

entry	deviation	2a NMR yield (%)	A NMR yield (%)	B NMR yield (%)	SM (%)
1	-	43	15	32	10
2	MeOLi (1.2 equiv.) instead of MeOH/*t*-BuOLi	18	14	14	54
3	MeOH (1.5 equiv.) was used at 60 °C	31	22	18	28
4	MeOH (1.5 equiv.) was used	33	15	20	29
5	MeOH (1.5 equiv.) in THF (0.05 M)	35	12	18	34
6	**LiI (20 mol%)**	**46 (51)**[a]	**20**	**27**	**<5**

Table S6. *N*-Methylation optimization. [a] Isolated yield. NMR yields were determined using CH_2Br_2 as an internal standard.

2.7 Secondary Nms-amides

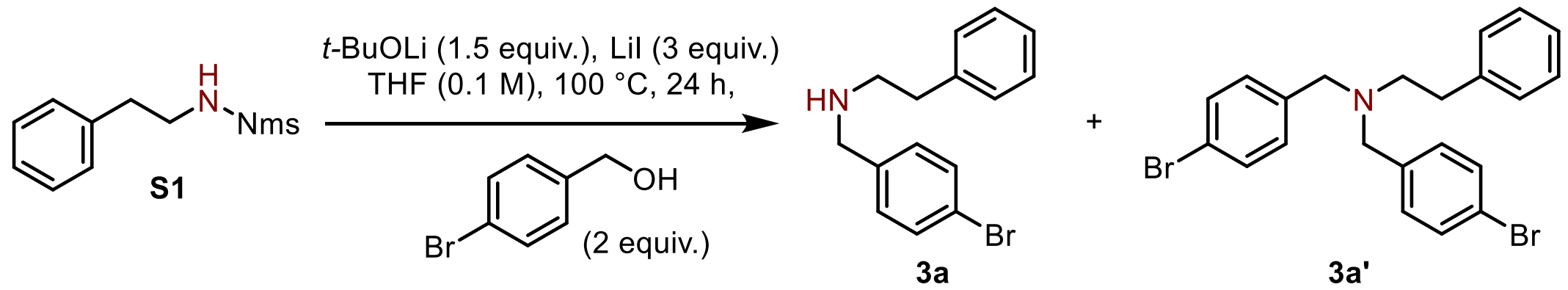

entry	deviation	3a NMR yield (%)	3a' NMR yield (%)	SM (%)
1	-	29	19	39
2	3 equiv. of alcohol	traces	31	30
3	NaH (1.5 equiv.) instead of *t*-BuOLi	18	44	28
4	*t*-BuOK (1.5 equiv.) instead of *t*-BuOLi	28	23	20
5	PhMe instead of THF	0	0	98
6	temp. 110 °C	26	24	33
7	concentration 0.2 M	13	33	40
**8**	**TMSOK (1.5 equiv.) instead of *t*-BuOL**i	**70**	8	**13**
9	*t*-AmOK (1.5 equiv.) instead of *t*-BuOLi	27	28	33
10	NaHMDS (1.5 equiv.) instead of *t*-BuOLi	40	18	5
11	w/o LiI	37	5	8
12	LiBr instead of LiI	20	21	32
13	LiCl instead of LiI	13	5	9

Table S7.1. Secondary Nms-amides optimization. NMR yields were determined using CH_2Br_2 as an internal standard.

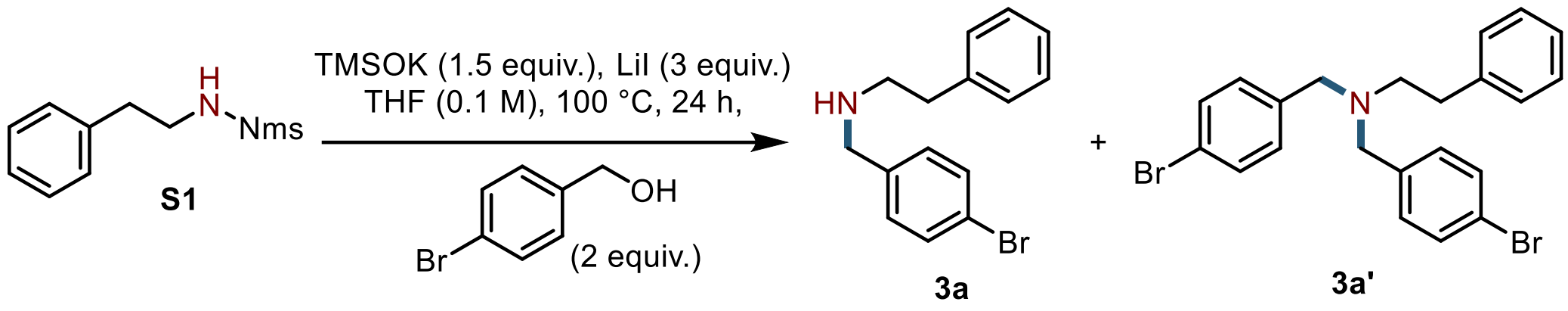

entry	deviation	3a NMR yield (%)	3a' NMR yield (%)	SM (%)
1	temp. 90 °C	62	6	26
2	temp. 80 °C	58	3	40
3	w/o LiI	0	0	0
4	reaction time 48 h	70	20	0
5	KI instead of LiI	0	0	0
**6**	**2 equiv. of TMSOK**	**78 (76)[a]**	**8**	**12**
7	TMSOLi (1.5 equiv.) instead of TMSOK	30	29	27

Table S7.2. Secondary Nms-amides optimization. [a] Isolated yield. NMR yields were determined using CH_2Br_2 as an internal standard.

3. Experimental procedures and characterization data

3.1 Alcohol synthesis

***N*-(2-(((*tert*-Butyldimethylsilyl)oxy)methyl)benzyl)-*N*-(3,4-dimethoxyphenethyl)-2,4,6-tris(trifluoromethyl)benzenesulfonamide (S2)**

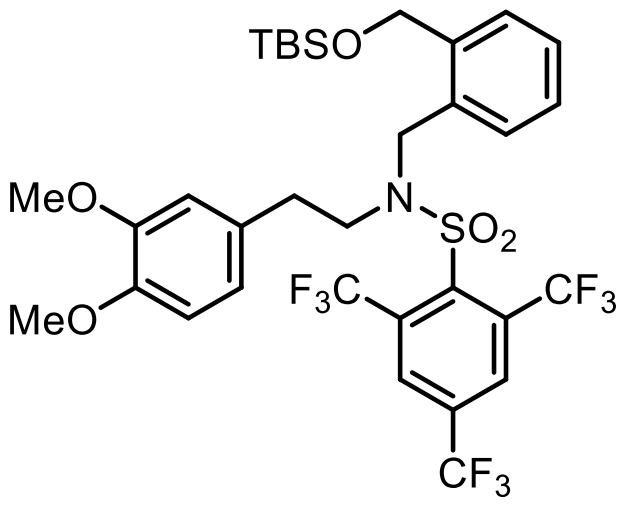

Under an argon atmosphere, a flame dried Schlenk flask was charged with anhydrous solvents (toluene/THF 4:1, 6.0 mL) and cooled to 0 °C before adding triphenylphosphine (393 mg, 1.50 mmol, 1.50 equiv.) and diizopropyl azodicarboxylate (DIAD, 0.295 mL, 1.50 mmol, 1.50 equiv.). After 15 min at 0 °C, the reaction mixture solidified and a mixture of (2-(((*tert*-butyldimethylsilyl)oxy)methyl)phenyl)methanol[51] (379 mg, 1.50 mmol, 1.50 equiv.) and *N*-(3,4-dimethoxyphenethyl)-2,4,6-tris(trifluoromethyl)benzenesulfonamide[34] (525 mg, 1.00 mmol, 1.00 equiv.) in toluene/THF (4:1, 2.0 mL) was added via syringe. The resulting mixture became a clear solution and was stirred at room temperature (25 °C) for 16 h. After that time, the solvent was removed *in vacuo* and the residue was purified by flash column chromatography (silica gel, 0%-30% EtOAc in *n*-heptane) to afford the title product as a colourless oil (545 mg, 0.717 mmol, 72% yield).

^{1}H NMR (400 MHz, CDCl$_3$): δ 8.26 (s, 2H), 7.50 – 7.44 (m, 1H), 7.41 – 7.36 (m, 1H), 7.32 – 7.26 (m, 2H), 6.63 (d, *J* = 8.2 Hz, 1H), 6.44 (dd, *J* = 8.1, 1.8 Hz, 1H), 6.36 (d, *J* = 1.8 Hz, 1H), 4.80 (s, 2H), 4.79 (s, 2H), 3.78 (s, 3H), 3.76 (s, 3H), 3.39 – 3.30 (m, 2H), 2.55 – 2.46 (m, 2H), 0.93 (s, 9H), 0.10 (s, 6H) ppm.

^{13}C NMR (101 MHz, CDCl$_3$): δ 148.9, 147.8, 145.5, 139.3, 134.2 (q, $^{2}J_{C-F}$ = 35.1 Hz), 133.6, 133.2 (q, $^{2}J_{C-F}$ = 33.7 Hz, 2C), 130.5, 129.5 (m, 2C), 129.1, 128.3, 128.2, 128.0, 122.2 (q, $^{1}J_{C-F}$ = 275.8 Hz, 2C), 122.0 (q, $^{1}J_{C-F}$ = 273.5 Hz), 120.7, 112.0, 111.2, 63.7, 55.9, 55.8, 50.5, 50.3, 34.4, 26.0 (3C), 18.5, -5.2 (2C) ppm.

^{19}F NMR (376 MHz, CDCl$_3$): δ -54.90 (s, 6F), -63.58 (s, 3F) ppm.

IR (neat) ν_{max}: 2933, 1516, 1363, 1271, 1181, 1141, 1075, 1030, 914, 835 cm^{-1}.

HRMS (ESI$^+$): exact mass calculated for $[M+Na]^+$ $(C_{33}H_{38}F_9NO_5{}^{32}S^{28}Si^{23}Na)^+$ requires *m/z* 782.1988, found *m/z* 782.1989.

Rf: 0.44 *n*-heptane:EtOAc 5:1.

N-(3,4-Dimethoxyphenethyl)-N-(2-(hydroxymethyl)benzyl)-2,4,6-tris(trifluoromethyl)benzenesulfonamide (S3)

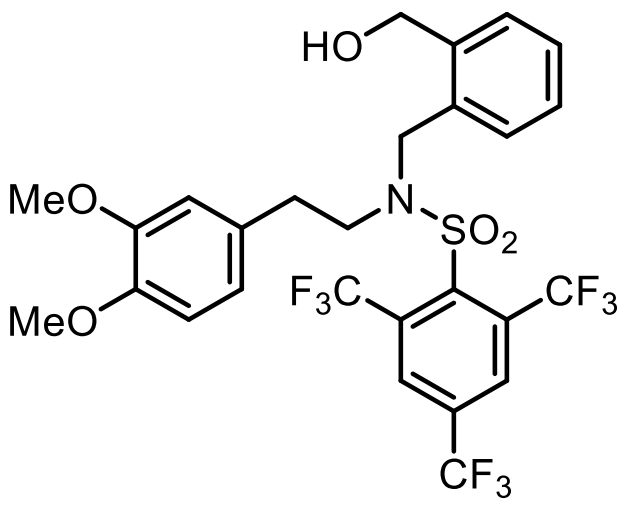

To a stirred solution of Nms-amide **S2** (513 mg, 0.675 mmol, 1.00 equiv.) in methanol (6.8 mL) was added (+)-10-camphorsulfonic acid (31.4 mg, 0.135 mmol, 0.20 equiv.) at 0 °C. After stirring for 16 h at room temperature (25 °C), the resulting mixture was quenched with sat. aq. $NaHCO_3$ (20 mL) and extracted with EtOAc (3 × 20 mL). The combined organic layer was dried over anhydrous Na_2SO_4 and filtered. The filtrate was concentrated *in vacuo*, and the residue was purified by flash column chromatography (silica gel, 20%-60% EtOAc in *n*-heptane) to afford the title product as a pale-yellow oil (362 mg, 0.561 mmol, 83% yield).

^{1}H NMR (400 MHz, $CDCl_3$): δ 8.26 (s, 2H), 7.53 – 7.45 (m, 1H), 7.40 – 7.28 (m, 3H), 6.63 (d, *J* = 8.1 Hz, 1H), 6.49 – 6.35 (m, 2H), 4.84 (s, 2H), 4.79 (d, *J* = 4.9 Hz, 2H), 3.78 (s, 3H), 3.76 (s, 3H), 3.42 – 3.30 (m, 2H), 2.56 – 2.43 (m, 2H), 1.93 (t, *J* = 5.2 Hz, 1H) ppm.

^{13}C NMR (101 MHz, $CDCl_3$): δ 148.9, 147.8, 145.3, 139.0, 134.3 (q, $^2J_{C\text{-}F}$ = 34.7 Hz), 134.2, 133.2 (q, $^2J_{C\text{-}F}$ = 33.7 Hz, 2C), 130.5, 130.0, 129.5 (m, 2C), 129.5, 128.7, 128.6, 122.2 (q, $^1J_{C\text{-}F}$ = 275.7 Hz, 2C), 122.0 (q, $^1J_{C\text{-}F}$ = 273.6 Hz), 120.7, 112.0, 111.2, 63.3, 55.9, 55.8, 50.4, 50.3, 34.3 ppm.

^{19}F NMR (376 MHz, $CDCl_3$): δ -54.93 (s, 6F), -63.59 (s, 3F) ppm.

IR (neat) ν_{max}: 3518, 2940, 1516, 1366, 1271, 1178, 1139, 913, 730 cm^{-1}.

HRMS (ESI$^+$): exact mass calculated for $[M+Na]^+$ $(C_{27}H_{24}F_9NO_5{}^{32}S^{23}Na)^+$ requires *m/z* 668.1124, found *m/z* 668.1115.

Rf: 0.31 *n*-heptane:EtOAc 2:1.

(*E*)-2-(5-Fluoro-2-methyl-1-(4-(methylsulfinyl)benzylidene)-1*H*-inden-3-yl)ethan-1-ol (S4)

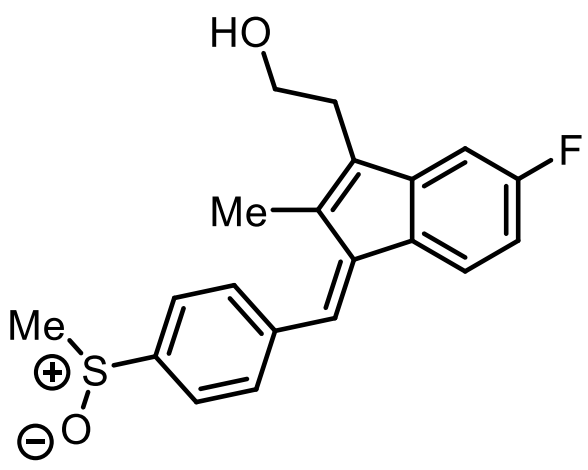

1,1'-Carbonyldiimidazole (178 mg, 1.10 mmol, 1.10 equiv.) was added portion-wise to a solution of Sulindac (356 mg, 1.00 mmol, 1.00 equiv.) in tetrahydrofuran (4.0 mL) at room temperature (25 °C). The reaction mixture was stirred for 1 h at the same temperature, followed by addition of sodium borohydride (113 mg, 3.00 mmol,

3.00 equiv.) in water (5.0 mL). The reaction was continued for 4 h at room temperature and partitioned between EtOAc (20 mL) and water (10 mL). The organic layer was separated and the aqueous layer was extracted with EtOAc (2 × 10 mL). The organic layers were combined, washed with brine (15 mL) and dried over anhydrous Na_2SO_4. After filtration, volatiles were removed *in vacuo* and the crude mixture was purified by flash column chromatography (silica gel, 100% EtOAc) to obtain the desired product as a yellow solid (234 mg, 0.683 mmol, 68% yield).

^{1}H NMR (400 MHz, $CDCl_3$): δ 7.71 (d, *J* = 8.2 Hz, 2H), 7.65 (d, *J* = 8.1 Hz, 2H), 7.16 – 7.09 (m, 2H), 6.86 (dd, *J* = 8.9, 2.4 Hz, 1H), 6.54 (td, *J* = 8.9, 2.3 Hz, 1H), 3.84 (q, *J* = 6.4 Hz, 2H), 2.84 (t, *J* = 6.7 Hz, 2H), 2.80 (s, 3H), 2.19 (s, 3H), 1.71 (s, 1H) ppm.

^{13}C NMR (101 MHz, $CDCl_3$): δ 163.5 (d, $^1J_{C\text{-}F}$ = 246.6 Hz), 147.4 (d, $^3J_{C\text{-}F}$ = 8.6 Hz), 145.4, 142.1, 140.0, 137.5, 136.1 (d, $^4J_{C\text{-}F}$ = 2.4 Hz), 130.4 (2C), 129.9 (d, $^4J_{C\text{-}F}$ = 2.9 Hz), 127.5 (d, $^5J_{C\text{-}F}$ = 1.9 Hz), 123.9 (2C), 123.8 (d, $^3J_{C\text{-}F}$ = 9.1 Hz), 110.7 (d, $^2J_{C\text{-}F}$ = 22.6 Hz), 106.0 (d, $^2J_{C\text{-}F}$ = 23.6 Hz), 61.4, 44.0, 29.3, 10.6 ppm.

^{19}F NMR (376 MHz, $CDCl_3$): δ -112.98 – -113.08 (m, 1F) ppm.

IR (neat) ν_{max}: 3352, 2916, 1708, 1600, 1466, 1362, 1030, 1012, 852, 813 cm^{-1}.

HRMS (ESI$^+$): exact mass calculated for $[M+H]^+$ $(C_{20}H_{20}FO_2{}^{32}S)^+$ requires *m/z* 343.1163, found *m/z* 343.1155.

Rf: 0.34 EtOAc.

5-(5-(Hydroxymethyl)-4-methylthiazol-2-yl)-2-isobutoxybenzonitrile (S5)

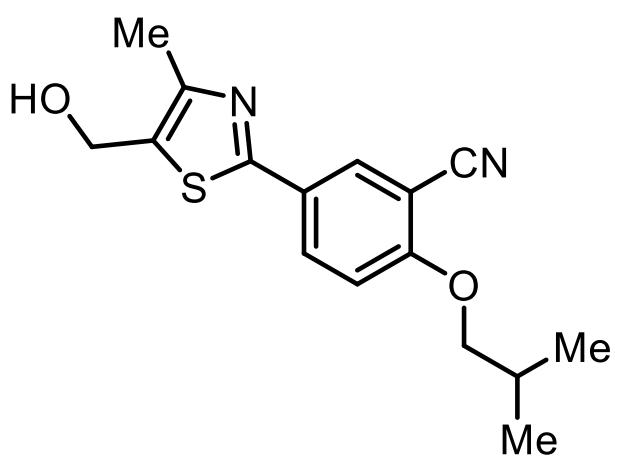

1,1'-Carbonyldiimidazole (178 mg, 1.10 mmol, 1.10 equiv.) was added portion-wise to a solution of Febuxostat (316 mg, 1.00 mmol, 1.00 equiv.) in tetrahydrofuran (4.0 mL) at room temperature (25 °C). The reaction mixture was stirred for 1 h at the same temperature, followed by addition of sodium borohydride (113 mg, 3.00 mmol, 3.00 equiv.) in water (5.0 mL). The reaction was continued for 4 h at room temperature and partitioned between EtOAc (20 mL) and water (10 mL). The organic layer was separated and the aqueous layer was extracted with EtOAc (2 × 10 mL). The organic layers were combined, washed with brine (15 mL) and dried over anhydrous Na_2SO_4. After filtration, volatiles were removed *in vacuo* and the crude mixture was purified by flash column chromatography (silica gel, 50%-100% EtOAc in *n*-heptane) to obtain the desired product as a colourless solid (232 mg, 0.769 mmol, 77% yield).

^{1}H NMR (400 MHz, $CDCl_3$): δ 8.07 (d, *J* = 2.2 Hz, 1H), 8.00 (dd, *J* = 8.8, 2.3 Hz, 1H), 6.97 (d, *J* = 8.9 Hz, 1H), 4.81 (s, 2H), 3.87 (d, *J* = 6.5 Hz, 2H), 2.43 (s, 3H), 2.18 (dp, *J* = 13.3, 6.6 Hz, 1H), 1.08 (d, *J* = 6.8 Hz, 6H) ppm. *(The signal for OH group could not be detected)*.

^{13}C NMR (101 MHz, CDCl$_3$): δ 163.5, 161.8, 150.5, 132.1, 131.5, 131.3, 126.8, 115.7, 112.6, 102.7, 75.6, 56.8, 28.2, 19.1 (2C), 15.1 ppm.

IR (neat) ν$_{max}$: 3267, 2961, 2874, 2227, 1606, 1507, 1445, 1277, 1119, 1008, 820 cm^{-1}.

HRMS (ESI$^+$): exact mass calculated for $[M+H]^+$ $(C_{16}H_{19}N_2O_2{}^{32}S)^+$ requires *m/z* 303.1162, found *m/z* 303.1156.

Rf: 0.36 *n*-heptane:EtOAc 1:1.

(4-(5-(2-Fluorophenyl)-1,2,4-oxadiazol-3-yl)phenyl)methanol (S6)

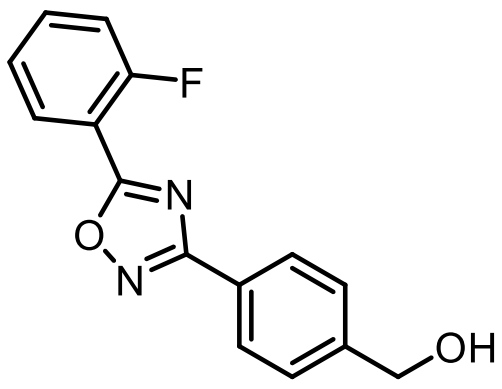

1,1’-Carbonyldiimidazole (178 mg, 1.10 mmol, 1.10 equiv.) was added portion-wise to a solution of Ataluren (284 mg, 1.00 mmol, 1.00 equiv.) in THF (4.0 mL) at room temperature (25 °C). The reaction mixture was stirred for 1 h at the same temperature, followed by addition of sodium borohydride (113 mg, 3.00 mmol, 3.00 equiv.) in water (5.0 mL). The reaction was continued for 4 h at room temperature and partitioned between EtOAc (20 mL) and water (10 mL). The organic layer was separated and the aqueous layer was extracted with EtOAc (2 × 10 mL). The organic layers were combined, washed with brine (15 mL) and dried over anhydrous Na_2SO_4. After filtration, volatiles were removed *in vacuo* and the crude mixture was purified by flash column chromatography (silica gel, 20-50% EtOAc in *n*-heptane) to obtain the desired product as a colourless solid (204 mg, 0.755 mmol, 76% yield).

^{1}H NMR (400 MHz, CDCl$_3$): δ 8.27 – 8.12 (m, 2H), 8.08 (d, *J* = 7.1 Hz, 1H), 7.66 – 7.45 (m, 3H), 7.38 – 7.24 (m, 2H), 4.78 (s, 2H), 2.36 (s, 1H) ppm.

^{13}C NMR (101 MHz, CDCl$_3$): δ 172.9, 168.7, 160.9 (d, $^1J_{C-F}$= 260.5 Hz), 141.9, 134.7 (d, $^3J_{C-F}$ = 8.5 Hz), 131.0, 129.9, 129.2, 127.0, 126.8, 126.1, 124.8 (d, $^3J_{C-F}$ = 3.8 Hz), 117.3 (d, $^2J_{C-F}$ = 20.9 Hz), 112.9 (d, $^2J_{C-F}$ = 11.4 Hz), 64.9 ppm.

^{19}F NMR (376 MHz, CDCl$_3$): δ -108.11 – -108.40 (m, 1F) ppm.

IR (neat) ν$_{max}$: 3347, 2928, 2877, 1619, 1420, 1355, 1023, 753, 700 cm^{-1}.

HRMS (ESI$^+$): exact mass calculated for $[M+Na]^+$ $(C_{15}H_{11}FN_2O_2{}^{23}Na)^+$ requires *m/z* 293.0697, found *m/z* 293.0695.

Rf: 0.24 *n*-heptane:EtOAc 2:1.

(*R*)-4-(1-Hydroxyethyl)-*N*,*N*-dipropylbenzenesulfonamide (S7)

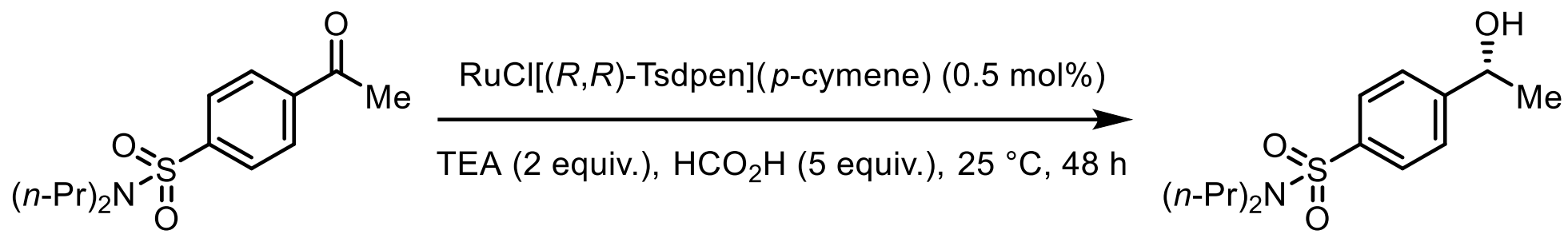

Formic acid (0.377 mL, 10.0 mmol, 5 equiv.) was added dropwise to a solution of 4-acetyl-*N*,*N*-dipropylbenzenesulfonamide[52] (567 mg, 2.00 mmol, 1.00 equiv.) in triethylamine (0.558 mL, 4.00 mmol, 2.00 equiv.) at 0 °C. The solution was allowed to warm to room temperature (25 °C) over 10 min. Subsequently, the Noyori-Ikariya catalyst ((RuCl[(*R*,*R*)-Tsdpen](*p*-cymene), 6.4 mg, 10 µmol, 0.5 mol%) was added in one portion and the resulting solution was stirred at room temperature for 48 h. After that time, the mixture was diluted with water and the aqueous layer was extracted with EtOAc (3 × 30 mL). The combined organic phases were washed with sat. aq. $NaHCO_3$ solution (30 mL), brine (30 mL), and dried over anhydrous Na_2SO_4. After filtration, volatiles were removed *in vacuo* and the crude mixture was purified by flash column chromatography (silica gel, 20-70% EtOAc in *n*-heptane) to obtain the desired product as a colourless solid (561 mg, 1.96 mmol, 98% yield).

^{1}H NMR (400 MHz, $CDCl_3$): δ 7.75 (d, *J* = 8.4 Hz, 2H), 7.49 (d, *J* = 8.2 Hz, 2H), 4.96 (qd, *J* = 6.4, 3.8 Hz, 1H), 3.09 – 3.02 (m, 4H), 2.06 (s, 1H), 1.62 – 1.47 (m, 7H), 0.87 (t, *J* = 7.4 Hz, 6H) ppm.

^{13}C NMR (101 MHz, $CDCl_3$): δ 150.5, 139.1, 127.4 (2C), 126.0 (2C), 69.8, 50.2 (2C), 25.6, 22.2 (2C), 11.3 (2C) ppm.

IR (neat) ν_{max}: 3515, 2968, 2934, 2876, 1459, 1330, 1152, 989, 571cm^{-1}.

HRMS (ESI$^+$): exact mass calculated for $[M+Na]^+$ $(C_{14}H_{23}NO_3{}^{32}S^{23}Na)^+$ requires *m/z* 308.1291, found *m/z* 308.1284.

Rf: 0.19 *n*-heptane:EtOAc 2:1.

$[\alpha]_D^{23}$ = 22.0 (*c* = 1.10, CH_2Cl_2).

Enantiomeric ratio: 96:4 determined by chiral HPLC analysis: Lux-Cellulose-3, (*n*-heptane + 0.1% IPA)/IPA 9:1, 0.7 mL/min, 25 °C, detection at 254 nm, retention time (min): 16.4 (major); 17.6 (minor).

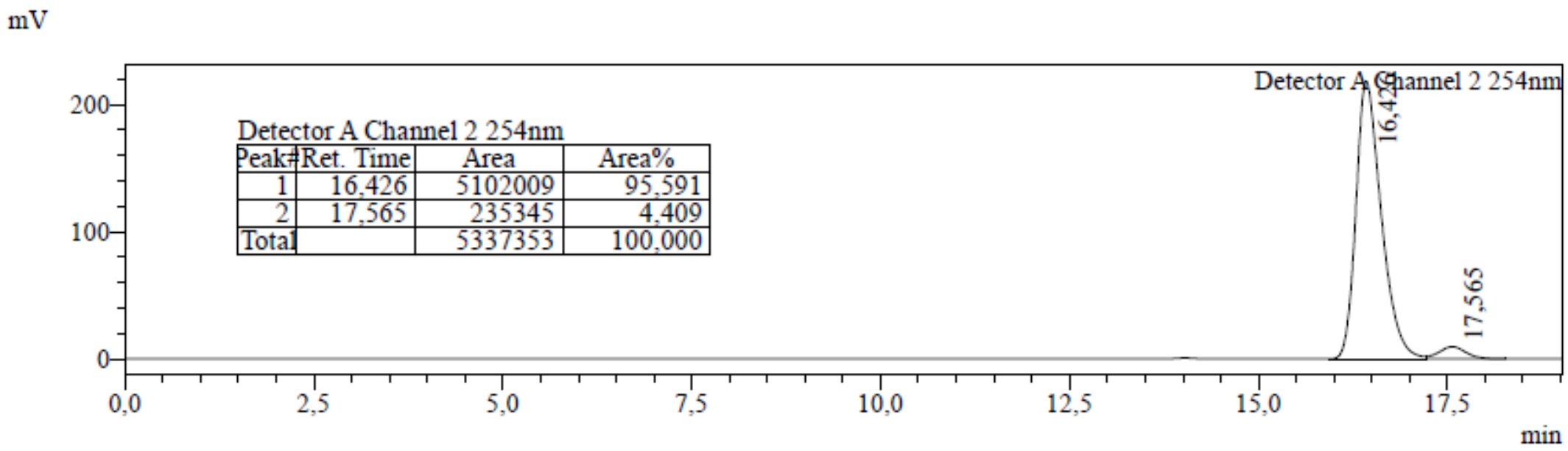

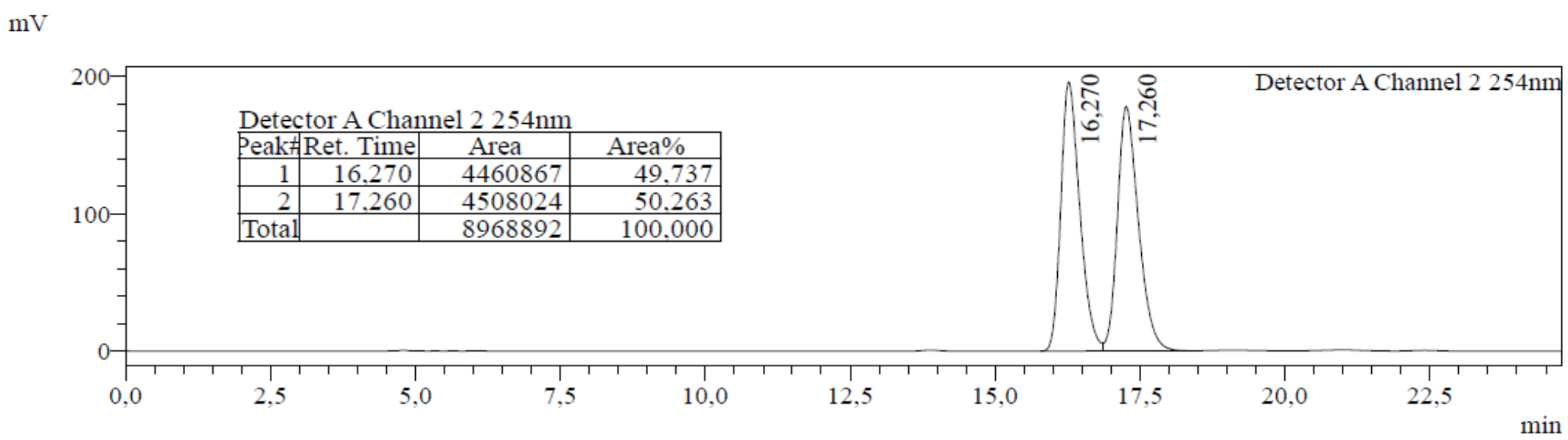

(*R*)-3-(Naphthalen-1-yloxy)-1-phenylpropan-1-ol (S8)

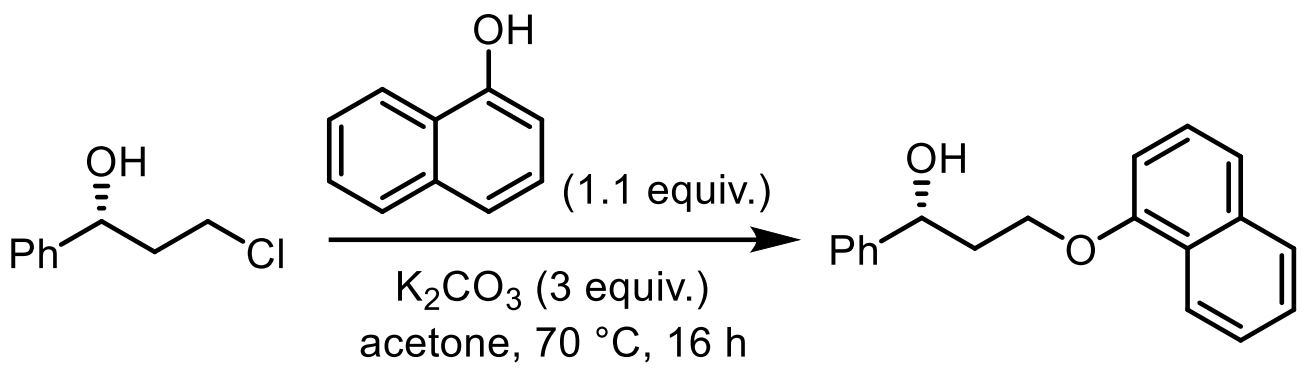

A modified procedure adopted from literature was used.[53] A mixture of (*R*)-3-chloro-1-phenylpropanol (853 mg, 5.00 mmol, 1.00 equiv.), 1-naphthol (793 mg, 5.50 mmol, 1.10 equiv.), and potassium carbonate (2.07 g, 15.00 mmol, 3.00 equiv.) in acetone (20 mL) was refluxed (70 °C) in an oil bath for 16 h. The reaction mixture was cooled to room temperature (25 °C), and the acetone was removed under reduced pressure. Then water (100 mL) was added, and the resulting mixture was extracted with ethyl acetate (3 × 50 mL), washed with brine (100 mL), dried over anhydrous Na_2SO_4, and concentrated *in vacuo*. The crude mixture was purified by flash column chromatography (silica gel, 10-25% EtOAc in *n*-heptane) to obtain the desired product as a white solid (664 mg, 2.39 mmol, 48% yield).

^{1}H NMR (400 MHz, $CDCl_3$): δ 8.28 – 8.23 (m, 1H), 7.84 – 7.78 (m, 1H), 7.55 – 7.48 (m, 2H), 7.46 – 7.41 (m, 3H), 7.41 – 7.34 (m, 3H), 7.33 – 7.28 (m, 1H), 6.81 (d, *J* = 7.6 Hz, 1H), 5.15 (dt, *J* = 8.2, 4.5 Hz, 1H), 4.40 – 4.32 (m, 1H), 4.25 – 4.17 (m, 1H), 2.48 – 2.29 (m, 3H) ppm.

All analytical data were in accordance with those reported in literature.[53]

Enantiomeric ratio: >99:1 determined by chiral HPLC analysis: Lux-Cellulose-1, (*n*-heptane + 0.1% IPA)/IPA 8:2, 1.0 mL/min, 25 °C, detection at 210 nm, retention time (min): 9.2 (major); 11.8 (minor).

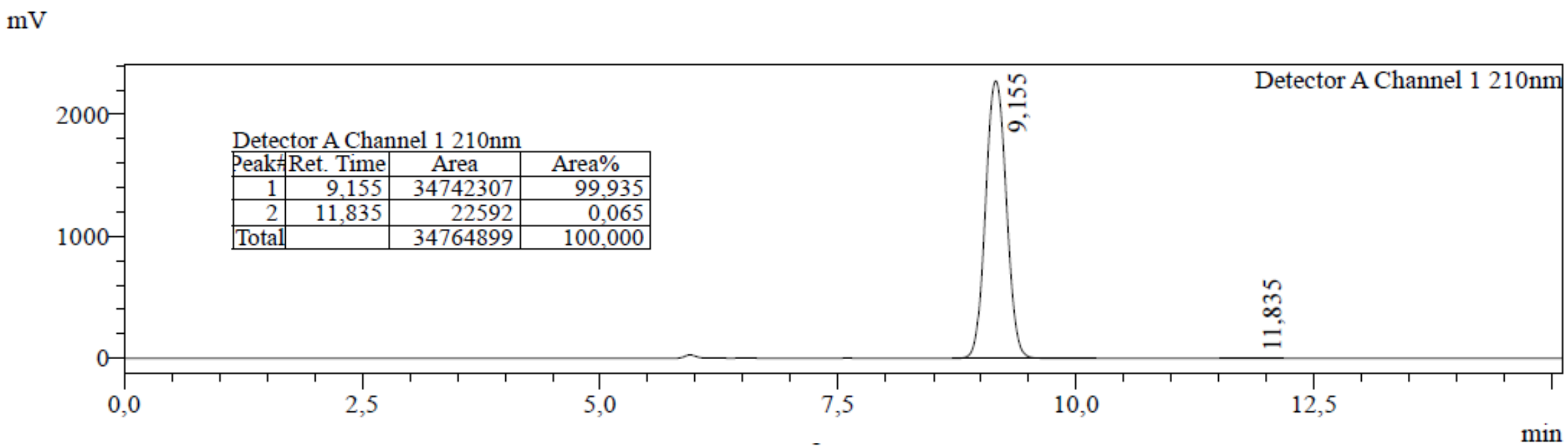

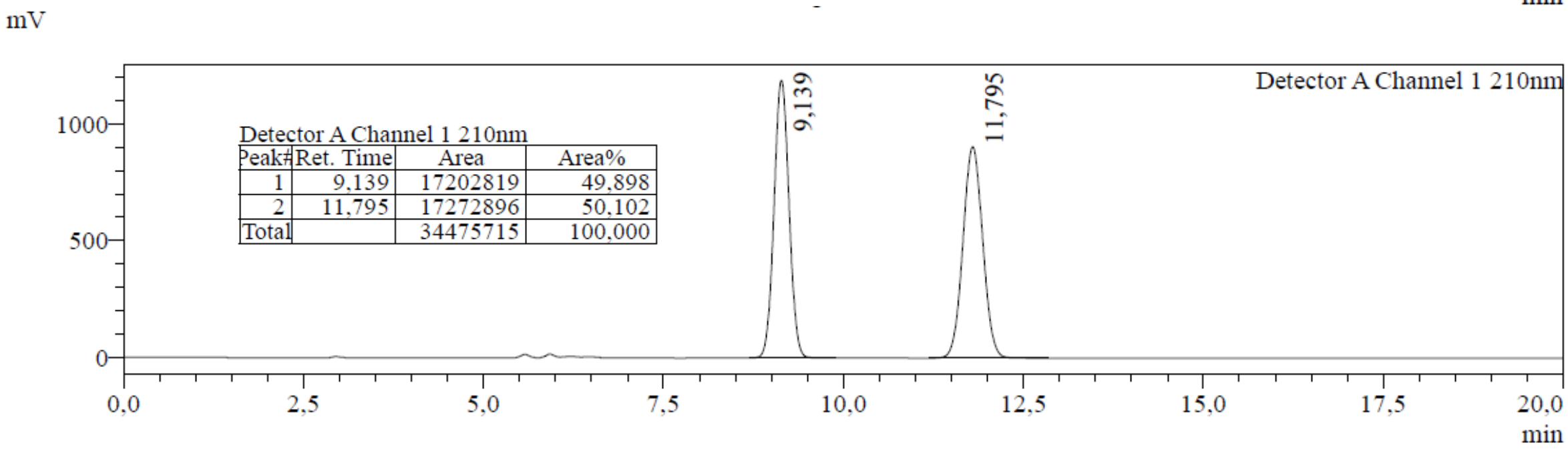

(*R*)-1-Phenylundec-4-yn-3-ol (S9)

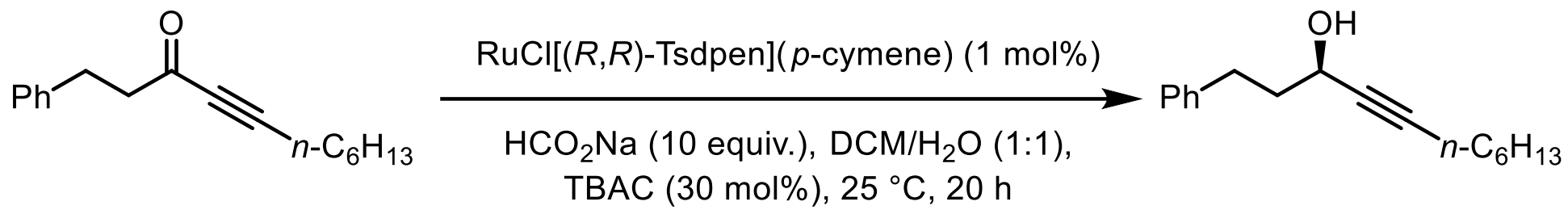

Noyori-Ikariya catalyst ((*R*,*R*)-Tsdpen-Ru(*p*-cymene)Cl, 3.7 mg, 6.0 µmol, 1 mol%) was added to a mixture of 1-phenylundec-4-yn-3-one[54] (140 mg, 0.578 mmol, 1.00 equiv.), sodium formate (393 mg, 0.173 mmol, 10.00 equiv.) and TBAC (48.2 mg, 0.173 mmol, 30 mol%) in DCM:water (1:1, 3.0 mL). The biphasic mixture was strongly stirred for 20 h at room temperature (25 °C). After completion, the crude reaction mixture was diluted with water (10 mL) and extracted with CH_2Cl_2 (2 x 20 mL). The organic layers were dried over anhydrous $mgSO_4$ and concentrated *in vacuo*. The resulting crude mixture was subjected to flash column chromatography (silica gel, 0%- 10% EtOAc in *n*-heptane) to obtain the title compound as a colourless oil (109 mg, 0.446 mmol, 77% yield).

^{1}H NMR (400 MHz, $CDCl_3$): δ 7.33 – 7.26 (m, 2H), 7.26 – 7.14 (m, 3H), 4.47 – 4.27 (m, 1H), 2.79 (t, *J* = 7.8 Hz, 2H), 2.23 (td, *J* = 7.1, 2.0 Hz, 2H), 2.09 – 1.91 (m, 2H), 1.72 (d, *J* = 4.8 Hz, 1H), 1.60 – 1.46 (m, 2H), 1.44 – 1.36 (m, 2H), 1.36 – 1.22 (m, 4H), 0.90 (t, *J* = 6.9 Hz, 3H) ppm.

All analytical data were in accordance with those reported in literature.[55]

Enantiomeric ratio: 98:2 determined by chiral HPLC analysis: Lux-Cellulose-1, *n*-heptane + 0.1% IPA:IPA 9:1, 1.0 mL/min, 25 °C, detection at 210 nm, retention time (min): 6.8 (minor); 8.6 (major).

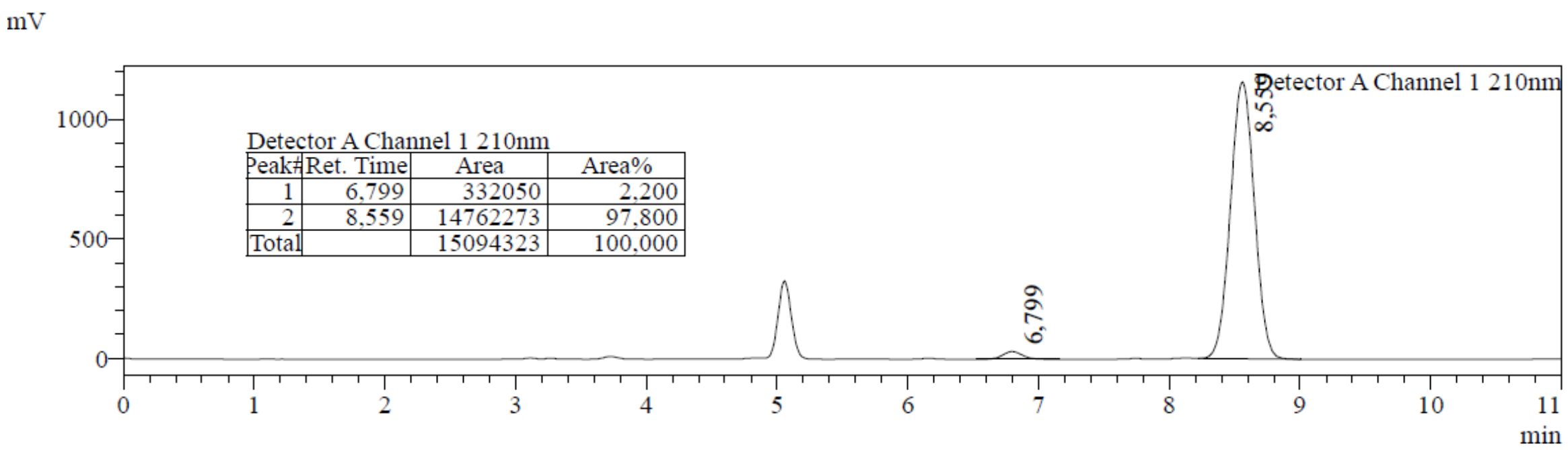

Detector A Channel 1 210nm

Peak#	Ret. Time	Area	Area%
1	6,799	332050	2,200
2	8,559	14762273	97,800
Total		15094323	100,000

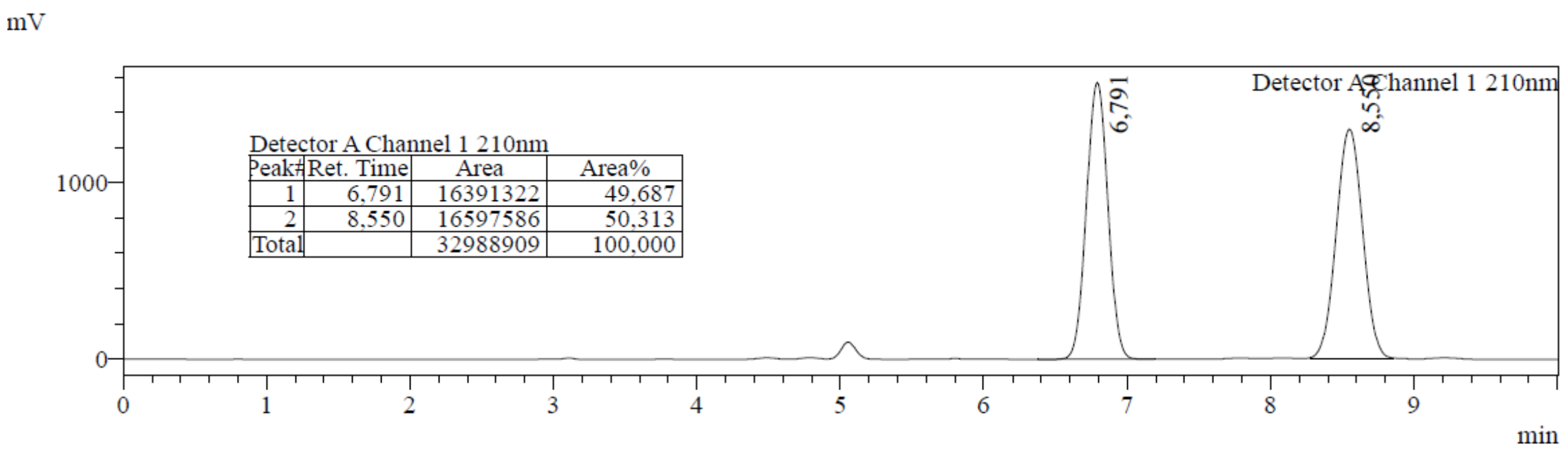

Detector A Channel 1 210nm

Peak#	Ret. Time	Area	Area%
1	6,791	16391322	49,687
2	8,550	16597586	50,313
Total		32988909	100,000

***tert*-Butyl 2-hydroxy-2-phenylacetate (S10)**

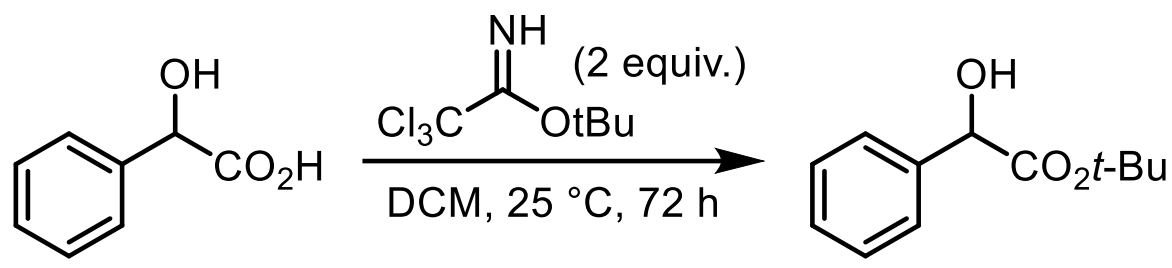

A modified procedure adopted from literature was used.[6] In a flame-dried flask, the *tert*-Butyl 2,2,2-trichloroacetimidate (0.536 mL, 3.0 mmol, 2 equiv.) was dissolved in dichloromethane (6 mL) under argon. The mandelic acid (228 mg, 1.5 mmol, 1 equiv.) was then added, and the mixture was stirred at room temperature (25 °C) for 72 h. After completion, the reaction mixture was poured into 2M NaOH and extracted with DCM (3 × 20 mL). The combined organic extracts were then dried over Na_2SO_4, filtered, and concentrated *in vacuo*. The resulting crude material was subjected to flash column chromatography (silica gel, 1%-20% EtOAc in *n*-heptane) to obtain the title compound as a colourless solid (221 mg, 1.06 mmol, 71% yield).

^{1}H NMR (400 MHz, $CDCl_3$): δ 7.43 – 7.28 (m, 5H), 5.04 (d, *J* = 5.9 Hz, 1H), 3.51 (d, *J* = 6.0 Hz, 1H), 1.41 (s, 9H) ppm.

All analytical data were in accordance with those reported in literature. [56]

(*R*)-3-(1-Hydroxyethyl)phenyl ethyl(methyl)carbamate (S11)

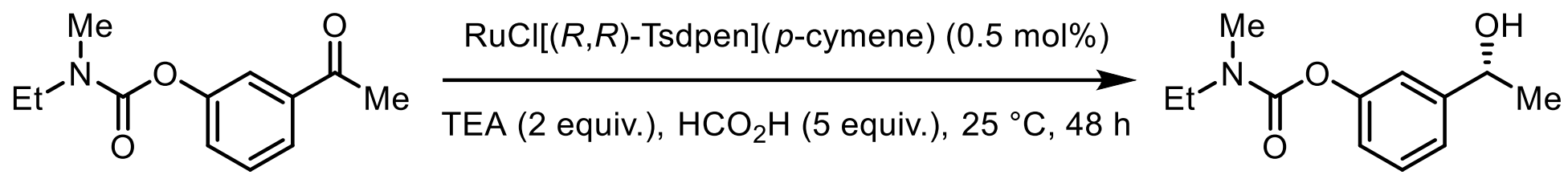

Formic acid (0.566 mL, 15.00 mmol, 5.00 equiv.) was added dropwise to a solution of 3-acetylphenyl ethyl(methyl)carbamate (664 mg, 3.00 mmol, 1.00 equiv.) in triethylamine (0.836 mL, 6.00 mmol, 2.00 equiv.) at 0 °C. The solution was allowed to warm to room temperature (25 °C) over 10 min. Subsequently, the Noyori-Ikariya catalyst ((RuCl[(*R*,*R*)-Tsdpen](*p*-cymene), 9.5 mg, 15 µmol, 0.5 mol%) was added in one portion and the resulting solution was stirred at room temperature for 48 h. After that time, the mixture was diluted with water and the aqueous layer was extracted with EtOAc (3 × 40 mL). The combined organic phases were washed with sat. aq. $NaHCO_3$ solution (40 mL), brine (40 mL), and dried over anhydrous Na_2SO_4. After filtration, volatiles were removed *in vacuo* and the crude mixture was purified by flash column chromatography (silica gel, 20-60% EtOAc in *n*-heptane) to obtain the desired product as a colourless oil (656 mg, 2.94 mmol, 98% yield).

^{1}H NMR (400 MHz, $CDCl_3$): δ 7.32 (t, *J* = 7.9 Hz, 1H), 7.18 (d, *J* = 7.7 Hz, 1H), 7.14 (s, 1H), 7.02 (d, *J* = 7.9 Hz, 1H), 4.88 (q, *J* = 6.4 Hz, 1H), 3.47 and 3.40 (rotamers, q, *J* = 7.0 Hz, 2H), 3.07 and 2.99 (rotamers, s, 3H), 2.02 (s, 1H), 1.48 (d, *J* = 6.5 Hz, 3H), 1.24 and 1.19 (rotamers, t, *J* = 7.0 Hz, 3H) ppm.

All analytical data were in accordance with those reported in literature.[57]

Enantiomeric ratio: 99:1 determined by chiral HPLC analysis: Lux-Cellulose-1, (*n*-heptane + 0,1% IPA)/IPA 8:2, 1.0 mL/min, 25 °C, detection at 210 nm, retention time (min): 5.9 (minor); 6.4 (major).

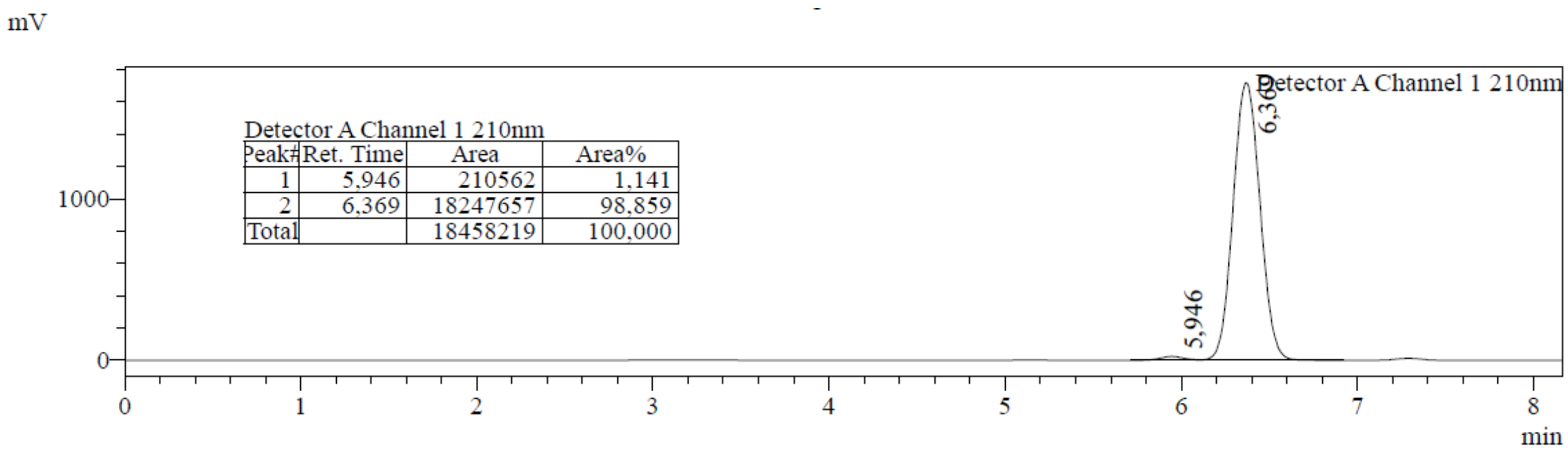

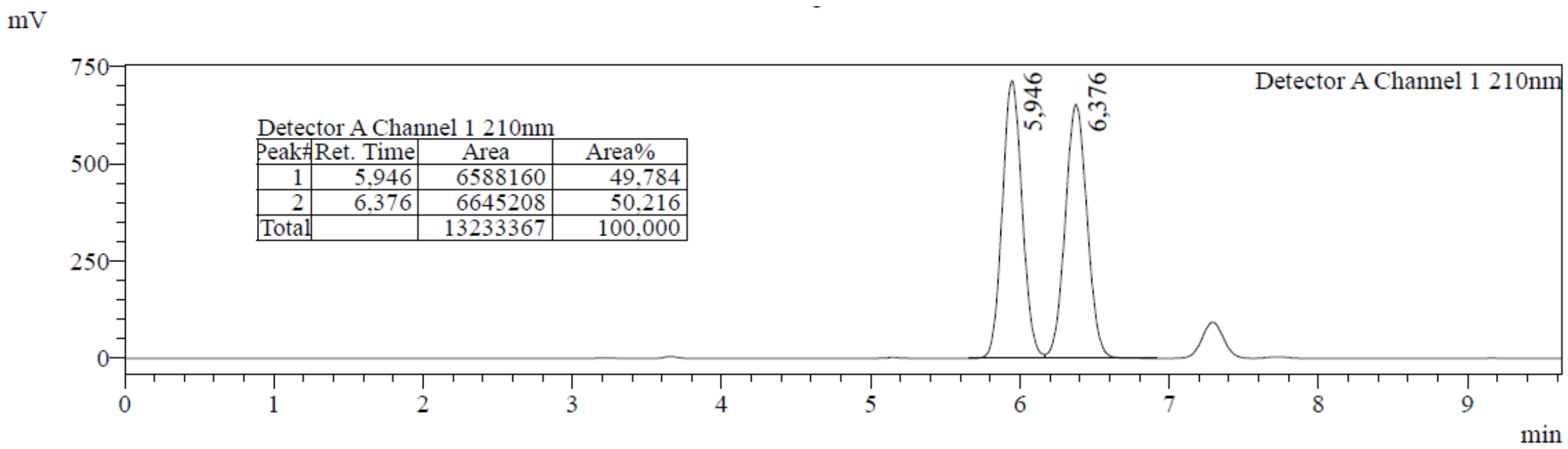

1-(3-((*tert*-Butyldimethylsilyl)oxy)propyl)-1-(4-fluorophenyl)-1,3-dihydroisobenzofuran-5-carbonitrile (S12)

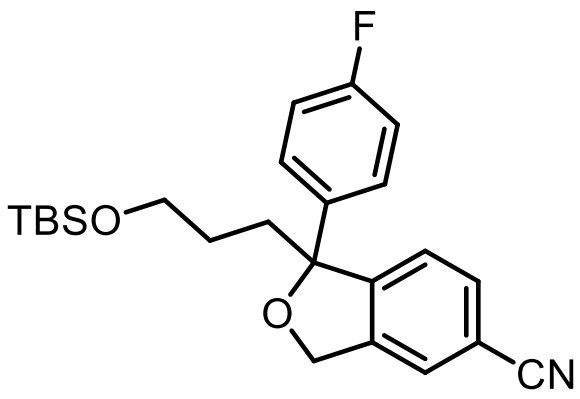

To a flame-dried, argon purged Schlenk flask containing tetrahydrofuran (6.0 mL) at -78 °C was added diisopropylamine (0.505 mL, 3.60 mmol, 1.20 equiv.), followed by slow addition of *n*-butyllithium (2.5 M in hexanes, 1.32 mL, 3.30 mmol, 1.10 equiv.). The resulting solution of LDA was stirred at -78 °C for 30 min. Subsequently, 1-(4-fluorophenyl)-1,3-dihydroisobenzofuran-5-carbonitrile (732 mg, 3.00 mmol, 1.00 equiv.) was added, obtaining a deep blue solution. After 30 min at -78 °C, the solution of (3-bromopropoxy)-*tert*-butyldimethylsilane (912 mg, 3.60 mmol, 1.20 equiv.) in dry THF (6.0 mL) was slowly added. The reaction mixture was allowed to warm to room temperature and stir for 1 h. After this time, distilled water (40 mL) was added to quench the reaction mixture and the reaction mixture was extracted with EtOAc (2 x 30 mL). The combined organic phases were washed with brine (30 mL) and dried over anhydrous Na_2SO_4. After filtration, volatiles were removed *in vacuo* and the crude mixture was purified by flash column chromatography (silica gel, 0-10% EtOAc in *n*-heptane) to obtain the desired product as a colourless oil (1.07 g, 2.59 mmol, 87% yield).

^{1}H NMR (400 MHz, $CDCl_3$): δ 7.59 (dd, *J* = 7.9, 1.3 Hz, 1H), 7.50 (s, 1H), 7.41 (ddt, *J* = 8.4, 5.3, 2.6 Hz, 2H), 7.36 (d, *J* = 7.9 Hz, 1H), 7.04 – 6.97 (m, 2H), 5.23 – 5.10 (m, 2H), 3.57 (t, *J* = 6.3 Hz, 2H), 2.29 – 2.12 (m, 2H), 1.58 – 1.46 (m, 1H), 1.42 – 1.31 (m, 1H), 0.87 (s, 9H), 0.00 (s, 6H) ppm.

^{13}C NMR (101 MHz, $CDCl_3$): δ 162.2 (d, $^1J_{C\text{-}F}$ = 246.3 Hz), 149.7, 140.5, 139.8 (d, $^4J_{C\text{-}F}$ = 3.2 Hz), 132.0, 127.0 (d, $^3J_{C\text{-}F}$ = 8.0 Hz, 2C), 125.3, 123.0, 118.8, 115.5 (d, $^2J_{C\text{-}F}$ = 21.4 Hz, 2C), 111.8, 91.3, 71.4, 62.9, 37.7, 27.6, 26.1 (3C), 18.4, -5.2 (2C) ppm.

^{19}F NMR (376 MHz, $CDCl_3$): δ -115.42 (tt, *J* = 8.5, 5.2 Hz, 1F) ppm.

IR (neat) ν_{max}: 2953, 2928, 2856, 2230, 1601, 1507, 1227, 1098, 829, 774 cm^{-1}.

HRMS (ESI$^+$): exact mass calculated for $[M+H]^+$ $(C_{24}H_{31}FNO_2{}^{28}Si)^+$ requires *m/z* 412.2103, found *m/z* 412.2100.

Rf: 0.21 *n*-heptane:EtOAc 20:1.

1-(4-Fluorophenyl)-1-(3-hydroxypropyl)-1,3-dihydroisobenzofuran-5-carbonitrile (S13)

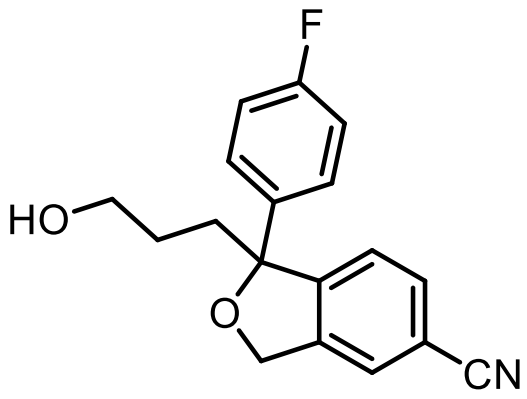

Tetrabutylammonium fluoride monohydrate (1.31 g, 5.00 mmol, 2.00 equiv.) was added to a solution of **S12** (1.03 g, 2.50 mmol, 1.00 equiv.) in THF (12.5 mL) at room temperature (25 °C). The reaction was stirred for 2 h at the same temperature, concentrated *in vacuo* and the crude mixture was purified by flash column chromatography (silica gel, 30-60% EtOAc in *n*-heptane) to obtain the desired product as a colourless oil (585 mg, 1.97 mmol, 79%).

^{1}H NMR (400 MHz, CDCl$_3$): δ 7.60 (dd, *J* = 7.9, 1.3 Hz, 1H), 7.51 (s, 1H), 7.46 – 7.36 (m, 3H), 7.06 – 6.96 (m, 2H), 5.25 – 5.11 (m, 2H), 3.61 (tt, *J* = 6.3, 3.2 Hz, 2H), 2.36 – 2.16 (m, 2H), 1.71 (br s, 1H), 1.63 – 1.40 (m, 2H) ppm.

^{13}C NMR (101 MHz, CDCl$_3$): δ 162.2 (d, $^{1}J_{C-F}$ = 246.5 Hz), 149.4, 140.3, 139.4 (d, $^{4}J_{C-F}$ = 3.1 Hz), 132.1, 126.9 (d, $^{3}J_{C-F}$ = 8.0 Hz, 2C), 125.4, 122.9, 118.7, 115.5 (d, $^{2}J_{C-F}$ = 21.4 Hz, 2C), 112.0, 91.3, 71.4, 62.7, 37.9, 27.4 ppm.

^{19}F NMR (376 MHz, CDCl$_3$): δ -115.20 (tt, *J* = 8.6, 5.3 Hz, 1F) ppm.

IR (neat) ν_{max}: 3433, 2950, 2865, 2230, 1600, 1506, 1224, 1031, 830, 730, 597 cm^{-1}.

HRMS (ESI$^+$): exact mass calculated for [M+Na]$^+$ ($C_{18}H_{16}FNO_2$)$^+$ requires *m/z* 320.1057, found *m/z* 320.1055.

Rf: 0.31 *n*-heptane:EtOAc 1:1.

3-(3-Chloro-10,11-dihydro-5*H*-dibenzo[*b,f*]azepin-5-yl)propan-1-ol (S15)

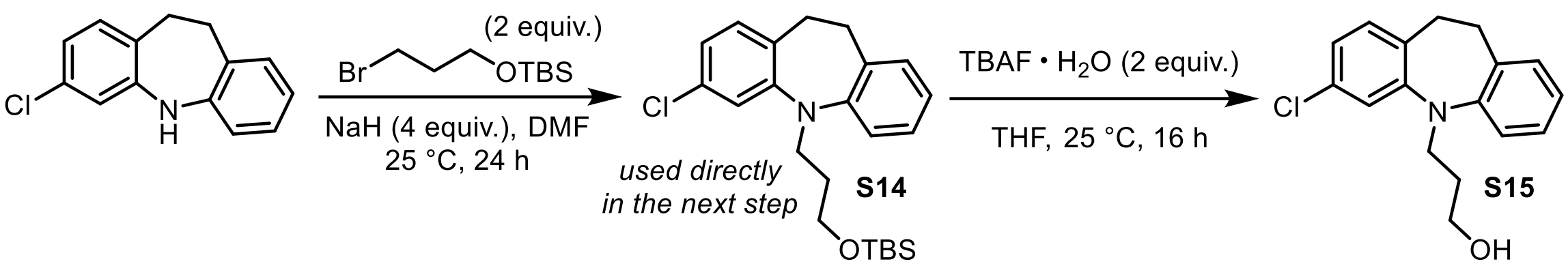

3-Chloro-10,11-dihydro-5*H*-dibenzo[*b,f*]azepine (1.15 g, 5.00 mmol, 1.00 equiv.) was loaded into a flame-dried Schlenk flask and dissolved in dry DMF (12.5 mL). Subsequently a solution of sodium hydride (60 % in oil, 0.80 g, 20.00 mmol, 4.00 equiv.) in DMF (12.5 mL) was added dropwise at room temperature (25 °C). The reaction mixture was stirred for 30 min, while a colour change to pale yellow was observed. Subsequently, (3-bromopropoxy)(*tert*-butyl)dimethylsilane (2.32 mL, 10.00 mmol, 2.00 equiv.) was slowly added. Then, the mixture was stirred for 24 h at room temperature. Afterwards, the crude reaction mixture was quenched with ice water (5.0 mL) and transferred into a separatory funnel, where the organic phase was washed with H_2O (50 mL) and brine (3 x 50 mL). The combined organic phases were dried over anhydrous Na_2SO_4, filtered and concentrated under reduced pressure to afford crude **S14** as a pale orange oil. The product was used directly in the next step without further purification.

Tetrabutylammonium fluoride monohydrate (2.67 g, 10.00 mmol, 2.00 equiv.) was added to a solution of crude **S14** in THF (50 mL) at room temperature (25 °C). The reaction was stirred for 16 h at the same temperature, concentrated *in vacuo* and the crude mixture was purified by flash column chromatography (silica gel, 10-50% EtOAc in *n*-heptane) to obtain the desired product as a colourless solid (910 mg, 3.16 mmol, 63% yield over two steps).

^{1}H NMR (400 MHz, $CDCl_3$): δ 7.19 – 7.05 (m, 4H), 7.02 – 6.95 (m, 2H), 6.91 – 6.86 (m, 1H), 3.83 (t, *J* = 6.6 Hz, 2H), 3.70 – 3.62 (m, 2H), 3.18 – 3.08 (m, 4H), 1.82 (p, *J* = 6.4 Hz, 2H), 1.51 (s, 1H) ppm.

^{13}C NMR (101 MHz, $CDCl_3$): δ 149.1, 147.9, 135.2, 131.72, 131.67, 131.4, 129.7, 126.7, 123.5, 122.3, 120.5, 119.9, 60.8, 47.3, 32.2, 31.6, 30.8 ppm.

IR (neat) ν_{max}: 3389, 2920, 2842, 1706, 1582, 1484, 1406, 1226, 755 cm^{-1}.

HRMS (ESI$^+$): exact mass calculated for $[M+H]^+$ $(C_{17}H_{19}{}^{35}ClNO)^+$ requires *m/z* 288.1150, found *m/z* 288.1147.

Rf: 0.36 *n*-heptane:EtOAc 2:1.

3.2 Nms-amide synthesis

General procedure 1 (GP1) for Nms-protection of amines:

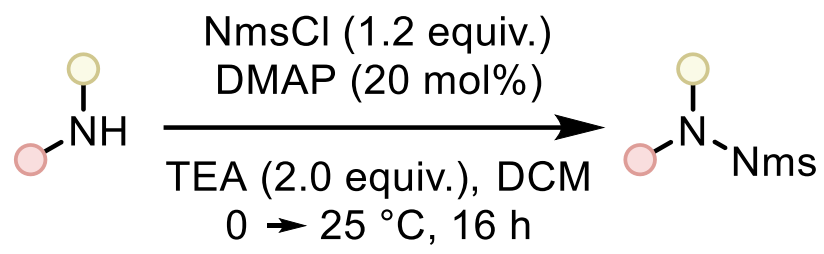

The corresponding amine (1.00 equiv.) was dissolved in DCM (0.1 M) at room temperature (25 °C). DMAP (20 mol%) and Et_3N (2.00 equiv. or 3.00 equiv., if the amine was used as a salt) were added at this temperature. Then, the mixture was cooled to 0 °C and 2,4,6-tris(trifluoromethyl)benzenesulfonyl chloride (1.20 equiv.) was added in one portion. The reaction was allowed to slowly warm to room temperature over 16 h (without removing the cooling bath after the addition of sulfonyl chloride). Then, the solvents were removed under reduced pressure and the crude material was subjected to flash column chromatography (silica gel) using the appropriate mixture of eluents.

***tert*-Butyl (4-((2,4,6-tris(trifluoromethyl)phenyl)sulfonamido)butyl)carbamate (S16)**

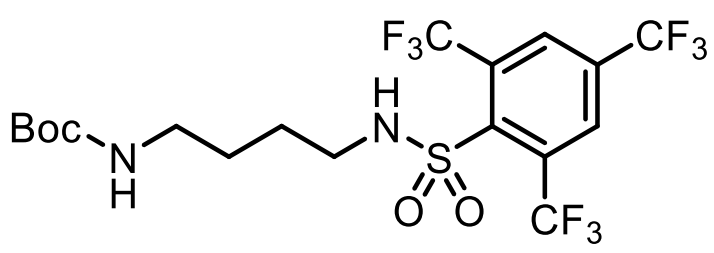

General procedure **GP1** was followed, using *N*-Boc-1,4-butanediamine (188 mg, 1.00 mmol, 1.00 equiv.). The resulting crude material was subjected to flash column chromatography (silica gel, 10%-50% EtOAc in *n*-heptane) to obtain the title compound as a colourless solid (374 mg, 0.70 mmol, 70% yield).

^{1}H NMR (700 MHz, $CDCl_3$): δ 8.29 (s, 2H), 5.39 (br s, 1H), 4.57 (br s, 1H), 3.26 – 3.21 (m, 2H), 3.16 – 3.11 (m, 2H), 1.64 – 1.59 (m, 2H), 1.54 (app p, J = 7.5 Hz, 2H), 1.43 (s, 9H) ppm.

^{13}C NMR (176 MHz, $CDCl_3$): δ 156.3, 145.8, 134.0 (q, $^2J_{C\text{-}F}$ = 33.1 Hz), 132.6 (q, $^2J_{C\text{-}F}$ = 33.8 Hz, 2C), 129.4 (m, 2C), 122.2 (q, $^1J_{C\text{-}F}$ = 274.0 Hz, 2C), 122.6 (q, $^1J_{C\text{-}F}$ = 273.6 Hz), 79.6, 43.7, 39.8, 28.5 (3C), 27.5, 27.3 ppm.

^{19}F NMR (376 MHz, $CDCl_3$): δ -54.72 (s, 6F), -63.56 (s, 3F) ppm.

IR (neat) ν_{max}: 1693, 1516, 1428, 1367, 127, 1193, 1084, 916, 864, 738, 707, 686, 669, 619, 569 cm^{-1}.

HRMS (ESI$^+$): exact mass calculated for $[M+Na]^+$ $(C_{18}H_{21}F_9N_2O_4{}^{32}S^{23}Na)^+$ requires *m/z* 555.0971, found *m/z* 555.0973.

R_f: 0.67 *n*-heptane:EtOAc 1:1.

(*R*)-*N*-(4-Oxo-4-(3-(trifluoromethyl)-5,6-dihydro-[1,2,4]triazolo[4,3-*a*]pyrazin-7(8*H*)-yl)-1-(2,4,5-trifluorophenyl)butan-2-yl)-2,4,6-tris(trifluoromethyl)benzenesulfonamide (S17)

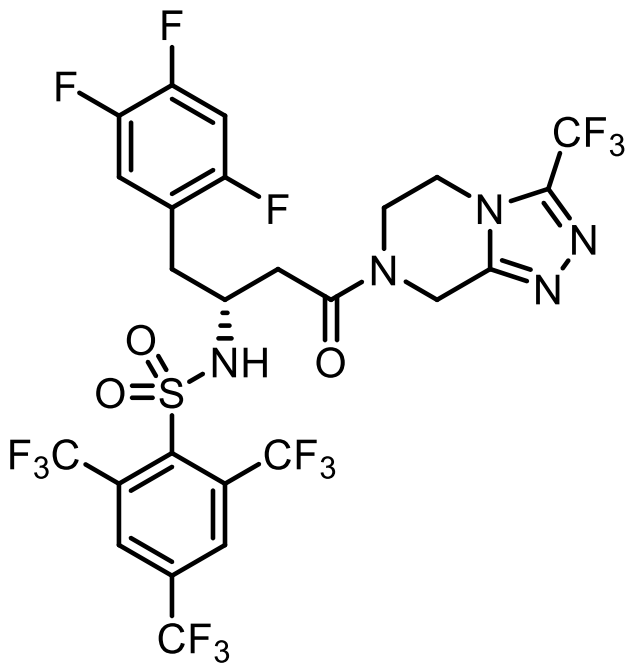

General procedure **GP1** was followed, using Sitagliptin (407 mg, 1.00 mmol, 1.00 equiv.). The resulting crude material was subjected to flash column chromatography (silica gel, 50%-100% EtOAc in *n*-heptane) to obtain the title compound as a colourless solid (669 mg, 0.892 mmol, 89% yield). The desired product was obtained as a mixture of two rotamers in a ratio of 1.2:1.

^{1}H NMR (400 MHz, Acetone-*d$_6$*): δ 8.47 (s, 2H), 7.30 (s, 1H), 7.18 (q, *J* = 8.8 Hz, 1H), 6.98 (p, *J* = 10.1 Hz, 1H), 5.12 – 4.96 (m, 1H), 4.87 (app q, *J* = 17.2 Hz, 1H), 4.48 – 4.32 (m, 2H), 4.29 – 3.96 (m, 3H), 3.18 – 2.92 (m, 4H) ppm.

Major isomer: **^{13}C NMR (101 MHz, Acetone-*d$_6$*):** δ 170.0, 157.0 (dd, *J* = 244.4, 9.6 Hz), 151.4, 149.2 (dd, *J* = 248.3, 14.4 Hz), 146.59, 146.56 (dd, *J* = 230.2, 3.9 Hz), 143.6 (q, *J* = 40.1, 39.7 Hz), 133.9 (q, *J* = 35.0 Hz), 132.1 (q, *J* = 33.1 Hz, 2C), 130.7 – 130.2 (m, 2C), 123.0 (q, *J* = 277.2 Hz, 2C), 122.7 (dt, *J* = 18.5, 5.6 Hz), 120.3 (dd, *J* = 19.2, 5.9 Hz), 120.2 (q, *J* = 273.2 Hz), 119.5 (q, *J* = 269.2 Hz), 105.8 (dd, *J* = 29.0, 21.0 Hz), 53.4, 44.3, 42.0, 39.2, 38.6, 34.0 ppm.

^{19}F NMR (376 MHz, Acetone-*d$_6$*): δ -54.99 (s, 6F), -63.68 (s, 3F), -64.11 (s, 3F), -119.01 – -119.43 (m, 1F), -137.65 – -137.95 (m, 1F), -144.83 – -145.19 (m, 1F) ppm.

Minor isomer: **^{13}C NMR (101 MHz, Acetone-*d$_6$*):** δ 170.0, 157.0 (dd, $^{1}J_{C-F}$ = 244.4, $^{3}J_{C-F}$ = 9.6 Hz), 151.1, 149.2 (dd, $^{1}J_{C-F}$ = 248.3, $^{3}J_{C-F}$ = 14.4 Hz), 146.59, 146.56 (dd, $^{1}J_{C-F}$ = 230.2, $^{4}J_{C-F}$ = 3.9 Hz), 143.6 (q, $^{2}J_{C-F}$ = 40.1, $^{2}J_{C-F}$ = 39.7 Hz), 133.9 (q, $^{2}J_{C-F}$ = 35.0 Hz), 132.1 (q, $^{2}J_{C-F}$ = 33.1 Hz, 2C), 130.7 – 130.2 (m, 2C), 123.0 (q, $^{1}J_{C-F}$ = 277.2 Hz, 2C), 122.7 (dt, $^{2}J_{C-F}$ = 18.5, $^{3}J_{C-F}$ = 5.6 Hz), 120.3 (dd, $^{2}J_{C-F}$ = 19.2, $^{3}J_{C-F}$ = 5.9 Hz), 120.2 (q, $^{1}J_{C-F}$ = 273.2 Hz), 119.5 (q, $^{1}J_{C-F}$ = 269.2 Hz), 105.8 (dd, $^{2}J_{C-F}$ = 29.0, $^{2}J_{C-F}$ = 21.0 Hz), 53.5, 43.9, 42.7, 39.0, 38.3, 34.0 ppm.

^{19}F NMR (376 MHz, Acetone-*d$_6$*): δ -54.96 (s, 6F), -63.66 (s, 3F), -64.14 (s, 3F), -119.01 – -119.43 (m, 1F), -137.65 – -137.95 (m, 1F), -144.83 – -145.19 (m, 1F) ppm.

IR (neat) ν_{max}: 3086, 1712, 1643, 1514, 1273, 1182, 1139, 1077, 708 cm^{-1}.

HRMS (ESI$^+$): exact mass calculated for $[M+Na]^+$ $(C_{25}H_{16}F_{15}N_5O_3{}^{32}S^{23}Na)^+$ requires *m/z* 774.0627, found *m/z* 774.0638.

Rf: 0.29 *n*-heptane:EtOAc 1:2.

$[\alpha]_D^{19}$ = 31.8 (*c* = 0.80, MeOH).

(*R*)-*N*-(1-(3-(2-Cyanobenzyl)-1-methyl-2,6-dioxo-1,2,3,6-tetrahydropyrimidin-4-yl)piperidin-3-yl)-2,4,6-tris(trifluoromethyl)benzenesulfonamide (S18)

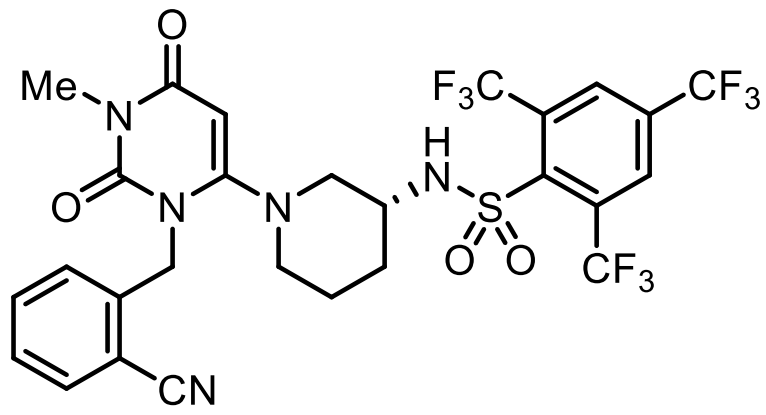

General procedure **GP1** was followed, using Alogliptin (411 mg, 1.21 mmol, 1.00 equiv.). The resulting crude material was subjected to flash column chromatography (silica gel, 50%-100% EtOAc in *n*-heptane) to obtain the title compound as a colourless solid (724 mg, 1.06 mmol, 88% yield).

^{1}H NMR (600 MHz, CDCl$_3$): δ 8.27 (s, 2H), 7.65 (d, *J* = 7.6 Hz, 1H), 7.55 (t, *J* = 7.6 Hz, 1H), 7.37 (t, *J* = 7.6 Hz, 1H), 7.16 (d, *J* = 7.8 Hz, 1H), 5.52 (d, *J* = 8.4 Hz, 1H), 5.37 (s, 1H), 5.29 – 5.14 (m, 2H), 3.80 (br s, 1H), 3.27 (s, 3H), 3.17 (br s, 1H), 3.02 – 2.44 (m, 3H), 1.93 (br s, 1H), 1.80 (br s, 1H), 1.74 – 1.38 (m, 2H) ppm.

^{13}C NMR (101 MHz, CDCl$_3$): δ 163.1, 159.3, 152.4, 145.8, 140.3, 134.3 (q, $^{2}J_{C-F}$ = 35.1 Hz), 133.4 (2C), 132.4 (q, $^{2}J_{C-F}$ = 33.7 Hz, 2C), 129.7 – 129.3 (m, 2C), 128.2, 127.1, 122.2 (q, $^{1}J_{C-F}$ = 275.5 Hz, 2C), 121.90 (q, $^{1}J_{C-F}$ = 273.4 Hz), 117.7, 110.8, 91.1, 56.8, 52.3, 50.7, 46.1, 30.7, 28.1, 23.0 ppm.

^{19}F NMR (376 MHz, CDCl$_3$): δ -54.59 (s, 6F), -63.59 (s, 3F) ppm.

IR (neat) ν$_{max}$: 2953, 2226, 1707, 1651, 1438, 1274, 1191, 1142, 707 cm^{-1}.

HRMS (ESI$^+$): exact mass calculated for [M+Na]$^+$ ($C_{27}H_{22}F_9N_5O_4{}^{32}S^{23}Na$)$^+$ requires *m/z* 706.1141, found *m/z* 706.1140.

Rf: 0.43 *n*-heptane:EtOAc 1:2.

[α]$_D^{19}$ = 22.9 (*c* = 0.87, CHCl$_3$).

***N*-(4-((6-Methoxyquinolin-8-yl)amino)pentyl)-2,4,6-tris(trifluoromethyl)benzenesulfonamide (S19)**

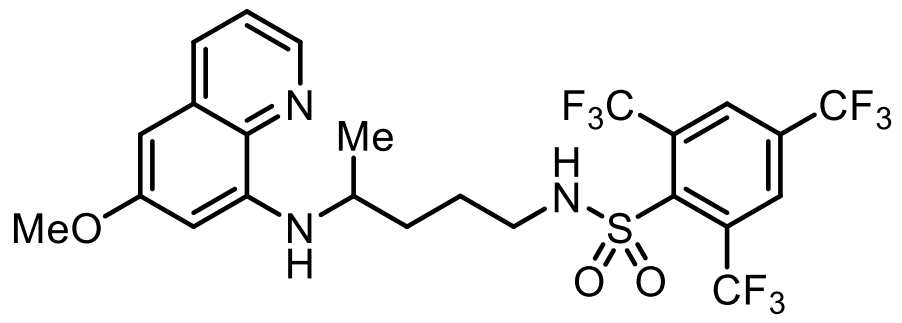

General procedure **GP1** was followed, using Primaquine (2.075 g, 8.00 mmol, 1.00 equiv.). The resulting crude material was subjected to flash column chromatography (silica gel, 10%-50% EtOAc in *n*-heptane) to obtain the title compound as a pale-yellow solid (4.296 g, 7.11 mmol, 89% yield).

^{1}H NMR (600 MHz, CDCl$_3$): δ 8.52 (d, *J* = 2.5 Hz, 1H), 8.27 (s, 2H), 7.92 (d, *J* = 8.1 Hz, 1H), 7.31 (dd, *J* = 8.0, 4.1 Hz, 1H), 6.34 (s, 1H), 6.26 (s, 1H), 5.96 (d, *J* = 8.1 Hz, 1H), 5.05 (s, 1H), 3.89 (s, 3H), 3.63 (s, 1H), 3.27 – 3.22 (m, 2H), 1.80 – 1.66 (m, 4H), 1.30 (d, *J* = 6.2 Hz, 3H) ppm.

^{13}C NMR (101 MHz, CDCl$_3$): δ 159.5, 145.7, 144.9, 144.5, 135.5, 135.0, 134.1 (q, $^2J_{C-F}$ = 35.0 Hz), 132.6 (q, $^2J_{C-F}$ = 33.5 Hz, 2C), 130.0, 129.6 – 129.3 (m, 2C), 122.3 (q, $^1J_{C-F}$ = 275.5 Hz, 2C), 122.04, 122.01 (q, $^1J_{C-F}$ = 273.9 Hz), 97.1, 92.1, 55.4, 47.9, 44.1, 33.7, 27.2, 20.8 ppm.

^{19}F NMR (376 MHz, CDCl$_3$): δ -54.74 (s, 6F), -63.58 (s, 3F) ppm.

IR (neat) ν_{max}: 3401, 2963, 1712, 1520, 1272, 1193, 1148, 1082, 706 cm^{-1}.

HRMS (ESI$^+$): exact mass calculated for [M+H]$^+$ ($C_{24}H_{23}F_9N_3O_3{}^{32}S$)$^+$ requires *m/z* 604.1311, found *m/z* 604.1315.

Rf: 0.24 *n*-heptane:EtOAc 5:1.

***N*-(4-Methoxyphenyl)-*N*-methyl-2,4,6-tris(trifluoromethyl)benzenesulfonamide (S20)**

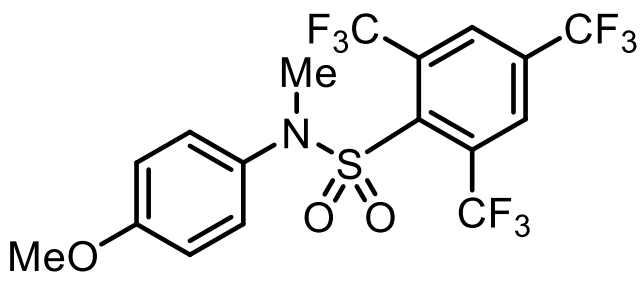

Under an argon atmosphere, a flame-dried Schlenk flask was charged with anhydrous solvents (toluene/THF 4:1, 2.0 mL) and cooled to 0 °C, before adding triphenylphosphine (162 mg, 0.62 mmol, 1.50 equiv.) and diisopropyl azodicarboxylate (DIAD, 122 µL, 125 mg, 0.62 mmol, 1.50 equiv.). After 15 min at 0 °C, the reaction mixture solidified and a mixture of methanol (25.1 µL, 19.5 mg, 0.62 mmol, 1.50 equiv.) and *N*-(4-methoxyphenyl)-2,4,6-tris(trifluoromethyl)benzenesulfonamide[33] (193 mg, 0.41 mmol, 1.00 equiv.) in toluene:THF (4:1, 1.2 mL) was added. The resulting mixture became a clear solution and was stirred at room temperature (25 °C) for 16 h. After that time, the solvent was removed *in vacuo* and the residue was purified by flash column chromatography (silica gel, 50% EtOAc in *n*-heptane followed by 100% toluene), to afford the title compound as a yellow solid (124 mg, 0.25 mmol, 62% yield).

^{1}H NMR (500 MHz, CDCl$_3$): δ 8.31 (s, 2H), 7.09 – 7.06 (m, 2H), 6.84 – 6.80 (m, 2H), 3.79 (s, 3H), 3.32 (s, 3H) ppm.

^{13}C NMR (126 MHz, CDCl$_3$): δ 159.8, 144.5, 134.5 (q, $^2J_{C-F}$ = 35.8 Hz), 133.8 (q, $^2J_{C-F}$ = 34.0 Hz, 2C), 132.5, 129.9 (2C), 129.8 – 129.3 (m, 2C), 122.0 (q, $^1J_{C-F}$ = 273.6 Hz), 121.9 (q, $^1J_{C-F}$ = 276.7 Hz, 2C), 115.0 (2C), 55.6, 41.8 ppm.

^{19}F NMR (376 MHz, CDCl$_3$): δ -54.98 (s, 6F), -63.62 (s, 3F) ppm.

IR (neat) ν_{max}: 1510, 1348, 1255, 1188, 1144, 1062, 1028, 915, 879, 797, 709, 684 cm^{-1}.

HRMS (ESI$^+$): exact mass calculated for [M+Na]$^+$ ($C_{17}H_{12}F_9NO_3{}^{32}S^{23}Na$) $^+$ requires *m/z* 504.0286, found *m/z* 504.0287.

R$_f$: 0.76 *n*-heptane:EtOAc 1:1.

tert-Butyl 4-((2,4,6-tris(trifluoromethyl)phenyl)sulfonyl)piperazine-1-carboxylate (7)

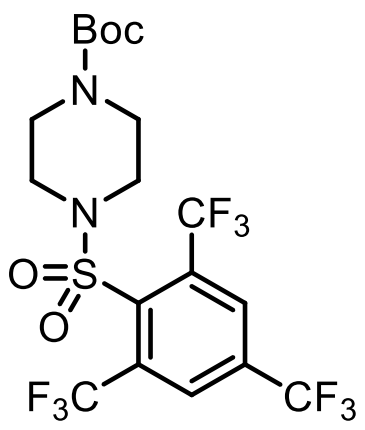

General procedure **GP1** was followed, using *tert*-butyl piperazine-1-carboxylate (186 mg, 1.00 mmol, 1.00 equiv.). The resulting crude material was subjected to flash column chromatography (silica gel, 0%-40% EtOAc in *n*-heptane) to obtain the title compound as a colourless solid (370 mg, 0.70 mmol, 70% yield).

^{1}H NMR (600 MHz, CDCl$_3$): δ 8.28 (s, 2H), 3.54 (t, *J* = 4.8 Hz, 4H), 3.33 (t, *J* = 4.8 Hz, 4H), 1.46 (s, 9H) ppm.

^{13}C NMR (151 MHz, CDCl$_3$): δ 154.5, 144.9, 134.5 (q, $^{2}J_{C-F}$ = 35.7 Hz), 133.2 (q, $^{2}J_{C-F}$ = 33.8 Hz, 2C), 129.7 – 129.2 (m, 2C), 122.2 (q, $^{1}J_{C-F}$ = 275.7 Hz, 2C), 122.0 (q, $^{1}J_{C-F}$ = 273.5 Hz), 80.8, 46.8 (2C), 44.5 – 43.2 (br m, 2C), 28.5 (3C).

^{19}F NMR (376 MHz, CDCl$_3$): δ -55.39 (s, 6F), -63.58 (s, 3F) ppm.

IR (neat) ν_{max}: 1680, 1358, 1270, 1180, 1148, 1133, 1113, 1088, 983, 914, 724 cm^{-1}.

HRMS (ESI^{+}): exact mass calculated for $[M+Na]^{+}$ $(C_{18}H_{19}F_9N_2O_4{}^{32}S^{23}Na)^{+}$ requires *m/z* 553.0814, found *m/z* 553.0805.

R_f: 0.73 *n*-heptane:EtOAc 1:1.

4-Phenyl-1-((2,4,6-tris(trifluoromethyl)phenyl)sulfonyl)piperidin-4-ol (S21)

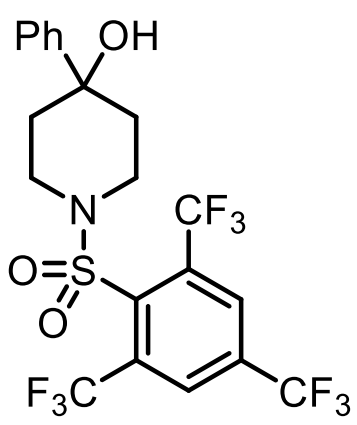

General procedure **GP1** was followed, using 4-phenylpiperidin-4-ol (88.6 mg, 0.50 mmol, 1.00 equiv.). The resulting crude material was subjected to flash column chromatography (silica gel, 0%-100% EtOAc in *n*-heptane) to obtain the title compound as a colourless solid (215 mg, 0.41 mmol, 83% yield).

^{1}H NMR (400 MHz, CDCl$_3$): δ 8.28 (s, 2H), 7.49 – 7.46 (m, 2H), 7.41 – 7.36 (m, 2H), 7.34 – 7.25 (m, 1H), 3.70 (dd, *J* = 13.6, 4.6 Hz, 2H), 3.58 – 3.44 (m, 2H), 2.29 (td, *J* = 13.6, 4.6 Hz, 2H), 1.80 (m, 2H), 1.51 (s, 1H) ppm. *(The signal for OH group could not be detected)*.

^{13}C NMR (126 MHz, CDCl$_3$): δ 147.3, 145.5, 134.0 (q, $^{2}J_{C-F}$ = 33.6 Hz), 133.2 (q, $^{2}J_{C-F}$ = 33.8 Hz, 2C), 129.6 – 129.2 (m, 2C), 128.8 (2C), 127.8, 124.4 (2C), 121.6 (q, $^{1}J_{C-F}$ = 277.1 Hz, 2C), 121.5 (q, $^{1}J_{C-F}$ = 273.4 Hz), 71.5, 43.3 (2C), 38.4 (2C) ppm.

^{19}F NMR (659 MHz, CDCl$_3$): δ -55.46 (s, 6F), -63.55 (s, 3F) ppm.

IR (neat) ν_{max}: 3547, 1352, 1283, 1136, 1115, 1088, 913, 670, 648 cm^{-1}.

HRMS (ESI⁺): exact mass calculated for $[M+Na]^+$ $(C_{20}H_{16}F_9NO_3{}^{32}S^{23}Na)^+$ requires *m/z* 544.0599, found *m/z* 544.0598.

R_f: 0.67 *n*-heptane:EtOAc 1:1.

5-((4-((2,4,6-Tris(trifluoromethyl)phenyl)sulfonyl)-1,4-diazepan-1-yl)sulfonyl)isoquinoline (S22)

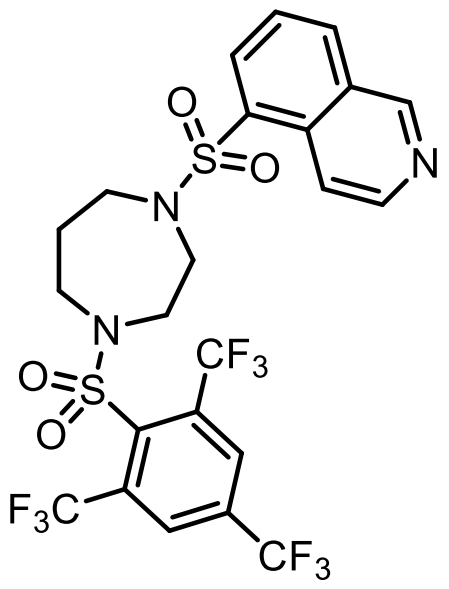

General procedure **GP1** was followed, using Fasudil hydrochloride (328 mg, 1.00 mmol, 1.00 equiv.). The resulting crude material was subjected to flash column chromatography (silica gel, 20%-100% EtOAc in *n*-heptane) to obtain the title compound as a colourless solid (315 mg, 0.496 mmol, 50% yield).

^{1}H NMR (400 MHz, $CDCl_3$): δ 9.35 (s, 1H), 8.68 (d, *J* = 6.0 Hz, 1H), 8.36 (d, *J* = 6.0 Hz, 1H), 8.27 (s, 3H), 8.21 (d, *J* = 8.1 Hz, 1H), 7.68 (t, *J* = 7.8 Hz, 1H), 3.62 – 3.46 (m, 8H), 2.17 – 2.07 (m, 2H) ppm.

^{13}C NMR (101 MHz, $CDCl_3$): δ 153.5, 145.5, 144.5, 134.4 (q, $^2J_{C-F}$ = 35.3 Hz), 134.1, 133.9, 133.0 (q, $^2J_{C-F}$ = 34.1 Hz, 2C), 132.7, 131.6, 129.63 – 129.40 (m, 2C), 129.36, 126.0, 122.1 (q, $^1J_{C-F}$ = 275.6 Hz, 2C), 121.9 (q, $^1J_{C-F}$ = 273.6 Hz), 117.4, 53.5, 51.6, 48.7, 47.6, 29.7 ppm.

^{19}F NMR (376 MHz, $CDCl_3$): δ -55.75 (s, 6F), -63.59 (s, 3F) ppm.

IR (neat) ν_{max}: 2941, 1618, 1362, 1279, 1195, 1152, 1136, 911, 711 cm^{-1}.

HRMS (ESI⁺): exact mass calculated for $[M+Na]^+$ $(C_{23}H_{18}F_9N_3O_4{}^{32}S_2{}^{23}Na)^+$ requires *m/z* 658.0487, found *m/z* 658.0489.

Rf: 0.29 *n*-heptane:EtOAc 1:1.

***N*-Methyl-*N*-((5-phenyl-1-(pyridin-3-ylsulfonyl)-1*H*-pyrrol-3-yl)methyl)-2,4,6-tris(trifluoromethyl) benzenesulfonamide (S23)**

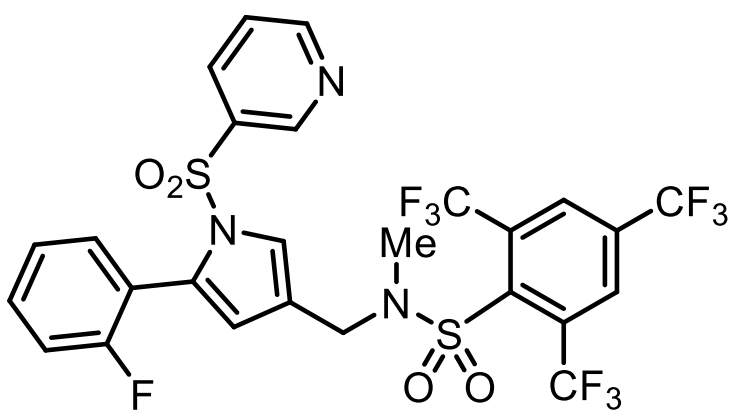

General procedure **GP1** was followed, using Vonoprazan (173 mg, 0.50 mmol, 1.00 equiv.). The resulting crude material was subjected to flash column chromatography (silica gel, 10%-70% EtOAc in *n*-heptane) to obtain the title product as a colourless solid (329 mg, 0.36 mmol, 71% yield).

^{1}H NMR (700 MHz, CDCl$_3$): δ 8.77 (dd, *J* = 4.7, 1.3 Hz, 1H), 8.60 (d, *J* = 2.0 Hz, 1H), 8.29 (s, 2H), 7.69 (dt, *J* = 8.1, 1.8 Hz, 1H), 7.49 (s, 1H), 7.44 – 7.38 (m, 1H), 7.34 (dd, *J* = 8.0, 4.9 Hz, 1H), 7.15 (p, *J* = 7.3 Hz, 2H), 7.03 (t, *J* = 8.8 Hz, 1H), 6.40 (d, *J* = 1.6 Hz, 1H), 4.35 (s, 2H), 2.65 (s, 3H) ppm.

^{13}C NMR (151 MHz, CDCl$_3$): δ 160.8 (d, $^{1}J_{C\text{-}F}$ = 249.6 Hz), 154.5, 148.0, 144.9, 135.1, 134.9, 134.6 (q, $^{2}J_{C\text{-}F}$ = 35.6 Hz), 133.4, 133.1 (q, $^{2}J_{C\text{-}F}$ = 35.6 Hz, 2C), 131.6 (d, $^{3}J_{C\text{-}F}$ = 7.6 Hz), 129.8, 129.61 – 129.36 (m, 2C), 123.7, 123.6 (d, $^{3}J_{C\text{-}F}$ = 4.4 Hz), 123.2, 122.4, 122.3 (q, $^{1}J_{C\text{-}F}$ = 275.7 Hz, 2C), 122.1 (q, $^{1}J_{C\text{-}F}$ = 273.8 Hz), 118.6 (d, $^{2}J_{C\text{-}F}$ = 15.3 Hz), 118.0, 115.5 (d, $^{2}J_{C\text{-}F}$ = 21.8 Hz), 47.3, 34.6 ppm.

^{19}F NMR (376 MHz, CDCl$_3$): δ -55.71 (s, 6F), -63.55 (s, 3F), -111.04 – -111.12 (m, 1F) ppm.

IR (neat) ν_{max}: 1738, 1272, 1193, 1106, 1073, 912, 749, 710, 684 cm^{-1}.

HRMS (ESI$^+$): exact mass calculated for [M+Na]$^+$ ($C_{26}H_{17}F_{10}N_3O_4{}^{32}S_2{}^{23}Na$)$^+$ requires *m/z* 712.0393, found *m/z* 712.0388.

R$_f$: 0.48 *n*-heptane:EtOAc 1:1.

***N*-(3,4-Dimethoxyphenethyl)-*N*-methyl-2,4-dinitrobenzenesulfonamide (14)**

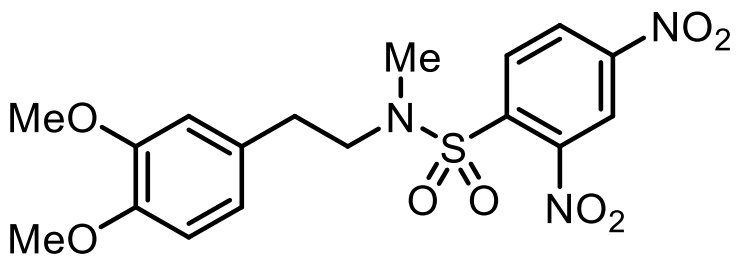

General procedure **GP1** was followed, using 2-(3,4-dimethoxyphenyl)-*N*-methylethylamine (617 mg, 3.00 mmol, 1.00 equiv.) and 2,4-dinitrobenzenesulfonyl chloride (960 mg, 3.60 mmol, 1.20 equiv.). The resulting crude material was subjected to flash column chromatography (silica gel, 10%-50% EtOAc in *n*-heptane) to obtain the title compound as a brown solid (823 mg, 1.93 mmol, 65% yield).

^{1}H NMR (400 MHz, CDCl$_3$): δ 8.38 (d, *J* = 2.1 Hz, 1H), 8.33 (dd, *J* = 8.6, 2.2 Hz, 1H), 7.99 (d, *J* = 8.6 Hz, 1H), 6.68 (d, *J* = 0.9 Hz, 2H), 6.63 (s, 1H), 3.82 (s, 3H), 3.80 (s, 3H), 3.54 (t, *J* = 7.1 Hz, 2H), 3.01 (s, 3H), 2.83 (t, *J* = 7.1 Hz, 2H) ppm.

^{13}C NMR (101 MHz, CDCl$_3$): δ 149.4, 148.9, 148.1, 147.8, 138.5, 132.3, 130.3, 126.0, 121.0, 119.6, 112.2, 111.3, 56.0, 55.9, 52.5, 35.2, 34.1 ppm.

IR (neat) ν_{max}: 2935, 2837, 1552, 1537, 1516, 1351, 1263, 1159, 752 cm^{-1}.

HRMS (ESI$^+$): exact mass calculated for [M+Na]$^+$ ($C_{17}H_{19}N_3O_8{}^{32}S^{23}Na$)$^+$ requires *m/z* 448.0785, found *m/z* 448.0780.

Rf: 0.20 *n*-heptane:EtOAc 2:1.

3.3 Amine synthesis through deprotective alkylation

General procedure 2 (GP2) for deprotective alkylation of tertiary Nms-amides:

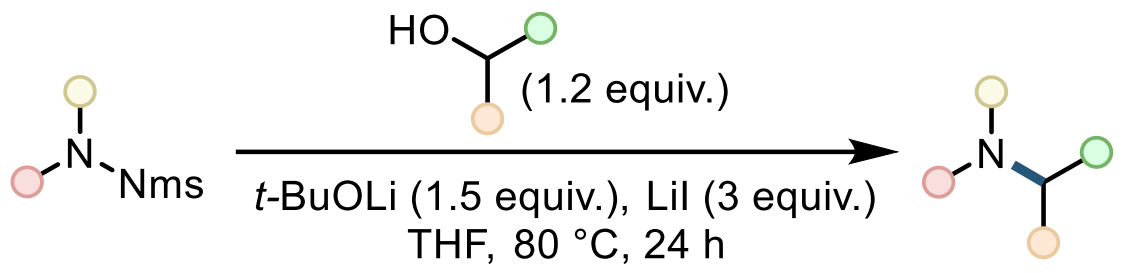

In a glovebox, an oven-dried vial was charged with solid lithium *tert*-butoxide (1.5 equiv.), capped and removed from glovebox. Removing the cap only briefly to maintain the inert atmosphere, the corresponding Nms-amide (1.00 equiv.) and lithium iodide (0.20 or 3.00 equiv.) were added, followed by the addition of the alcohol (1.20 equiv.) dissolved in THF (0.1 M). The vial was tightly sealed and placed into a metal heating-block. The reaction mixture was stirred for 24 h at 80 °C. After that time, the reaction mixture was cooled to room temperature (25 °C), quenched with water (2.0 mL) and transferred to separatory funnel. The crude material was diluted with 1M NaOH aqueous solution (10 mL) and extracted with DCM (3 x 10 mL). The combined organic phases were dried over anhydrous Na_2SO_4 and concentrated *in vacuo*. The crude material was subjected to flash column chromatography (silica gel) using the appropriate mixture of eluents.

General procedure 3 (GP3) for deprotective alkylation of secondary Nms-amides:

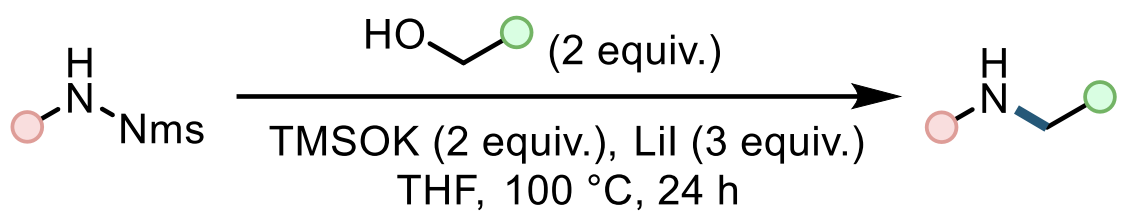

An oven-dried vial was charged with the corresponding Nms-amide (1.0 equiv.) and lithium iodide (3.0 equiv.), followed by addition of the alcohol (2.0 equiv.) dissolved in THF (0.1 M). Subsequently, a solution of TMSOK (1 M in THF, 2.0 equiv.) was added. The vial was tightly sealed and placed into a metal heating-block. The reaction mixture was stirred for 24 h at 100 °C. After that time, the reaction mixture was cooled to room temperature (25 °C), quenched with water (2 mL) and transferred to separatory funnel. The crude material was diluted with 1M NaOH aqueous solution (10 mL) and extracted with DCM (3 x 10 mL) The combined organic phases were dried over anhydrous Na_2SO_4 and concentrated *in vacuo*. The crude material was subjected to flash column chromatography (silica gel) using the appropriate mixture of eluents.

2-(3,4-Dimethoxyphenyl)-*N*,*N*-dimethylethan-1-amine (2a)

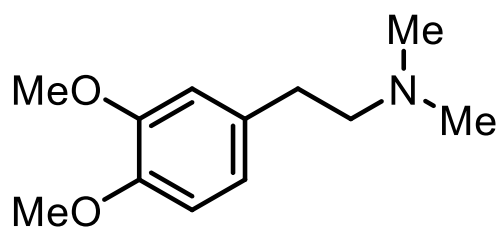

General procedure **GP2** was followed, using sulfonamide **1a** (80.9 mg, 0.15 mmol, 1.00 equiv.), methanol (7.3 µL, 0.18 mmol, 1.20 equiv.) and lithium iodide in catalytic amount (4.0 mg, 0.03 mmol, 20 mol%). The resulting crude material was subjected to flash column chromatography (silica gel, 10%-20% EtOH in EtOAc with 1% NH_4OH (25% in H_2O)) to obtain the title compound as a colourless oil (15.9 mg, 0.076 mmol, 51% yield).

^{1}H NMR (400 MHz, $CDCl_3$): δ 6.81 – 6.77 (m, 1H), 6.76 – 6.71 (m, 2H), 3.87 (s, 3H), 3.85 (s, 3H), 2.78 – 2.72 (m, 2H), 2.58 – 2.51 (m, 2H), 2.32 (s, 6H) ppm.

^{13}C NMR (101 MHz, $CDCl_3$): δ 149.0, 147.5, 132.8, 120.6, 112.1, 111.4, 61.8, 56.1, 56.0, 45.5 (2C), 34.0 ppm.

IR (neat) ν_{max}: 2938, 2831, 1514, 1462, 1260, 1234, 1143, 1027, 764 cm^{-1}.

HRMS (ESI$^+$): exact mass calculated for $[M+H]^+$ $(C_{12}H_{20}NO_2)^+$ requires *m/z* 210.1489, found *m/z* 210.1487.

Rf: 0.19 EtOAc:MeOH 10:1.

2-(3,4-Dimethoxyphenyl)-*N*-ethyl-*N*-methylethan-1-amine (2b)

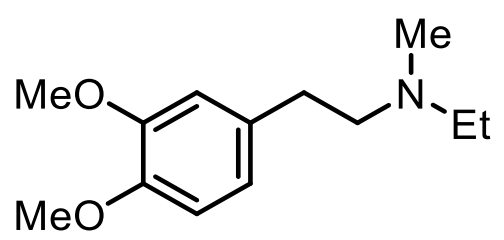

General procedure **GP2** was followed, using sulfonamide **1a** (80.9 mg, 0.15 mmol, 1.00 equiv.) and ethanol (10.5 µL, 0.18 mmol, 1.20 equiv.). The resulting crude material was subjected to flash column chromatography (silica gel, 10%-20% EtOH in EtOAc with 1% NH_4OH (25% in H_2O)) to obtain the title compound as a colourless oil (20.3 mg, 0.091 mmol, 61% yield).

^{1}H NMR (400 MHz, $CDCl_3$): δ 6.81 – 6.76 (m, 1H), 6.76 – 6.70 (m, 2H), 3.87 (s, 3H), 3.85 (s, 3H), 2.77 – 2.69 (m, 2H), 2.63 – 2.55 (m, 2H), 2.49 (q, *J* = 7.2 Hz, 2H), 2.30 (s, 3H), 1.08 (t, *J* = 7.2 Hz, 3H) ppm.

^{13}C NMR (101 MHz, $CDCl_3$): δ 149.0, 147.4, 133.3, 120.6, 112.1, 111.4, 59.6, 56.1, 56.0, 51.5, 41.7, 33.7, 12.4 ppm.

IR (neat) ν_{max}: 2934, 2834, 1514, 1463, 1260, 1234, 1140, 802 cm^{-1}.

HRMS (ESI$^+$): exact mass calculated for $[M+H]^+$ $(C_{13}H_{22}NO_2)^+$ requires *m/z* 224.1645, found *m/z* 224.1643.

Rf: 0.35 EtOAc:EtOH 6:1 + 1% NH_4OH (25% in H_2O).

N-Benzyl-2-(3,4-dimethoxyphenyl)-N-methylethan-1-amine (2c)

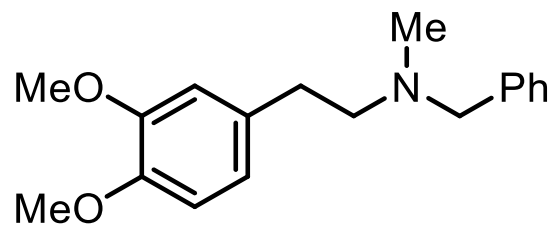

General procedure **GP2** was followed, using sulfonamide **1a** (80.9 mg, 0.15 mmol, 1.00 equiv.) and benzyl alcohol (19.5 mg, 0.18 mmol, 1.20 equiv.). The resulting crude material was subjected to flash column chromatography (silica gel, 10%-100% EtOAc in *n*-heptane) to obtain the title compound as a pale-yellow oil (36.6 mg, 0.128 mmol, 86% yield).

^{1}H NMR (400 MHz, CDCl$_3$): δ 7.33 – 7.27 (m, 4H), 7.26 – 7.22 (m, 1H), 6.79 (d, *J* = 8.0 Hz, 1H), 6.74 – 6.70 (m, 2H), 3.85 (d, *J* = 2.0 Hz, 6H), 3.56 (s, 2H), 2.78 (dd, *J* = 9.4, 6.1 Hz, 2H), 2.64 (dd, *J* = 9.2, 5.9 Hz, 2H), 2.28 (s, 3H) ppm.

^{13}C NMR (101 MHz, CDCl$_3$): δ 148.9, 147.4, 139.2, 133.3, 129.2 (2C), 128.3 (2C), 127.1, 120.7, 112.2, 111.3, 62.4, 59.5, 56.1, 55.9, 42.4, 33.7 ppm.

IR (neat) ν_{max}: 2934, 2835, 2787, 1514, 1262, 1234, 1140, 1027, 734 cm^{-1}.

HRMS (ESI$^+$): exact mass calculated for $[M+H]^+$ $(C_{18}H_{24}NO_2)^+$ requires *m/z* 286.1802, found *m/z* 286.1801.

Rf: 0.36 *n*-heptane:EtOAc 1:1.

N-(4-Bromobenzyl)-2-(3,4-dimethoxyphenyl)-N-methylethan-1-amine (2d)

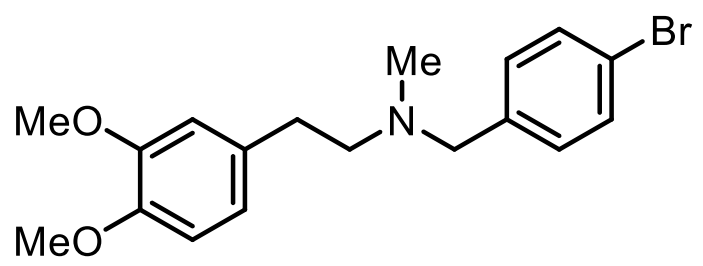

General procedure **GP2** was followed, using sulfonamide **1a** (80.9 mg, 0.15 mmol, 1.00 equiv.) and 4-bromobenzyl alcohol (33.7 mg, 0.18 mmol, 1.20 equiv.). The resulting crude material was subjected to flash column chromatography (silica gel, 20%-100% EtOAc in *n*-heptane) to obtain the title compound as a colourless oil (44.9 mg, 0.123 mmol, 82% yield).

^{1}H NMR (400 MHz, CDCl$_3$): δ 7.42 (d, *J* = 8.2 Hz, 2H), 7.16 (d, *J* = 8.2 Hz, 2H), 6.79 (d, *J* = 8.1 Hz, 1H), 6.74 – 6.65 (m, 2H), 3.86 (s, 3H), 3.85 (s, 3H), 3.52 (s, 2H), 2.80 – 2.73 (m, 2H), 2.66 – 2.59 (m, 2H), 2.28 (s, 3H) ppm.

^{13}C NMR (101 MHz, CDCl$_3$): δ 148.9, 147.5, 137.9, 132.9, 131.4 (2C), 130.8 (2C), 121.0, 120.7, 112.1, 111.3, 61.6, 59.3, 56.0, 55.9, 42.2, 33.5 ppm.

IR (neat) ν_{max}: 2933, 2834, 1514, 1462, 1261, 1234, 1141, 1028, 1009, 798 cm^{-1}.

HRMS (ESI$^+$): exact mass calculated for $[M+H]^+$ $(C_{18}H_{23}{}^{79}BrNO_2)^+$ requires *m/z* 364.0907, found *m/z* 364.0906.

Rf: 0.44 *n*-heptane:EtOAc 1:2.

2-(3,4-Dimethoxyphenyl)-*N*-methyl-*N*-(4-(trimethylsilyl)benzyl)ethan-1-amine (2e)

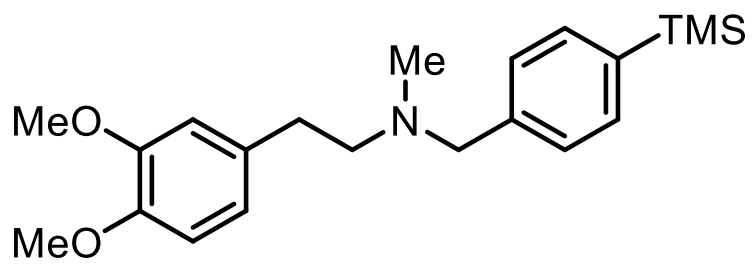

General procedure **GP2** was followed, using sulfonamide **1a** (80.9 mg, 0.15 mmol, 1.00 equiv.) and (4-(trimethylsilyl)phenyl)methanol[58] (32.5 mg, 0.18 mmol, 1.20 equiv.). The resulting crude material was subjected to flash column chromatography (silica gel, 0%-70% EtOAc in *n*-heptane) to obtain the title compound as a colourless oil (37.5 mg, 0.105 mmol, 70% yield).

^{1}H NMR (400 MHz, $CDCl_3$): δ 7.47 (d, *J* = 7.9 Hz, 2H), 7.29 (d, *J* = 7.8 Hz, 2H), 6.79 (d, *J* = 8.0 Hz, 1H), 6.76 – 6.63 (m, 2H), 3.86 (s, 3H), 3.85 (s, 3H), 3.56 (s, 2H), 2.78 (dd, *J* = 9.3, 6.1 Hz, 2H), 2.65 (dd, *J* = 9.2, 6.0 Hz, 2H), 2.29 (s, 3H), 0.27 (s, 9H) ppm.

^{13}C NMR (101 MHz, $CDCl_3$): δ 148.9, 147.4, 139.8, 139.0, 133.4 (2C), 133.3, 128.5 (2C), 120.7, 112.2, 111.3, 62.3, 59.4, 56.1, 55.9, 42.4, 33.6, -0.9 (3C) ppm.

IR (neat) ν_{max}: 2951, 2788, 1514, 1463, 1260, 1236, 1030, 833, 800, 754 cm^{-1}.

HRMS (ESI$^+$): exact mass calculated for $[M+H]^+$ $(C_{21}H_{32}NO_2{}^{28}Si)^+$ requires *m/z* 358.2197, found *m/z* 358.2194.

Rf: 0.27 *n*-heptane:EtOAc 1:1.

4-(((3,4-Dimethoxyphenethyl)(methyl)amino)methyl)benzaldehyde (2f)

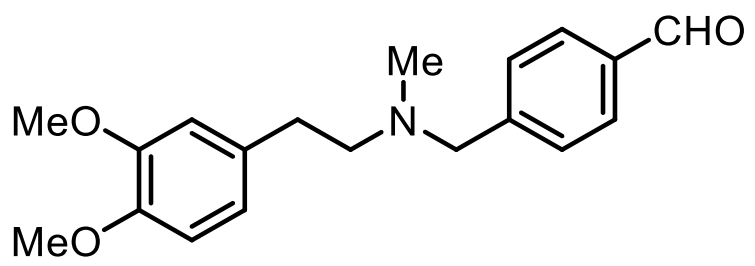

General procedure **GP2** was followed, using sulfonamide **1a** (80.9 mg, 0.15 mmol, 1.00 equiv.) and 4-(hydroxymethyl)benzaldehyde (24.5 mg, 0.18 mmol, 1.20 equiv.). The resulting crude material was subjected to flash column chromatography (silica gel, 0%-20% MeOH in EtOAc) to obtain the title compound as a colourless oil (38.0 mg, 0.121 mmol, 81% yield).

^{1}H NMR (400 MHz, $CDCl_3$): δ 9.99 (s, 1H), 7.81 (d, *J* = 8.2 Hz, 2H), 7.46 (d, *J* = 8.0 Hz, 2H), 6.79 (d, *J* = 8.0 Hz, 1H), 6.74 – 6.68 (m, 2H), 3.86 (s, 3H), 3.85 (s, 3H), 3.62 (s, 2H), 2.81 – 2.73 (m, 2H), 2.68 – 2.61 (m, 2H), 2.29 (s, 3H).

^{13}C NMR (101 MHz, $CDCl_3$): δ 192.1, 148.9, 147.5, 146.9, 135.5, 133.1, 129.9 (2C), 129.4 (2C), 120.7, 112.2, 111.3, 62.1, 59.6, 56.1, 55.9, 42.5, 33.7 ppm.

IR (neat) ν_{max}: 2936, 2833, 2789, 1695, 1606, 1514, 1260, 1234, 1140, 1027, 807 cm^{-1}.

HRMS (ESI$^+$): exact mass calculated for $[M+H]^+$ $(C_{19}H_{24}NO_3)^+$ requires *m/z* 314.1751, found *m/z* 314.1752.

Rf: 0.29 EtOAc:MeOH 10:1.

(*E*)-*N*-(3,4-Dimethoxyphenethyl)-*N*,3,7-trimethylocta-2,6-dien-1-amine (2g)

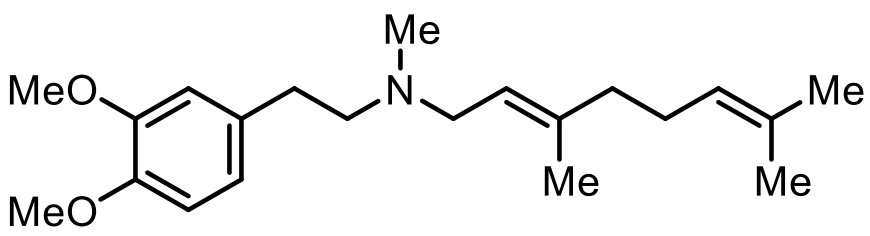

General procedure **GP2** was followed, using sulfonamide **1a** (80.9 mg, 0.15 mmol, 1.00 equiv.) and geraniol (27.8 mg, 0.18 mmol, 1.20 equiv.). The resulting crude material was subjected to flash column chromatography (silica gel, 0%-20% MeOH in EtOAc) to obtain the title compound as a pale-yellow oil (30.8 mg, 0.093 mmol, 62% yield).

^{1}H NMR (400 MHz, CDCl$_3$): δ 6.81 – 6.77 (m, 1H), 6.75 – 6.71 (m, 2H), 5.32 – 5.26 (m, 1H), 5.11 – 5.05 (m, 1H), 3.86 (s, 3H), 3.85 (s, 3H), 3.14 (d, *J* = 7.0 Hz, 2H), 2.84 – 2.76 (m, 2H), 2.71 – 2.64 (m, 2H), 2.35 (s, 3H), 2.13 – 2.02 (m, 4H), 1.66 (s, 6H), 1.59 (s, 3H) ppm.

^{13}C NMR (101 MHz, CDCl$_3$): δ 149.0, 147.6, 140.2, 132.5, 131.8, 124.1, 120.6, 119.9, 112.1, 111.4, 59.1, 56.1, 56.0, 55.0, 41.7, 39.9, 33.3, 26.5, 25.8, 17.8, 16.6 ppm.

IR (neat) ν_{max}: 2929, 2834, 1515, 1452, 1417, 1261, 1235, 1030, 802 cm^{-1}.

HRMS (ESI$^+$): exact mass calculated for [M+H]$^+$ ($C_{21}H_{34}NO_2$)$^+$ requires *m/z* 332.2584, found *m/z* 332.2576.

Rf: 0.38 EtOAc:MeOH 5:1.

***N*-(3,4-Dimethoxyphenethyl)-*N*-methyl-3-phenylpropan-1-amine (2h)**

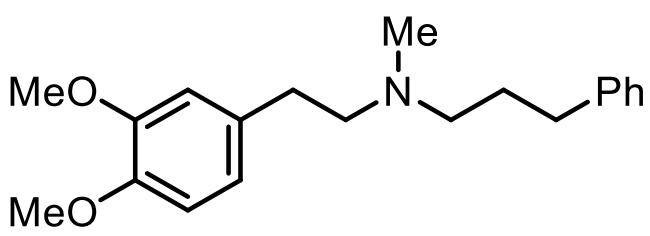

General procedure **GP2** was followed, using sulfonamide **1a** (80.9 mg, 0.15 mmol, 1.00 equiv.) and 3-phenylpropan-1-ol (24.5 mg, 0.18 mmol, 1.20 equiv.). The resulting crude material was subjected to flash column chromatography (silica gel, 0%-20% MeOH in EtOAc) to obtain the title compound as a pale-yellow oil (37.9 mg, 0.121 mmol, 81% yield).

^{1}H NMR (400 MHz, CDCl$_3$): δ 7.32 – 7.24 (m, 2H), 7.22 – 7.15 (m, 3H), 6.82 – 6.77 (m, 1H), 6.75 – 6.70 (m, 2H), 3.87 (s, 3H), 3.85 (s, 3H), 2.76 – 2.70 (m, 2H), 2.67 – 2.58 (m, 4H), 2.50 – 2.44 (m, 2H), 2.33 (s, 3H), 1.84 (p, *J* = 7.6 Hz, 2H) ppm.

^{13}C NMR (101 MHz, CDCl$_3$): δ 149.0, 147.5, 142.2, 133.1, 128.51 (2C), 128.45 (2C), 125.9, 120.6, 112.1, 111.4, 59.8, 57.2, 56.1, 56.0, 42.2, 33.8, 33.5, 29.0 ppm.

IR (neat) ν_{max}: 2936, 2834, 2788, 1514, 1453, 1260, 1234, 1140, 1028, 699 cm^{-1}.

HRMS (ESI$^+$): exact mass calculated for [M+H]$^+$ ($C_{20}H_{28}NO_2$)$^+$ requires *m/z* 314.2115, found *m/z* 314.2110.

Rf: 0.16 EtOAc:MeOH 10:1.

N-(2-(1H-Indol-3-yl)ethyl)-2-(3,4-dimethoxyphenyl)-N-methylethan-1-amine (2i)

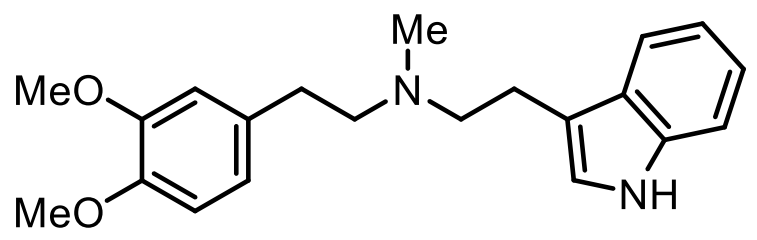

General procedure **GP2** was followed, using sulfonamide **1a** (80.9 mg, 0.15 mmol, 1.00 equiv.) and tryptophol (29.0 mg, 0.18 mmol, 1.20 equiv.). The resulting crude material was subjected to flash column chromatography (silica gel, 0%-20% MeOH in EtOAc) to obtain the title compound as a pale-yellow oil (28.1 mg, 0.083 mmol, 55% yield).

^{1}H NMR (400 MHz, $CDCl_3$): δ 8.10 (br s, 1H), 7.61 (d, *J* = 7.9 Hz, 1H), 7.36 (d, *J* = 8.1 Hz, 1H), 7.23 – 7.16 (m, 1H), 7.15 – 7.09 (m, 1H), 7.02 (d, *J* = 2.2 Hz, 1H), 6.82 – 6.77 (m, 1H), 6.76 – 6.70 (m, 2H), 3.86 (s, 3H), 3.86 (s, 3H), 3.06 – 3.00 (m, 2H), 2.91 – 2.76 (m, 6H), 2.51 (s, 3H) ppm.

^{13}C NMR (101 MHz, $CDCl_3$): δ 149.0, 147.6, 136.4, 132.6, 127.5, 122.2, 121.8, 120.7, 119.5, 118.8, 113.9, 112.2, 111.5, 111.3, 59.7, 58.1, 56.1, 56.0, 42.1, 33.2, 23.0 ppm.

IR (neat) ν_{max}: 3364, 2932, 2834, 1514, 1455, 1260, 1232, 1140, 1025, 738 cm^{-1}.

HRMS (ESI$^+$): exact mass calculated for $[M+H]^+$ $(C_{21}H_{27}N_2O_2)^+$ requires *m/z* 339.2067, found *m/z* 339.2060.

Rf: 0.31 EtOAc:MeOH 5:1.

4-((tert-Butyldimethylsilyl)oxy)-N-(3,4-dimethoxyphenethyl)-N-methylbutan-1-amine (2j)

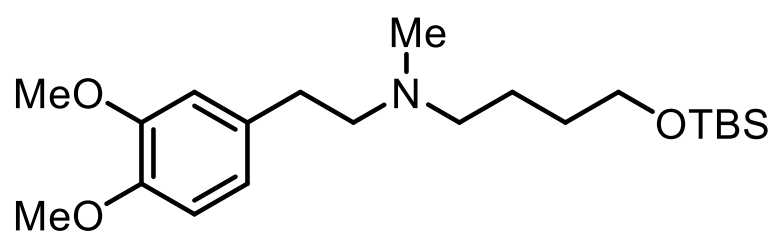

General procedure **GP2** was followed, using sulfonamide **1a** (80.9 mg, 0.15 mmol, 1.00 equiv.) and 4-((*tert*-butyldimethylsilyl)oxy)butan-1-ol (36.8 mg, 0.18 mmol, 1.20 equiv.). The resulting crude material was subjected to flash column chromatography (silica gel, 100% EtOAc) to obtain the title compound as a pale-yellow oil (46.5 mg, 0.122 mmol, 81% yield).

^{1}H NMR (400 MHz, $CDCl_3$): δ 6.81 – 6.77 (m, 1H), 6.75 – 6.71 (m, 2H), 3.87 (s, 3H), 3.85 (s, 3H), 3.62 (t, *J* = 6.0 Hz, 2H), 2.80 – 2.73 (m, 2H), 2.70 – 2.63 (m, 2H), 2.54 – 2.47 (m, 2H), 2.36 (s, 3H), 1.62 – 1.49 (m, 4H), 0.89 (s, 9H), 0.04 (s, 6H) ppm.

^{13}C NMR (101 MHz, $CDCl_3$): δ 149.0, 147.5, 132.7, 120.6, 112.1, 111.4, 63.1, 59.5, 57.3, 56.1, 56.0, 41.9, 33.1, 30.8, 26.1 (3C), 23.3, 18.5, -5.2 (2C) ppm.

IR (neat) ν_{max}: 2929, 2856, 1515, 1463, 1257, 1236, 1098, 1031, 834, 774 cm^{-1}.

HRMS (ESI$^+$): exact mass calculated for $[M+H]^+$ $(C_{21}H_{40}NO_3{}^{28}Si)^+$ requires *m/z* 382.2772, found *m/z* 382.2761.

Rf: 0.22 EtOAc.

N-(3,4-Dimethoxyphenethyl)-N-methyl-4-(4-nitrophenoxy)butan-1-amine (2k)

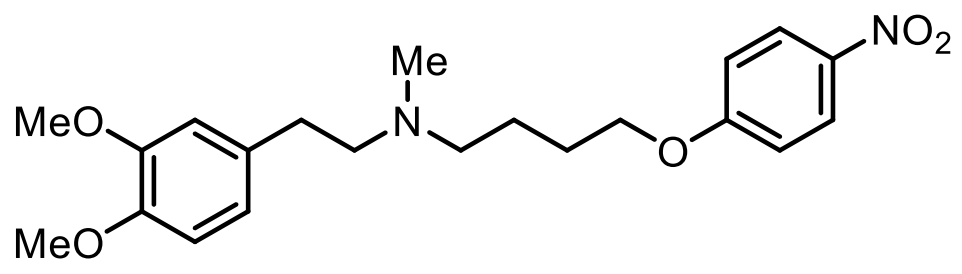

General procedure **GP2** was followed, using sulfonamide **1a** (80.9 mg, 0.15 mmol, 1.00 equiv.) and 4-(4-nitrophenoxy)butan-1-ol[59] (38.0 mg, 0.18 mmol, 1.20 equiv.). The resulting crude material was subjected to flash column chromatography (silica gel, 0%-20% MeOH in EtOAc) to obtain the title compound as a yellow oil (44.4 mg, 0.114 mmol, 76% yield).

^{1}H NMR (400 MHz, CDCl$_3$): δ 8.21 – 8.15 (m, 2H), 6.95 – 6.89 (m, 2H), 6.80 – 6.76 (m, 1H), 6.75 – 6.71 (m, 2H), 4.05 (t, J = 6.3 Hz, 2H), 3.86 (s, 3H), 3.84 (s, 3H), 2.79 – 2.73 (m, 2H), 2.68 – 2.63 (m, 2H), 2.55 – 2.50 (m, 2H), 2.35 (s, 3H), 1.89 – 1.80 (m, 2H), 1.74 – 1.65 (m, 2H) ppm.

^{13}C NMR (101 MHz, CDCl$_3$): δ 164.2, 149.0, 147.6, 141.5, 132.8, 126.0 (2C), 120.6, 114.5 (2C), 112.2, 111.4, 68.7, 59.8, 57.2, 56.1, 56.0, 42.0, 33.4, 27.0, 23.7 ppm.

IR (neat) ν_{max}: 2933, 1591, 1510, 1331, 1258, 1109, 1027, 844, 752 cm^{-1}.

HRMS (ESI$^+$): exact mass calculated for $[M+H]^+$ $(C_{21}H_{29}N_2O_5)^+$ requires *m/z* 389.2071, found *m/z* 389.2058.

Rf: 0.26 EtOAc:MeOH 5:1.

2-(3,4-Dimethoxyphenethyl)isoindoline (2l)

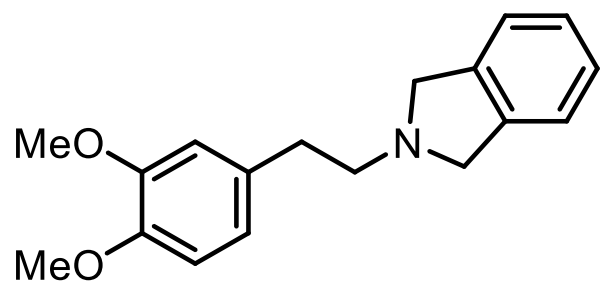

A modified general procedure **GP2** was followed, using sulfonamide **S3** (116.0 mg, 0.18 mmol, 1.20 equiv.) without addition of lithium iodide. The resulting crude material was subjected to flash column chromatography (silica gel, 0%-70% EtOAc in *n*-heptane) to obtain the title compound as a pale-yellow solid (36.2 mg, 0.128 mmol, 85% yield).

^{1}H NMR (400 MHz, CDCl$_3$): δ 7.25 – 7.17 (m, 4H), 6.84 – 6.76 (m, 3H), 4.02 (s, 4H), 3.89 (s, 3H), 3.87 (s, 3H), 3.02 – 2.95 (m, 2H), 2.91 – 2.83 (m, 2H) ppm.

^{13}C NMR (101 MHz, CDCl$_3$): δ 149.0, 147.5, 140.0 (2C), 132.9, 126.9 (2C), 122.4 (2C), 120.6, 112.1, 111.4, 59.3 (2C), 58.2, 56.0, 56.0, 35.4 ppm.

IR (neat) ν_{max}: 2934, 2784, 1514, 1463, 1263, 1237, 1142, 1029, 743 cm^{-1}.

HRMS (ESI$^+$): exact mass calculated for $[M+H]^+$ $(C_{18}H_{22}NO_2)^+$ requires *m/z* 284.1645, found *m/z* 284.1644.

Rf: 0.19 *n*-heptane:EtOAc 1:1.

N-(3,4-Dimethoxyphenethyl)-3-(4,5-diphenyloxazol-2-yl)-N-methylpropan-1-amine (2m)

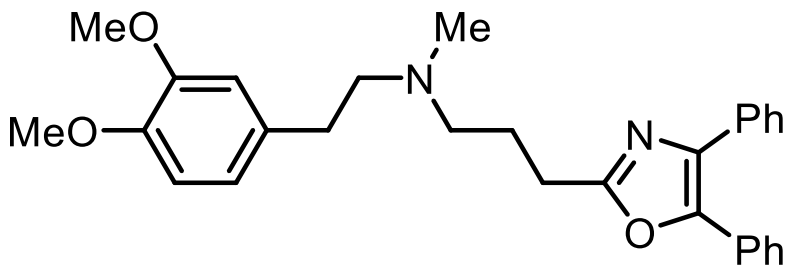

General procedure **GP2** was followed, using sulfonamide **1a** (80.9 mg, 0.15 mmol, 1.00 equiv.) and 3-(4,5-diphenyloxazol-2-yl)propan-1-ol[60] (50.3 mg, 0.18 mmol, 1.20 equiv.). The resulting crude material was subjected to flash column chromatography (silica gel, 0%-20% MeOH in EtOAc) to obtain the title compound as a pale-yellow oil (48.2 mg, 0.106 mmol, 70% yield).

^{1}H NMR (400 MHz, $CDCl_3$): δ 7.66 – 7.61 (m, 2H), 7.60 – 7.54 (m, 2H), 7.40 – 7.27 (m, 6H), 6.80 – 6.75 (m, 1H), 6.75 – 6.70 (m, 2H), 3.85 (s, 3H), 3.84 (s, 3H), 2.90 (t, *J* = 7.5 Hz, 2H), 2.81 – 2.74 (m, 2H), 2.73 – 2.60 (m, 4H), 2.39 (s, 3H), 2.09 (p, *J* = 7.5 Hz, 2H) ppm.

^{13}C NMR (101 MHz, $CDCl_3$): δ 163.3, 149.0, 147.5, 145.3, 135.2, 132.8, 132.7, 129.2, 128.8 (2C), 128.7 (2C), 128.5, 128.2, 128.0 (2C), 126.6, 120.6 (2C), 112.1, 111.4, 59.6, 56.7, 56.0, 56.0, 42.1, 33.3, 26.1, 24.5 ppm.

IR (neat) ν_{max}: 2934, 2834, 2790, 1514, 1445, 1260, 1234, 1140, 1026, 762, 693 cm^{-1}.

HRMS (ESI$^+$): exact mass calculated for $[M+H]^+$ $(C_{29}H_{33}N_2O_3)^+$ requires *m/z* 457.2486, found *m/z* 457.2481.

Rf: 0.26 EtOAc:MeOH 10:1.

(3a*S*,4*S*,6a*R*)-4-(5-((3,4-Dimethoxyphenethyl)(methyl)amino)pentyl)tetrahydro-1*H*-thieno[3,4-*d*]imidazol-2(3*H*)-one (2n)

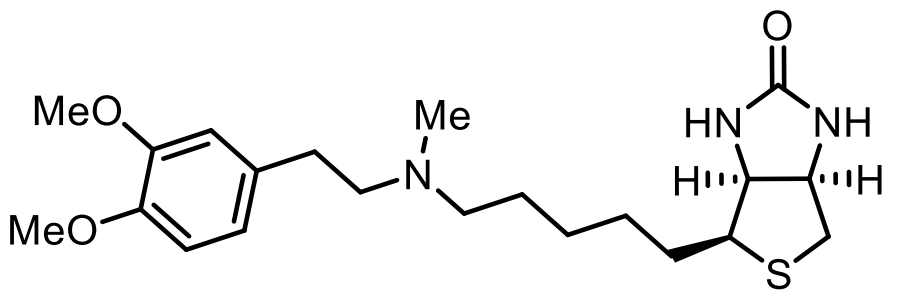

General procedure **GP2** was followed, using sulfonamide **1a** (80.9 mg, 0.15 mmol, 1.00 equiv.) and (3a*S*,4*S*,6a*R*)-4-(5-hydroxypentyl)tetrahydro-1*H*-thieno[3,4-d]imidazol-2(3*H*)-one[61] (41.5 mg, 0.18 mmol, 1.20 equiv.). The resulting crude material was subjected to flash column chromatography (silica gel, 20%-70% MeOH in DCM) to obtain the title compound as a colourless solid (36.5 mg, 0.090 mmol, 60% yield).

^{1}H NMR (400 MHz, CD_3OD): δ 6.87 (d, *J* = 8.2 Hz, 1H), 6.83 (d, *J* = 1.9 Hz, 1H), 6.76 (dd, *J* = 8.1, 1.9 Hz, 1H), 4.49 (dd, *J* = 7.8, 4.8 Hz, 1H), 4.30 (dd, *J* = 7.9, 4.5 Hz, 1H), 3.82 (s, 3H), 3.79 (s, 3H), 3.20 (dt, *J* = 9.5, 5.3 Hz, 1H), 2.92 (dd, *J* = 12.7, 5.0 Hz, 1H), 2.78 – 2.60 (m, 5H), 2.50 – 2.42 (m, 2H), 2.34 (s, 3H), 1.78 – 1.66 (m, 1H), 1.64 – 1.49 (m, 3H), 1.49 – 1.31 (m, 4H) ppm. *(The signals for NH groups could not be detected)*.

^{13}C NMR (101 MHz, CD_3OD): δ 166.2, 150.5, 149.0, 134.2, 122.0, 113.8, 113.3, 63.5, 61.7, 60.5, 58.4, 57.2, 56.6, 56.5, 42.3, 41.1, 33.6, 30.2, 29.8, 28.5, 27.5 ppm.

IR (neat) ν_{max}: 3234, 2935, 2846, 1682, 1518, 1467, 1262, 1235, 1140, 1026, 805 cm^{-1}.

HRMS (ESI$^+$): exact mass calculated for $[M+H]^+$ $(C_{21}H_{34}N_3O_3{}^{32}S)^+$ requires *m/z* 408.2315, found *m/z* 408.2299.

Rf: 0.14 DCM:MeOH 5:1.

$[\alpha]_D^{23}$ = 49.2 (*c* = 0.50, MeOH).

(*E*)-*N*-(3,4-Dimethoxyphenethyl)-2-(5-fluoro-2-methyl-1-(4-(methylsulfinyl)benzylidene)-1*H*-inden-3-yl)-*N*-methylethan-1-amine (2o)

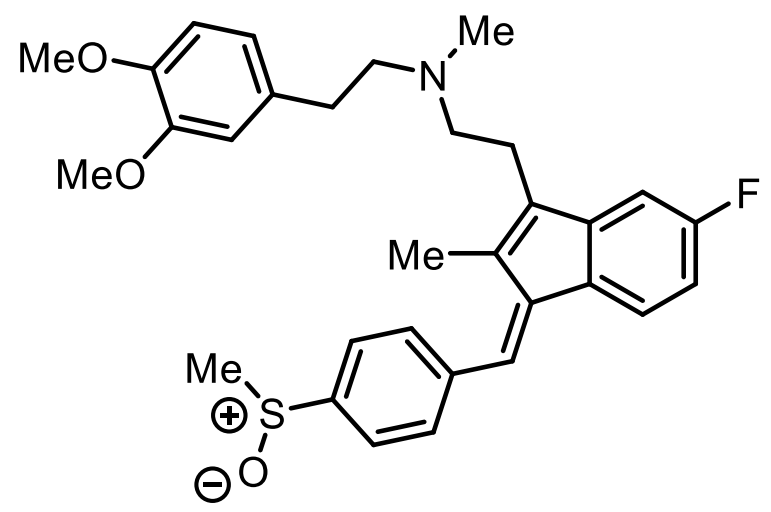

General procedure **GP2** was followed, using sulfonamide **1a** (80.9 mg, 0.15 mmol, 1.00 equiv.) and **S4** (61,6 mg, 0.18 mmol, 1.20 equiv.). The resulting crude material was subjected to flash column chromatography (silica gel, 0%-20% MeOH in EtOAc) to obtain the title compound as a yellow oil (39.6 mg, 0.076 mmol, 51% yield).

^{1}H NMR (400 MHz, $CDCl_3$): δ 7.71 (d, *J* = 8.3 Hz, 2H), 7.65 (d, *J* = 8.2 Hz, 2H), 7.14 (dd, *J* = 8.3, 5.2 Hz, 1H), 7.08 (s, 1H), 6.87 – 6.78 (m, 2H), 6.78 – 6.72 (m, 2H), 6.55 (td, *J* = 8.9, 2.3 Hz, 1H), 3.88 (s, 3H), 3.86 (s, 3H), 2.84 – 2.68 (m, 9H), 2.68 – 2.60 (m, 2H), 2.48 (s, 3H), 2.16 (s, 3H) ppm.

^{13}C NMR (101 MHz, $CDCl_3$): δ 163.5 (d, $^1J_{C\text{-}F}$ = 246.4 Hz), 149.0, 147.6, 147.4 (d, $^3J_{C\text{-}F}$ = 8.4 Hz), 145.4, 142.1, 140.0, 137.7 (d, $^4J_{C\text{-}F}$ = 3.4 Hz), 136.2, 132.8, 130.4 (2C), 130.0 (d, $^4J_{C\text{-}F}$ = 2.9 Hz), 127.2, 123.9 (2C), 123.8 (d, $^3J_{C\text{-}F}$ = 9.1 Hz), 120.7, 112.1, 111.5, 110.6 (d, $^2J_{C\text{-}F}$ = 22.7 Hz), 105.8 (d, $^2J_{C\text{-}F}$ = 23.6 Hz), 59.6, 56.1, 56.00, 55.96, 44.1, 42.2, 33.6, 23.6, 10.5 ppm.

^{19}F NMR (376 MHz, $CDCl_3$): δ -113.06 – -113.18 (m, 1F) ppm.

IR (neat) ν_{max}: 2935, 2835, 1600, 1514, 1464, 1261, 1155, 1028, 810, 701 cm^{-1}.

HRMS (ESI$^+$): exact mass calculated for $[M+H]^+$ $(C_{31}H_{35}FNO_3{}^{32}S)^+$ requires *m/z* 520.2316, found *m/z* 520.2317.

Rf: 0.48 EtOAc:MeOH 5:1.

5-(5-(((3,4-Dimethoxyphenethyl)(methyl)amino)methyl)-4-methylthiazol-2-yl)-2-isobutoxybenzonitrile (2p)

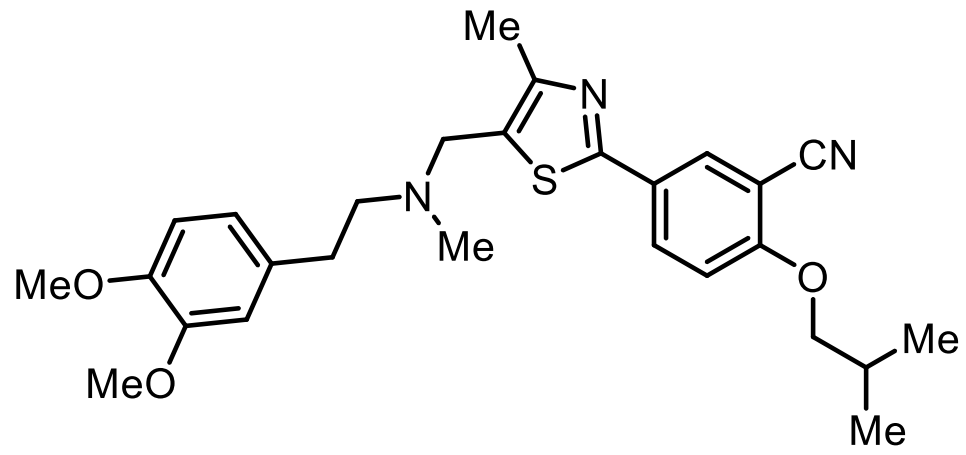

General procedure **GP2** was followed, using sulfonamide **1a** (80.9 mg, 0.15 mmol, 1.00 equiv.) and **S5** (54.4 mg, 0.18 mmol, 1.20 equiv.). The resulting crude material was subjected to flash column chromatography (silica gel, 0%-70% EtOAc in *n*-heptane) to obtain the title compound as a colourless oil (48.3 mg, 0.101 mmol, 67% yield).

^{1}H NMR (400 MHz, $CDCl_3$): δ 8.09 (d, *J* = 2.1 Hz, 1H), 7.99 (dd, *J* = 8.8, 2.1 Hz, 1H), 6.97 (d, *J* = 8.9 Hz, 1H), 6.79 (d, *J* = 8.0 Hz, 1H), 6.76 – 6.68 (m, 2H), 3.95 – 3.79 (m, 8H), 3.68 (s, 2H), 2.81 – 2.72 (m, 2H), 2.72 – 2.63 (m, 2H), 2.39 (s, 3H), 2.35 (s, 3H), 2.19 (dp, *J* = 13.2, 6.6 Hz, 1H), 1.08 (d, *J* = 6.7 Hz, 6H) ppm.

^{13}C NMR (101 MHz, $CDCl_3$): δ 163.3, 161.7, 150.2, 149.0, 147.5, 132.9, 132.0, 131.4, 131.0, 127.2, 120.7, 115.9, 112.6, 112.2, 111.4, 102.8, 75.7, 59.2, 56.1, 56.0, 53.5, 42.3, 33.8, 28.3, 19.2 (2C), 15.5 ppm.

IR (neat) ν_{max}: 2932, 2223, 1606, 1507, 1449, 1261, 1235, 1141, 1011, 731 cm^{-1}.

HRMS (ESI^{+}): exact mass calculated for $[M+H]^+$ ($C_{27}H_{34}N_3O_3{}^{32}S)^+$ requires *m/z* 480.2315, found *m/z* 480.2314.

Rf: 0.24 *n*-heptane:EtOAc 1:1.

2-(3,4-Dimethoxyphenyl)-*N*-(4-(5-(2-fluorophenyl)-1,2,4-oxadiazol-3-yl)benzyl)-*N*-methylethan-1-amine (2q)

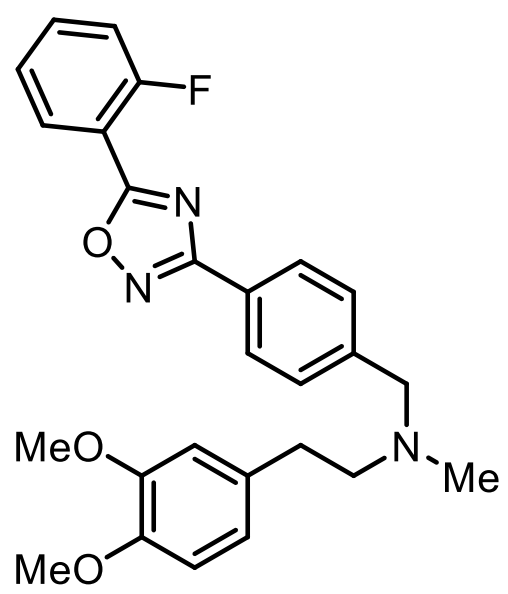

General procedure **GP2** was followed, using sulfonamide **1a** (80.9 mg, 0.15 mmol, 1.00 equiv.) and benzyl alcohol **S6** (48.6 mg, 0.18 mmol, 1.20 equiv.). The resulting crude material was subjected to flash column chromatography (silica gel, 20%-100% EtOAc in *n*-heptane) to obtain the title compound as a colourless oil (56.1 mg, 0.125 mmol, 84% yield).

^{1}H NMR (400 MHz, $CDCl_3$): δ 8.22 (td, *J* = 7.8, 1.7 Hz, 1H), 8.13 (s, 1H), 8.07 (dt, *J* = 7.1, 1.6 Hz, 1H), 7.63 – 7.56 (m, 1H), 7.50 – 7.42 (m, 2H), 7.36 – 7.25 (m, 2H), 6.78 (d, *J* = 7.9 Hz, 1H), 6.75 – 6.70 (m, 2H), 3.84 (s, 6H), 3.66 (s, 2H), 2.84 – 2.78 (m, 2H), 2.72 – 2.65 (m, 2H), 2.33 (s, 3H) ppm.

^{13}C NMR (101 MHz, $CDCl_3$): δ 172.8 (d, $^{3}J_{C\text{-}F}$ = 4.3 Hz), 168.9, 160.9 (d, $^{1}J_{C\text{-}F}$ = 260.5 Hz), 148.9, 147.4, 139.9, 134.7 (d, $^{3}J_{C\text{-}F}$ = 8.6 Hz), 133.1, 132.1, 131.1 (d, $^{4}J_{C\text{-}F}$ = 1.2 Hz), 129.0, 128.2, 126.8, 126.5, 124.8 (d, $^{3}J_{C\text{-}F}$ = 3.8 Hz), 120.7, 117.3 (d, $^{2}J_{C\text{-}F}$ = 20.9 Hz), 113.0 (d, $^{2}J_{C\text{-}F}$ = 11.4 Hz), 112.1, 111.3, 61.9, 59.4, 56.0, 55.9, 42.3, 33.6 ppm.

^{19}F NMR (376 MHz, CDCl$_3$): δ -108.28 – -108.37 (m, 1F) ppm.

IR (neat) ν_{max}: 2935, 2834, 1514, 1461, 1233, 1141, 1028, 752 cm^{-1}.

HRMS (ESI$^+$): exact mass calculated for [M+H]$^+$ ($C_{26}H_{27}FN_3O_3$)$^+$ requires *m/z* 448.2031, found *m/z* 448.2027.

Rf: 0.35 *n*-heptane:EtOAc 1:2.

(3*R*,5*R*,8*R*,9*S*,10*S*,13*R*,14*S*,17*R*)-17-((*R*)-5-((3,4-Dimethoxyphenethyl)(methyl)amino)pentan-2-yl)-10,13-dimethylhexadecahydro-1*H*-cyclopenta[*a*]phenanthren-3-ol (2r)

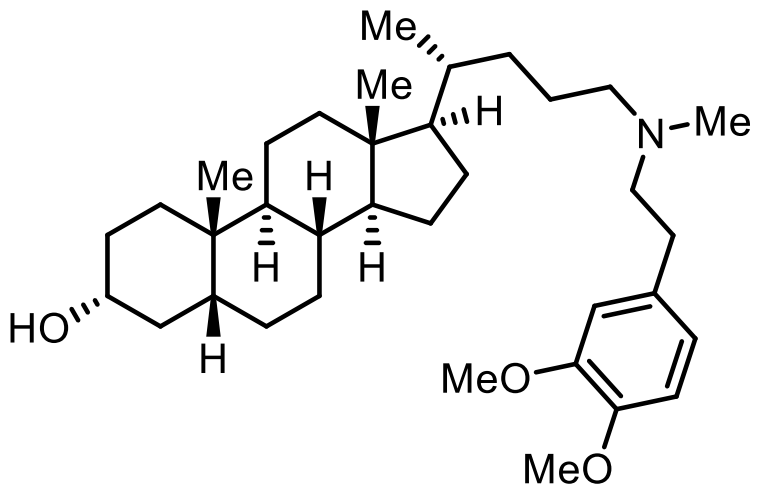

General procedure **GP2** was followed, using sulfonamide **1a** (80.9 mg, 0.15 mmol, 1.00 equiv.) and 5β-cholane-3α,24-diol[62] (65.3 mg, 0.18 mmol, 1.20 equiv.). The resulting crude material was subjected to flash column chromatography (silica gel, 100% EtOAc) to obtain the title compound as a colourless oil (54.2 mg, 0.101 mmol, 67% yield).

^{1}H NMR (400 MHz, CDCl$_3$): δ 6.81 – 6.77 (m, 1H), 6.76 – 6.71 (m, 2H), 3.87 (s, 3H), 3.85 (s, 3H), 3.61 (tt, *J* = 10.9, 4.6 Hz, 1H), 2.83 – 2.76 (m, 2H), 2.73 – 2.65 (m, 2H), 2.51 – 2.43 (m, 2H), 2.40 (s, 3H), 1.98 – 1.92 (m, 1H), 1.88 – 1.72 (m, 4H), 1.66 – 1.48 (m, 4H), 1.43 – 1.32 (m, 8H), 1.28 – 1.15 (m, 5H), 1.13 – 0.94 (m, 7H), 0.92 (d, *J* = 5.2 Hz, 6H), 0.63 (s, 3H) ppm.

^{13}C NMR (101 MHz, CDCl$_3$): δ 149.1, 147.6, 132.4, 120.7, 112.2, 111.4, 72.0, 59.5, 58.2, 56.6, 56.3, 56.1, 56.0, 42.8, 42.2, 41.9, 40.6, 40.3, 36.6, 36.0, 35.8, 35.5, 34.7, 33.7, 33.0, 30.7, 28.5, 27.3, 26.6, 24.4, 23.5, 23.3, 21.0, 18.8, 12.2 ppm.

IR (neat) ν_{max}: 2926, 2861, 1516, 1449, 1261, 1235, 1141, 1030, 730 cm^{-1}.

HRMS (ESI$^+$): exact mass calculated for [M+H]$^+$ ($C_{35}H_{58}NO_3$)$^+$ requires *m/z* 540.4411, found *m/z* 540.4421.

Rf: 0.22 EtOAc 100%.

(*S*)-*N*-(3,4-Dimethoxyphenethyl)-*N*-methyl-1-phenylethan-1-amine (2s)

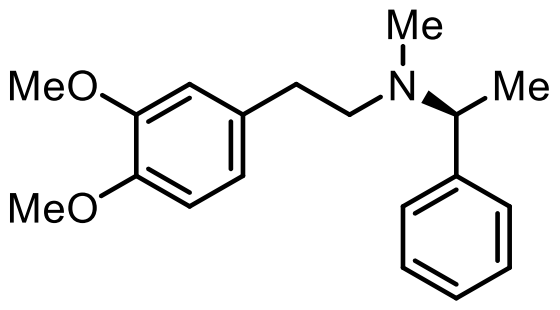

A modified general procedure **GP2** was followed, using sulfonamide **1a** (53.9 mg, 0.10 mmol, 1.00 equiv.) and (*R*)-(+)-1-phenylethyl alcohol (14.7 mg, 0.12 mmol, 1.20 equiv.). The reaction was performed for 72 h without lithium iodide. The resulting crude material was subjected to flash column chromatography (silica gel, 0%-70% EtOAc in *n*-heptane) to obtain the title compound as a pale-yellow solid (19.9 mg, 0.066 mmol, 67% yield).

^{1}H NMR (400 MHz, $CDCl_3$): δ 7.34 – 7.28 (m, 4H), 7.25 – 7.19 (m, 1H), 6.76 (d, J = 8.1 Hz, 1H), 6.68 – 6.60 (m, 2H), 3.85 (s, 3H), 3.83 (s, 3H), 3.64 (q, J = 6.7 Hz, 1H), 2.77 – 2.63 (m, 3H), 2.58 – 2.50 (m, 1H), 2.30 (s, 3H), 1.38 (d, J = 6.7 Hz, 3H) ppm.

^{13}C NMR (101 MHz, $CDCl_3$): δ 148.9, 147.4, 144.0, 133.4, 128.3 (2C), 127.8 (2C), 126.9, 120.7, 112.2, 111.3, 63.4, 56.6, 56.1, 55.9, 38.8, 33.4, 18.8 ppm.

IR (neat) ν_{max}: 2934, 2834, 2786, 1513, 1451, 1260, 1234, 1141, 1028, 701 cm^{-1}.

HRMS (ESI^{+}): exact mass calculated for $[M+H]^+$ $(C_{19}H_{26}NO_2)^+$ requires *m/z* 300.1958, found *m/z* 300.1956.

Rf: 0.34 *n*-heptane:EtOAc 1:1.

$[\alpha]_D^{25}$ = –16.3 (*c* = 0.71, $CHCl_3$).

Enantiomeric ratio: 94:6 determined by chiral HPLC analysis: Chiralcel OD-H, 50% (*n*-heptane/IPA/DEA 93:7:0.1) : 50% (*n*-heptane + 0.1% IPA), 0.7 mL/min, 25 °C, detection at 220 nm, retention time (min): 14.5 (major); 15.6 (minor).

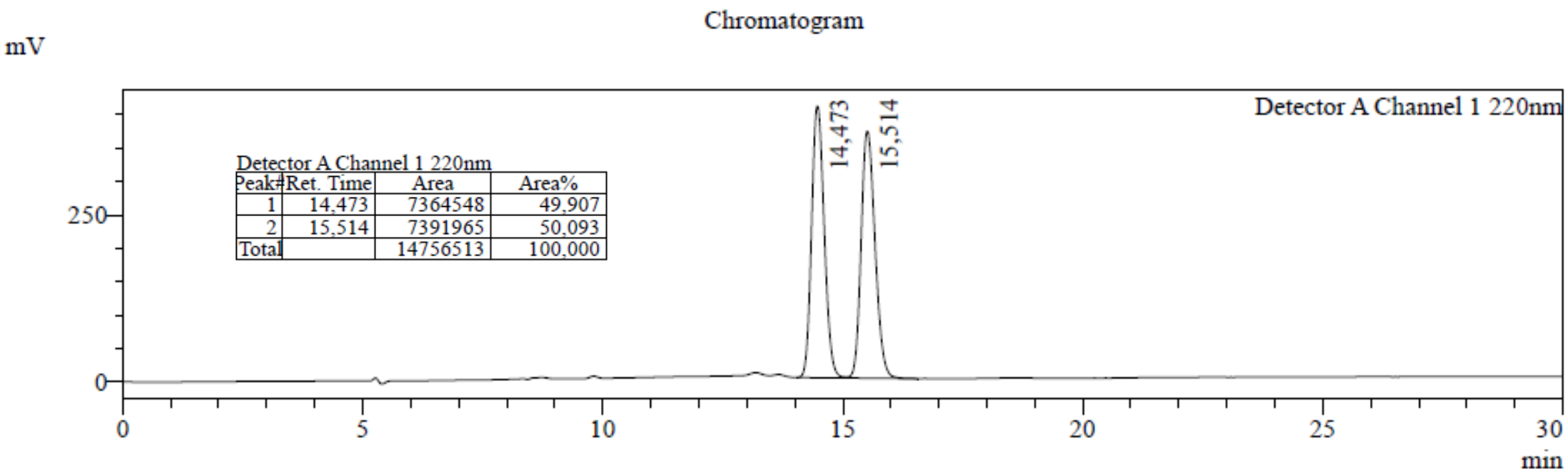

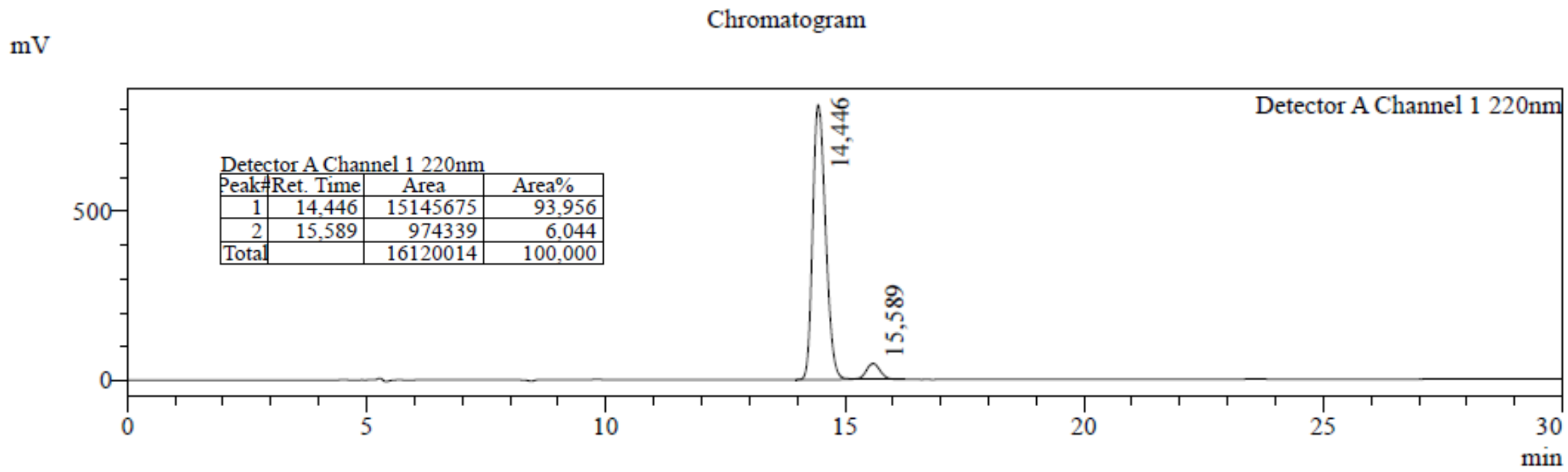

(3*R*,4*S*)-3-((*R*)-3-((3,4-Dimethoxyphenethyl)(methyl)amino)-3-(4-fluorophenyl)propyl)-1-(4-fluorophenyl)-4-(4-methoxyphenyl)azetidin-2-one (2t)

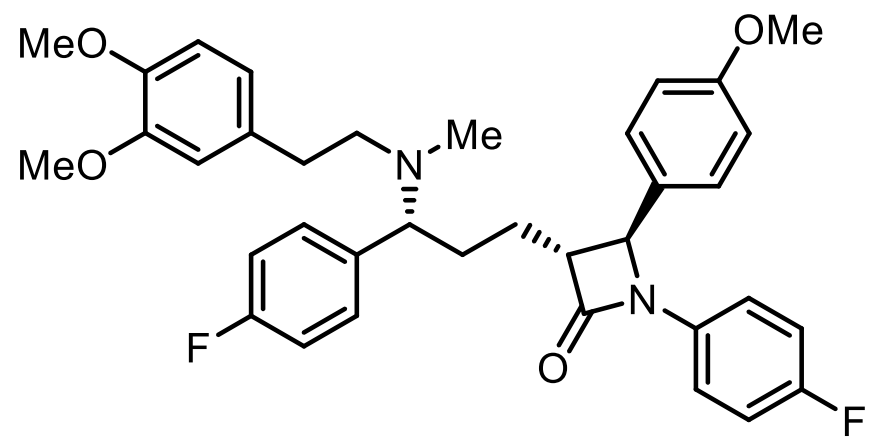

A modified general procedure **GP2** was followed, using sulfonamide **1a** (80.9 mg, 0.15 mmol, 1.00 equiv.) and (3*R*,4*S*)-1-(4-fluorophenyl)-3-((*S*)-3-(4-fluorophenyl)-3-hydroxypropyl)-4-(4-methoxyphenyl)azetidin-2-one (76.2 mg, 0.18 mmol, 1.20 equiv.). The reaction was performed for 72 h without lithium iodide. The resulting crude material was subjected to flash column chromatography (silica gel, 0%-70% EtOAc in *n*-heptane) to obtain the title compound as a yellow oil (46.2 mg, 0.077 mmol, 51% yield). The product was obtained as a mixture of two diastereoisomers in a ratio of 96:4. A single NMR peak in the ^{1}H NMR corresponding exclusively to the minor isomer is indicated with an asterisk (*).

^{1}H NMR (700 MHz, $CDCl_3$): δ 7.24 – 7.19 (m, 4H), 7.17 – 7.13 (m, 2H), 7.00 (t, *J* = 8.6 Hz, 2H), 6.93 – 6.87 (m, 4H), 6.75 (d, *J* = 8.1 Hz, 1H), 6.65 – 6.63 (m, 1H), 6.62 – 6.60 (m, 1H), 4.47 (d, *J* = 2.3 Hz, 0.04H*), 4.45 (d, *J* = 2.3 Hz, 0.96H), 3.83 (s, 3H), 3.82 (s, 3H), 3.79 (s, 3H), 3.46 (t, *J* = 6.9 Hz, 1H), 3.02 (td, *J* = 7.6, 7.0, 2.3 Hz, 1H), 2.70 – 2.57 (m, 3H), 2.47 – 2.41 (m, 1H), 2.24 (s, 3H), 2.17 – 2.11 (m, 1H), 1.86 – 1.75 (m, 2H), 1.61 – 1.54 (m, 1H) ppm.

DEPT-Q ^{13}C NMR (176 MHz, $CDCl_3$): δ 167.6, 162.1 (d, *J* = 245.4 Hz), 159.9, 159.0 (d, *J* = 243.2 Hz), 148.9, 147.4, 135.6, 134.1, 133.2, 130.0 (d, *J* = 7.7 Hz, 2C), 129.6, 127.3 (2C), 120.7, 118.4 (d, *J* = 7.7 Hz, 2C), 115.9 (d, *J* = 22.6 Hz, 2C), 115.1 (d, *J* = 21.0 Hz, 2C), 114.7 (2C), 112.2, 111.3, 67.9, 61.1, 60.8, 56.5, 56.0, 55.9, 55.4, 38.3, 33.4, 30.5, 26.1 ppm.

^{19}F NMR (659 MHz, $CDCl_3$): δ -115.43 (s, 1F), -118.24 (s, 1F) ppm.

IR (neat) ν_{max}: 2935, 1743, 1506, 1383, 1218, 1140, 1028, 910, 832, 729 cm^{-1}.

HRMS (ESI$^+$): exact mass calculated for $[M+H]^+$ $(C_{36}H_{39}F_2N_2O_4)^+$ requires *m/z* 601.2872, found *m/z* 601.2872.

Rf: 0.20 *n*-heptane:EtOAc 1:1.

$[\alpha]_D^{25}$ = 13.0 (*c* = 0.97, $CHCl_3$).

(*S*)-4-(1-((3,4-Dimethoxyphenethyl)(methyl)amino)ethyl)-*N*,*N*-dipropylbenzenesulfonamide (2u)

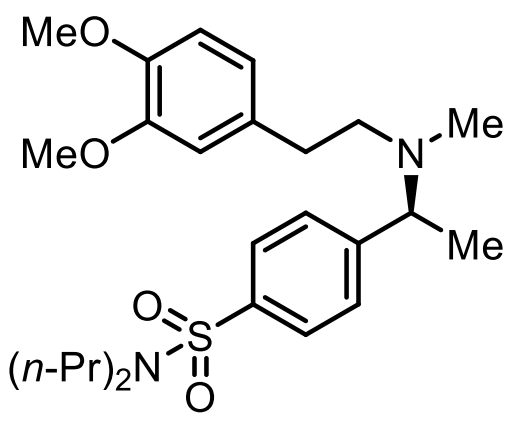

A modified general procedure **GP2** was followed, using sulfonamide **1a** (80.9 mg, 0.15 mmol, 1.00 equiv.) and **S7** (51.4 mg, 0.18 mmol, 1.20 equiv.). The reaction was performed without lithium iodide. The resulting crude material was subjected to flash column chromatography (silica gel, 30%-70% EtOAc in *n*-heptane) to obtain the title compound as a pale-yellow oil (34.9 mg, 0.075 mmol, 50% yield).

^{1}H NMR (400 MHz, CDCl$_3$): δ 7.72 (d, *J* = 8.4 Hz, 2H), 7.42 (d, *J* = 8.3 Hz, 2H), 6.79 – 6.74 (m, 1H), 6.65 – 6.61 (m, 2H), 3.85 (s, 3H), 3.84 (s, 3H), 3.67 (q, *J* = 6.7 Hz, 1H), 3.11 – 3.02 (m, 4H), 2.76 – 2.58 (m, 3H), 2.58 – 2.49 (m, 1H), 2.28 (s, 3H), 1.55 (h, *J* = 7.4 Hz, 4H), 1.36 (d, *J* = 6.7 Hz, 3H), 0.86 (t, *J* = 7.4 Hz, 6H) ppm.

^{13}C NMR (101 MHz, CDCl$_3$): δ 149.2, 148.9, 147.5, 138.7, 133.1, 128.2 (2C), 127.2 (2C), 120.7, 112.3, 111.4, 63.1, 56.6, 56.1, 56.0, 50.2 (2C), 38.6, 33.5, 22.2 (2C), 18.2, 11.3 (2C) ppm.

IR (neat) ν_{max}: 2964, 2933, 2874, 1515, 1463, 1335, 1260, 1153, 1029, 575 cm^{-1}.

HRMS (ESI$^+$): exact mass calculated for [M+H]$^+$ ($C_{25}H_{39}N_2O_4{}^{32}S$)$^+$ requires *m/z* 463.2625, found *m/z* 463.2615.

Rf: 0.18 *n*-heptane:EtOAc 1:1.

[α]$_D^{23}$ = –10.6 (*c* = 0.49, CH_2Cl_2).

Enantiomeric ratio: 96:4 determined by chiral HPLC analysis: Lux-Cellulose-3, *n*-heptane/EtOH 65:35, 0.7 mL/min, 25 °C, detection at 210 nm, retention time (min): 8.2 (major); 8.9 (minor).

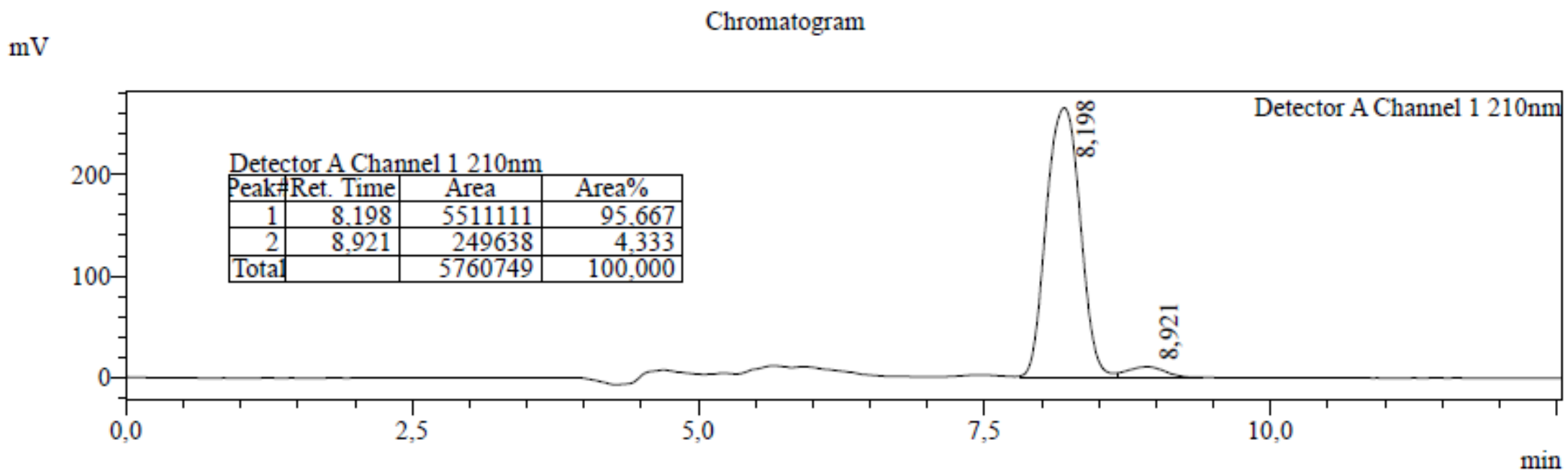

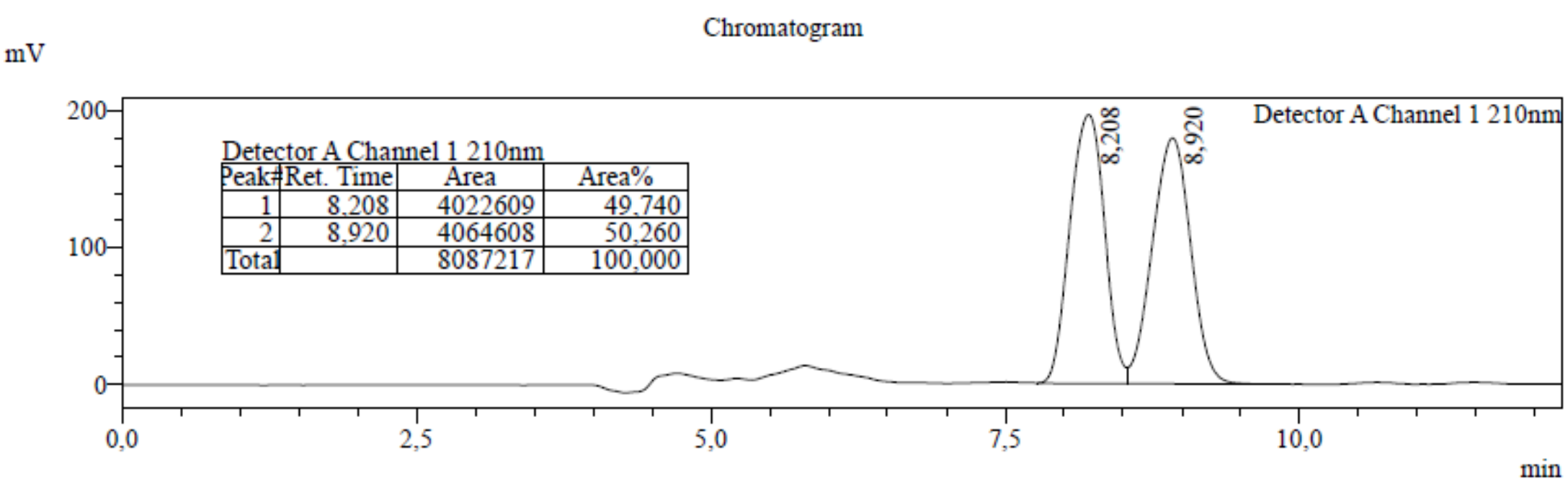

(*S*)-*N*-(3,4-Dimethoxyphenethyl)-*N*-methyl-3-(naphthalen-1-yloxy)-1-phenylpropan-1-amine (2v)

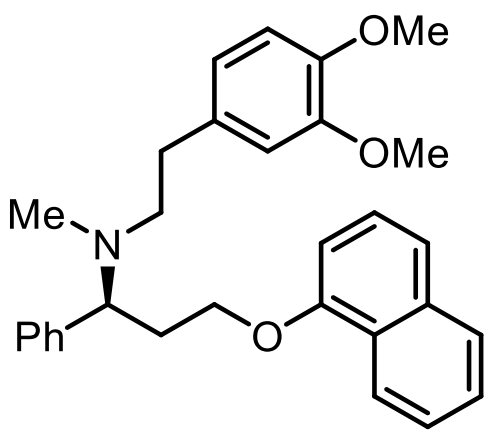

A modified general procedure **GP2** was followed, using sulfonamide **1a** (80.9 mg, 0.15 mmol, 1.00 equiv.) and **S8** (50.1 mg, 0.18 mmol, 1.20 equiv.). The reaction was performed for 72 h without lithium iodide. The resulting crude material was subjected to flash column chromatography (silica gel, 100% EtOAc) to obtain the title compound as an orange oil (37.6 mg, 0.083 mmol, 55% yield).

^{1}H NMR (400 MHz, $CDCl_3$): δ 8.24 – 8.19 (m, 1H), 7.79 – 7.73 (m, 1H), 7.48 – 7.40 (m, 2H), 7.37 (d, *J* = 8.2 Hz, 1H), 7.33 – 7.22 (m, 6H), 6.70 (d, *J* = 8.1 Hz, 1H), 6.67 – 6.59 (m, 3H), 4.11 – 4.04 (m, 1H), 3.97 – 3.89 (m, 2H), 3.80 (s, 3H), 3.78 (s, 3H), 2.76 – 2.64 (m, 3H), 2.61 – 2.49 (m, 2H), 2.33 – 2.22 (m, 4H) ppm.

^{13}C NMR (101 MHz, $CDCl_3$): δ 154.8, 148.9, 147.4, 139.5, 134.6, 133.4, 128.8 (2C), 128.3 (2C), 127.6, 127.4, 126.4, 126.0, 125.8, 125.2, 122.1, 120.7, 120.1, 112.2, 111.3, 104.8, 65.8, 65.3, 56.4, 56.0, 55.9, 38.4, 33.6, 32.5 ppm.

IR (neat) ν_{max}: 2934, 2833, 2791, 1579, 1513, 1460, 1265, 1235, 1098, 1027, 769, 702 cm^{-1}.

HRMS (ESI$^+$): exact mass calculated for $[M+H]^+$ $(C_{30}H_{34}NO_3)^+$ requires *m/z* 456.2533, found *m/z* 456.2532.

Rf: 0.19 EtOAc 100%.

$[\alpha]_D^{23}$ = 16.1 (*c* = 0.91, CH_2Cl_2).

Enantiomeric ratio: 99:1 determined by chiral HPLC analysis: Lux-Cellulose-1, 90% (*n*-heptane/IPA/DEA 98:2:0.1) : 10% IPA, 0.7 mL/min, 25 °C, detection at 230 nm, retention time (min): 16.8 (minor); 19.8 (major).

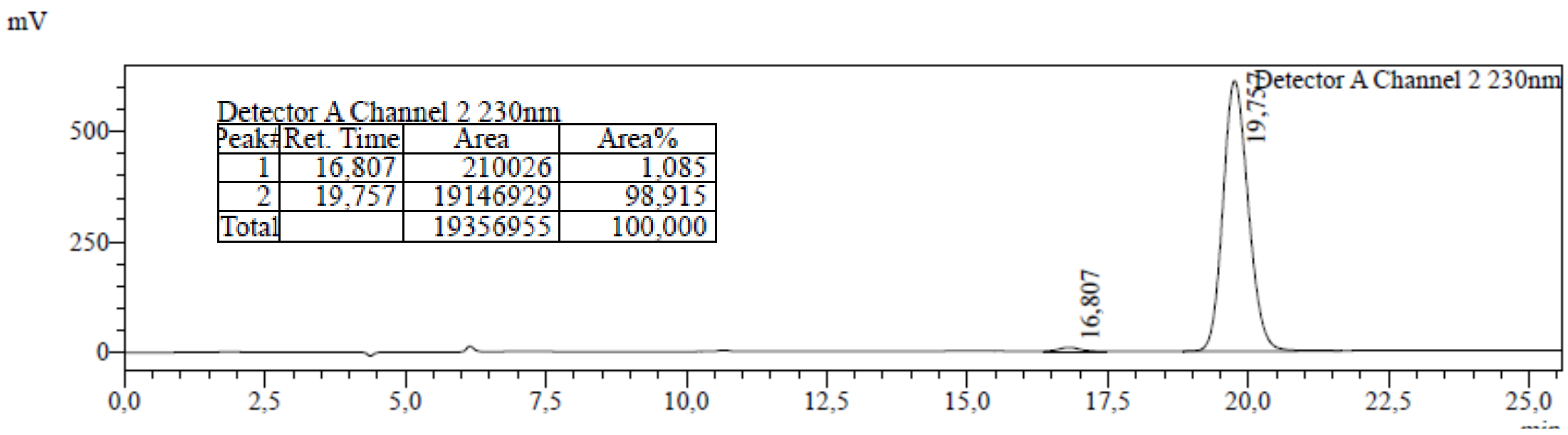

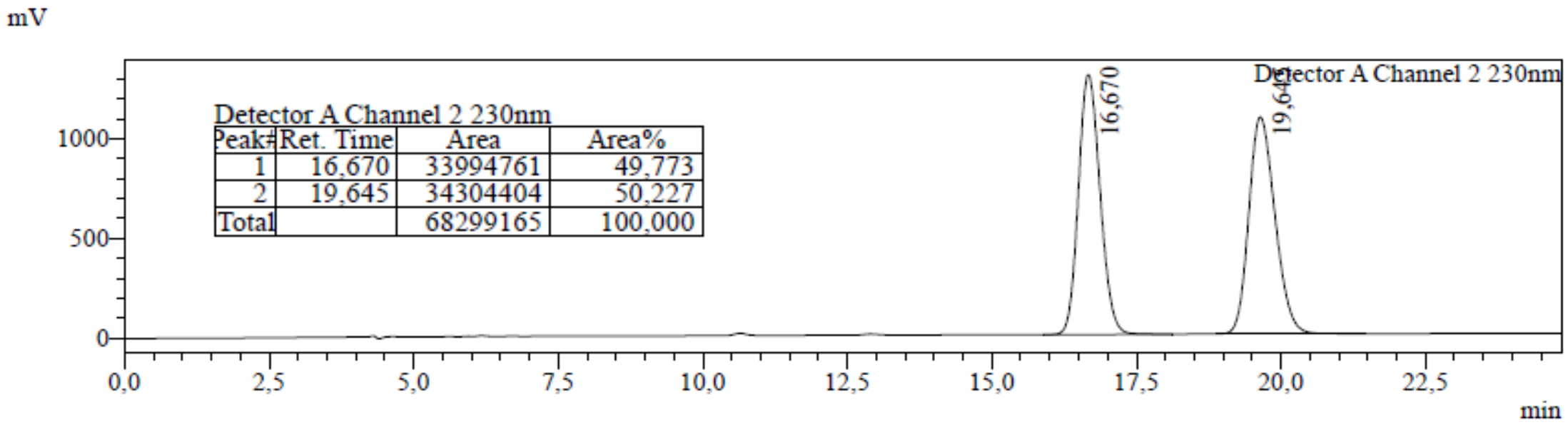

(*S*)-*N*-(3,4-Dimethoxyphenethyl)-*N*-methyl-1-phenylundec-4-yn-3-amine (2w)

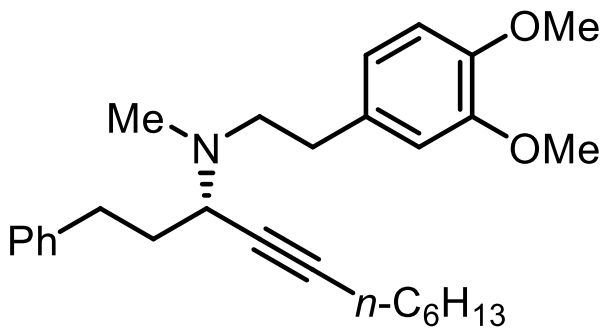

General procedure **GP2** was followed, using sulfonamide **1a** (80.9 mg, 0.15 mmol, 1.00 equiv.) and alcohol **S9** (44.0 mg, 0.18 mmol, 1.20 equiv.). The reaction was performed for 72 h without lithium iodide. The resulting crude material was subjected to flash column chromatography (silica gel, 100% 3:1:2% EtOAc:EtOH:NH_4OH followed by 1:1:2% EtOAc:*n*-heptane:NH_4OH) to obtain the title compound as a yellow oil (51.1 mg, 0.077 mmol, 70% yield).

^{1}H NMR (400 MHz, $CDCl_3$): δ 7.29 – 7.24 (m, 2H), 7.20 – 7.15 (m, 1H), 6.80 – 6.77 (m, 1H), 6.75 – 6.72 (m, 2H), 3.86 (s, 3H), 3.85 (s, 3H), 3.42 – 3.33 (m, 1H), 2.82 – 2.56 (m, 6H), 2.29 (s, 3H), 2.22 (td, *J* = 6.9, 2.1 Hz, 2H), 1.94 – 1.85 (m, 2H), 1.58 – 1.46 (m, 2H), 1.46 – 1.37 (m, 2H), 1.37 – 1.23 (m, 6H), 0.88 (d, *J* = 6.5 Hz, 3H) ppm.

^{13}C NMR (101 MHz, $CDCl_3$): δ 148.9, 147.4, 142.1, 133.5, 128.7 (2C), 128.4 (2C), 125.9, 120.7, 112.3, 111.3, 86.2, 77.4, 56.9, 56.1, 55.95, 55.92, 37.9, 36.0, 34.4, 32.9, 31.5, 29.3, 28.7, 22.7, 18.8, 14.2 ppm.

IR (neat) ν_{max}: 2930, 2856, 1737, 1591, 1515, 1454, 1417, 1332, 1261, 1235, 1155, 1140, 1030, 849, 804, 750, 700, 547 cm^{-1}.

HRMS (ESI$^+$): exact mass calculated for $[M+H]^+$ $(C_{28}H_{40}NO_2)^+$ requires *m/z* 422.3054, found *m/z* 422.3039.

R_f: 0.76 *n*-heptane:(EtOAc:EtOH:NH_4OH (3:1:2%)) 1:1.

$[\alpha]_D^{23}$ = -8.8 (*c* = 1.18, CH_2Cl_2).

Enantiomeric ratio: 96:4 determined by chiral HPLC analysis: Lux-Cellulose-1, *n*-heptane/IPA/DEA 93:7:0.1, 0.7 mL/min, 25 °C, detection at 220 nm, retention time (min): 9.4 (minor); 10.2 (major).

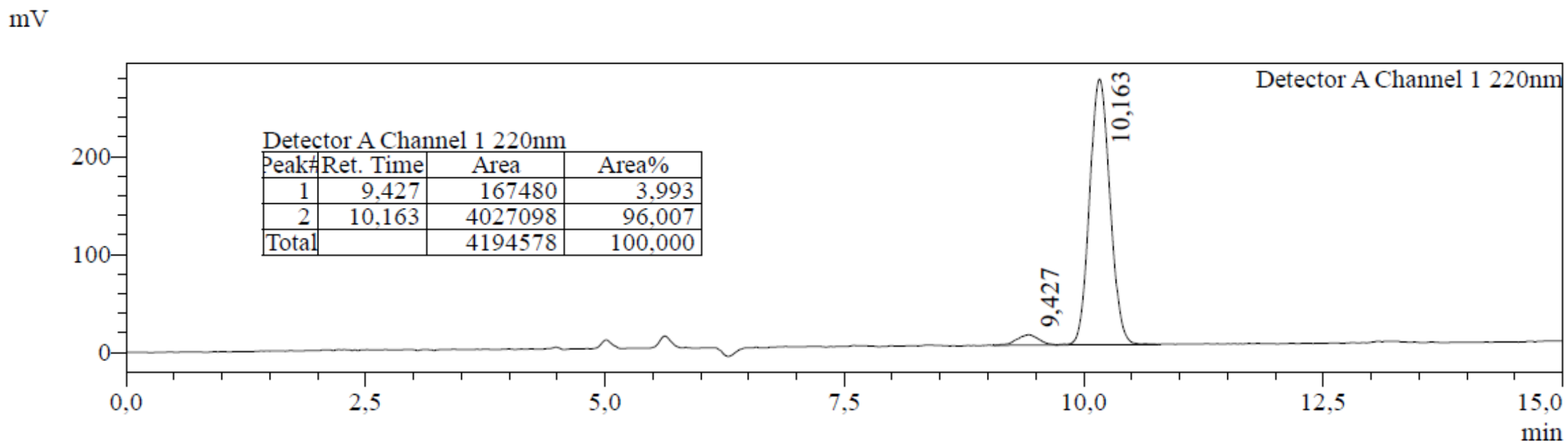

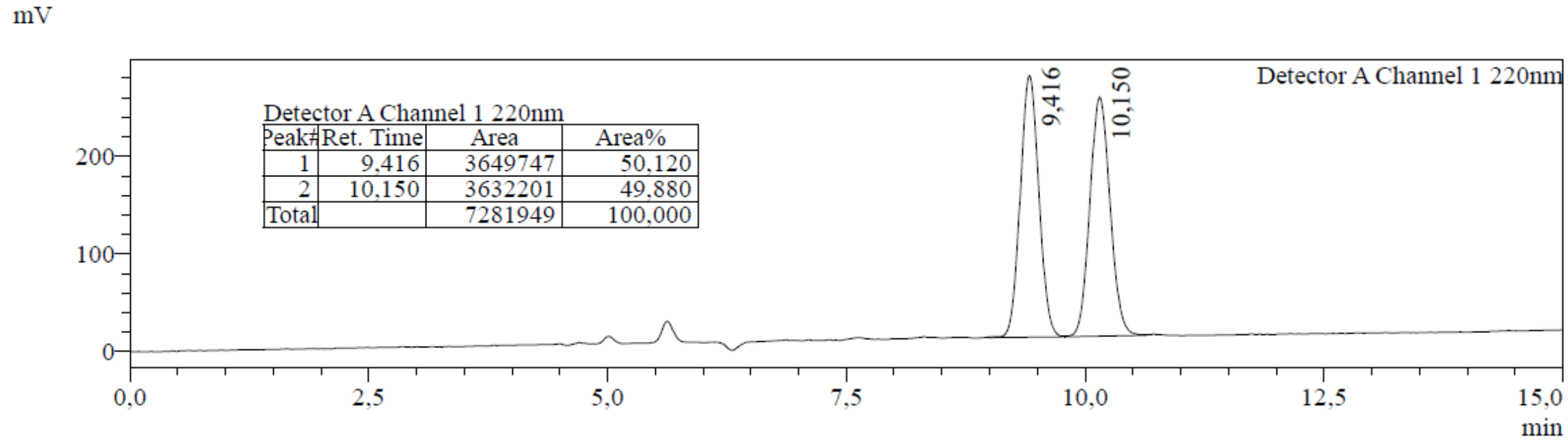

***tert*-Butyl 2-((3,4-dimethoxyphenethyl)(methyl)amino)-2-phenylacetate (2x)**

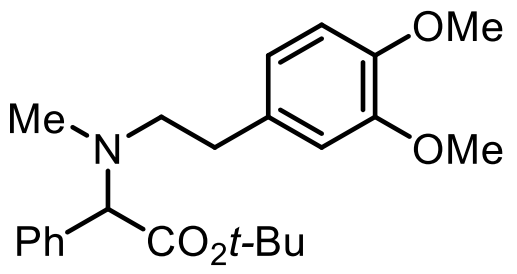

General procedure **GP1** was followed using sulfonamide **1a** (80.9 mg, 0.150 mmol, 1.0 equiv.) and **S10** (39.5 mg, 0.180 mmol, 1.2 equiv.) for 72 h without lithium iodide. The resulting crude material was subjected to flash column chromatography (silica gel, 10%-30% EtOAc in n-heptane) to obtain the title compound as a colorless oil (29.8 mg, 0.077 mmol, 52% yield).

^{1}H NMR (400 MHz, $CDCl_3$): δ 7.40 – 7.35 (m, 2H), 7.34 – 7.25 (m, 3H), 6.75 (d, *J* = 8.1 Hz, 1H), 6.67 – 6.58 (m, 2H), 4.15 (s, 1H), 3.84 (s, 3H), 3.81 (s, 3H), 2.77 – 2.64 (m, 4H), 2.37 (s, 3H), 1.42 (s, 9H) ppm.

^{13}C NMR (101 MHz, $CDCl_3$): δ 171.3, 148.9, 147.4, 137.3, 133.2, 128.9 (2C), 128.4 (2C), 127.9, 120.7, 112.2, 111.3, 81.3, 73.4, 56.7, 56.0, 55.9, 39.4, 33.3, 28.1 (3C) ppm.

IR (neat) ν_{max}: 2934, 2834, 1728, 1515, 1260, 1140, 1029, 728, 698 cm^{-1}.

HRMS (ESI$^+$): exact mass calculated for $[M+H]^+$ $(C_{23}H_{32}NO_4)^+$ requires *m/z* 386.2326, found *m/z* 386.2324.

Rf: 0.27 *n*-heptane:EtOAc 5:1

(*Z*)-*N*^{2},*N*^{2}-Dibutyl-1-(2,7-dichloro-9-(4-chlorobenzylidene)-9*H*-fluoren-4-yl)-*N*^{1}-(3,4-dimethoxyphenethyl)-*N*^{1}-methylethane-1,2-diamine (2y)

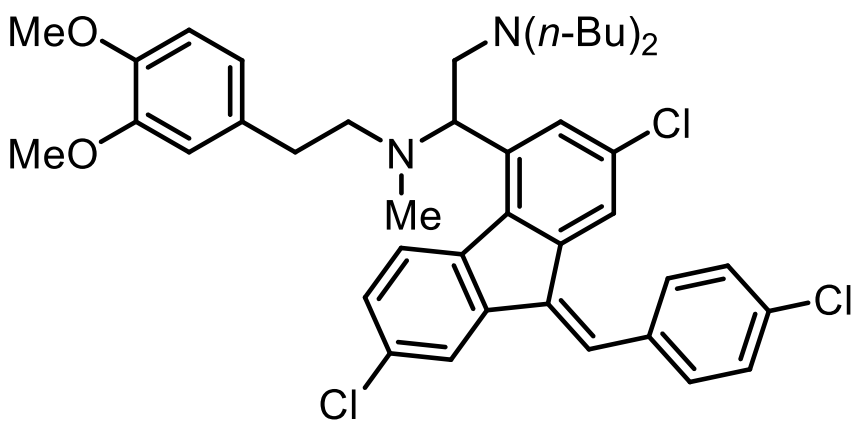

General procedure **GP2** was followed, using sulfonamide **1a** (80.9 mg, 0.15 mmol, 1.00 equiv.) and lumefantrine (97.2 mg, 0.18 mmol, 1.20 equiv.). The resulting crude material was subjected to flash column chromatography (silica gel, 0%-20% EtOAc in *n*-heptane) to obtain the title compound as a yellow oil (55.6 mg, 0.079 mmol, 53% yield).

^{1}H NMR (600 MHz, Acetone-*d$_6$*): δ 7.94 (d, *J* = 6.3 Hz, 3H), 7.65 (d, *J* = 7.3 Hz, 2H), 7.59 (d, *J* = 8.4 Hz, 2H), 7.39 (br s, 3H), 6.78 – 6.39 (m, 3H), 3.71 (s, 3H), 3.64 (s, 3H), 2.95 (br s, 2H), 2.89 – 2.11 (m, 12H), 1.28 – 0.98 (m, 8H), 0.72 (t, *J* = 6.3 Hz, 6H) ppm.

^{13}C NMR (151 MHz, Acetone-*d$_6$*): δ 150.0, 148.5, 142.6, 141.0, 139.4, 138.8, 138.0, 136.0, 135.9, 134.8, 133.9, 133.2, 132.1, 131.8 (2C), 129.8 (2C), 128.9, 128.8, 126.8, 123.0, 121.6, 121.1, 113.2, 112.4, 73.5, 62.5, 56.4, 56.0, 55.8, 55.2 (2C), 54.9, 39.4, 34.1, 21.1 (2C), 14.4 (2C) ppm. *(Two carbon peaks could not be clearly identified due to strong broadening)*

IR (neat) ν_{max}: 2954, 2858, 1514, 1441, 1260, 1235, 1085, 1030, 732 cm^{-1}.

HRMS (ESI$^+$): exact mass calculated for $[M+H]^+$ $(C_{41}H_{48}Cl_3N_2O_2)^+$ requires *m/z* 705.2776, found *m/z* 705.2777.

Rf: 0.16 *n*-heptane:EtOAc 10:1.

***N*-(4-Bromobenzyl)-2-phenylethan-1-amine (3a)**

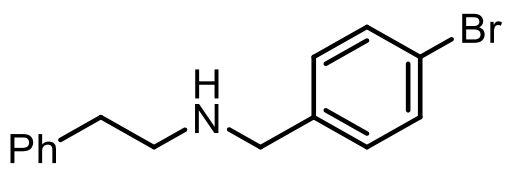

General procedure **GP3** was followed, using sulfonamide **S1**[34] (69.8 mg, 0.15 mmol, 1.00 equiv.) and 4-bromobenzyl alcohol (56.1 mg, 0.30 mmol, 2.00 equiv.). The resulting crude material was subjected to flash column chromatography (silica gel, 20%-50% EtOAc in *n*-heptane) to obtain the title compound as a colourless oil (33.2 mg, 0.114 mmol, 76% yield).

^{1}H NMR (400 MHz, $CDCl_3$): δ 7.45 – 7.40 (m, 2H), 7.33 – 7.27 (m, 2H), 7.24 – 7.13 (m, 5H), 3.76 (s, 2H), 2.92 – 2.86 (m, 2H), 2.86 – 2.79 (m, 2H), 1.89 (br s, 1H) ppm.

^{13}C NMR (101 MHz, $CDCl_3$): δ 139.9, 139.1, 131.6 (2C), 129.9 (2C), 128.8 (2C), 128.7 (2C), 126.4, 120.9, 53.1, 50.4, 36.3 ppm.

IR (neat) ν_{max}: 3026, 2920, 2823, 1486, 1453, 1269, 1111, 1010, 697 cm^{-1}.

HRMS (ESI$^+$): exact mass calculated for $[M+H]^+$ $(C_{15}H_{17}{}^{79}BrN)^+$ requires *m/z* 290.0539, found *m/z* 290.0536.

Rf: 0.33 *n*-heptane:EtOAc 2:1.

***tert*-Butyl (4-((4-bromobenzyl)amino)butyl)carbamate (3b)**

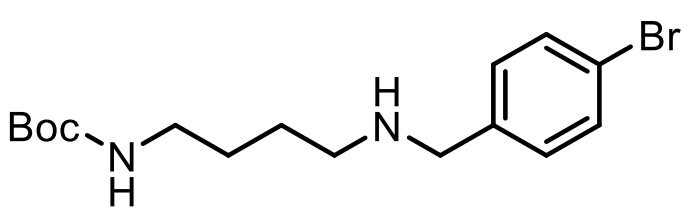

General procedure **GP3** was followed, using sulfonamide **S16** (79.9 mg, 0.15 mmol, 1.00 equiv.) and 4-bromobenzyl alcohol (56.1 mg, 0.30 mmol, 2.00 equiv.). The resulting crude material was subjected to flash column chromatography (silica gel, 0%-50% EtOAc:EtOH:NH_4OH (3:1:2%) in *n*-heptane) to obtain the title compound as a yellow solid (37.1 mg, 0.104 mmol, 69% yield).

^{1}H NMR (700 MHz, $CDCl_3$): δ 7.50 – 7.42 (m, 2H), 7.32 – 7.24 (m, 2H), 4.82 (s, 1H), 3.82 (s, 2H), 3.15 – 3.05 (m, 2H), 2.70 (t, *J* = 7.0 Hz, 2H), 1.66 – 1.57 (m, 2H), 1.56 – 1.48 (m, 2H), 1.42 (s, 9H), 1.25 (s, 1H) ppm.

^{13}C NMR (176 MHz, $CDCl_3$): δ 156.3, 136.1 (br s), 131.9 (2C), 130.7 (2C), 122.0, 79.4, 52.4, 48.1, 40.2, 28.6 (3C), 27.8, 26.0 ppm.

IR (neat) ν_{max}: 1693, 1516, 1428, 1367, 127, 1193, 1084, 916, 864, 738, 707, 686, 669, 619, 569 cm^{-1}.

HRMS (ESI+): exact mass calculated for $[M+H]^+$ $(C_{16}H_{26}{}^{79}BrN_2O_2)^+$ requires *m/z* 357.1172, found *m/z* 357.1160.

R_f: 0.21 *n*-heptane:(EtOAc:EtOH:NH_4OH (3:1:2%)) 1:1.

***N*-(4-Bromobenzyl)-1-(thiophen-2-yl)methanamine (3c)**

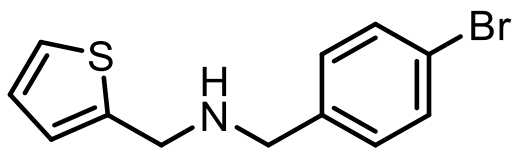

General procedure **GP3** was followed, using *N*-(thiophen-2-ylmethyl)-2,4,6-tris(trifluoromethyl)benzenesulfonamide[33] (68.6 mg, 0.15 mmol, 1.00 equiv.) and 4-bromobenzyl alcohol (56.1 mg, 0.30 mmol, 2.00 equiv.). The resulting crude material was subjected to flash column chromatography (silica gel, 50% EtOAc:EtOH:NH_4OH (3:1:2) in *n*-heptane) to obtain the title compound as a yellow oil (34.4 mg, 0.122 mmol, 81% yield).

^{1}H NMR (700 MHz, $CDCl_3$): δ 7.46 – 7.44 (m, 2H), 7.24 – 7.21 (m, 3H), 6.96 (dd, *J* = 5.0, 3.4 Hz, 1H), 6.93 – 6.91 (m, 1H), 3.98 (d, *J* = 0.9 Hz, 2H), 3.79 (s, 2H), 1.64 (s, 1H) ppm.

^{13}C NMR (176 MHz, $CDCl_3$): δ 144.1, 139.2, 131.6 (2C), 130.1 (2C), 126.8, 125.1, 124.6, 120.9, 52.2, 47.6 ppm.

IR (neat) ν_{max}: 1798, 1486, 1368, 1216, 1070, 1011, 799, 778 cm^{-1}.

HRMS (ESI+): exact mass calculated for $[M+H]^+$ $(C_{12}H_{13}{}^{81}BrN^{32}S)^+$ requires *m/z* 281.9947, found *m/z* 281.9943.

R_f: 0.73 *n*-heptane:(EtOAc:EtOH:NH4OH (3:1:2%)) 1:1.

***rac*-(1*R*,2*S*,5*R*)-*N*-(4-Bromobenzyl)-2-isopropyl-5-methylcyclohexan-1-amine (3d)**

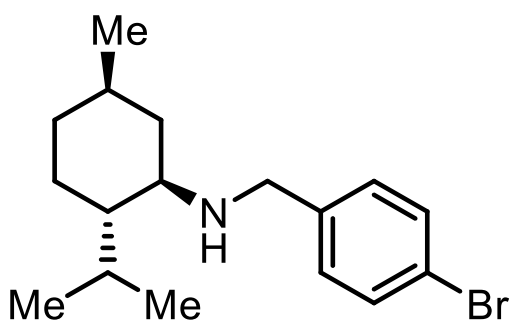

General procedure **GP3** was followed, using sulfonamide *rac*-*N*-((1*R*,2*S*,5*R*)-2-isopropyl-5-methylcyclohexyl)-2,4,6-tris(trifluoromethyl)benzenesulfonamide[33] (74.9 mg, 0.15 mmol, 1.00 equiv.) and 4-bromobenzyl alcohol (56.1 mg, 0.30 mmol, 2.00 equiv.). The resulting crude material was subjected to flash column chromatography (silica gel, 10%-50% EtOAc in *n*-heptane) to obtain the title compound as a colourless oil (32.6 mg, 0.101 mmol, 67% yield).

^{1}H NMR (400 MHz, $CDCl_3$): δ 7.43 (d, *J* = 8.3 Hz, 2H), 7.21 (d, *J* = 8.3 Hz, 2H), 3.85 (d, *J* = 13.2 Hz, 1H), 3.62 (d, *J* = 13.2 Hz, 1H), 2.30 (td, *J* = 10.6, 3.8 Hz, 1H), 2.20 (pd, *J* = 6.9, 2.8 Hz, 1H), 2.09 (dq, *J* = 12.3, 3.5 Hz, 1H), 1.68 (dt, *J* = 12.4, 2.6 Hz, 1H), 1.62 (dq, *J* = 12.2, 2.9 Hz, 1H), 1.40 – 1.24 (m, 2H), 1.13 – 1.04 (m, 1H), 0.98 (td, *J* = 12.5, 3.1 Hz, 1H), 0.91 (d, *J* = 2.6 Hz, 3H), 0.90 (d, *J* = 3.0 Hz, 3H), 0.85 (dd, *J* = 12.0, 3.2 Hz, 1H), 0.79 – 0.70 (m, 1H), 0.68 (d, *J* = 6.9 Hz, 3H) ppm.

^{13}C NMR (101 MHz, $CDCl_3$): δ 140.5, 131.5 (2C), 130.1 (2C), 120.6, 57.4, 50.3, 48.1, 42.0, 35.0, 32.1, 25.9, 23.7, 22.7, 21.5, 15.8 ppm.

IR (neat) ν_{max}: 2951, 2917, 2867, 1486, 1454, 1070, 1011, 803, 695 cm^{-1}.

HRMS (ESI$^+$): exact mass calculated for $[M+H]^+$ $(C_{17}H_{27}{}^{79}BrN)^+$ requires *m/z* 324.1321, found *m/z* 324.1320.

Rf: 0.38 *n*-heptane:EtOAc 5:1.

(*R*)-3-((4-Bromobenzyl)amino)-1-(3-(trifluoromethyl)-5,6-dihydro-[1,2,4]triazolo[4,3-*a*]pyrazin-7(8*H*)-yl)-4-(2,4,5-trifluorophenyl)butan-1-one (3e)

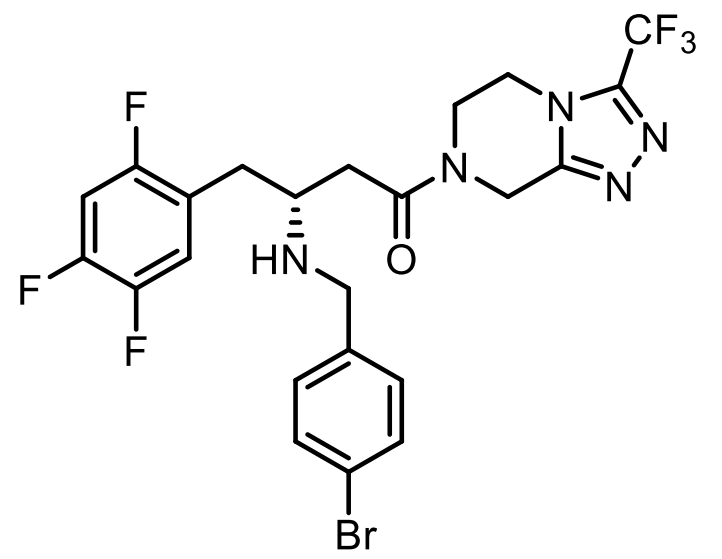

General procedure **GP3** was followed, using sulfonamide **S17** (113.0 mg, 0.15 mmol, 1.00 equiv.) and 4-bromobenzyl alcohol (56.1 mg, 0.30 mmol, 2.00 equiv.). The resulting crude material was subjected to flash column chromatography (silica gel, 100% EtOAc) to obtain the title compound as a yellow oil (45.9 mg, 0.080 mmol, 53% yield). The desired product was obtained as a mixture of two rotamers in a ratio of 2:1.

^{1}H NMR (400 MHz, $CDCl_3$): δ 7.39 (d, *J* = 8.2 Hz, 2H), 7.10 (d, *J* = 8.1 Hz, 2H), 7.02 (q, *J* = 8.9 Hz, 1H), 6.88 (q, *J* = 9.3 Hz, 1H), 5.09 – 4.77 (m, 2H), 4.16 – 3.97 (m, 3.3H), 3.86 – 3.69 (m, 2.7H), 3.37 – 3.26 (m, 1H), 2.95 – 2.86 (m, 1H), 2.79 – 2.70 (m, 1H), 2.55 – 2.41 (m, 2H), 1.88 (br s, 1H) ppm.

Major Isomer: **^{13}C NMR (101 MHz, $CDCl_3$):** δ 170.6, 156.2 (ddd, $^1J_{C-F}$ = 243.2, $^3J_{C-F}$ = 9.1, $^4J_{C-F}$ = 2.5 Hz), 149.6, 149.0 (ddd, $^1J_{C-F}$ = 250.7, $^3J_{C-F}$ = 14.1, $^3J_{C-F}$ = 12.6 Hz), 146.8 (ddd, $^1J_{C-F}$ = 245.2, $^3J_{C-F}$ = 12.5, $^4J_{C-F}$ = 3.5 Hz), 144.0 (q, $^2J_{C-F}$ = 39.0 Hz), 139.0, 131.7 (2C), 129.8 (2C), 121.8 (dd, $^2J_{C-F}$ = 20.1, $^3J_{C-F}$ = 6.4 Hz), 121.1, 119.2 (dd, $^2J_{C-F}$ = 19.5, $^3J_{C-F}$ = 6.1 Hz), 118.3 (q, $^1J_{C-F}$ = 270.2 Hz), 105.7 (dd, $^2J_{C-F}$ = 28.9, $^2J_{C-F}$ = 20.7 Hz), 54.8, 50.9, 43.3, 42.7, 38.2, 37.3, 32.9 ppm.

^{19}F NMR (376 MHz, $CDCl_3$): δ -63.05 (s, 3F), -119.05 – -119.28 (m, 1F), -135.16 – -135.50 (m, 1F), -142.23 – -142.63 (m, 1F) ppm.

IR (neat) ν_{max}: 2934, 1650, 1516, 1422, 1209, 1137, 1012, 728 cm^{-1}.

HRMS (ESI$^+$): exact mass calculated for $[M+H]^+$ $(C_{23}H_{21}{}^{79}BrF_6N_5O_1)^+$ requires *m/z* 576.0828, found *m/z* 576.0833.

Rf: 0.21 EtOAc 100%.

$[\alpha]_D^{19}$ = -4.6 (*c* = 0.87, $CHCl_3$).

(*R*)-2-((6-(3-((4-Bromobenzyl)amino)piperidin-1-yl)-3-methyl-2,4-dioxo-3,4-dihydropyrimidin-1(2*H*)-yl)methyl)benzonitrile (3f)

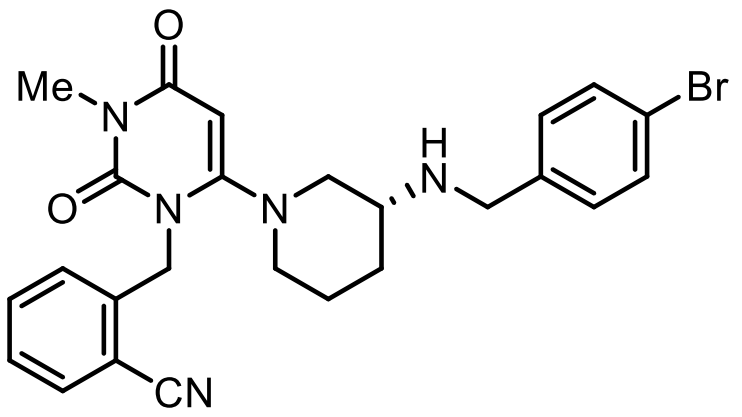

General procedure **GP3** was followed, using sulfonamide **S18** (103.0 mg, 0.15 mmol, 1.00 equiv.) and 4-bromobenzyl alcohol (56.1 mg, 0.30 mmol, 2.00 equiv.). The resulting crude material was subjected to flash column chromatography (silica gel, 10%-20% EtOH in EtOAc with 1% NH_4OH (25% in H_2O)) to obtain the title compound as a colourless oil (55.9 mg, 0.110 mmol, 73% yield).

^{1}H NMR (400 MHz, $CDCl_3$): δ 7.61 (d, *J* = 7.6 Hz, 1H), 7.53 (td, *J* = 7.8, 1.1 Hz, 1H), 7.40 (d, *J* = 8.3 Hz, 2H), 7.33 (t, *J* = 7.5 Hz, 1H), 7.12 (d, *J* = 7.9 Hz, 1H), 7.08 (d, *J* = 8.3 Hz, 2H), 5.39 – 5.18 (m, 3H), 3.51 (dd, *J* = 13.3 Hz, 2H), 3.31 (s, 3H), 3.09 (d, *J* = 10.6 Hz, 1H), 2.93 (d, *J* = 11.0 Hz, 1H), 2.68 (dt, *J* = 8.8, 4.9 Hz, 2H), 2.41 (br s, 1H), 1.90 (br s, 1H), 1.81 – 1.72 (m, 1H), 1.67 – 1.54 (m, 1H), 1.30 (br s, 2H) ppm.

^{13}C NMR (101 MHz, $CDCl_3$): δ 163.2, 159.9, 152.8, 140.9, 139.2, 133.3, 133.2, 131.6 (2C), 129.7 (2C), 127.9, 126.6, 120.9, 117.1, 110.8, 90.6, 57.2, 53.2, 52.1, 50.4, 46.5, 30.6, 28.1, 23.2 ppm.

IR (neat) ν_{max}: 2940, 2834, 2224, 1699, 1645, 1603, 1434, 1216, 726 cm^{-1}.

HRMS (ESI$^+$): exact mass calculated for $[M+H]^+$ $(C_{25}H_{27}{}^{79}BrN_5O_2)^+$ requires *m/z* 508.1343, found *m/z* 508.1333.

Rf: 0.29 EtOAc:MeOH 10:1.

$[\alpha]_D^{19}$ = 17.4 (*c* = 0.89, $CHCl_3$).

***N*^{1}-(4-Bromobenzyl)-*N*^{4}-(6-methoxyquinolin-8-yl)pentane-1,4-diamine (3g)**

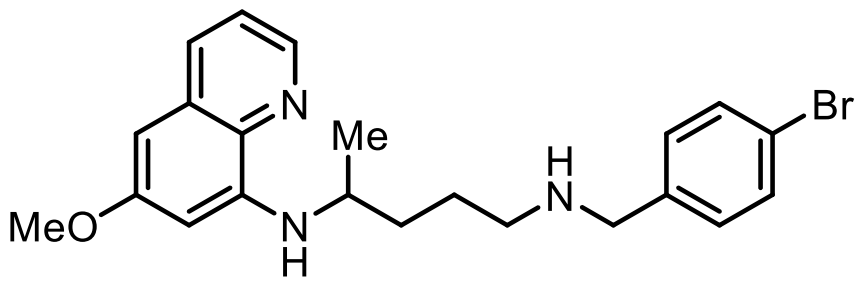

General procedure **GP3** was followed, using sulfonamide **S19** (90.5 mg, 0.15 mmol, 1.00 equiv.) and 4-bromobenzyl alcohol (56.1 mg, 0.30 mmol, 2.00 equiv.). The resulting crude material was subjected to flash column chromatography (silica gel, 10%-20% EtOH in EtOAc with 1% NH_4OH (25% in H_2O)) to obtain the title compound as a brown oil (48.9 mg, 0.114 mmol, 76% yield).

^{1}H NMR (400 MHz, $CDCl_3$): δ 8.52 (dd, *J* = 4.2, 1.6 Hz, 1H), 7.91 (dd, *J* = 8.3, 1.6 Hz, 1H), 7.43 – 7.36 (m, 2H), 7.29 (dd, *J* = 8.2, 4.2 Hz, 1H), 7.16 (d, *J* = 8.4 Hz, 2H), 6.31 (dd, *J* = 19.8, 2.5 Hz, 2H), 6.04 (br s, 1H), 3.89 (s, 3H), 3.72 (s, 2H), 3.61 (br s, 1H), 2.65 (t, *J* = 6.8 Hz, 2H), 2.16 (br s, 1H), 1.79 – 1.59 (m, 4H), 1.30 (d, *J* = 6.4 Hz, 3H) ppm.

^{13}C NMR (101 MHz, $CDCl_3$): δ 159.5, 145.1, 144.4, 139.1, 135.5, 134.9, 131.5 (2C), 130.0 (3C), 122.0, 120.9, 96.9, 91.7, 55.3, 53.2, 49.3, 48.1, 34.5, 26.6, 20.7 ppm.

IR (neat) ν_{max}: 2927, 2855, 1614, 1517, 1454, 1356, 1158, 818, 789 cm^{-1}.

HRMS (ESI$^+$): exact mass calculated for $[M+H]^+$ $(C_{22}H_{27}{}^{79}BrN_3O)^+$ requires *m/z* 428.1332, found *m/z* 428.1330.

Rf: 0.34 EtOAc:MeOH 10:1.

***N*-(4-Bromobenzyl)-4-methoxy-*N*-methylaniline (3h)**

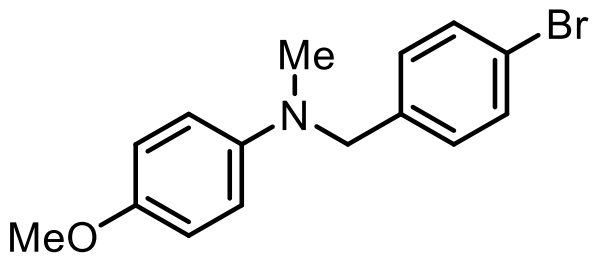

General procedure **GP3** was followed, using **S20** (72.2 mg, 0.15 mmol, 1.00 equiv.), and 4-bromobenzyl alcohol (33.7 mg, 0.18 mmol, 1.20 equiv.). The resulting crude material was subjected to flash column chromatography (silica gel, 0%-10% EtOAc in *n*-heptane) to obtain the title compound as a dark brown oil (38.6 mg, 0.126 mmol, 84% yield).

^{1}H NMR (400 MHz, CDCl$_3$): δ 7.42 (d, *J* = 8.3 Hz, 2H), 7.12 (d, *J* = 8.3 Hz, 2H), 6.86 – 6.79 (m, 2H), 6.75 – 6.68 (m, 2H), 4.36 (s, 2H), 3.76 (s, 3H), 2.90 (s, 3H) ppm.

^{13}C NMR (101 MHz, CDCl$_3$): δ 152.1, 144.7, 138.4, 131.7 (2C), 129.0 (2C), 120.7, 114.91 (2C), 114.86 (2C), 57.7, 55.9, 39.3 ppm.

IR (neat) ν_{max}: 2931, 2830, 1510, 1485, 1452, 1239, 1180, 1123, 1069, 1038, 1010, 810, 524 cm^{-1}.

HRMS: exact mass calculated for $[M+H]^+$ $(C_{15}H_{17}{}^{79}BrNO)^+$ requires *m/z* 306.0488, found *m/z* 306.0489.

R_f: 0.82 *n*-heptane:EtOAc 1:1.

***tert*-Butyl 4-(4-bromobenzyl)piperazine-1-carboxylate (3i)**

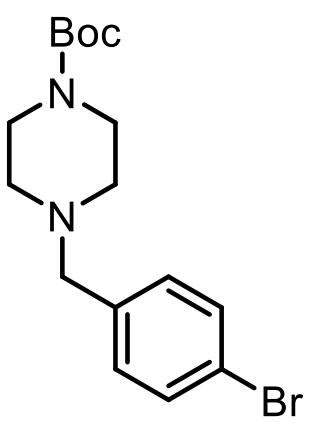

General procedure **GP3** was followed, using sulfonamide **7** (79.6 mg, 0.15 mmol, 1.00 equiv.) and 4-bromobenzyl alcohol (33.7 mg, 0.18 mmol, 1.20 equiv.). The resulting crude material was subjected to flash column chromatography (silica gel, 0%-50% Et_2O in *n*-heptane) to obtain the title compound as an orange solid (40.2 mg, 0.113 mmol, 75% yield).

^{1}H NMR (700 MHz, CDCl$_3$): δ 7.44 (d, *J* = 8.4 Hz, 2H), 7.20 (d, *J* = 8.4 Hz, 2H), 3.45 (s, 2H), 3.44 – 3.39 (m, 4H), 2.38 – 2.33 (m, 4H), 1.45 (s, 9H) ppm.

^{13}C NMR (176 MHz, CDCl$_3$): δ 154.9, 137.2, 131.5 (2C), 130.9 (2C), 121.1, 79.8, 62.4, 53.0 (2C), 44.3 – 43.6 (br m, 2C), 28.6 (3C) ppm.

IR (neat) ν_{max}: 2974, 2931, 2814, 1693, 1486, 1456, 1419, 1365, 1245, 1170, 1124, 1070, 1006, 790 cm^{-1}.

HRMS (ESI⁺): exact mass calculated for [M+H]⁺ ($C_{16}H_{24}{}^{79}BrN_2O_2$)⁺ requires *m/z* 355.1016, found *m/z* 355.1010.

R$_f$: 0.45 *n*-heptane:EtOAc 1:1.

1-(4-Bromobenzyl)-4-phenylpiperidin-4-ol (3j)

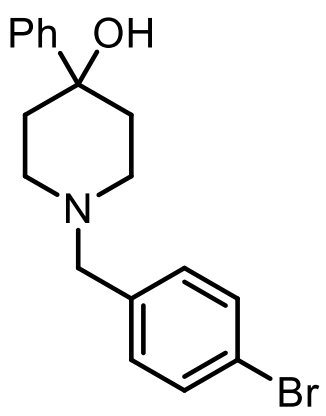

A modified general procedure **GP3** was followed, using lithium *tert*-butoxide (30.0 mg, 0.375 mmol, 2.50 equiv.), sulfonamide **S21** (78.2 mg, 0.15 mmol, 1.00 equiv.) and 4-bromobenzyl alcohol (33.7 mg, 0.18 mmol, 1.20 equiv.). The resulting crude material was subjected to flash column chromatography (silica gel, 0%-50% EtOAc in *n*-heptane) to obtain the title compound as a yellow solid (43.2 mg, 0.125 mmol, 83% yield).

^{1}H NMR (600 MHz, CDCl$_3$): δ 7.51 (d, *J* = 7.4 Hz, 2H), 7.46 (d, *J* = 8.3 Hz, 2H), 7.36 (t, *J* = 7.7 Hz, 2H), 7.29 – 7.24 (m, 3H), 3.57 (s, 2H), 2.78 (br s, 2H), 2.53 (br s, 2H), 2.20 (br s, 2H), 1.76 (d, *J* = 12.2 Hz, 2H), 1.63 (br s, 1H) ppm.

^{13}C NMR (151 MHz, CDCl$_3$): δ 148.3, 137.3, 131.5 (2C), 131.1 (2C), 128.5 (2C), 127.2, 124.7 (2C), 121.2, 62.5 (2C), 49.5 (2C), 38.4 (2C) ppm.

IR (neat) ν$_{max}$: 2920, 2818, 1738, 1487, 1366, 1130, 1071, 1044, 1012, 789, 760, 699 cm^{-1}.

HRMS (ESI⁺): exact mass calculated for [M+H]⁺ ($C_{18}H_{21}{}^{79}BrNO$)⁺ requires *m/z* 346.0801, found *m/z* 346.0793.

R$_f$: 0.18 *n*-heptane:EtOAc 1:1.

(3*S*,4*R*)-3-((Benzo[*d*][1,3]dioxol-5-yloxy)methyl)-1-(4-bromobenzyl)-4-(4-fluorophenyl)piperidine (3k)

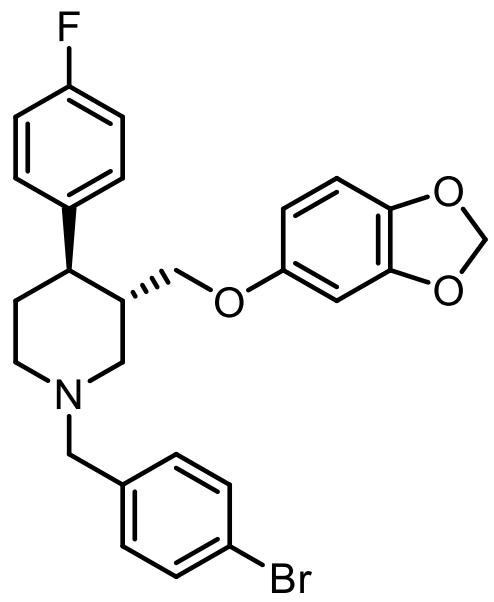

General procedure **GP3** was followed, using (3*S*,4*R*)-3-((benzo[*d*][1,3]dioxol-5-yloxy)methyl)-4-(4-fluorophenyl)-1-((2,4,6-tris(trifluoromethyl)phenyl)sulfonyl)piperidine[33] (101 mg, 0.15 mmol, 1.00 equiv.) and 4-bromobenzyl alcohol (33.7 mg, 0.18 mmol, 1.20 equiv.). The resulting crude material was subjected to flash column chromatography (silica gel, 0%-40% EtOAc in *n*-heptane) to obtain the title compound as a yellow oil (50.0 mg, 0.10 mmol, 67% yield).

^{1}H NMR (400 MHz, CDCl$_3$): δ 7.48 – 7.37 (m, 2H), 7.24 (d, *J* = 8.3 Hz, 2H), 7.19 – 7.11 (m, 2H), 7.02 – 6.92 (m, 2H), 6.61 (d, *J* = 8.5 Hz, 1H), 6.32 (d, *J* = 2.5 Hz, 1H), 6.10 (dd, *J* = 8.5, 2.5 Hz, 1H), 5.88 (s, 2H), 3.62 – 3.36 (m, 4H), 3.23

– 3.17 (m, 1H), 3.04 – 2.88 (m, 1H), 2.47 (td, J = 11.3, 4.9 Hz, 1H), 2.25 – 2.14 (m, 1H), 2.12 – 2.01 (m, 2H), 1.91 – 1.73 (m, 2H) ppm.

^{13}C NMR (176 MHz, CDCl$_3$): δ 161.7 (d, $^1J_{C-F}$ = 243.2 Hz), 154.5, 148.3, 141.7, 139.7, 137.3 (br s), 131.5 (2C), 131.0 (2C), 129.0 (d, $^3J_{C-F}$ = 7.8 Hz, 2C), 121.1, 115.6 (d, $^2J_{C-F}$ = 21.1 Hz, 2C), 108.0, 105.7, 101.2, 98.1, 69.6, 62.7, 57.6, 53.9, 44.1, 42.2, 34.3 ppm.

^{19}F NMR (659MHz, CDCl$_3$): δ -116.54 (s, 1F) ppm.

IR (neat) ν_{max}: 2917, 1635, 1605, 1509, 1487, 1468, 1391, 1343, 1269, 1223, 1184, 1159, 1134, 1096, 1070, 1039, 1012, 937, 831, 794 cm^{-1}.

HRMS (ESI$^+$): exact mass calculated for $[M+H]^+$ $(C_{26}H_{26}{}^{79}BrFNO_3)^+$ requires *m/z* 498.1069, found *m/z* 498.1069.

R$_f$: 0.18 *n*-heptane:EtOAc 1:1.

$[\alpha]_D^{23}$ = 7.2 (c = 0.34, CH_2Cl_2).

5-((4-(4-Bromobenzyl)-1,4-diazepan-1-yl)sulfonyl)isoquinoline (3l)

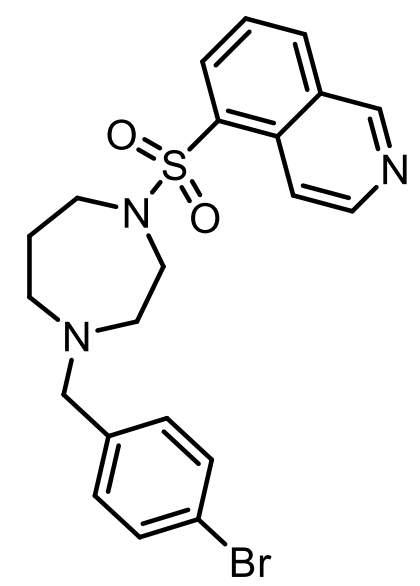

General procedure **GP3** was followed, using sulfonamide **S22** (95.3 mg, 0.15 mmol, 1.00 equiv.) and 4-bromobenzyl alcohol (33.7 mg, 0.18 mmol, 1.20 equiv.). The resulting crude material was subjected to flash column chromatography (silica gel, 100% EtOAc) to obtain the title compound as a white solid (37.9 mg, 0.082 mmol, 55% yield).

^{1}H NMR (400 MHz, CDCl$_3$): δ 9.35 (s, 1H), 8.68 (d, J = 6.1 Hz, 1H), 8.45 (d, J = 6.1 Hz, 1H), 8.34 (dd, J = 7.4, 1.1 Hz, 1H), 8.19 (d, J = 8.2 Hz, 1H), 7.68 (t, J = 7.9 Hz, 1H), 7.40 (d, J = 8.3 Hz, 2H), 7.13 (d, J = 8.3 Hz, 2H), 3.55 (s, 2H), 3.52 – 3.44 (m, 4H), 2.70 – 2.61 (m, 4H), 1.83 (p, J = 6.0 Hz, 2H) ppm.

^{13}C NMR (101 MHz, CDCl$_3$): δ 153.4, 145.2, 138.1, 134.7, 133.5, 133.2, 131.8, 131.6 (2C), 130.5 (2C), 129.4, 126.0, 121.0, 117.8, 61.5, 56.1, 54.5, 48.4, 46.9, 28.6 ppm.

IR (neat) ν_{max}: 2923, 2854, 1486, 1322, 1155, 1135, 1069, 825, 594 cm^{-1}.

HRMS (ESI$^+$): exact mass calculated for $[M+H]^+$ $(C_{21}H_{23}{}^{79}BrN_3O_2{}^{32}S)^+$ requires *m/z* 460.0689, found *m/z* 460.0695.

Rf: 0.25 EtOAc 100%.

11-(1-(4-Bromobenzyl)piperidin-4-ylidene)-8-chloro-6,11-dihydro-5*H*-benzo[5,6]cyclohepta[1,2-*b*]pyridine (3m)

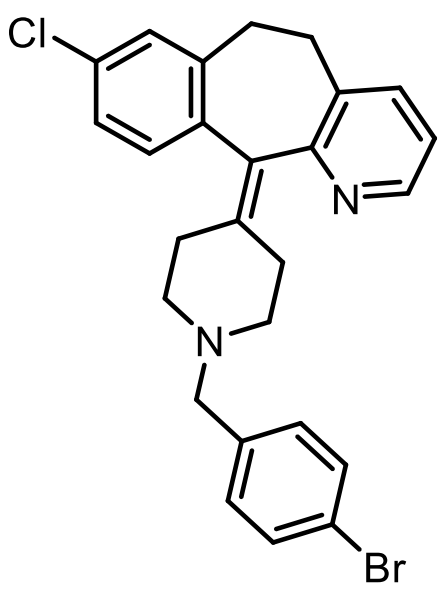

General procedure **GP3** was followed, using 8-chloro-11-(1-((2,4,6-tris(trifluoromethyl)phenyl)sulfonyl)piperidin-4-ylidene)-6,11-dihydro-5*H*-benzo[5,6]cyclohepta[1,2-*b*]pyridine[33] (110 mg, 0.15 mmol, 1.00 equiv.), and 4-bromobenzyl alcohol (33.7 mg, 0.18 mmol, 1.20 equiv.). The resulting crude material was subjected to flash column chromatography (silica gel, 100% EtOAc:EtOH:NH_4OH (3:1:2%)) to obtain the title compound as an orange oil (45.0 mg, 0.095 mmol, 63% yield).

^{1}H NMR (600 MHz, $CDCl_3$): δ 8.38 (dd, *J* = 4.8, 1.7 Hz, 1H), 7.44 – 7.31 (m, 3H), 7.18 (dt, *J* = 8.4, 1.7 Hz, 2H), 7.16 – 7.10 (m, 3H), 7.07 (dd, *J* = 7.6, 4.8 Hz, 1H), 3.44 (s, 2H), 3.43 – 3.29 (m, 2H), 2.88 – 2.75 (m, 2H), 2.75 – 2.66 (m, 2H), 2.56 – 2.47 (m, 1H), 2.45 – 2.37 (m, 1H), 2.37 – 2.26 (m, 2H), 2.20 – 2.07 (m, 2H) ppm.

^{13}C NMR (151 MHz, $CDCl_3$): δ 158.0, 147.0, 139.8, 139.2, 138.2, 137.8, 137.6, 133.7, 133.04, 132.98, 131.6 (2C), 131.2, 131.1, 129.3, 126.3, 122.4, 121.1, 62.4 (2C), 55.1, 55.0, 32.2, 31.8, 31.3, 31.1 ppm.

IR (neat) ν_{max}: 2899, 2797, 1483, 1403, 1069, 1011, 989, 826, 788 cm^{-1}.

HRMS (ESI$^+$): exact mass calculated for $[M+H]^+$ $(C_{26}H_{25}{}^{79}Br^{35}ClN_2)^+$ requires *m/z* 479.0884, found *m/z* 479.0882.

R_f: 0.10 *n*-heptane:EtOAc 1:1.

***N*-(4-Bromobenzyl)-*N*-methyl-1-(5-phenyl-1-(pyridin-3-ylsulfonyl)-1*H*-pyrrol-3-yl)methanamine (3n)**

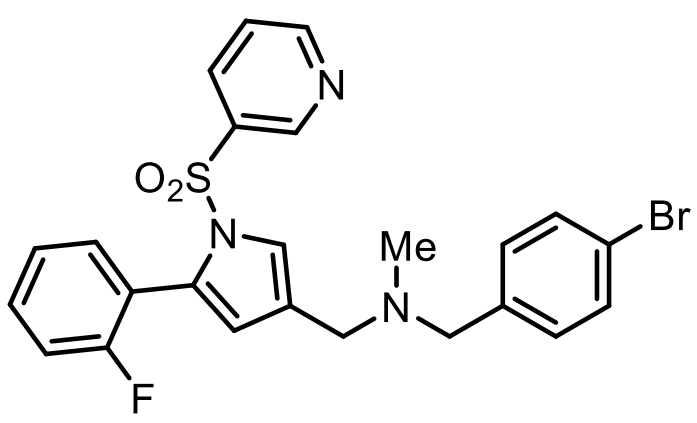

General procedure **GP3** was followed, using sulfonamide **S23** (103.0 mg, 0.15 mmol, 1.00 equiv.), and 4-bromobenzyl alcohol (33.7 mg, 0.18 mmol, 1.20 equiv.). The resulting crude material was subjected to flash column chromatography (silica gel, 100% EtOAc:EtOH:NH_4OH (3:1:2%) followed by 100% EtOAc:*n*-heptane:NH_4OH (1:1:2%)) to obtain the title compound as a yellow oil (53.6 mg, 0.105 mmol, 70% yield).

^{1}H NMR (400 MHz, $CDCl_3$): δ 8.73 (dd, *J* = 8.2, 4.9 Hz, 1H), 8.61 (m, 1H), 7.67 (dd, *J* = 8.0, 2.0 Hz, 1H), 7.45 – 7.36 (m, 3H), 7.36 – 7.33 (m, 1H), 7.28 (dd, *J* = 8.4, 4.6 Hz, 1H), 7.24 – 7.12 (m, 4H), 7.05 (m, 1H), 6.28 (d, *J* = 1.9 Hz, 1H), 3.42 (s, 2H), 3.37 (s, 2H), 2.17 (s, 3H) ppm.

^{13}C NMR (176 MHz, CDCl$_3$): δ 160.8 (d, $^{1}J_{C-F}$ = 248.4 Hz), 154.2, 148.0, 138.2, 135.2, 134.7, 133.3, 131.5 (2C), 131.2 (d, $^{3}J_{C-F}$ = 9.1 Hz), 130.6 (2C), 129.2, 126.3, 123.52, 123.49, 122.4, 121.0, 119.2 (d, $^{3}J_{C-F}$ = 15.6 Hz), 119.1, 115.5 (d, $^{2}J_{C-F}$ = 22.1 Hz), 60.6, 53.5, 42.3 ppm.

^{19}F NMR (376 MHz, CDCl$_3$): δ -111.50 (s, 1F) ppm.

IR (neat) ν_{max}: 1738, 1587, 1469, 1417, 1374, 1228, 1181, 1105, 1070, 1011, 819, 759, 699, 625 cm^{-1}.

HRMS (ESI$^+$): exact mass calculated for [M+H]$^+$ ($C_{24}H_{22}{}^{79}BrFN_3O_2{}^{32}S$)$^+$ requires *m/z* 514.0595, found *m/z* 514.0602.

R$_f$: 0.55 *n*-heptane:(EtOAc:EtOH:NH4OH (3:1:2%)) 1:1.

3.4 Experimental evidence for stereoinversion

To unambiguously confirm the inversion of configuration of the enantiopure starting alcohol (*R*)-(+)-1-phenylethyl alcohol, **2s** was independently synthesized starting from commercially available, optically pure (*S*)-α-methylbenzylamine. Comparison of the chiral HPLC chromatograms of the amine **2s** produced by deprotective alkylation and following the procedure reported below, indicates that the configuration of both products is identical, confirming that deprotective alkylation proceeds through the S_N2 mechanism.

(*S*)-2-(3,4-Dimethoxyphenyl)-*N*-(1-phenylethyl)acetamide (S24)

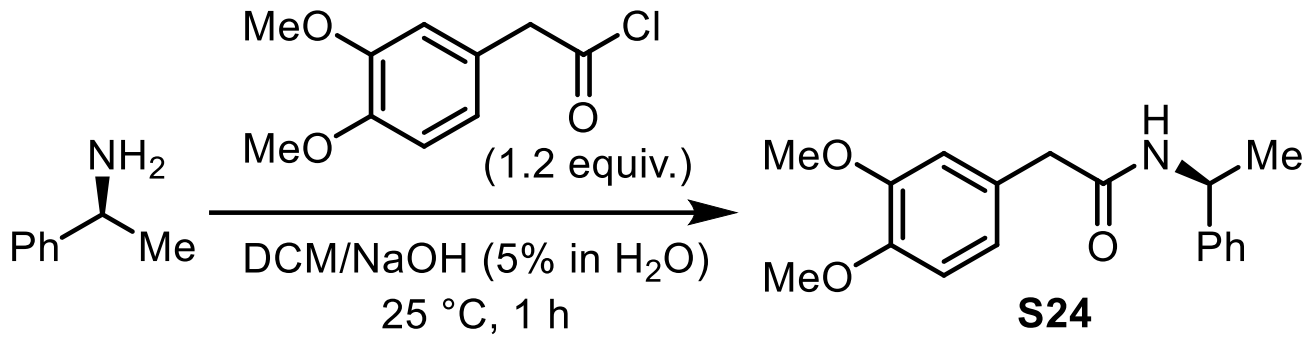

To a stirred mixture of (*S*)-α-methylbenzylamine (606 mg, 5.00 mmol, 1.00 equiv.) and DCM/5% aq. NaOH (1:1.5, 10 mL) a solution of 2-(3,4-dimethoxyphenyl)acetyl chloride (1.288 g, 6.00 mmol, 1.20 equiv.) in DCM (10 mL) was added dropwise at 0 °C. After stirring at room temperature (24 °C) for 1 h, the reaction mixture was extracted with DCM (30 mL × 2), washed with water (20 mL), dried over anhydrous Na_2SO_4, filtered, and the solvent was concentrated *in vacuo*. The residue was recrystallized from 30% EtOAc/*n*-heptane mixture to afford the title compound as a colourless crystals (1.342 g, 4.35 mmol, 87% yield).

^{1}H NMR (400 MHz, CDCl$_3$): δ 7.34 – 7.15 (m, 5H), 6.84 (d, *J* = 8.1 Hz, 1H), 6.81 – 6.71 (m, 2H), 5.63 (d, *J* = 7.6 Hz, 1H), 5.13 (p, *J* = 7.0 Hz, 1H), 3.88 (s, 3H), 3.84 (s, 3H), 3.52 (s, 2H), 1.40 (d, *J* = 6.9 Hz, 3H) ppm.

All analytical data were in accordance with those reported in literature.[63]

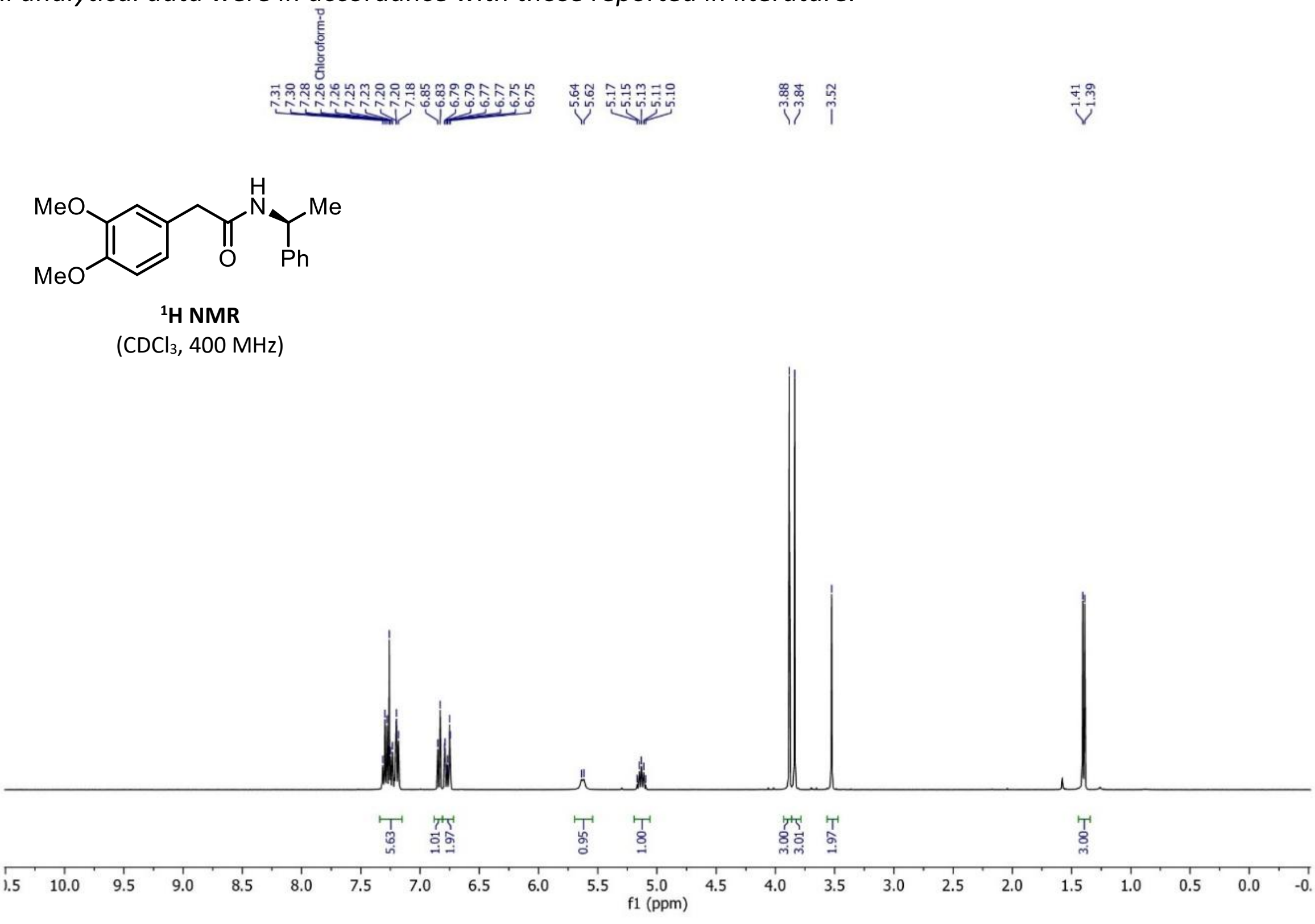

(*S*)-*N*-(3,4-Dimethoxyphenethyl)-1-phenylethan-1-amine (S25)

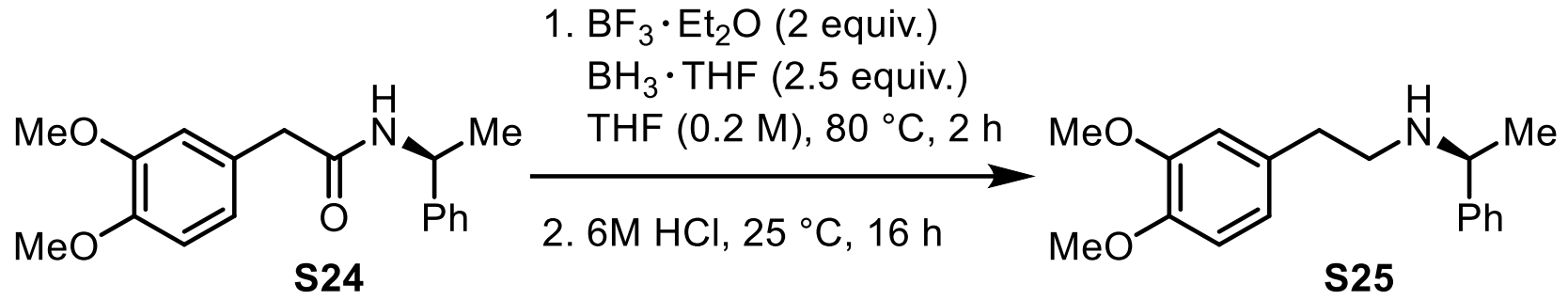

A 100-mL Schlenk flask, fitted with a magnetic stirring bar and connected to both an argon inlet tube and a water condenser, was charged with anhydrous THF (22 mL), amide **S24** (1.29 g, 4.30 mmol, 1.00 equiv.) and $BF_3 \cdot Et_2O$ (1.06 mL, 8.60 mmol, 2.00 equiv.). The resulting solution was heated at reflux (80 °C), and $BH_3 \cdot THF$ (1.0 M in THF, 10.8 mL, 10.8 mmol, 2.50 equiv.) was added dropwise at that temperature. The solution was heated at reflux for an additional 2 h period and was cooled to 0 °C. The reaction mixture was quenched by the slow addition of a 6M aqueous HCl solution (30 mL), and the resulting mixture was stirred for 16 h at room temperature (24 °C). The solution was basified with solid KOH until the complete precipitation of the amine as an oily residue. The solvent was removed *in vacuo*, and the aqueous phase was extracted with DCM (3 × 50 mL). The combined organic phases were dried over anhydrous Na_2SO_4 and concentrated *in vacuo*. The crude material was subjected to column chromatography (silica gel, 100% EtOAc) to obtain the desired amine as a yellow oil (258 mg, 0.904 mmol, 21% yield).

^{1}H NMR (400 MHz, $CDCl_3$): δ 7.35 – 7.19 (m, 5H), 6.78 (d, *J* = 8.1 Hz, 1H), 6.74 – 6.63 (m, 2H), 3.86 (s, 3H), 3.84 (s, 3H), 3.77 (q, *J* = 6.6 Hz,1H), 2.79 – 2.65 (m, 4H), 1.60 (br s, 1H), 1.33 (d, *J* = 6.6 Hz, 3H) ppm.

All analytical data were in accordance with those reported in literature.[63]

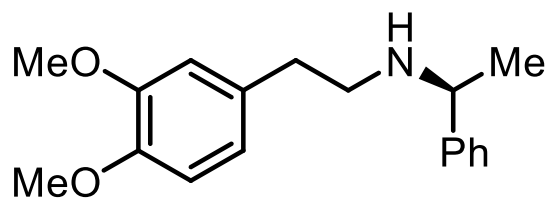

^{1}H NMR
($CDCl_3$, 400 MHz)

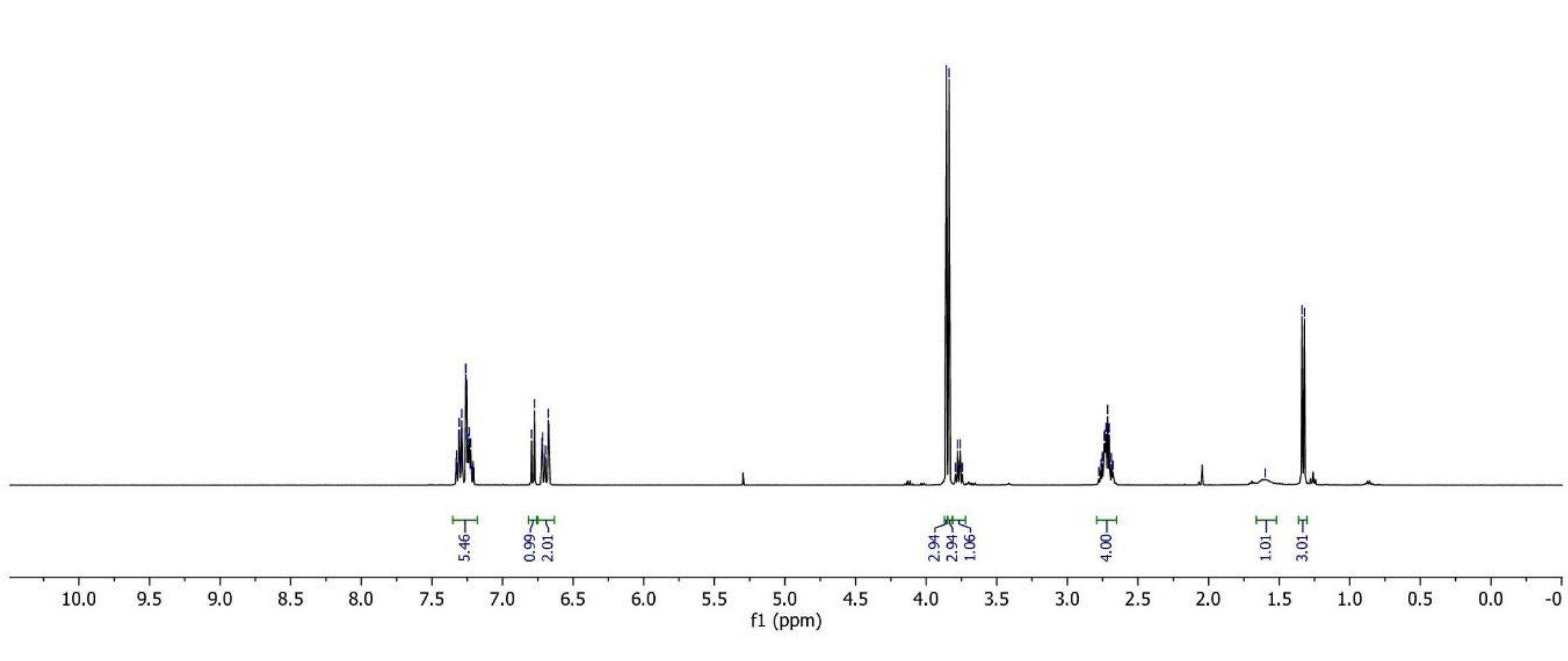

(*S*)-*N*-(3,4-Dimethoxyphenethyl)-*N*-methyl-1-phenylethan-1-amine (2s)

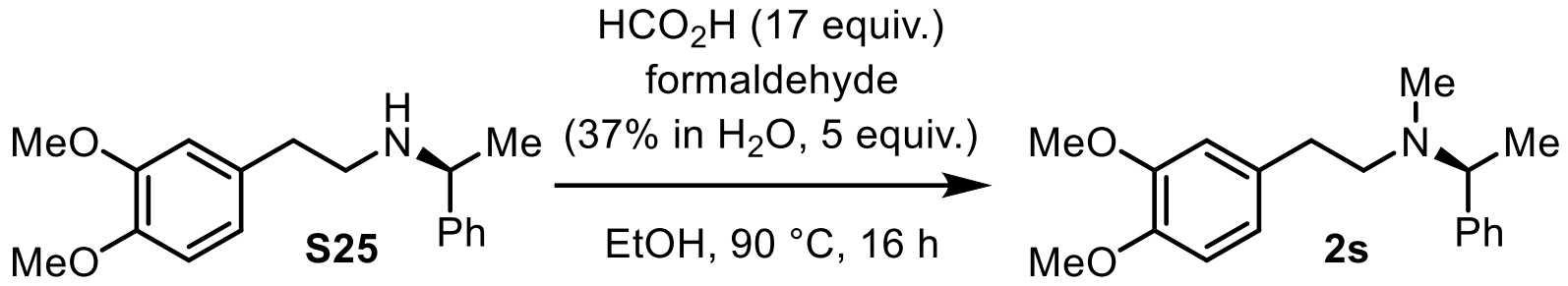

A solution of amine **S25** (128 mg, 0.45 mmol, 1.00 equiv.), formic acid (0.289 mL, 7.65 mmol, 17.0 equiv.) and formaldehyde (37% in water, 0.168 mL, 2.25 mmol, 5.00 equiv.) in absolute ethanol (4.5 mL) was heated at reflux (90 °C) for 16 h. The solvent was then removed under reduced pressure and the residue was treated with a sat. aq. solution of $NaHCO_3$ (10 mL) and extracted with dichloromethane (3 × 15 mL). The organic layers were collected, dried over anhydrous Na_2SO_4 and concentrated *in vacuo.* The crude material was subjected to column chromatography (silica gel, 100% EtOAc) to obtain the desired amine as a yellow oil (102 mg, 0.341 mmol, 76% yield).

^{1}H NMR (400 MHz, CDCl$_3$): δ 7.34 – 7.28 (m, 4H), 7.25 – 7.19 (m, 1H), 6.76 (d, *J* = 8.1 Hz, 1H), 6.68 – 6.60 (m, 2H), 3.85 (s, 3H), 3.83 (s, 3H), 3.64 (q, *J* = 6.7 Hz, 1H), 2.77 – 2.63 (m, 3H), 2.58 – 2.50 (m, 1H), 2.30 (s, 3H), 1.38 (d, *J* = 6.7 Hz, 3H) ppm.

Enantiomeric ratio: 99:1 determined by chiral HPLC analysis: Chiralcel OD-H, 50% (*n*-heptane/IPA/DEA 93:7:0,1) : 50% (*n*-heptane + 0,1% IPA), 0.7 mL/min, 25 °C, detection at 220 nm, retention time (min): 12.4 (major); 13.3 (minor).

All analytical data were in good accordance with **2s**.

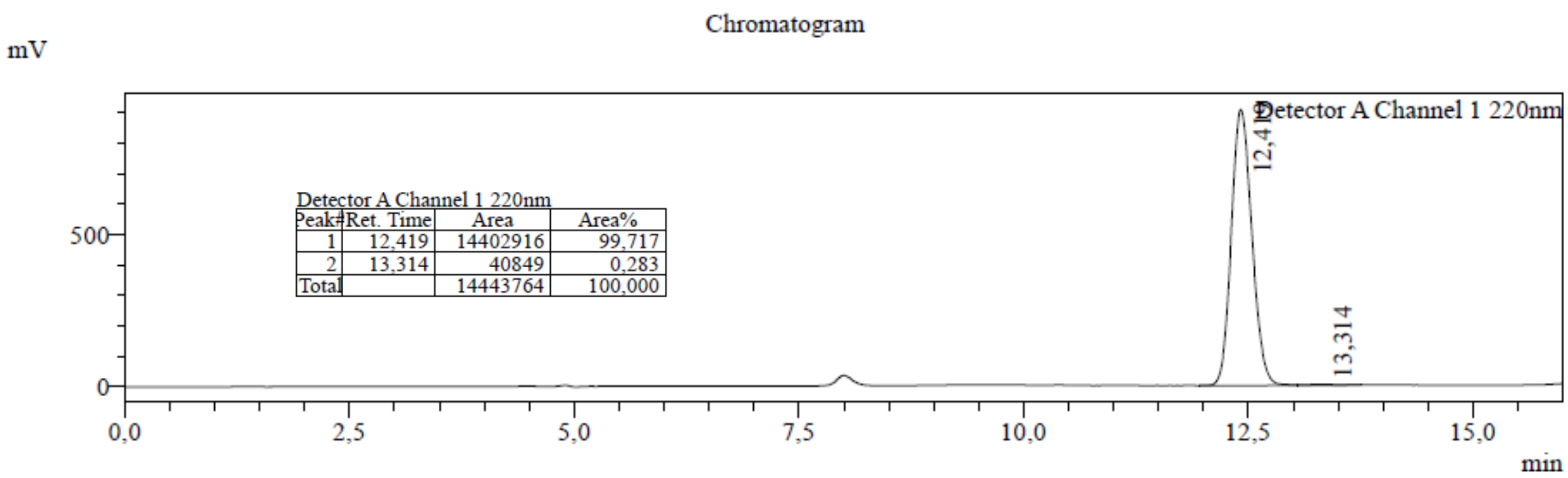

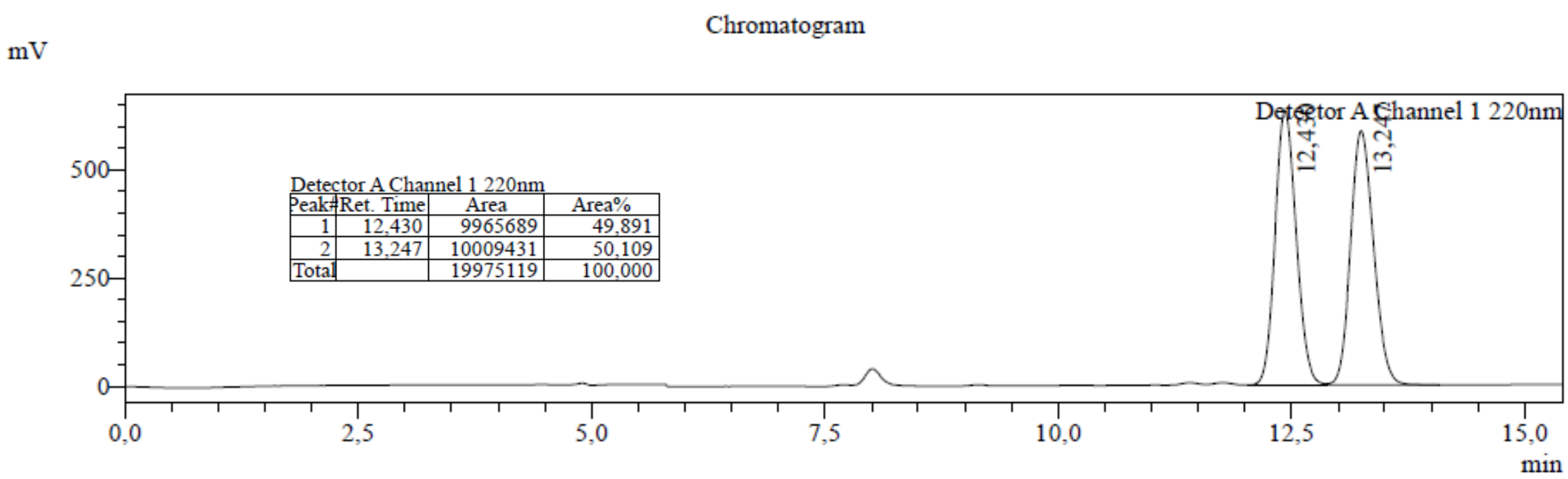

3.5 Scale-up procedure

***N*-(4-((6-Methoxyquinolin-8-yl)amino)pentyl)-2,4,6-tris(trifluoromethyl)benzenesulfonamide (3g)**

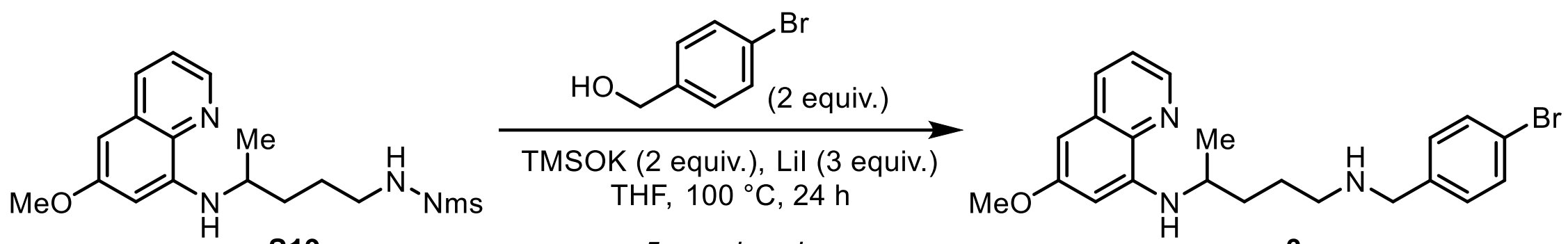

A flame-dried 150 mL Schlenk flask under argon atmosphere was charged with sulfonamide **S19** (3.02 g, 5.00 mmol, 1.00 equiv.), lithium iodide (2.01 g, 15.00 mmol, 3.00 equiv.) and 4-bromobenzyl alcohol (1.87 g, 10.00 mmol, 2.00 equiv.). The Schlenk flask was evacuated, back-filled with argon and anhydrous THF (50 mL) was added. Subsequently, a solution of TMSOK (1 M in THF, 5 mL, 10.00 mmol, 1.50 equiv.) was added to the reaction mixture, immediately forming cloudy suspension. The Schlenk flask was tightly sealed with a Telfon screw-cap and placed into a preheated oil bath at 100 °C. The reaction mixture was stirred for 24 h at 100 °C. After that time, the reaction mixture was cooled to room temperature (25 °C), quenched with water (20 mL) and transferred to separatory funnel. The crude material was diluted with 1M NaOH solution (50 mL) and extracted with DCM (3 x 50 mL). The combined organic phases were dried over anhydrous Na_2SO_4 and concentrated *in vacuo*. The crude material was subjected to flash column chromatography (silica gel, 10%-20% EtOH in EtOAc with 1% NH_4OH (25% in H_2O)) to obtain the title compound as a brown oil (1.398 g, 3.26 mmol, 65% yield). Since Primaquine exhibits moderate stability in its free form when stored for extended periods, the product was converted to its hydrochloride salt by treating it with a 2M solution of HCl in Et_2O (10 mL). The orange crystals that formed were filtered off, dried under reduced pressure, and stored without argon protection.

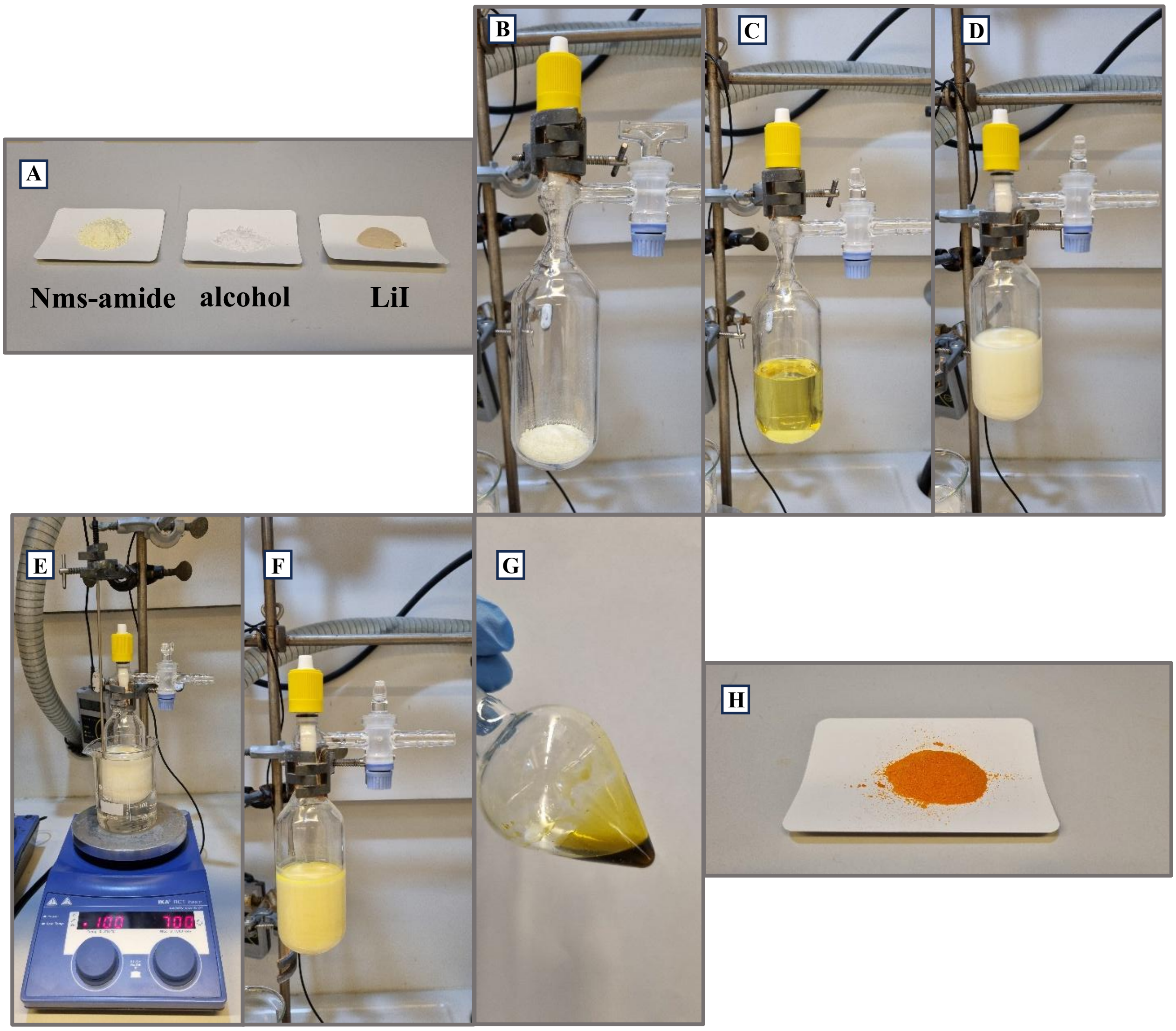

A. Substrates after weighing.
B. Solid substrates transferred to Schlenk flask.
C. Substrates dissolved in anhydrous THF.
D. The reaction mixture immediately after adding TMSOK.
E. Reaction set-up (Schlenk flask placed in a preheated oil bath).
F. Reaction mixture after 24 h.
G. Product of the reaction after column chromatography.
H. Product of the reaction as a hydrochloride salt.

3.6 Unsuccessful and low-yielding examples

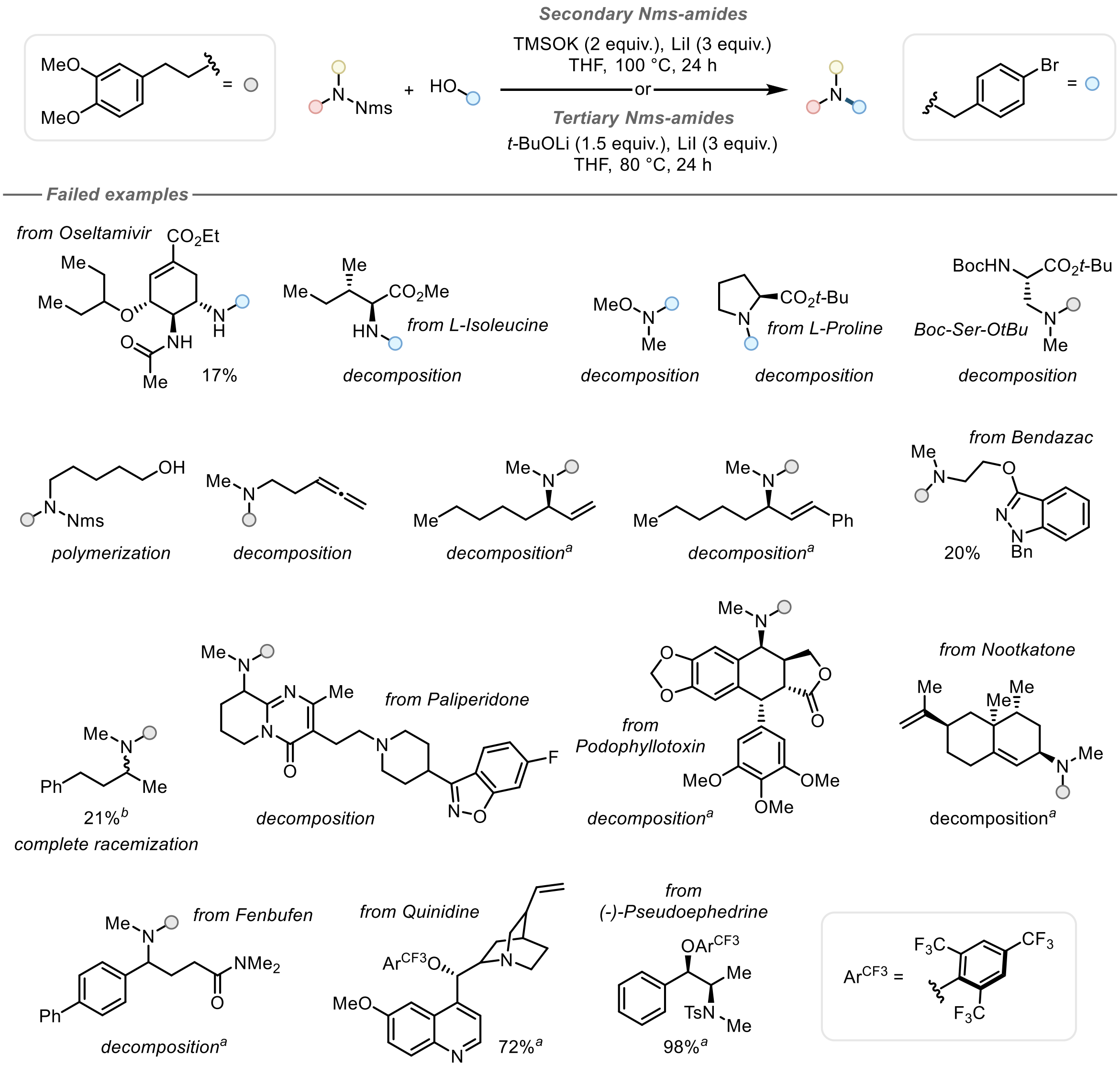

Scheme S1. Examples of unsuccessful substrates. Secondary Nms-amide reaction conditions: sulfonamide (0.15 mmol), alcohol (0.30 mmol), TMSOK (0.30 mmol), LiI (0.45 mmol), THF (1.5 mL), 80 °C, 24 h, air atmosphere. Tertiary Nms-amide reaction conditions: sulfonamide (0.15 mmol), alcohol (0.18 mmol), *t*-BuOLi (0.225 mmol), LiI (0.45 mmol), THF (1.5 mL), 80 °C, 24 h, air atmosphere. (a) Reaction performed without LiI. (b) Reaction performed at 100 °C.

3.7 Penfluridol synthesis

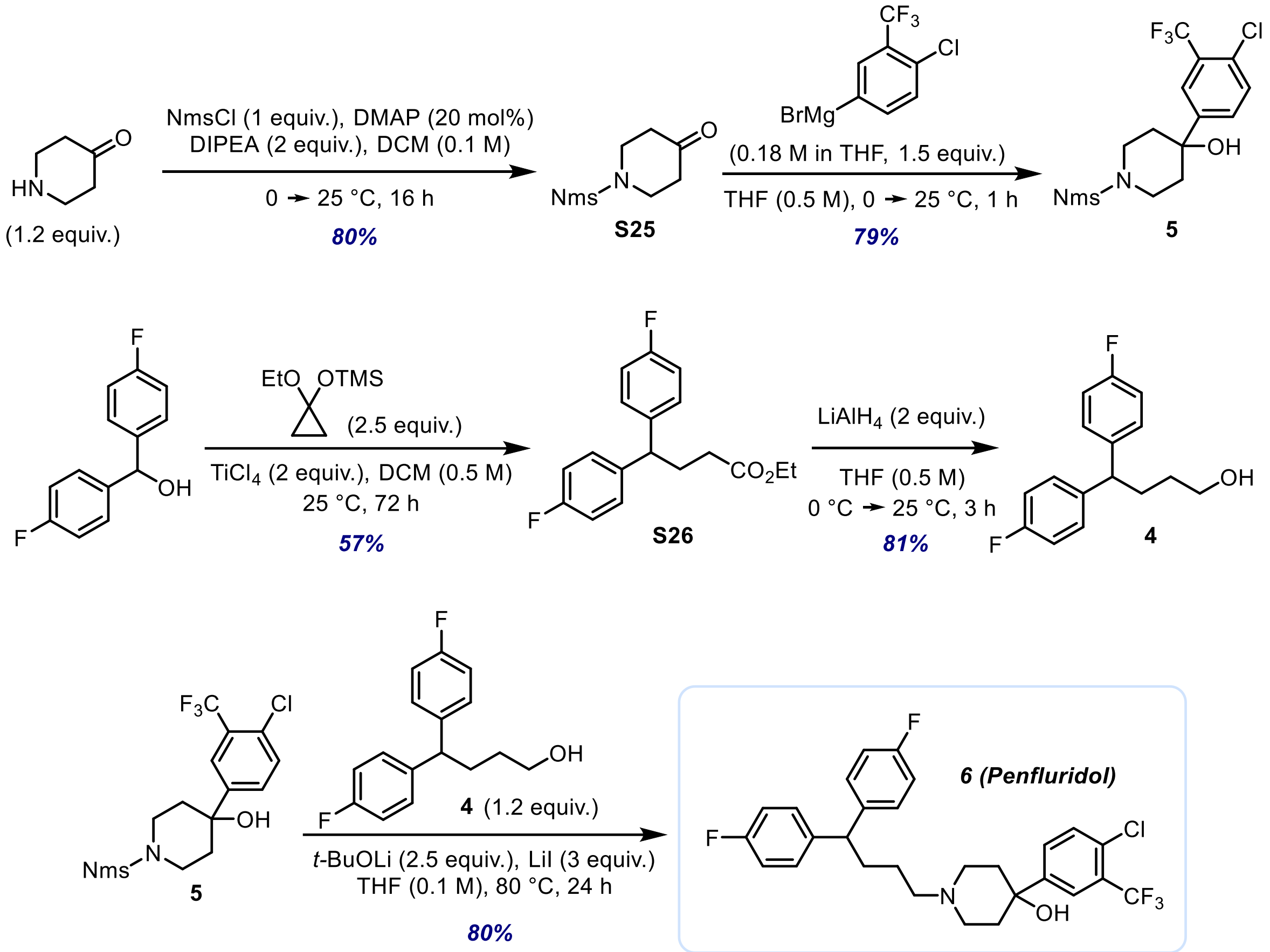

Scheme S2. Synthetic route towards Penfluridol.

1-((2,4,6-Tris(trifluoromethyl)phenyl)sulfonyl)piperidin-4-one (S26)

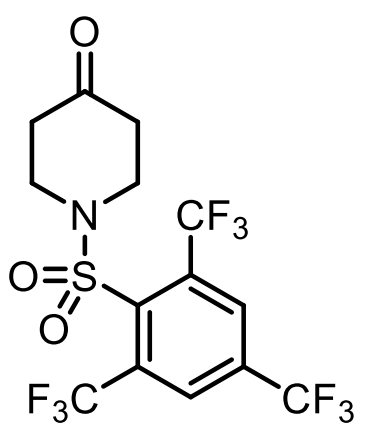

4-Piperidone monohydrate hydrochloride (564 mg, 3.60 mmol, 1.20 equiv.) was dissolved in DCM (30 mL) and placed in 100 mL separatory funnel. Subsequently, the organic phase was extracted with 1M NaOH aqueous solution (20 mL). Then, the organic phase was separated and transferred to 100 mL round-bottom flask, followed by addition of DMAP (73.3 mg, 0.60 mmol, 20 mol%) and *N*,*N*-diisopropylethylamine (1.05 mL, 6.00 mmol, 2.00 equiv.) at room temperature (25 °C). The resulting mixture was cooled to 0 °C and 2,4,6-tris(trifluoromethyl)benzenesulfonyl chloride (1.142 g, 3.00 mmol, 1.00 equiv.) was added in one portion. The reaction was allowed to slowly warm to room temperature over 16 h (without removing the cooling bath after the addition of sulfonyl chloride). Then, the solvents were removed under reduced pressure and the crude material was subjected to flash column chromatography (silica gel, 10%-50% EtOAc in *n*-heptane) to obtain the title compound as a white solid (1.063 g, 2.40 mmol, 80% yield).

^{1}H NMR (400 MHz, Acetone-d_6): δ 8.60 (s, 2H), 3.71 (t, *J* = 6.2 Hz, 4H), 2.54 (t, *J* = 6.2 Hz, 4H) ppm.

^{13}C NMR (101 MHz, Acetone-d_6): δ 204.8, 145.3, 134.7 (q, $^{2}J_{C-F}$ = 35.0 Hz), 133.2 (q, $^{2}J_{C-F}$ = 33.9 Hz, 2C), 131.1 – 130.6 (m, 2C), 123.2 (q, $^{1}J_{C-F}$ = 274.7 Hz, 2C), 123.1 (q, $^{1}J_{C-F}$ = 272.9 Hz), 46.5 (2C), 41.9 (2C) ppm.

^{19}F NMR (376 MHz, Acetone-d_6): δ -55.85 (s, 6F), -64.07 (s, 3F) ppm.

IR (neat) ν_{max}: 1741, 1363, 1282, 1192, 1165, 1150, 1088, 913 cm^{-1}.

HRMS (ESI$^-$): exact mass calculated for [M-H]$^-$ ($C_{14}H_9F_9NO_3{}^{32}S$)$^-$ requires *m/z* 442.0165, found *m/z* 442.0170.

Rf: 0.31 *n*-heptane:EtOAc 3:1.

4-(4-Chloro-3-(trifluoromethyl)phenyl)-1-((2,4,6-tris(trifluoromethyl)phenyl)sulfonyl)piperidin-4-ol (5)

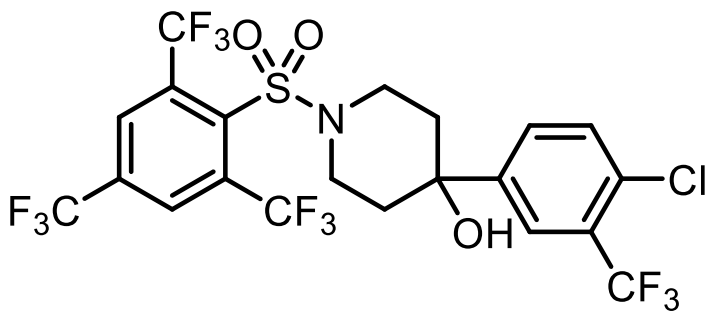

Grignard reagent synthesis: A flame-dried 25 mL Schlenk flask was charged with activated Mg turnings (182,3 mg, 7.50 mmol, 1.50 equiv.) and anhydrous THF (5 mL). To this suspension were added 2 drops of 1,2-dibromoethane. After 5 min, a solution of 4-bromo-1-chloro-2-(trifluoromethyl)benzene (1.30 g, 5.00 mmol, 1.00 equiv.) in anhydrous THF (5 mL) was slowly added to the suspension of Mg at room temperature (25 °C). The reaction was mildly exothermic. The concentration of Grignard reagent was determined using the Knochel titration method[64] (I_2/LiCl) and was found to be 0.18 M.

Grignard reagent addition: Ketone **S26** (222 mg, 0.50 mmol, 1.00 equiv.) was placed in a Schlenk flask under argon atmosphere, dissolved in anhydrous THF (1.0 mL) and cooled to 0 °C. Then, the previously prepared Grignard reagent (4.27 mL, 0.18M in THF, 1.50 equiv.) was added dropwise. The reaction mixture was left to warm to room temperature (25 °C) over 1 h. After that time, the reaction was quenched with saturated aqueous solution of NH_4Cl, diluted with water (10 mL), EtOAc (5 mL) and extracted with EtOAc (3 x 5 mL). The combined organic phases were dried over anhydrous Na_2SO_4 and concentrated *in vacuo*. The crude material was subjected to flash column chromatography (silica gel, 10%-30% EtOAc in *n*-heptane) to obtain the title compound as a white solid (246 mg, 0.394 mmol, 79% yield).

^{1}H NMR (400 MHz, $CDCl_3$): δ 8.29 (s, 2H), 7.82 (d, *J* = 2.1 Hz, 1H), 7.56 (dd, *J* = 8.4, 2.2 Hz, 1H), 7.49 (d, *J* = 8.4 Hz, 1H), 3.70 (dd, *J* = 13.8, 4.3 Hz, 2H), 3.46 (t, *J* = 12.3 Hz, 2H), 2.24 (td, *J* = 13.6, 4.6 Hz, 2H), 1.81 – 1.72 (m, 3H) ppm.

^{13}C NMR (101 MHz, $CDCl_3$): δ 146.6, 145.0, 134.4 (q, $^{2}J_{C-F}$ = 35.2 Hz), 133.1 (q, $^{2}J_{C-F}$ = 34.0 Hz, 2C), 131.8, 131.6, 129.72 – 129.31 (m, 2C), 129.26, 128.7 (q, $^{2}J_{C-F}$ = 31.2 Hz), 123.9 (q, $^{3}J_{C-F}$ = 5.3 Hz), 122.9 (q, $^{1}J_{C-F}$ = 273.4 Hz), 122.2 (q, $^{1}J_{C-F}$ = 275.6 Hz, 2C), 122.0 (q, $^{1}J_{C-F}$ = 273.5 Hz), 71.3, 43.0 (2C), 38.2 (2C) ppm.

^{19}F NMR (376 MHz, $CDCl_3$): δ -55.52 (s, 6F), -62.59 (s, 3F), -63.62 (s, 3F) ppm.

IR (neat) ν_{max}: 3383, 2959, 2924, 1708, 1281, 1181, 1135, 1058, 916, 726 cm^{-1}.

HRMS (ESI$^-$): exact mass calculated for $[M+Cl]^-$ $(C_{21}H_{14}F_{12}NO_3{}^{32}S^{35}Cl_2)^-$ requires *m/z* 657.9885, found *m/z* 657.9892.

Rf: 0.32 *n*-heptane:EtOAc 5:1.

Ethyl 4,4-bis(4-fluorophenyl)butanoate (S27)

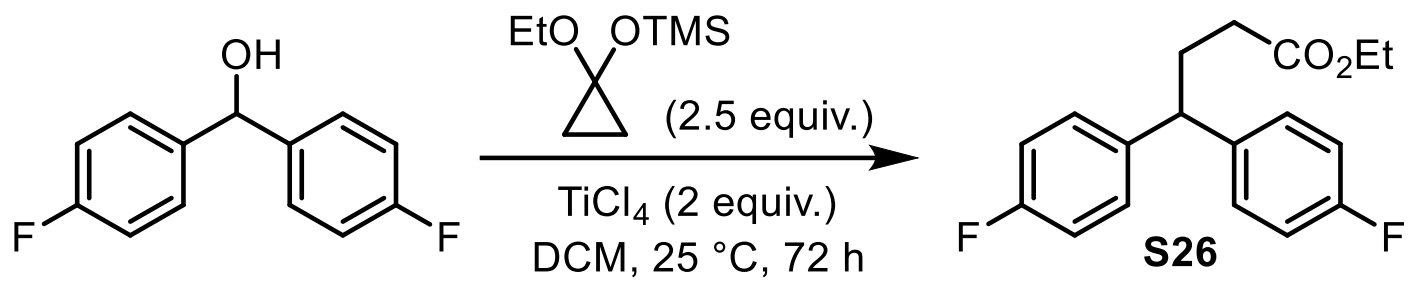

A modified procedure adopted from literature was used.[65] Under argon, bis-(4-fluorophenyl)-methanol (110 mg, 0.50 mmol, 1.00 equiv.), (1-ethoxycyclopropoxy)trimethylsilane (0.259 mL, 1.25 mmol, 2.50 equiv.), $TiCl_4$ (0.11 mL, 1.00 mmol, 2.00 equiv.) and anhydrous DCM (1 mL) were added to a flask equipped with a septum and a magnetic stir bar. The reaction mixture was stirred at room temperature (25 °C) for 72 h, followed by quenching with sat. aq. $NaHCO_3$ solution (10 mL) and extracting with EtOAc (3 × 30 mL). The combined extracts were washed with brine (30 mL), dried over anhydrous Na_2SO_4, and concentrated *in vacuo*. The resulting crude material was subjected to flash column chromatography (silica gel, 1%-10% EtOAc in *n*-heptane) to obtain the title compound as a yellow oil (86.1 mg, 0.283 mmol, 57% yield).

^{1}H NMR (400 MHz, $CDCl_3$): δ 7.17 (dd, *J* = 8.3, 5.5 Hz, 4H), 6.98 (t, *J* = 8.6 Hz, 4H), 4.11 (q, *J* = 7.1 Hz, 2H), 3.92 (t, *J* = 7.7 Hz, 1H), 2.37 – 2.29 (m, 2H), 2.28 – 2.21 (m, 2H), 1.24 (t, *J* = 7.1 Hz, 3H) ppm.

All analytical data were in accordance with those reported in literature.[65]

4,4-Bis(4-fluorophenyl)butan-1-ol (4)

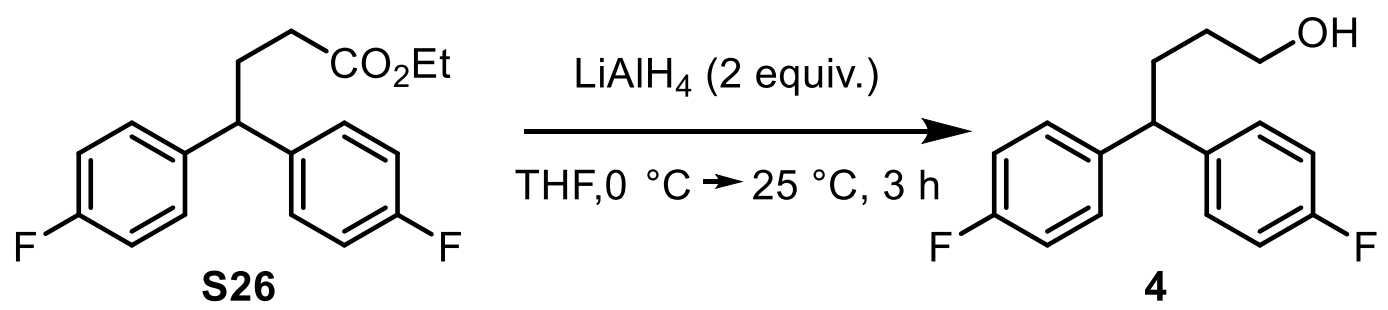

To a suspension of $LiAlH_4$ (152 mg, 4.00 mmol, 2.00 equiv.) in THF (3.0 mL) at 0 °C was slowly added solution of ester **S27** (609 mg, 2.00 mmol, 1.00 equiv.) in THF (1.0 mL) °C. The mixture was stirred at room temperature (25 °C) for 2 h, after which it was quenched by addition of a few drops of water at 0 °C. The reaction mixture was acidified with 2 M HCl (10 mL), extracted with EtOAc (3 × 10 mL), and then the combined organic phases were washed with brine (10 mL), dried over anhydrous Na_2SO_4, and concentrated *in vacuo*. The resulting crude material was subjected to flash column chromatography (silica gel, 5%-25% EtOAc in *n*-heptane) to obtain the title compound as a colourless oil (425 mg, 1.62 mmol, 81% yield).

^{1}H NMR (400 MHz, $CDCl_3$): δ 7.17 (dd, *J* = 8.5, 5.4 Hz, 4H), 6.97 (t, *J* = 8.7 Hz, 4H), 3.89 (t, *J* = 7.9 Hz, 1H), 3.65 (t, *J* = 6.4 Hz, 2H), 2.08 (q, *J* = 7.9 Hz, 2H), 1.51 (dt, *J* = 14.2, 6.5 Hz, 2H), 1.31 (br s, 1H) ppm.

All analytical data were in accordance with those reported in literature.[66]

1-(4,4-Bis(4-fluorophenyl)butyl)-4-(4-chloro-3-(trifluoromethyl)phenyl)piperidin-4-ol (6-*Penfluridol*)

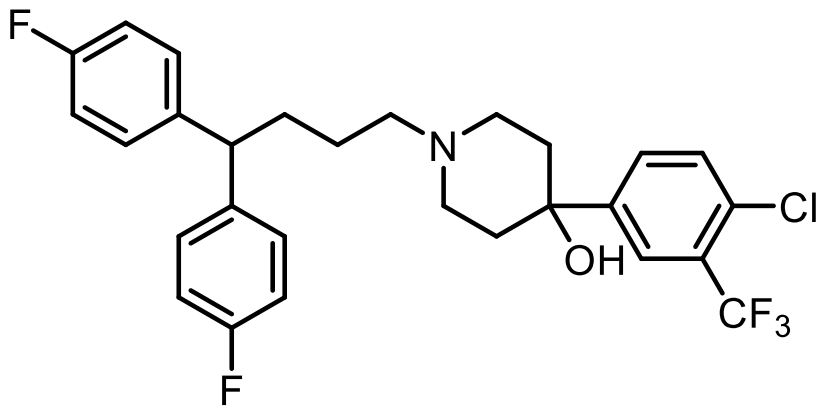

General procedure **GP2** was followed, using sulfonamide **5** (93.6 mg, 0.15 mmol, 1.00 equiv.), alcohol **4** (47.2 mg, 0.18 mmol, 1.20 equiv.) and *t*-BuOLi (30.0 mg, 0.375 mmol, 2.50 equiv.). The resulting crude material was subjected to flash column chromatography (silica gel, 50%-100% EtOAc in *n*-heptane) to obtain the title compound as a pale-yellow solid (62.6 mg, 0.119 mmol, 80% yield).

^{1}H NMR (400 MHz, CDCl$_3$): δ 7.88 – 7.83 (m, 1H), 7.61 – 7.56 (m, 1H), 7.46 (d, *J* = 8.4 Hz, 1H), 7.17 (dd, *J* = 8.3, 5.5 Hz, 4H), 6.96 (t, *J* = 8.6 Hz, 4H), 3.87 (t, *J* = 7.8 Hz, 1H), 2.76 (d, *J* = 11.0 Hz, 2H), 2.47 – 2.30 (m, 4H), 2.17 – 1.82 (m, 5H), 1.68 (d, *J* = 12.7 Hz, 2H), 1.52 – 1.41 (m, 2H) ppm.

^{13}C NMR (101 MHz, CDCl$_3$): δ 161.5 (d, $^{1}J_{C-F}$ = 244.5 Hz, 2C), 147.7, 140.6 (d, $^{4}J_{C-F}$ = 3.2 Hz, 2C), 131.5, 130.8, 129.3, 129.2 (d, $^{3}J_{C-F}$ = 7.8 Hz, 4C), 128.3 (q, $^{2}J_{C-F}$ = 31.1 Hz), 124.3 (q, $^{3}J_{C-F}$ = 5.3 Hz), 123.1 (q, $^{1}J_{C-F}$ = 273.3 Hz), 115.4 (d, $^{2}J_{C-F}$ = 21.1 Hz, 4C), 71.2, 58.7, 49.9, 49.4 (2C), 38.6 (2C), 34.0, 25.6 ppm.

^{19}F NMR (376 MHz, CDCl$_3$): δ -62.43 (s, 3F), -116.85 – -116.96 (m, 2F) ppm.

IR (neat) ν_{max}: 2945, 2820, 1710, 1507, 1316, 1220, 1128, 829, 530 cm^{-1}.

HRMS (ESI^{+}): exact mass calculated for $[M+H]^{+}$ $(C_{28}H_{28}{}^{35}ClF_5NO)^{+}$ requires *m/z* 524.1774, found *m/z* 524.1786.

Rf: 0.22 *n*-heptane:EtOAc 1:2.

3.8 Comparison with BEHT triflate

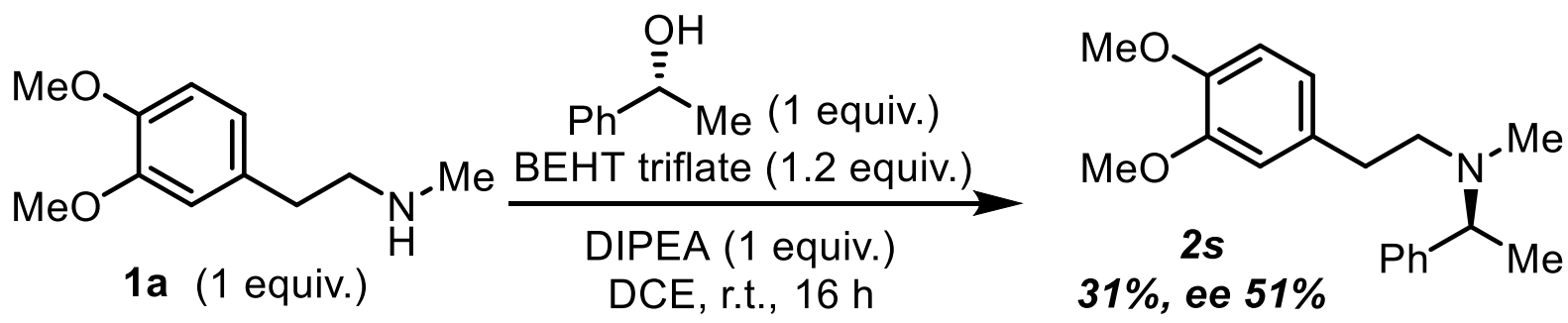

Charette's procedure was followed.[31] To a flame-dried and argon-purged 10 mL round-bottom flask equipped with a stir bar and a septum were added BEHT triflate (106 mg, 0.18 mmol, 1.20 equiv.) and anhydrous DCE (0.15 mL, 1 M). (*R*)-1-Phenylethyl Alcohol (0.15 mmol, 1.00 equiv.), 2-(3,4-dimethoxyphenyl)-*N*-methylethylamine (0.15 mmol, 1.00 equiv.) and *N*,*N*-diisopropylethylamine (0.174 mL, 0.15 mmol, 1.00 equiv) were rapidly added and the reaction was stirred for 18 h at room temperature (25 °C). The reaction was quenched by addition of sat. aq. $NaHCO_3$ (5.0 mL). The biphasic mixture was then transferred to a 60 mL separation funnel using DCM (20 mL) and the layers were separated. The aqueous layer was extracted with DCM (2 × 15 mL) and the organic layers were combined. The organic solution was dried over anhydrous Na_2SO_4, filtered, and concentrated *in vacuo*. The resulting crude material was subjected to flash column chromatography (silica gel, 0%-70% EtOAc in *n*-heptane) to obtain the title compound as a pale-yellow oil (14.1 mg, 0.047 mmol, 31% yield).

The analytical data is in accordance with **2s**.

Enantiomeric ratio: 74:26 determined by chiral HPLC analysis: Chiralcel OD-H, 50% (*n*-heptane/IPA/DEA 93:7:0,1):50% (*n*-heptane + 0,1% IPA), 0.7 mL/min, 25 °C, detection at 220 nm, retention time (min): 12.8 (major); 13.7 (minor).

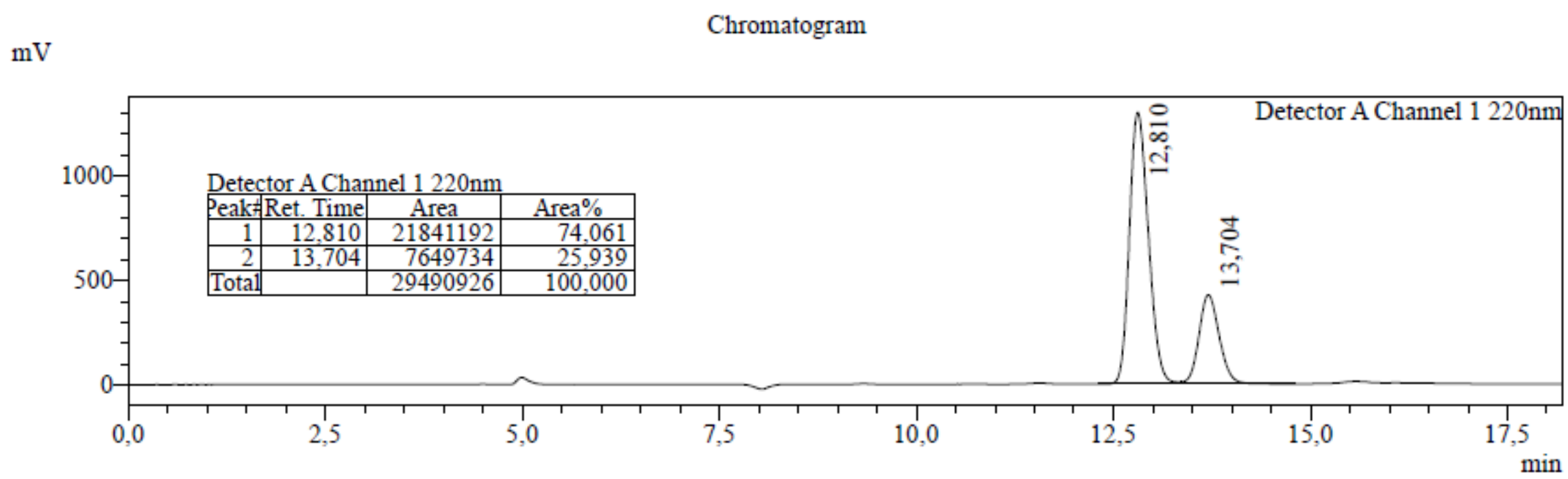

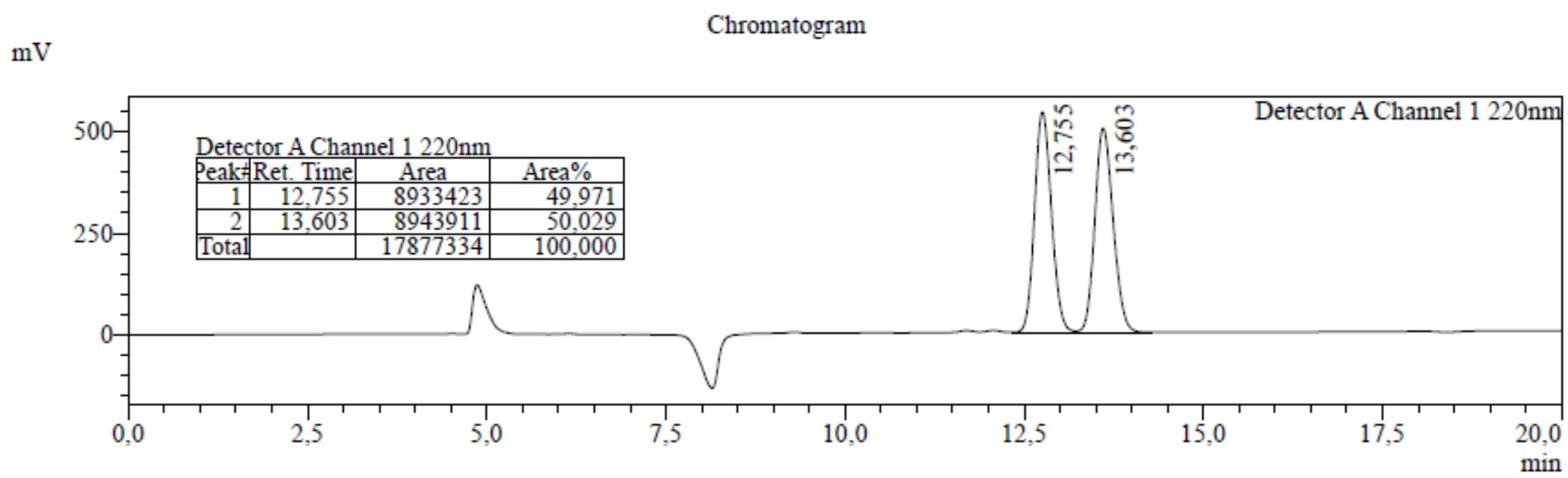

3.9 Synthesis of isotope labelled amines

tert-Butyl 4-(methyl-d_3)piperazine-1-carboxylate (8)

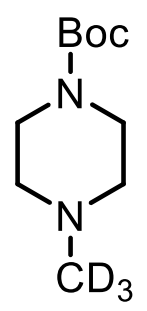

General procedure **GP2** was followed, using sulfonamide **7** (79.6 mg, 0.150 mmol, 1.0 equiv.) and methanol-d_4 (7.3 µL, 0.180 mmol, 1.2 equiv.) and lithium iodide in catalytic amount (4.0 mg, 0.03 mmol, 20 mol%). The resulting crude material was subjected to flash column chromatography (silica gel, 0%-30% MeOH in EtOAc) to obtain the title compound as a colourless oil (19.2 mg, 0.095 mmol, 63% yield).

^{1}H NMR (400 MHz, $CDCl_3$): δ 3.46 – 3.41 (m, 4H), 2.37 – 2.31 (m, 4H), 1.45 (s, 9H) ppm.

^{13}C NMR (101 MHz, $CDCl_3$): δ 154.9, 79.8, 54.9 (2C), 45.6 (br s), 43.9 – 43.3 (br m, 2C), 28.6 (3C) ppm.

IR (neat) ν_{max}: 2975, 2931, 2799, 1694, 1415, 1253, 1163, 1126, 990, 767 cm^{-1}.

HRMS (ESI$^+$): exact mass calculated for $[M+H]^+$ $(C_{10}H_{18}D_3N_2O_2)^+$ requires *m/z* 204.1786, found *m/z* 204.1783.

Rf: 0.26 EtOAc:MeOH 5:1.

***N*,*N*-Dimethyl-2,4,6-tris(trifluoromethyl)benzenesulfonamide-^{15}*N* (9)**

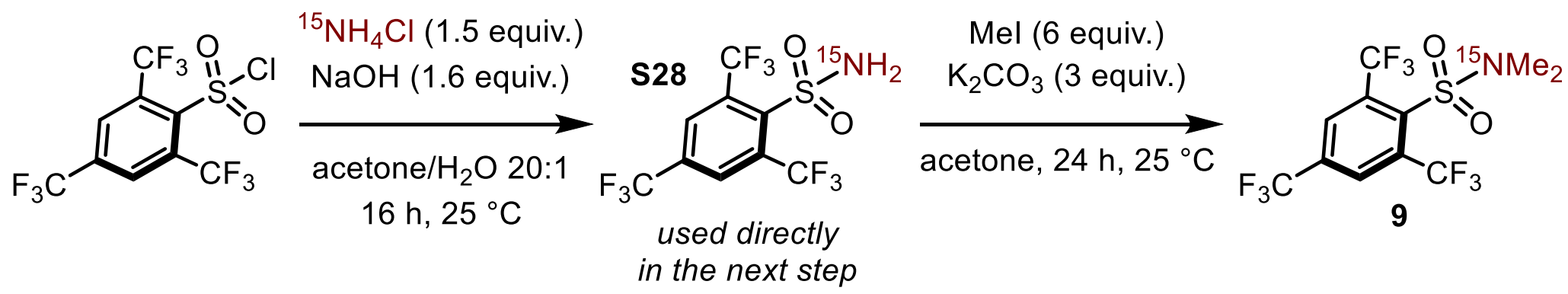

2,4,6-Tris(trifluoromethyl)benzenesulfonyl chloride (761 mg, 2.00 mmol, 1.00 equiv) was dissolved in a mixture of water and acetone (20:1, 10 mL). Then ^{15}*N*-ammonium chloride (Sigma-Aldrich, ≥ 98% atom ^{15}N) was added in one portion (163 mg, 3.00 mmol, 1.50 equiv.), followed by addition of finely ground sodium hydroxide (128 mg, 3.20 mmol, 1.60 equiv.). The reaction mixture was stirred at room temperature (25 °C) for 16 h. Then, the mixture was concentrated *in vacuo* and the residue was dissolved in EtOAc (50 mL) and distilled water (50 mL) and transferred to a separatory funnel. The aqueous phase was extracted with EtOAc (2 × 30 mL) and the combined organic phases were dried over anhydrous Na_2SO_4, filtered and concentrated under reduced pressure to afford the crude **S28** as a colourless solid. The product was used directly in the next step without further purification.

Methyl iodide (0.747 mL, 12.00 mmol, 6.00 equiv.) was added to a mixture of the sulfonamide **S28** and K_2CO_3 (0.83 g, 6.00 mmol, 3.00 equiv.) in acetone (10 mL) at room temperature (25 °C). The reaction mixture was stirred at the same temperature for 24 h. The progress of the reaction was monitored by TLC analysis. Then, H_2O was added, the phases were separated and the aqueous phase was extracted with EtOAc (2 × 30 mL). The combined organic phases were combined, dried over anhydrous Na_2SO_4, filtered and concentrated *in vacuo*. The

resulting crude material was subjected to flash column chromatography (silica gel, 5%-20% EtOAc in *n*-heptane) to obtain the title compound as a colourless solid (626 mg, 1.60 mmol, 80% yield over two steps).

^{1}H NMR (400 MHz, $CDCl_3$): δ 8.27 (s, 2H), 2.86 (s, 6H) ppm.

^{13}C NMR (101 MHz, $CDCl_3$): δ 144.8 (d, $^{3}J_{C\text{-}F}$ = 9.8 Hz), 134.2 (q, $^{2}J_{C\text{-}F}$ = 35.1 Hz), 133.2 (q, $^{2}J_{C\text{-}F}$ = 34.0 Hz, 2C), 129.6 – 129.1 (m, 2C), 122.2 (q, $^{1}J_{C\text{-}F}$ = 275.5 Hz, 2C), 122.0 (q, $^{1}J_{C\text{-}F}$ = 273.5 Hz), 37.7 (2C) ppm.

^{19}F NMR (376 MHz, $CDCl_3$): δ -55.99 (s, 6F), -63.61 (s, 3F) ppm.

IR (neat) ν_{max}: 2926, 2862, 1712, 1355, 1282, 1143, 962, 735, 568 cm^{-1}.

HRMS (ESI$^+$): exact mass calculated for $[M+Na]^+$ $(C_{11}H_8F_9{}^{15}NO_2{}^{32}S^{23}Na)^+$ requires *m/z* 412.9995, found *m/z* 412.9992.

Rf: 0.47 *n*-heptane:EtOAc 5:1.

(*S*)-3-(1-(Dimethylamino-^{15}*N*)ethyl)phenyl ethyl(methyl)carbamate (10a)

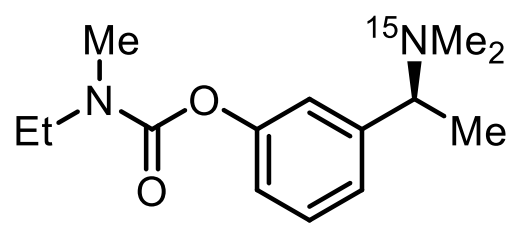

A modified general procedure **GP2** was followed, using sulfonamide **9** (58.5 mg, 0.15 mmol, 1.00 equiv.) and alcohol **S11** (40.2 mg, 0.18 mmol, 1.20 equiv.). The reaction was performed without lithium iodide. The resulting crude material was subjected to flash column chromatography (silica gel, 0%-20% MeOH in DCM) to obtain the title compound as a colourless oil (26.6 mg, 0.106 mmol, 71% yield).

^{1}H NMR (400 MHz, $CDCl_3$): δ 7.30 (t, *J* = 7.8 Hz, 1H), 7.14 (d, *J* = 7.7 Hz, 1H), 7.08 (s, 1H), 7.03 (d, *J* = 7.4 Hz, 1H), 3.50 – 3.33 (m, 3H), 3.05 and 2.98 (rotamers, s, 3H), 2.26 (s, 6H), 1.42 and 1.40 (rotamers, d, *J* = 2.1 Hz, 3H), 1.23 and 1.18 (rotamers, t, *J* = 7.0 Hz, 3H) ppm.

^{13}C NMR (101 MHz, $CDCl_3$): δ 154.7 and 154.5 (rotamers), 151.7, 144.4 and 144.3 (rotamers), 129.2, 124.5, 121.14 and 121.08 (rotamers), 120.8, 65.8, 44.2 (2C), 43.0, 34.3 and 33.9 (rotamers), 19.8, 13.4 and 12.6 (rotamers) ppm.

IR (neat) ν_{max}: 2974, 2934, 2766, 1716, 1396, 1226, 1157, 1086, 699 cm^{-1}.

HRMS (ESI$^+$): exact mass calculated for $[M+H]^+$ $(C_{14}H_{23}{}^{15}NNO_2)^+$ requires *m/z* 252.1724, found *m/z* 252.1719.

Rf: 0.25 DCM:MeOH 10:1.

$[\alpha]_D^{23}$ = –22.2 (*c* = 0.82, CH_2Cl_2).

Enantiomeric ratio: 97:3 determined by chiral HPLC analysis: Lux-Cellulose-1, 90% (98% *n*-heptane + 2% EtOH + 0,2% TFA):10% IPA, 1.0 mL/min, 25 °C, detection at 210 nm, retention time (min): 28.5 (minor); 33.1 (major).

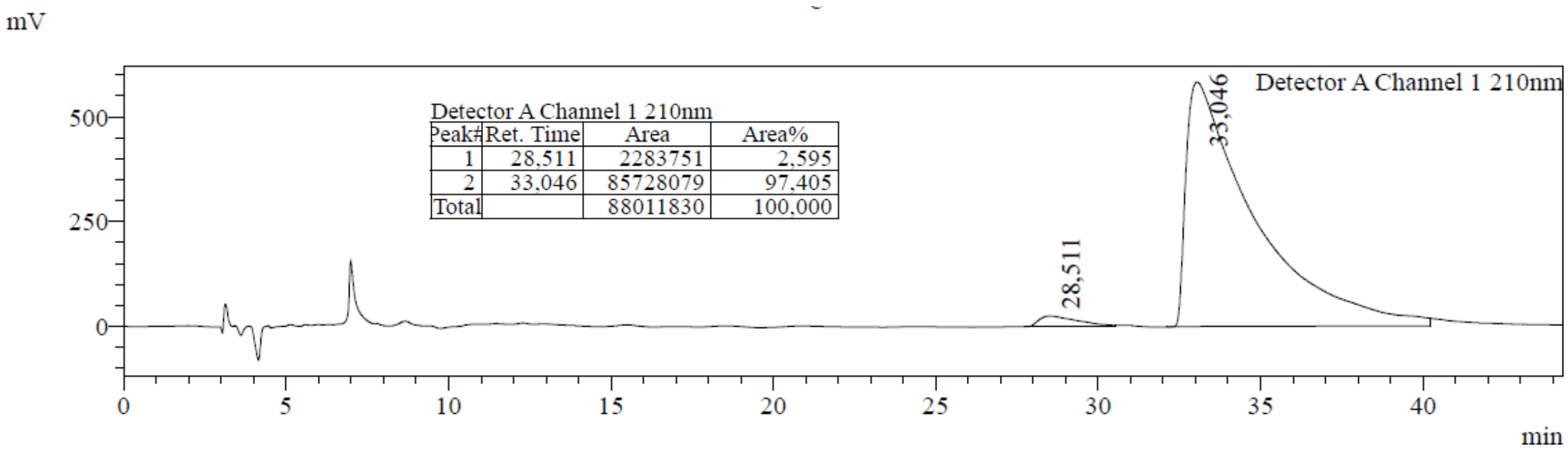

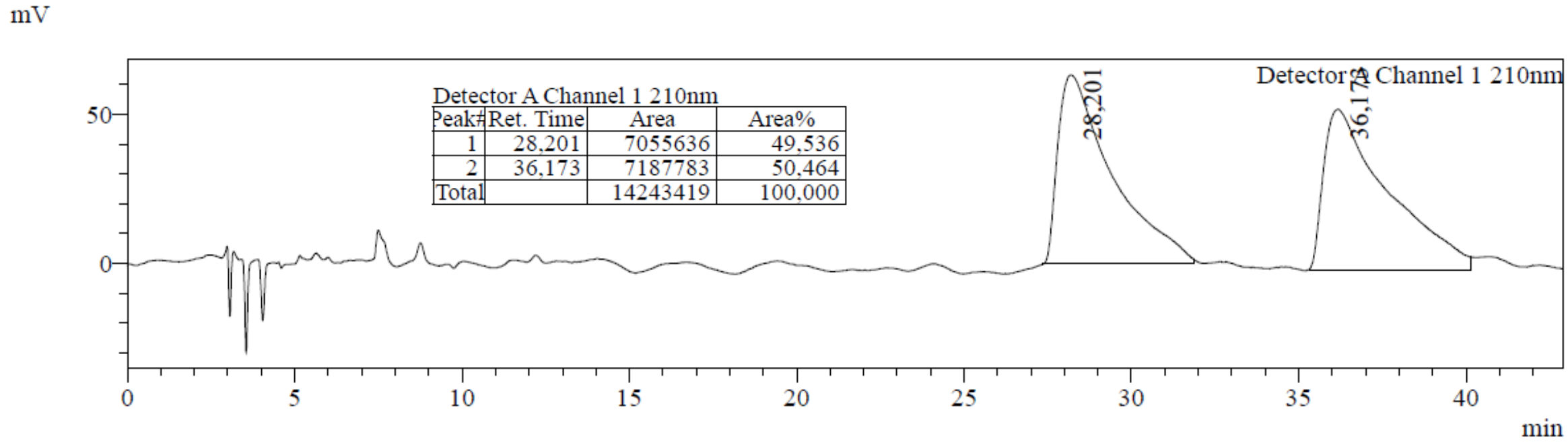

1-(3-(Dimethylamino-^{15}*N*)propyl)-1-(4-fluorophenyl)-1,3-dihydroisobenzofuran-5-carbonitrile (10b)

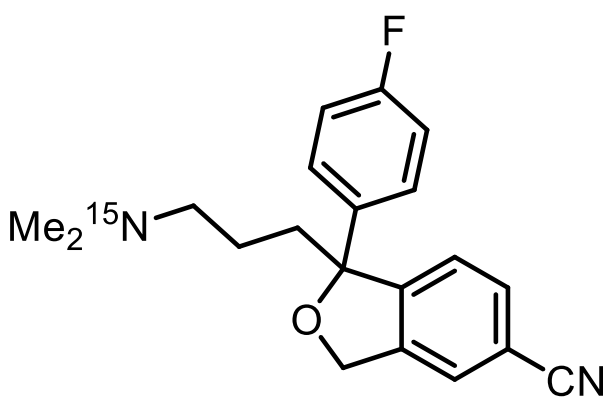

General procedure **GP2** was followed, using sulfonamide **9** (58.5 mg, 0.15 mmol, 1.00 equiv.) and alcohol **S13** (53.5 mg, 0.18 mmol, 1.20 equiv.). The resulting crude material was subjected to flash column chromatography (silica gel, 0%-20% MeOH in DCM) to obtain the title compound as a colourless oil (30.4 mg, 0.093 mmol, 62% yield).

^{1}H NMR (400 MHz, CDCl$_3$): δ 7.59 (d, *J* = 9.1 Hz, 1H), 7.50 (s, 1H), 7.45 – 7.36 (m, 3H), 7.05 – 6.96 (m, 2H), 5.22 – 5.12 (m, 2H), 2.26 – 2.08 (m, 10H), 1.52 – 1.39 (m, 1H), 1.38 – 1.27 (m, 1H) ppm.

^{13}C NMR (101 MHz, CDCl$_3$): δ 162.2 (d, $^1J_{C\text{-}F}$ = 246.3 Hz), 149.6, 140.5, 139.7 (d, $^4J_{C\text{-}F}$ = 3.2 Hz), 132.0, 126.9 (d, $^3J_{C\text{-}F}$ = 8.1 Hz, 2C), 125.3, 122.9, 118.8, 115.5 (d, $^2J_{C\text{-}F}$ = 21.4 Hz, 2C), 111.9, 91.3, 71.4, 59.6 (d, $^1J_{C\text{-}N}$ = 3.9 Hz), 45.5 (d, $^1J_{C\text{-}N}$ = 4.1 Hz, 2C), 39.1 (d, $^3J_{C\text{-}N}$ = 1.9 Hz), 22.3 (d, $^2J_{C\text{-}N}$ = 2.5 Hz) ppm.

^{19}F NMR (376 MHz, CDCl$_3$): δ -115.47 (tt, *J* = 8.5, 5.3 Hz, 1F) ppm.

IR (neat) ν_{max}: 2946, 2857, 2766, 2229, 1506, 1224, 1033, 830, 597 cm^{-1}.

HRMS (ESI$^+$): exact mass calculated for $[M+H]^+$ $(C_{20}H_{22}F^{15}NNO)^+$ requires *m/z* 326.1681, found *m/z* 326.1677.

Rf: 0.24 DCM:MeOH 10:1.

3-(3-Chloro-10,11-dihydro-5*H*-dibenzo[*b*,*f*]azepin-5-yl)-*N*,*N*-dimethylpropan-1-amine-*N*-^{15}*N* (10c)

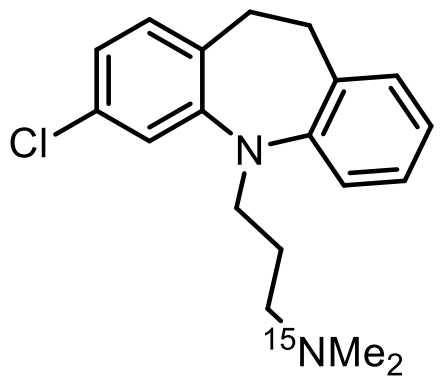

General procedure **GP2** was followed, using sulfonamide **9** (58.5 mg, 0.15 mmol, 1.00 equiv.) and alcohol **S15** (51.8 mg, 0.18 mmol, 1.20 equiv.). The resulting crude material was subjected to flash column chromatography (silica gel, 0%-20% MeOH in DCM) to obtain the title compound as a pale-yellow oil (34.4 mg, 0.109 mmol, 73% yield).

^{1}H NMR (400 MHz, $CDCl_3$): δ 7.17 – 7.04 (m, 4H), 7.00 – 6.93 (m, 2H), 6.85 (dd, *J* = 8.1, 2.0 Hz, 1H), 3.74 (t, *J* = 6.9 Hz, 2H), 3.18 – 3.07 (m, 4H), 2.29 (t, *J* = 7.2 Hz, 2H), 2.15 (s, 6H), 1.71 (p, *J* = 7.3 Hz, 2H) ppm.

^{13}C NMR (101 MHz, $CDCl_3$): δ 149.2, 148.1, 135.2, 131.68, 131.66, 131.3, 129.6, 126.7, 123.4, 122.1, 120.7, 120.0, 57.7 (d, *J* = 4.0 Hz), 49.1 (d, *J* = 2.2 Hz), 45.7 (d, *J* = 4.0 Hz, 2C), 32.3, 31.7, 26.3 (d, *J* = 2.5 Hz) ppm.

IR (neat) ν_{max}: 2921, 2854, 2814, 2762, 1583, 1484, 1406, 1226, 939, 748 cm^{-1}.

HRMS (ESI$^+$): exact mass calculated for $[M+H]^+$ $(C_{19}H_{24}{}^{35}Cl^{15}NN)^+$ requires *m/z* 316.1593, found *m/z* 316.1588.

Rf: 0.31 DCM:MeOH 10:1.

4. Mechanistic studies

4.1 Intermediate isolation

2-(3-Phenylpropoxy)-1,3,5-tris(trifluoromethyl)benzene (11)

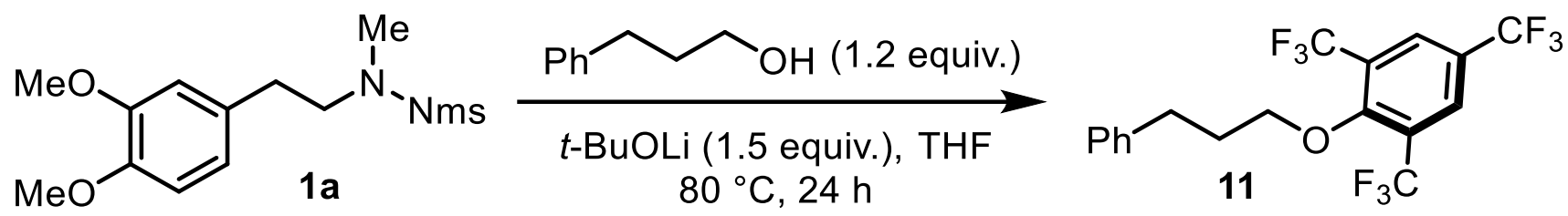

In a glovebox, an oven-dried vial was charged with solid lithium *tert*-butoxide (36.0 mg, 0.45 mmol, 1.50 equiv.), after which it was capped and removed from the glovebox. Then, the Nms-amide **1a** (162.0 mg, 0.30 mmol, 1.00 equiv.) was added, followed by addition of 3-phenylpropanol (49.0 mg, 0.36 mmol, 1.20 equiv.) dissolved in THF (3.0 mL). The vial was tightly sealed and placed into a metal heating-block. The reaction mixture was stirred for 24 h at 80 °C. After that time, the reaction mixture was cooled to room temperature (25 °C), quenched with water (3.0 mL) and transferred to separatory funnel. The crude material was diluted with a 1M aqueous solution of NaOH (10 mL) and extracted with DCM (3 x 15 mL). The combined organic phases were dried over anhydrous Na_2SO_4 and concentrated *in vacuo*. The resulting crude material was subjected to flash column chromatography (silica gel, 100 % *n*-heptane) to obtain the title compound as a colourless oil (50.1 mg, 0.120 mmol, 40% yield).

^{1}H NMR (400 MHz, CDCl$_3$): δ 8.09 (s, 2H), 7.32 (t, *J* = 7.4 Hz, 2H), 7.28 – 7.19 (m, 3H), 4.16 (t, *J* = 6.4 Hz, 2H), 2.88 – 2.81 (m, 2H), 2.26 – 2.16 (m, 2H) ppm.

^{13}C NMR (101 MHz, CDCl$_3$): δ 159.2, 141.4, 129.0 – 128.7 (m, 2C), 128.6 (2C), 128.6 (2C), 127.4 (q, *J* = 32.2 Hz, 2C), 126.6 (q, *J* = 34.6 Hz), 126.2, 122.8 (q, *J* = 272.5 Hz), 122.4 (q, *J* = 273.6 Hz, 2C), 78.0, 31.9, 31.7 ppm.

^{19}F NMR (376 MHz, CDCl$_3$): δ -60.76 (s, 6F), -62.65 (s, 3F) ppm.

IR (neat) ν_{max}: 2960, 1632, 1489, 1473, 1272, 1206, 1129, 917, 661 cm^{-1}.

HRMS (ESI$^-$): exact mass calculated for $[M-H]^-$ $(C_{18}H_{12}F_9O)^-$ requires *m/z* 415.0750, found *m/z* 415.0737.

Rf: 0.34 100% *n*-heptane

In the absence of lithium iodide in the reaction medium, primary, non-benzylic alcohols do not form the final amine product but are retained at the aryl ether stage. Due to the insufficient electrophilicity of the aryl ether, the S_N2 reaction with the amide is not possible. ^{1}H NMR analysis of the crude reaction mixture indicated the presence of only the free amine and aryl ether. The low yield of the aryl ether may be due to its moderate stability on silica gel during column chromatography.

(3-Iodopropyl)benzene (12)

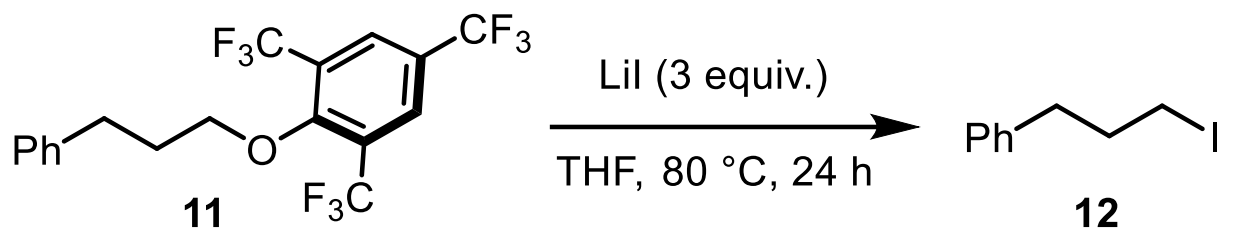

11 12

Under an argon atmosphere, an oven-dried vial was charged with **11** (37.5 mg, 0.09 mmol, 1.00 equiv.), followed by addition of lithium iodide (36.1 mg, 0.27 mmol, 3.00 equiv.) and anhydrous THF (0.9 mL). The vial was tightly sealed and placed into a metal heating-block. The reaction mixture was stirred for 24 h at 80 °C. After that time, the reaction mixture was cooled to room temperature (25 °C) and concentrated *in vacuo*. The resulting crude material was subjected to flash column chromatography (silica gel, 100 % *n*-heptane) to obtain the title compound as a colourless oil (18.9 mg, 0.077 mmol, 85% yield).

^{1}H NMR (400 MHz, $CDCl_3$): δ 7.33 – 7.27 (m, 2H), 7.24 – 7.17 (m, 3H), 3.18 (t, *J* = 6.8 Hz, 2H), 2.73 (t, *J* = 7.3 Hz, 2H), 2.18 – 2.10 (m, 2H) ppm.

All analytical data were in accordance with those reported in literature.[67]

^{1}H NMR analysis of the crude reaction mixture indicated complete conversion of aryl ether to corresponding (3-iodopropyl)benzene (98% NMR yield). The isolated yield is likely attributable to the small scale and the volatility of the product.

4.2 Comparison with other protecting groups

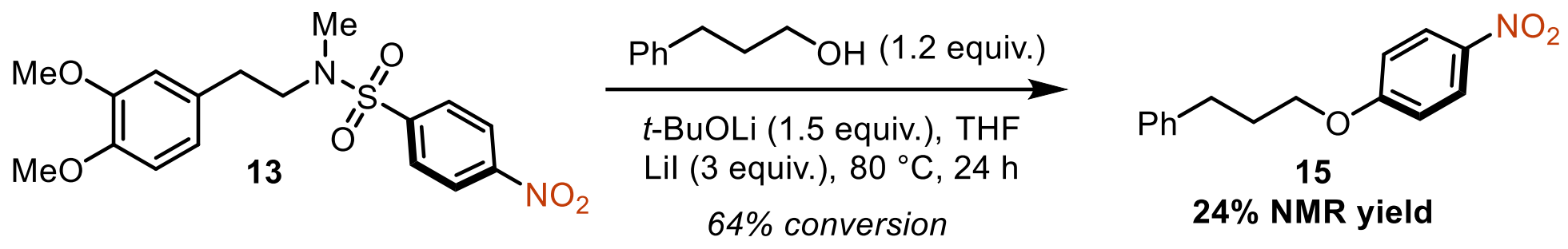

In a glovebox, an oven-dried vial was charged with solid lithium *tert*-butoxide (12.0 mg, 0.15 mmol, 1.50 equiv.), after which it was capped and removed from the glovebox. Then, Ns-amide **13**[34] (38.0 mg, 0.10 mmol, 1.00 equiv.) and lithium iodide (40.2 mg, 0.30 mmol, 3.00 equiv.) were added, followed by addition of 3-phenylpropanol (16.3 mg, 0.12 mmol, 1.20 equiv.) dissolved in THF (1.0 mL). The vial was tightly sealed and placed into a metal heating-block. The reaction mixture was stirred for 24 h at 80 °C. After that time, the reaction mixture was cooled to room temperature (25 °C), quenched with water (3.0 mL) and transferred to separatory funnel. The crude material was diluted with a aqueous solution 1M of NaOH (10 mL) and extracted with DCM (3 x 10 mL). The combined organic phases were dried over anhydrous Na_2SO_4 and concentrated *in vacuo*. The resulting crude material was analysed by ^1H NMR with CH_2Br_2 (0.10 mmol) as an internal standard.

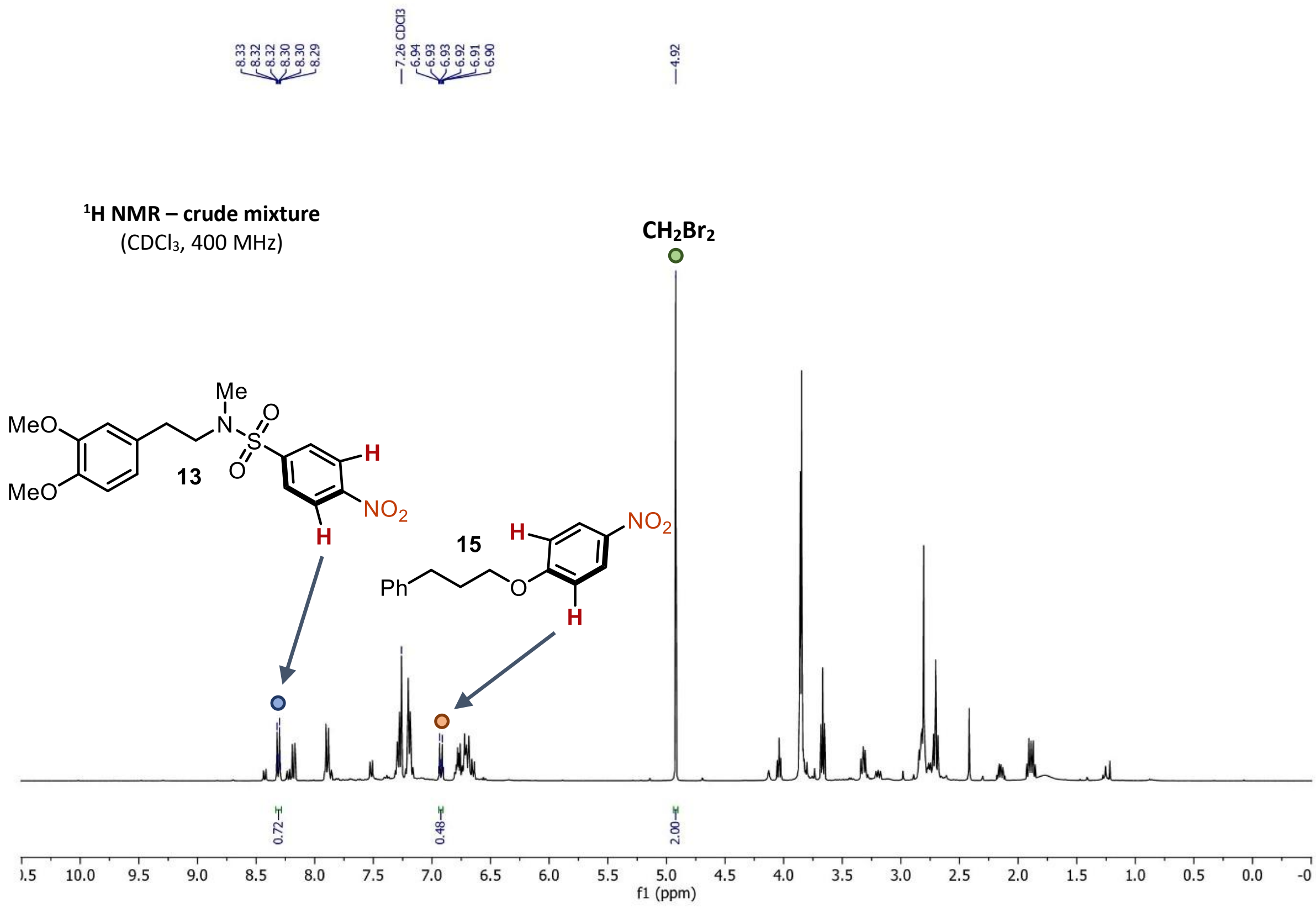

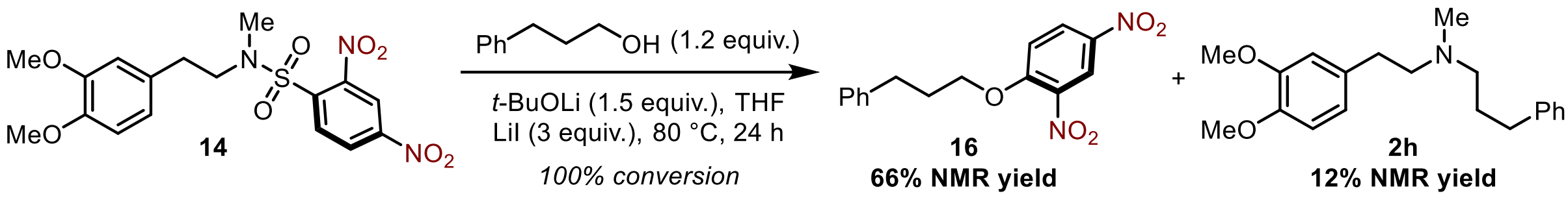

In a glovebox, an oven-dried vial was charged with solid lithium *tert*-butoxide (12.0 mg, 0.15 mmol, 1.50 equiv.), after which is was capped and removed from the glovebox. Then, **14** (42.5 mg, 0.10 mmol, 1.00 equiv.) and lithium iodide (40.2 mg, 0.30 mmol, 3.00 equiv.) were added, followed by addition of 3-phenylpropanol (16.3 mg, 0.12 mmol, 1.20 equiv.) dissolved in THF (1.0 mL). The vial was tightly sealed and placed into a metal heating-block. The reaction mixture was stirred for 24 h at 80 °C. After that time, the reaction mixture was cooled to room temperature (25 °C), quenched with water (3.0 mL) and transferred to separatory funnel. The crude material was diluted with a aqueous solution 1M of NaOH (10 mL) and extracted with DCM (3 x 10 mL). The combined organic phases were dried over anhydrous Na_2SO_4 and concentrated *in vacuo*. The resulting crude material was analysed by ^{1}H NMR with CH_2Br_2 (0.10 mmol) as an internal standard.

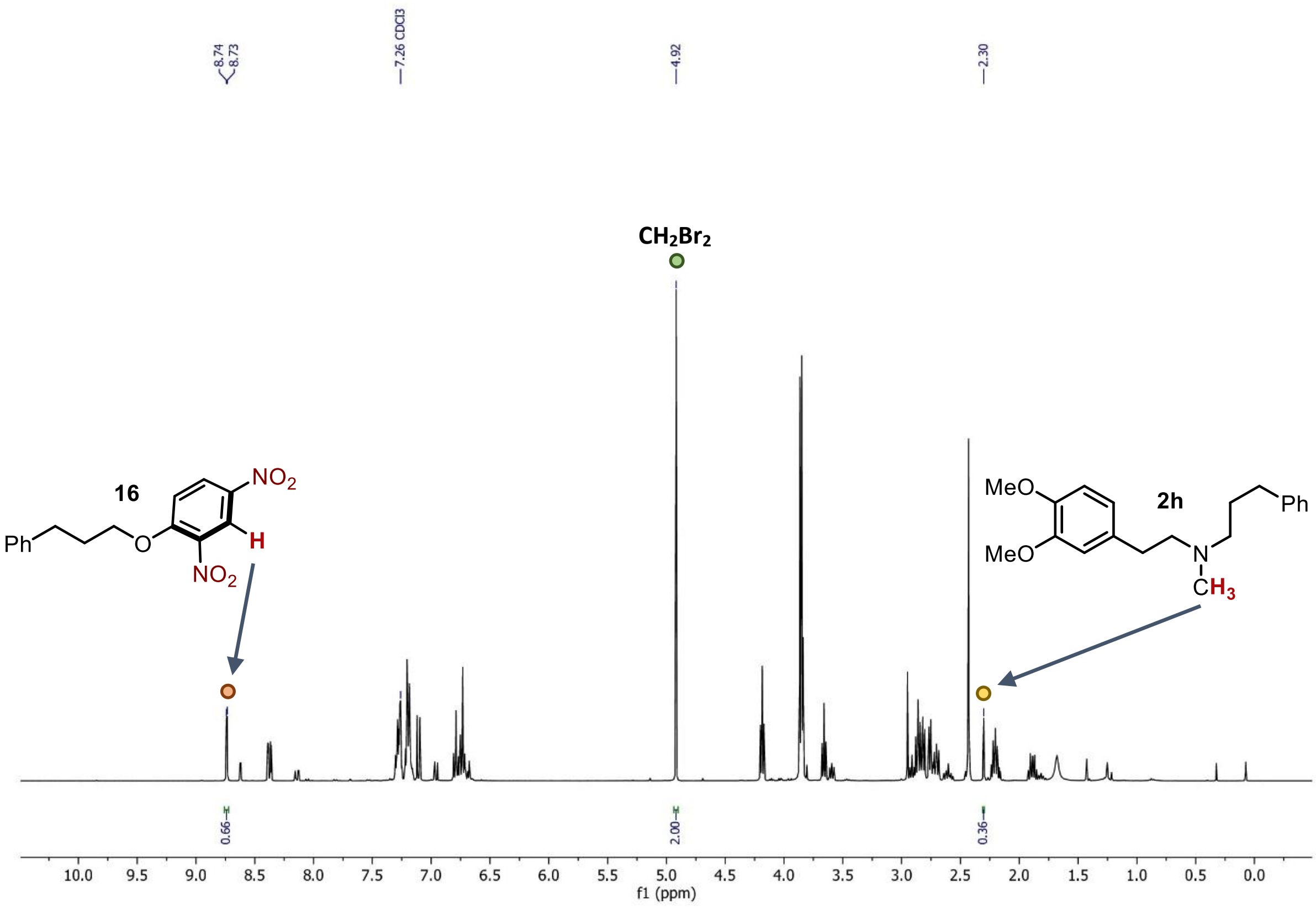

4.3 DFT calculations

The conformational space of all molecules was initially explored using metadynamics simulations based on tight-binding quantum chemical calculations as implemented in the software package Conformer-Rotamer Ensemble Sampling Tool (CREST).[68-70]

The structures located with CREST were subsequently subjected to geometry optimization at the B3LYP-D3(BJ)/def2-SVP.[44-49] The nature of all stationary points (minima and transition states) was verified through the computation of harmonic vibrational frequencies. Thermal corrections to the Gibbs free energies were combined with the single point energies calculated at the B3LYP-D3(BJ)/def2-TZVP level of theory to yield Gibbs free energies ("*G*"). Final free energies were obtained using GoodVibes[71,72] by applying Grimme's quasi-harmonic (QRRHO) correction and conversion to a 1 M standard state at 353.15 K

Solvent (THF) effects were considered using the implicit solvation model based on density (SMD)[73,74] with refined parameters for iodine (SMD18).[75]

All DFT calculations were performed using the Gaussian 16 program package.[76] All energies are reported in kcal mol^{-1}.

Cartesian coordinates of the most stable conformations, computed at the B3LYP-D3(BJ),SMD-18(THF)/def2-TZVP//B3LYP-D3(BJ)/def2-SVP level of theory

NMS_ArOMe

C	-1.08781	0.00010	-0.05287
C	-0.37110	1.21453	-0.03900
C	1.02203	1.20711	0.03218
C	1.71860	-0.00023	0.08151
C	1.02176	-1.20739	0.03218
C	-0.37140	-1.21447	-0.03900
H	1.56402	2.15192	0.04598
H	1.56351	-2.15232	0.04597
C	-1.10322	-2.53396	-0.16630
C	-1.10261	2.53417	-0.16631
C	3.22646	-0.00032	0.10991
O	-2.43560	0.00025	-0.15566
C	-3.21308	0.00037	1.04968
H	-4.26228	0.00047	0.72981
H	-3.01038	-0.90105	1.64450
H	-3.01018	0.90175	1.64447
F	-1.93005	2.75908	0.87446
F	-1.83916	2.58668	-1.28125
F	-0.24079	3.56452	-0.20398
F	-1.83972	-2.58636	-1.28128
F	-1.93080	-2.75862	0.87441
F	-0.24168	-3.56455	-0.20386
F	3.70741	-1.08640	0.73643
F	3.74089	0.00068	-1.13279
F	3.70748	1.08472	0.73818

NMS_TS

C	-0.61215	0.00003	0.88263
C	-1.28102	1.21706	0.51200
C	-2.48688	1.20660	-0.17677
C	-3.09748	-0.00002	-0.53754
C	-2.48685	-1.20661	-0.17675
C	-1.28099	-1.21704	0.51202
H	-2.96014	2.15227	-0.44069

H	-2.96009	-2.15230	-0.44065
C	-0.67112	-2.52772	0.92610
C	-0.67118	2.52777	0.92605
C	-4.42411	-0.00005	-1.22448
O	0.50322	0.00005	1.52256
C	2.12344	0.00002	0.53874
H	2.71917	-0.00017	1.44346
H	1.94576	-0.93893	0.02942
F	0.54605	2.73731	0.38553
F	-0.53146	2.63232	2.25843
F	-1.43792	3.57719	0.53939
F	-0.53140	-2.63223	2.25849
F	0.54611	-2.73724	0.38559
F	-1.43784	-3.57716	0.53947
F	-4.59604	-1.08651	-2.00678
F	-5.46481	-0.00020	-0.35436
F	-4.59620	1.08653	-2.00659
H	1.94592	0.93915	0.02969
I	4.56878	-0.00001	-0.82804

NS_ArOMe

C	1.63140	0.28616	-0.00014
C	1.05342	-0.99652	-0.00001
C	-0.33399	-1.13016	-0.00001
C	-1.13461	0.00985	-0.00005
C	-0.57528	1.29305	-0.00018
C	0.80391	1.42753	-0.00026
H	-0.80904	-2.11047	0.00011
H	-1.23520	2.15964	-0.00026
O	2.95764	0.51940	0.00003
C	3.85590	-0.57161	0.00020
H	4.86570	-0.14217	0.00068
H	3.73041	-1.20036	0.89888
H	3.73106	-1.20003	-0.89875
H	1.27889	2.40969	-0.00040
H	1.67322	-1.89197	0.00011
N	-2.59451	-0.13823	0.00006
O	-3.26707	0.88212	0.00052
O	-3.05032	-1.27233	-0.00031

NS_TS

C	-1.25194	1.16290	-0.00001
C	-2.49763	1.89155	0.00000
C	-3.71987	1.25574	0.00001
C	-3.78101	-0.15238	0.00000
C	-2.59295	-0.90515	-0.00001
C	-1.36408	-0.27377	-0.00001
H	-4.65543	1.81542	0.00001
H	-2.66973	-1.99266	-0.00001
O	-0.14561	1.80066	-0.00001
C	1.64665	0.80451	-0.00000
H	2.09042	1.79114	0.00000
H	1.39652	0.32710	0.93866
H	1.39653	0.32711	-0.93868
I	4.00932	-0.34620	0.00000
H	-2.42884	2.98189	0.00001
H	-0.45388	-0.87508	-0.00002
N	-5.05431	-0.81620	0.00000
O	-5.07866	-2.04982	-0.00000
O	-6.07880	-0.12754	0.00001

DNS_ArOMe

C	1.18135	-0.73360	-0.01976
C	0.26275	-1.80375	-0.01173
C	-1.10772	-1.57083	0.01364
C	-1.58264	-0.25967	0.01599
C	-0.71071	0.82420	-0.00277
C	0.65611	0.58470	-0.00086

H	-1.82235	-2.39308	0.02537
H	-1.08899	1.84447	-0.01797
O	2.50176	-0.89084	-0.08002
C	3.06348	-2.19145	-0.12001
H	4.14996	-2.04924	-0.15933
H	2.80431	-2.76708	0.78412
H	2.73380	-2.74445	-1.01564
H	0.62509	-2.83008	-0.02931
N	-3.03235	-0.01415	0.03365
O	-3.40563	1.14693	0.04151
O	-3.76547	-0.99035	0.03937
N	1.54146	1.76306	0.02662
O	2.59073	1.67021	0.63501
O	1.13595	2.76400	-0.54288

DNS_TS

C	0.93932	0.58483	-0.06124
C	2.29325	1.10855	-0.01094
C	3.41322	0.29055	0.03058
C	3.26308	-1.09238	-0.01772
C	1.97655	-1.66447	-0.08821
C	0.86226	-0.85822	-0.10453
H	4.40341	0.73537	0.09662
H	1.88990	-2.75019	-0.12607
O	-0.08058	1.33732	-0.03653
C	-1.89179	0.58590	-0.00948
H	-2.28562	1.59297	0.04369
H	-1.78501	0.10546	-0.97384
H	-1.72600	0.02650	0.90265
I	-4.42579	-0.38358	0.03349
H	-0.12582	-1.31511	-0.15151
N	4.43372	-1.94186	0.00207
O	4.26466	-3.15912	-0.03718
O	5.54005	-1.41071	0.05726
N	2.54037	2.54889	0.00506
O	1.71316	3.28975	-0.49955
O	3.59297	2.93740	0.510

5. NMR spectra

***N*-(2-(((*tert*-Butyldimethylsilyl)oxy)methyl)benzyl)-*N*-(3,4-dimethoxyphenethyl)-2,4,6-tris(trifluoromethyl)benzenesulfonamide (S2)**

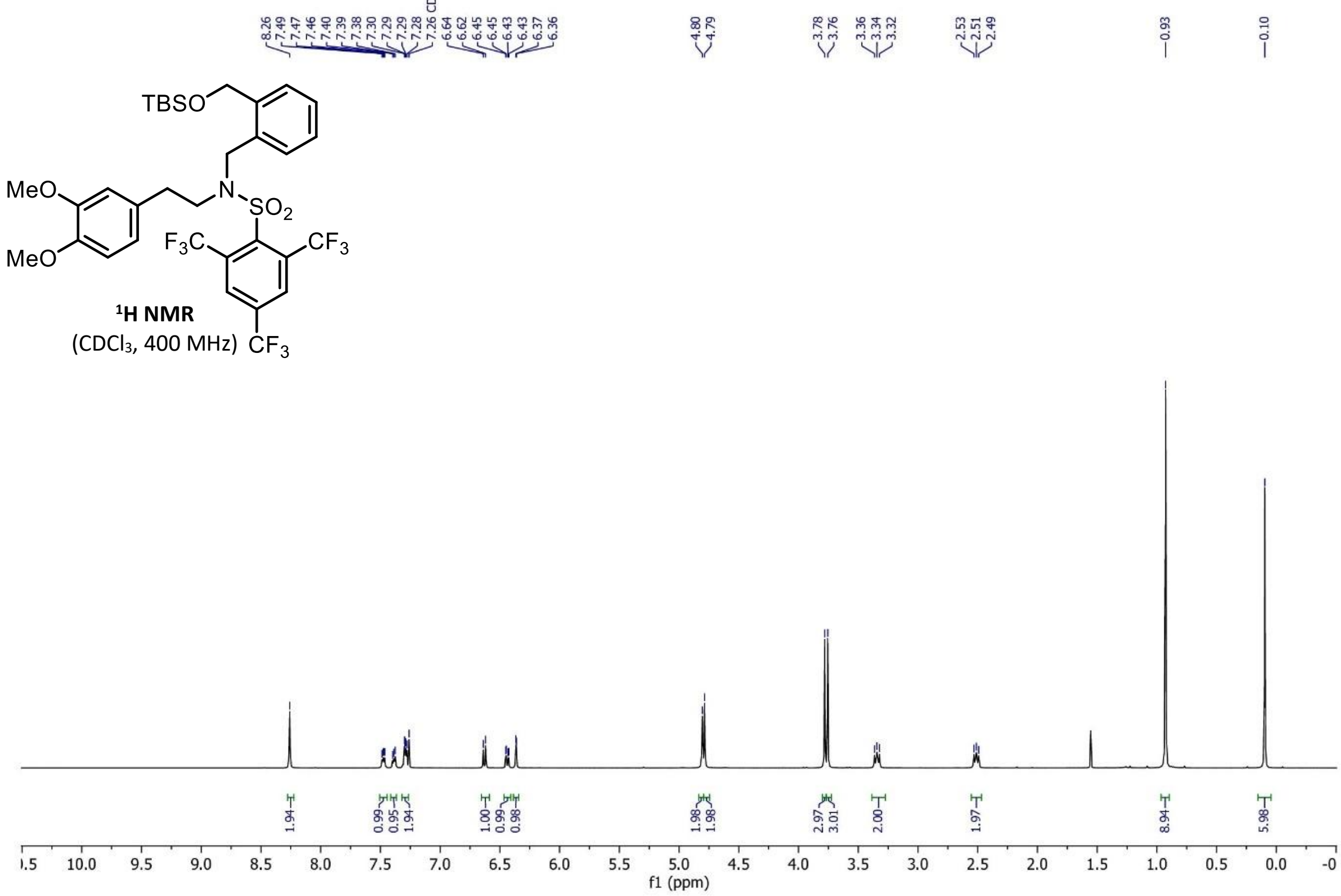

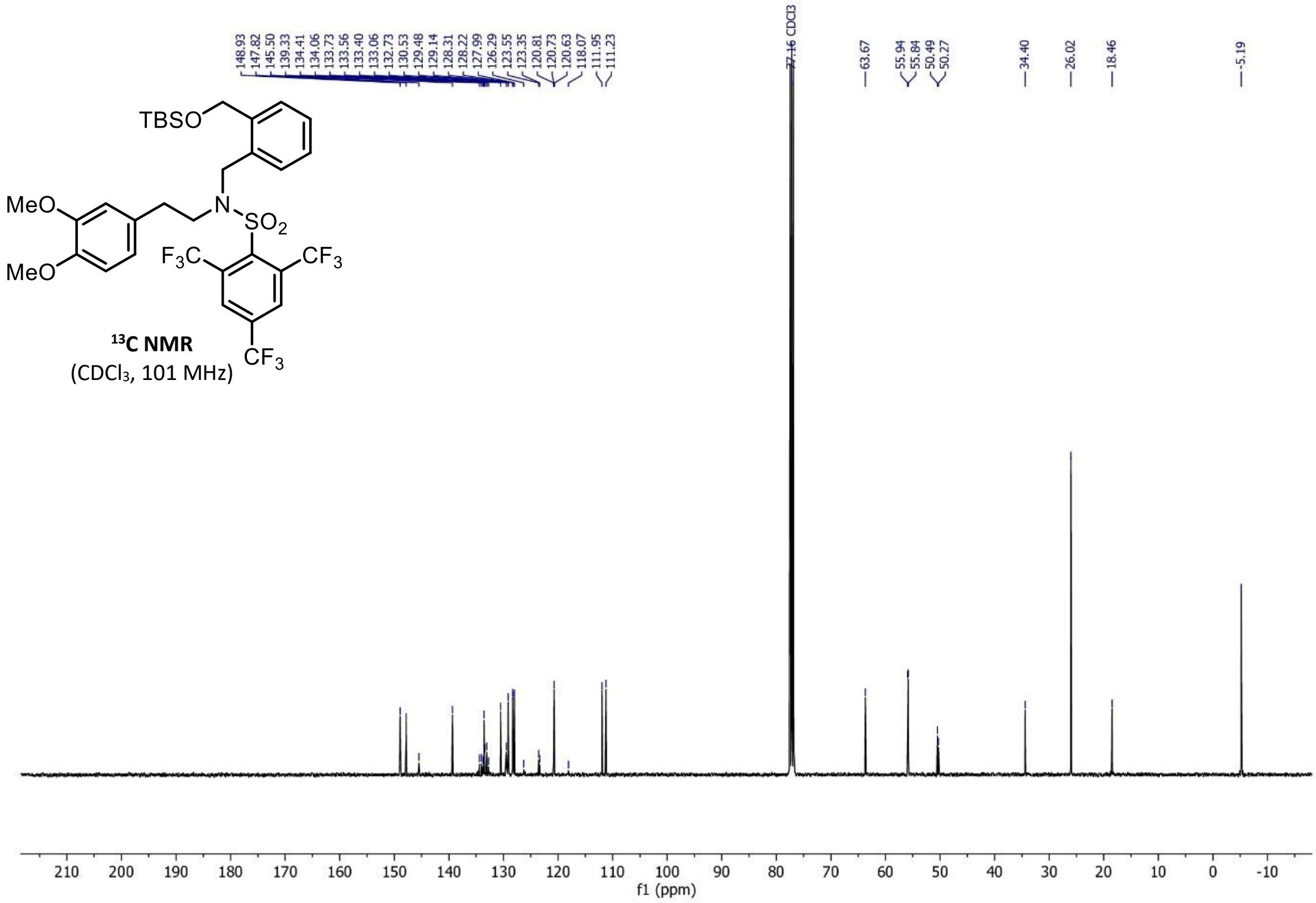

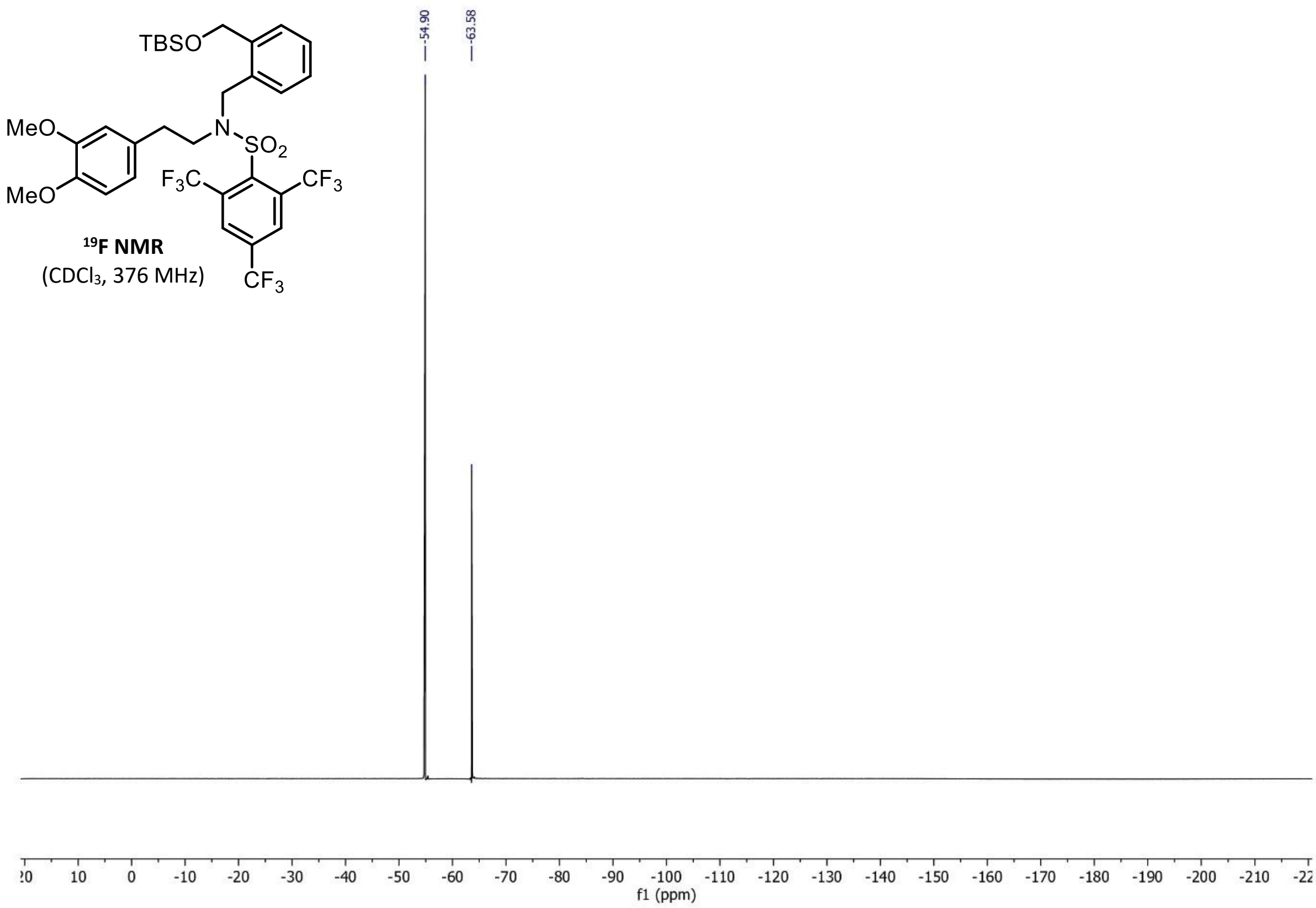

TBSO
MeO
MeO
N
SO2
F3C
CF3
CF3
19F NMR
(CDCl3, 376 MHz)
-54.90
-63.58
f1 (ppm)

***N*-(3,4-Dimethoxyphenethyl)-*N*-(2-(hydroxymethyl)benzyl)-2,4,6-tris(trifluoromethyl)benzenesulfonamide (S3)**

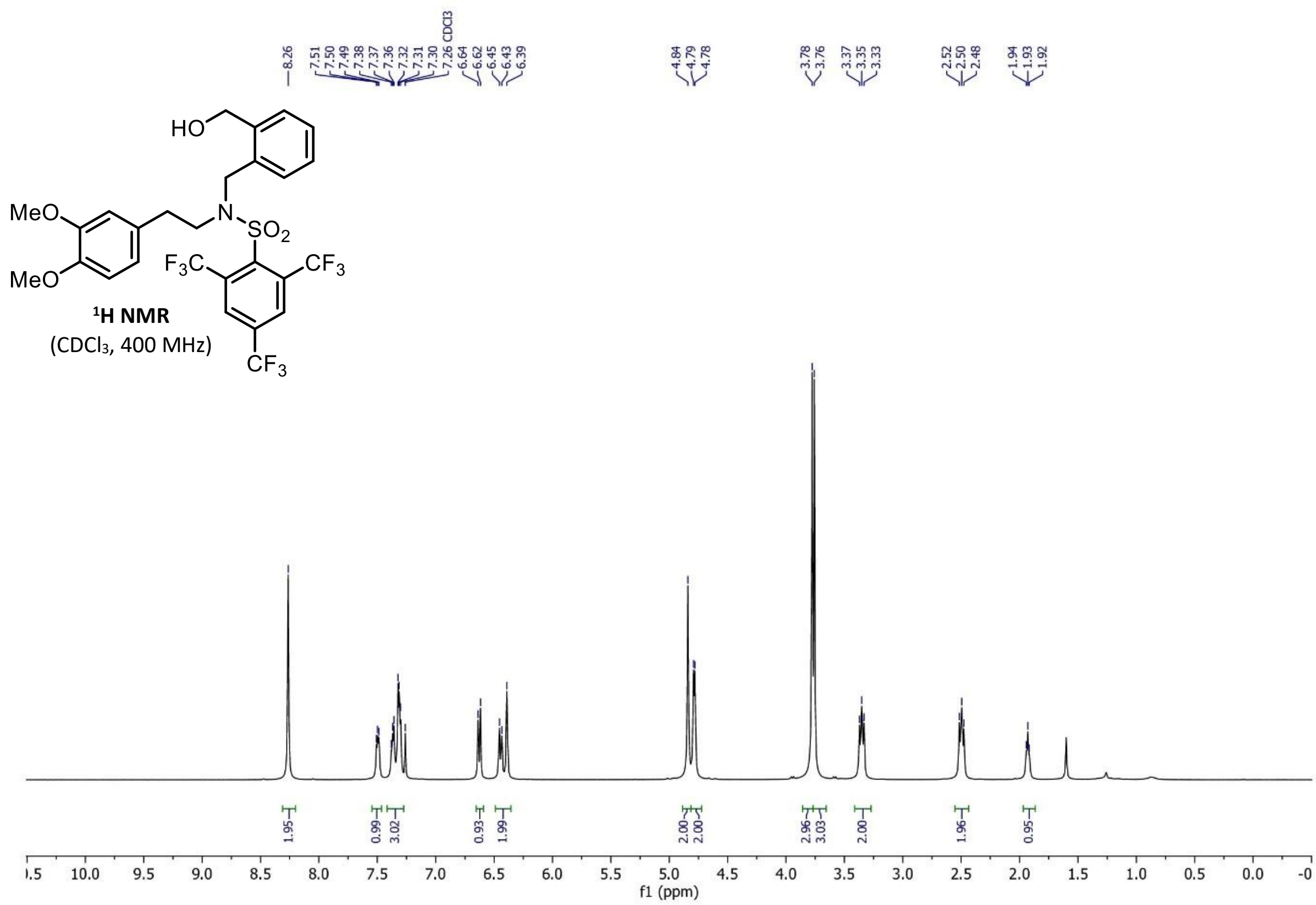

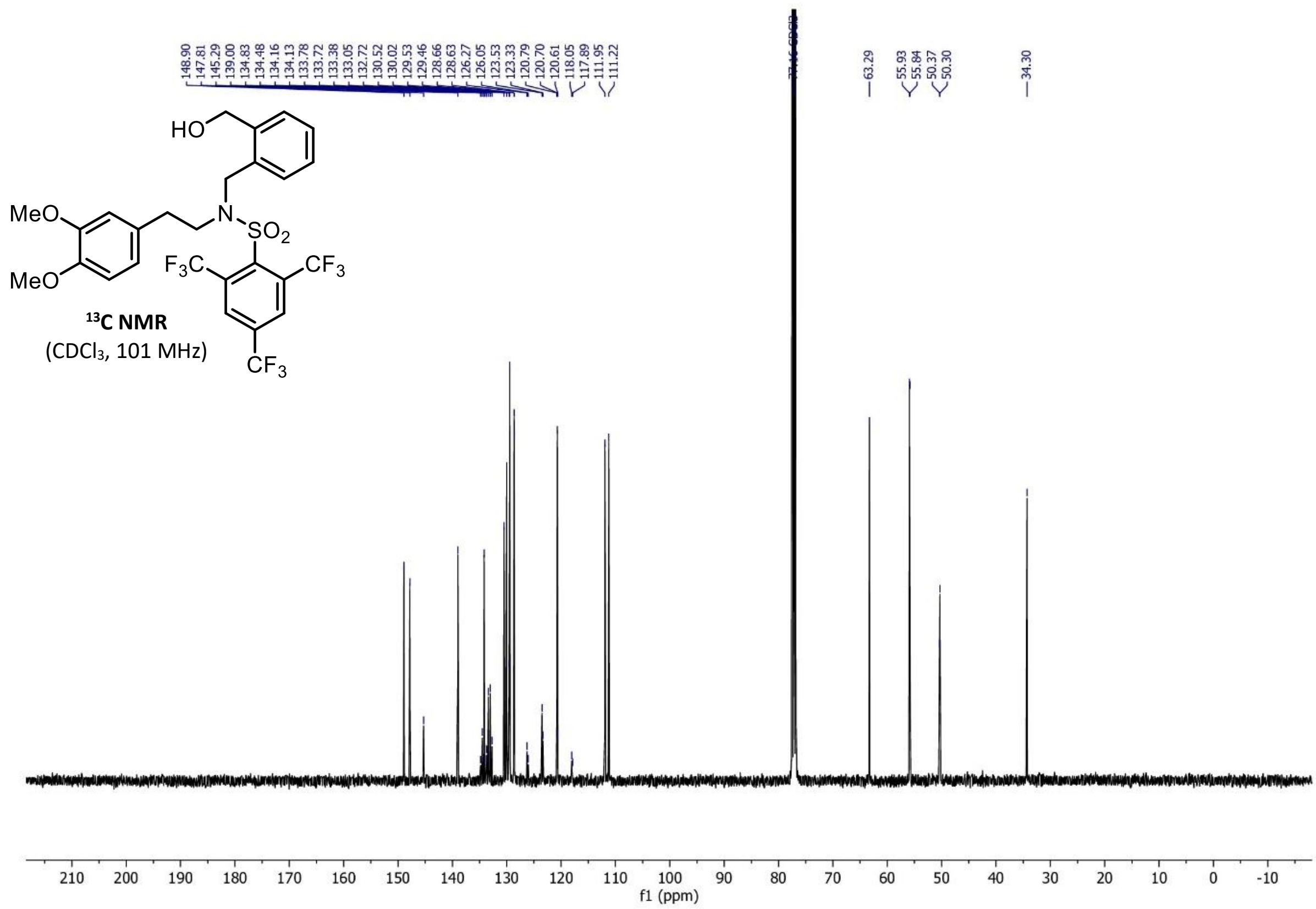

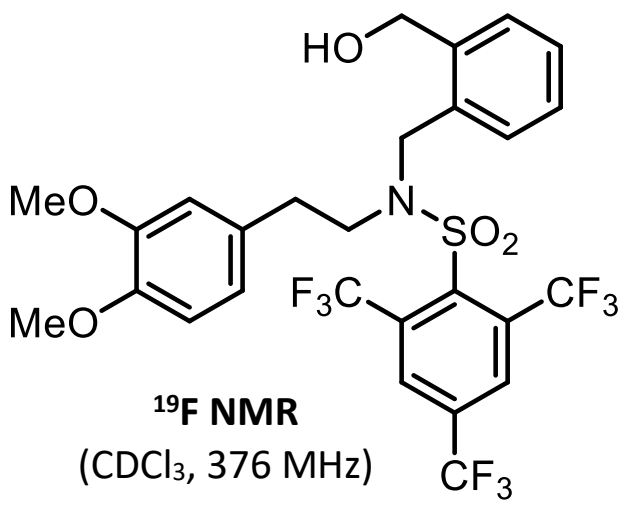

19F NMR
($CDCl_3$, 376 MHz)

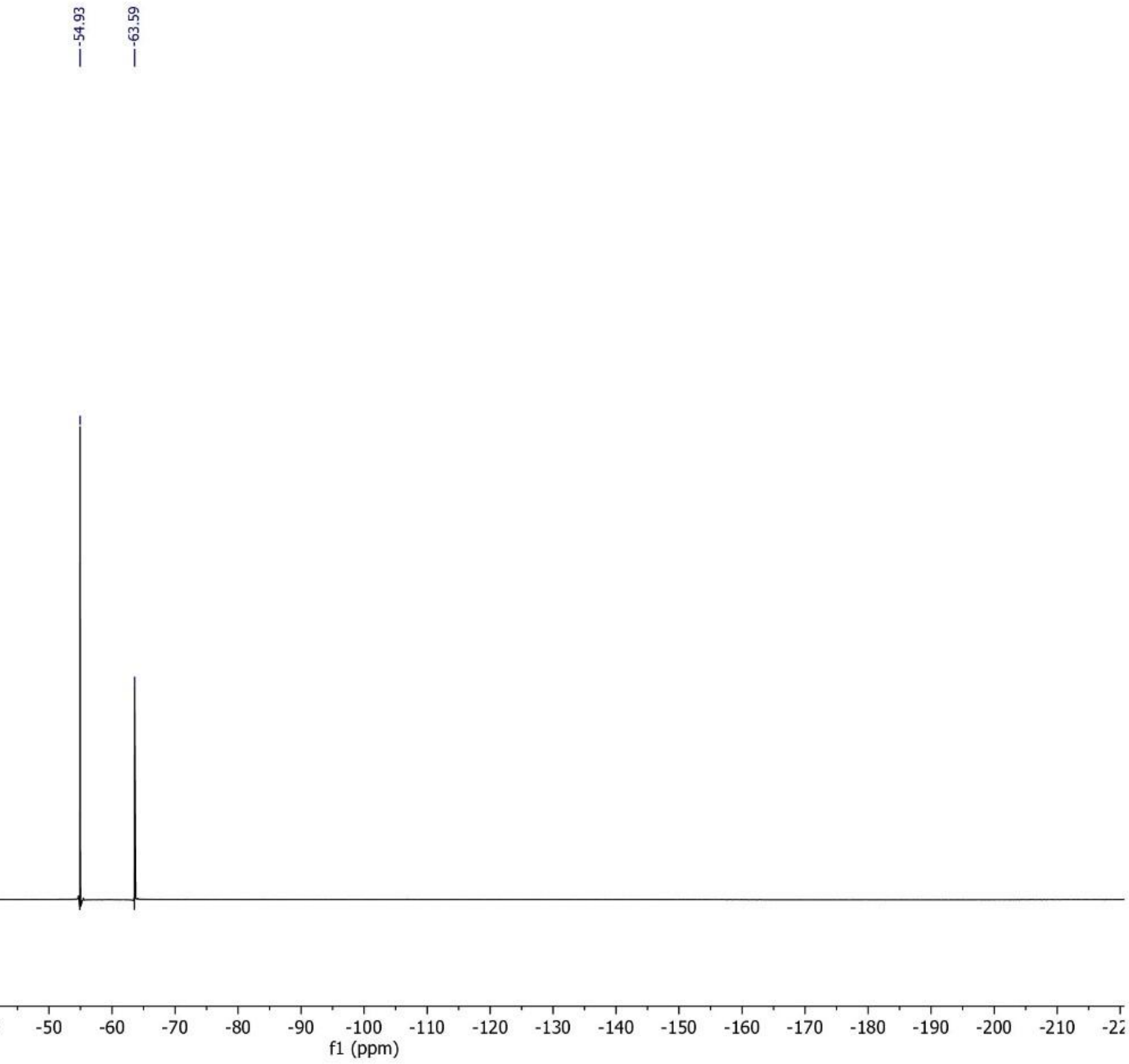

(*E*)-2-(5-Fluoro-2-methyl-1-(4-(methylsulfinyl)benzylidene)-1*H*-inden-3-yl)ethan-1-ol (S4)

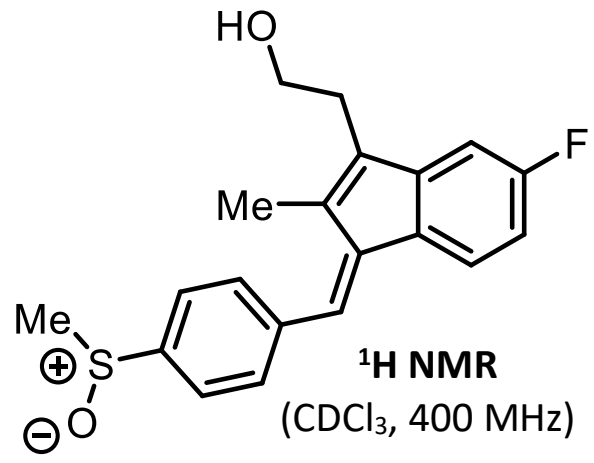

^{1}H NMR
(CDCl₃, 400 MHz)

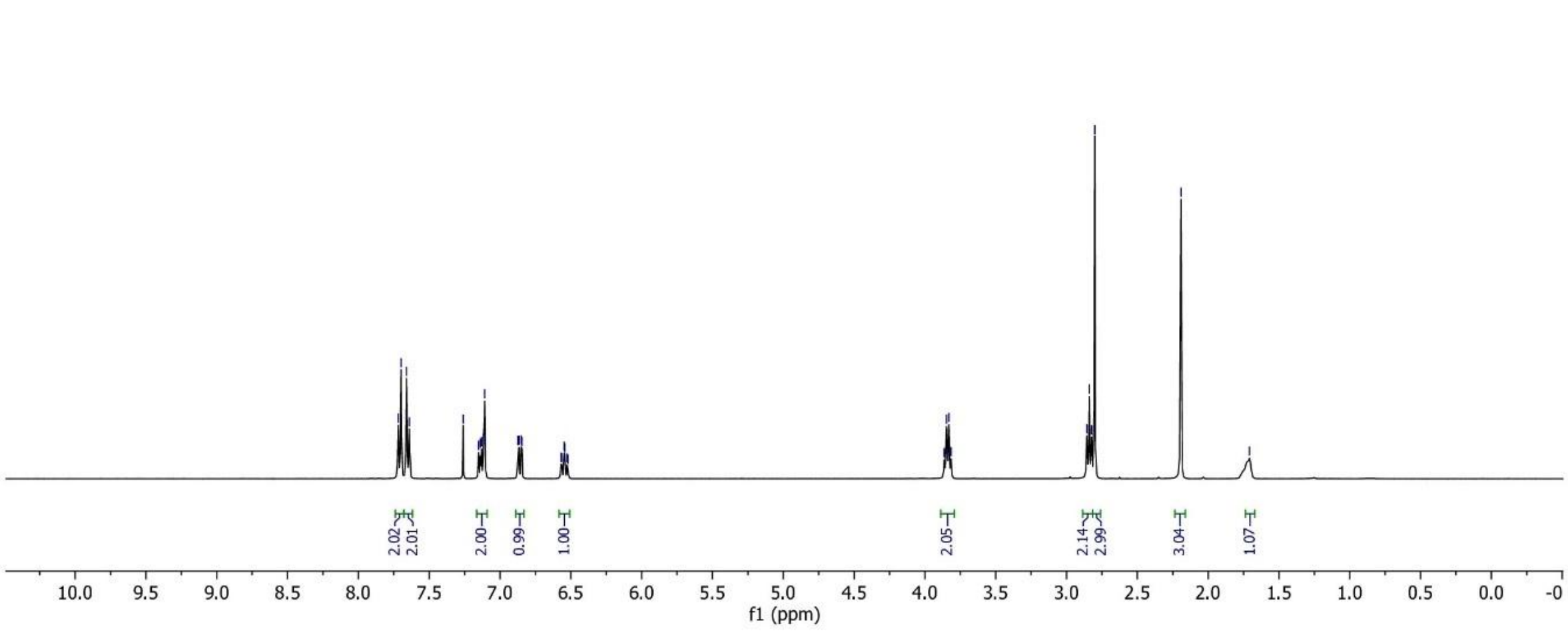

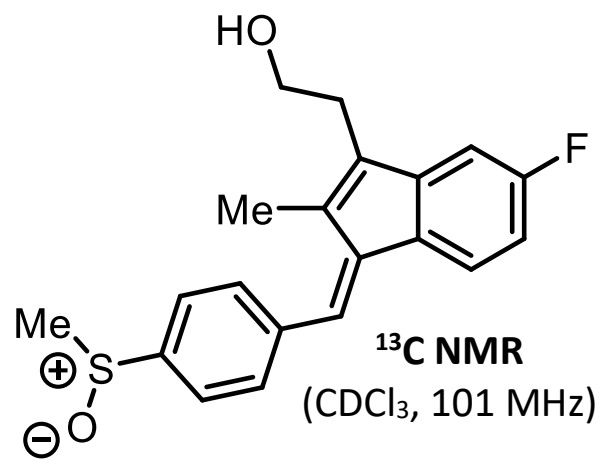

^{13}C NMR
(CDCl₃, 101 MHz)

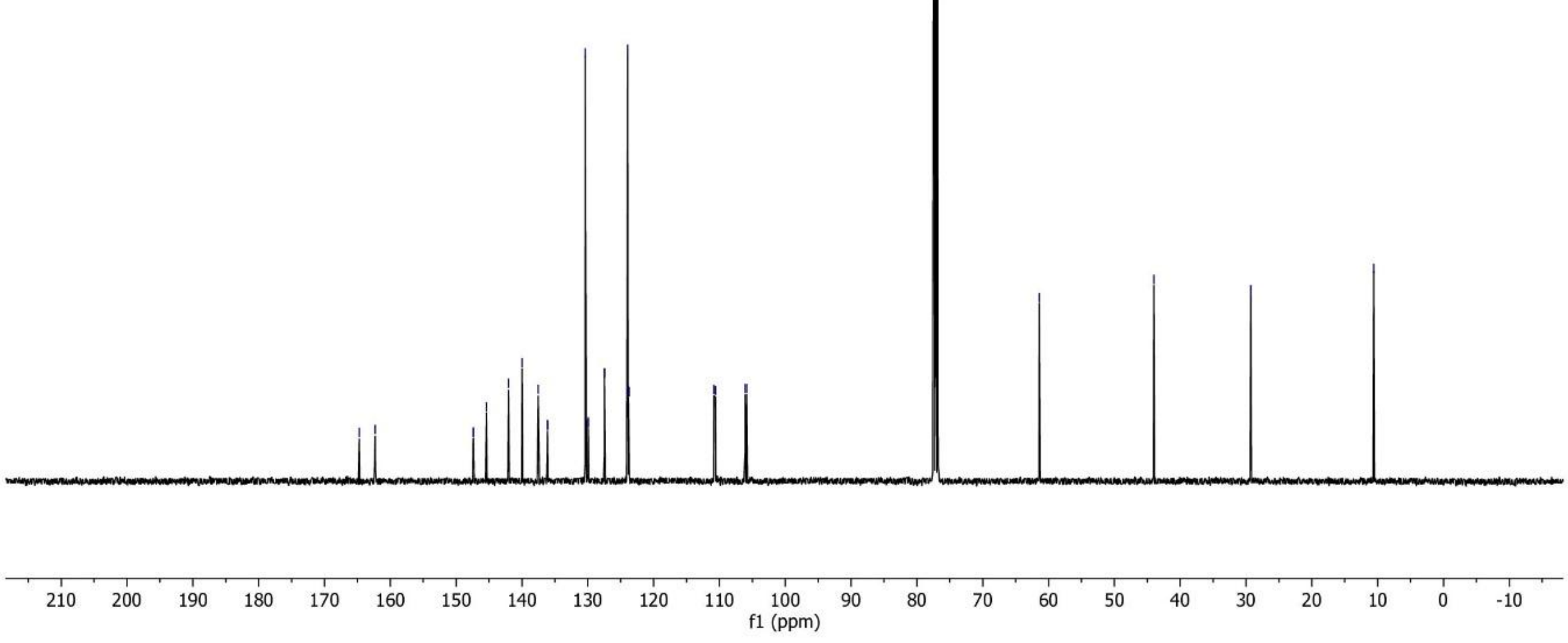

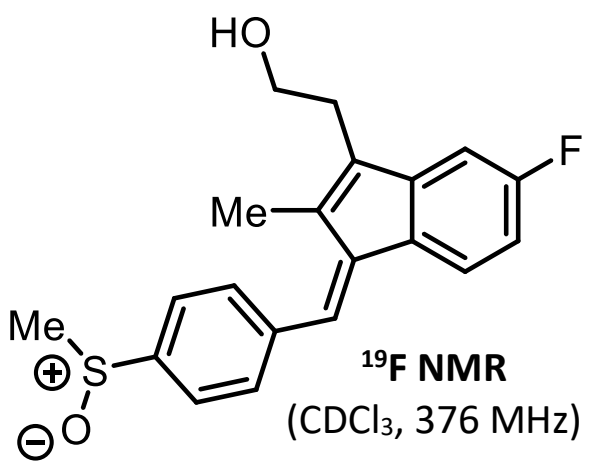

^{19}F NMR
($CDCl_3$, 376 MHz)

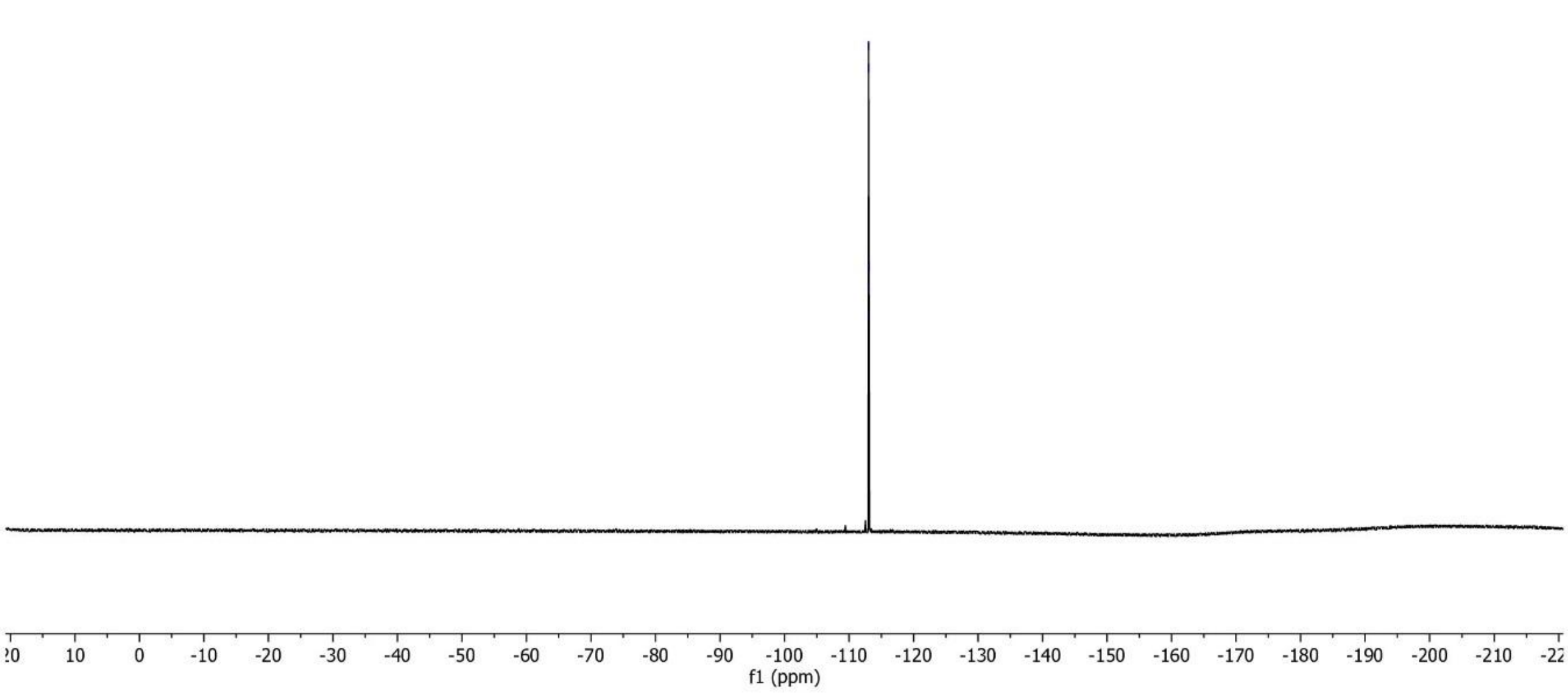

5-(5-(Hydroxymethyl)-4-methylthiazol-2-yl)-2-isobutoxybenzonitrile (S5)

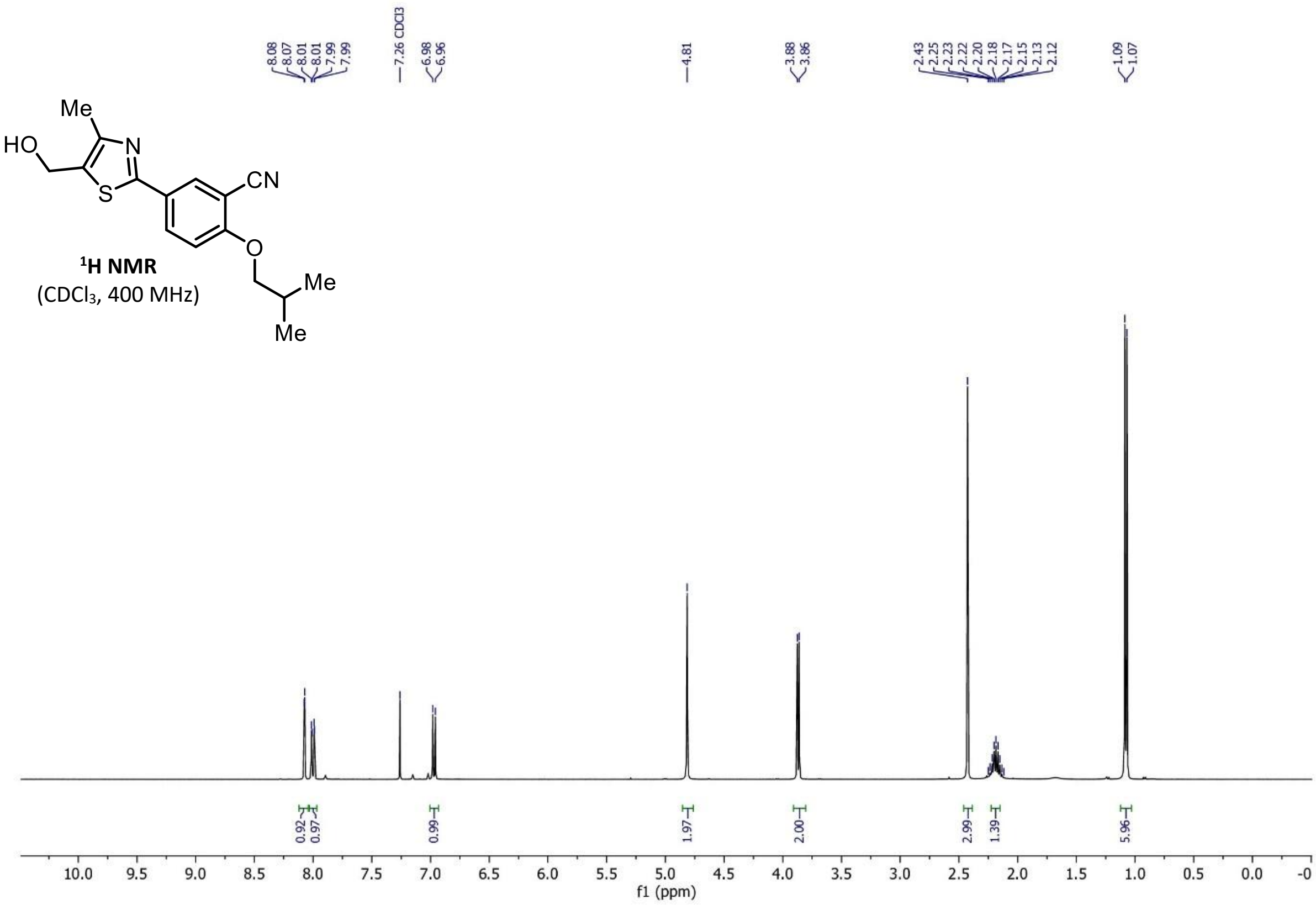

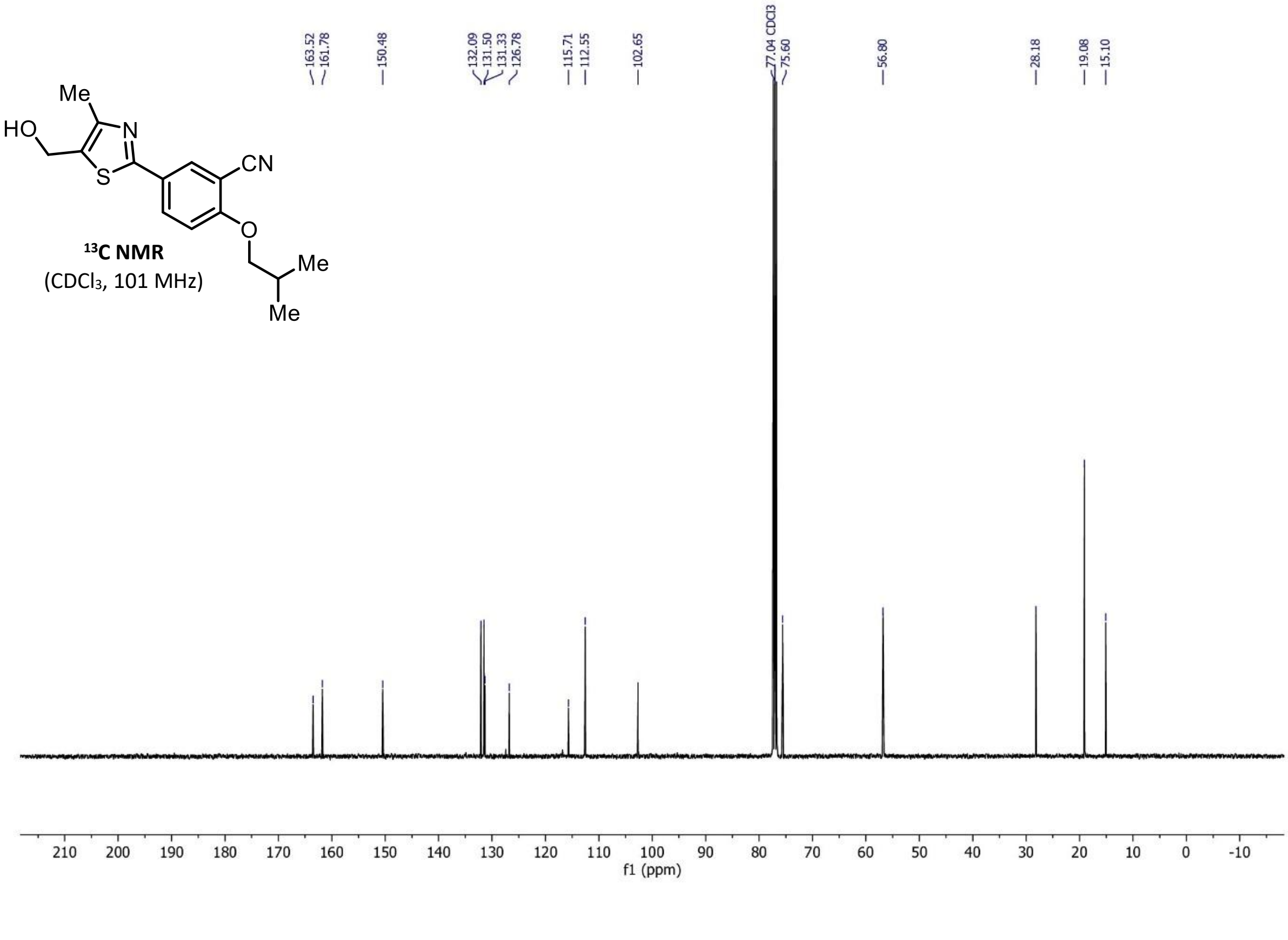

(4-(5-(2-Fluorophenyl)-1,2,4-oxadiazol-3-yl)phenyl)methanol (S6)

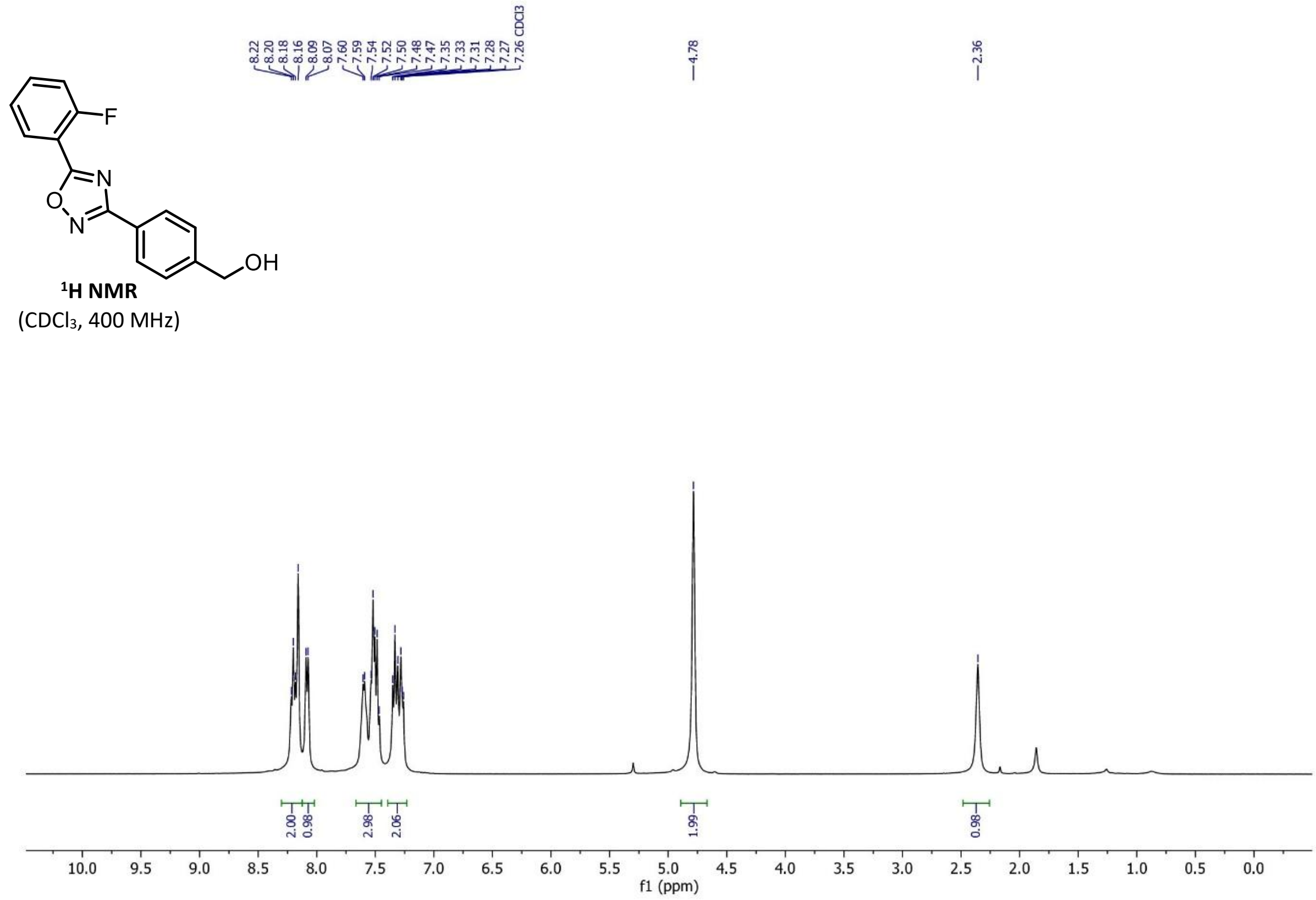

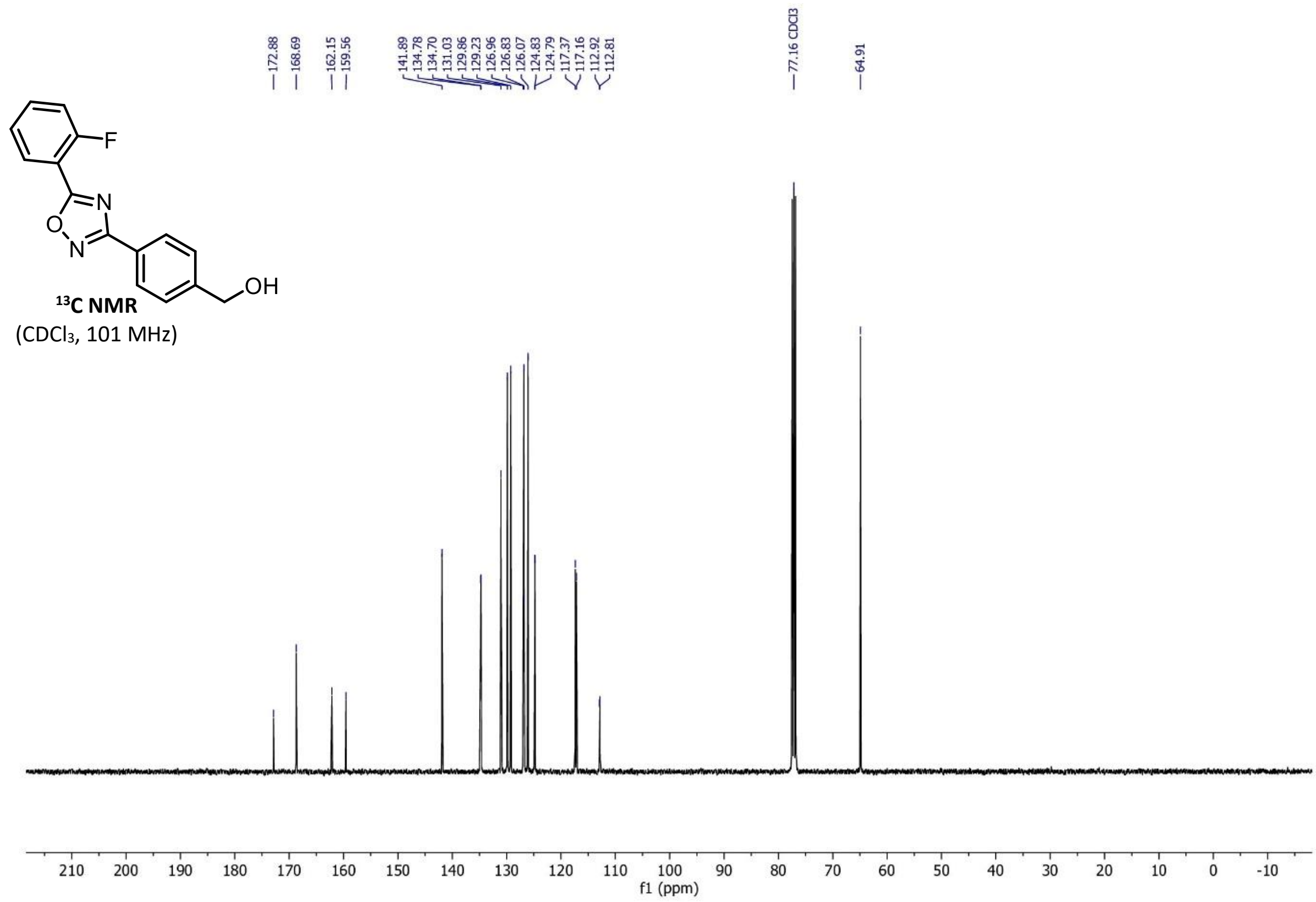

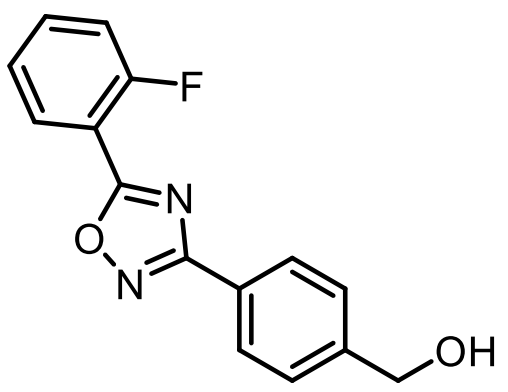

^{19}F NMR
($CDCl_3$, 376 MHz)

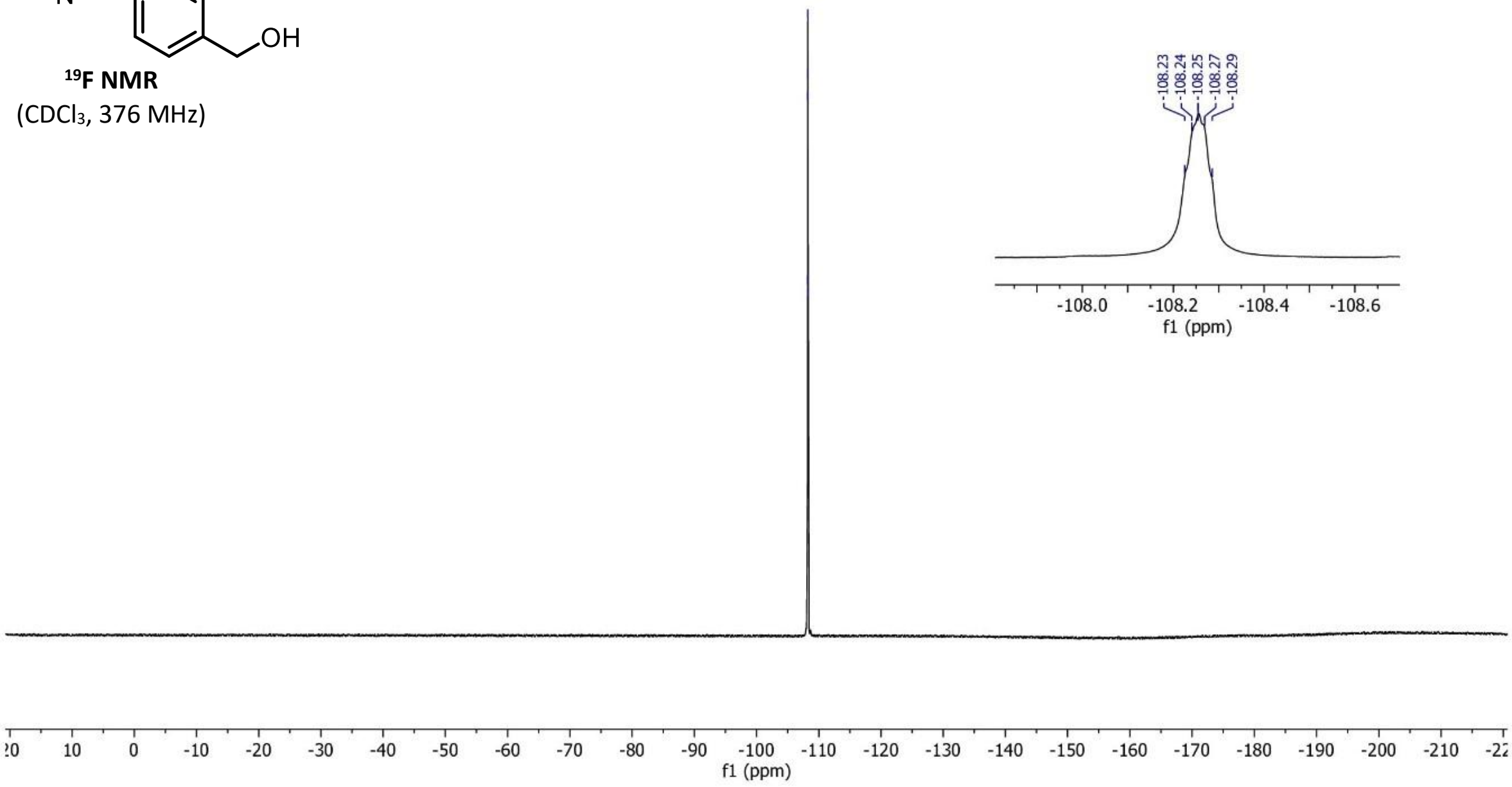

(*R*)-4-(1-Hydroxyethyl)-*N*,*N*-dipropylbenzenesulfonamide (S7)

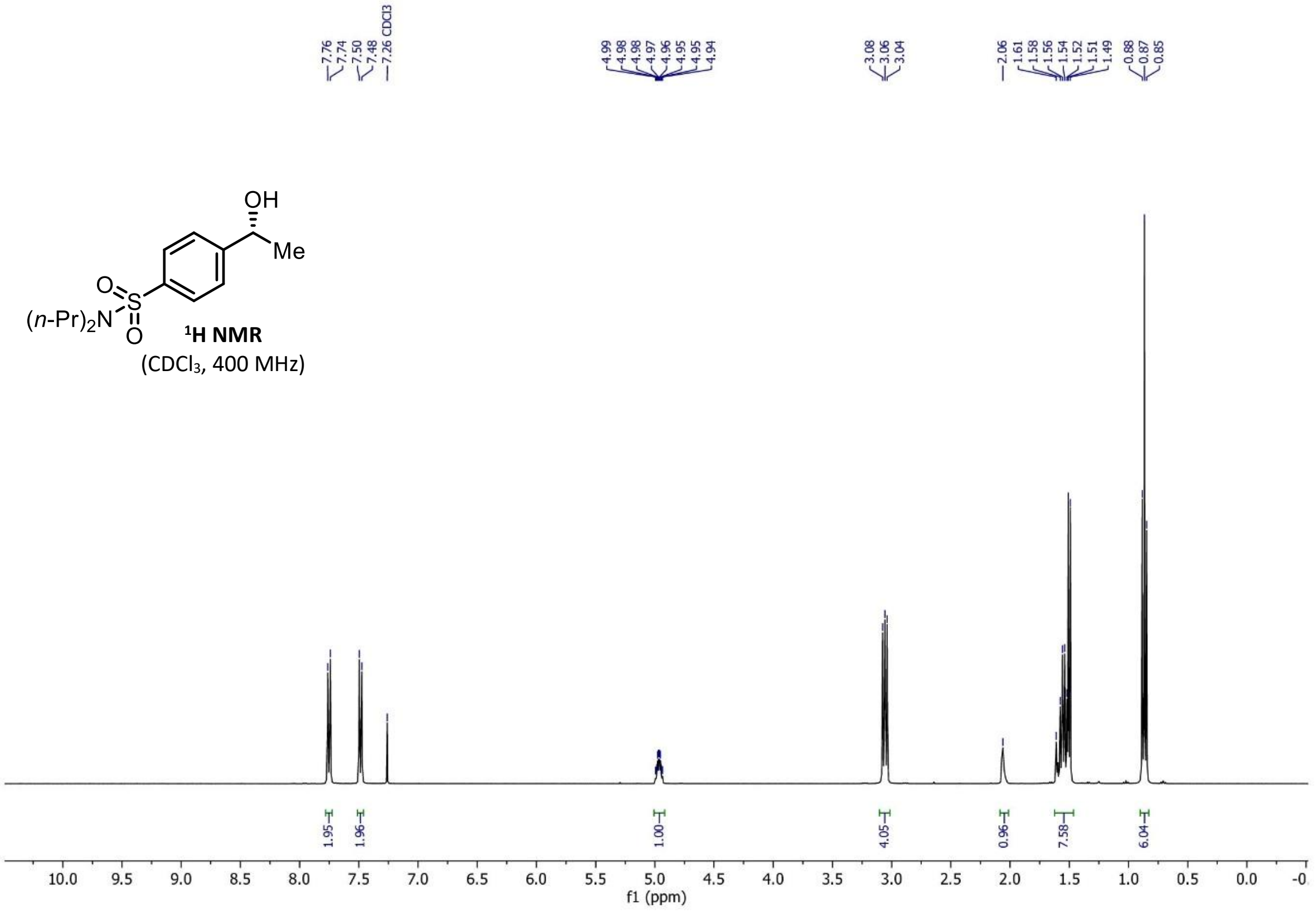

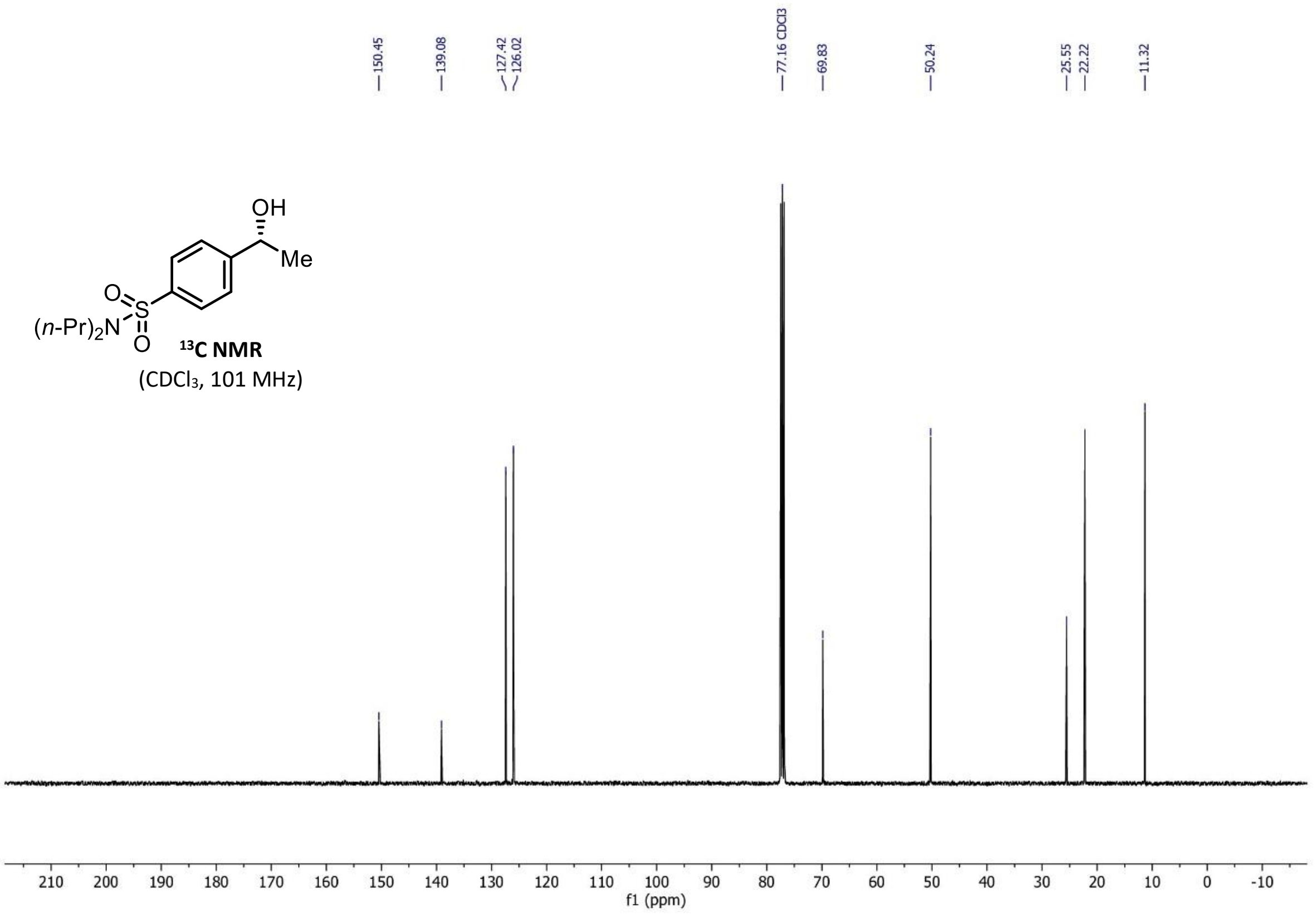

1-(3-((*tert*-Butyldimethylsilyl)oxy)propyl)-1-(4-fluorophenyl)-1,3-dihydroisobenzofuran-5-carbonitrile (S12)

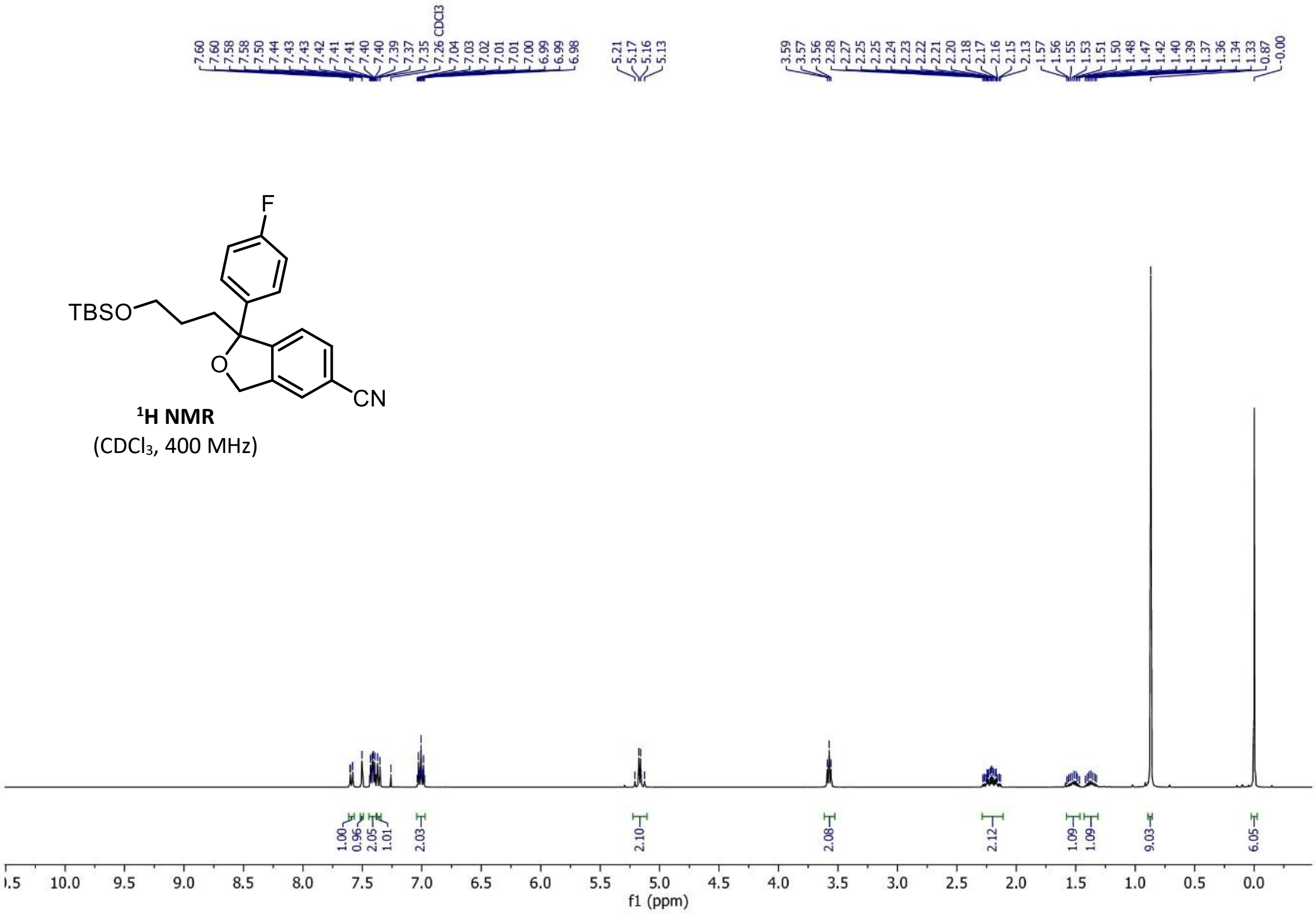

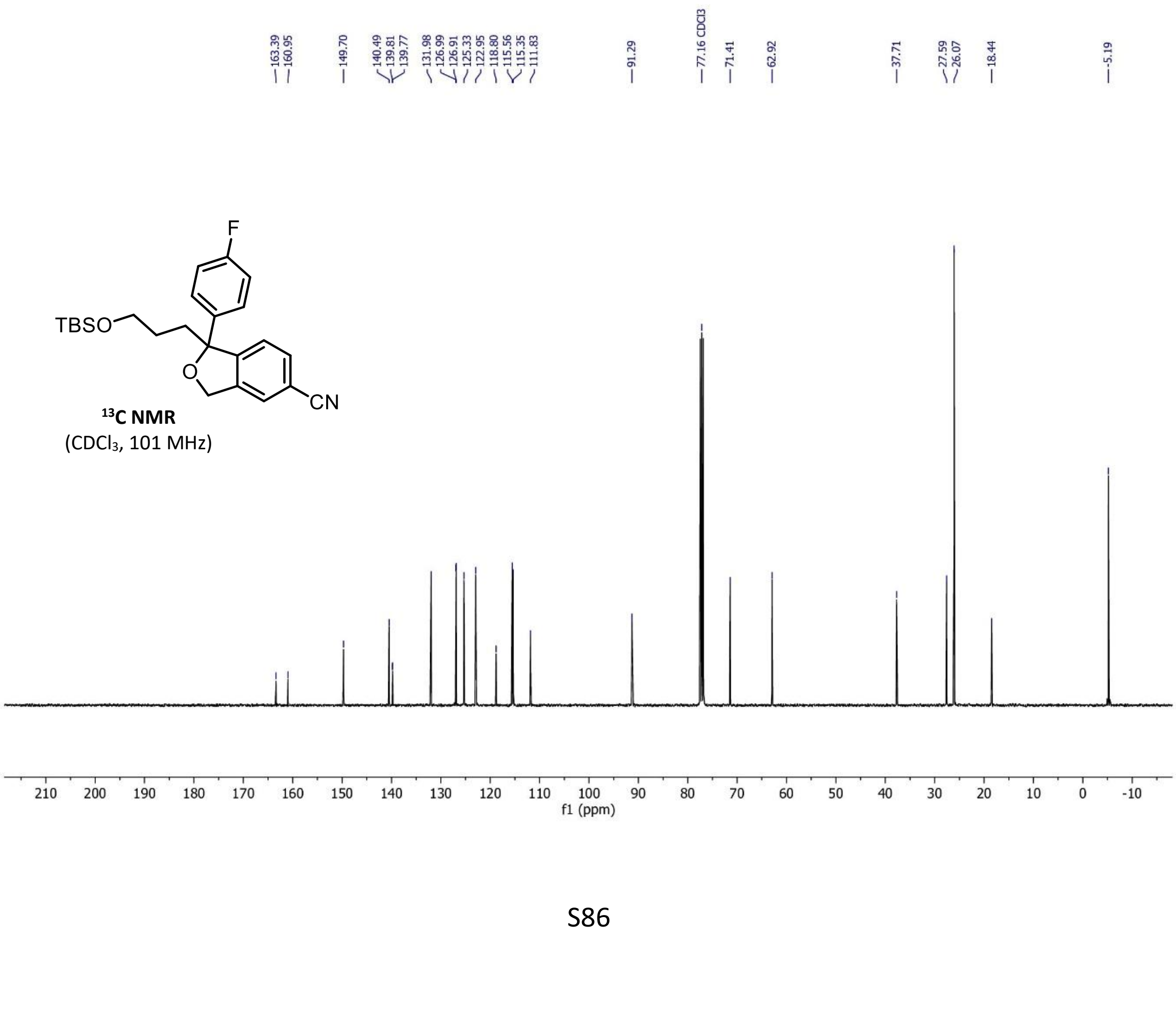

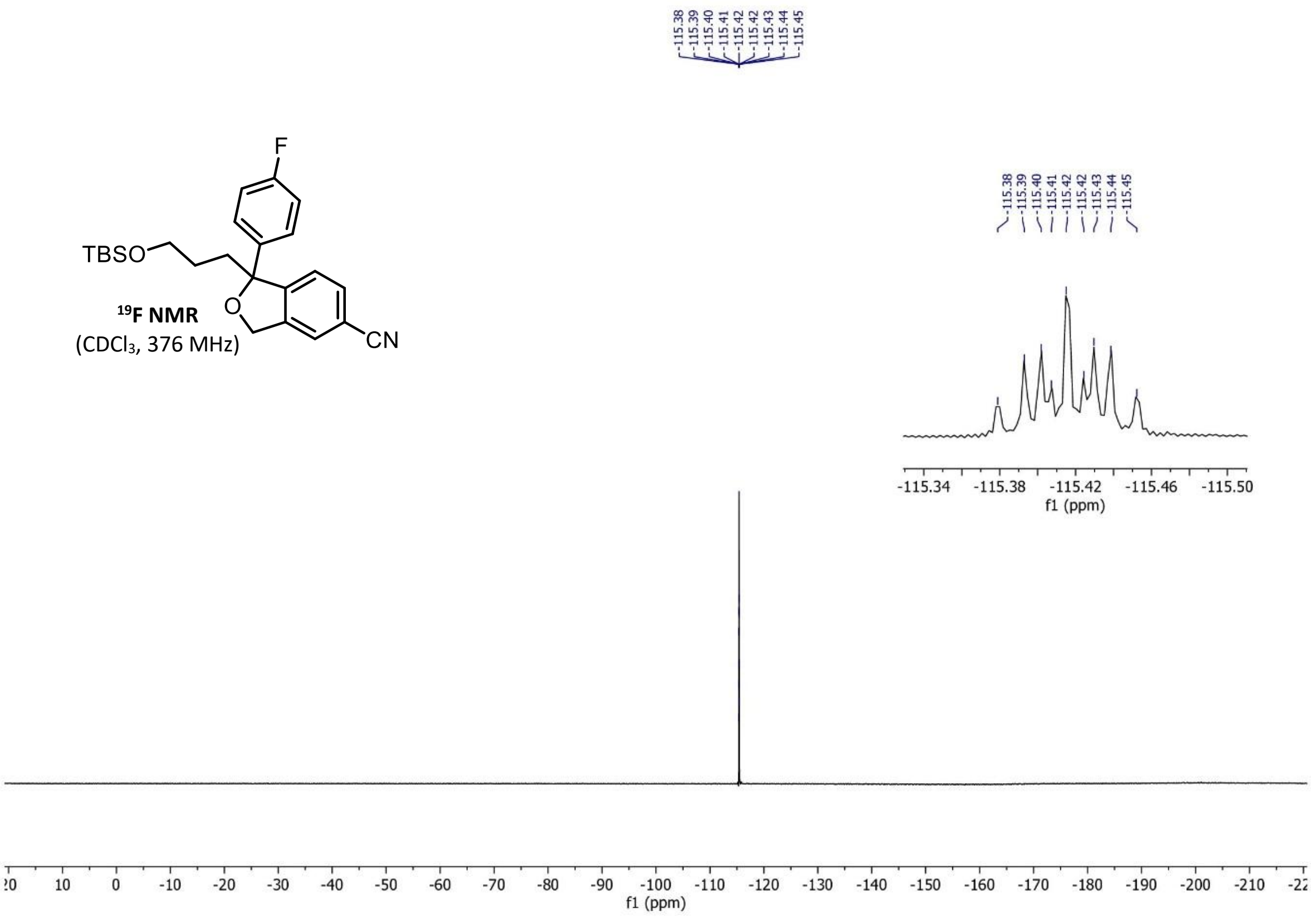

19F NMR
(CDCl3, 376 MHz)
-115.38
-115.39
-115.40
-115.41
-115.42
-115.42
-115.43
-115.44
-115.45
-115.34 -115.38 -115.42 -115.46 -115.50
f1 (ppm)
20 10 0 -10 -20 -30 -40 -50 -60 -70 -80 -90 -100 -110 -120 -130 -140 -150 -160 -170 -180 -190 -200 -210 -22
f1 (ppm)

1-(4-Fluorophenyl)-1-(3-hydroxypropyl)-1,3-dihydroisobenzofuran-5-carbonitrile (S13)

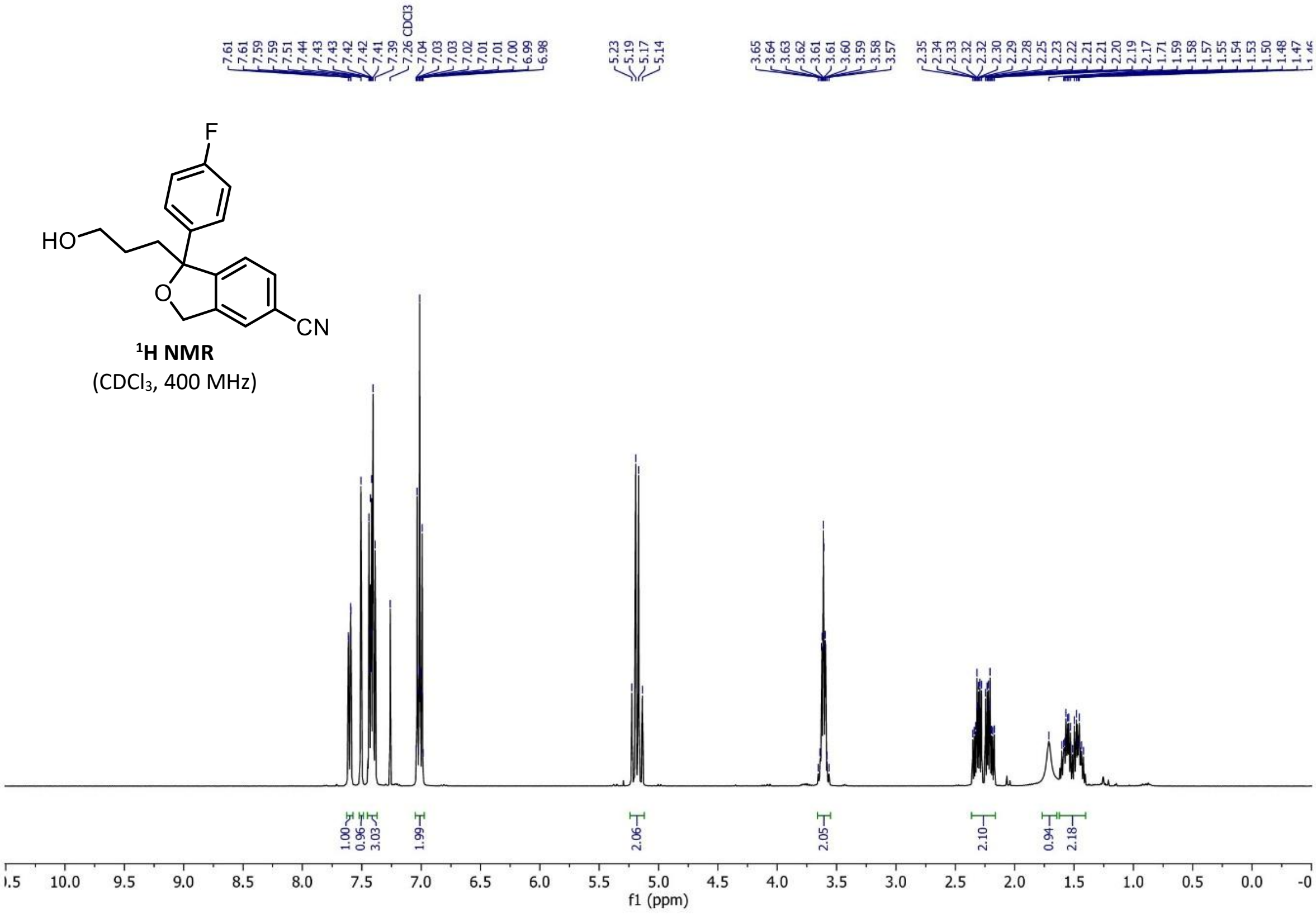

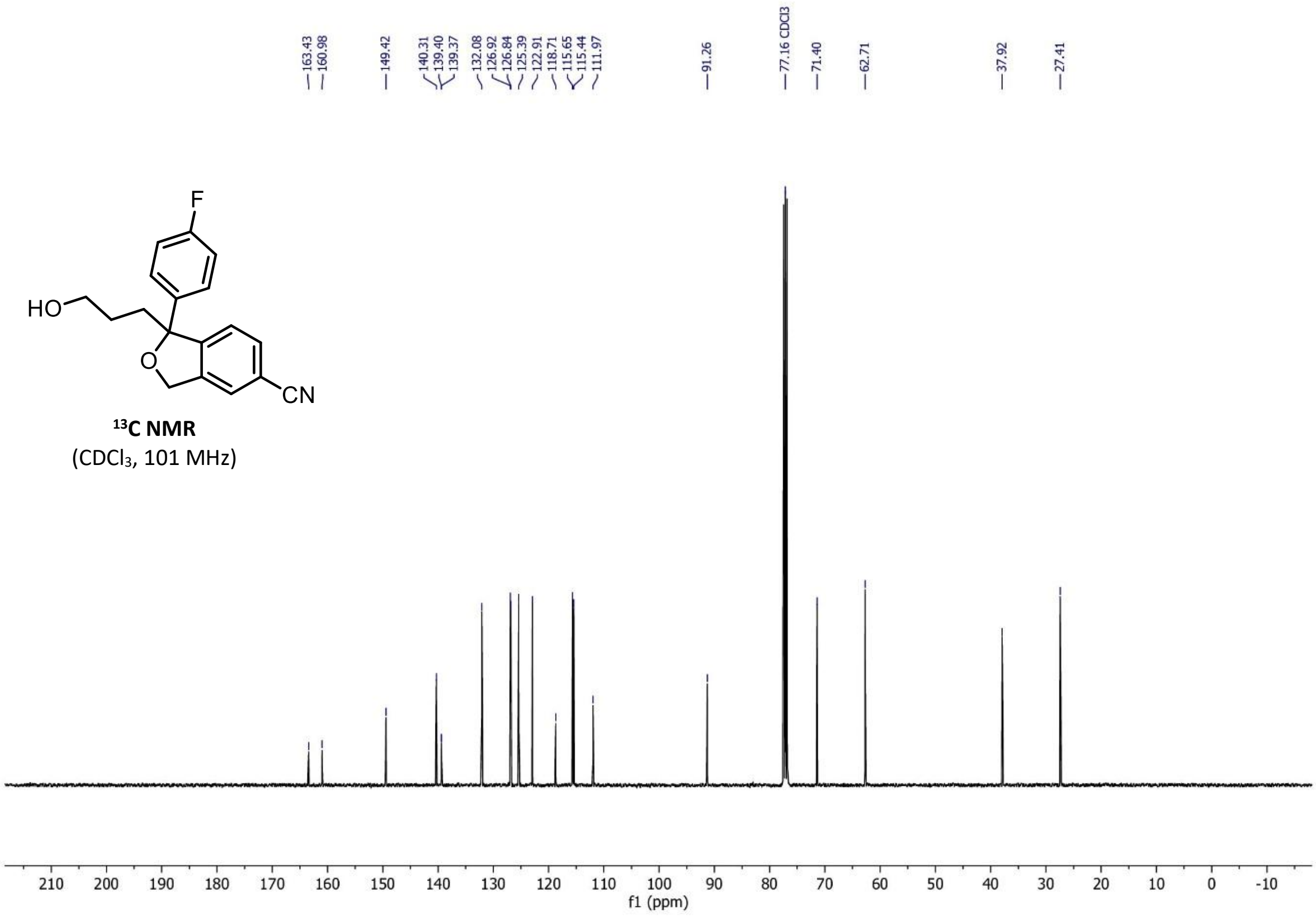

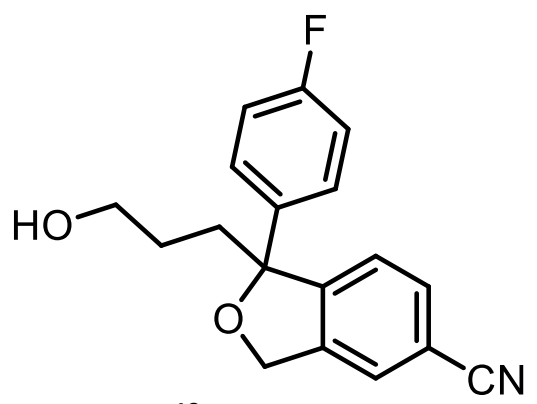

^{19}F NMR
($CDCl_3$, 376 MHz)

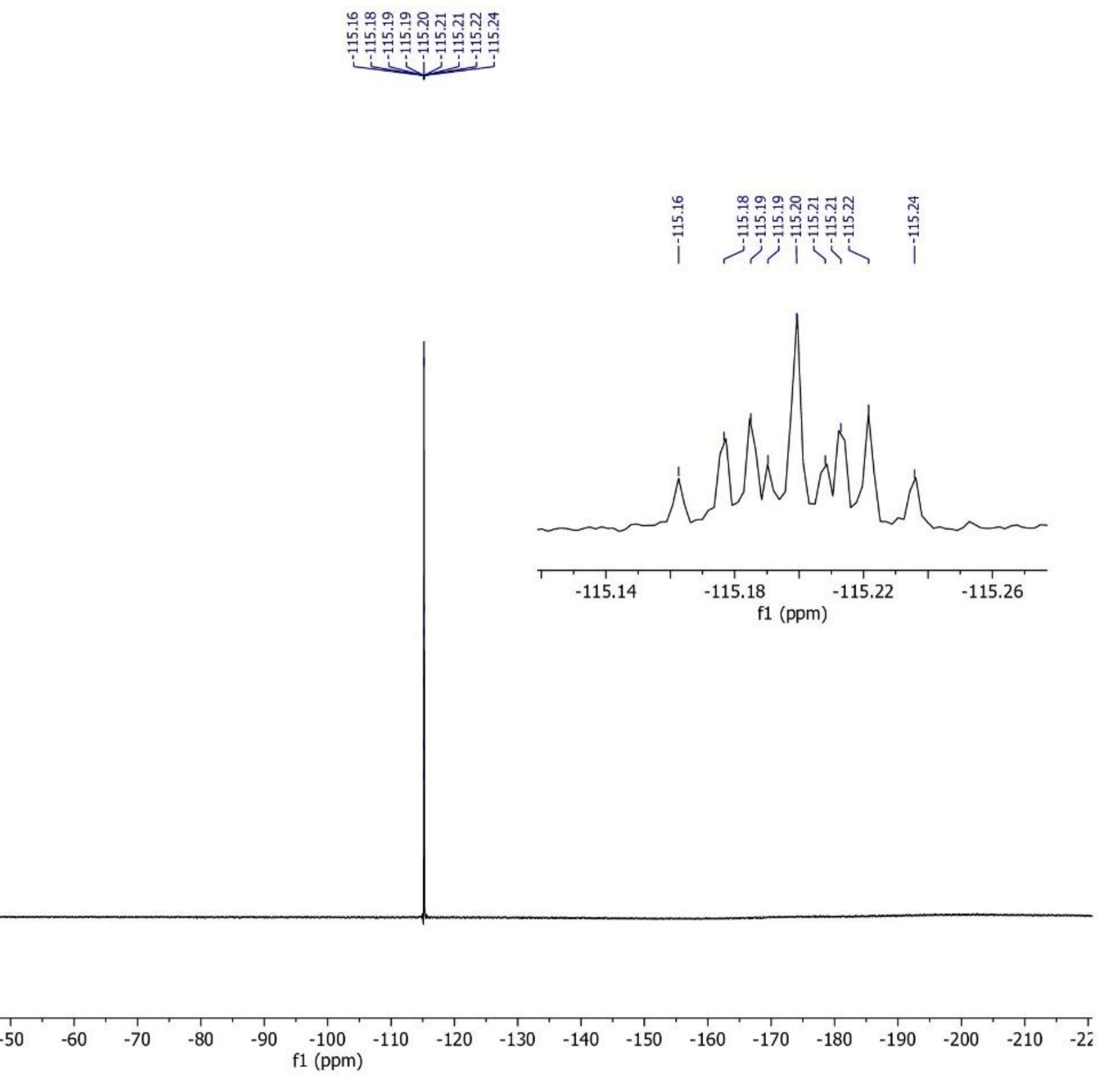

3-(3-Chloro-10,11-dihydro-5*H*-dibenzo[*b*,*f*]azepin-5-yl)propan-1-ol (S15)

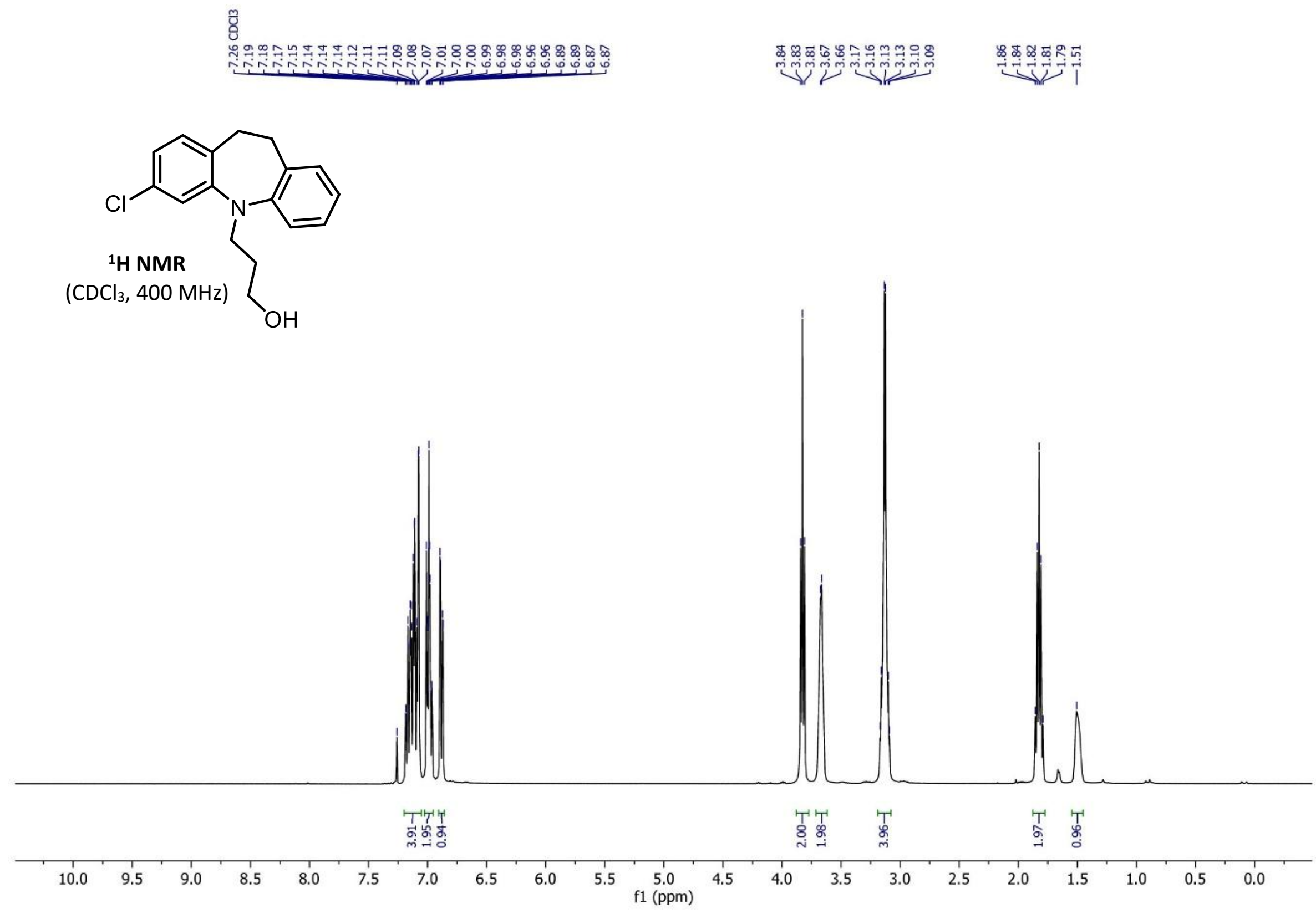

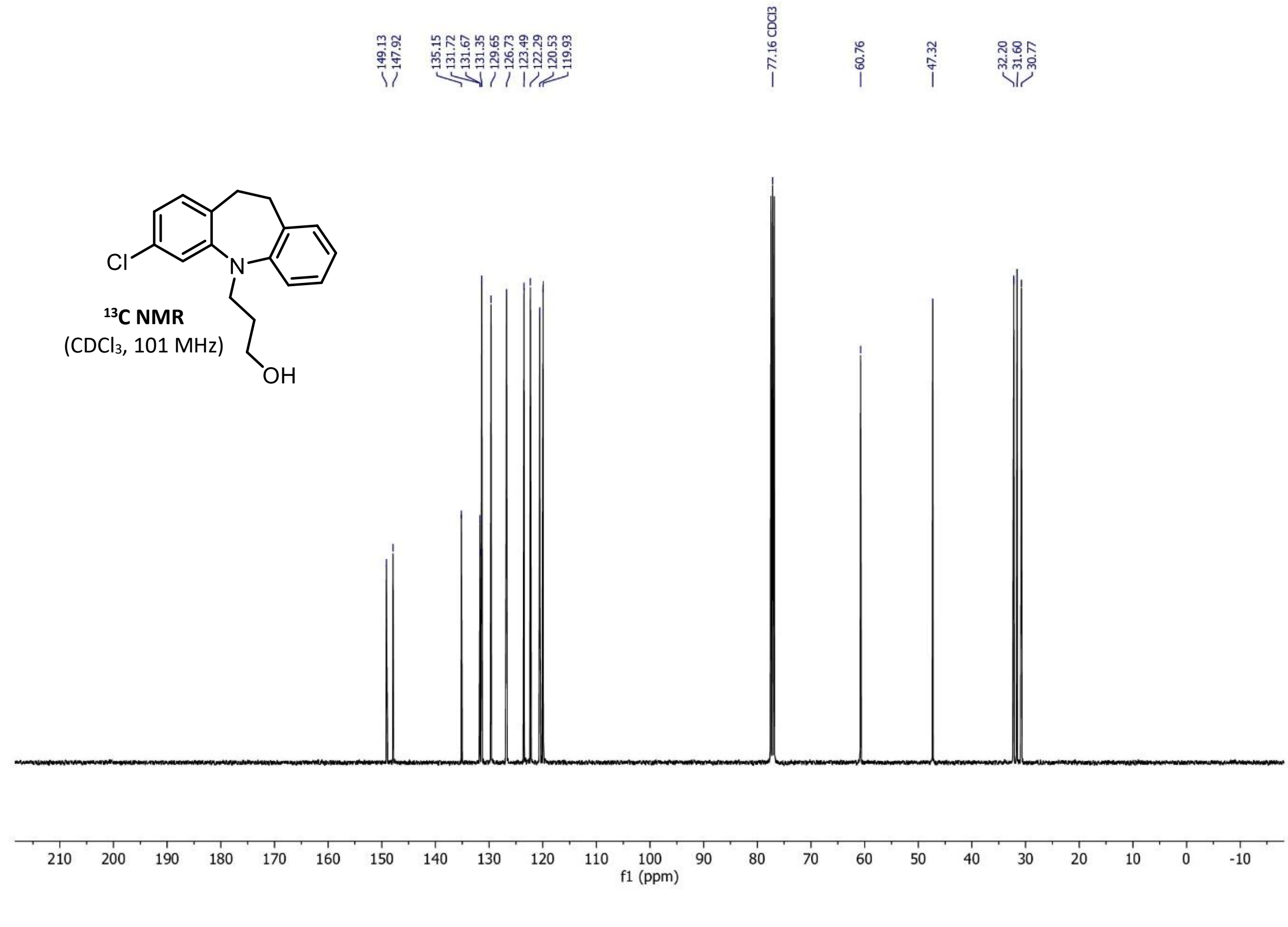

***tert*-Butyl (4-((2,4,6-tris(trifluoromethyl)phenyl)sulfonamido)butyl)carbamate (S16)**

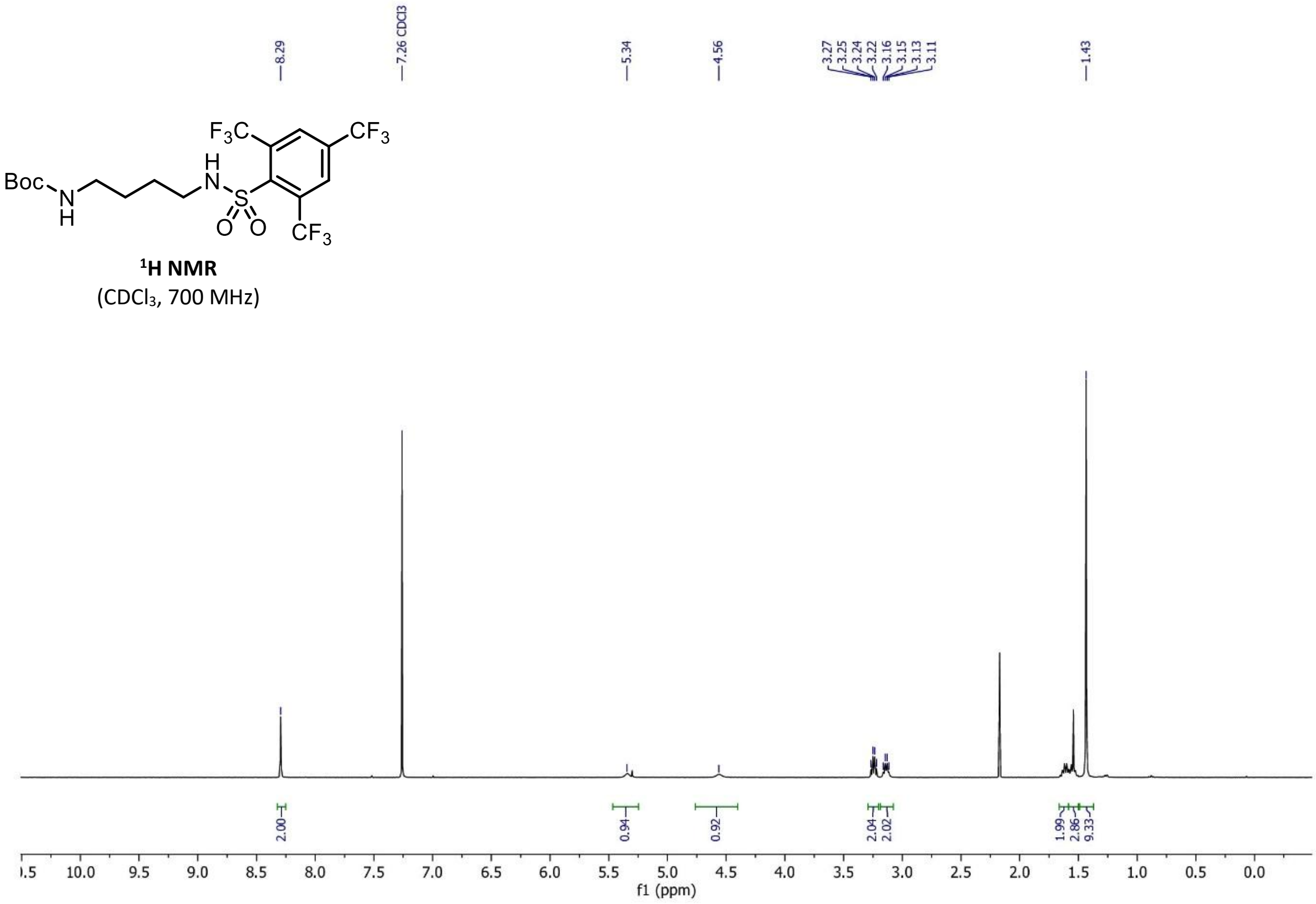

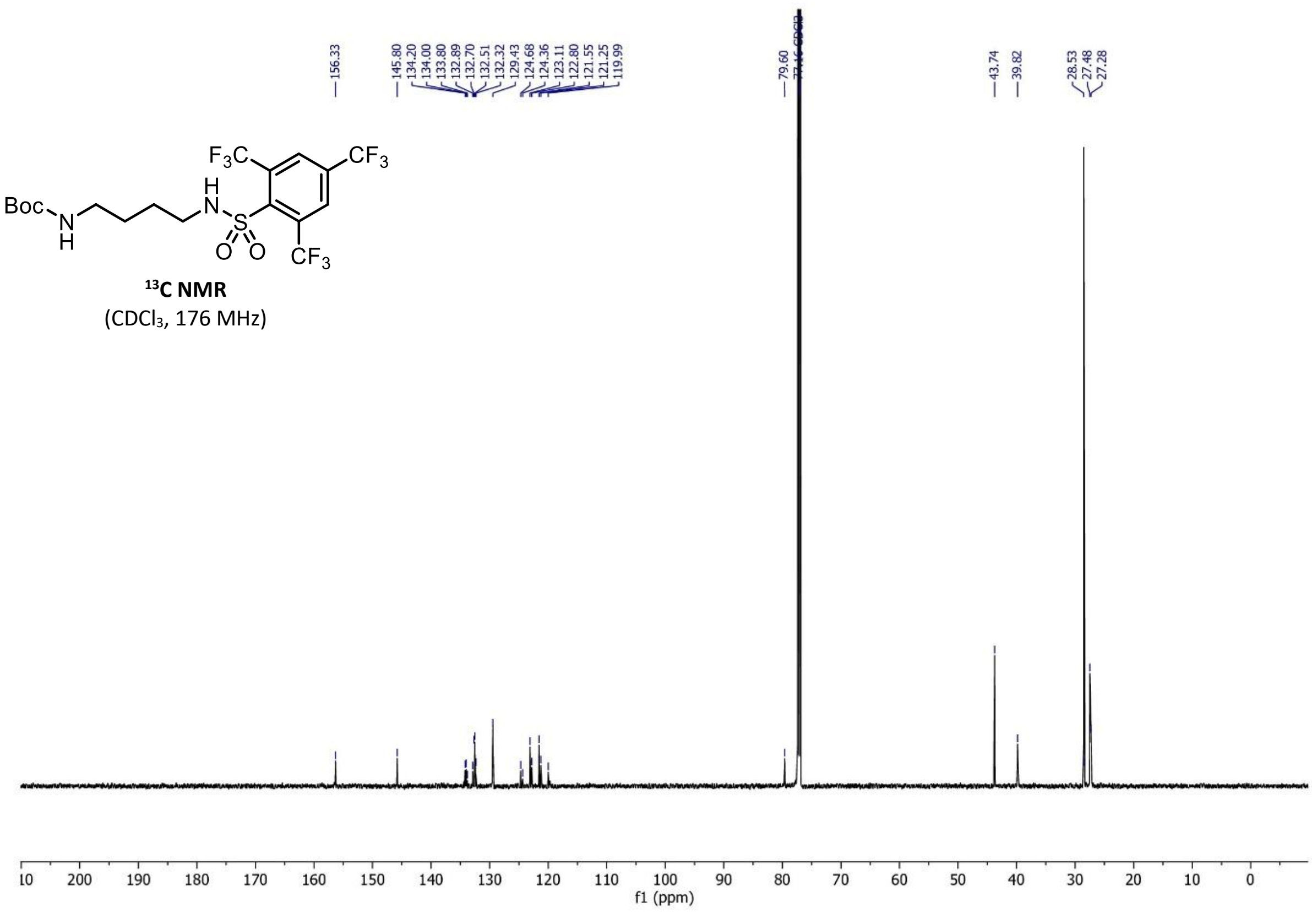

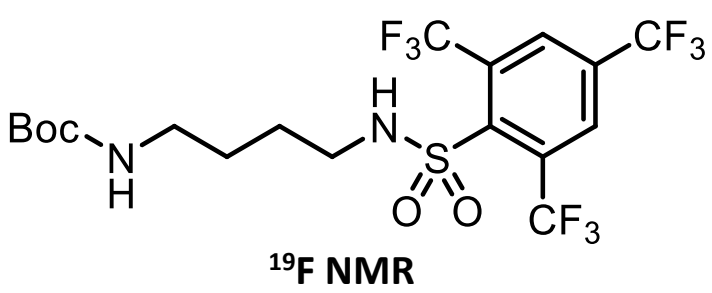

^{19}F NMR
(CDCl₃, 376 MHz)

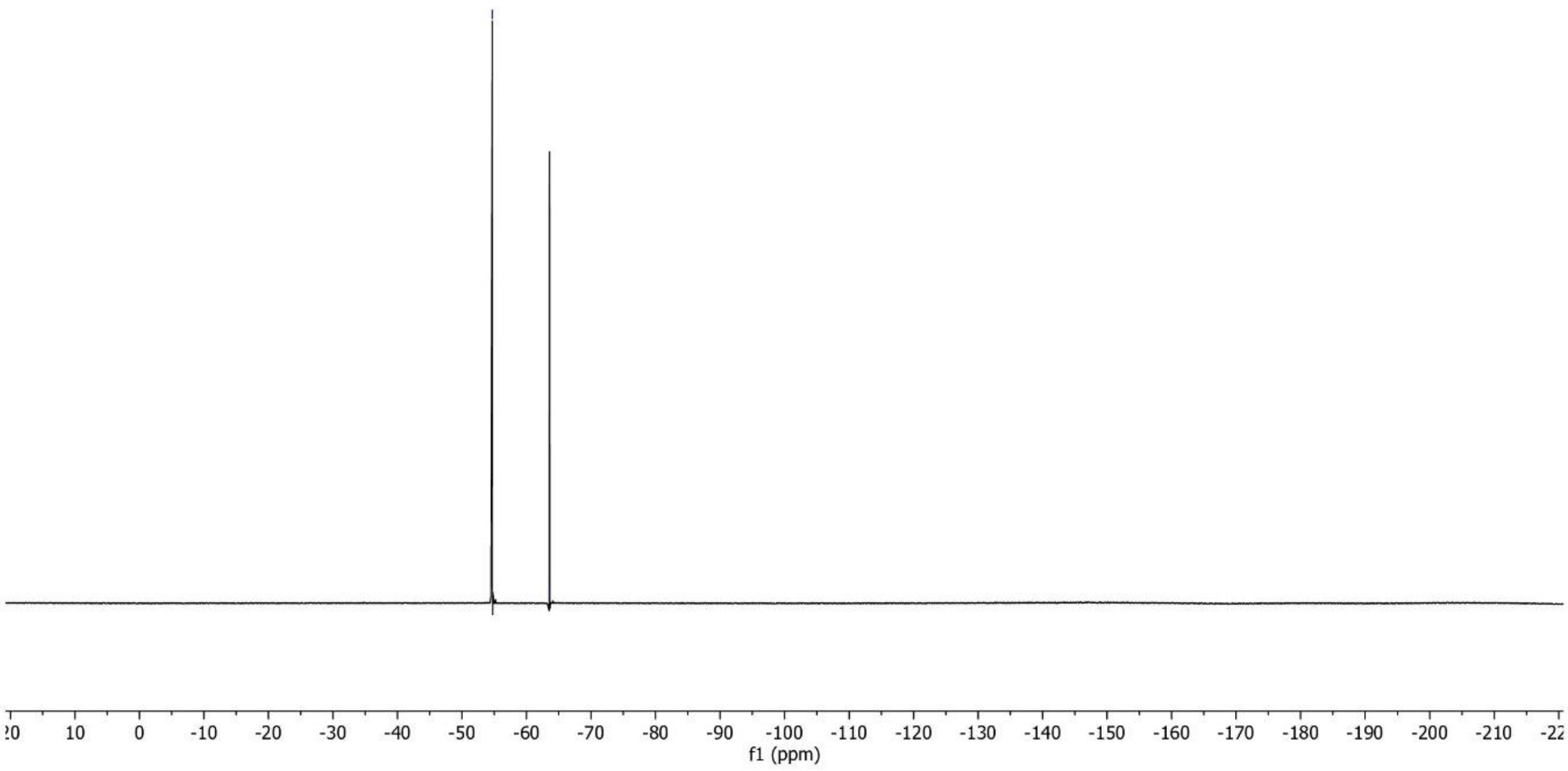

(*R*)-*N*-(4-Oxo-4-(3-(trifluoromethyl)-5,6-dihydro-[1,2,4]triazolo[4,3-*a*]pyrazin-7(8*H*)-yl)-1-(2,4,5-trifluorophenyl)butan-2-yl)-2,4,6-tris(trifluoromethyl)benzenesulfonamide (S17)

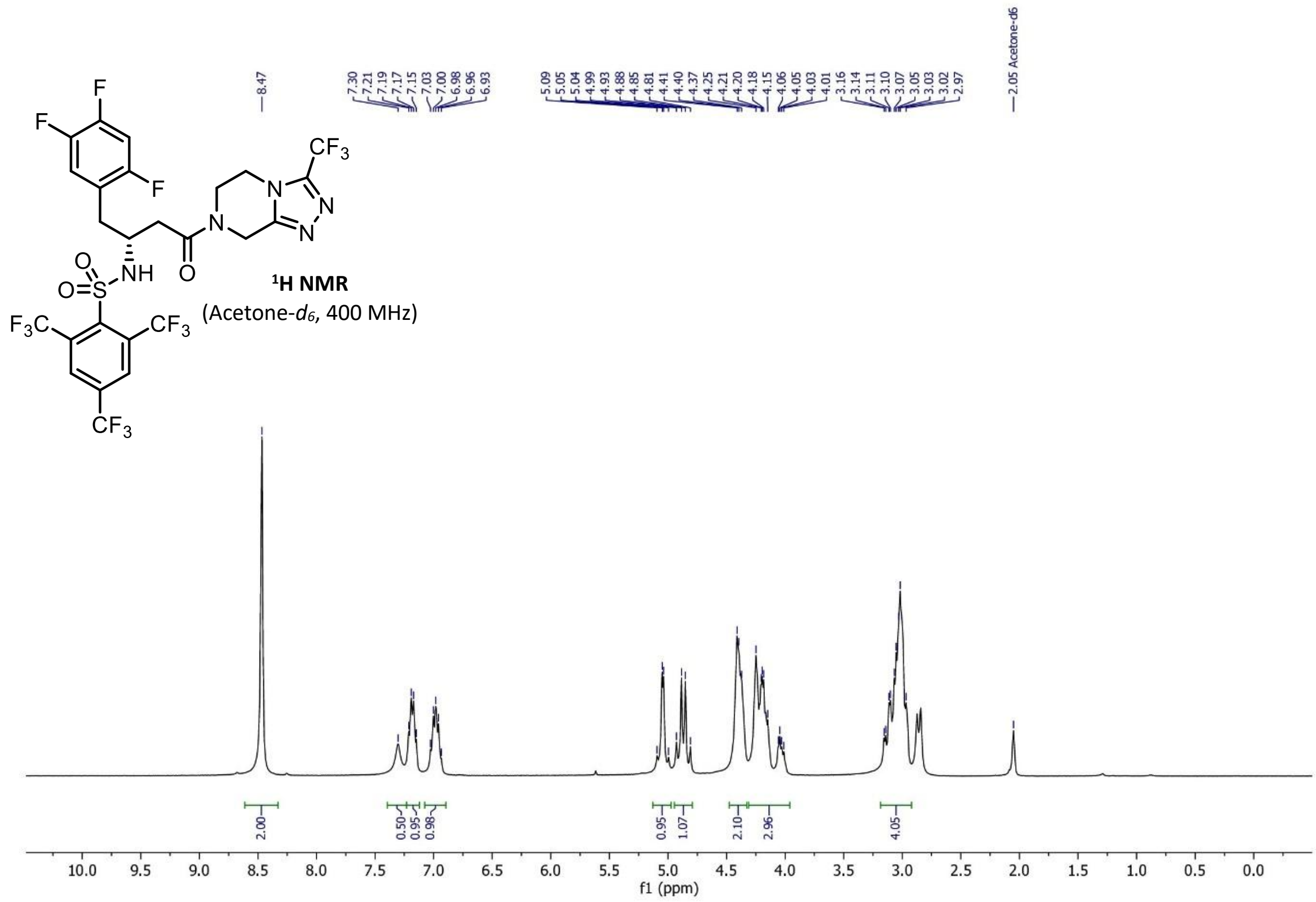

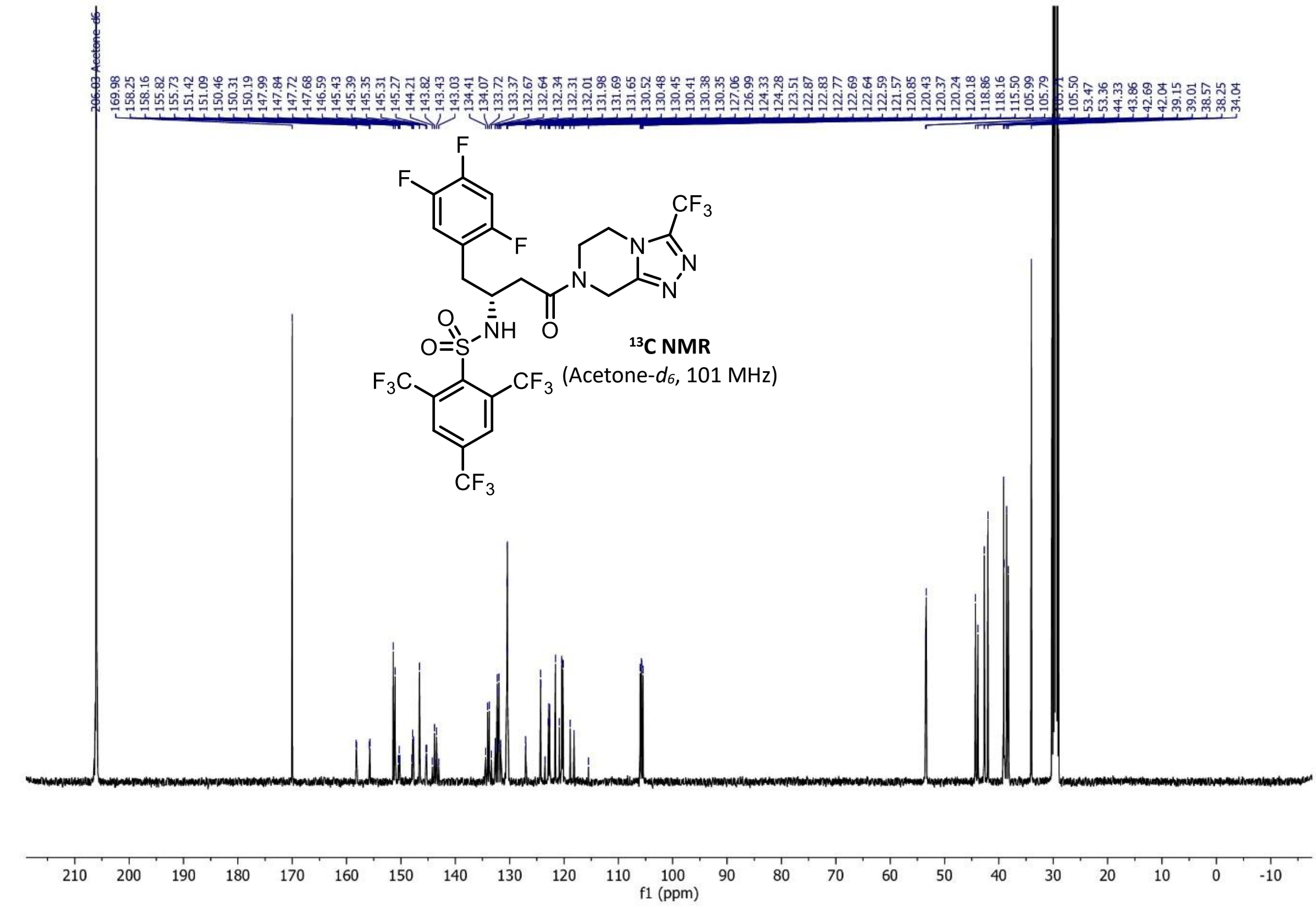

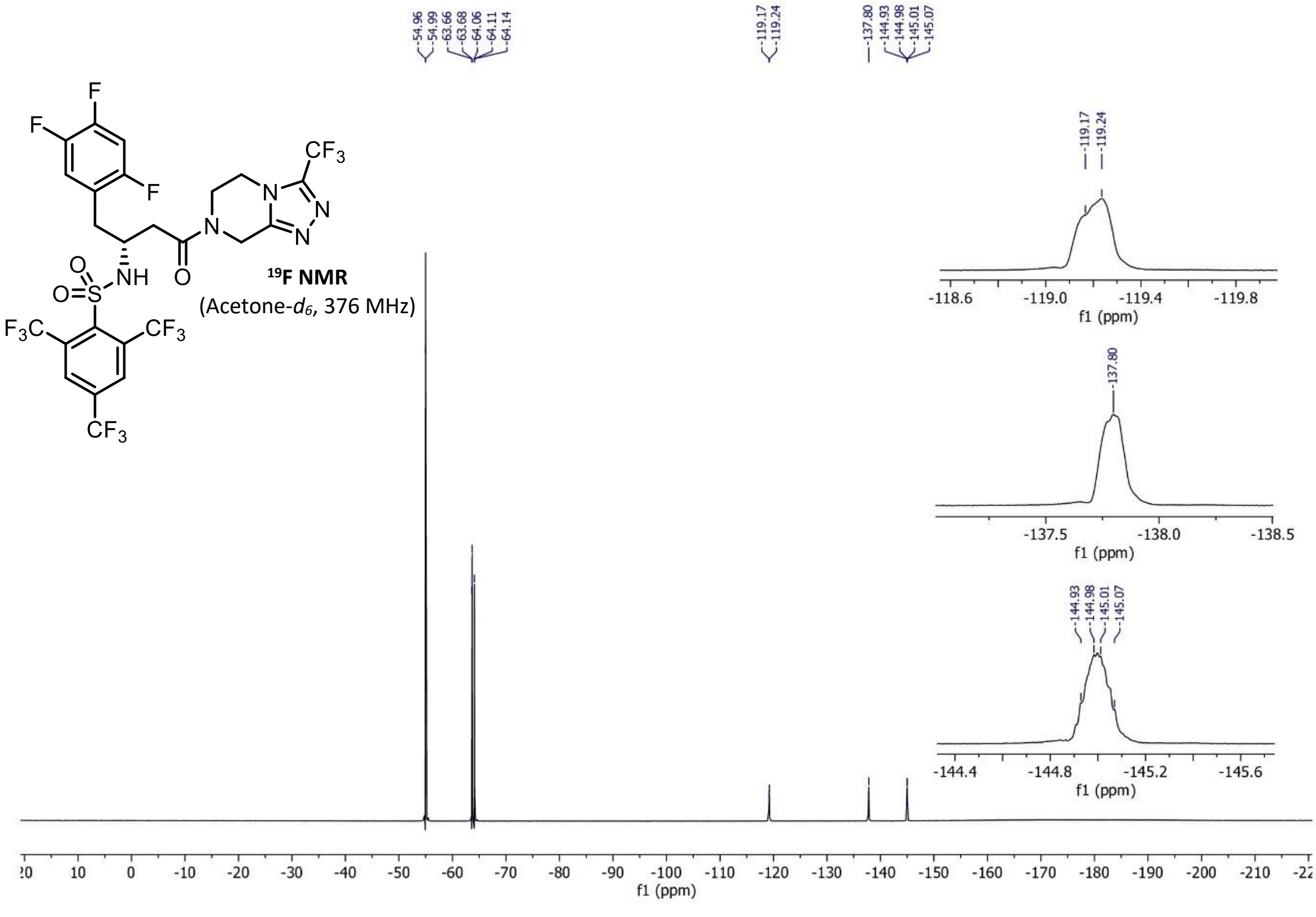

¹⁹F NMR
(Acetone-d6, 376 MHz)
-54.96
-54.99
-63.66
-63.68
-64.06
-64.11
-64.14
-119.17
-119.24
-137.80
-144.93
-144.98
-145.01
-145.07
f1 (ppm)

(*R*)-*N*-(1-(3-(2-Cyanobenzyl)-1-methyl-2,6-dioxo-1,2,3,6-tetrahydropyrimidin-4-yl)piperidin-3-yl)-2,4,6-tris(trifluoromethyl)benzenesulfonamide (S18)

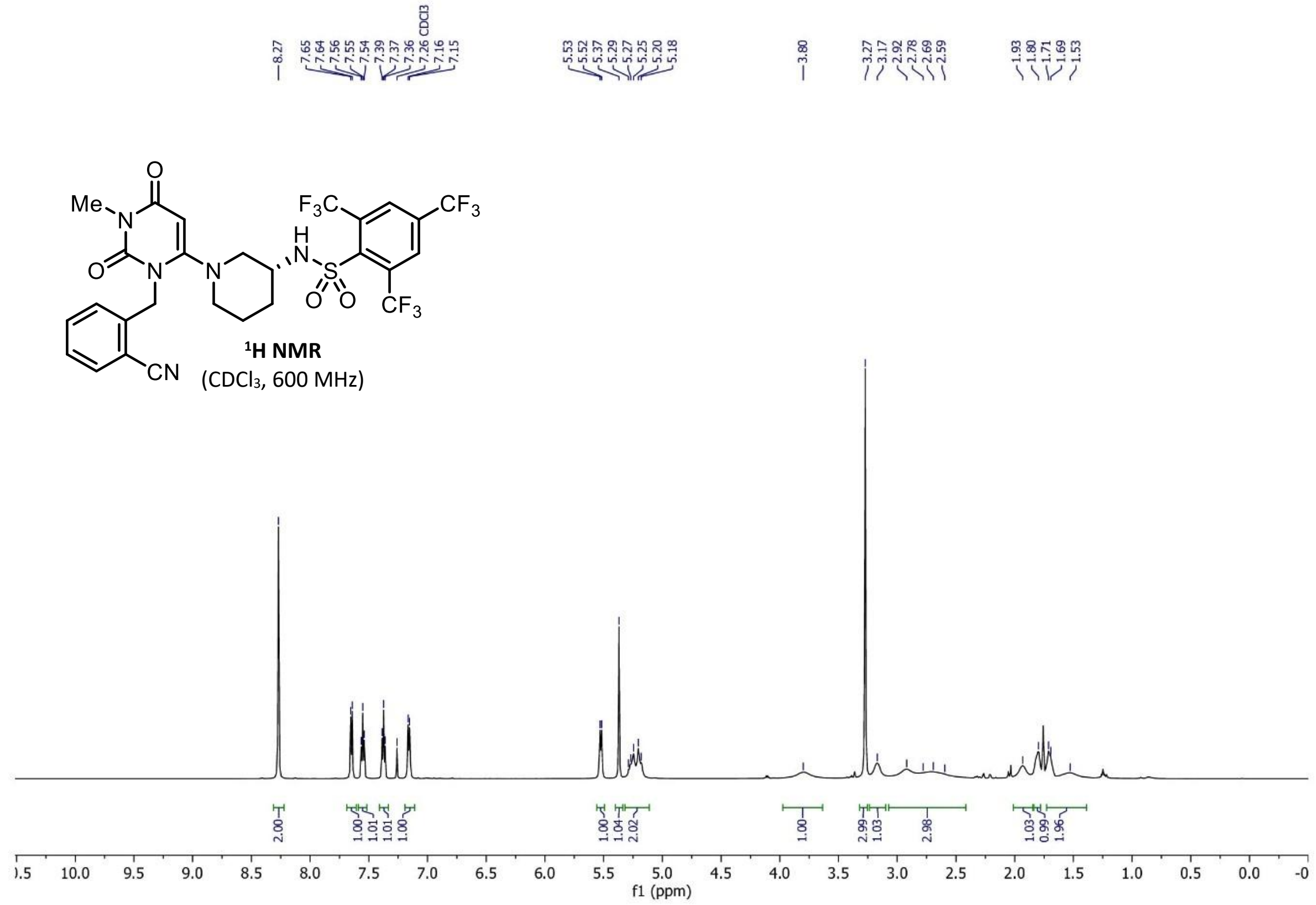

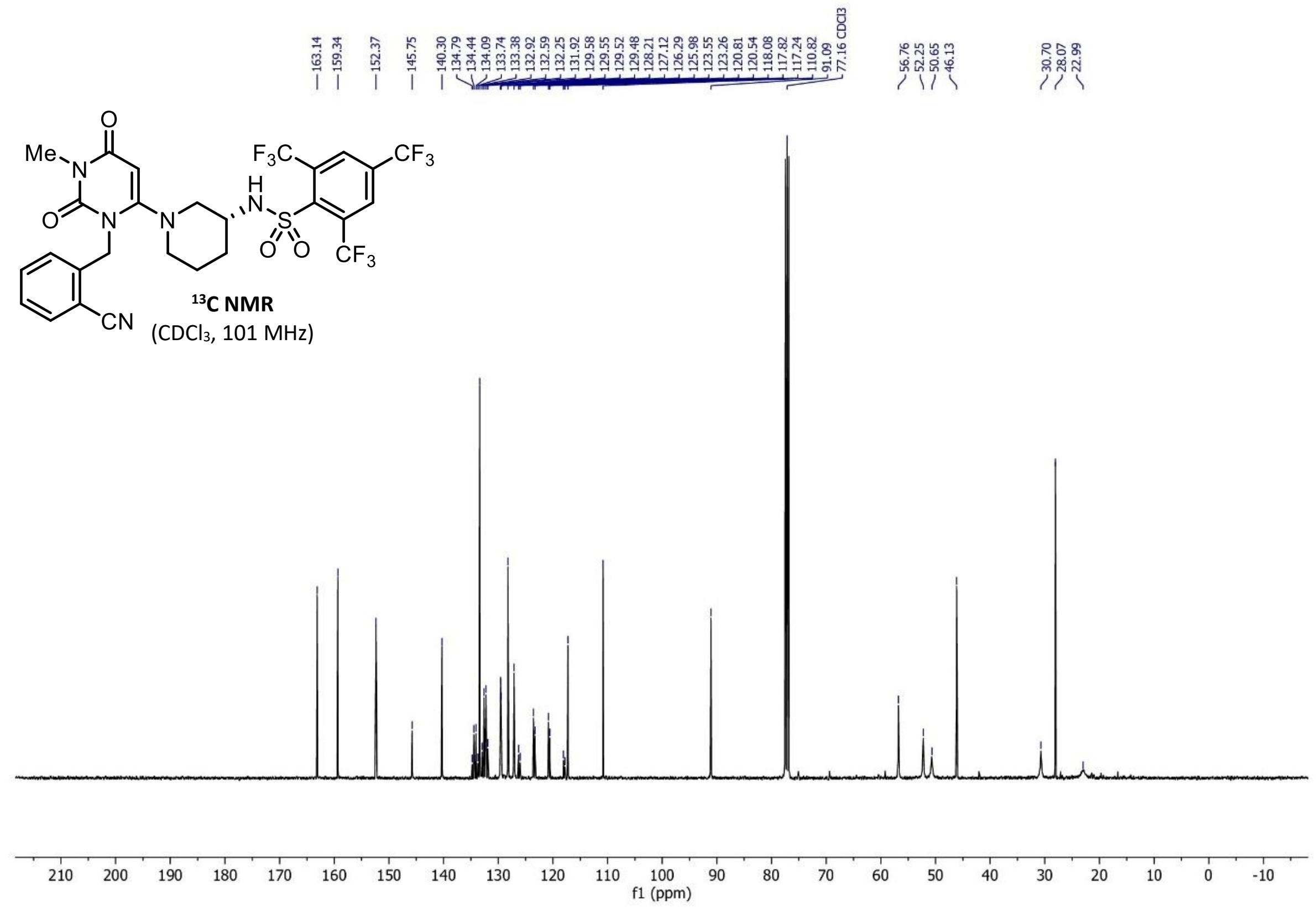

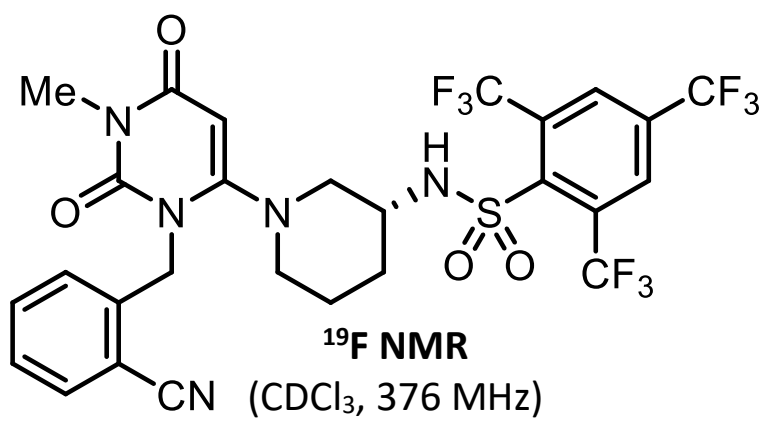

^{19}F NMR
($CDCl_3$, 376 MHz)

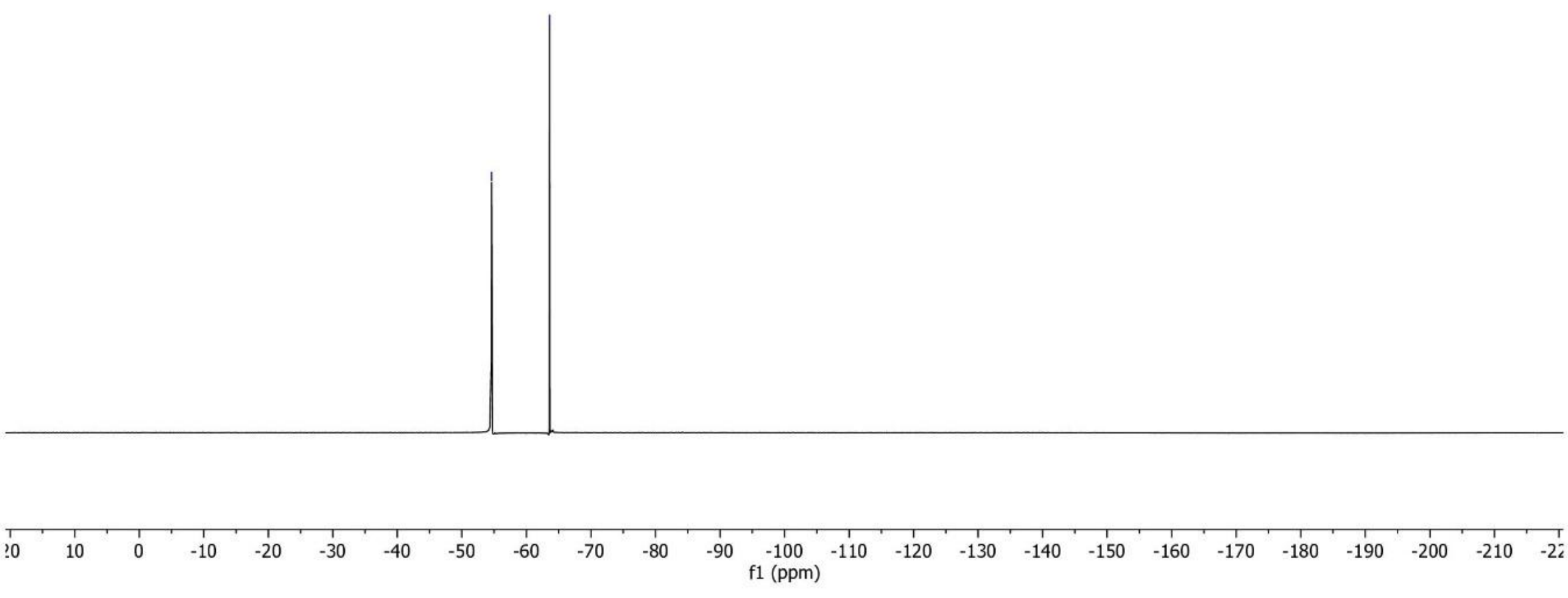

***N*-(4-((6-Methoxyquinolin-8-yl)amino)pentyl)-2,4,6-tris(trifluoromethyl)benzenesulfonamide (S19)**

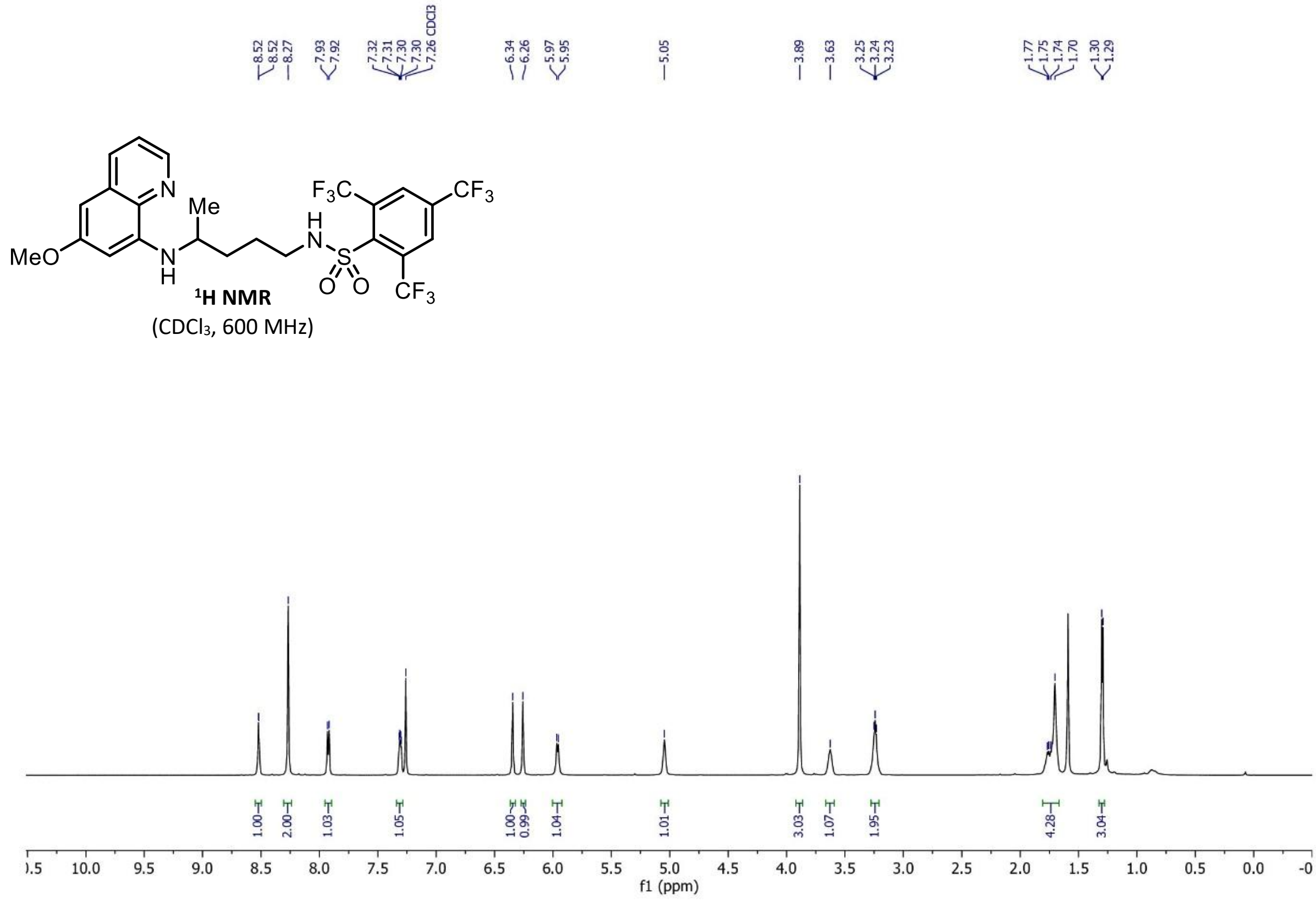

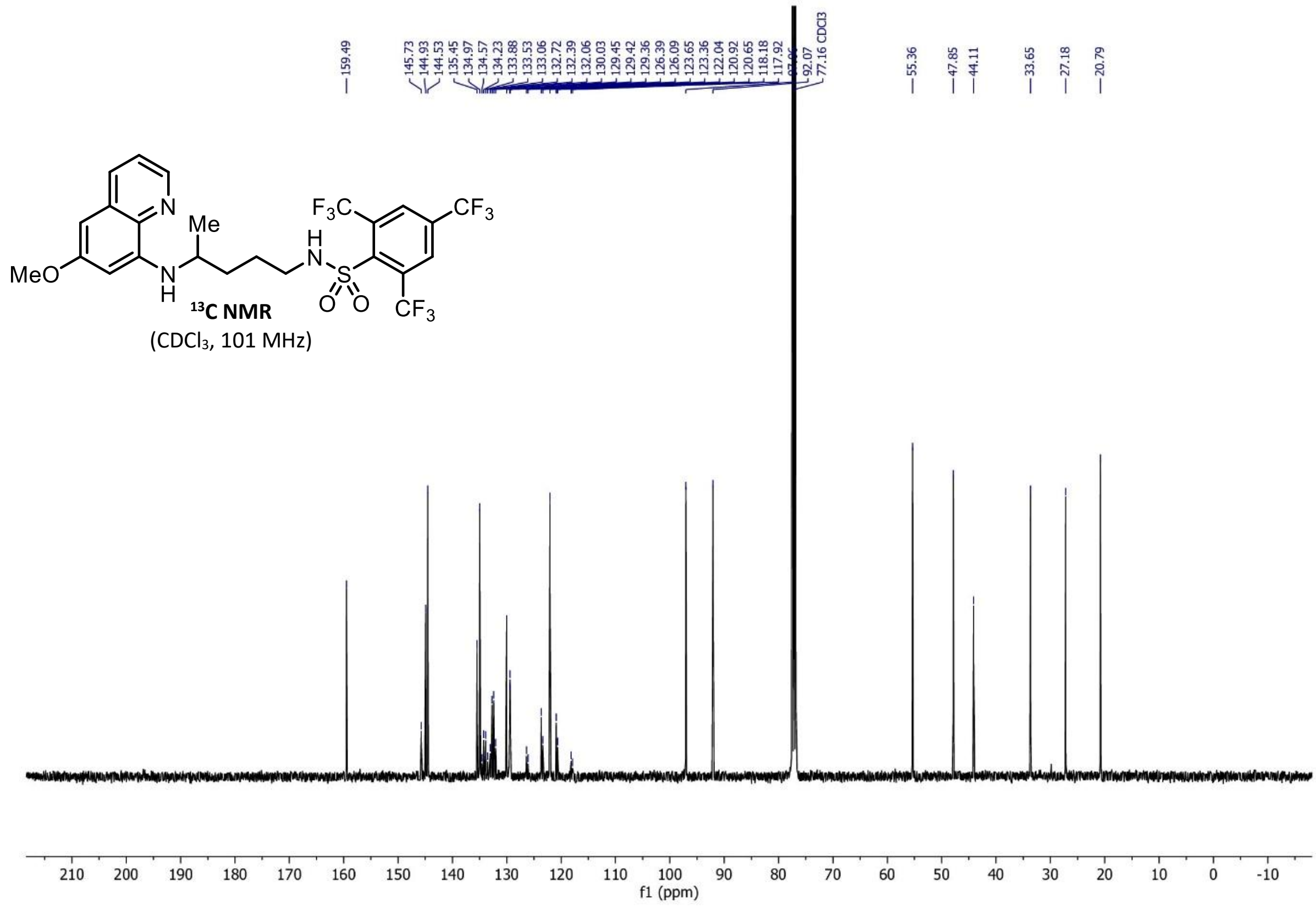

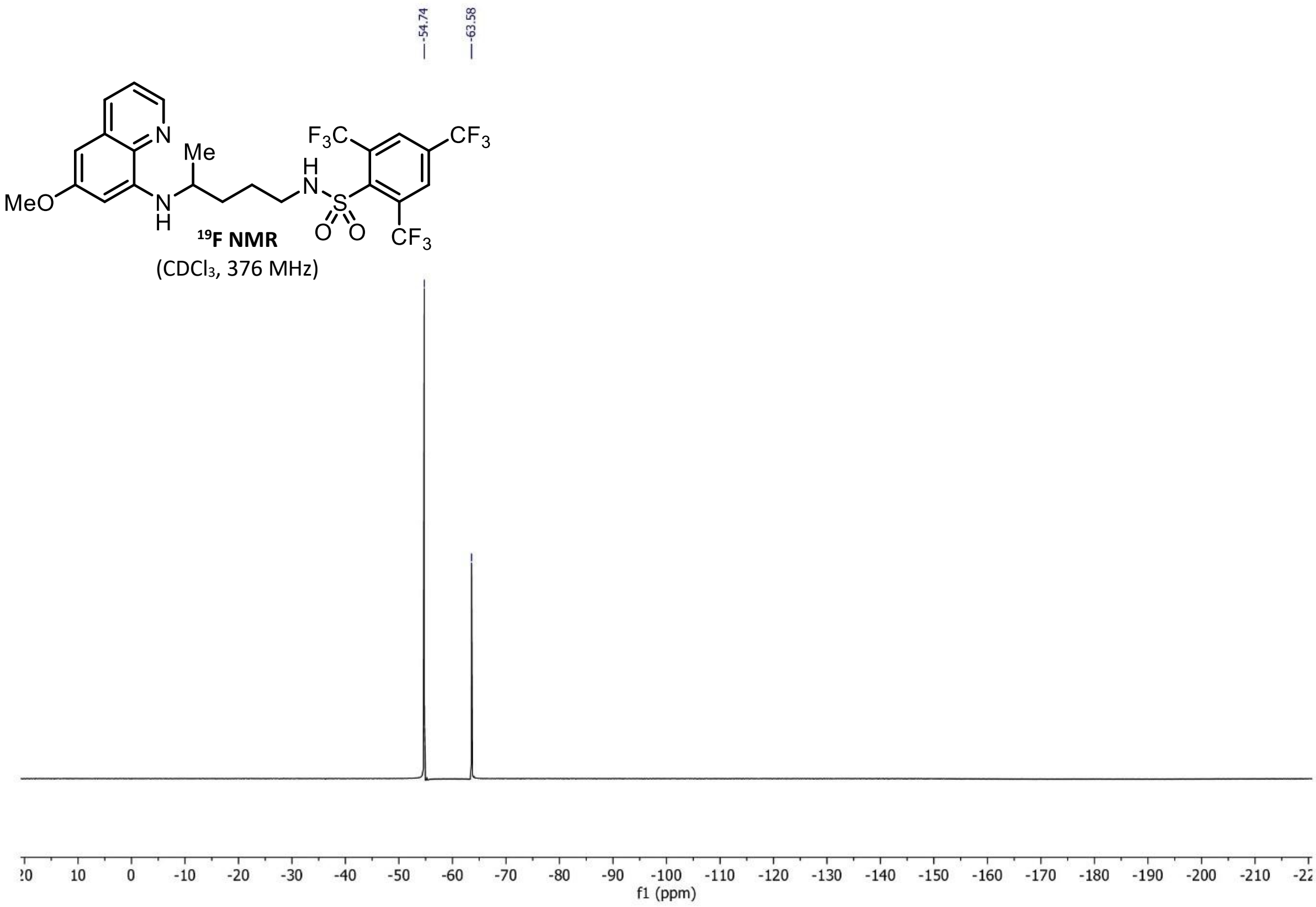

^{19}F NMR
($CDCl_3$, 376 MHz)

N-(4-Methoxyphenyl)-N-methyl-2,4,6-tris(trifluoromethyl)benzenesulfonamide (S20)

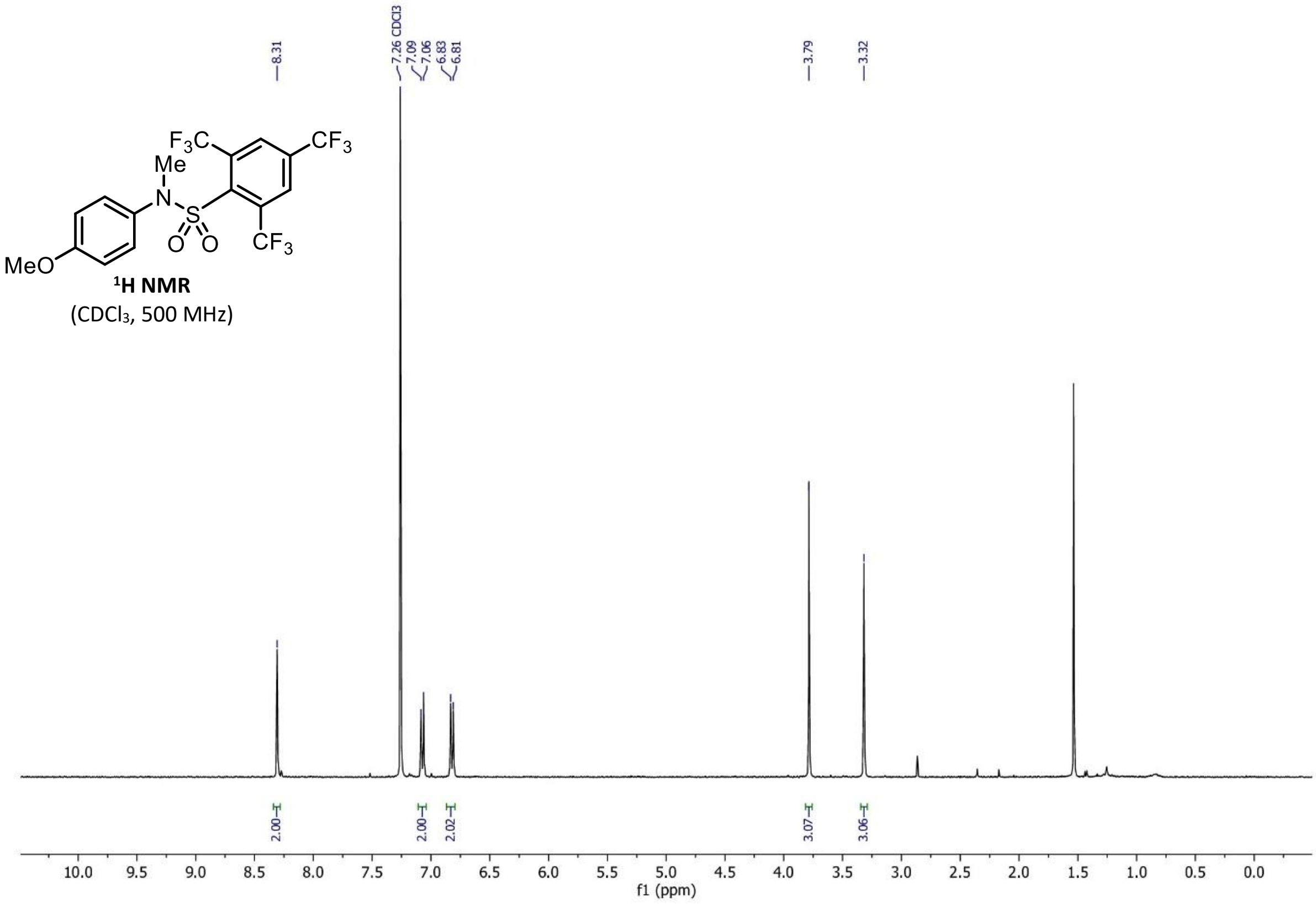

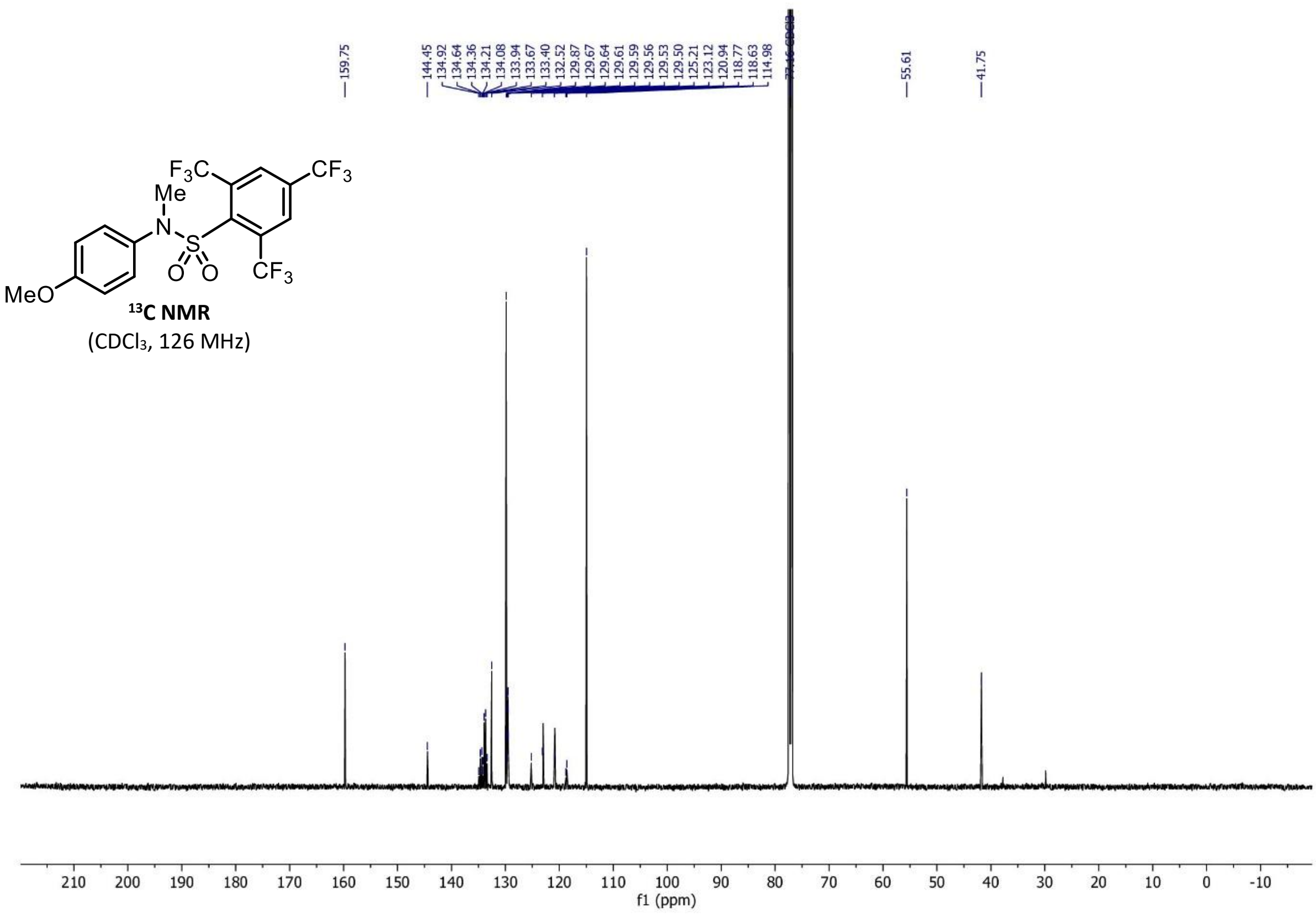

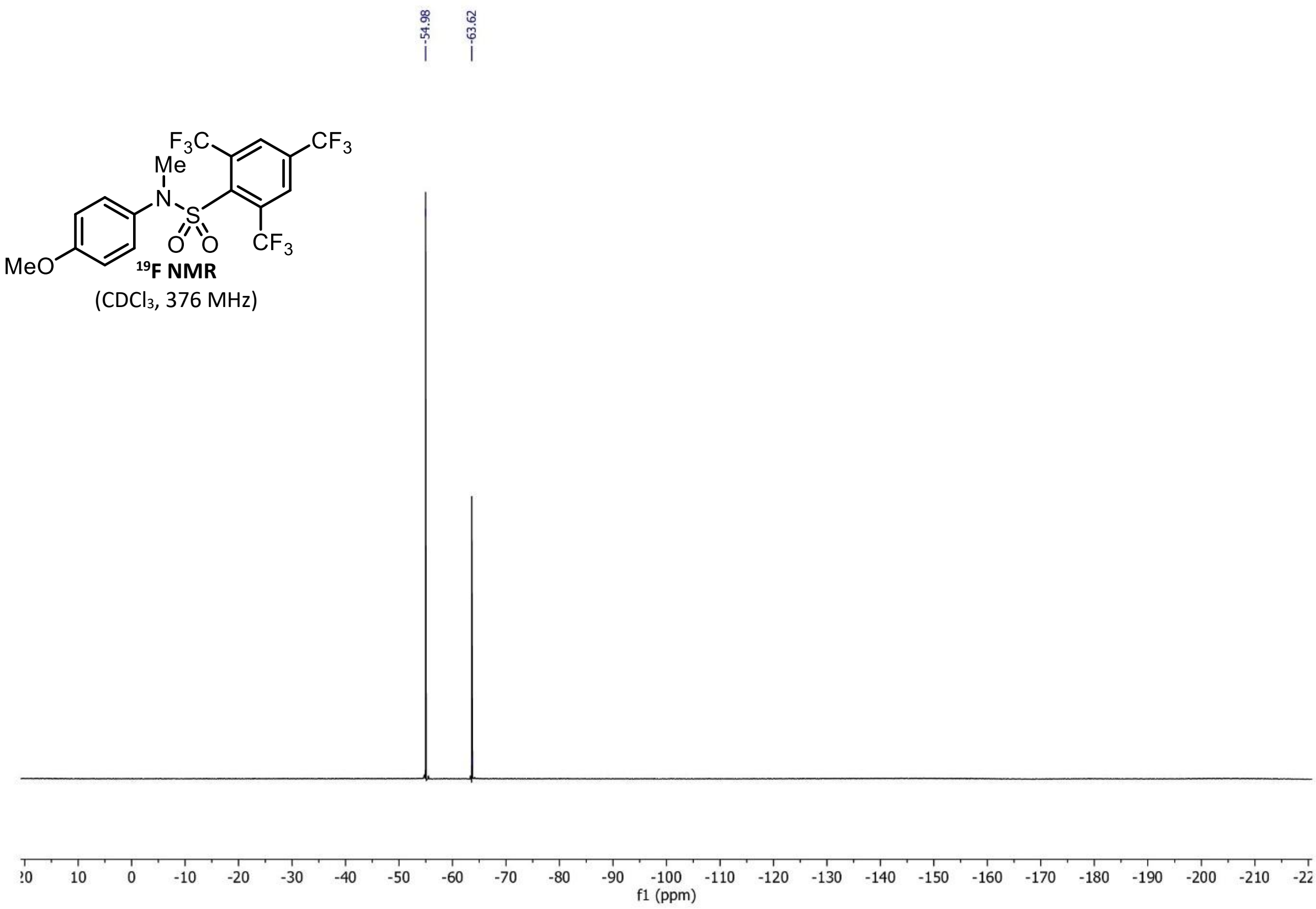

-54.98
-63.62
F3C
CF3
Me
N
S
O O
CF3
MeO
19F NMR
(CDCl3, 376 MHz)
20 10 0 -10 -20 -30 -40 -50 -60 -70 -80 -90 -100 -110 -120 -130 -140 -150 -160 -170 -180 -190 -200 -210 -22
f1 (ppm)

tert-Butyl 4-((2,4,6-tris(trifluoromethyl)phenyl)sulfonyl)piperazine-1-carboxylate (7)

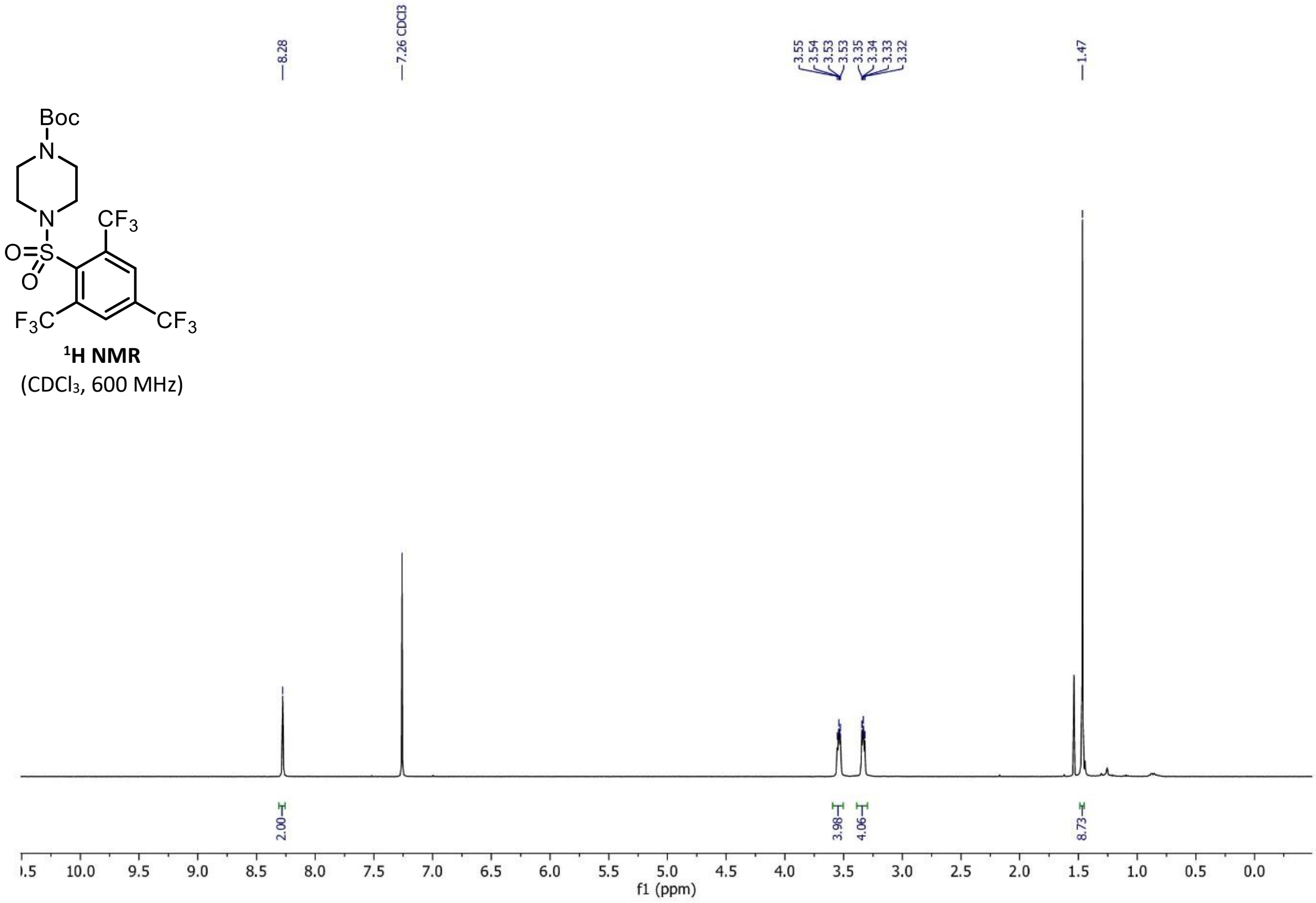

^{1}H NMR
($CDCl_3$, 600 MHz)

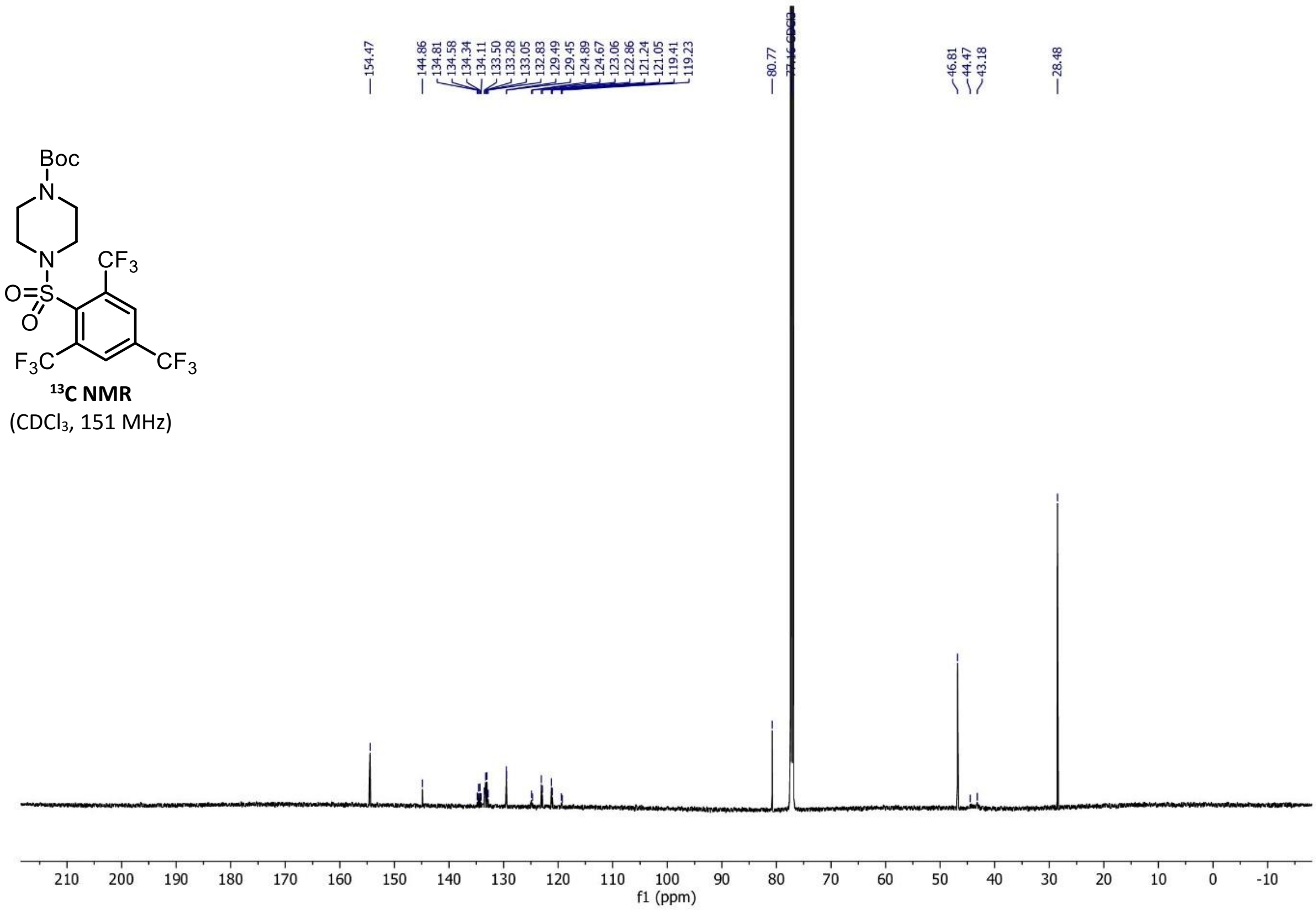

^{13}C NMR
($CDCl_3$, 151 MHz)

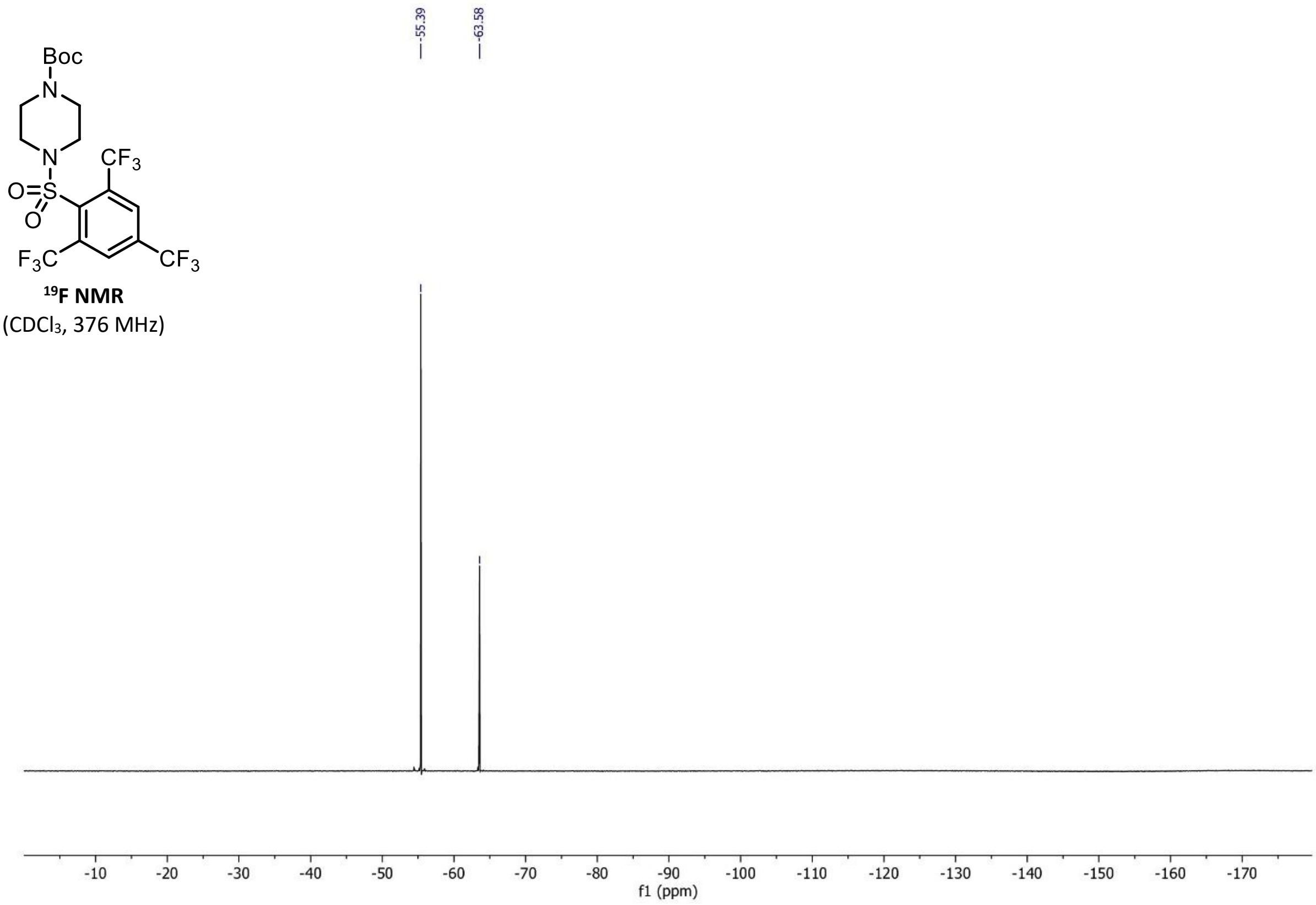

^{19}F NMR
($CDCl_3$, 376 MHz)

4-Phenyl-1-((2,4,6-tris(trifluoromethyl)phenyl)sulfonyl)piperidin-4-ol (S21)

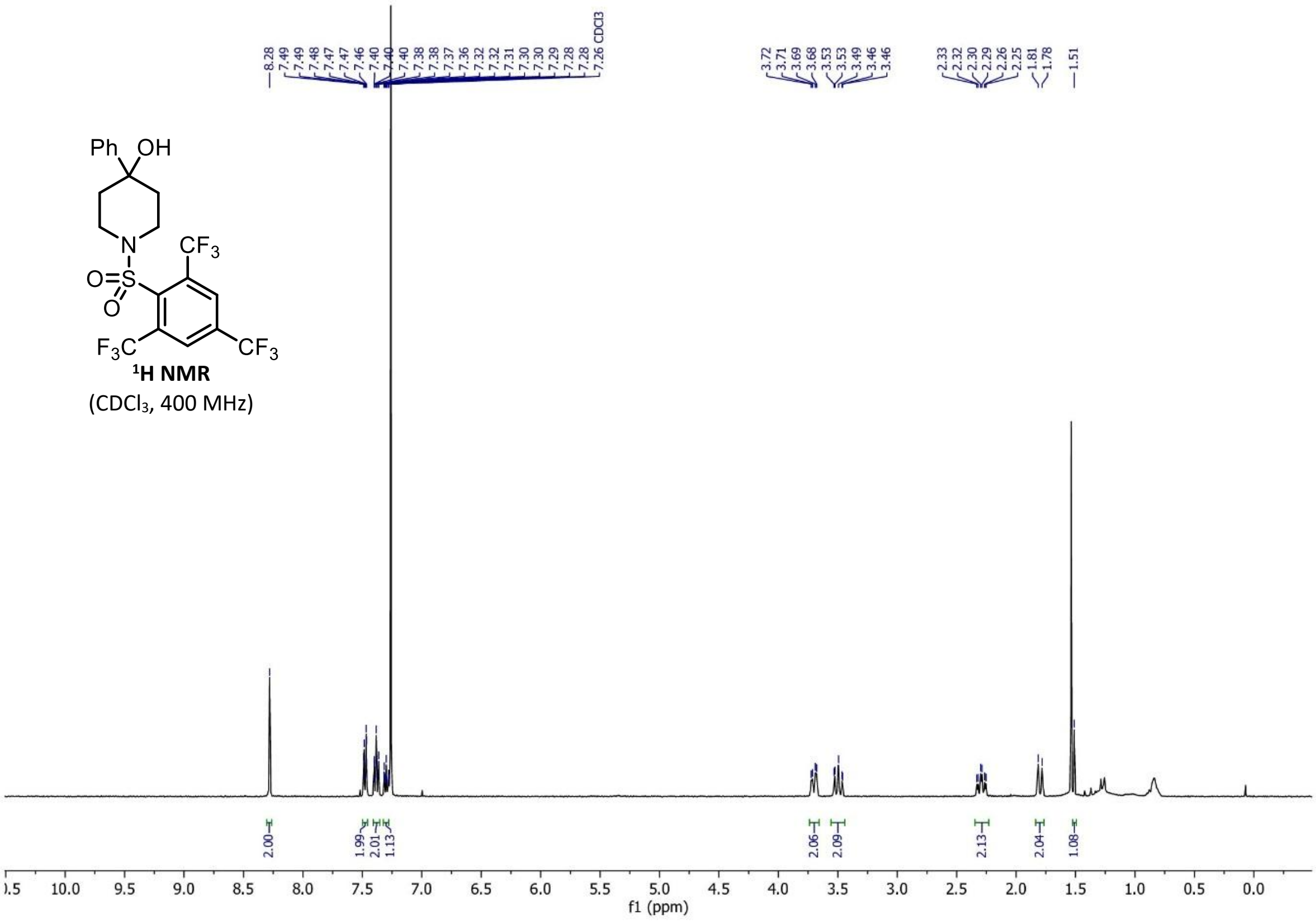

^{1}H NMR
($CDCl_3$, 400 MHz)

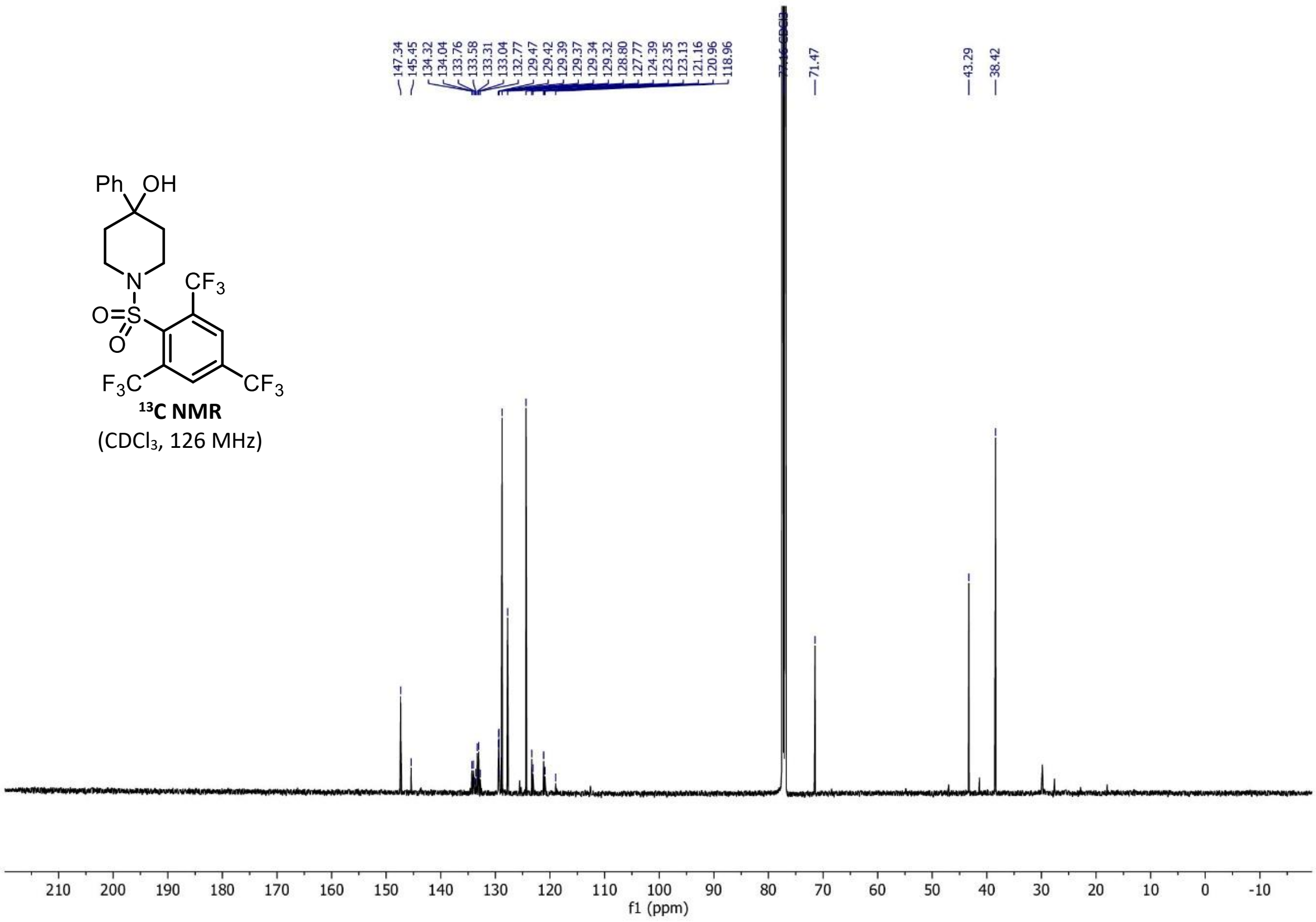

^{13}C NMR
($CDCl_3$, 126 MHz)

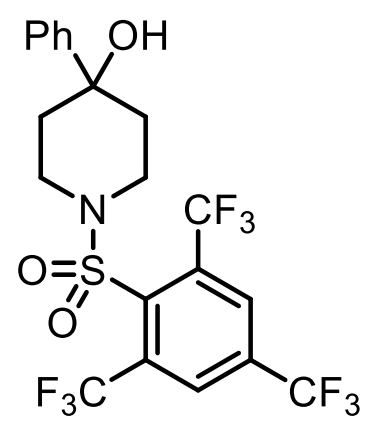

^{19}F NMR
($CDCl_3$, 659 MHz)

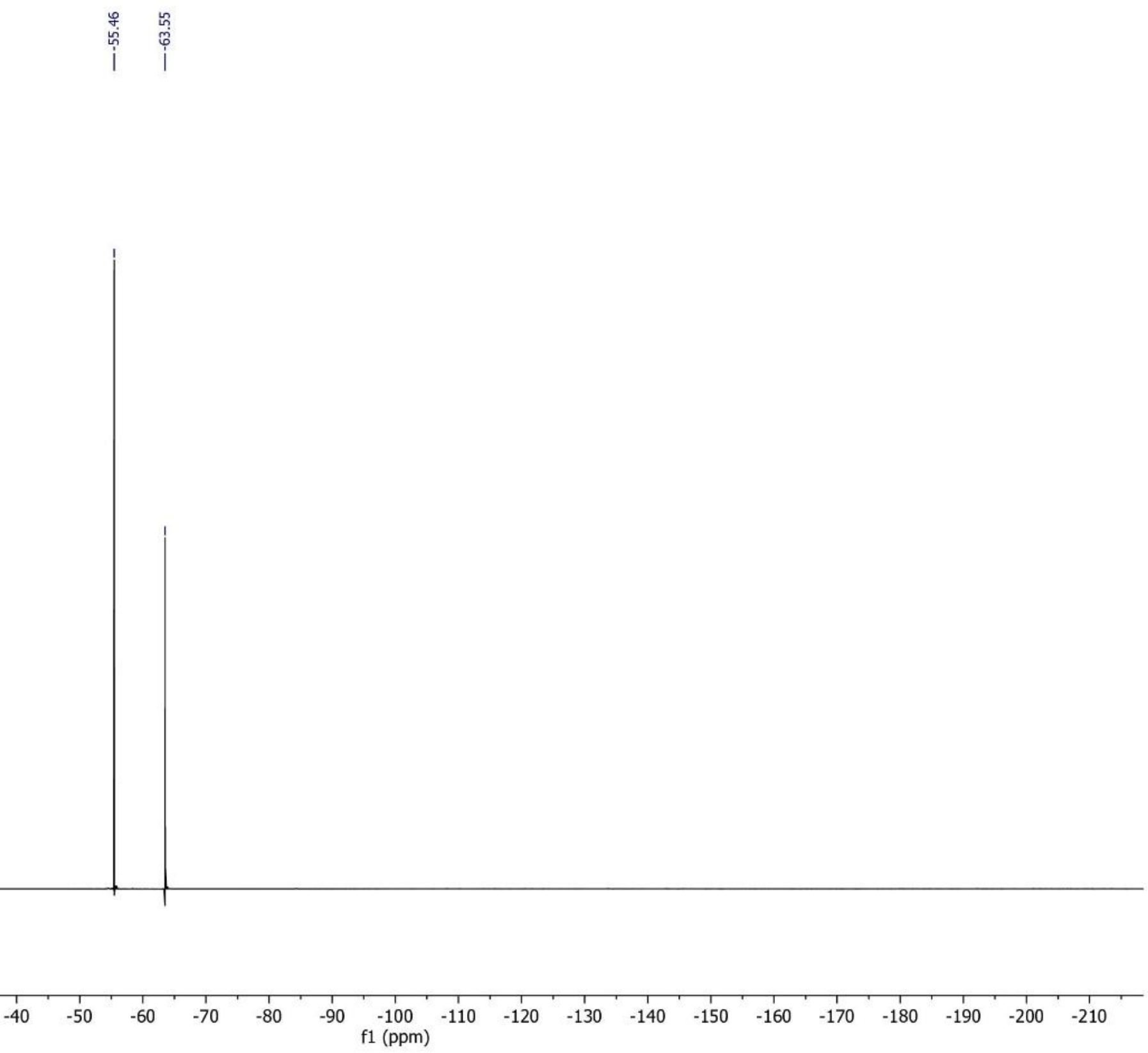

5-((4-((2,4,6-Tris(trifluoromethyl)phenyl)sulfonyl)-1,4-diazepan-1-yl)sulfonyl)isoquinoline (S22)

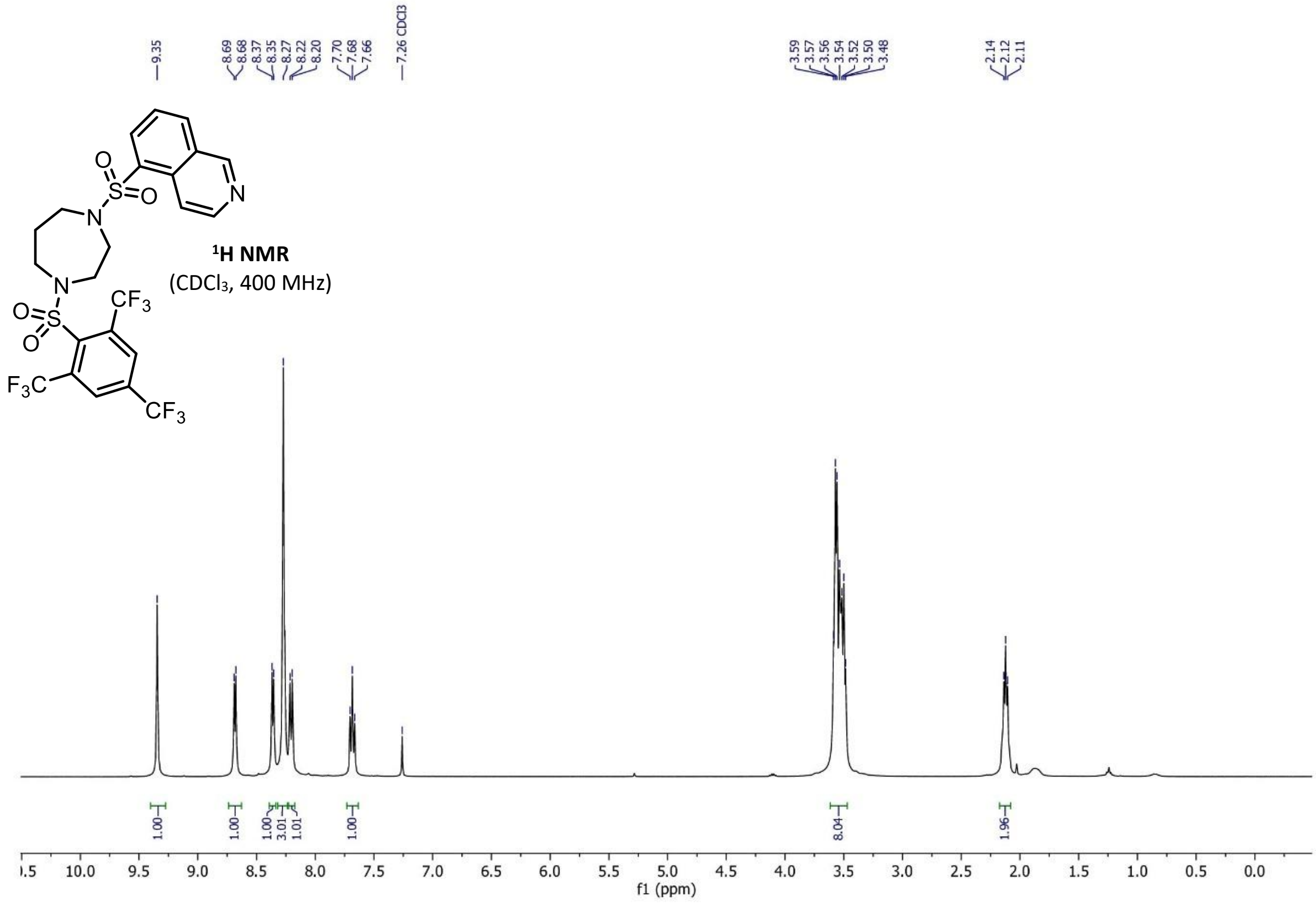

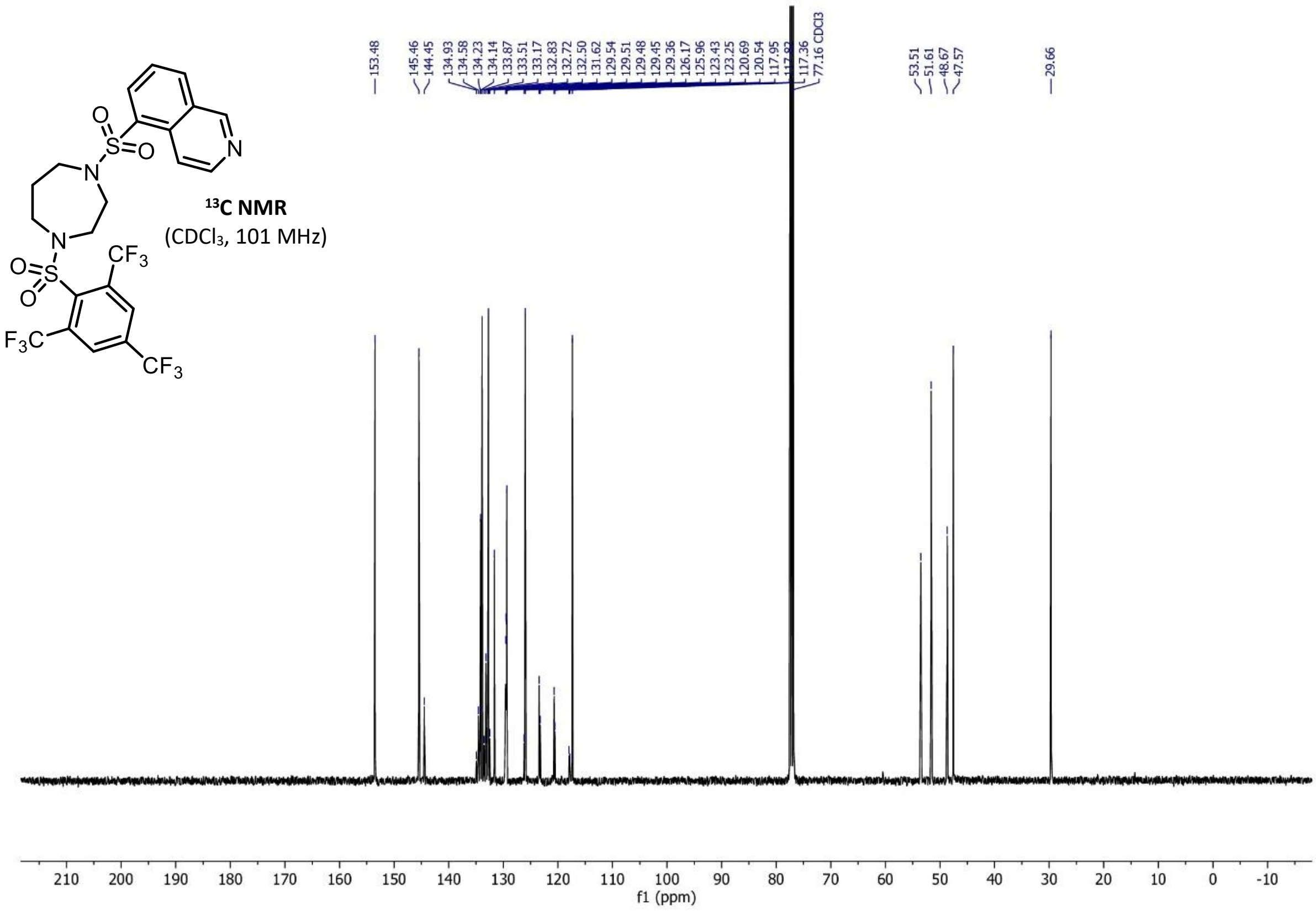

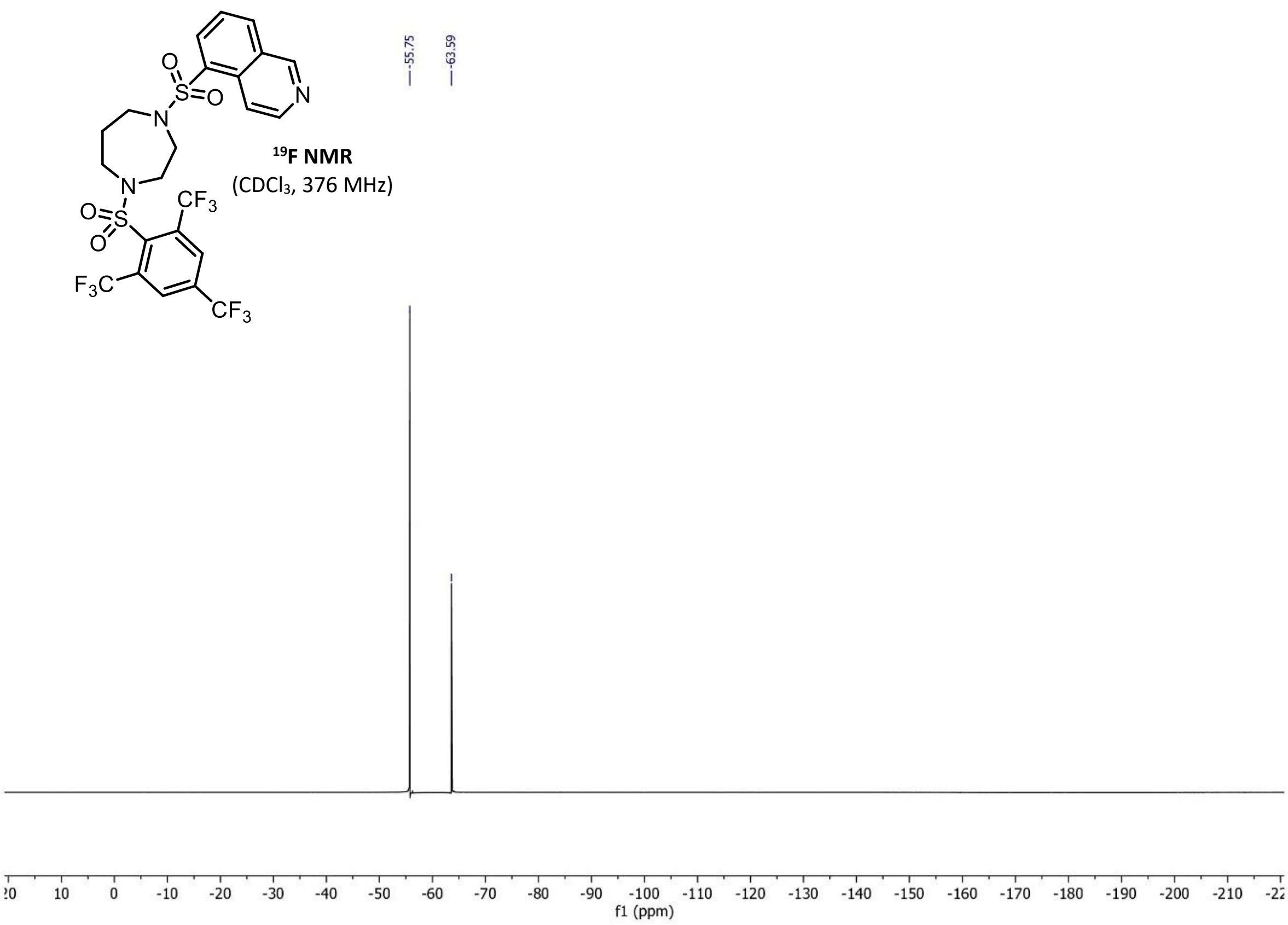

¹⁹F NMR
(CDCl₃, 376 MHz)
-55.75
-63.59
CF3
F3C
CF3
N
O
S
f1 (ppm)

***N*-Methyl-*N*-((5-phenyl-1-(pyridin-3-ylsulfonyl)-1*H*-pyrrol-3-yl)methyl)-2,4,6-tris(trifluoromethyl) benzenesulfonamide (S23)**

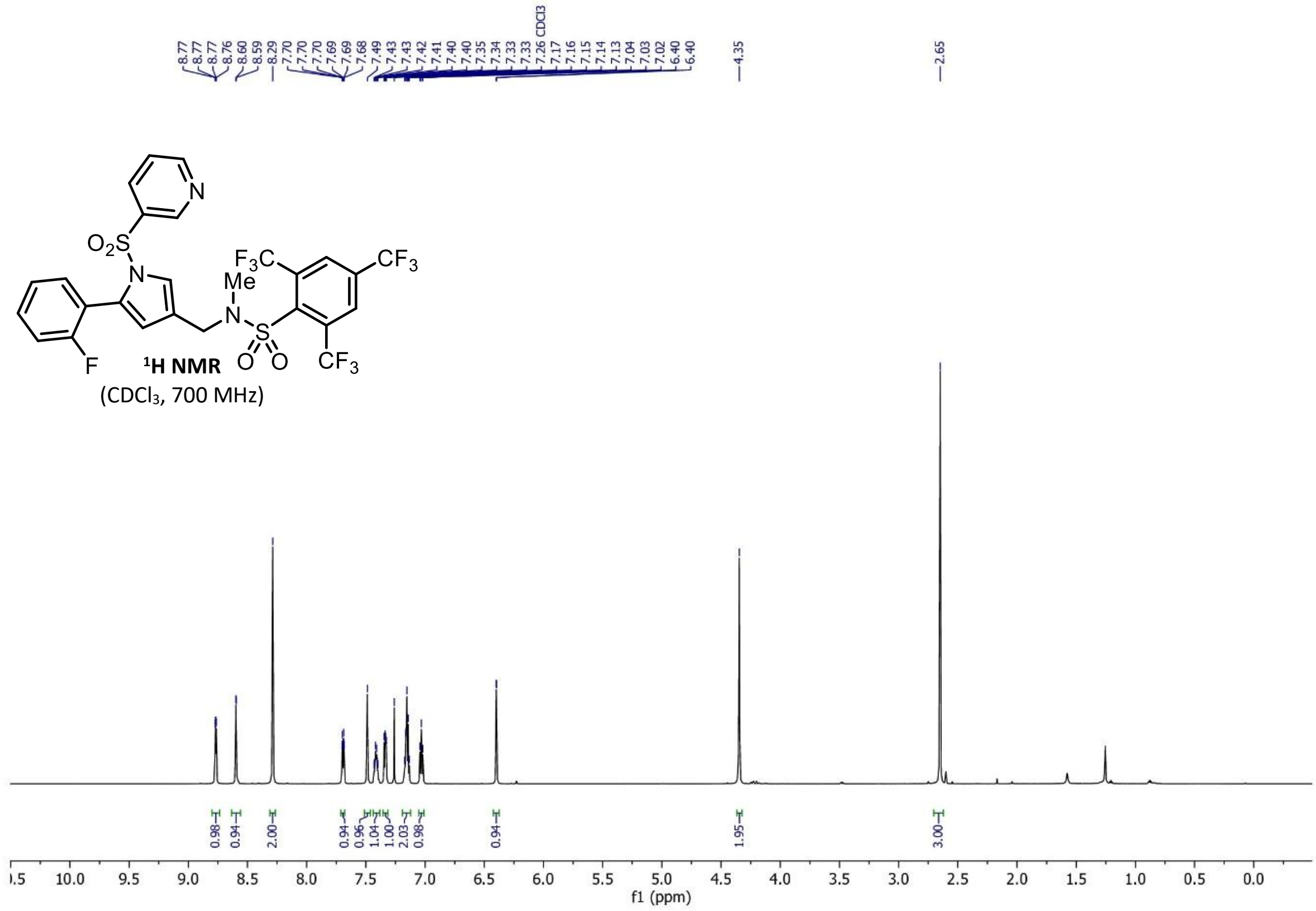

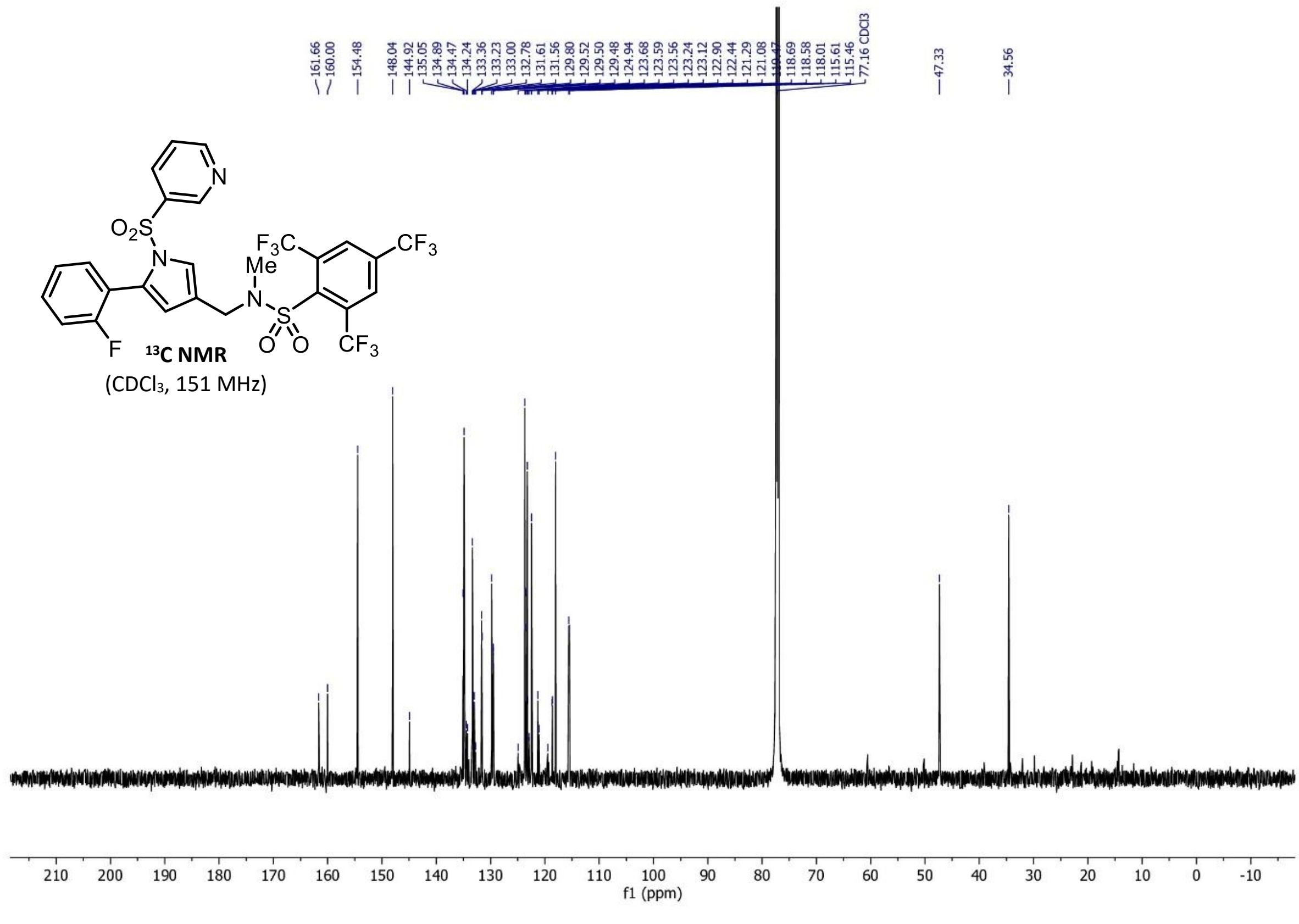

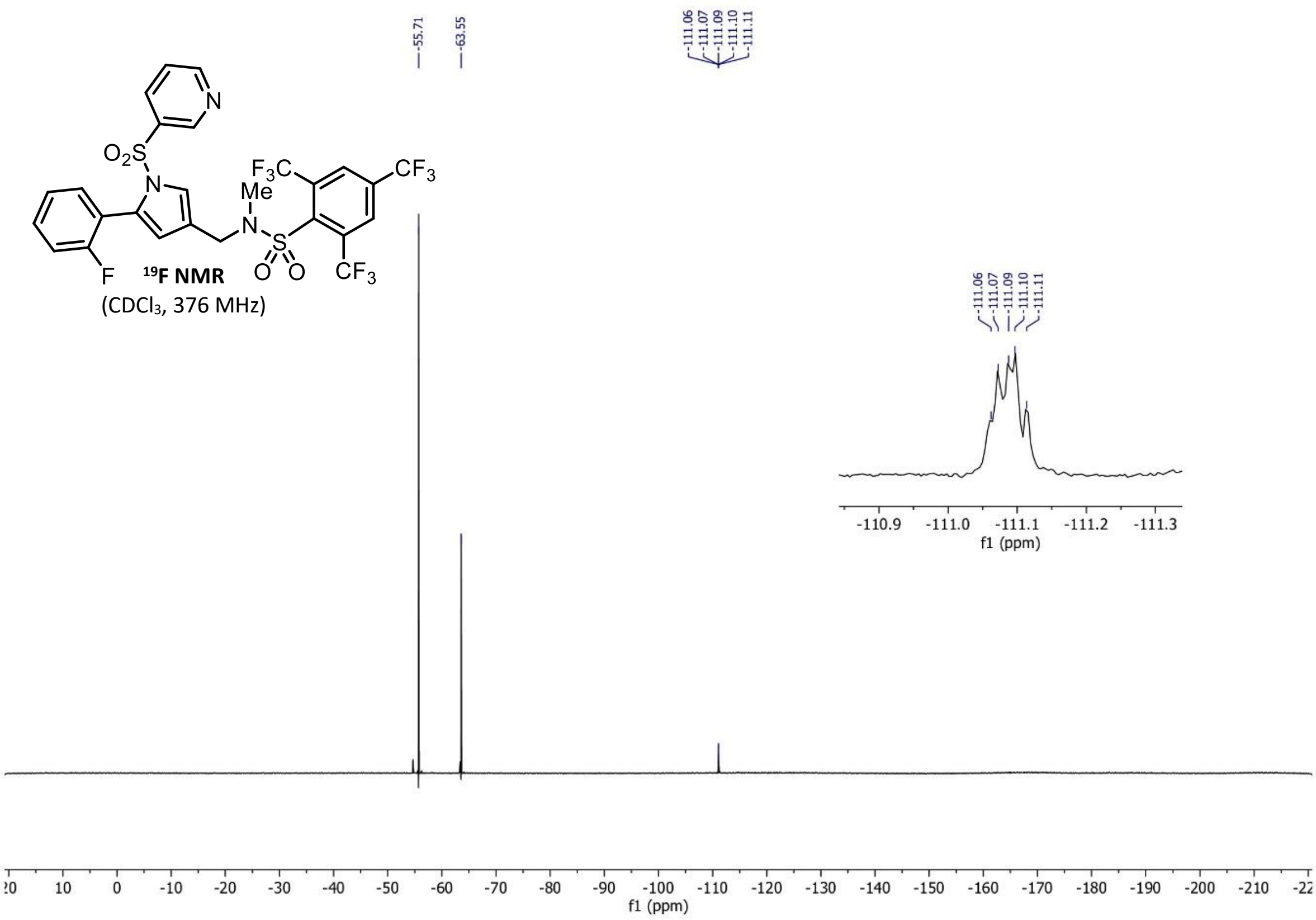

^{19}F NMR
(CDCl$_3$, 376 MHz)
-55.71
-63.55
-111.06
-111.07
-111.09
-111.10
-111.11
-110.9 -111.0 -111.1 -111.2 -111.3
f1 (ppm)
20 10 0 -10 -20 -30 -40 -50 -60 -70 -80 -90 -100 -110 -120 -130 -140 -150 -160 -170 -180 -190 -200 -210 -220
f1 (ppm)

***N*-(3,4-Dimethoxyphenethyl)-*N*-methyl-2,4-dinitrobenzenesulfonamide (14)**

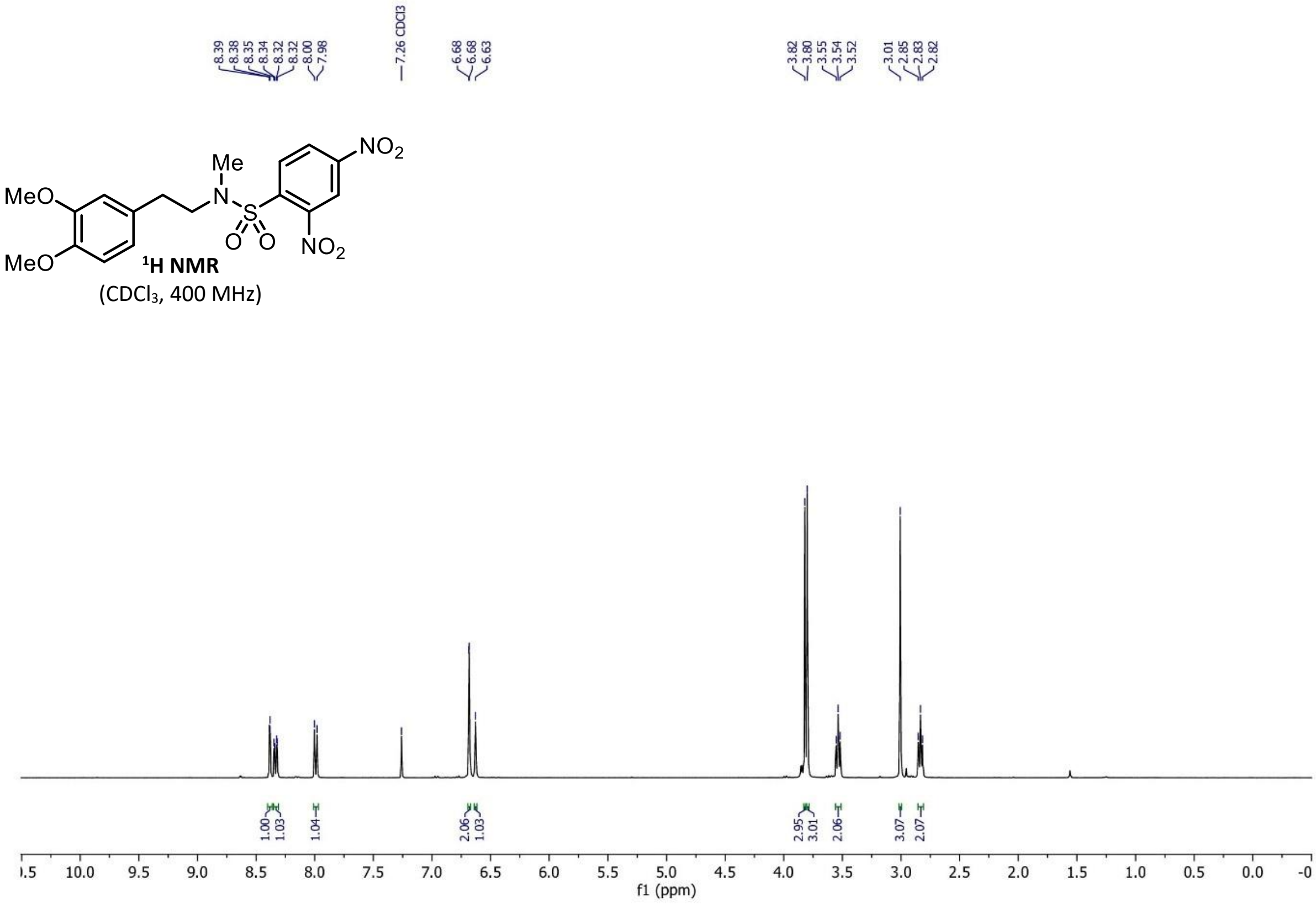

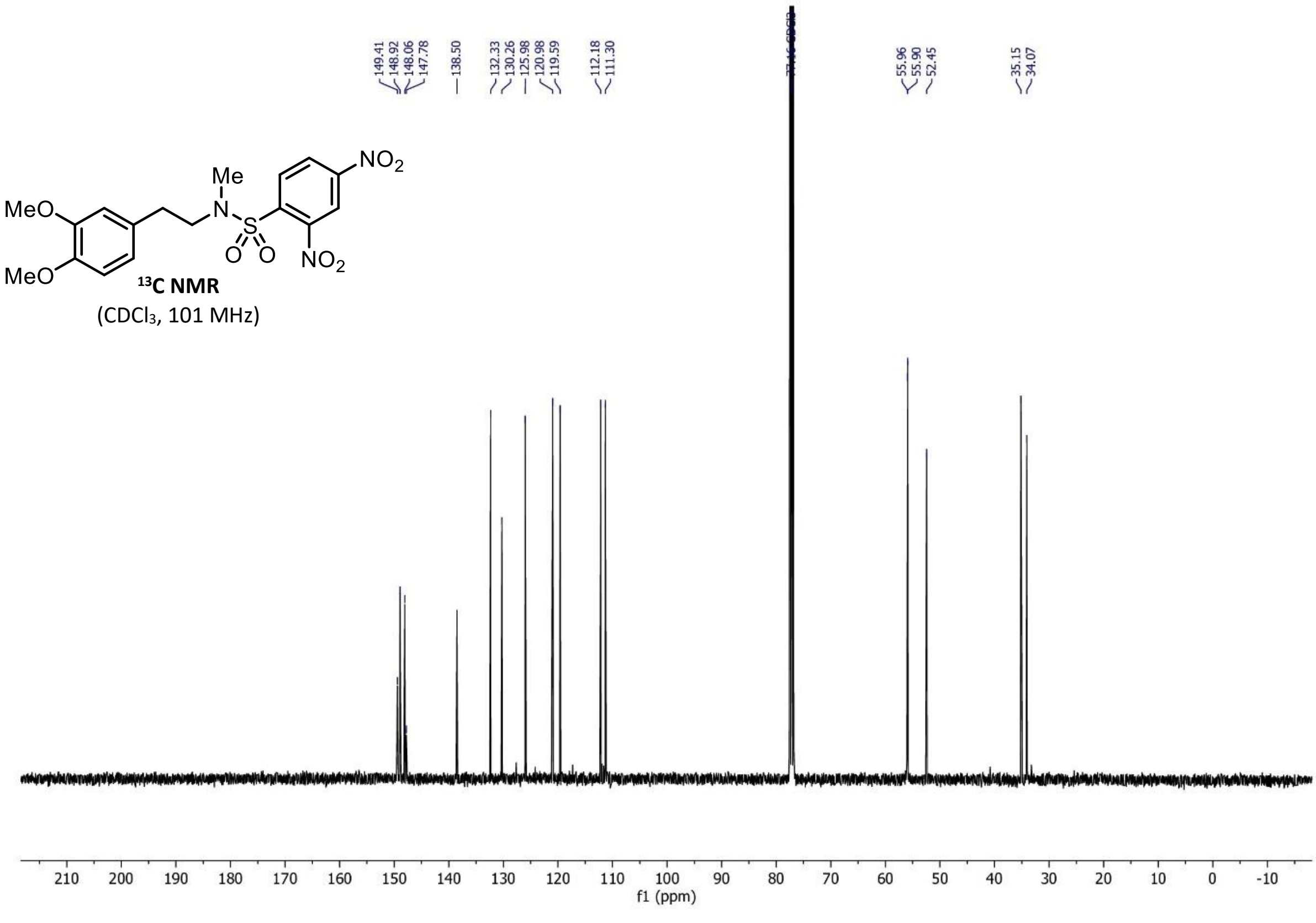

2-(3,4-Dimethoxyphenyl)-*N*,*N*-dimethylethan-1-amine (2a)

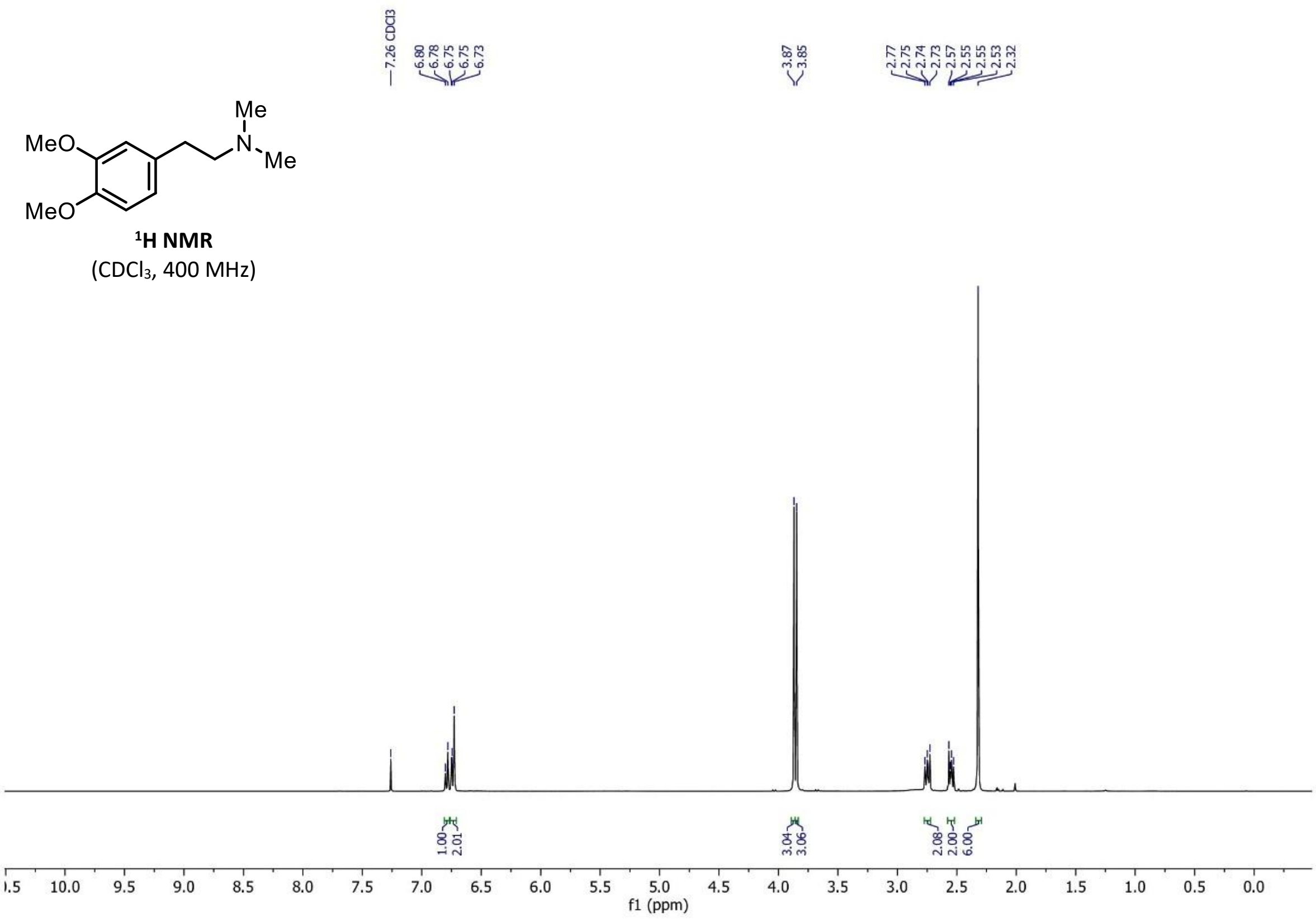

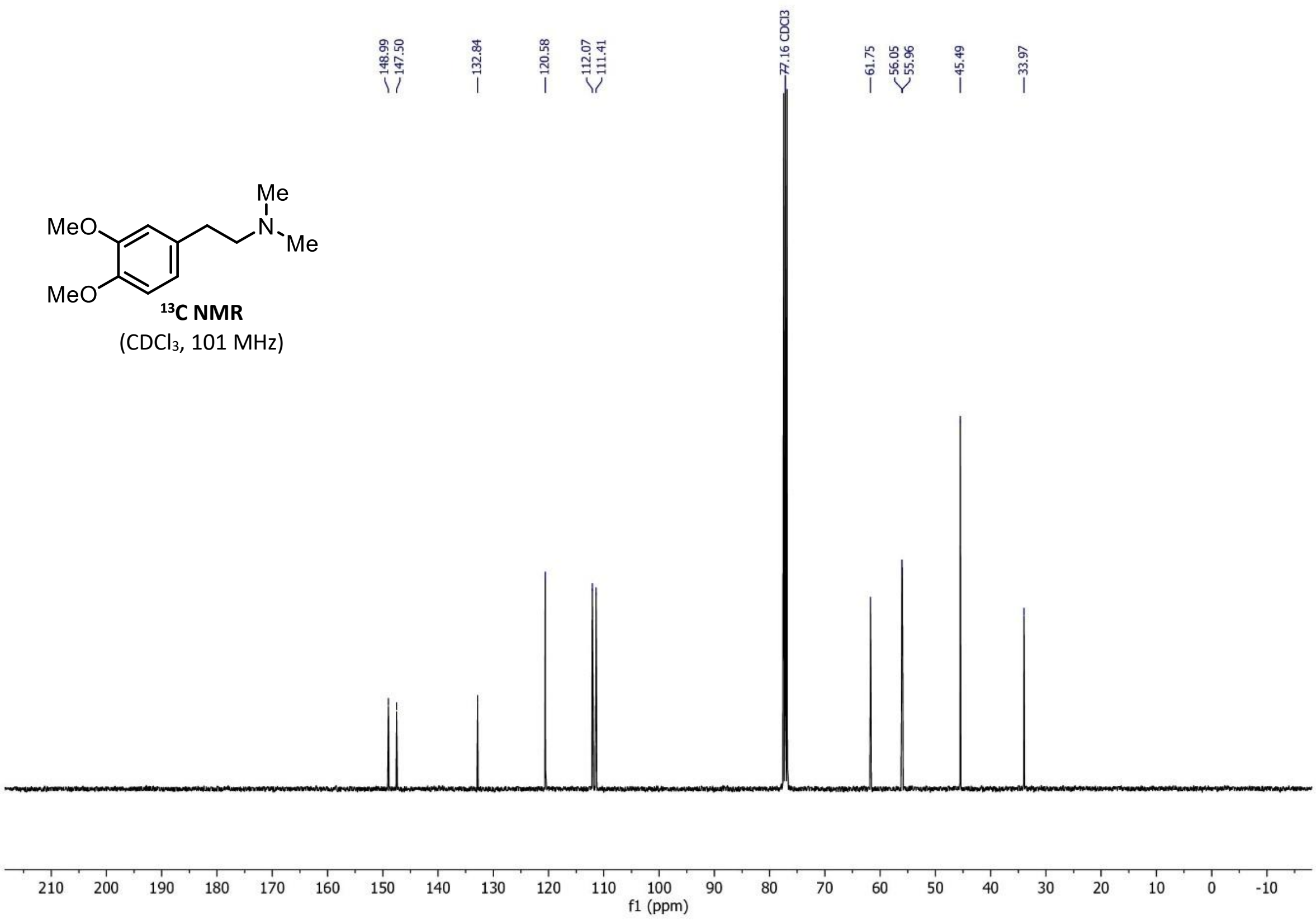

2-(3,4-Dimethoxyphenyl)-*N*-ethyl-*N*-methylethan-1-amine (2b)

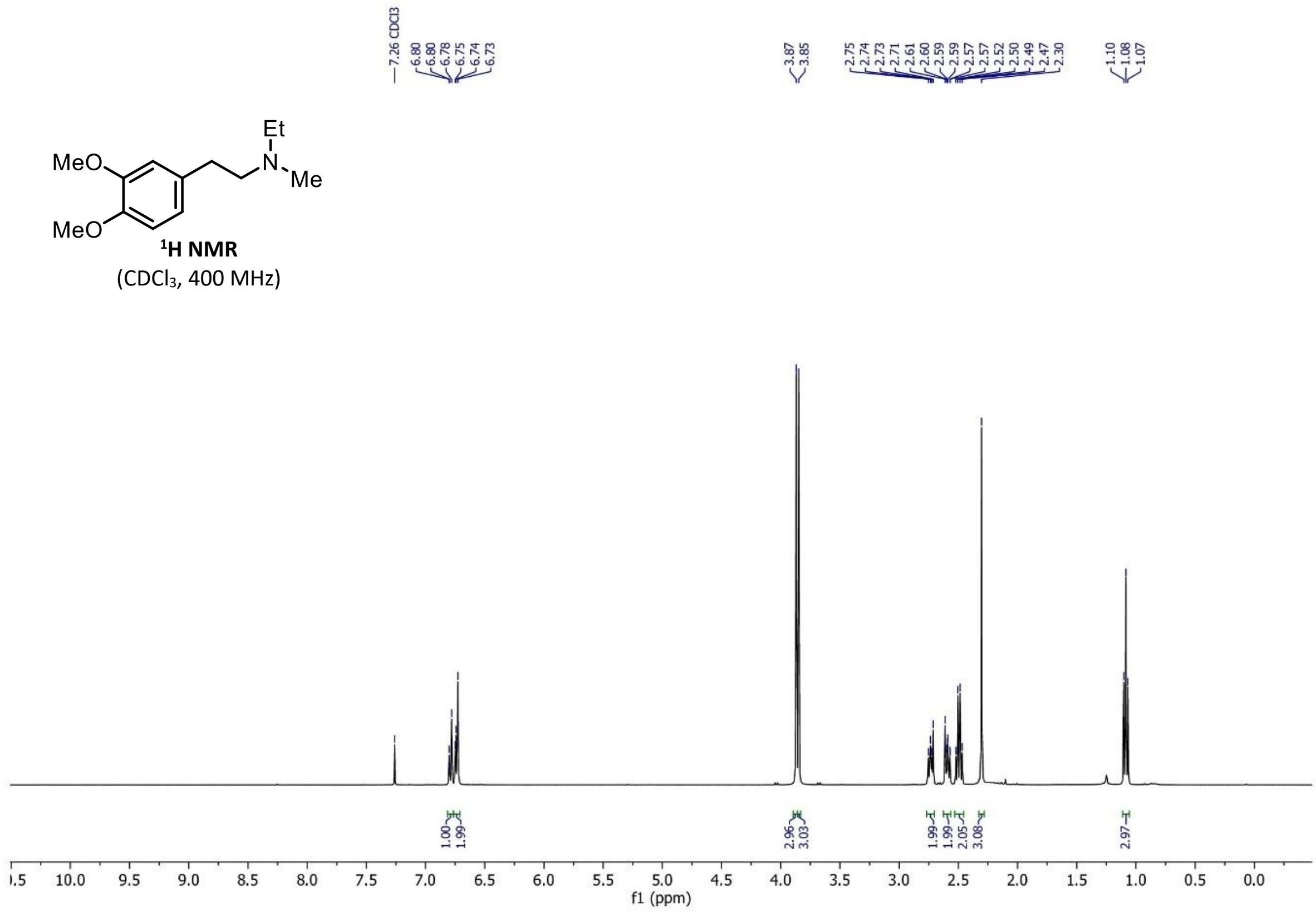

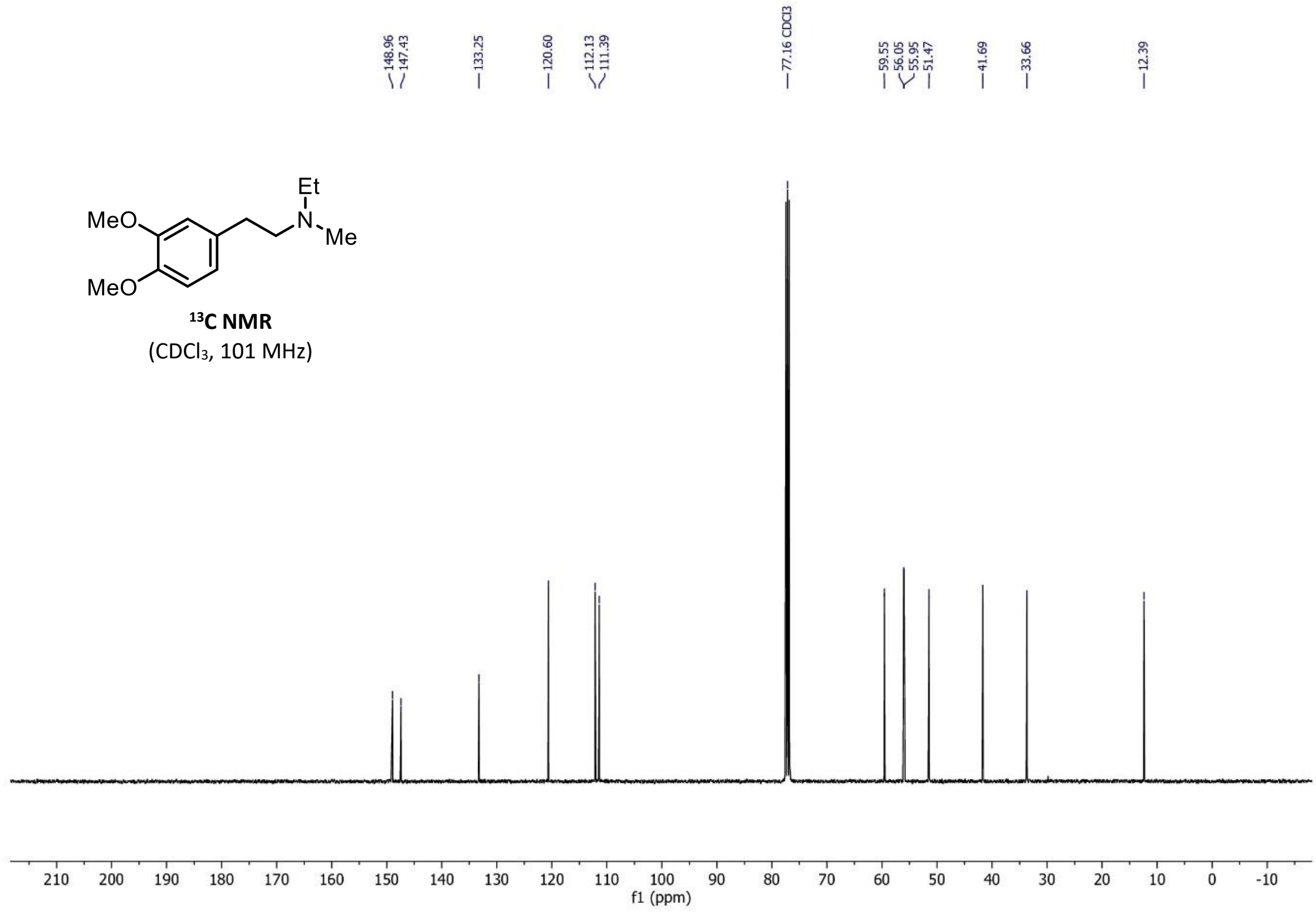

_N_-Benzyl-2-(3,4-dimethoxyphenyl)-_N_-methylethan-1-amine (2c)

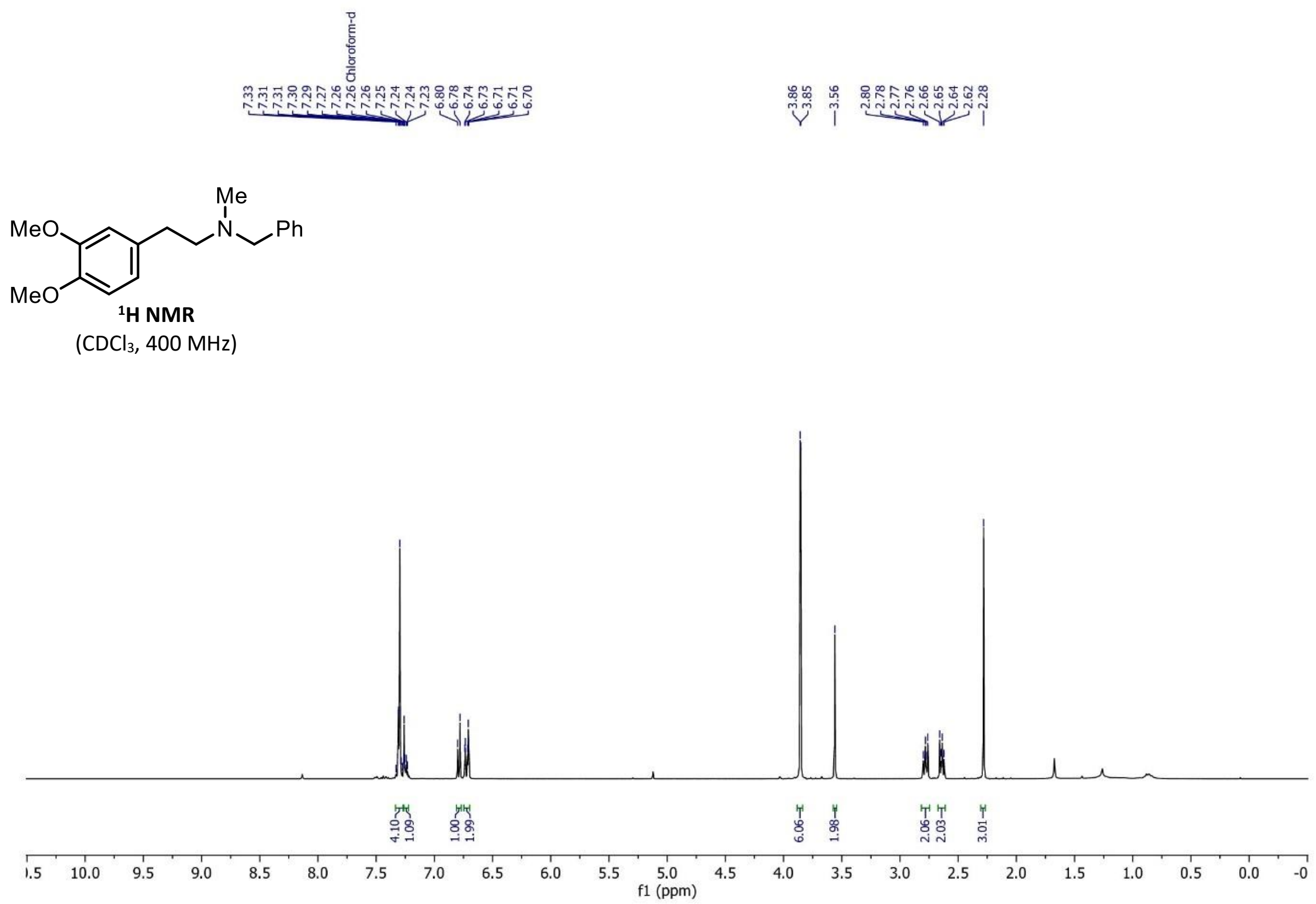

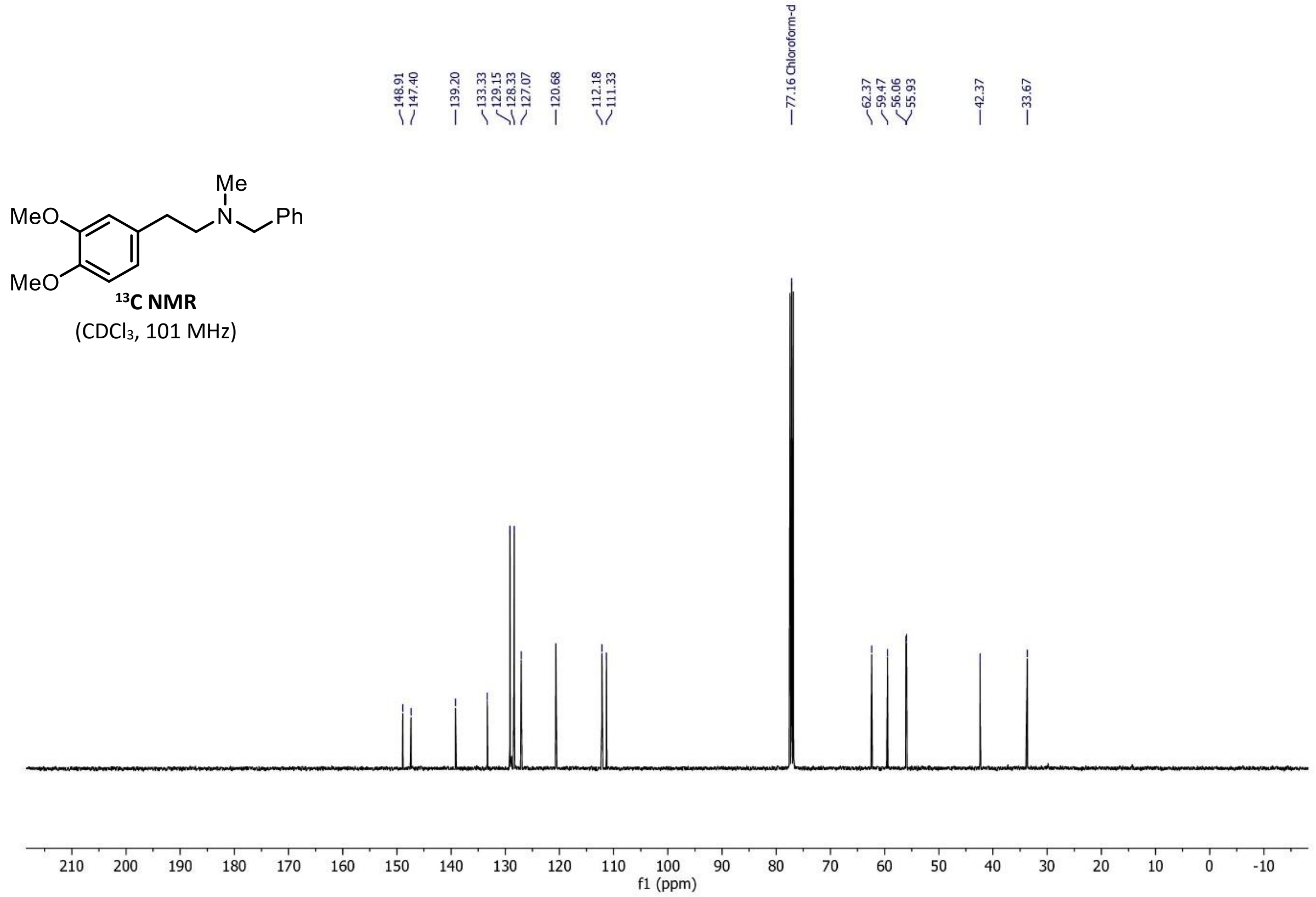

_N_-(4-Bromobenzyl)-2-(3,4-dimethoxyphenyl)-_N_-methylethan-1-amine (2d)

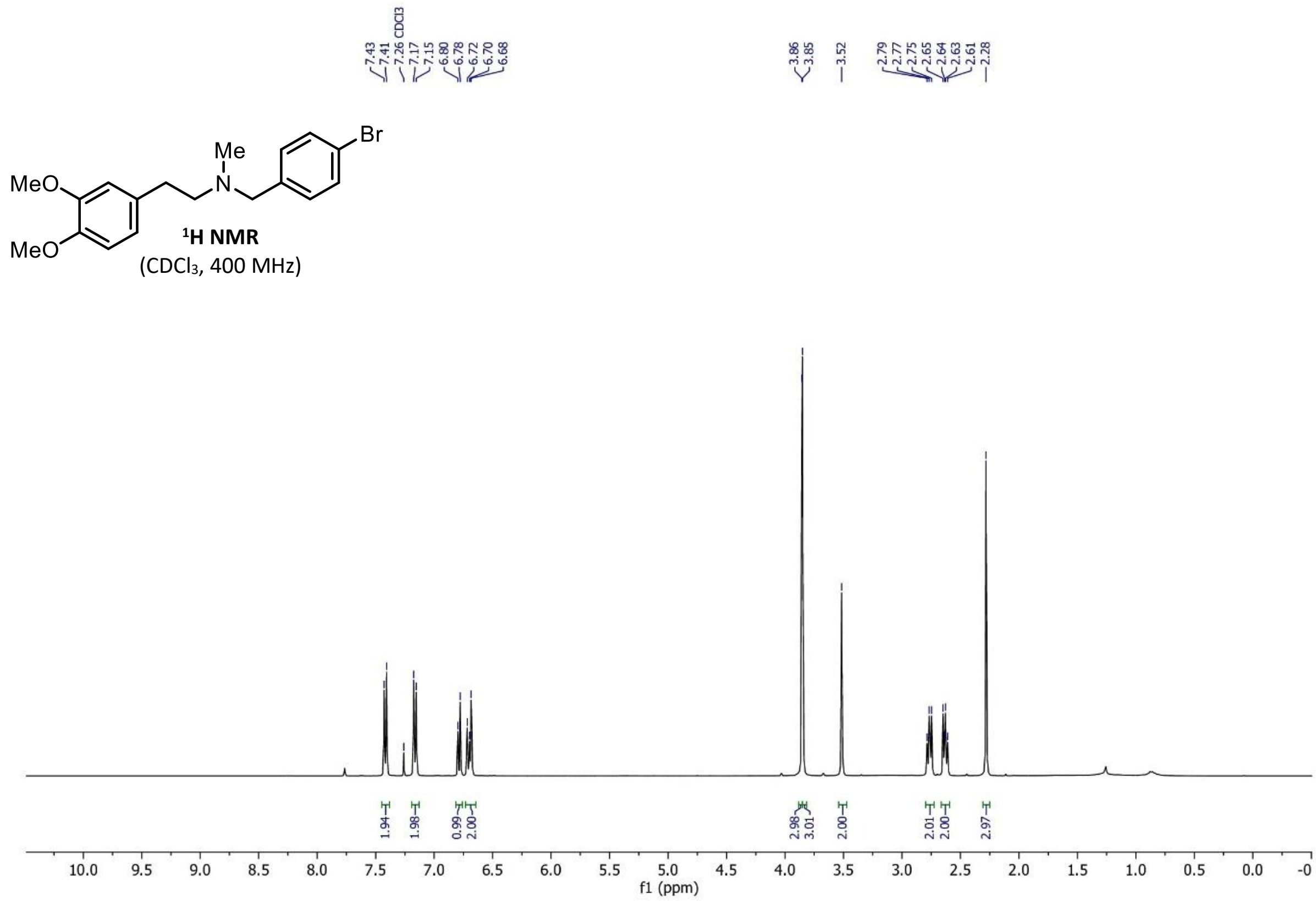

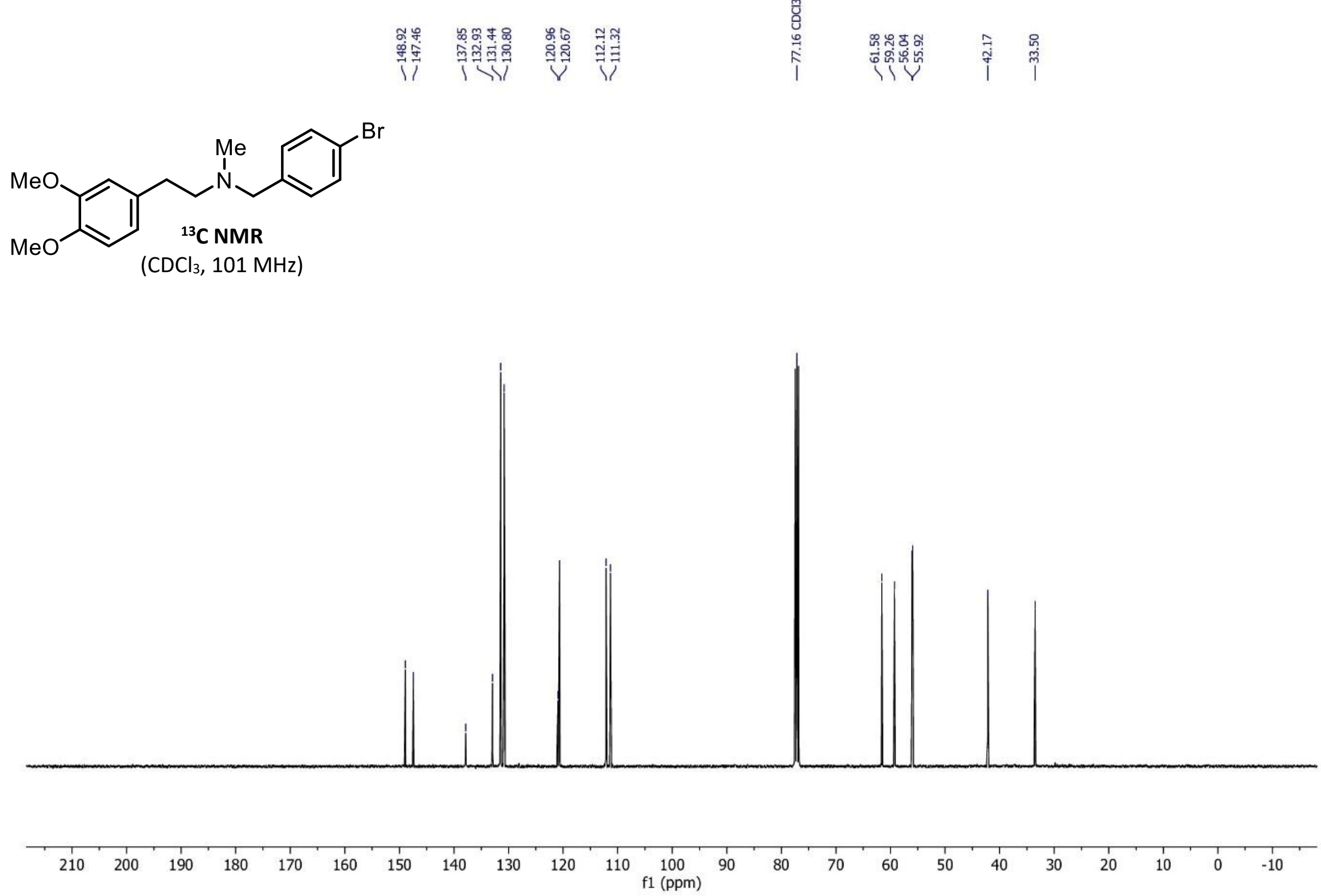

2-(3,4-Dimethoxyphenyl)-*N*-methyl-*N*-(4-(trimethylsilyl)benzyl)ethan-1-amine (2e)

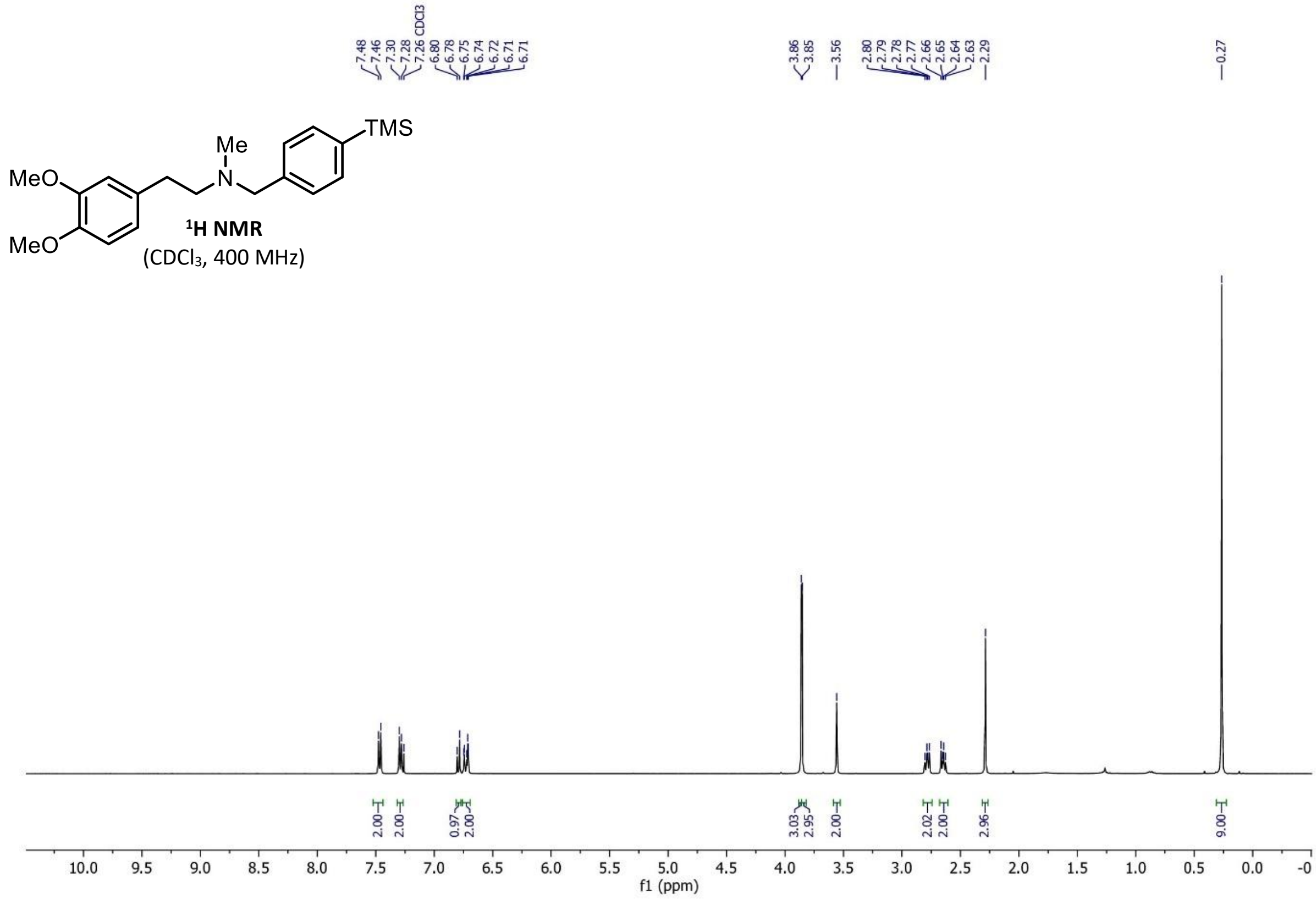

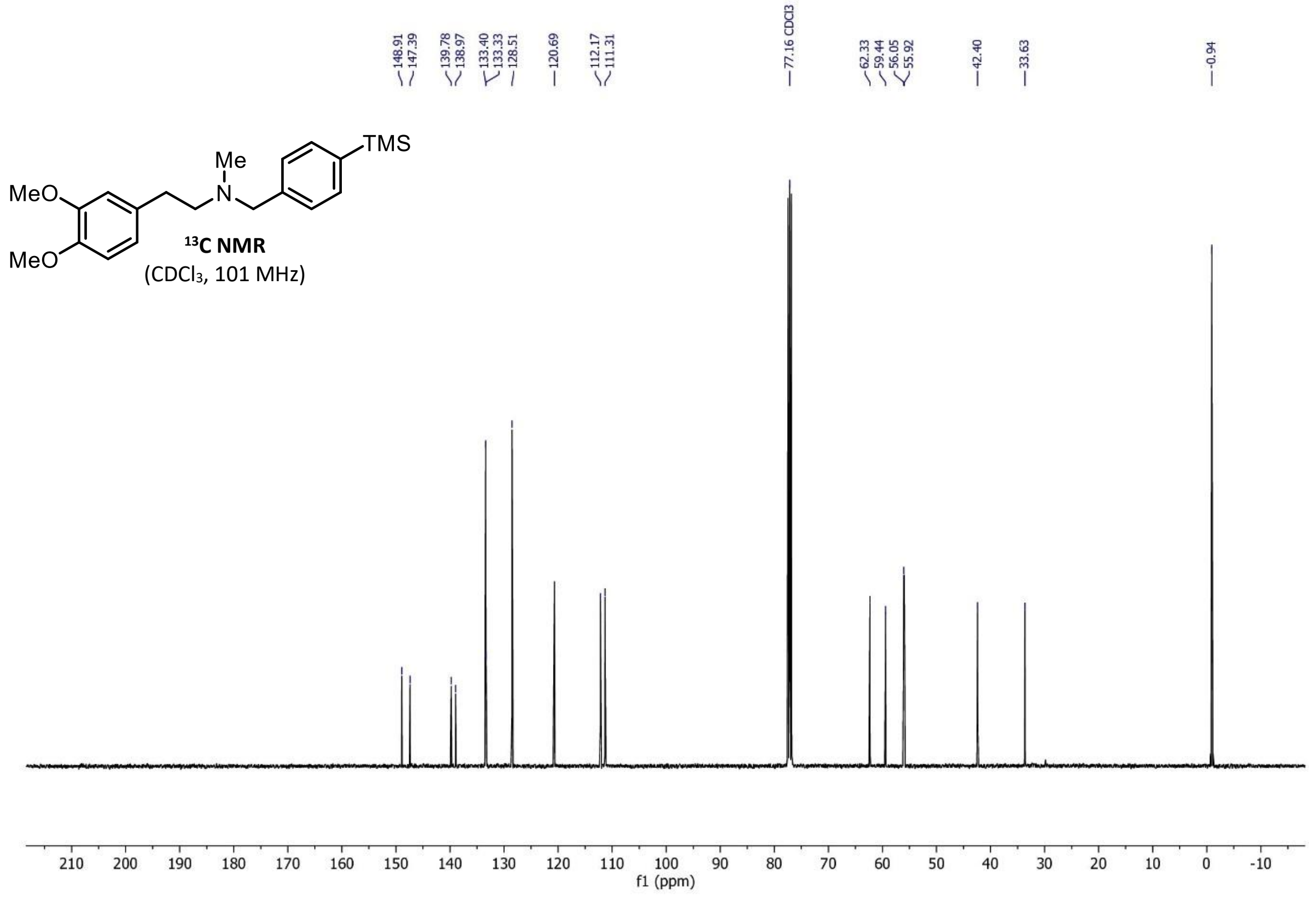

4-(((3,4-Dimethoxyphenethyl)(methyl)amino)methyl)benzaldehyde (2f)

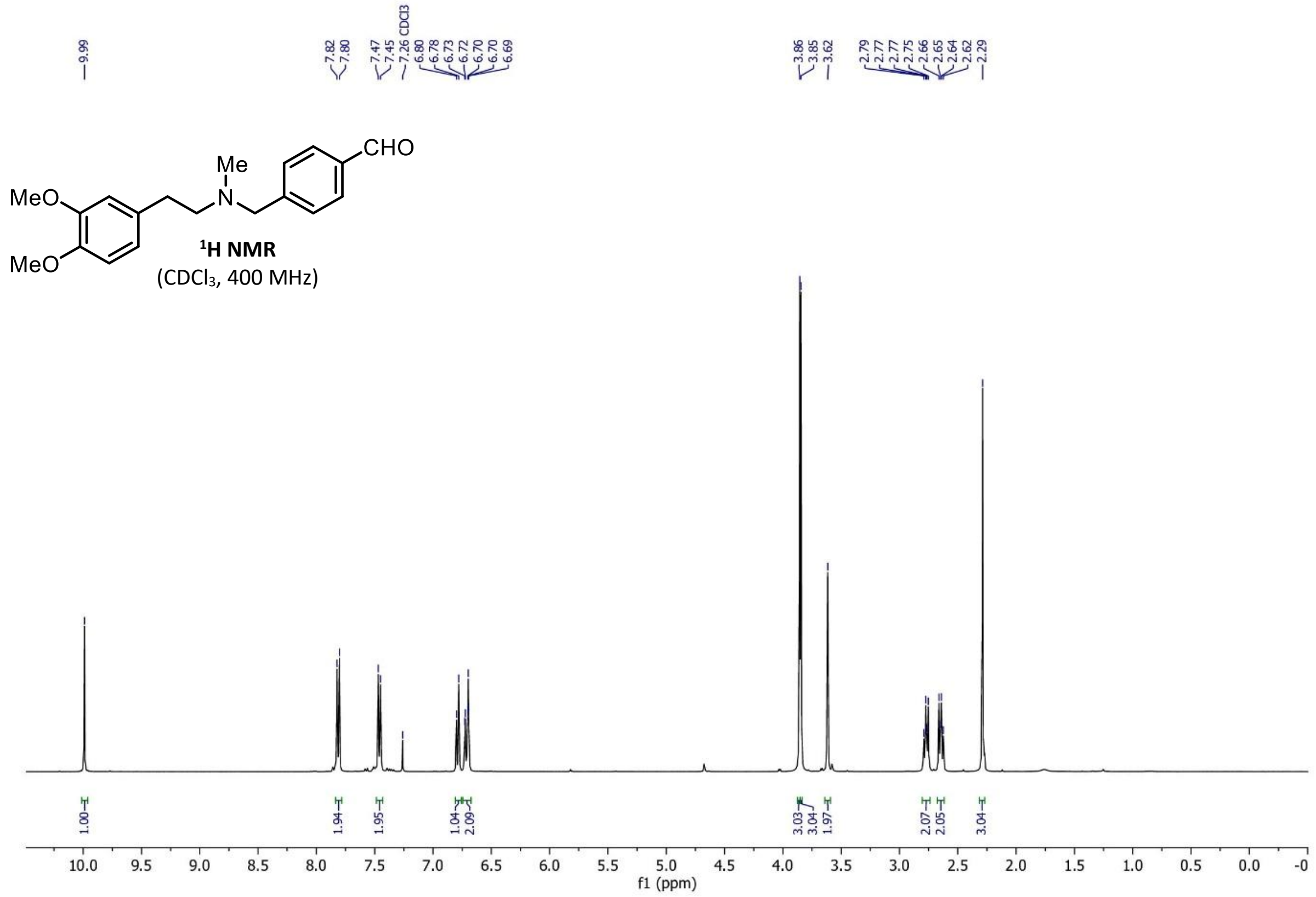

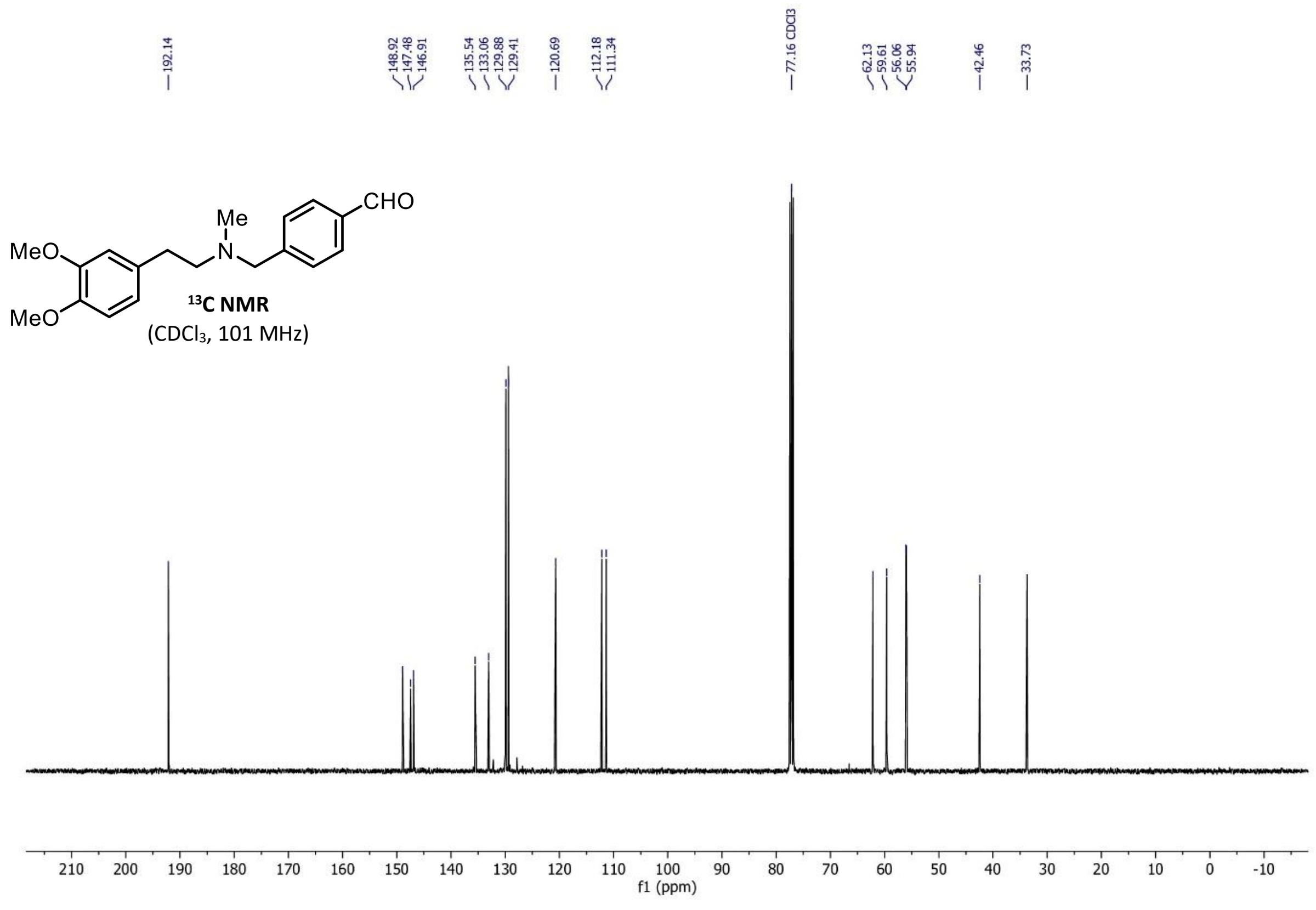

(*E*)-*N*-(3,4-Dimethoxyphenethyl)-*N*,3,7-trimethylocta-2,6-dien-1-amine (2g)

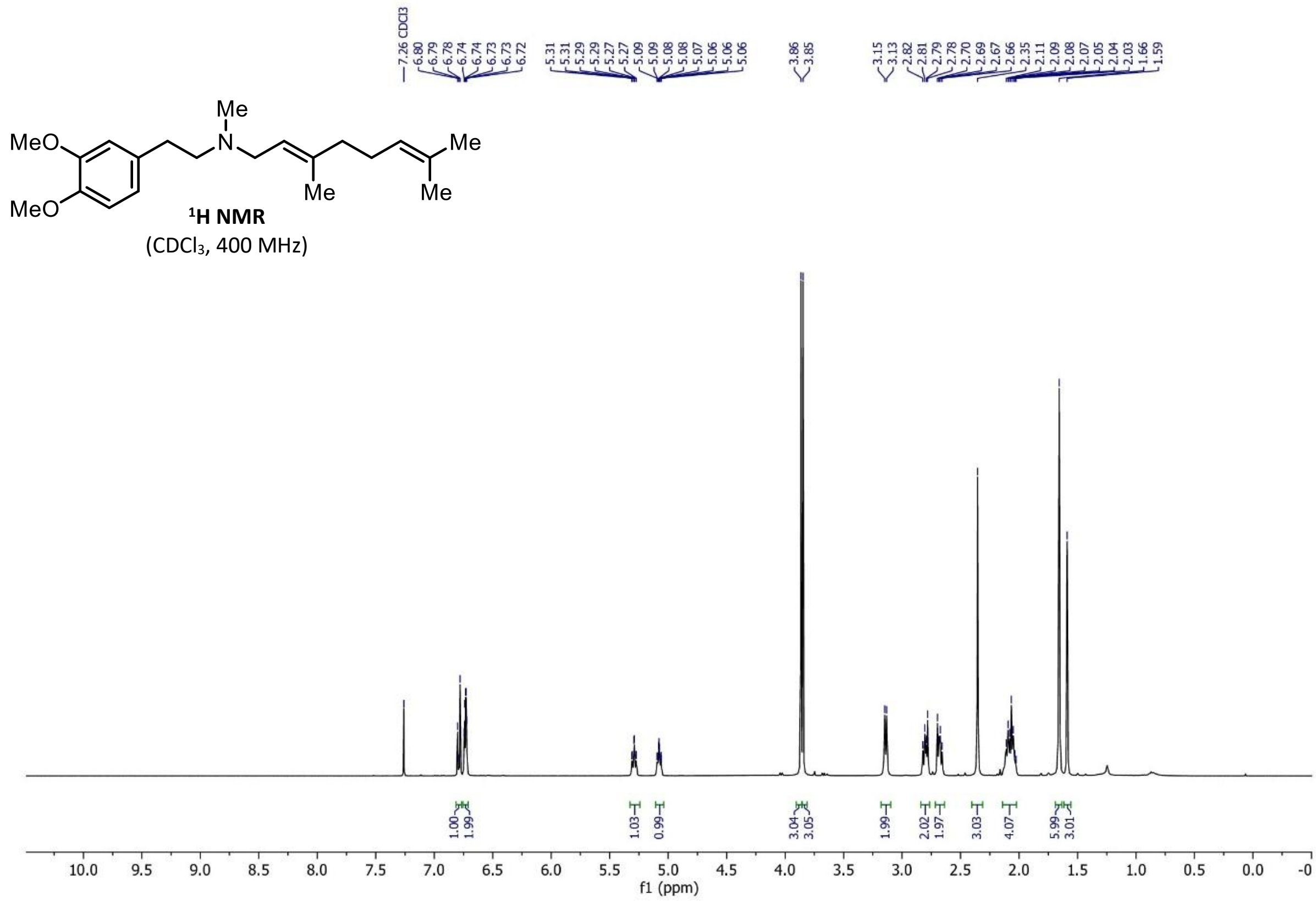

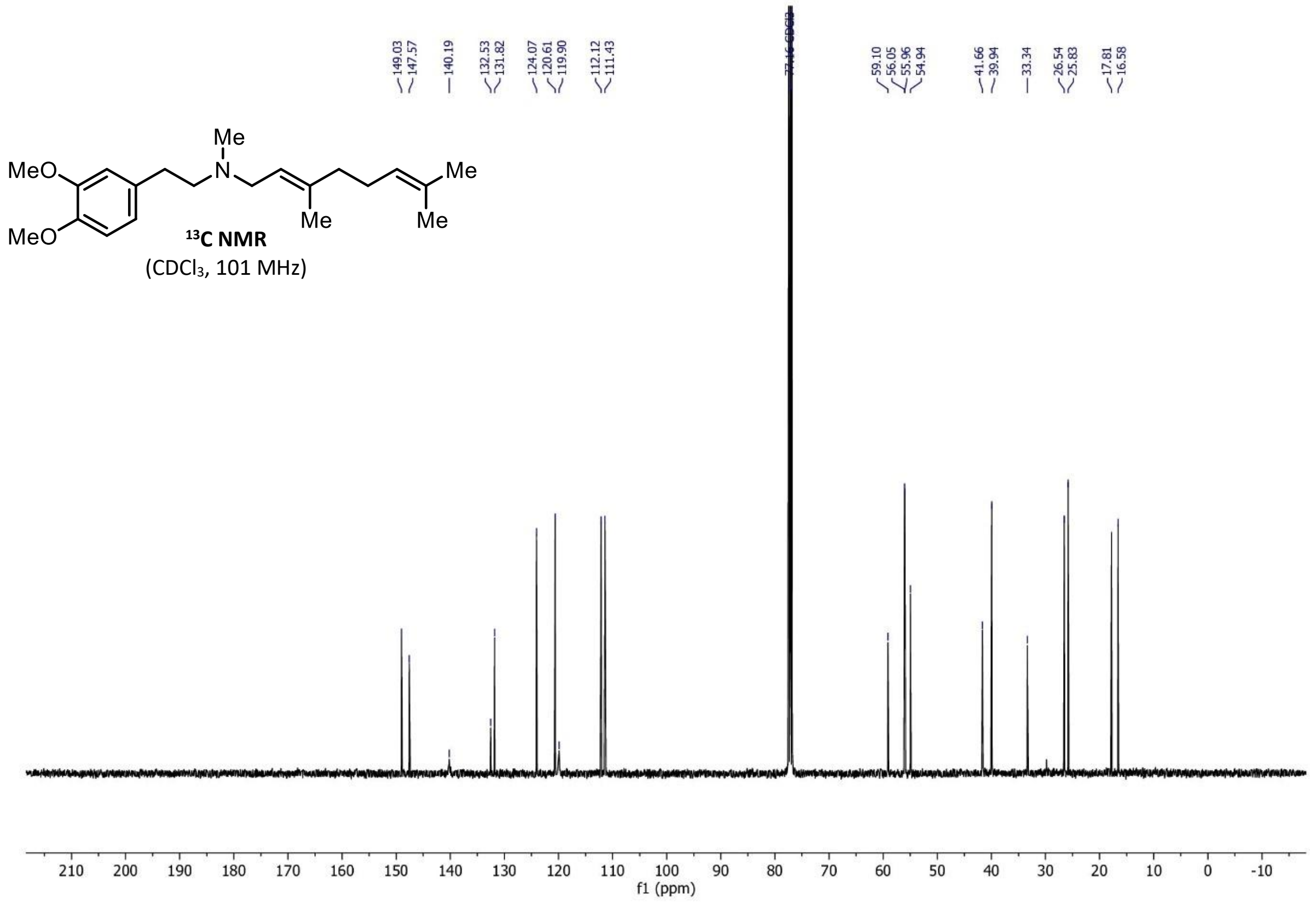

***N*-(3,4-Dimethoxyphenethyl)-*N*-methyl-3-phenylpropan-1-amine (2h)**

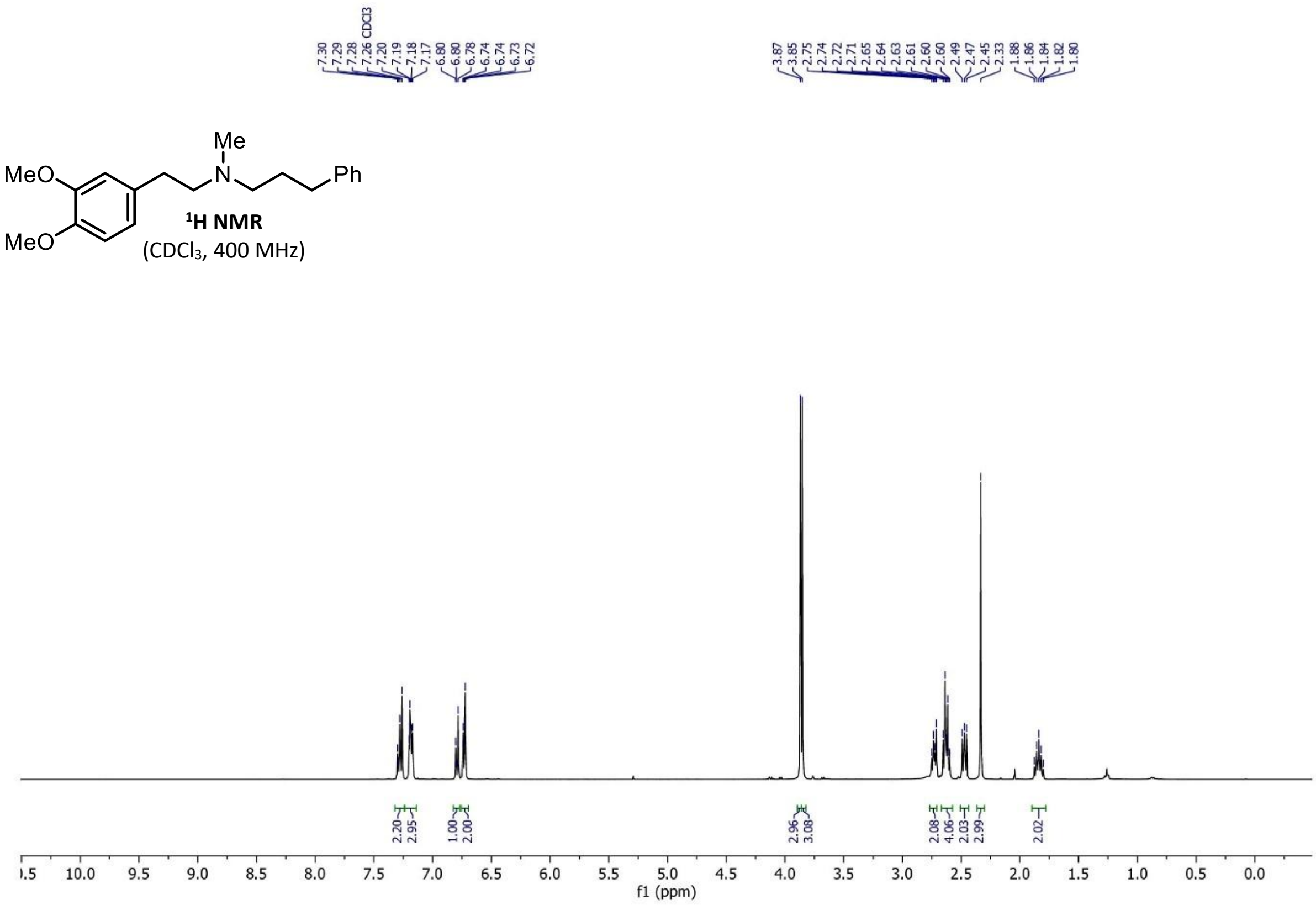

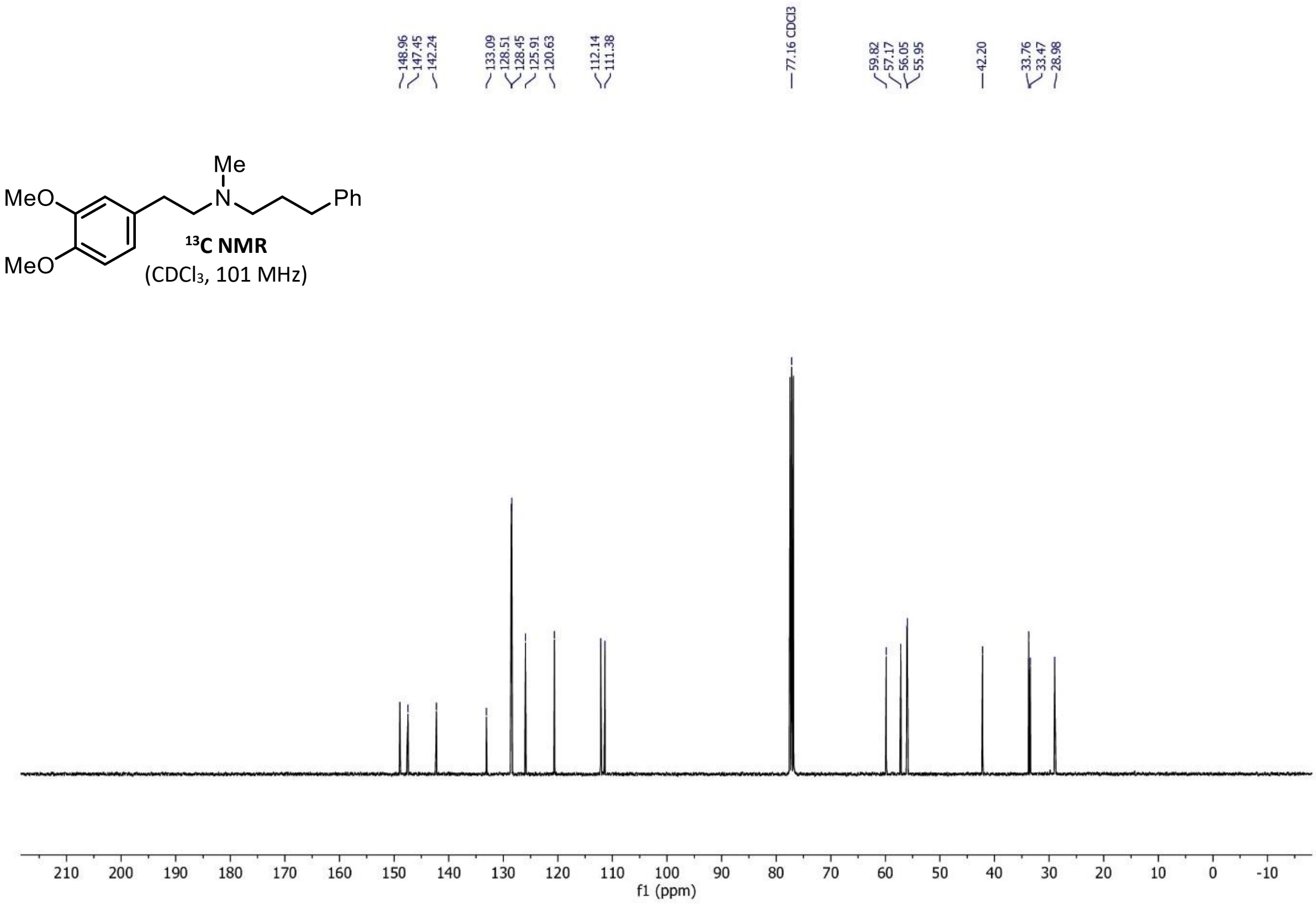

***N*-(2-(1*H*-Indol-3-yl)ethyl)-2-(3,4-dimethoxyphenyl)-*N*-methylethan-1-amine (2i)**

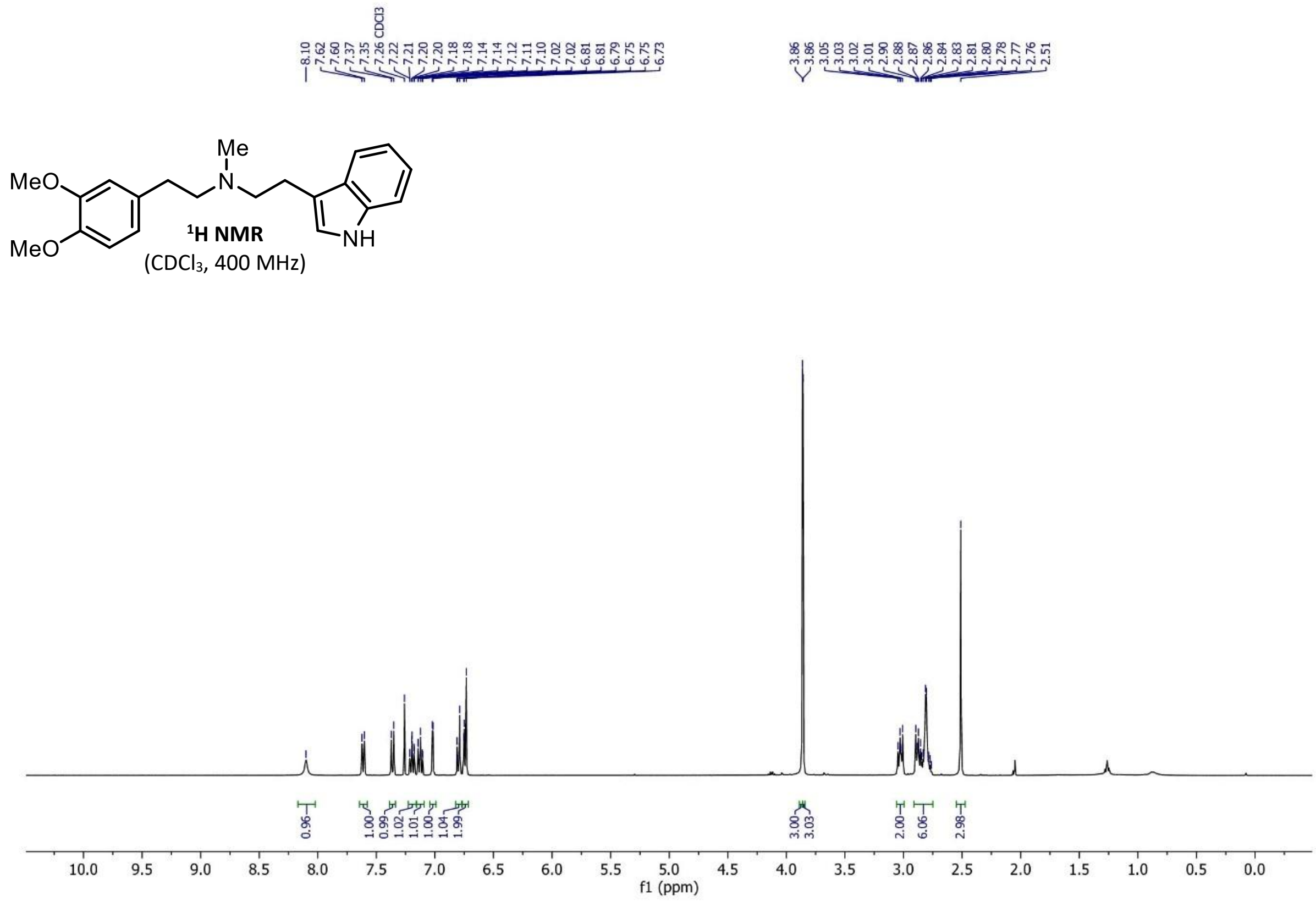

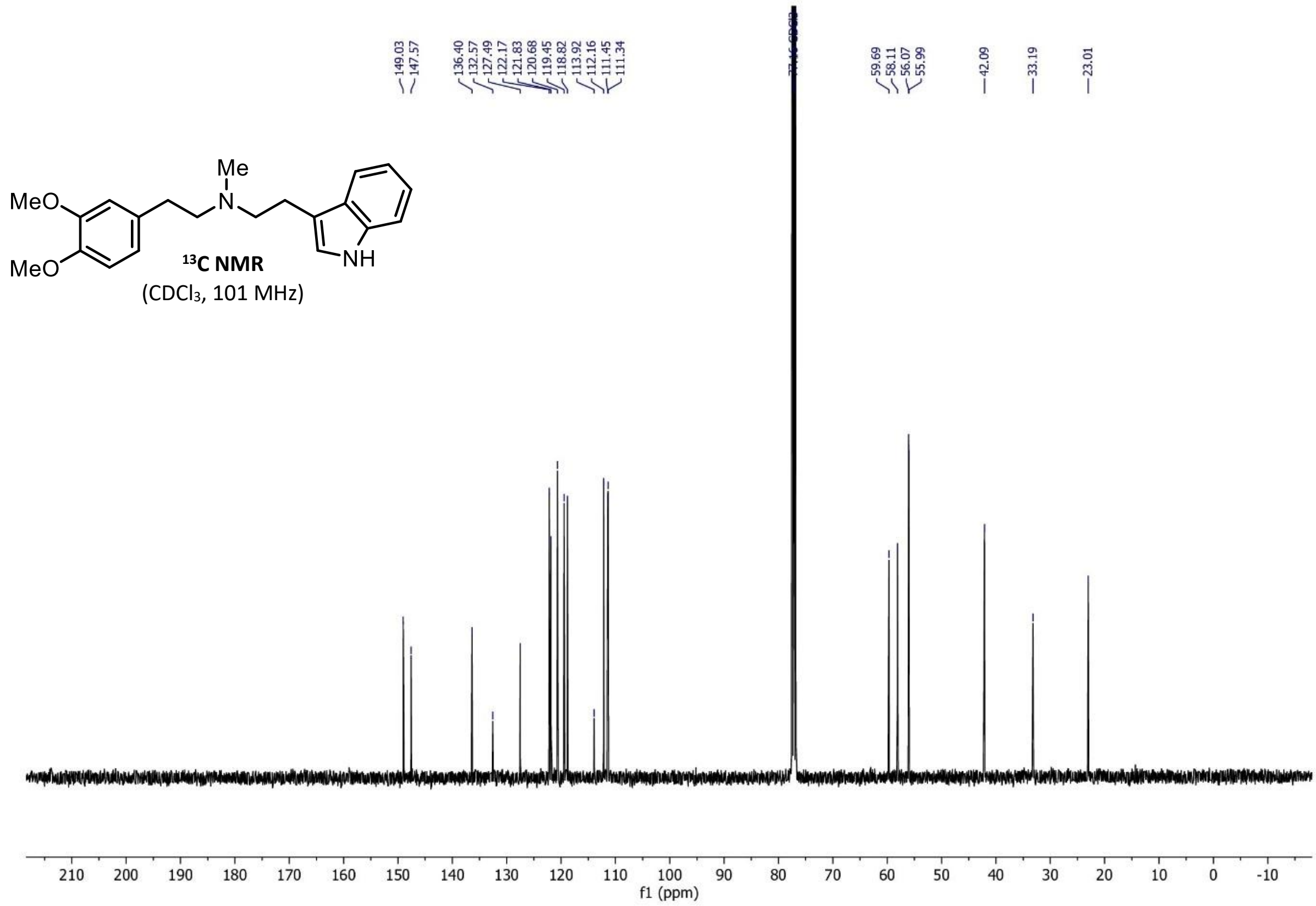

4-((*tert*-Butyldimethylsilyl)oxy)-*N*-(3,4-dimethoxyphenethyl)-*N*-methylbutan-1-amine (2j)

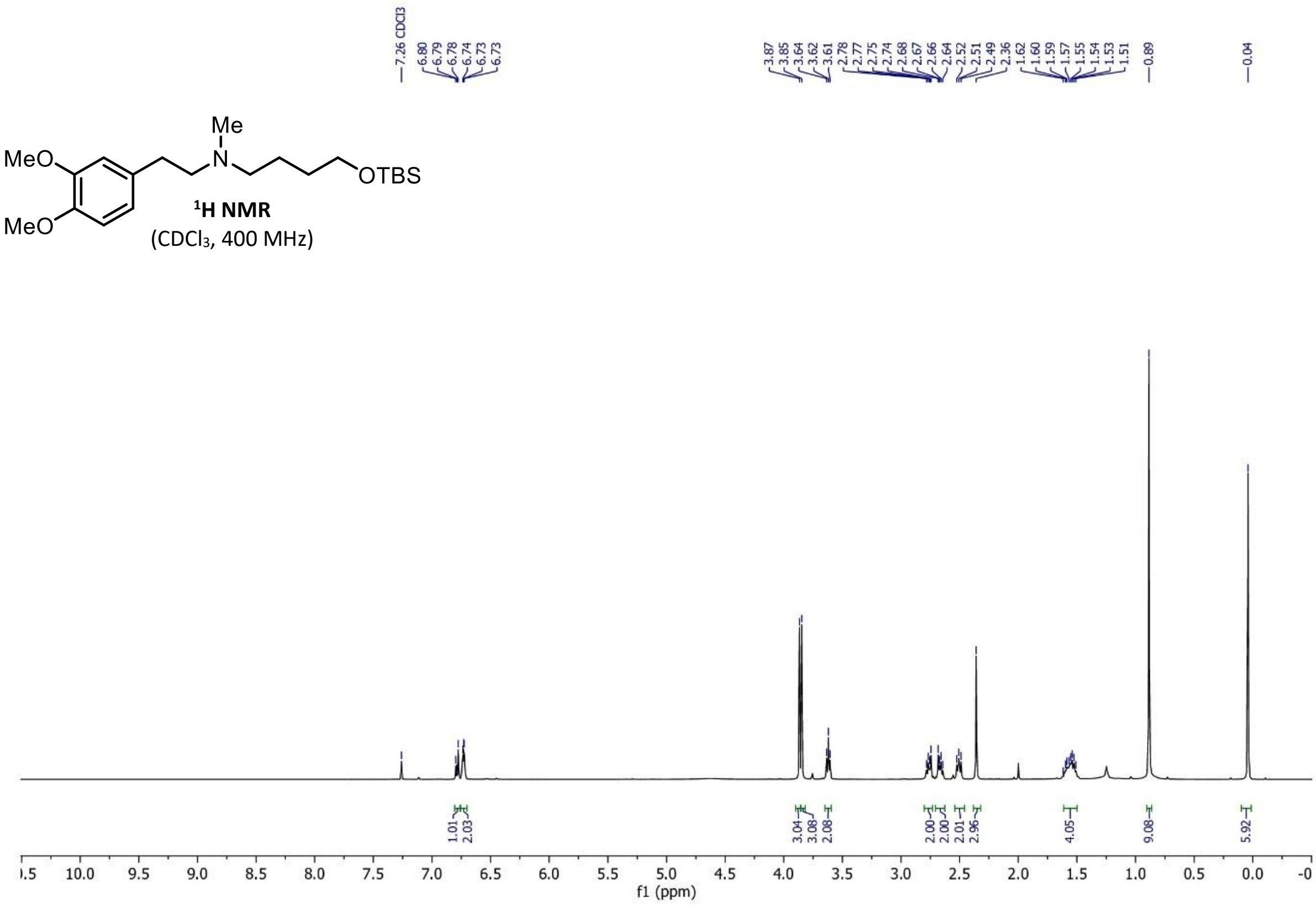

^{1}H NMR

($CDCl_3$, 400 MHz)

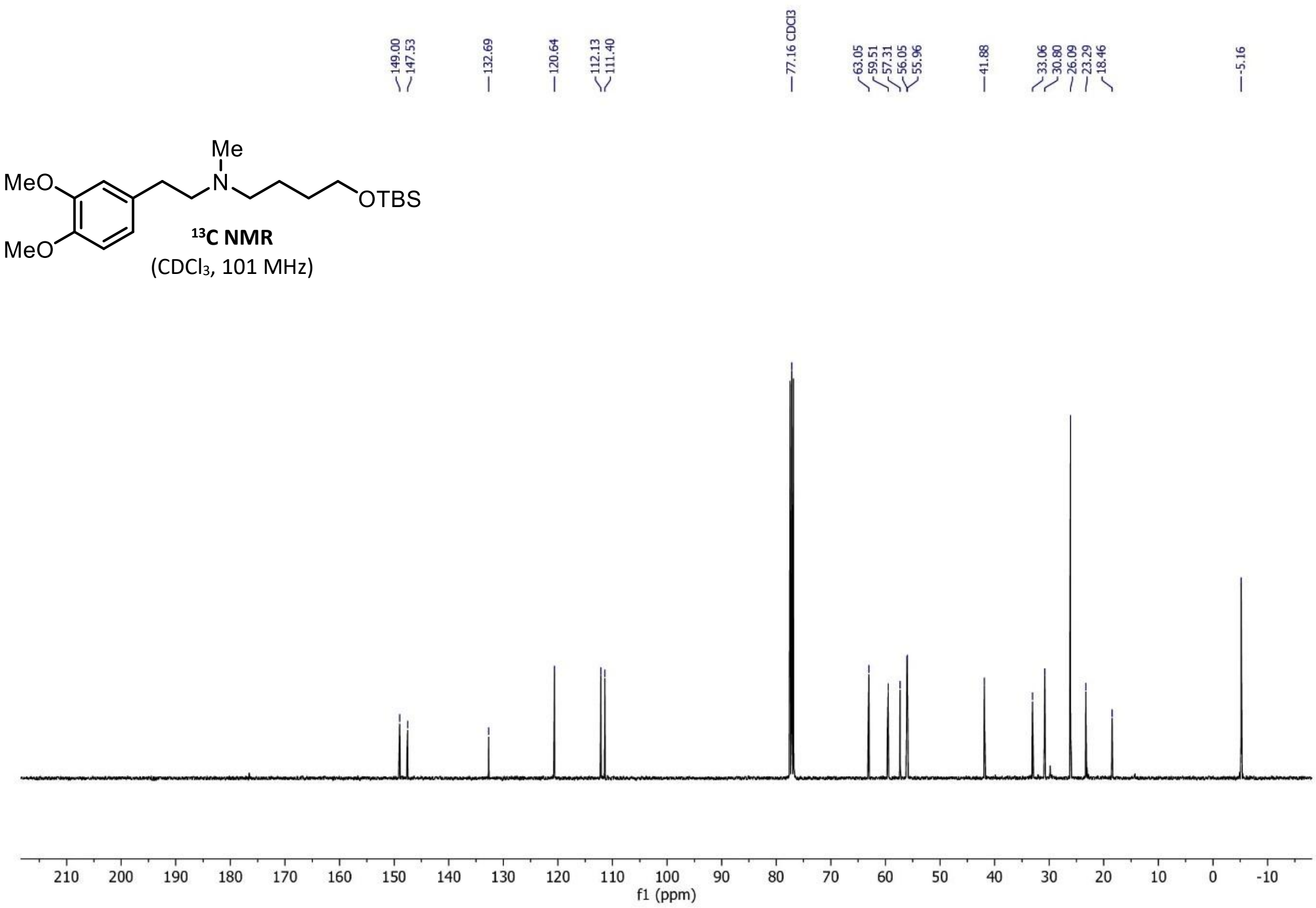

^{13}C NMR

($CDCl_3$, 101 MHz)

_N_-(3,4-Dimethoxyphenethyl)-_N_-methyl-4-(4-nitrophenoxy)butan-1-amine (2k)

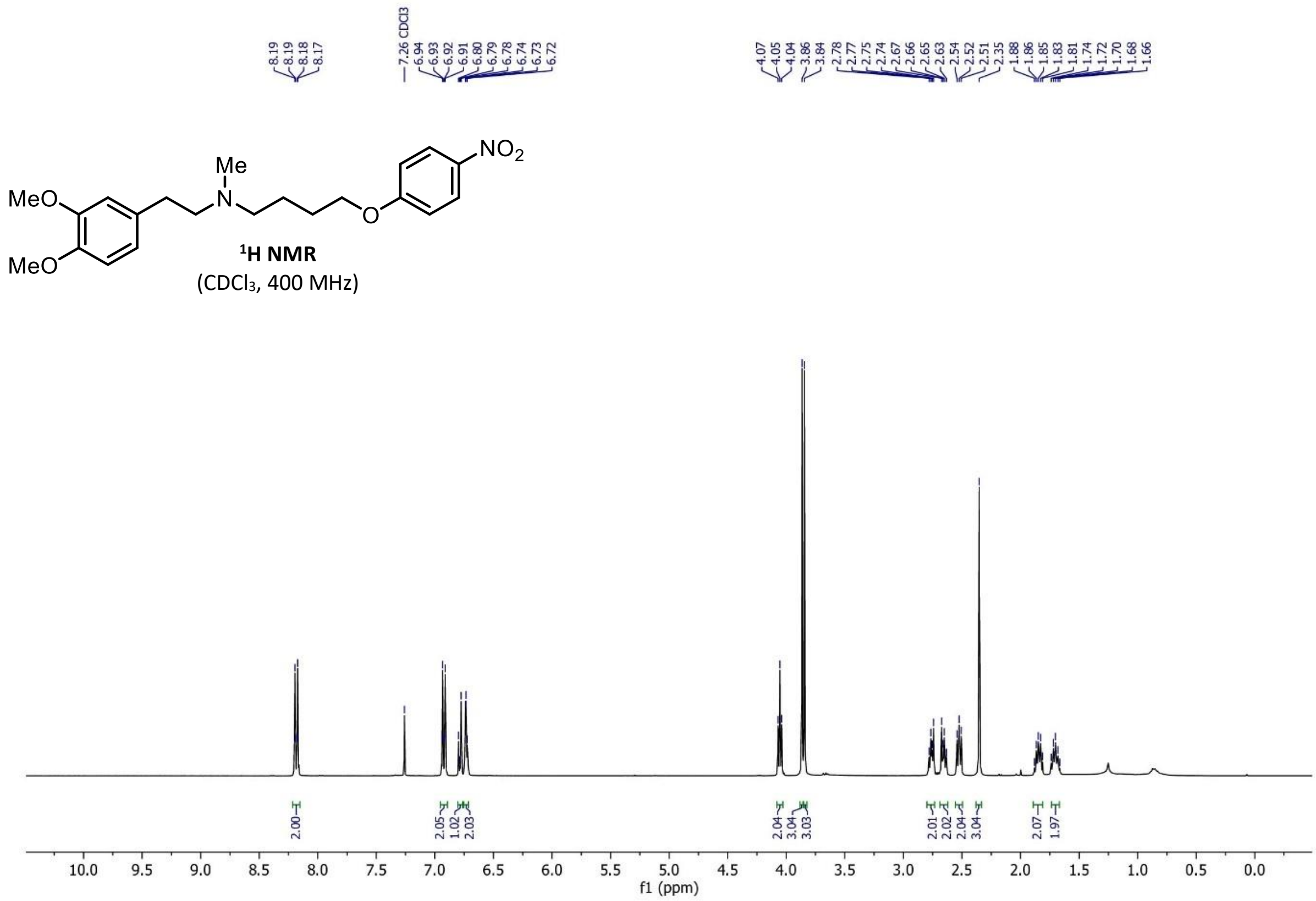

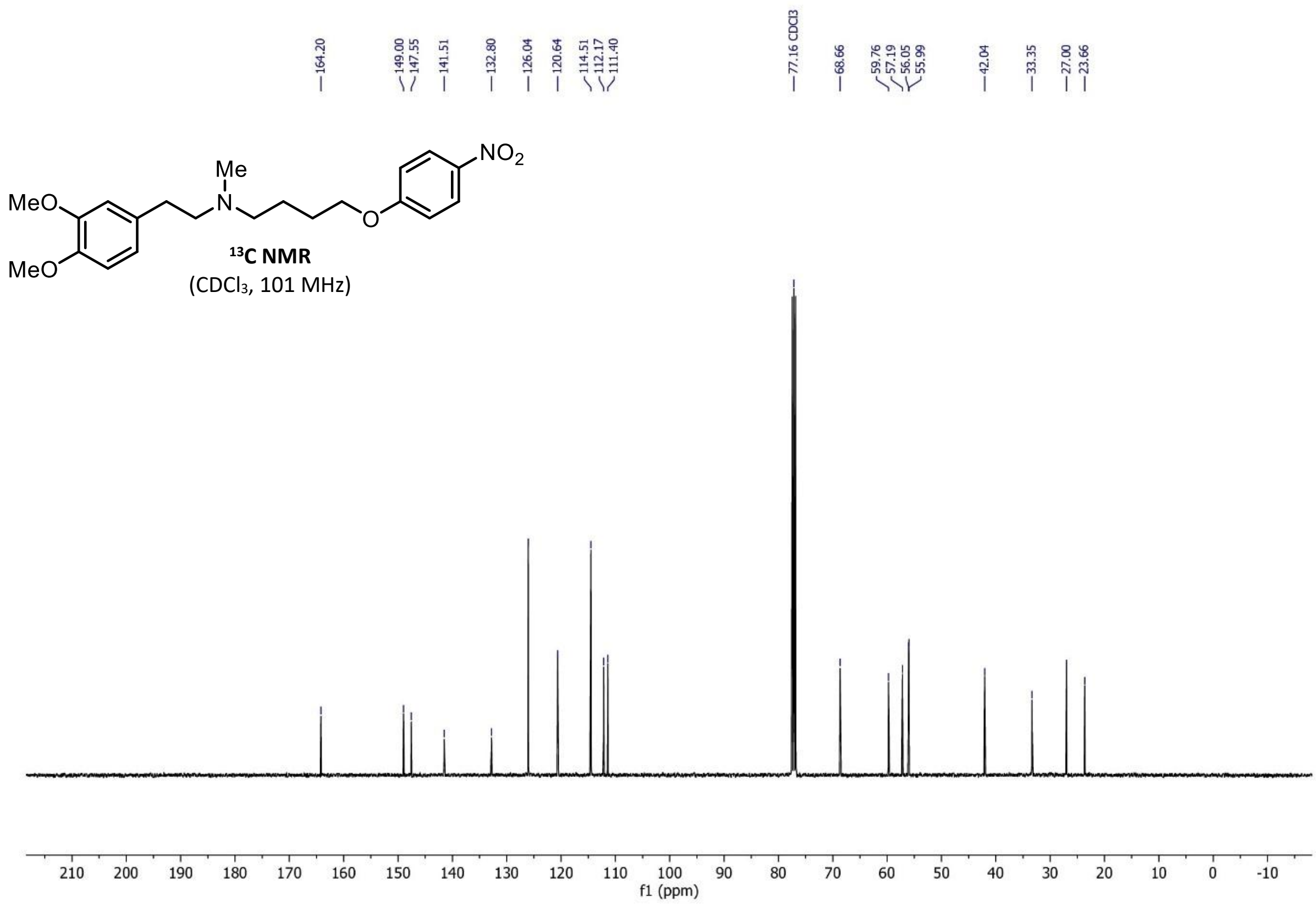

2-(3,4-Dimethoxyphenethyl)isoindoline (2l)

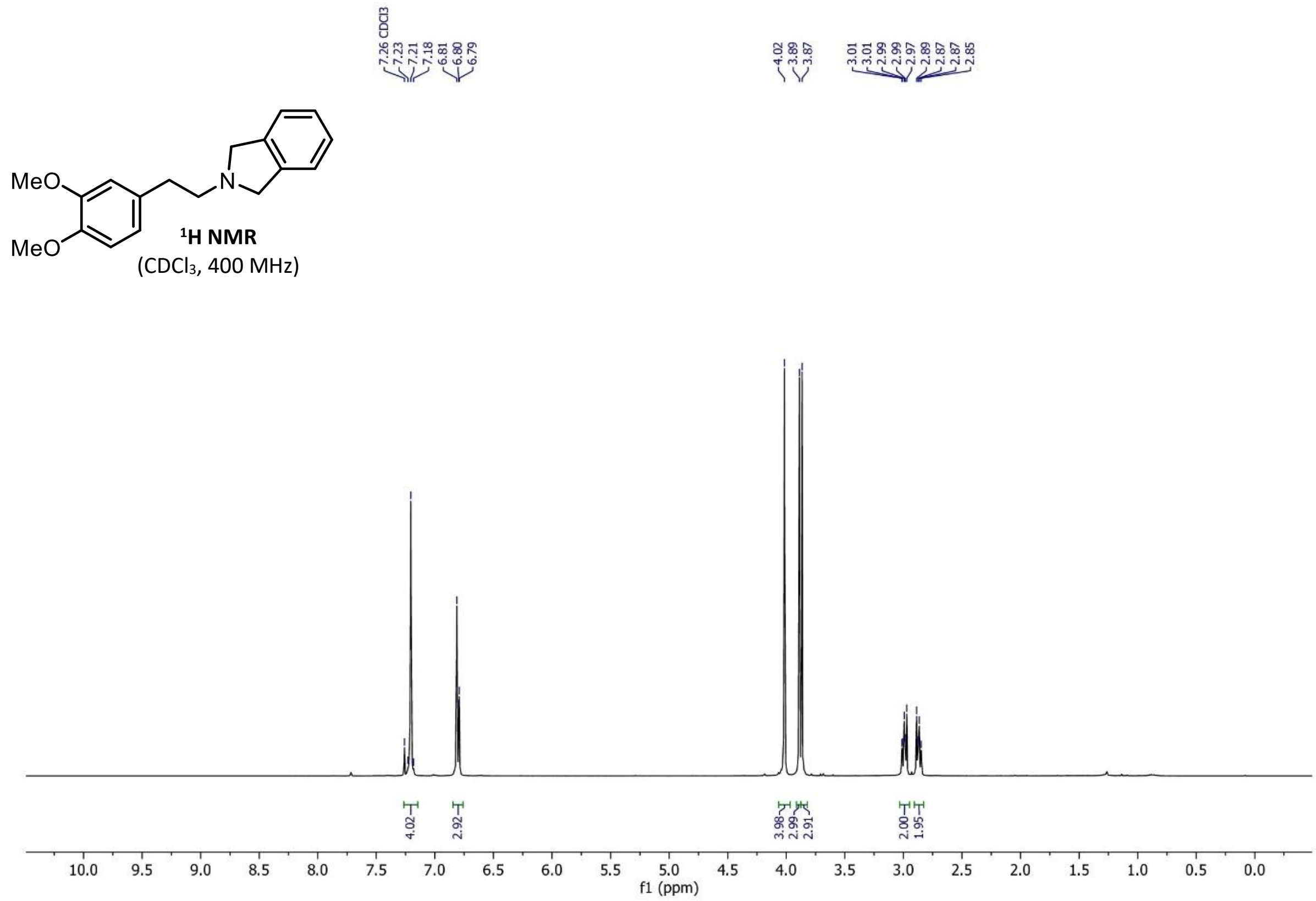

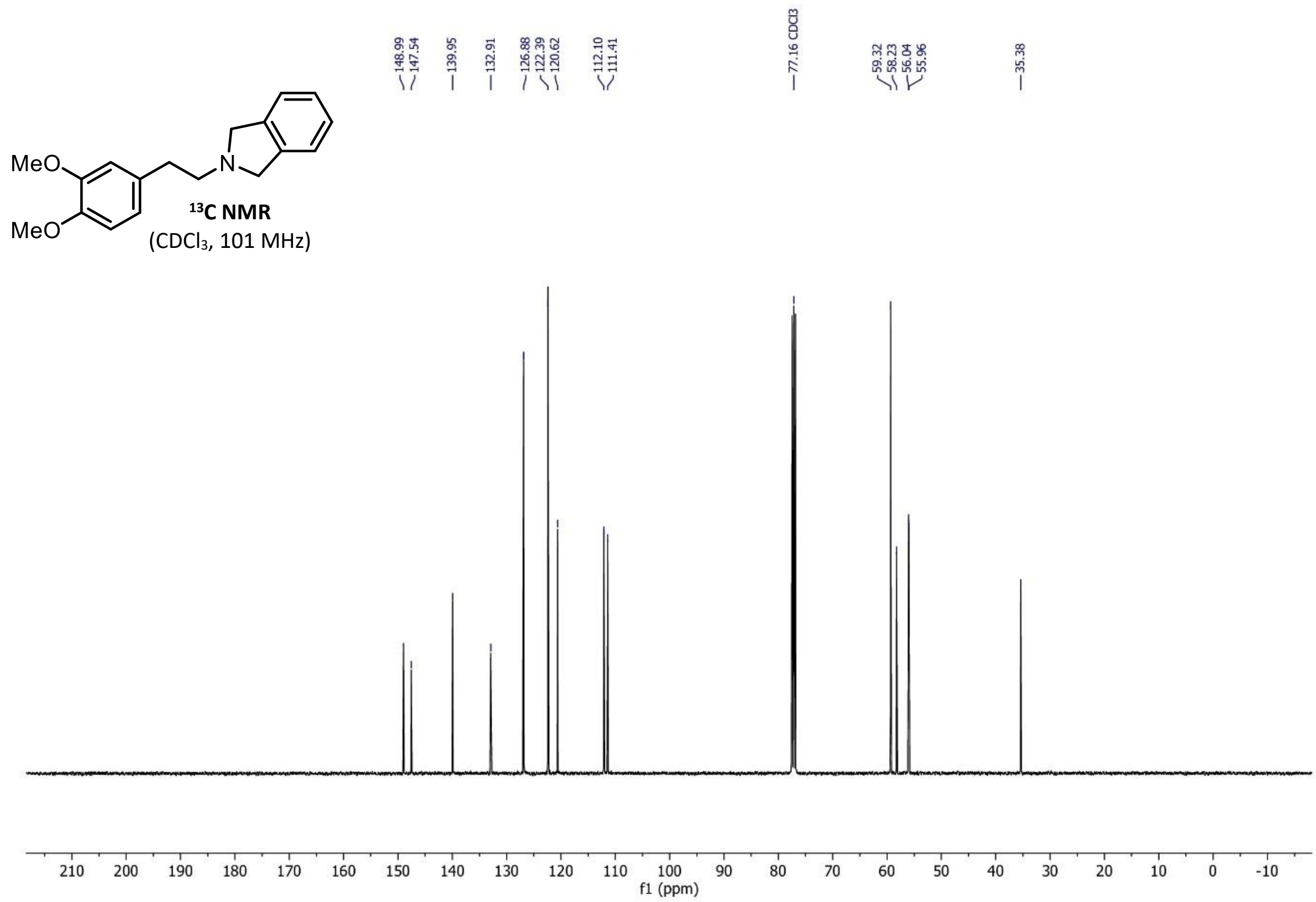

_N_-(3,4-Dimethoxyphenethyl)-3-(4,5-diphenyloxazol-2-yl)-_N_-methylpropan-1-amine (2m)

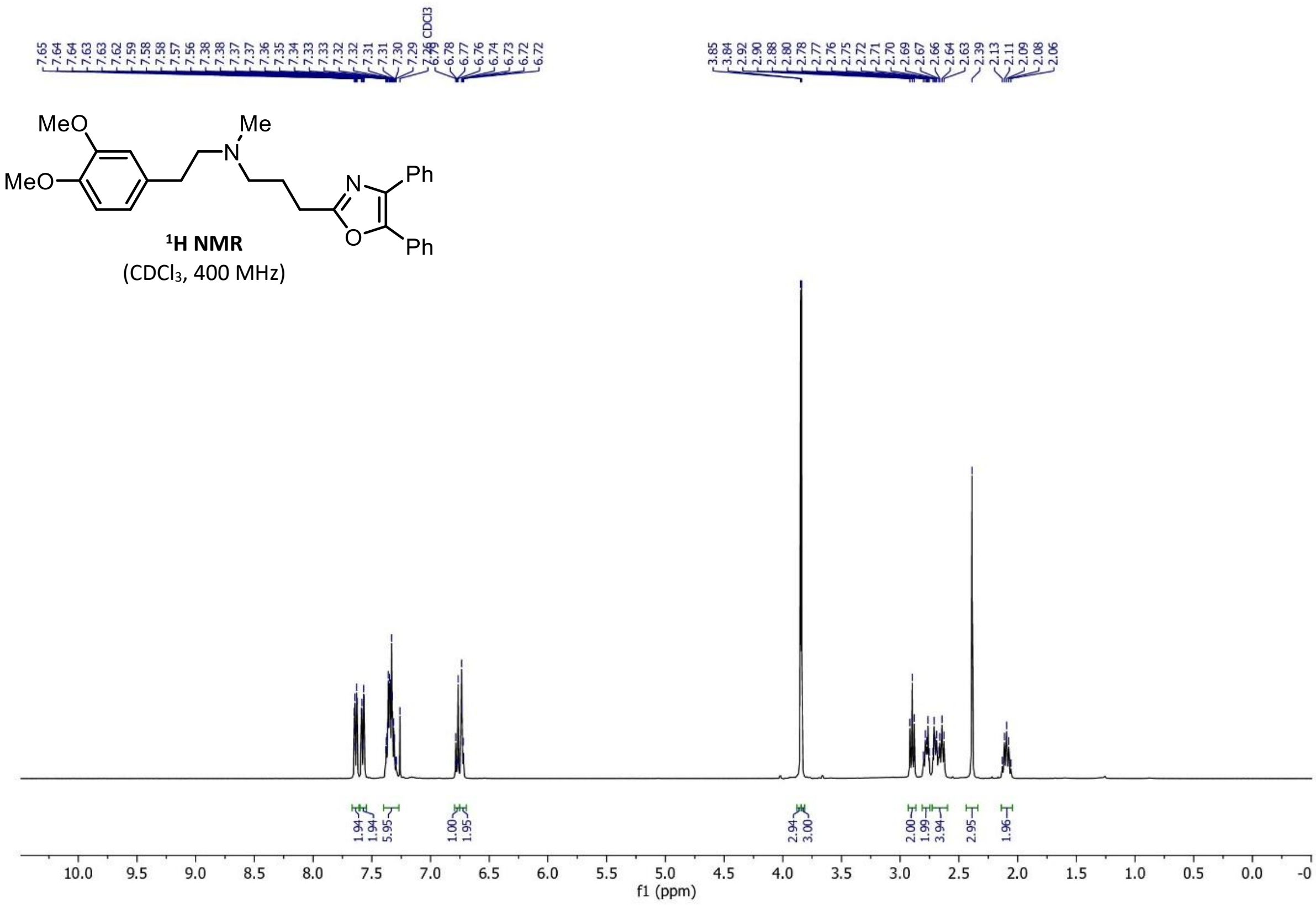

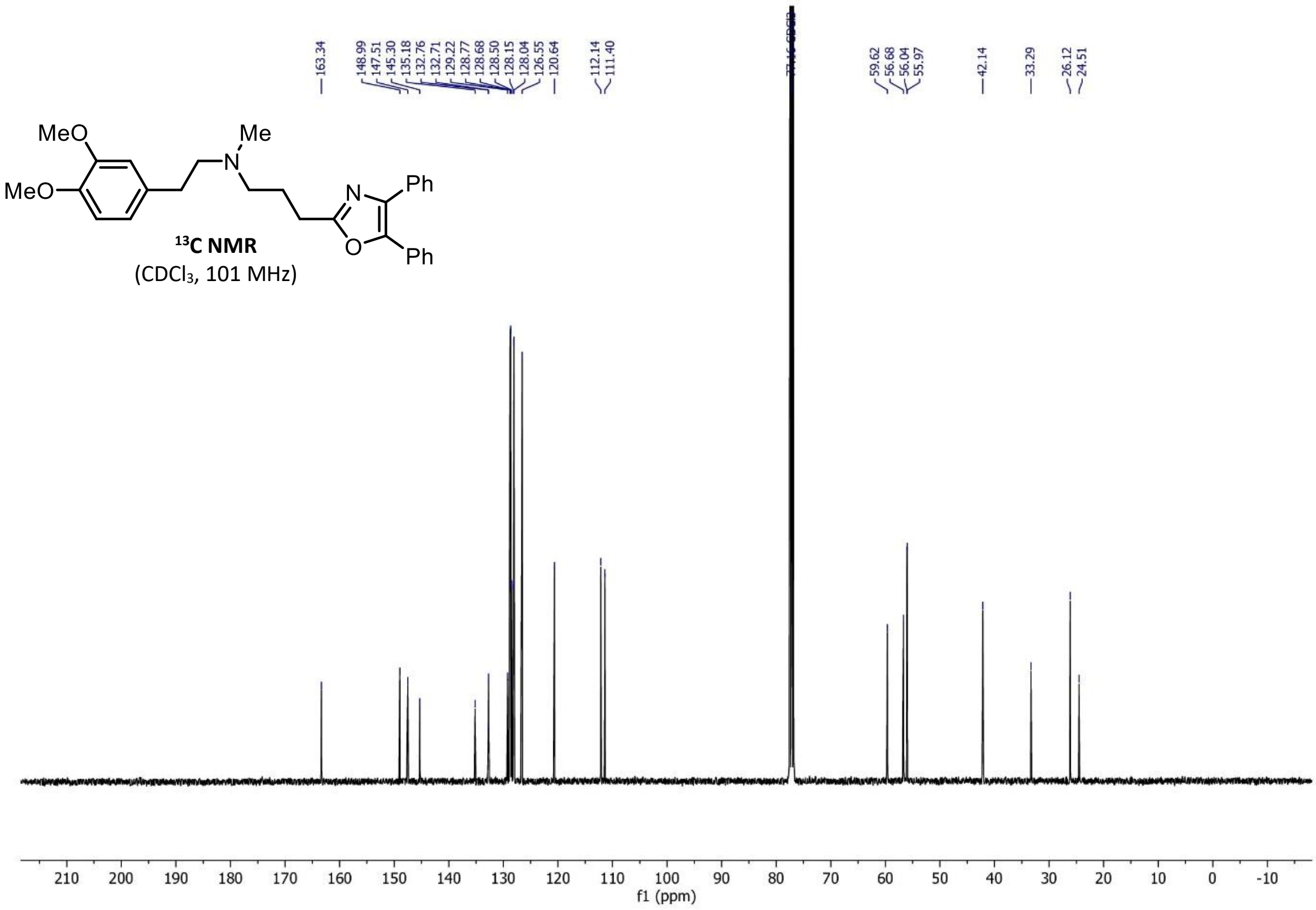

(3a*S*,4*S*,6a*R*)-4-(5-((3,4-Dimethoxyphenethyl)(methyl)amino)pentyl)tetrahydro-1*H*-thieno[3,4-*d*]imidazol-2(3*H*)-one (2n)

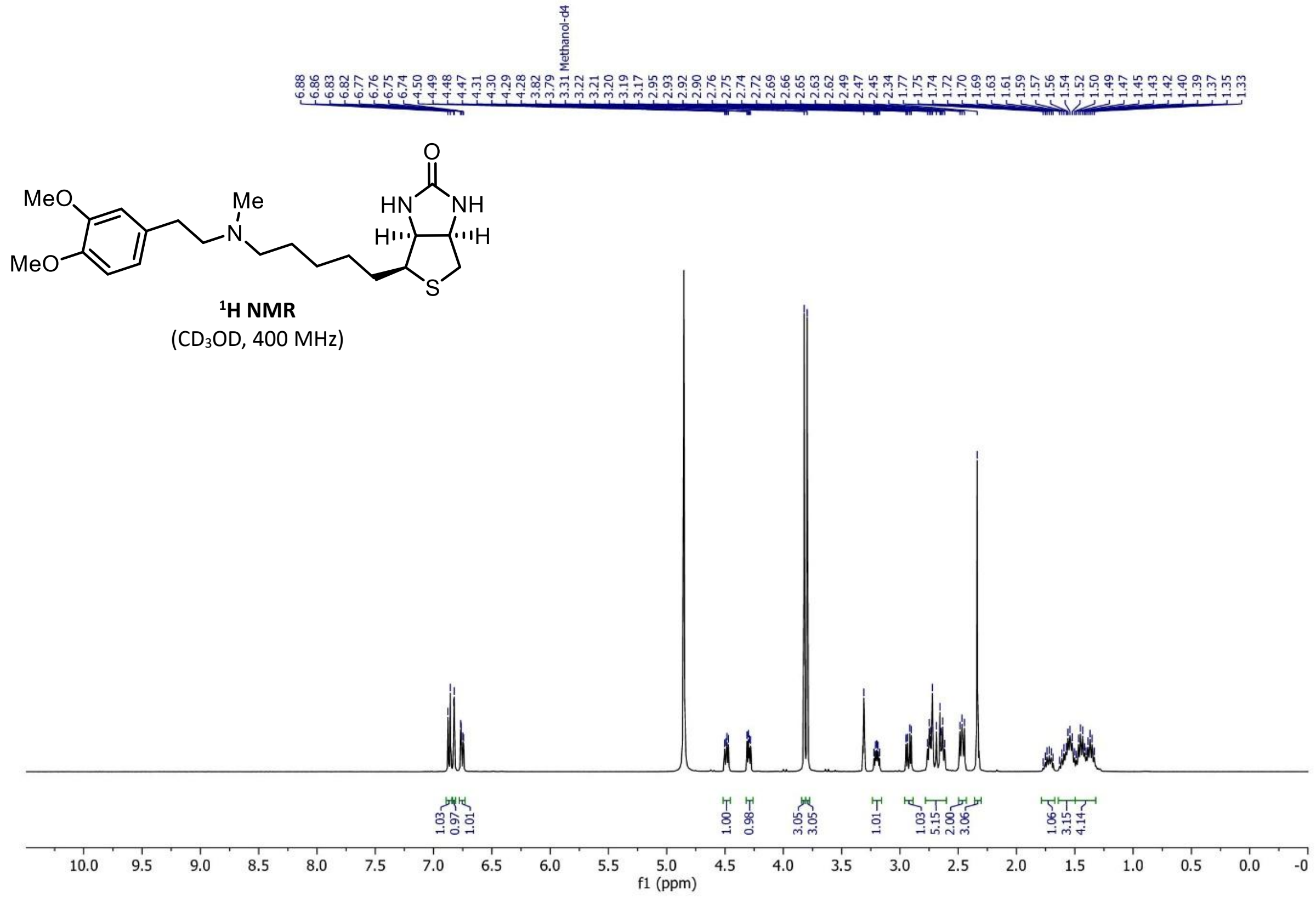

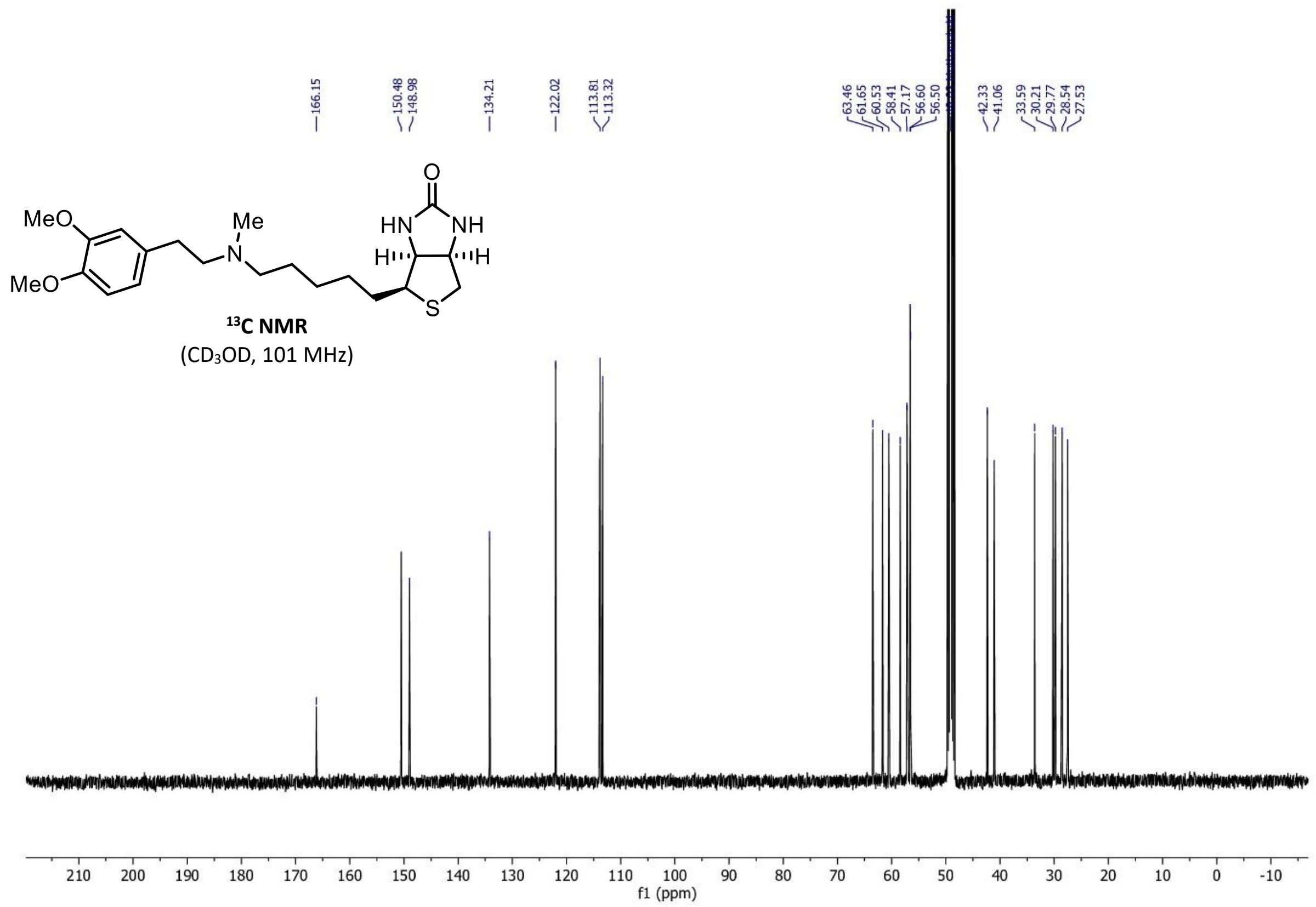

(*E*)-*N*-(3,4-Dimethoxyphenethyl)-2-(5-fluoro-2-methyl-1-(4-(methylsulfinyl)benzylidene)-1*H*-inden-3-yl)-*N*-methylethan-1-amine (2o)

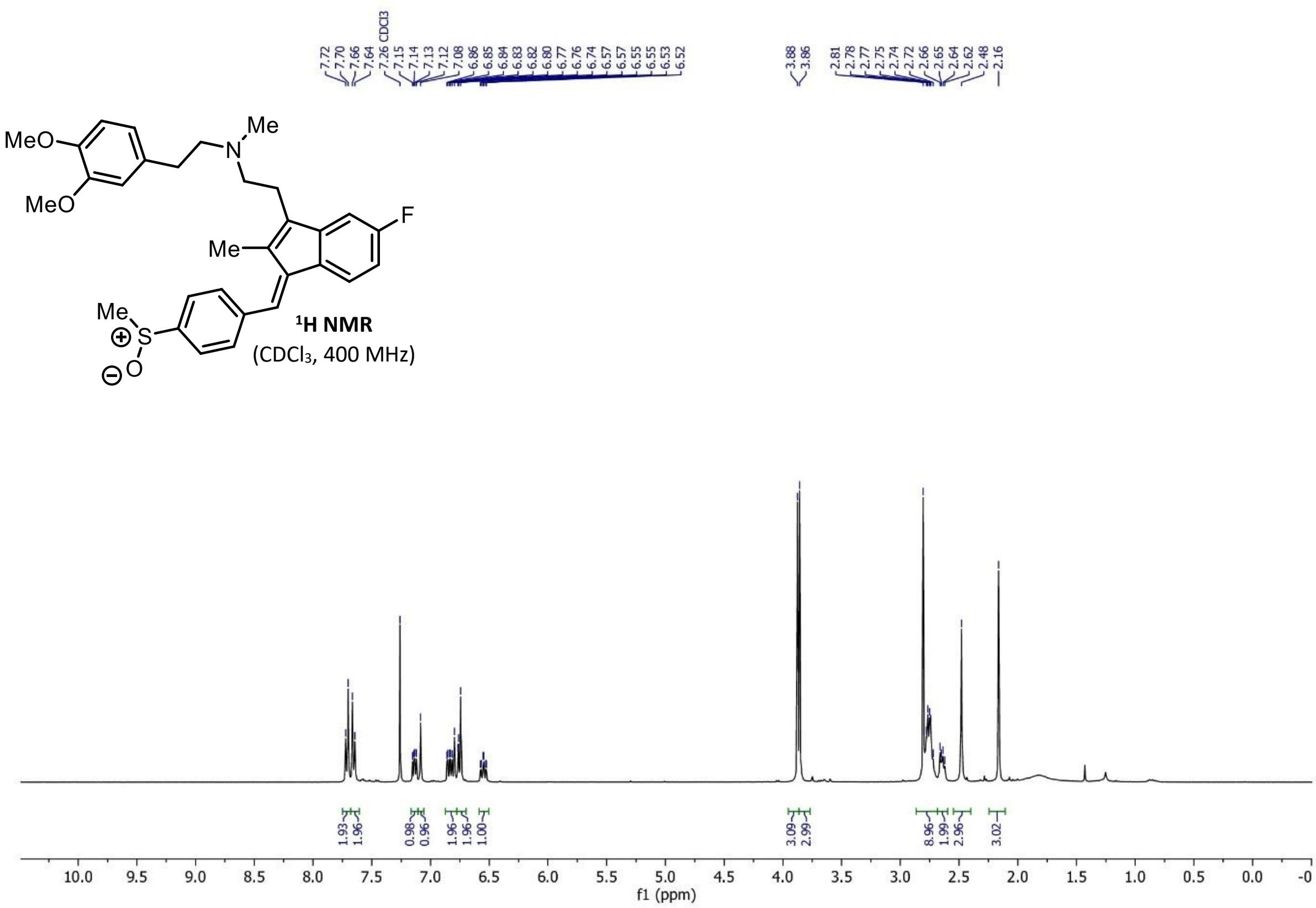

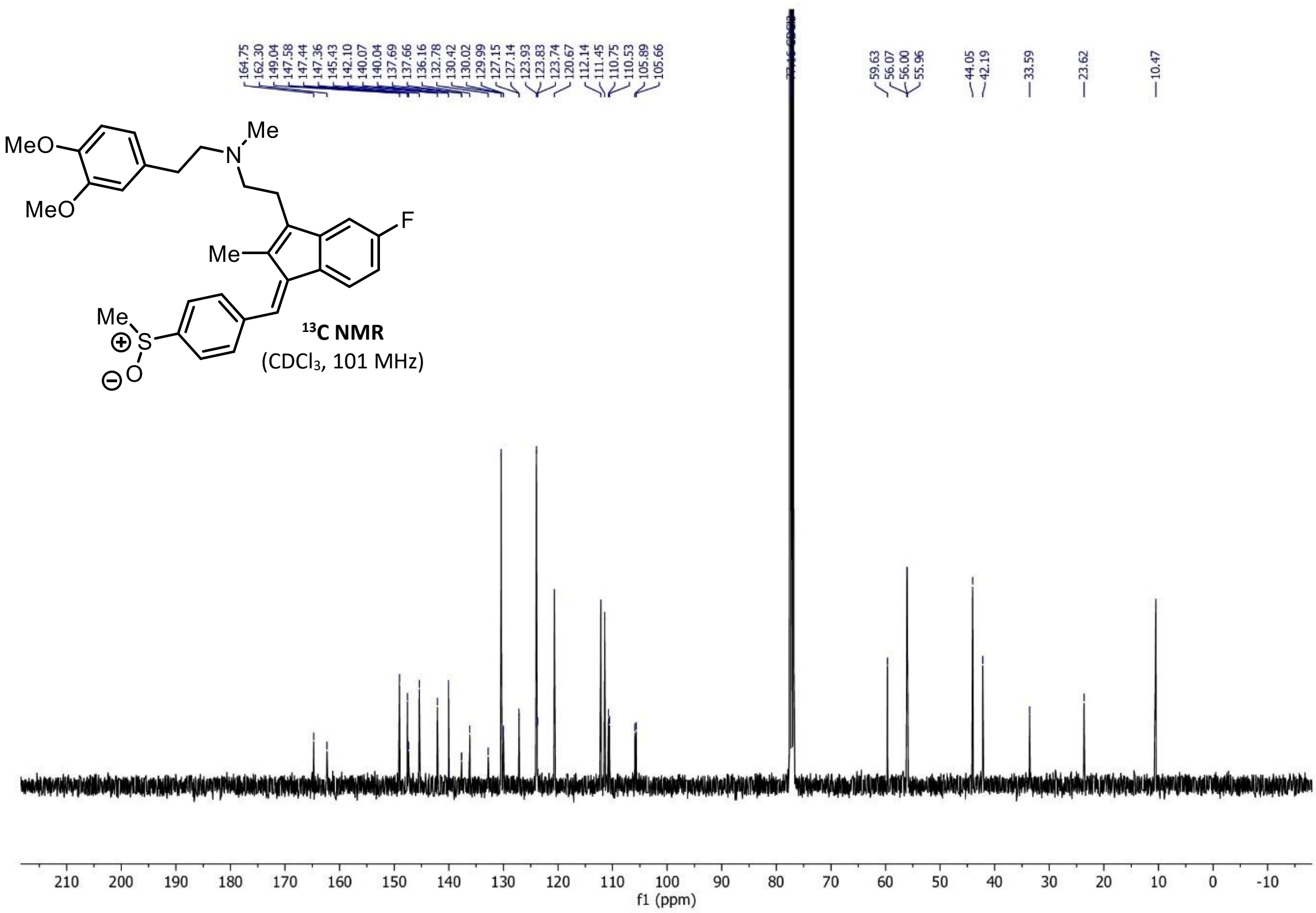

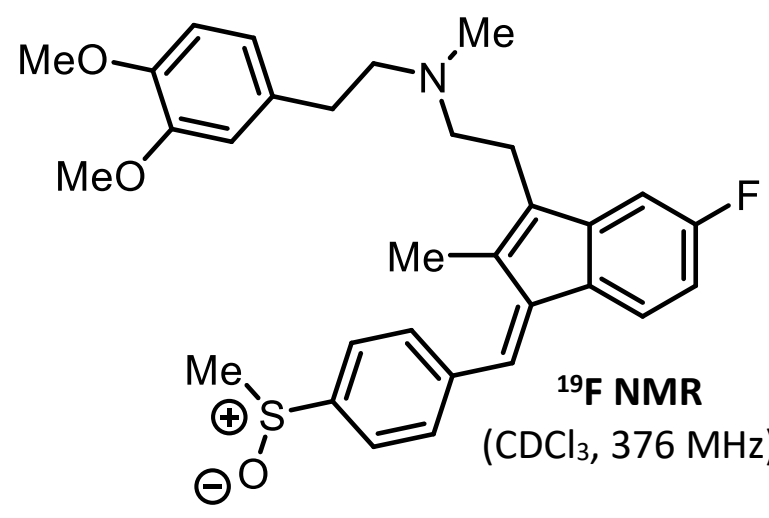

^{19}F NMR
($CDCl_3$, 376 MHz)

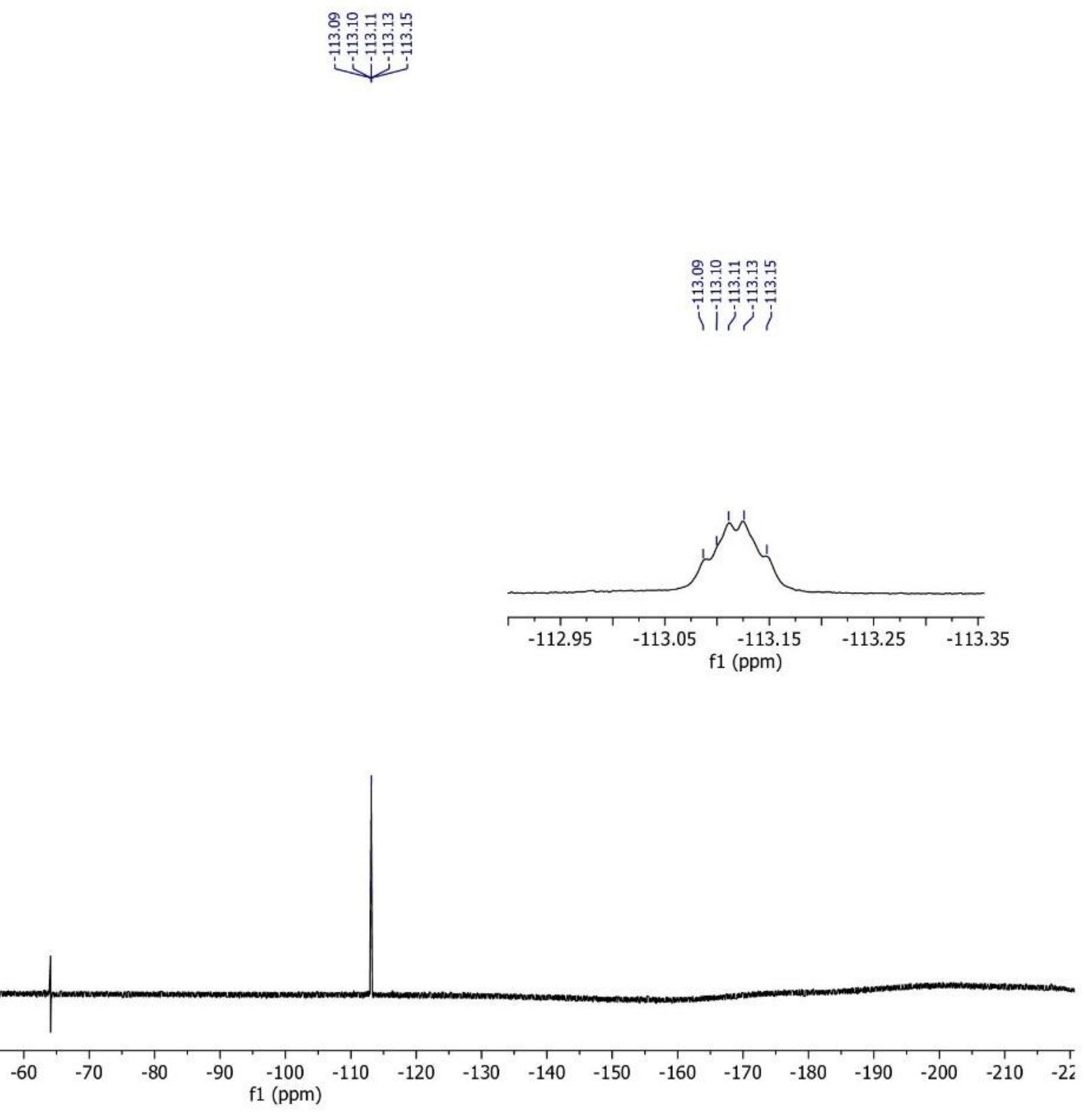

5-(5-(((3,4-Dimethoxyphenethyl)(methyl)amino)methyl)-4-methylthiazol-2-yl)-2-isobutoxybenzonitrile (2p)

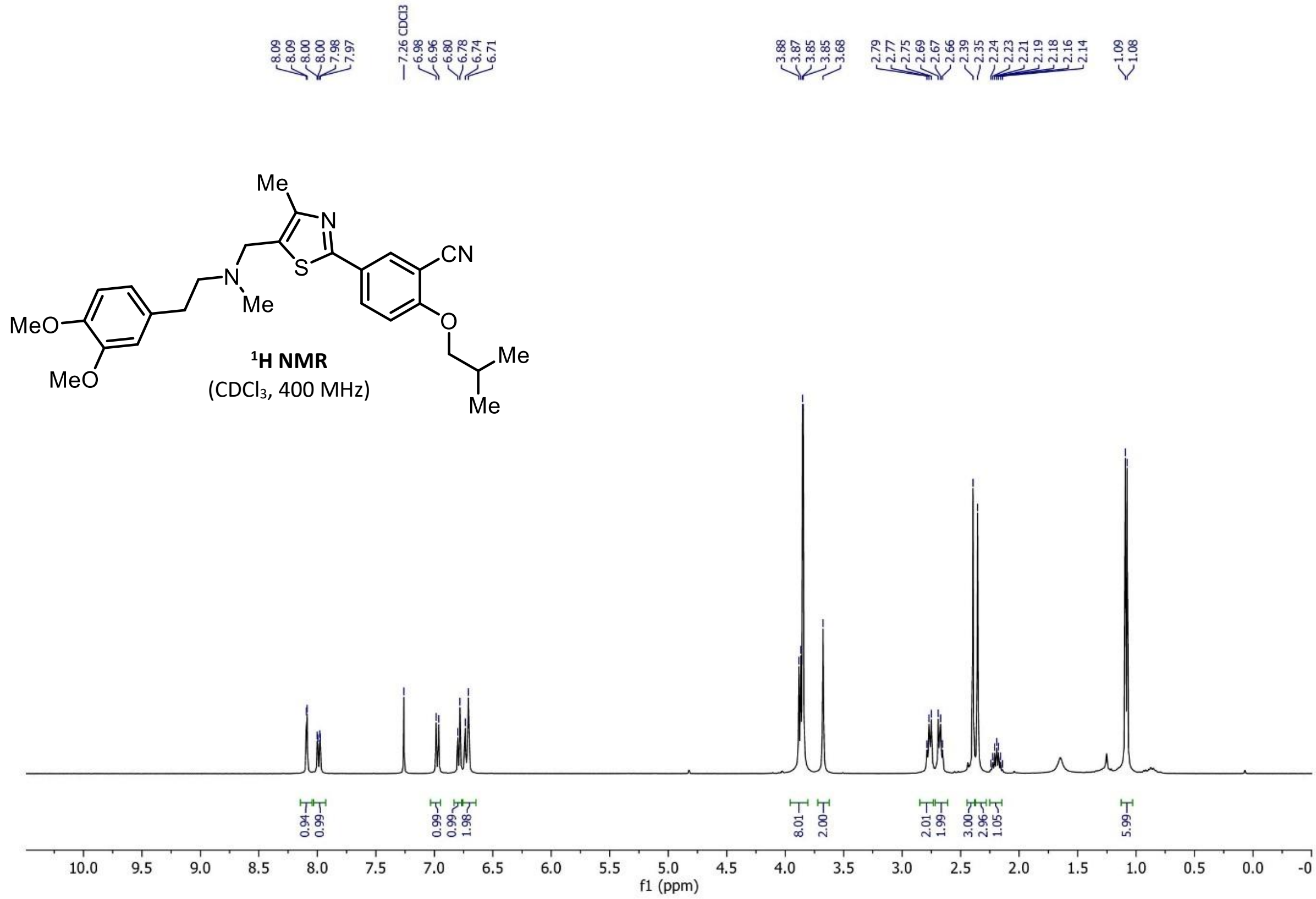

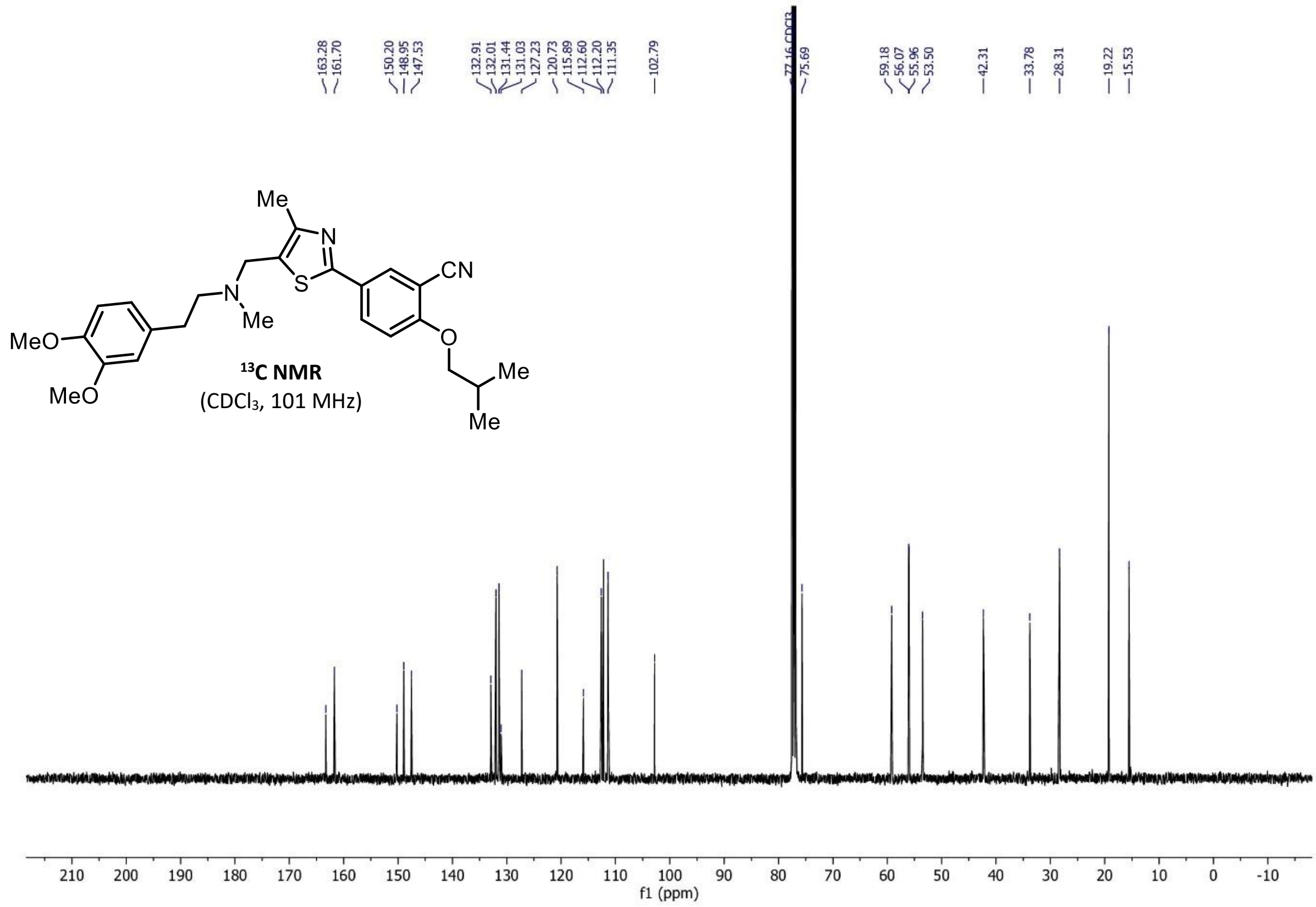

2-(3,4-Dimethoxyphenyl)-*N*-(4-(5-(2-fluorophenyl)-1,2,4-oxadiazol-3-yl)benzyl)-*N*-methylethan-1-amine (2q)

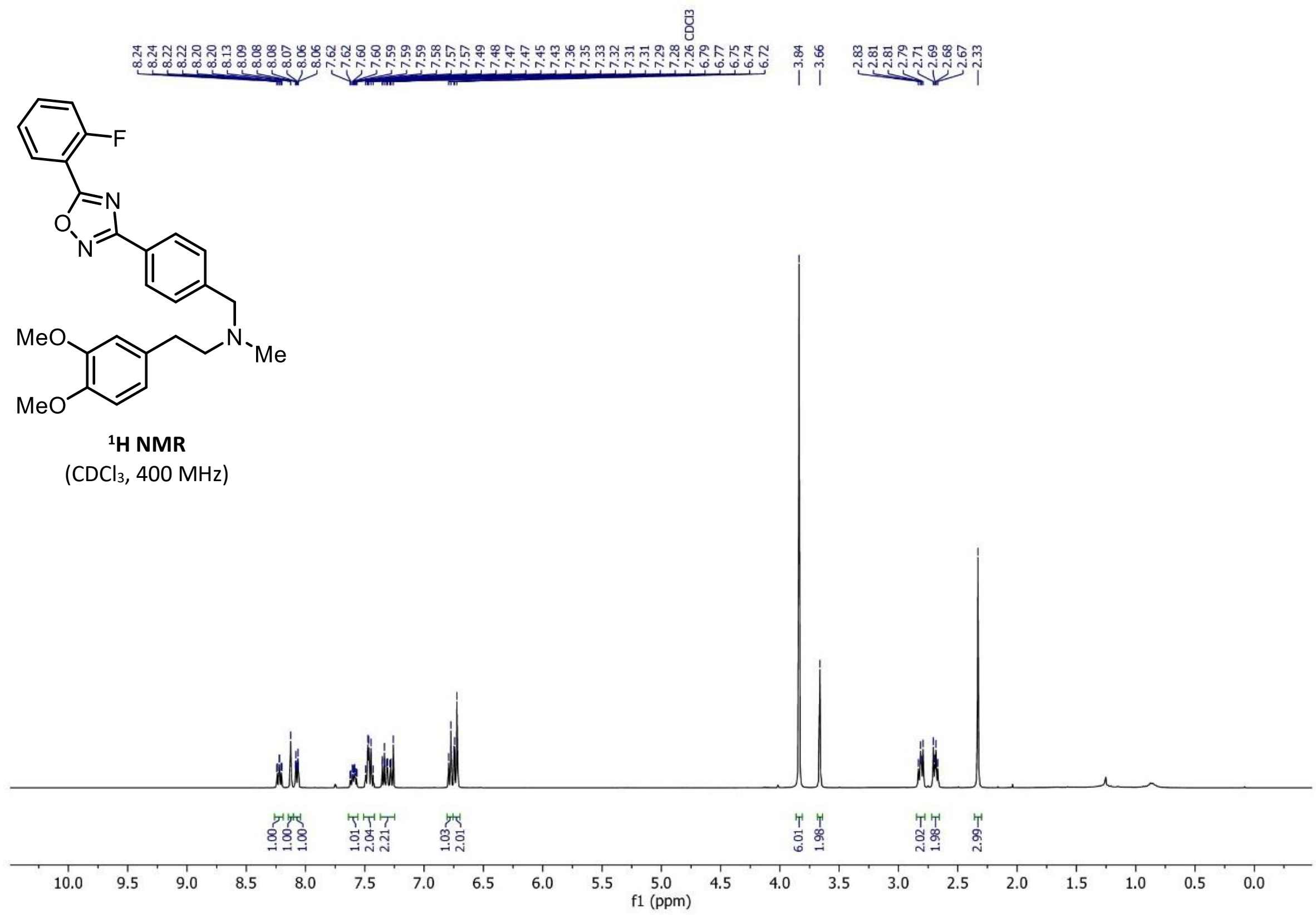

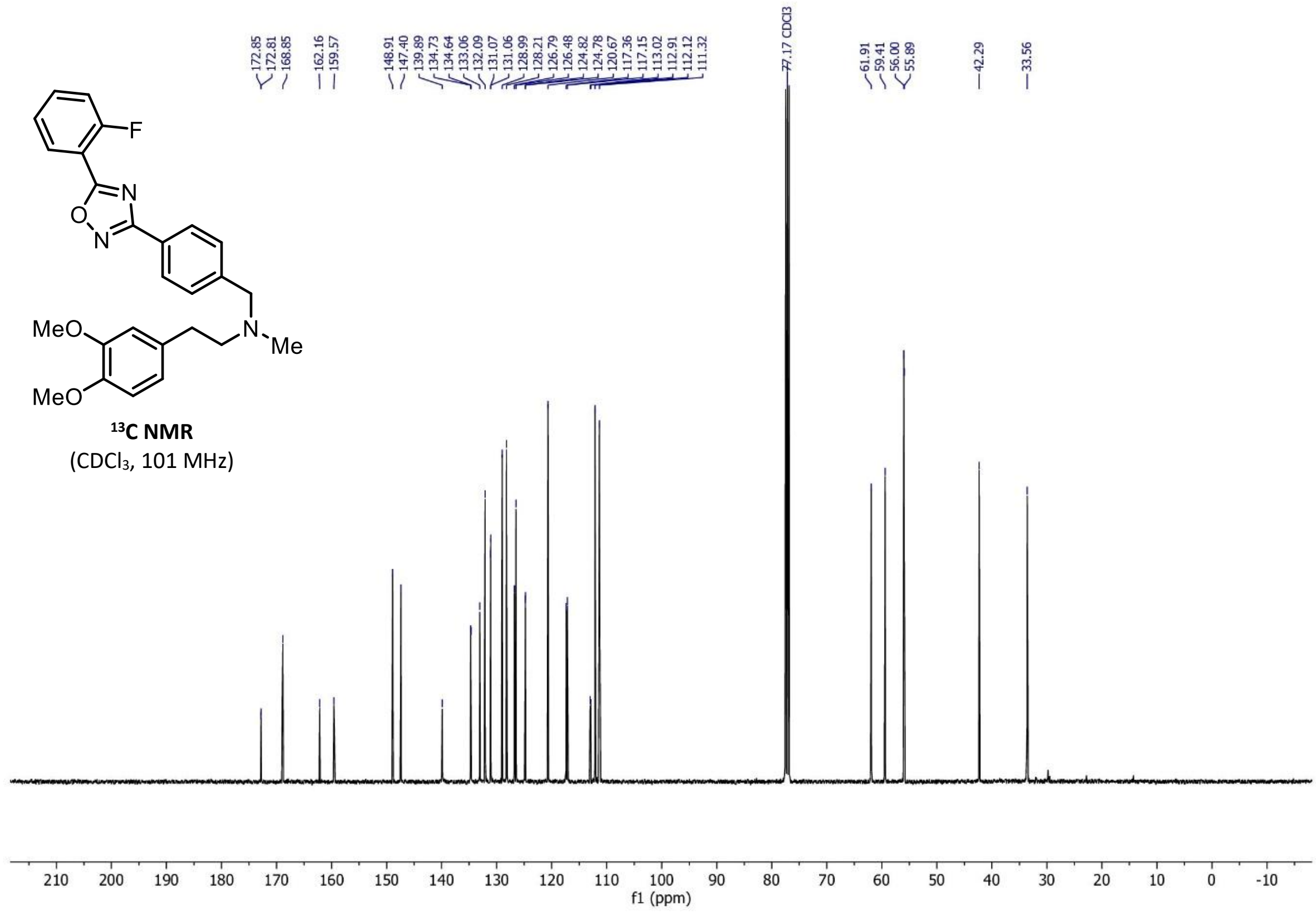

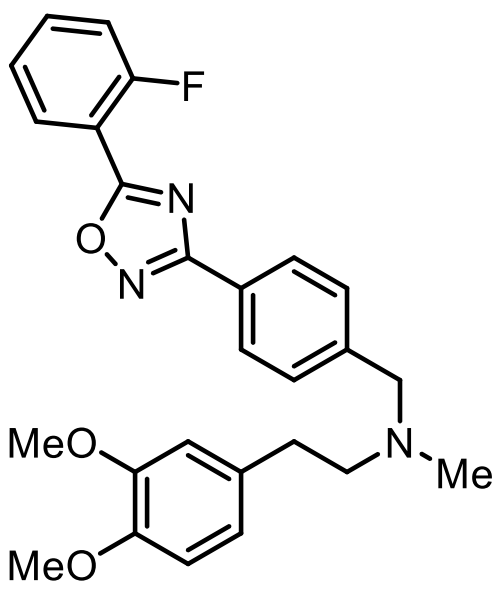

^{19}F NMR
($CDCl_3$, 376 MHz)

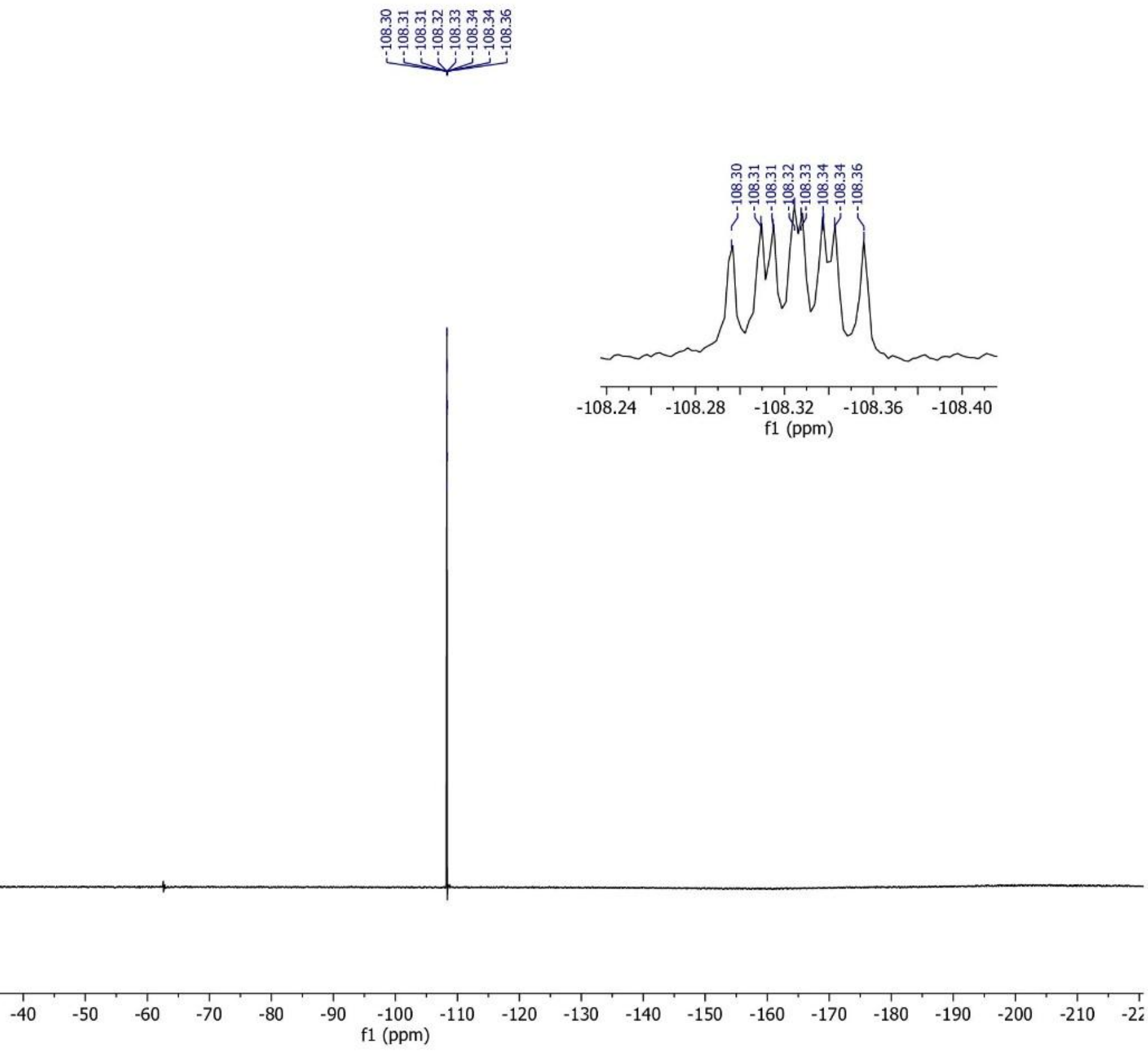

(3*R*,5*R*,8*R*,9*S*,10*S*,13*R*,14*S*,17*R*)-17-((*R*)-5-((3,4-Dimethoxyphenethyl)(methyl)amino)pentan-2-yl)-10,13-dimethylhexadecahydro-1*H*-cyclopenta[*a*]phenanthren-3-ol (2r)

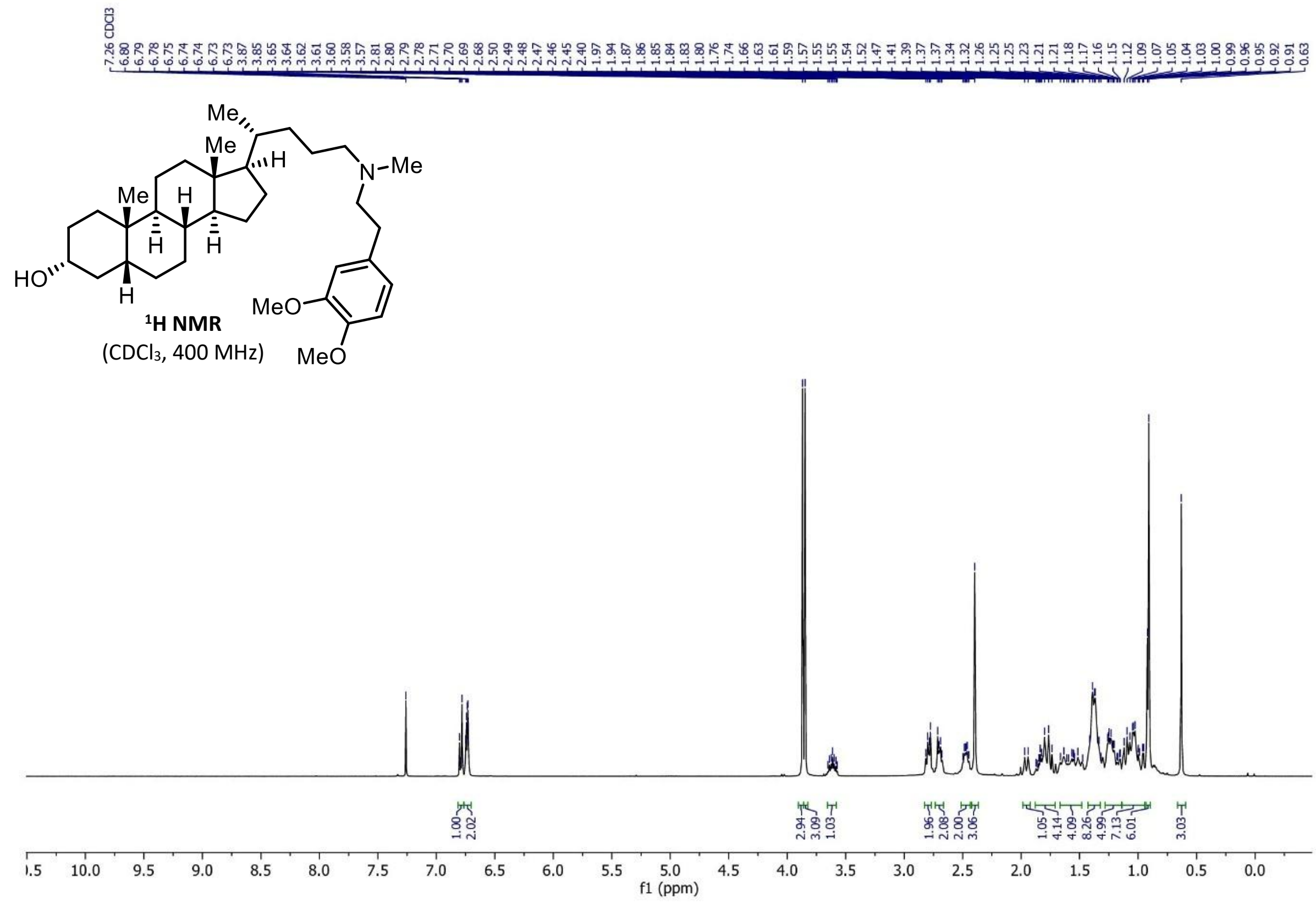

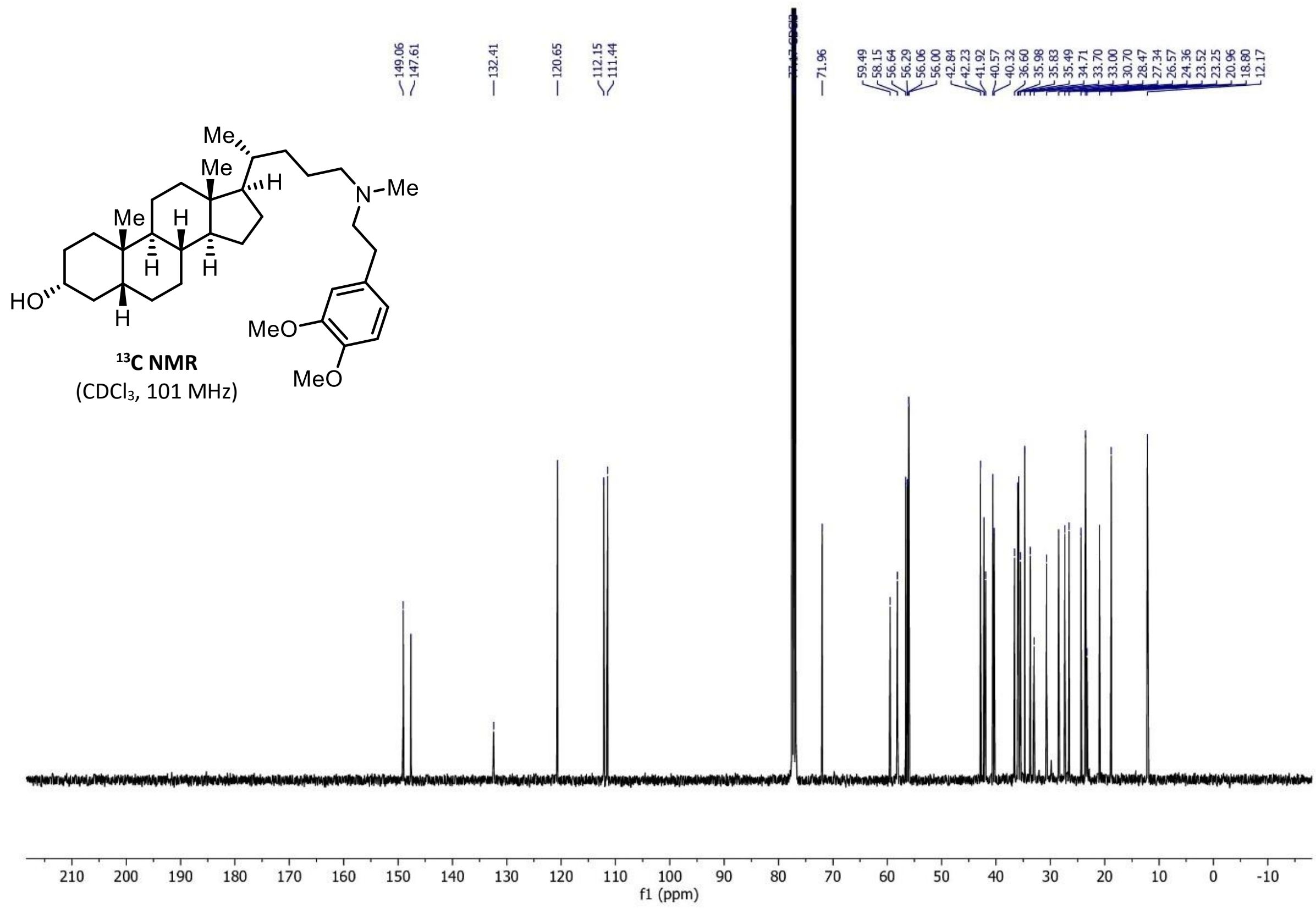

(*S*)-*N*-(3,4-Dimethoxyphenethyl)-*N*-methyl-1-phenylethan-1-amine (2s)

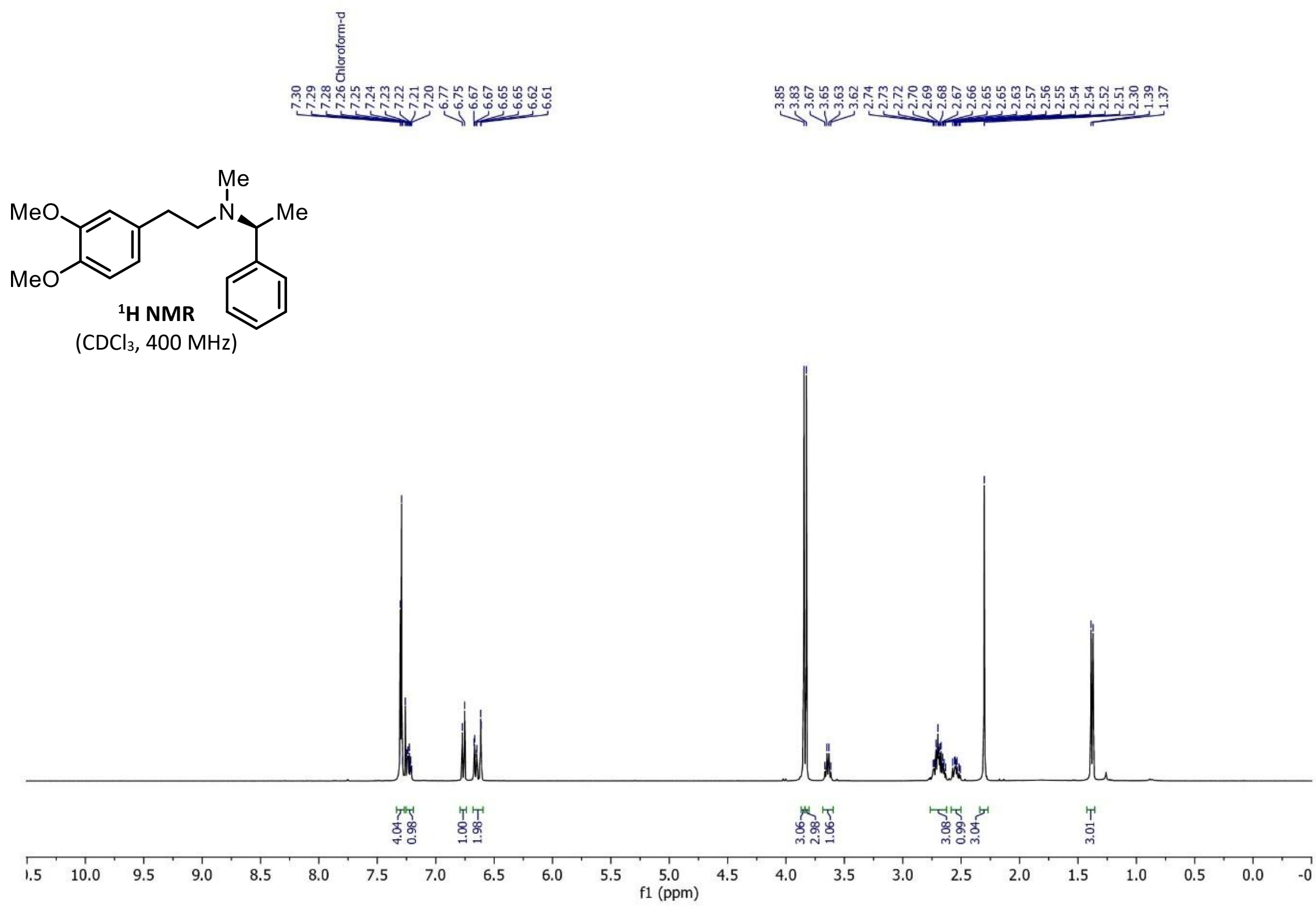

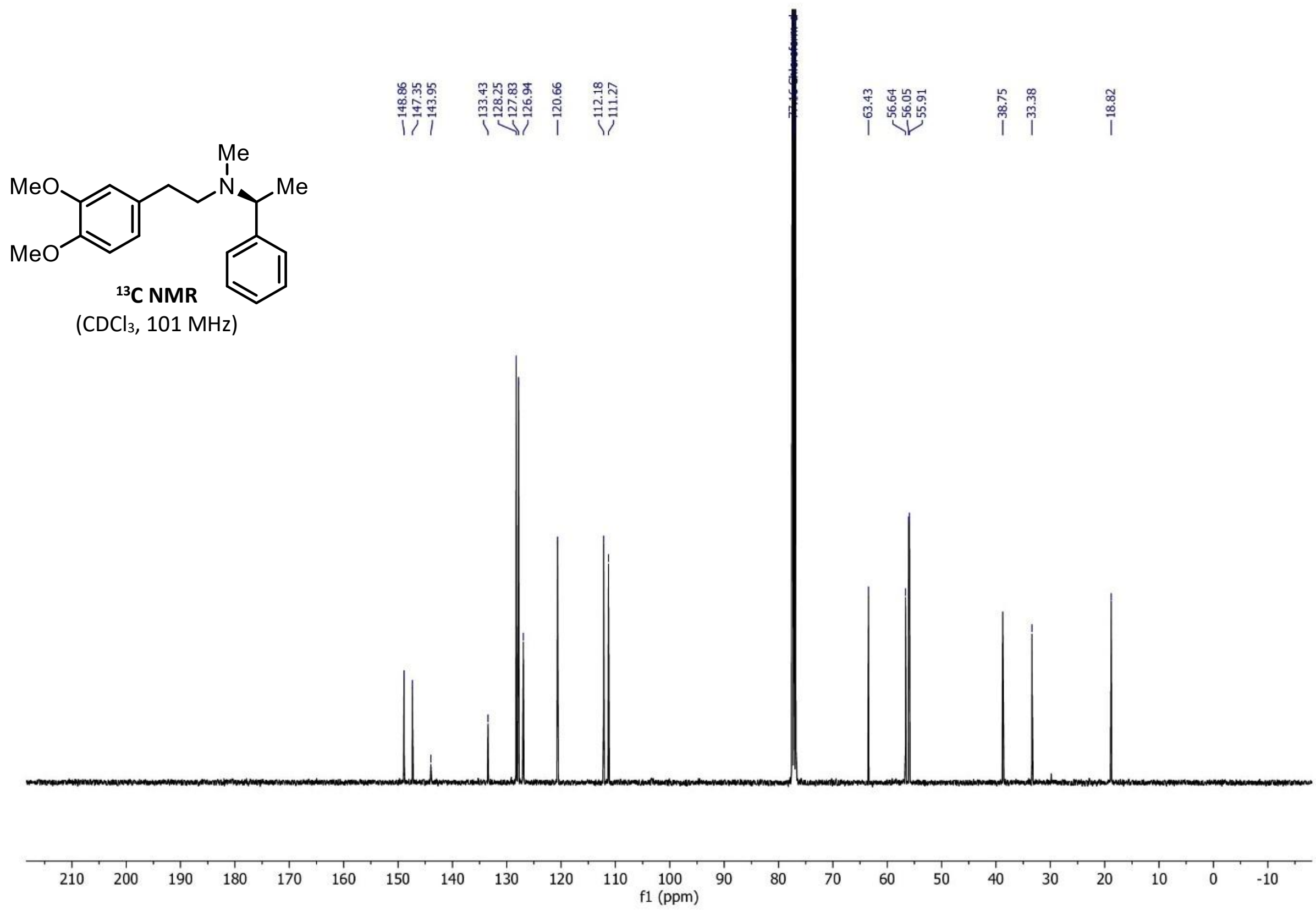

(3*R*,4*S*)-3-((*R*)-3-((3,4-Dimethoxyphenethyl)(methyl)amino)-3-(4-fluorophenyl)propyl)-1-(4-fluorophenyl)-4-(4-methoxyphenyl)azetidin-2-one (2t)

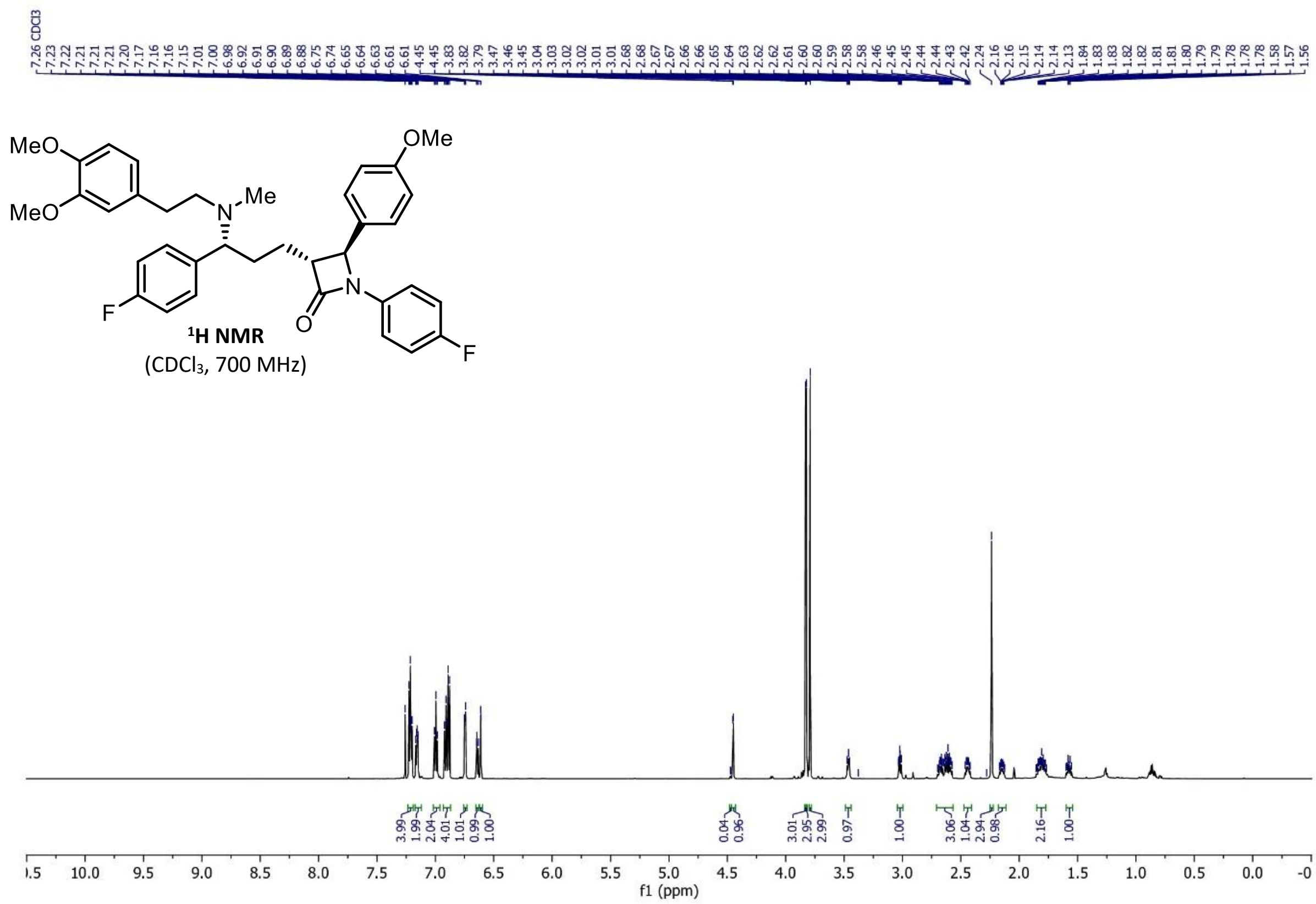

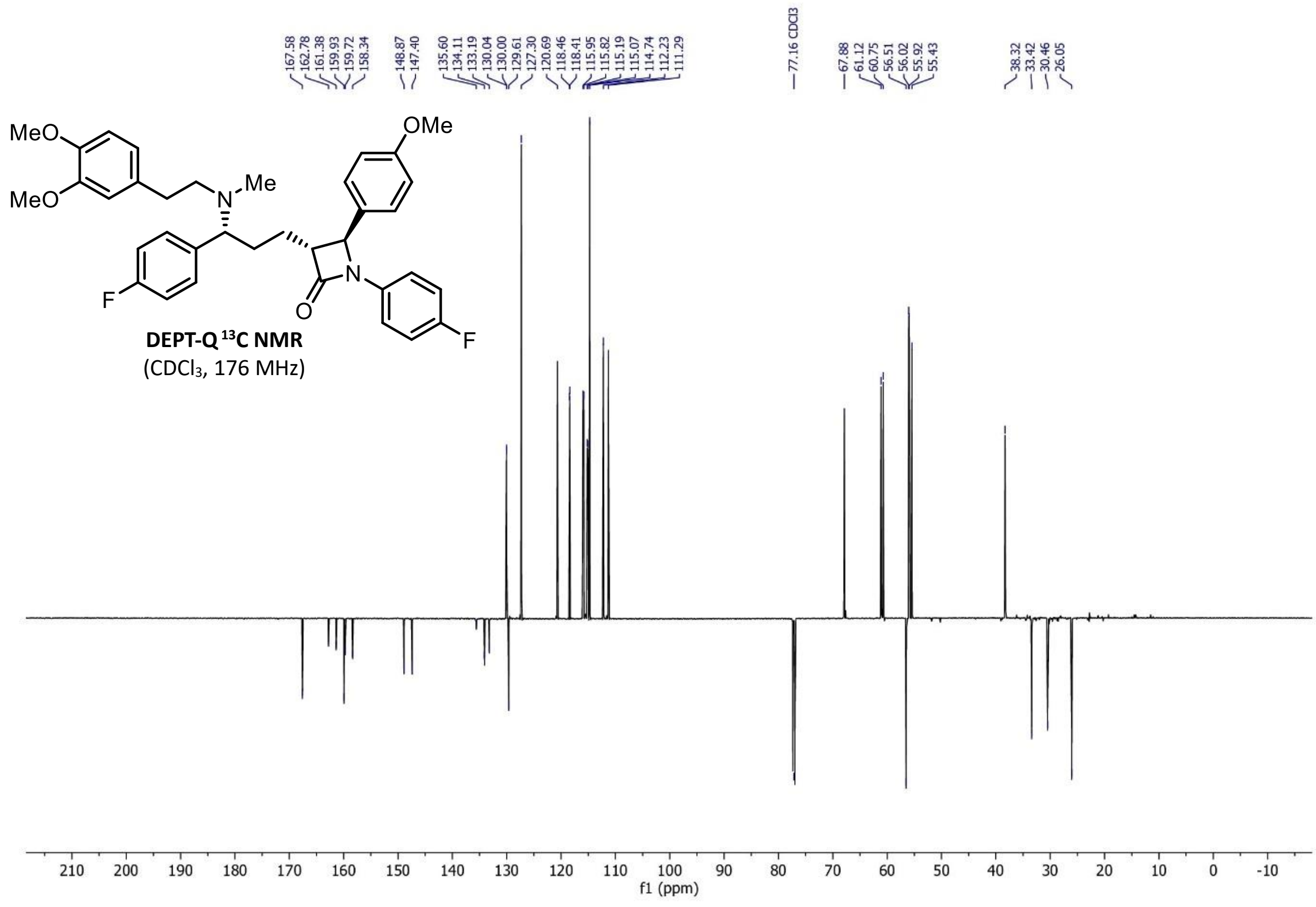

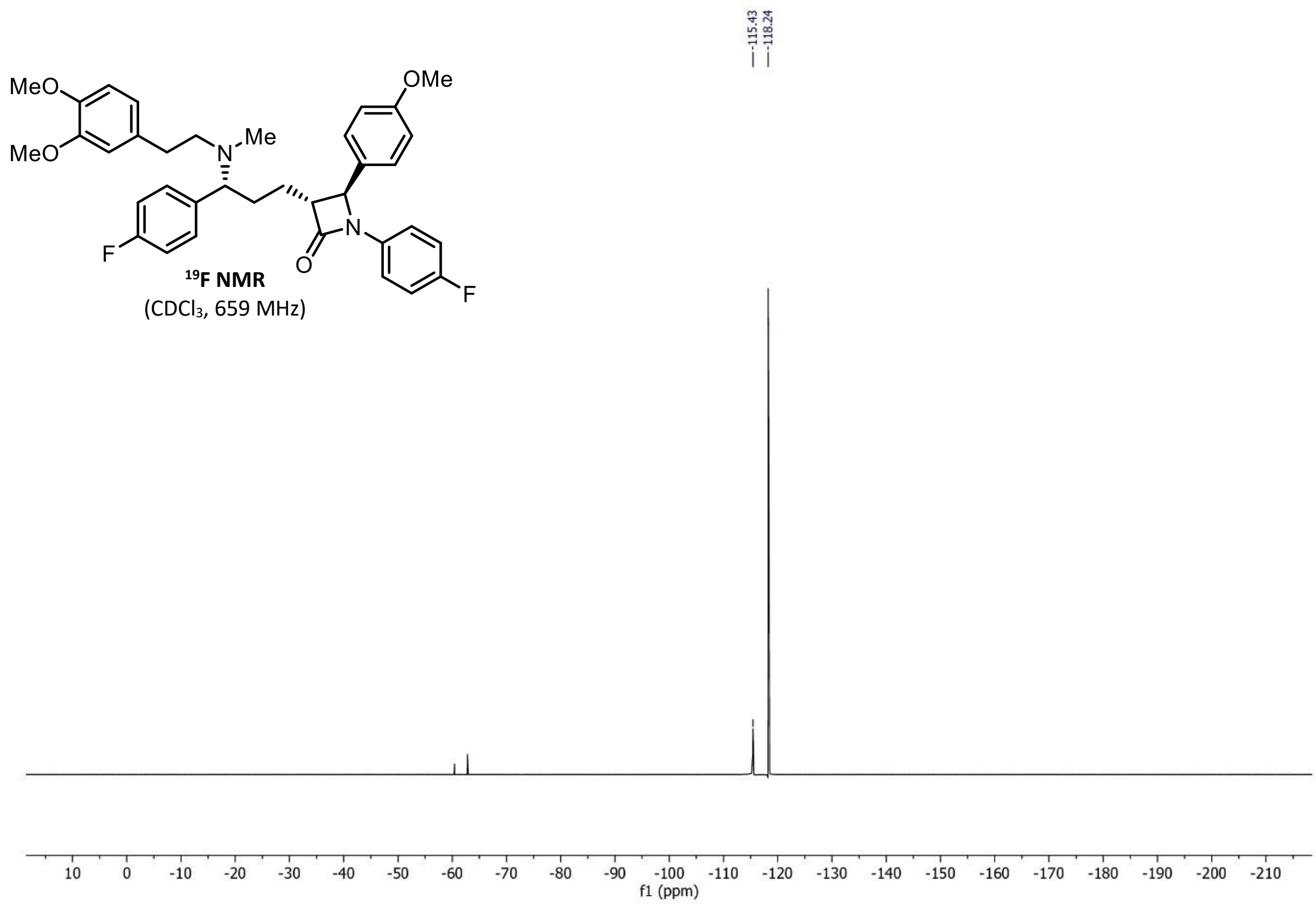
MeO
MeO
Me
OMe
N
O
F
F
¹⁹F NMR
(CDCl3, 659 MHz)
-115.43
-118.24
10 0 -10 -20 -30 -40 -50 -60 -70 -80 -90 -100 -110 -120 -130 -140 -150 -160 -170 -180 -190 -200 -210
f1 (ppm)

(*S*)-4-(1-((3,4-Dimethoxyphenethyl)(methyl)amino)ethyl)-*N*,*N*-dipropylbenzenesulfonamide (2u)

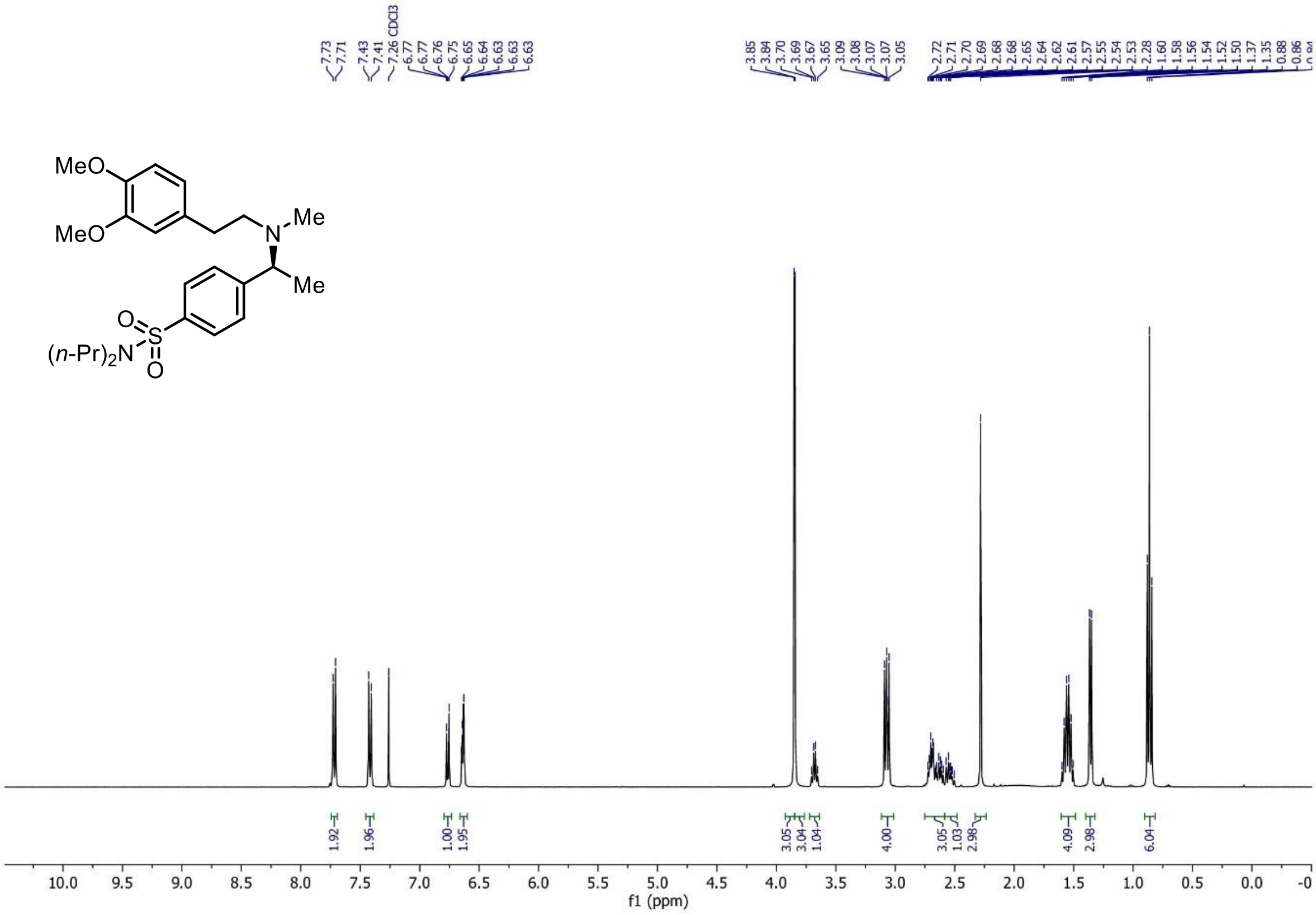

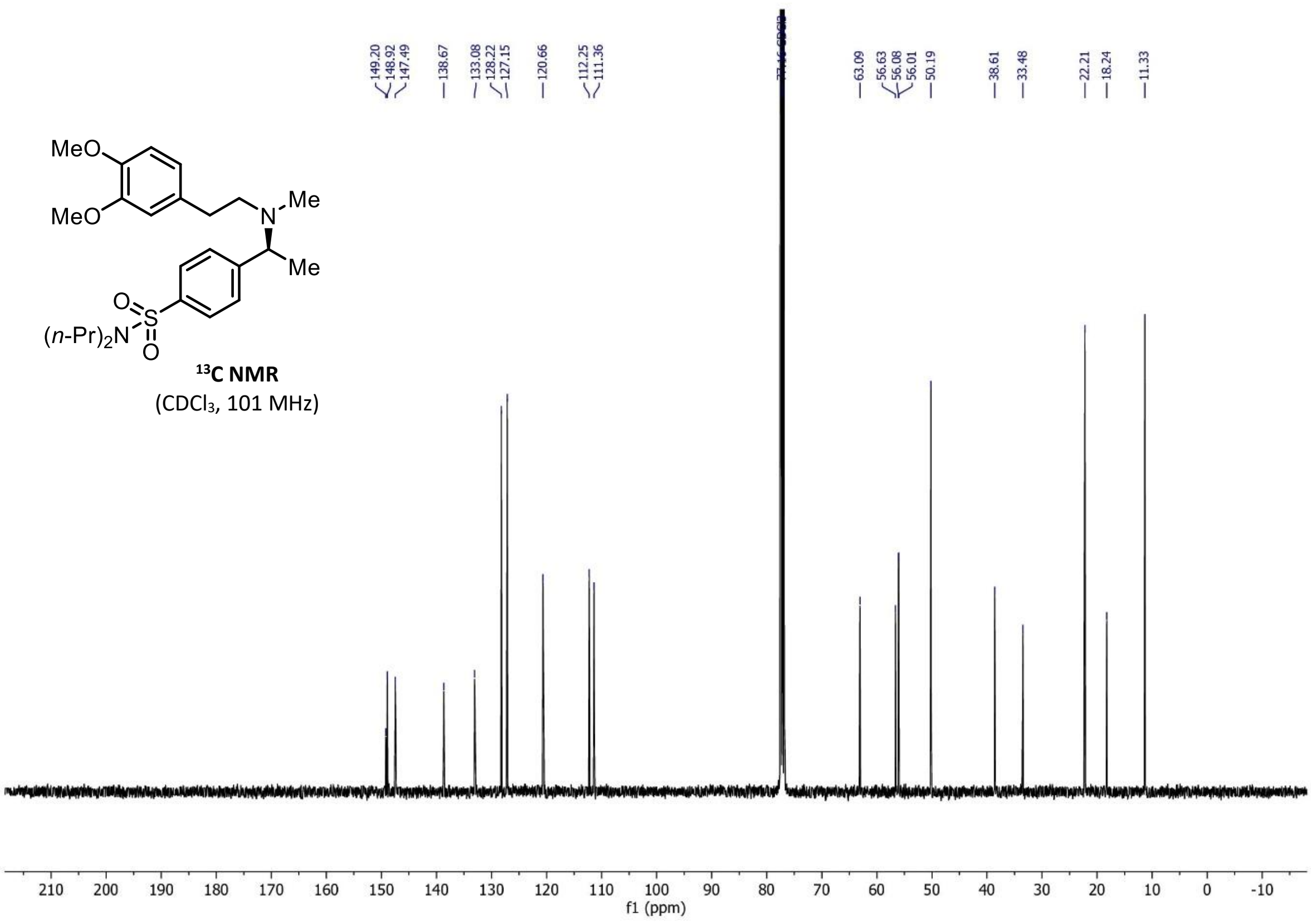

(*S*)-*N*-(3,4-Dimethoxyphenethyl)-*N*-methyl-3-(naphthalen-1-yloxy)-1-phenylpropan-1-amine (2v)

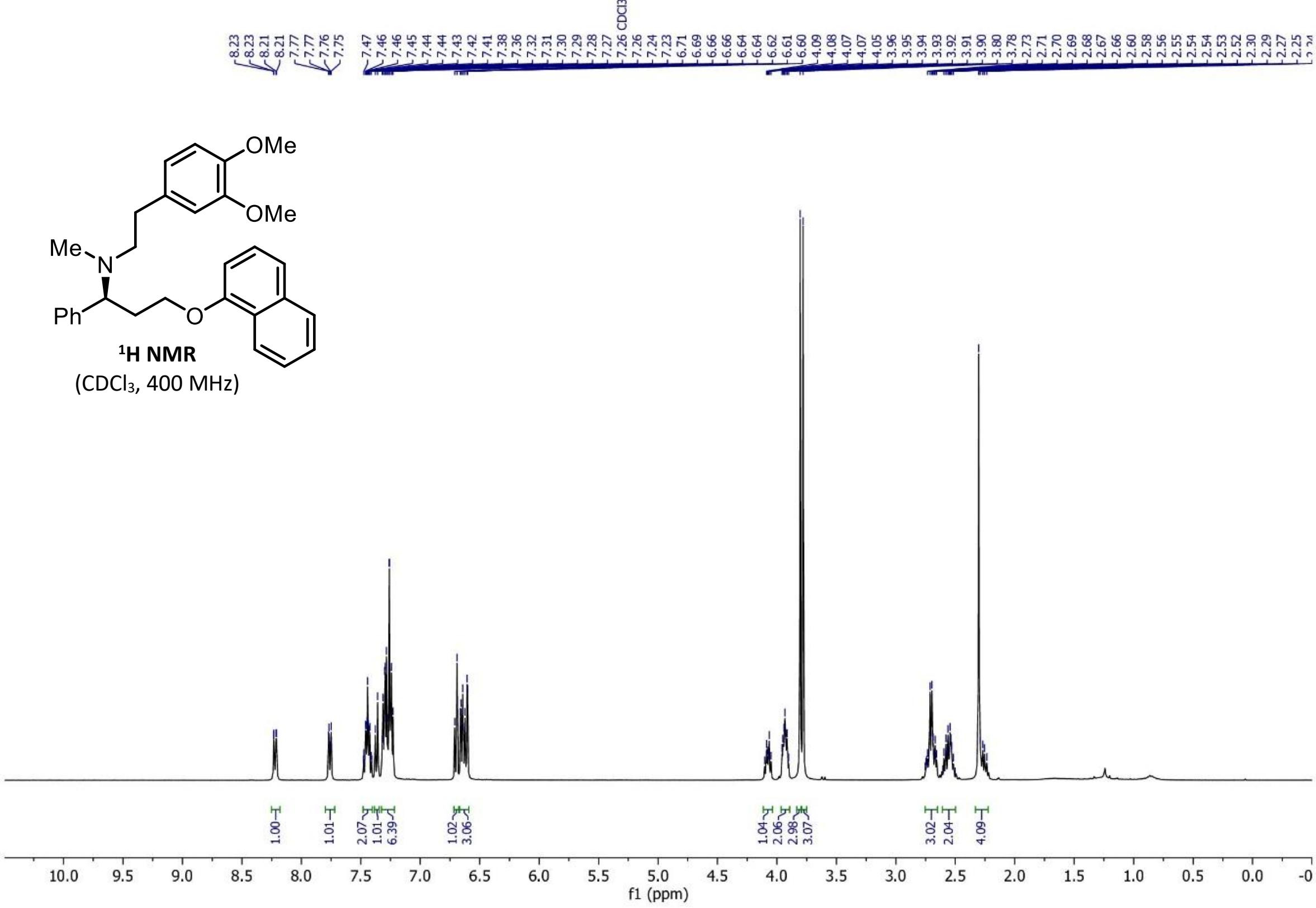

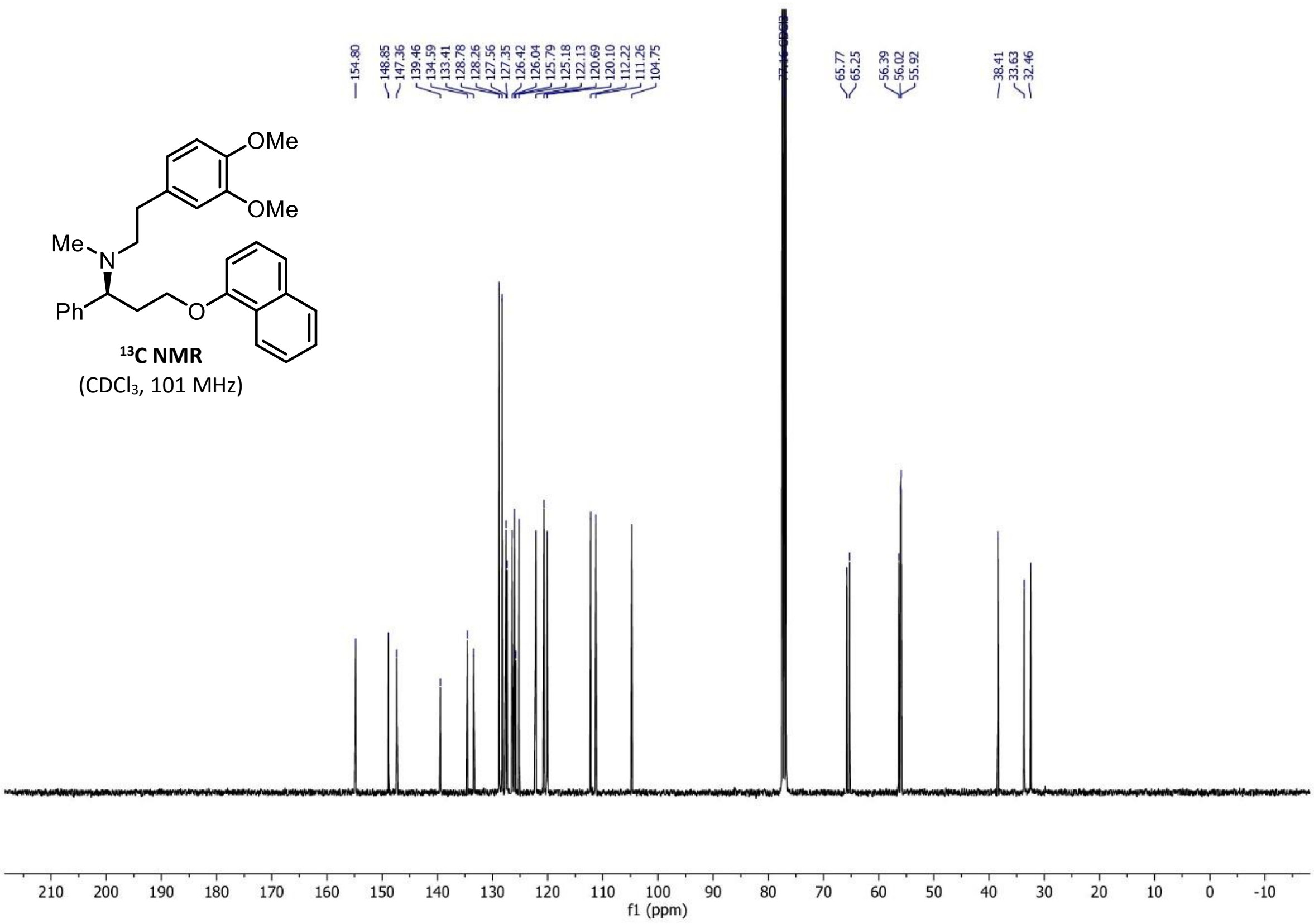

(*S*)-*N*-(3,4-Dimethoxyphenethyl)-*N*-methyl-1-phenylundec-4-yn-3-amine (2w)

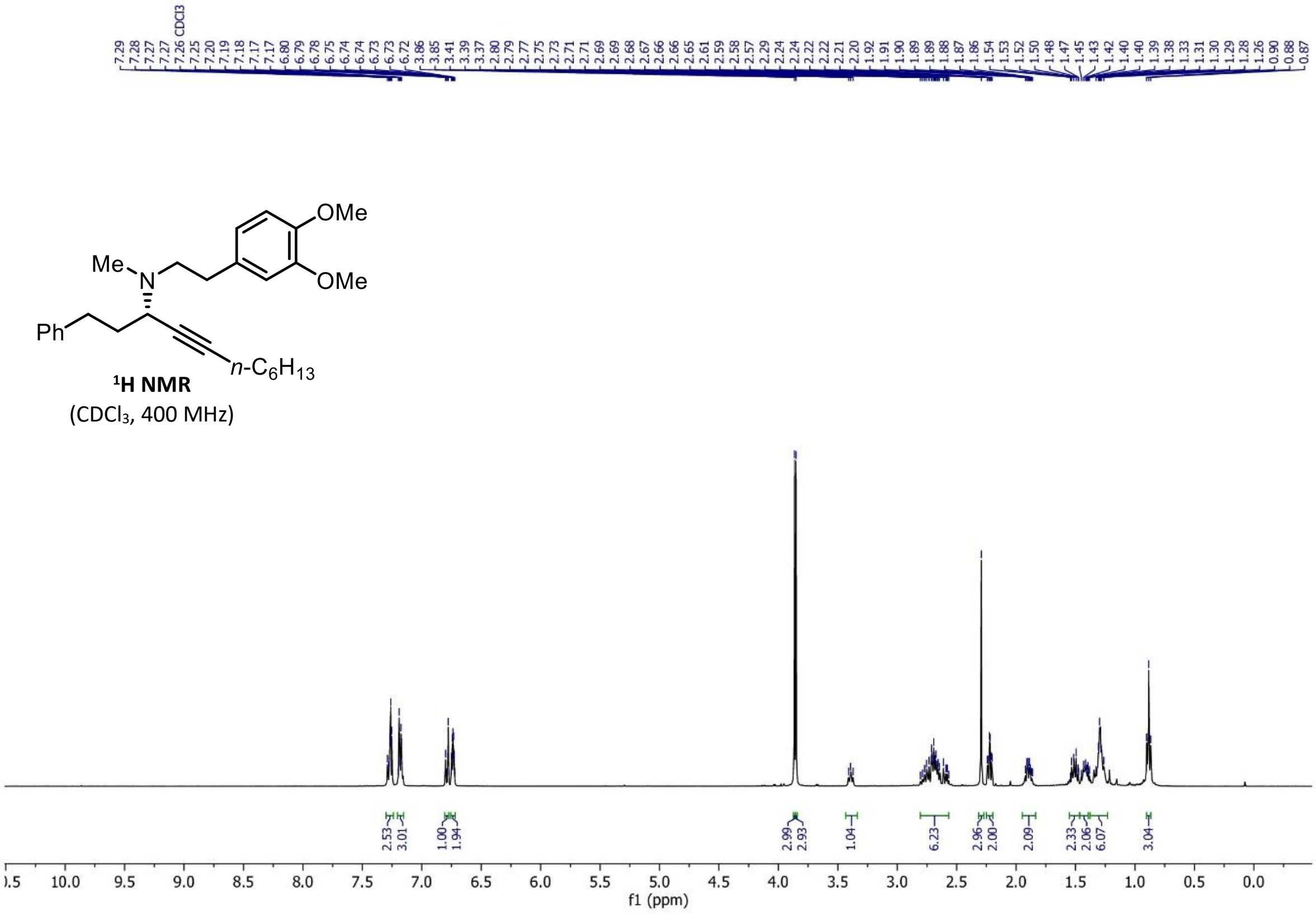

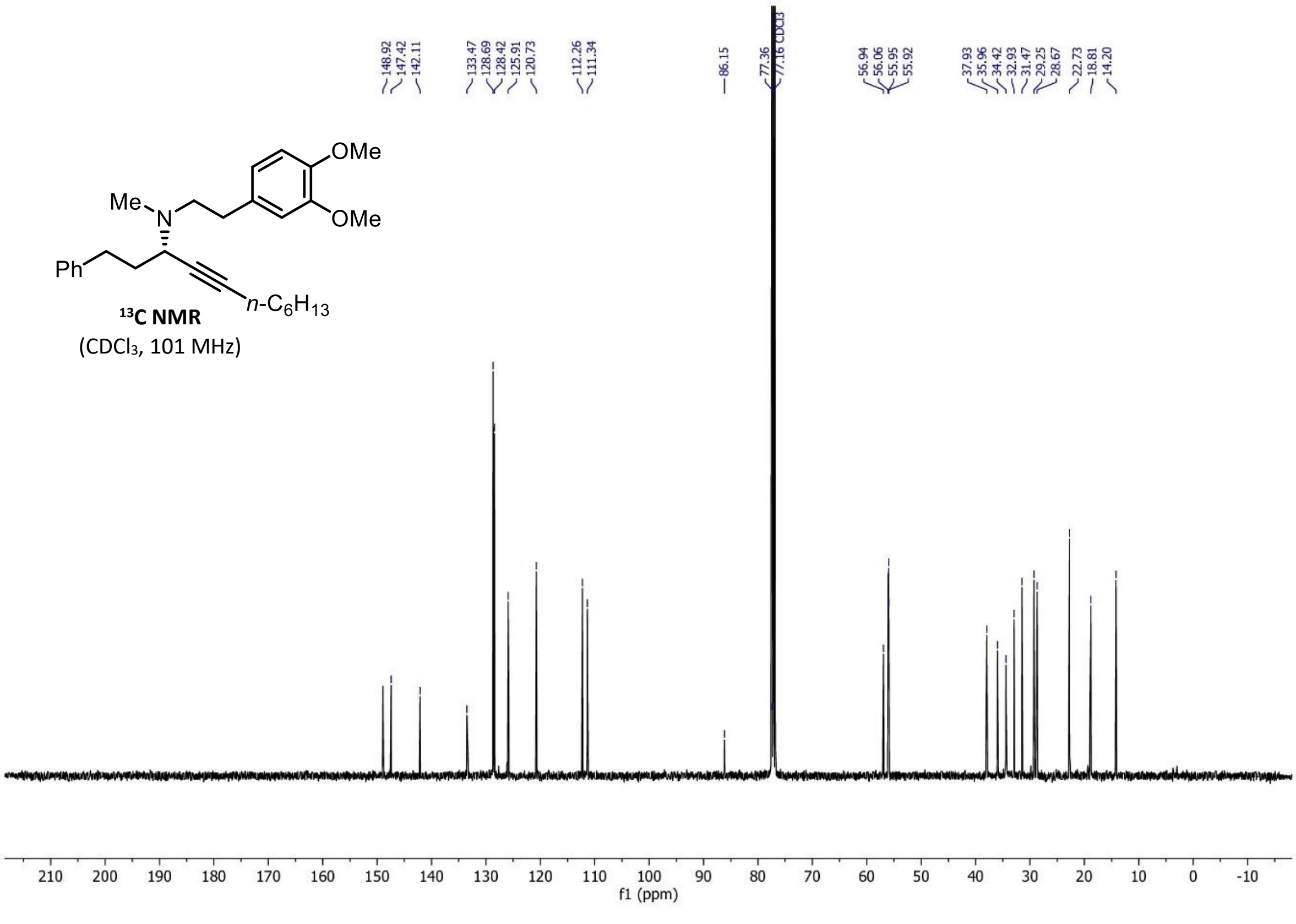

***tert*-Butyl 2-((3,4-dimethoxyphenethyl)(methyl)amino)-2-phenylacetate (2x)**

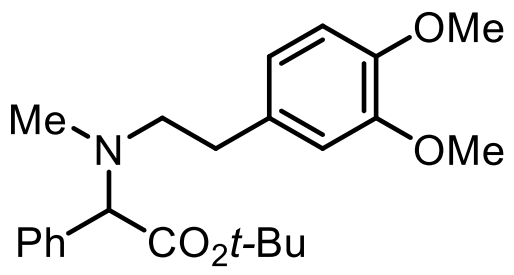

^{1}H NMR
($CDCl_3$, 400 MHz)

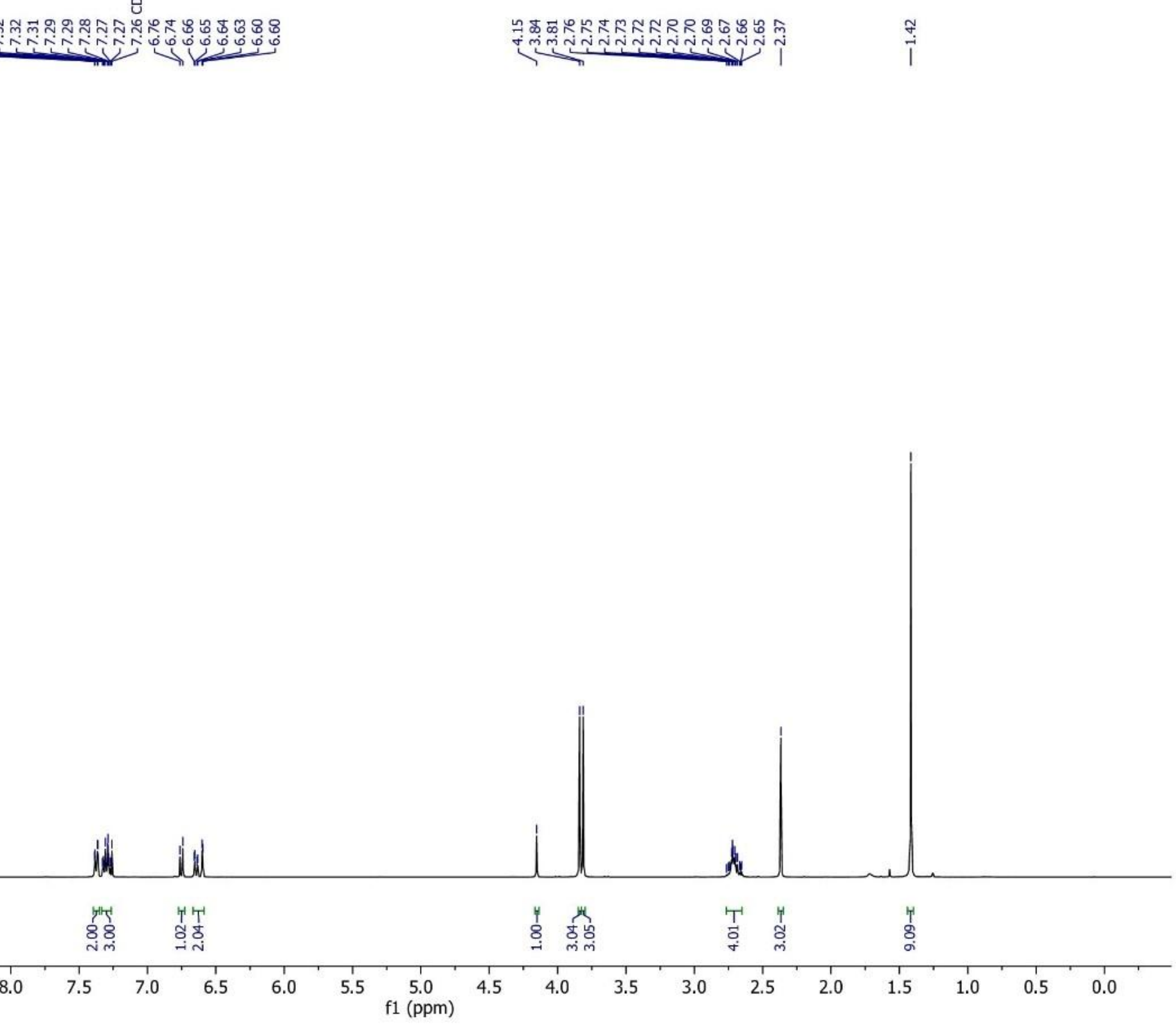

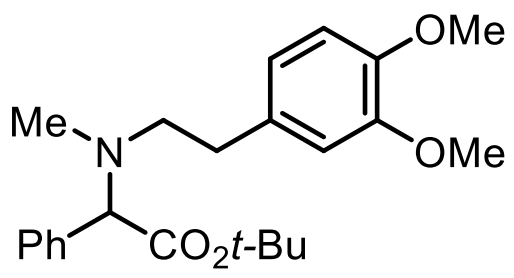

^{13}C NMR
($CDCl_3$, 101 MHz)

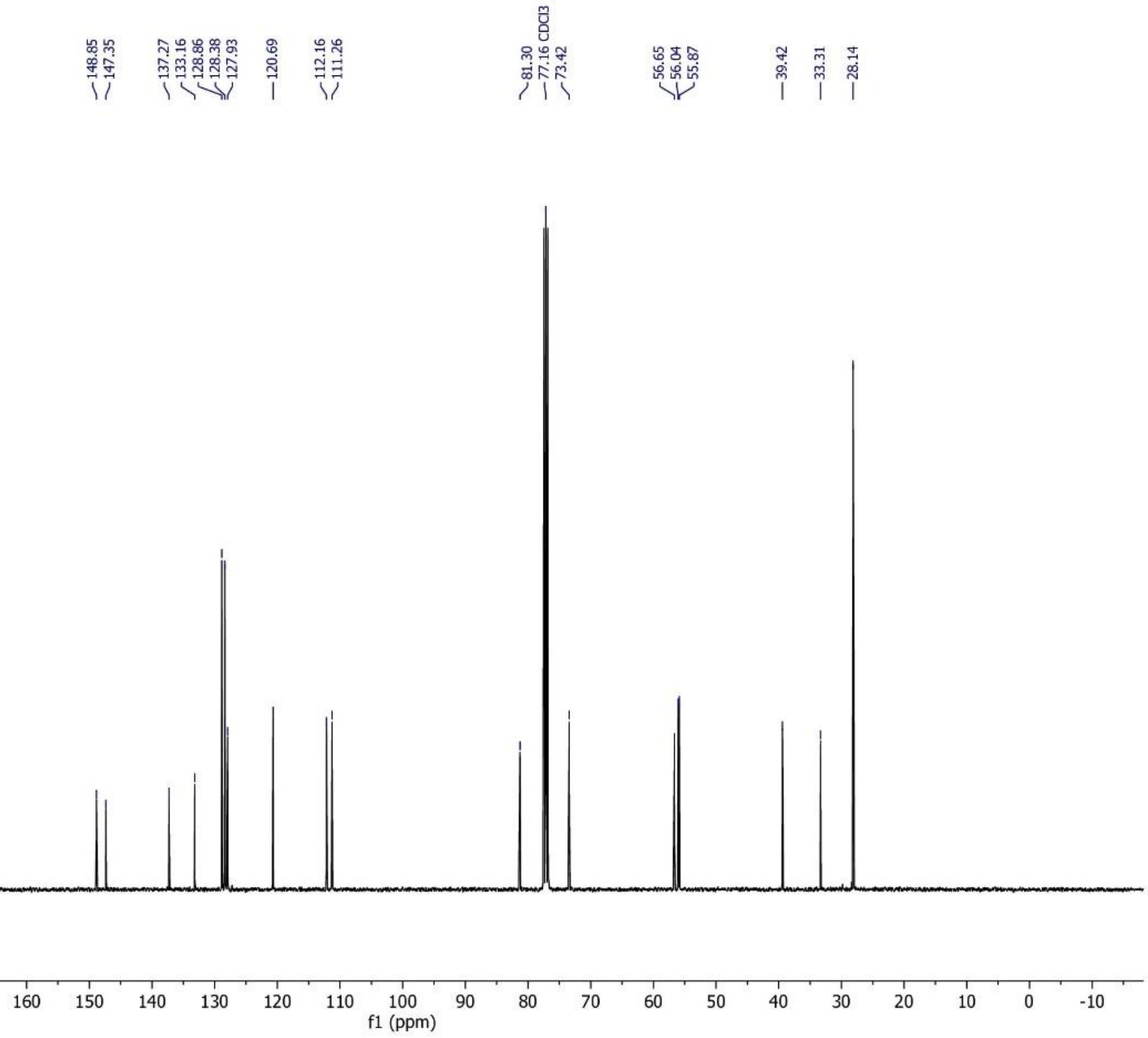

(*Z*)-*N*²,*N*²-Dibutyl-1-(2,7-dichloro-9-(4-chlorobenzylidene)-9*H*-fluoren-4-yl)-*N*¹-(3,4-dimethoxyphenethyl)-*N*¹-methylethane-1,2-diamine (2y)

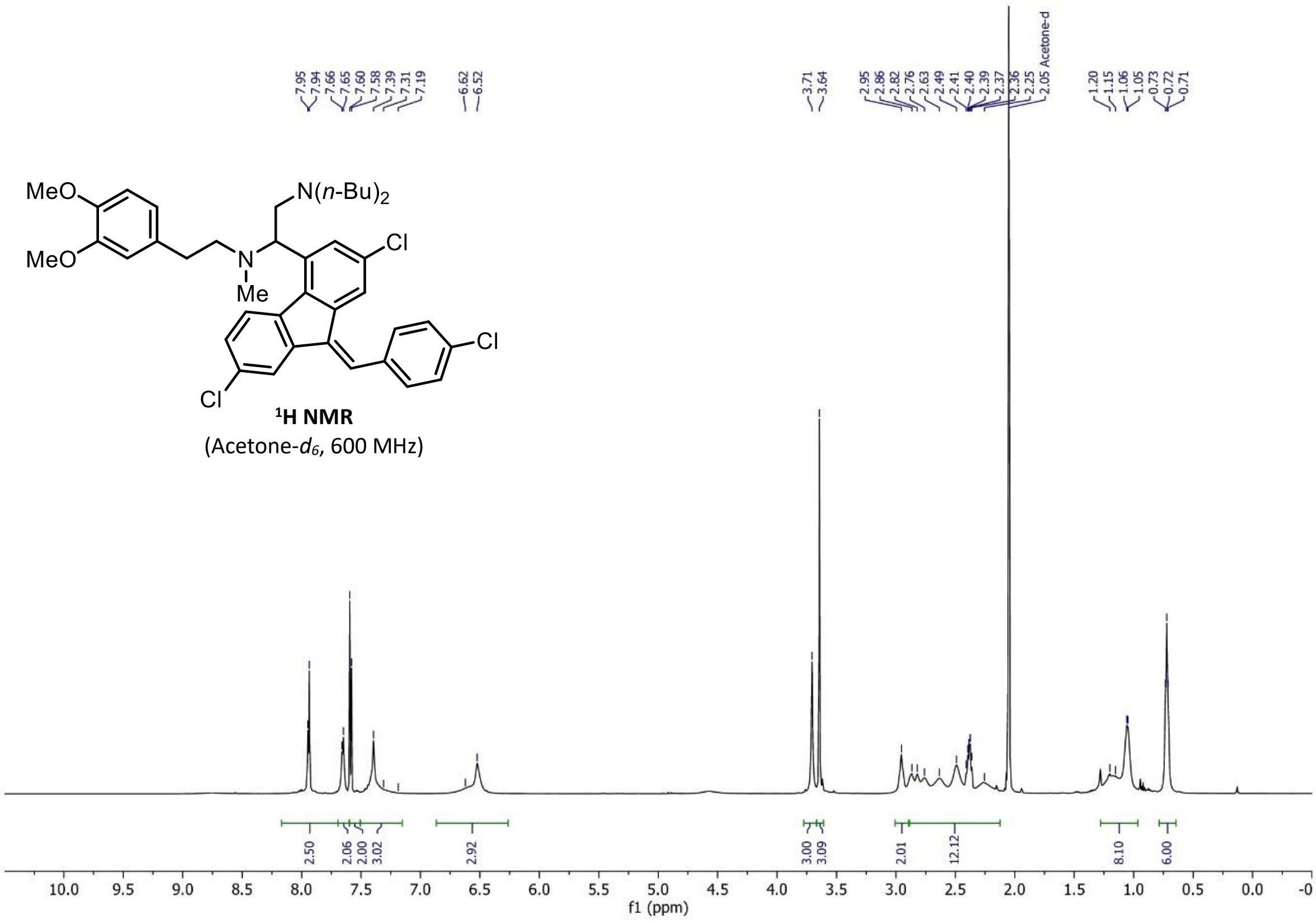

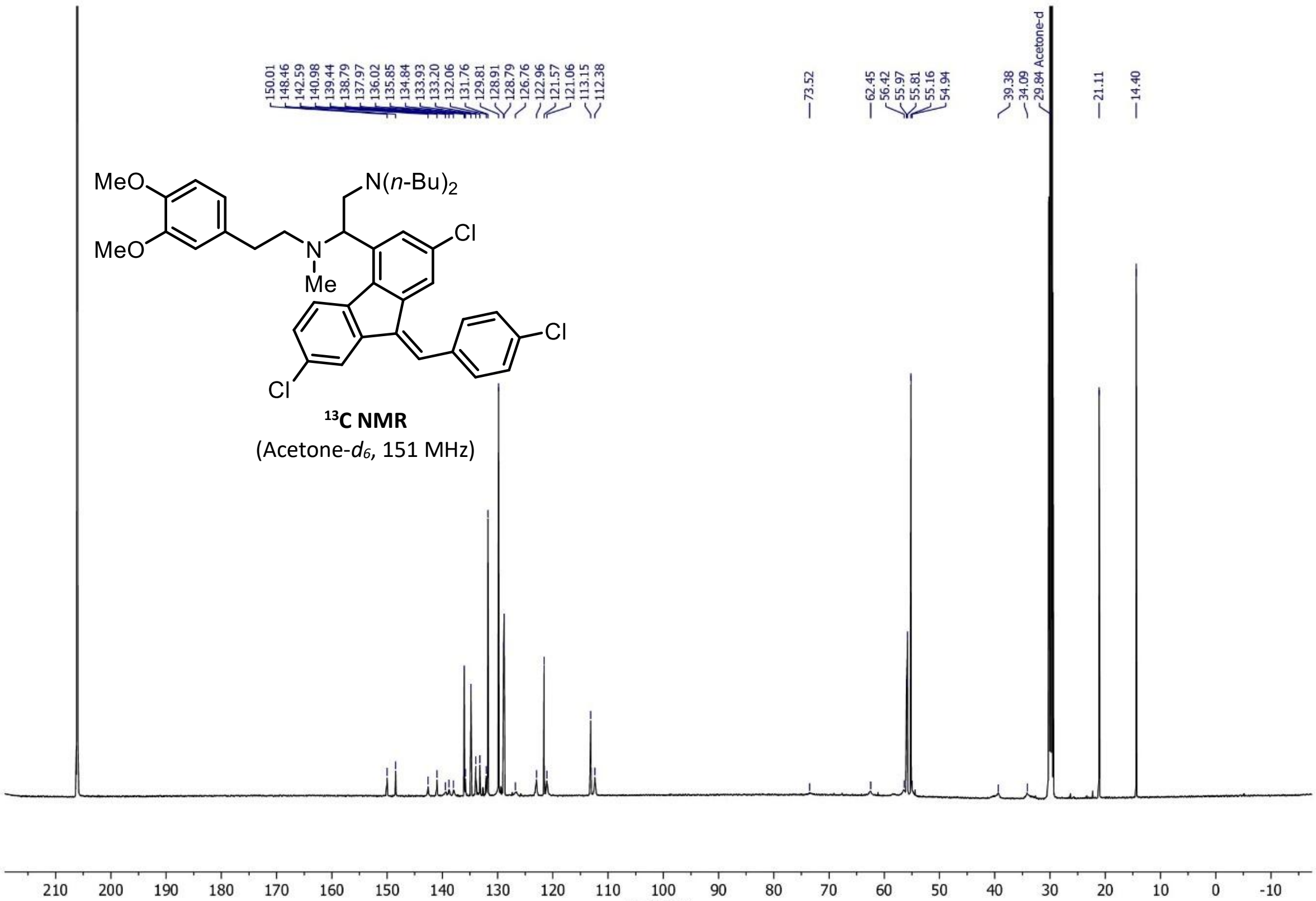

***N*-(4-Bromobenzyl)-2-phenylethan-1-amine (3a)**

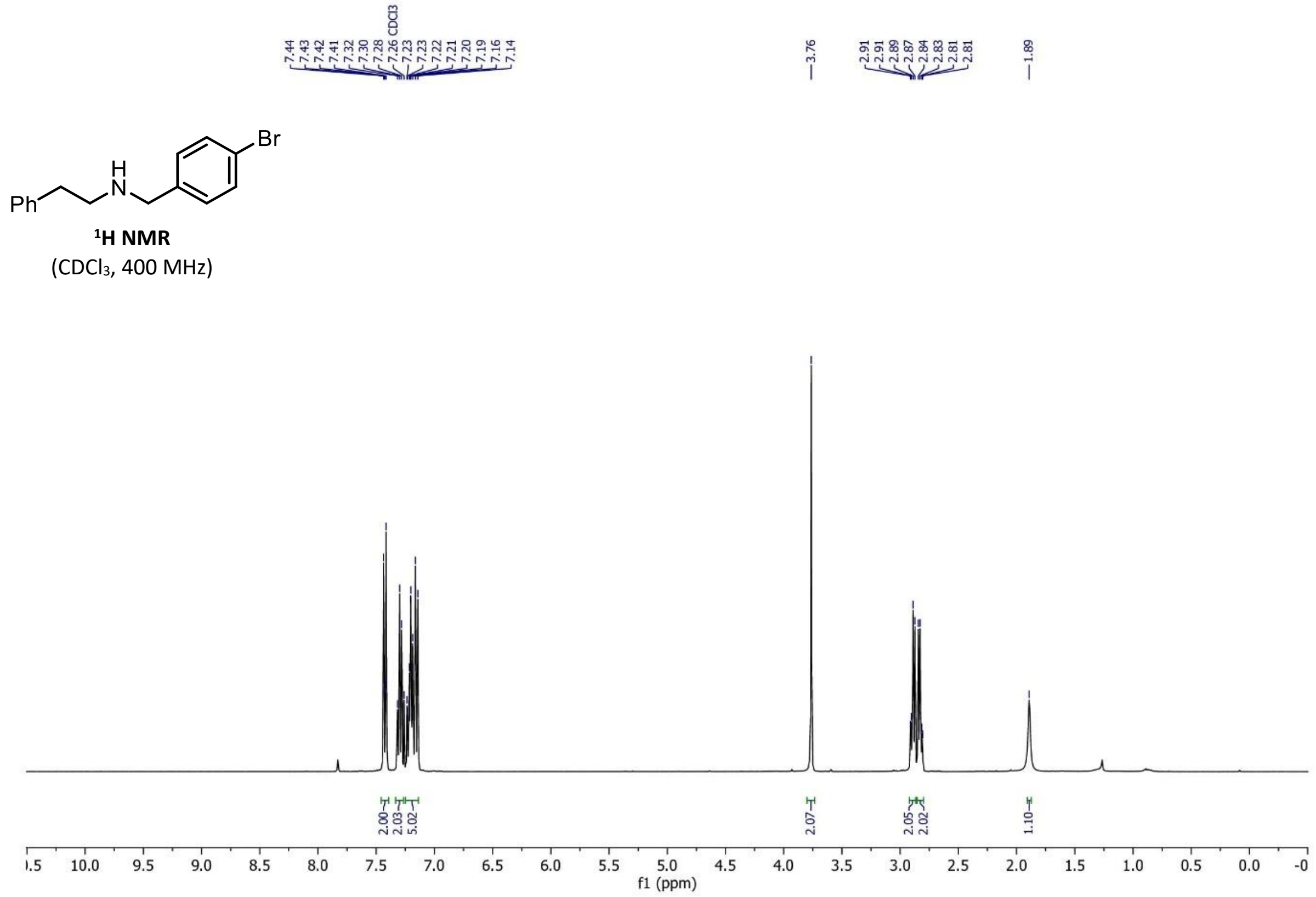

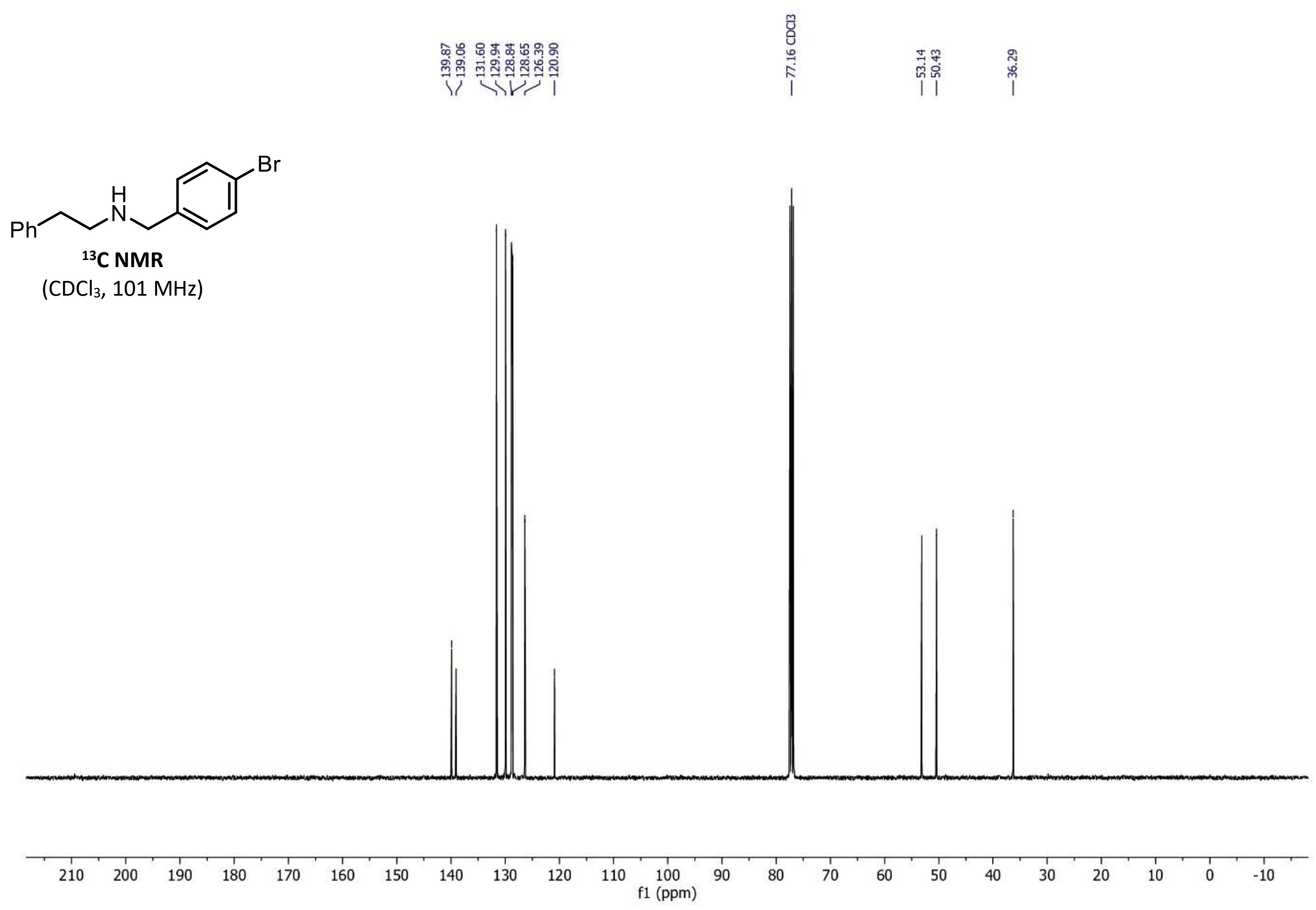

tert-Butyl (4-((4-bromobenzyl)amino)butyl)carbamate (3b)

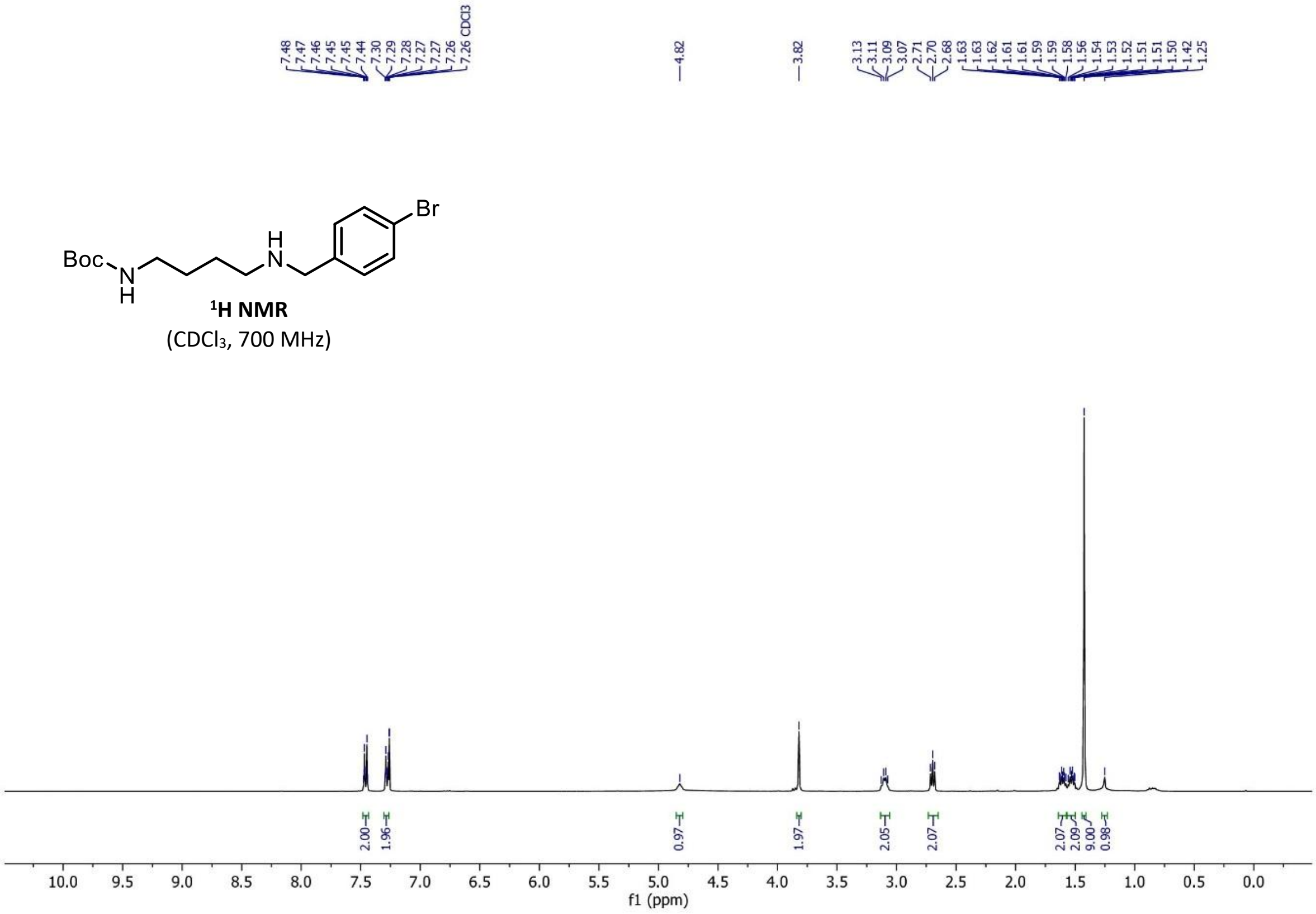

^{1}H NMR
($CDCl_3$, 700 MHz)

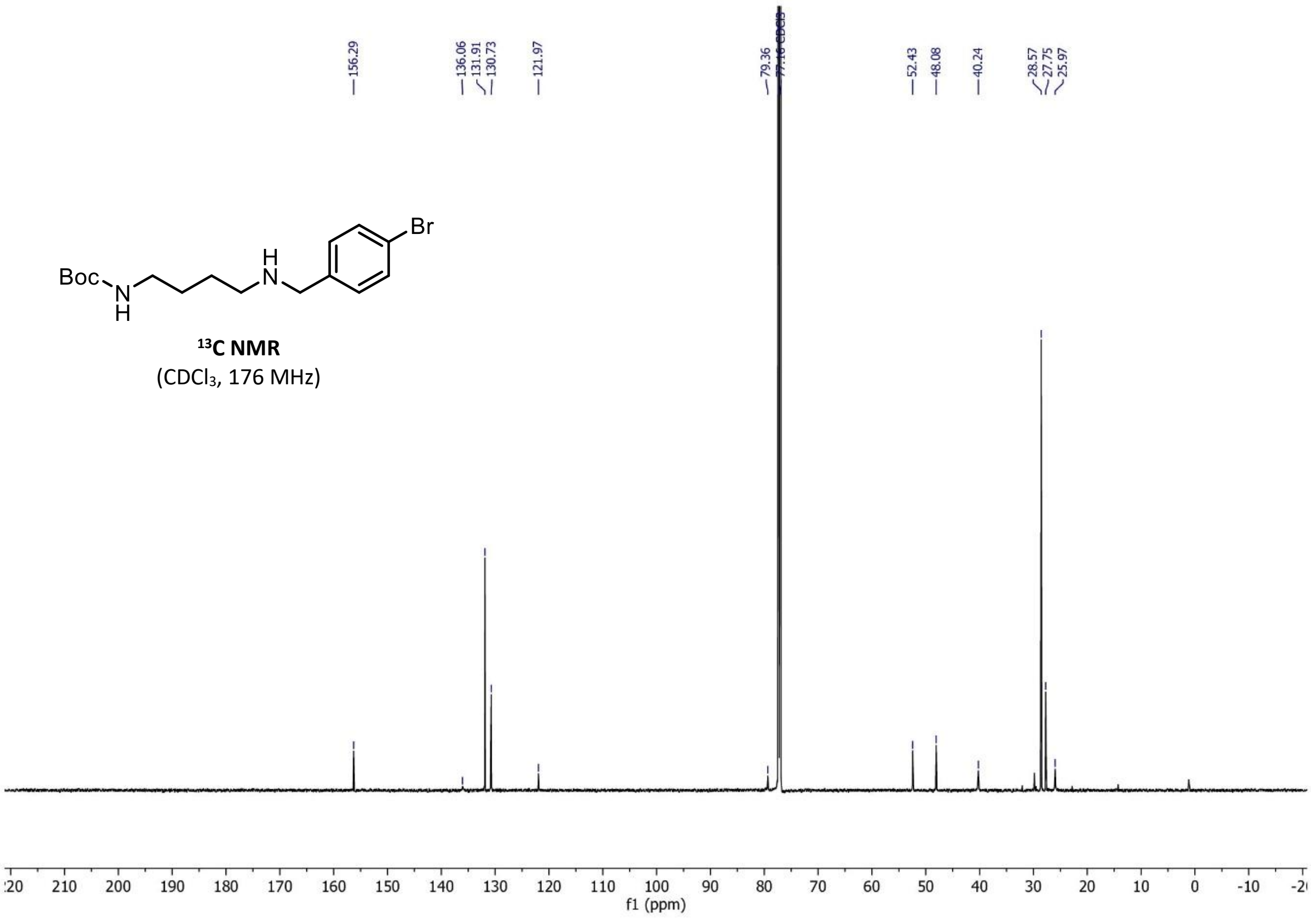

^{13}C NMR
($CDCl_3$, 176 MHz)

_N_-(4-Bromobenzyl)-1-(thiophen-2-yl)methanamine (3c)

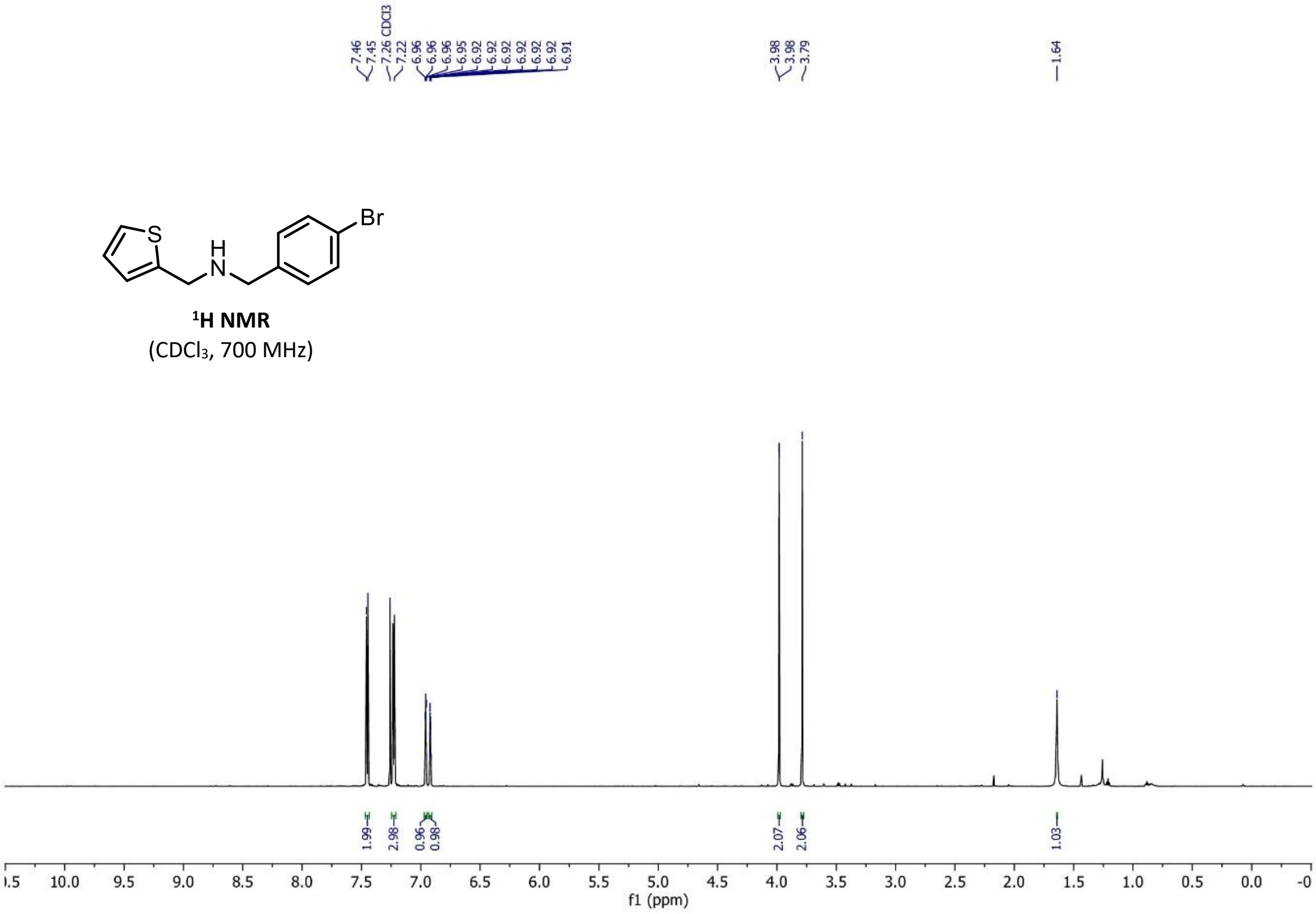

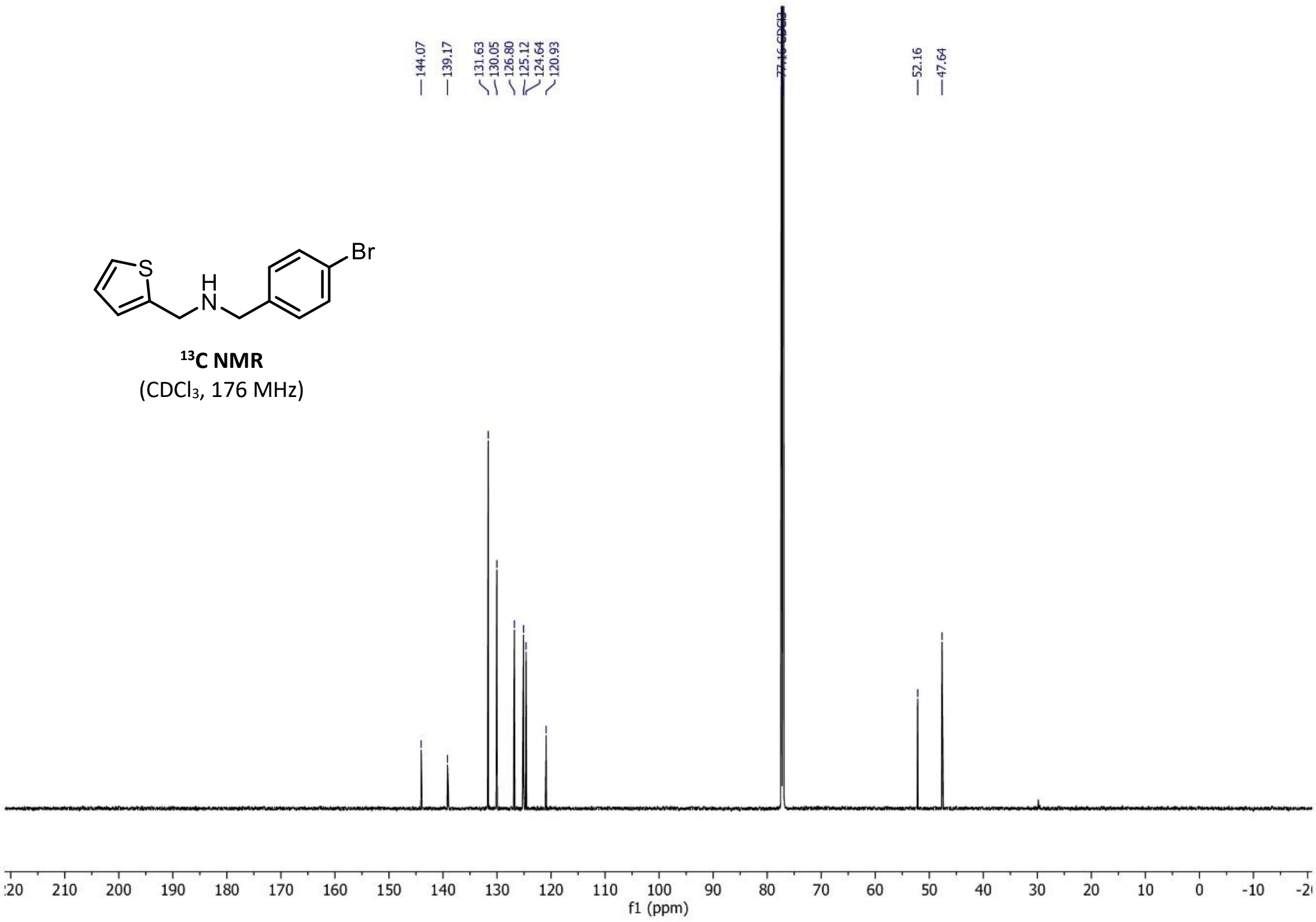

rac-(1*R*,2*S*,5*R*)-*N*-(4-Bromobenzyl)-2-isopropyl-5-methylcyclohexan-1-amine (3d)

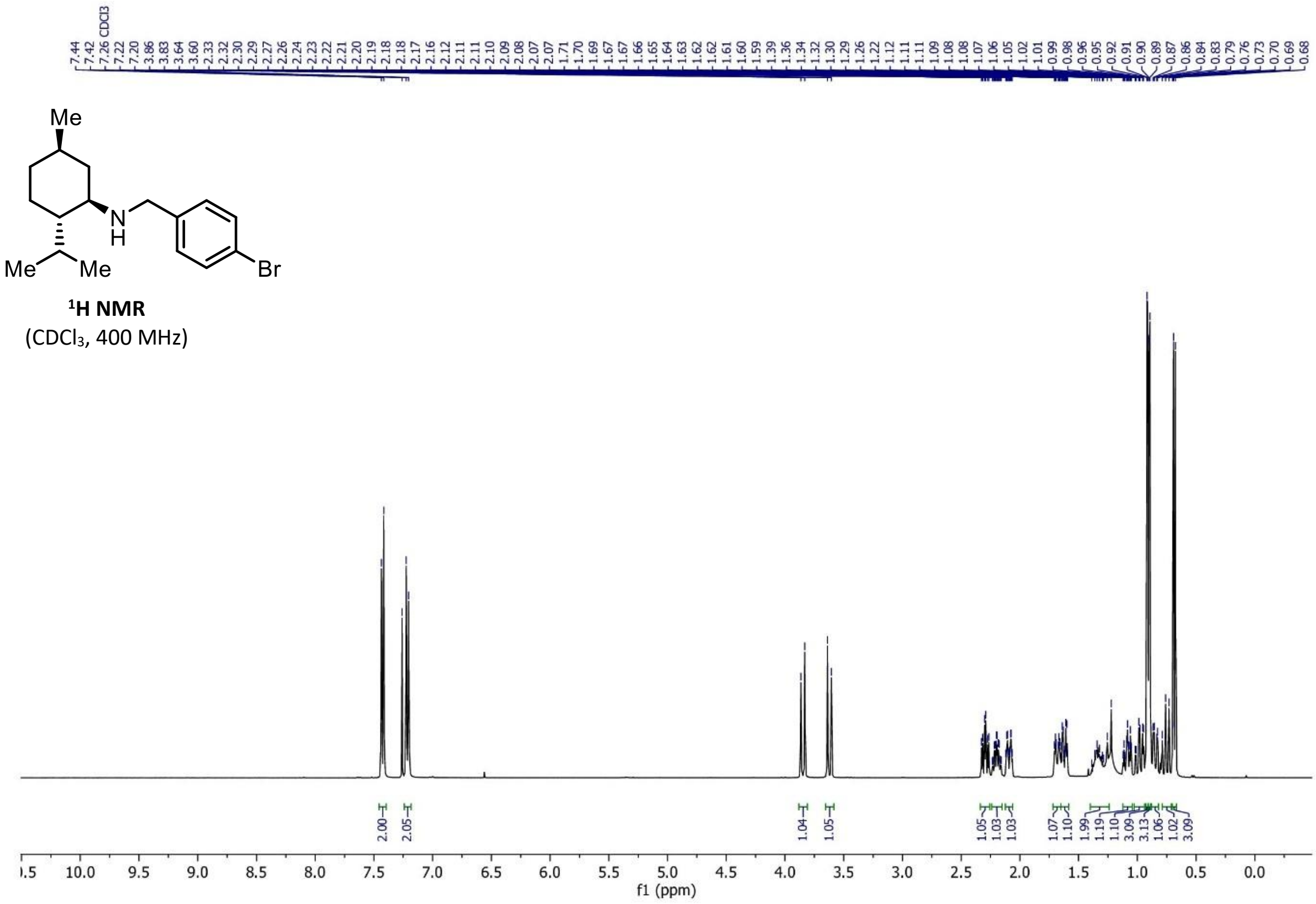

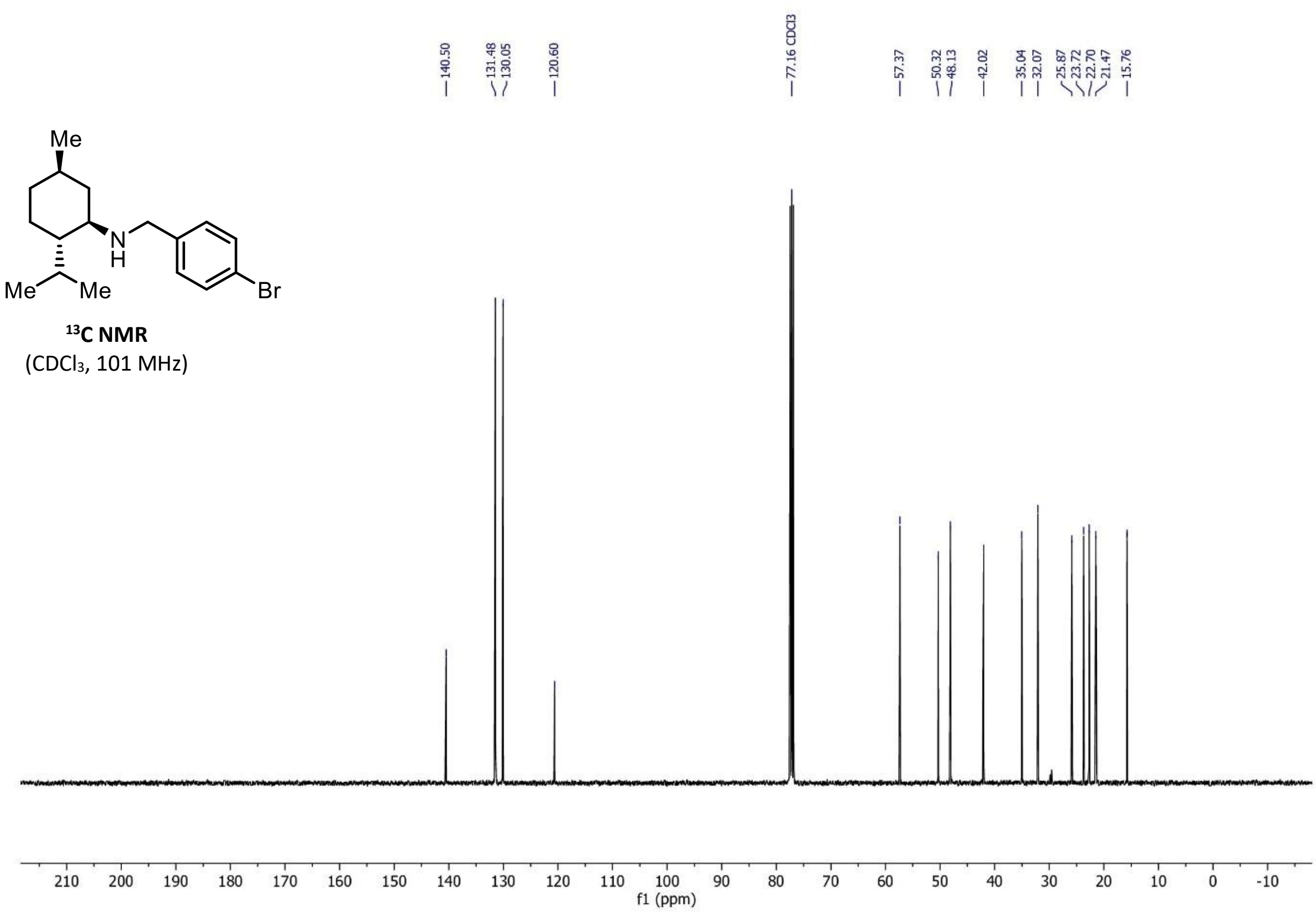

(*R*)-3-((4-Bromobenzyl)amino)-1-(3-(trifluoromethyl)-5,6-dihydro-[1,2,4]triazolo[4,3-*a*]pyrazin-7(8*H*)-yl)-4-(2,4,5-trifluorophenyl)butan-1-one (3e)

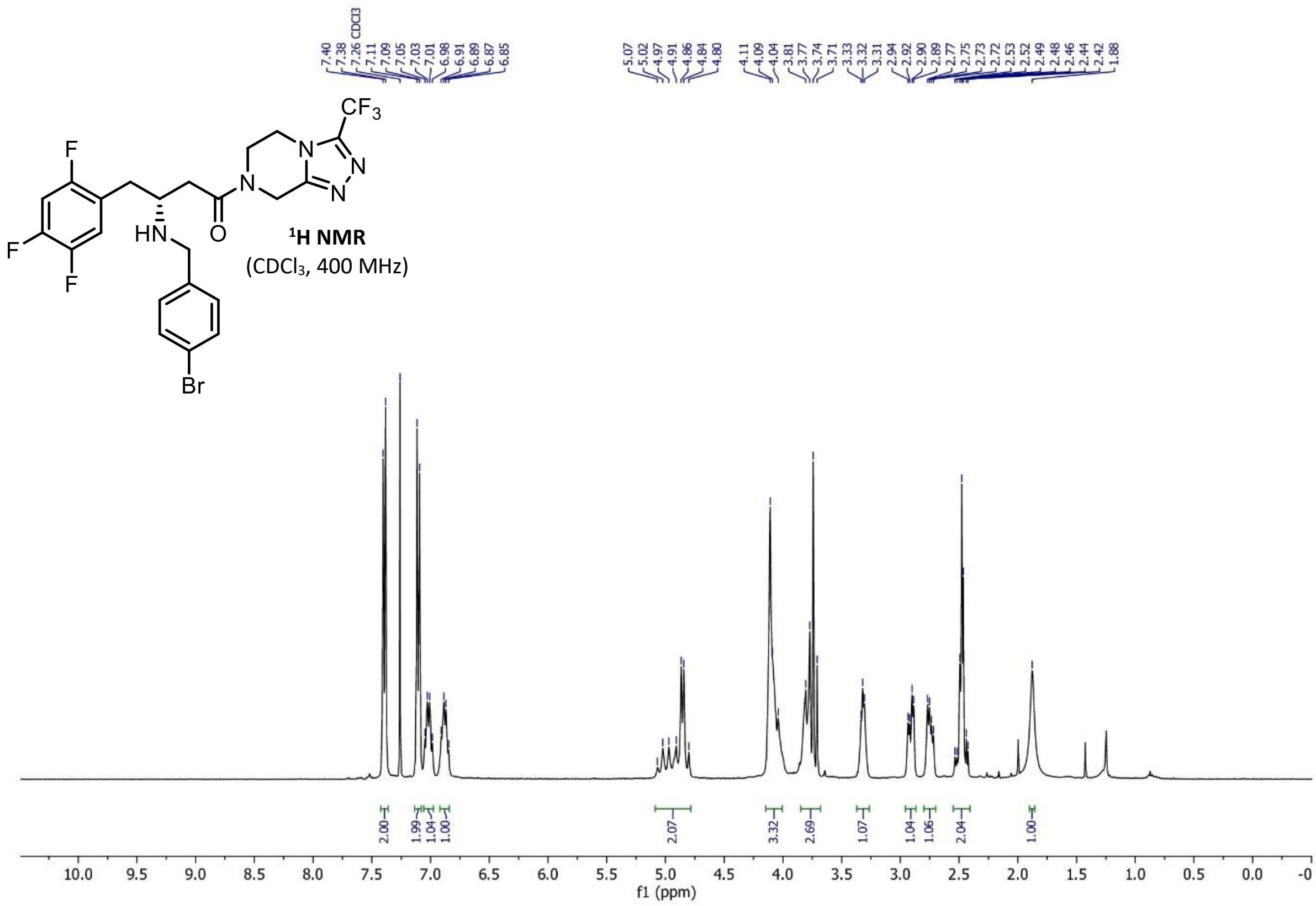

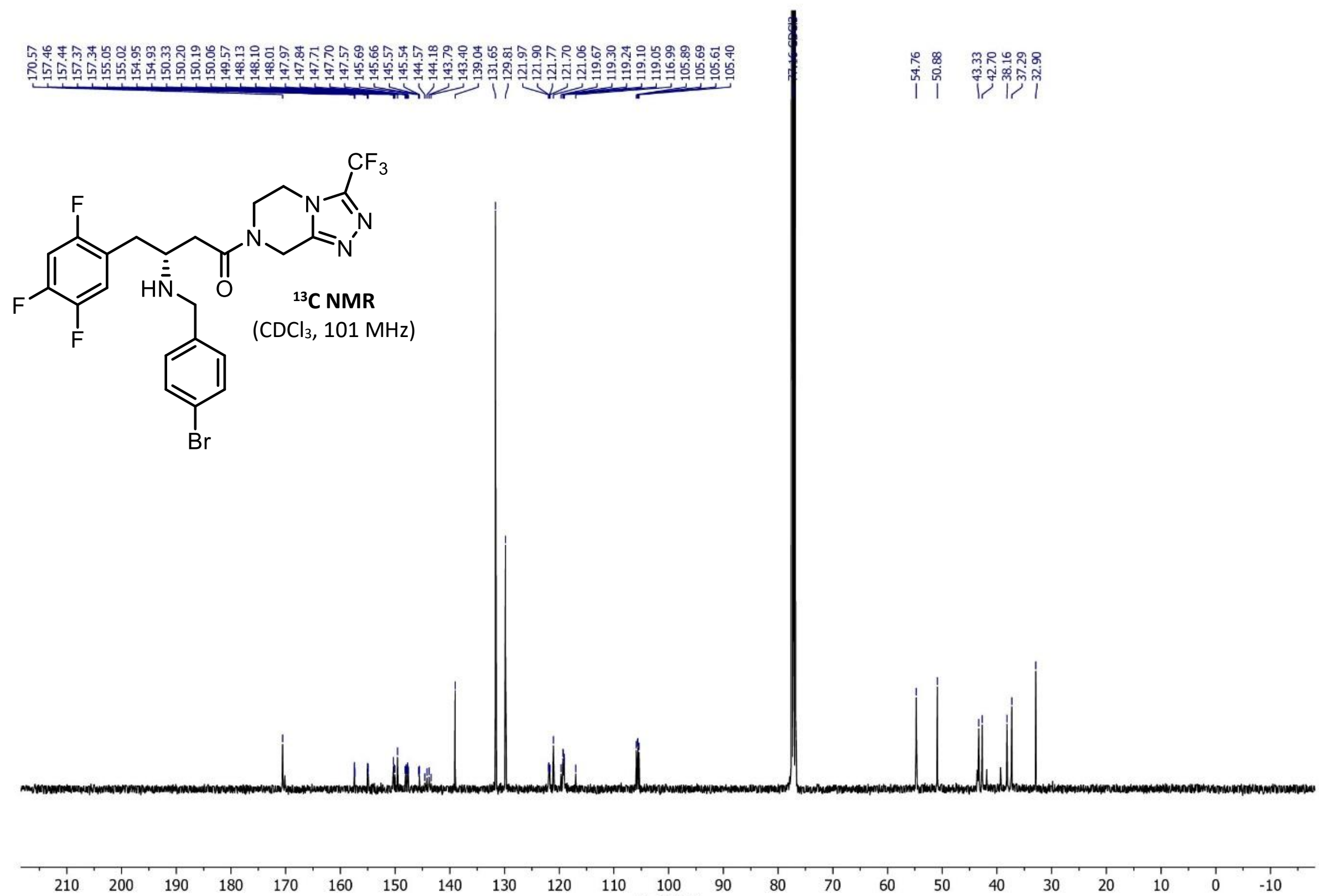

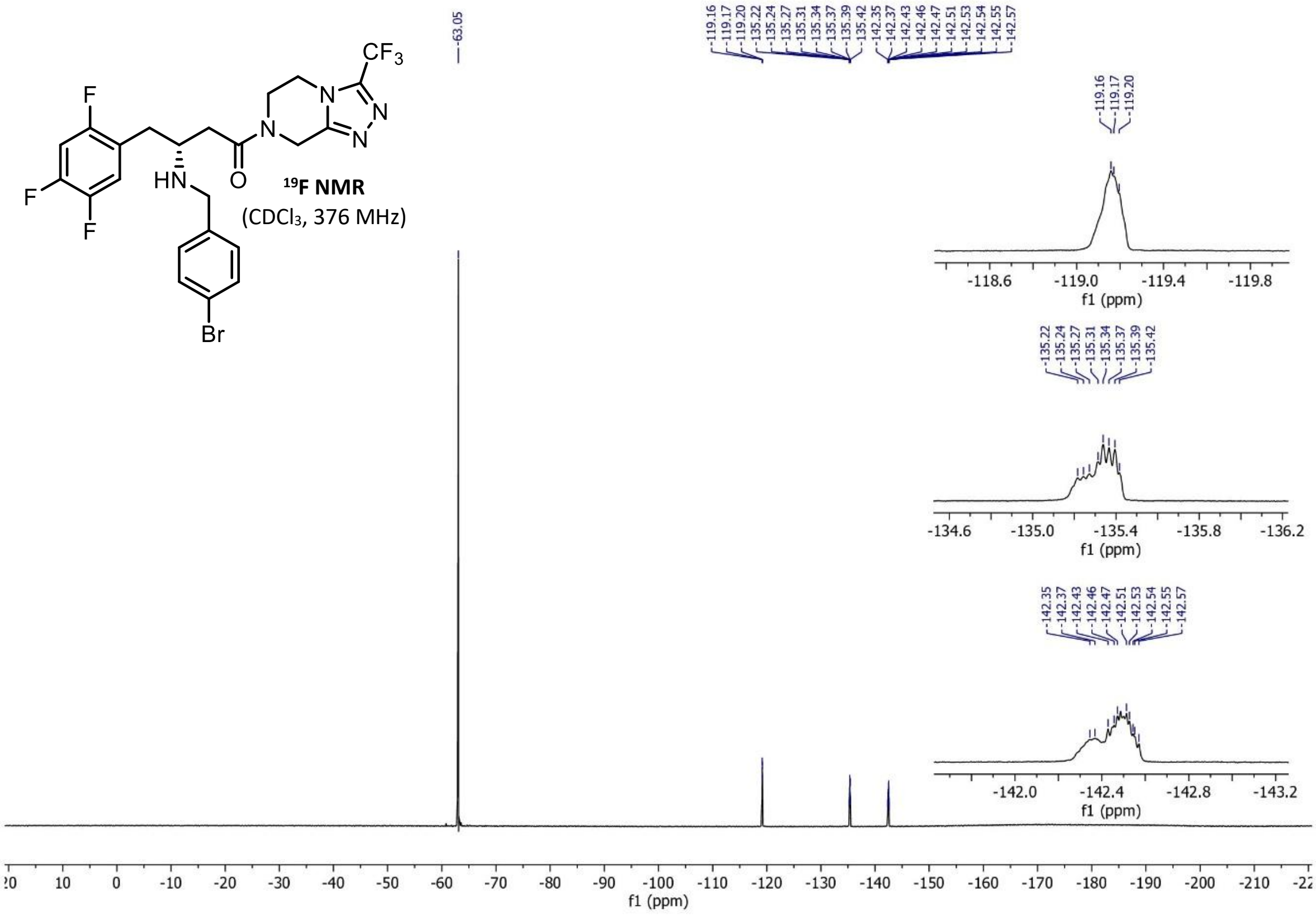
¹⁹F NMR
(CDCl₃, 376 MHz)
CF₃
N
F
HN
O
Br
-63.05
-119.16
-119.17
-119.20
-135.22
-135.24
-135.27
-135.31
-135.34
-135.37
-135.39
-135.42
-142.35
-142.37
-142.43
-142.46
-142.47
-142.51
-142.53
-142.54
-142.55
-142.57
f1 (ppm)

(*R*)-2-((6-(3-((4-Bromobenzyl)amino)piperidin-1-yl)-3-methyl-2,4-dioxo-3,4-dihydropyrimidin-1(2*H*)-yl)methyl)benzonitrile (3f)

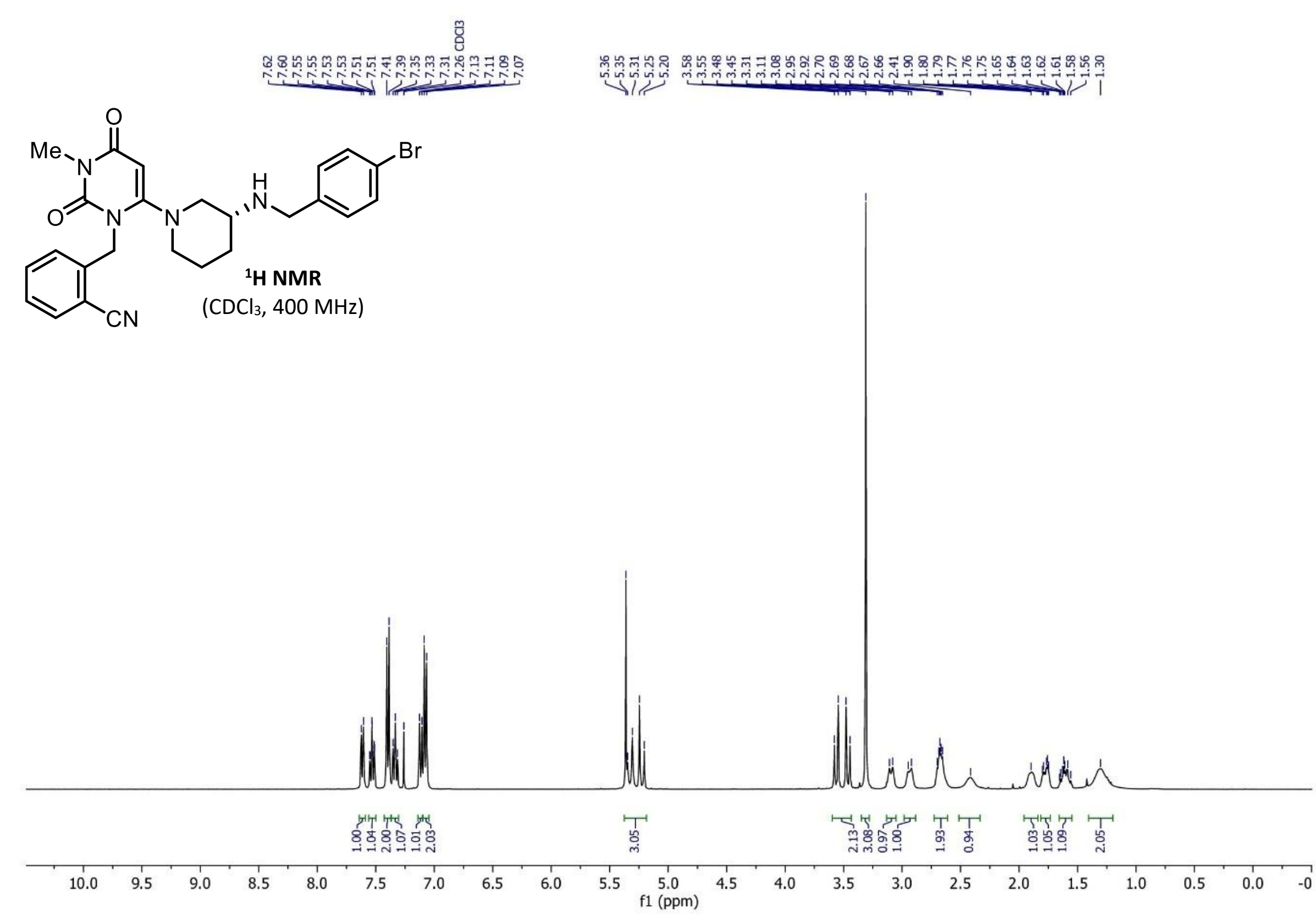

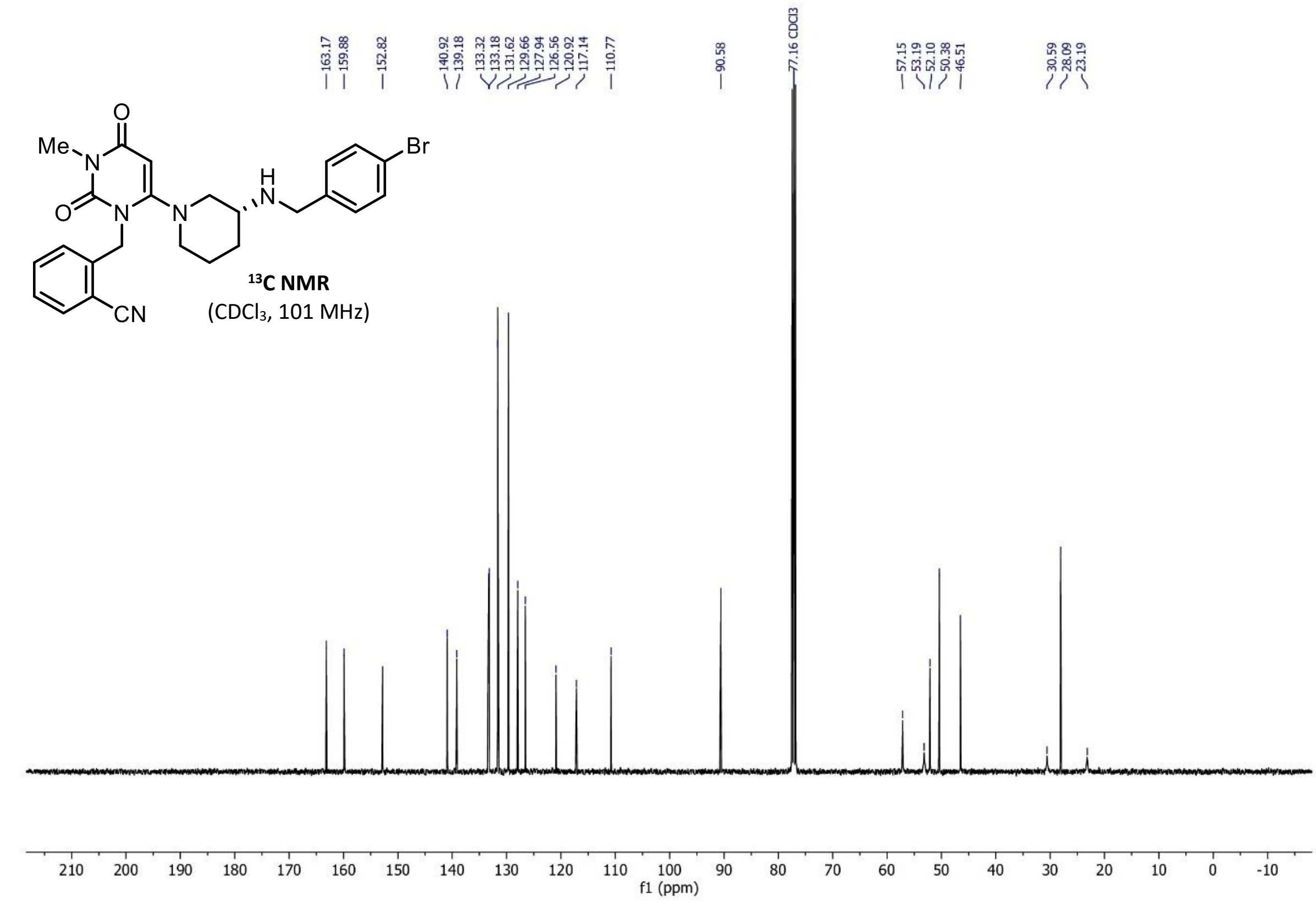

N^1-(4-Bromobenzyl)-N^4-(6-methoxyquinolin-8-yl)pentane-1,4-diamine (3g)

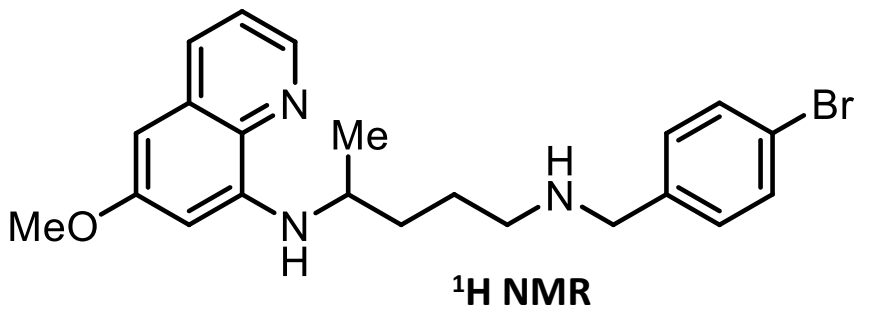

^{1}H NMR
($CDCl_3$, 400 MHz)

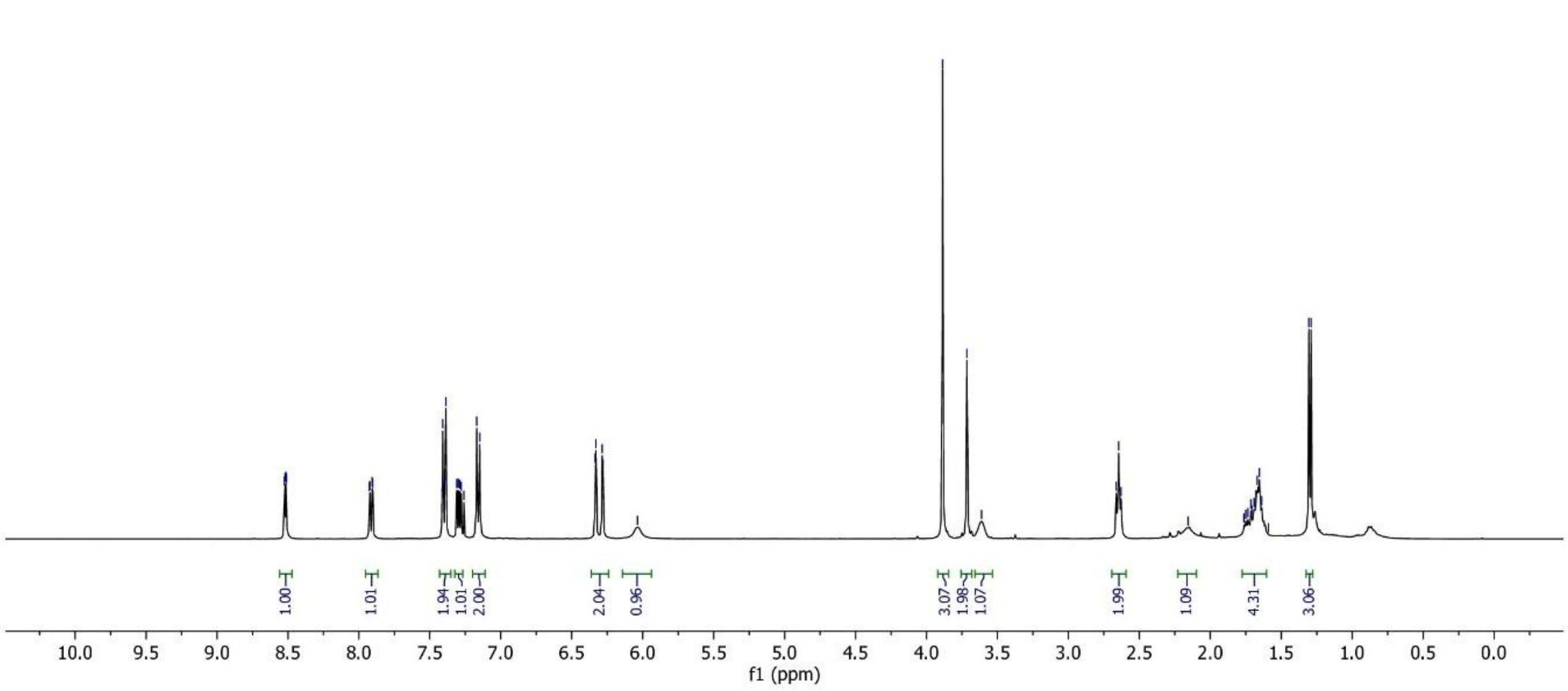

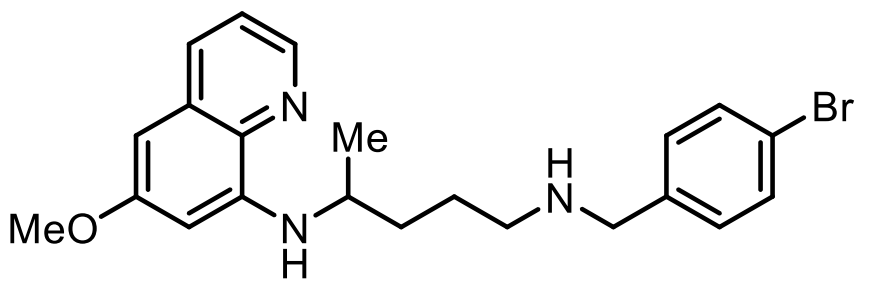

^{13}C NMR
($CDCl_3$, 101 MHz)

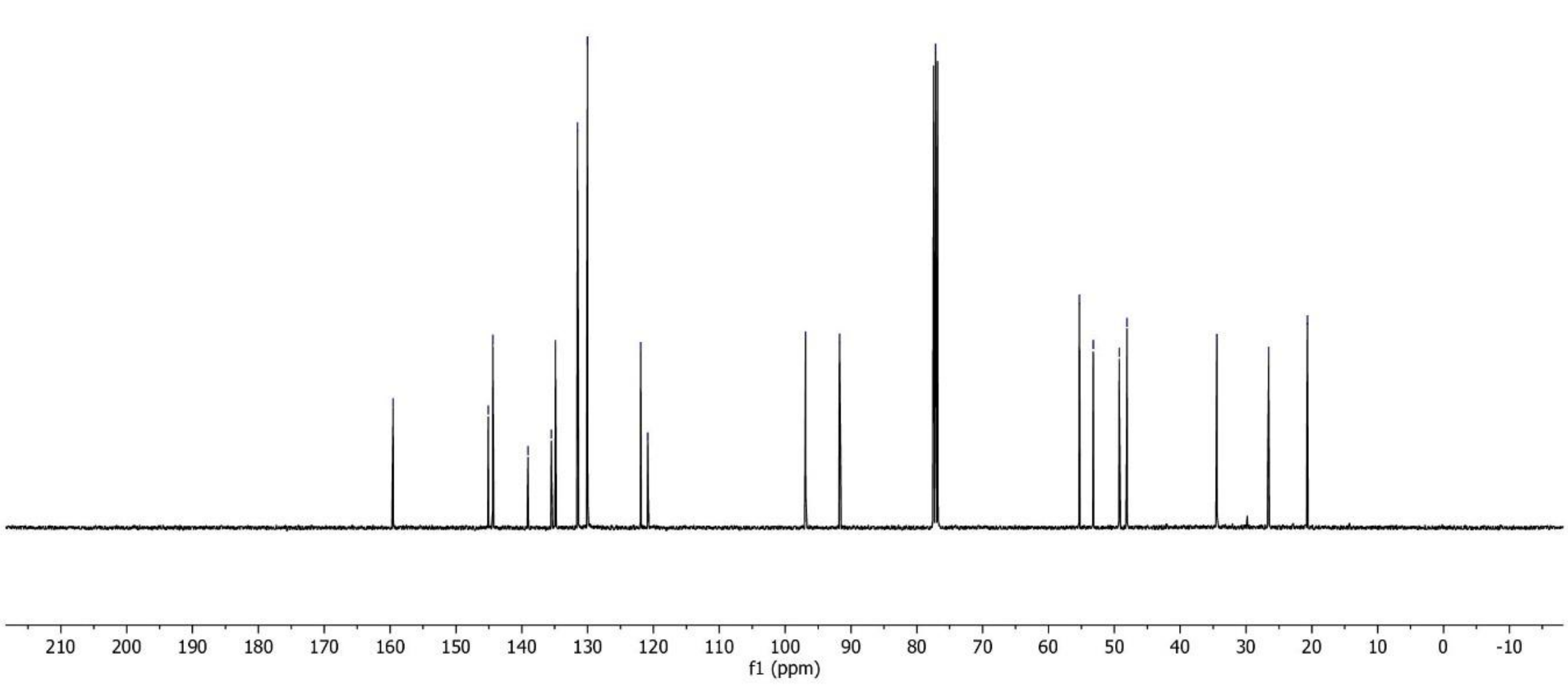

***N*-(4-Bromobenzyl)-4-methoxy-*N*-methylaniline (3h)**

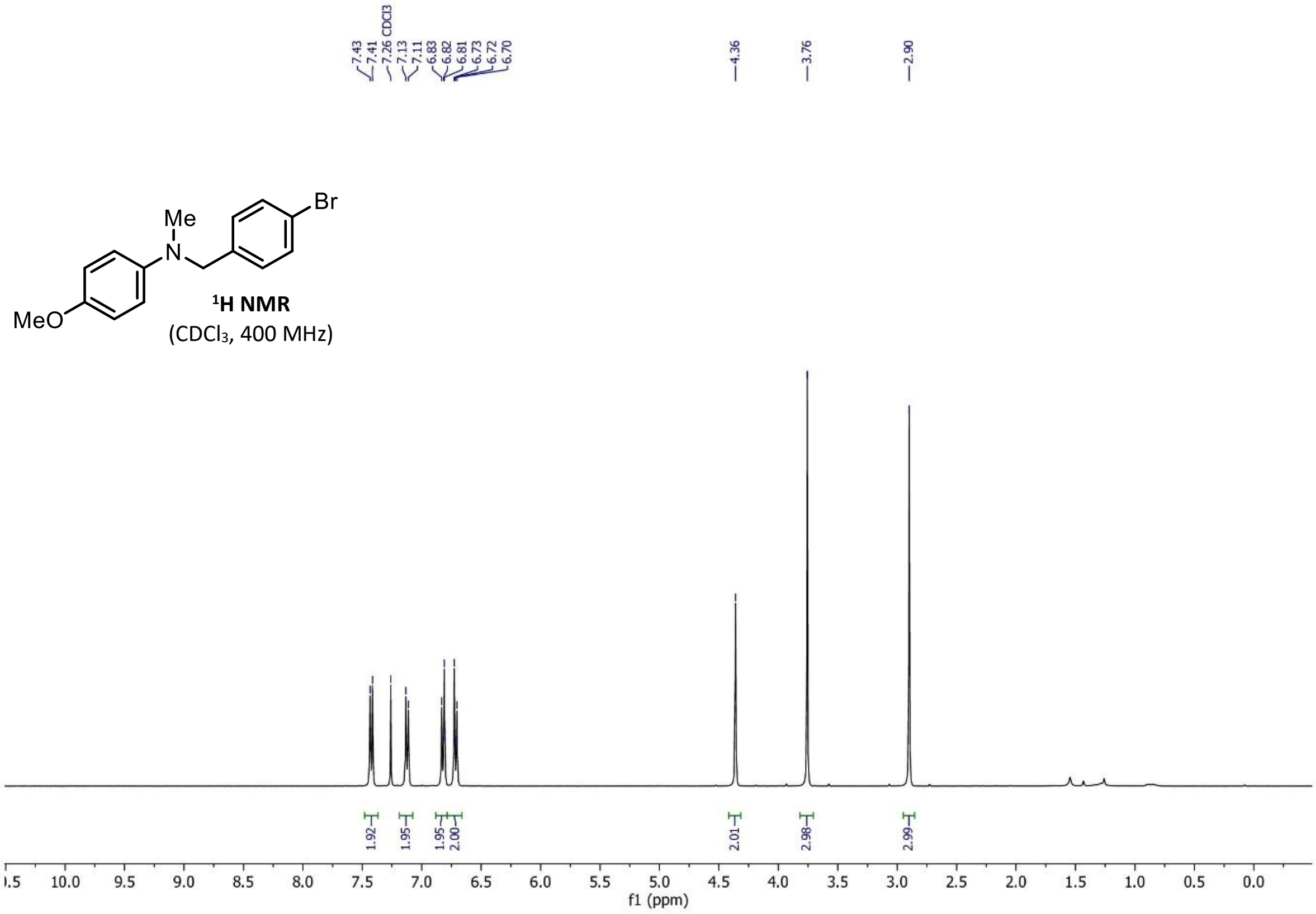

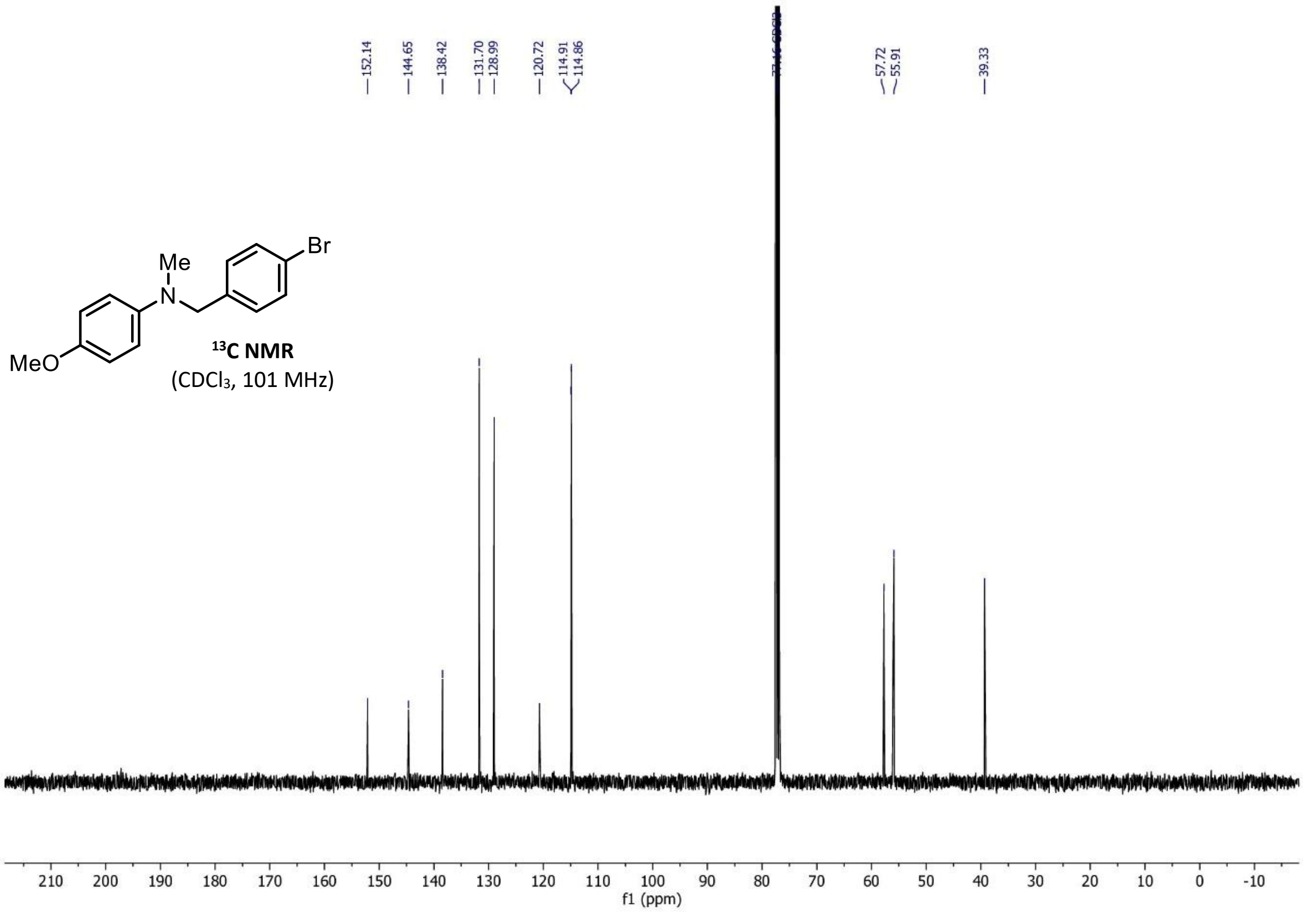

***tert*-Butyl 4-(4-bromobenzyl)piperazine-1-carboxylate (3i)**

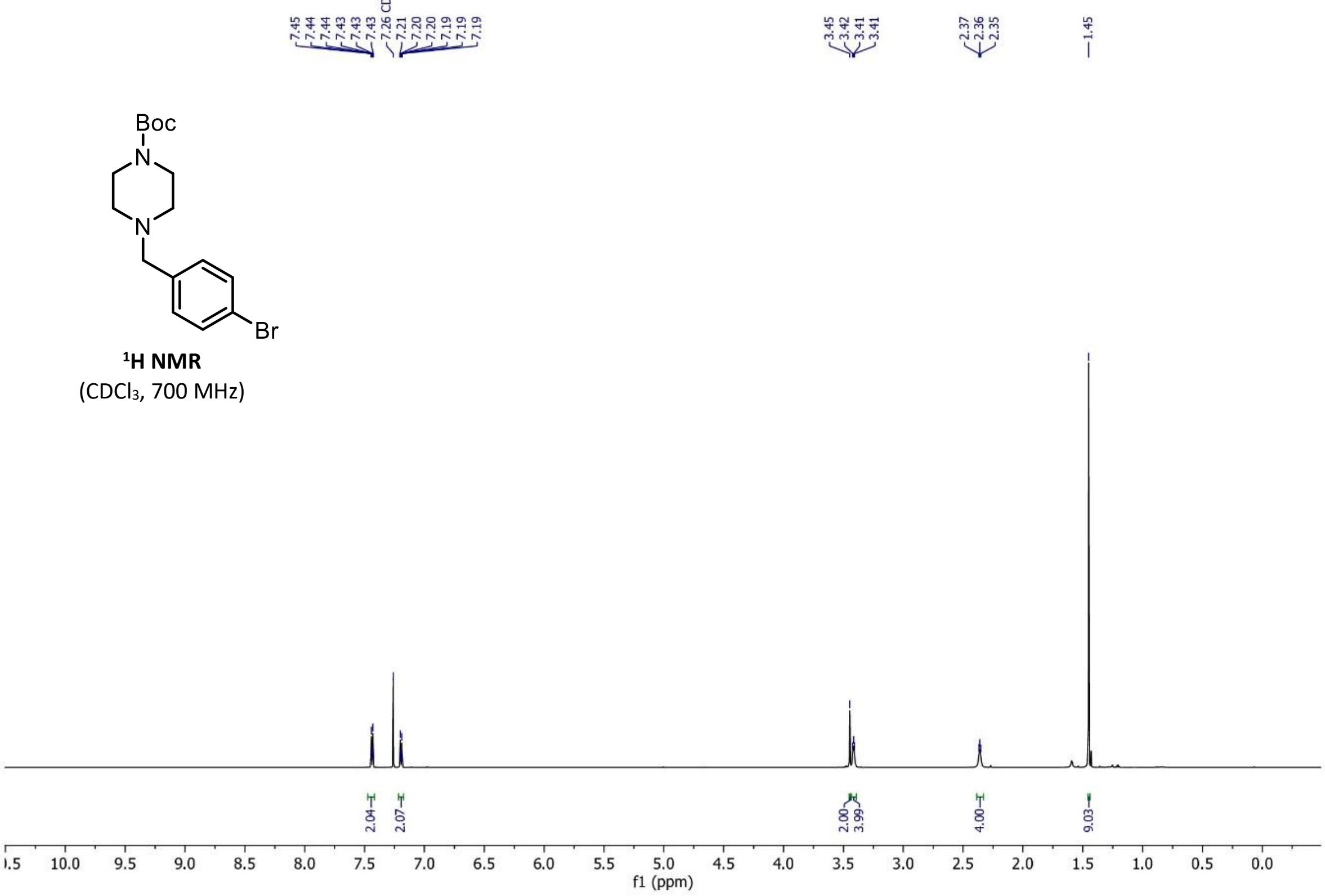

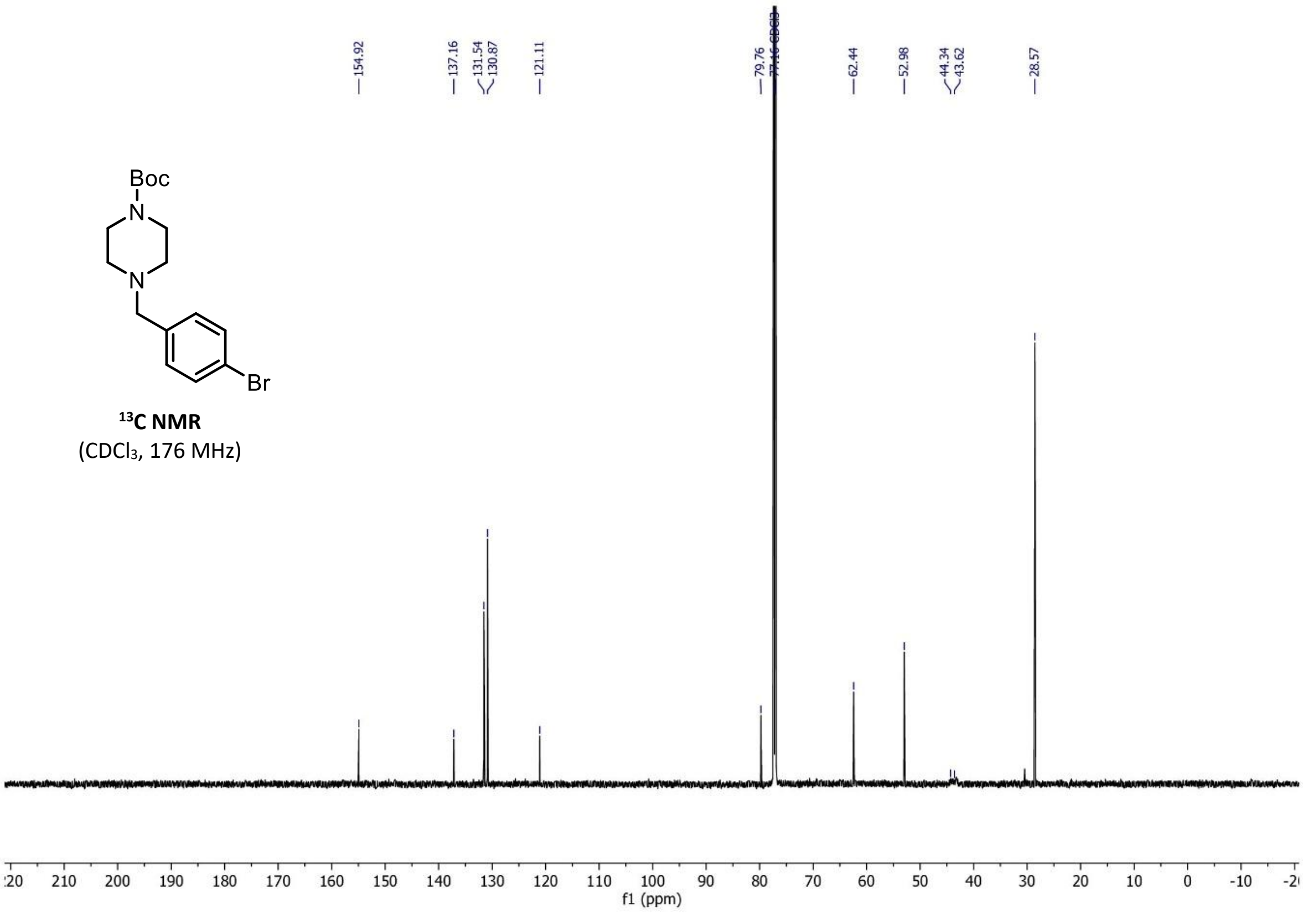

1-(4-Bromobenzyl)-4-phenylpiperidin-4-ol (3j)

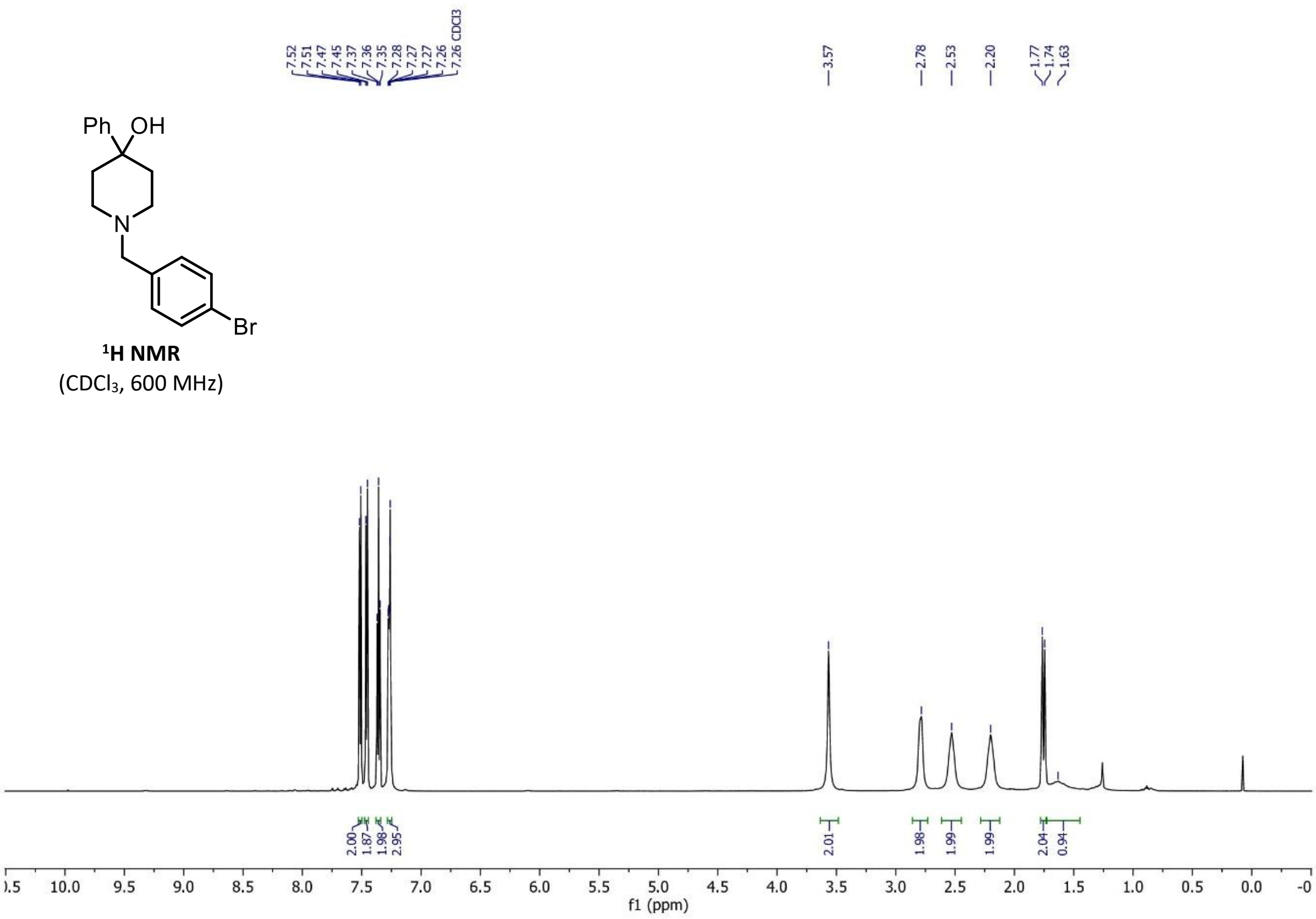

^{1}H NMR
($CDCl_3$, 600 MHz)

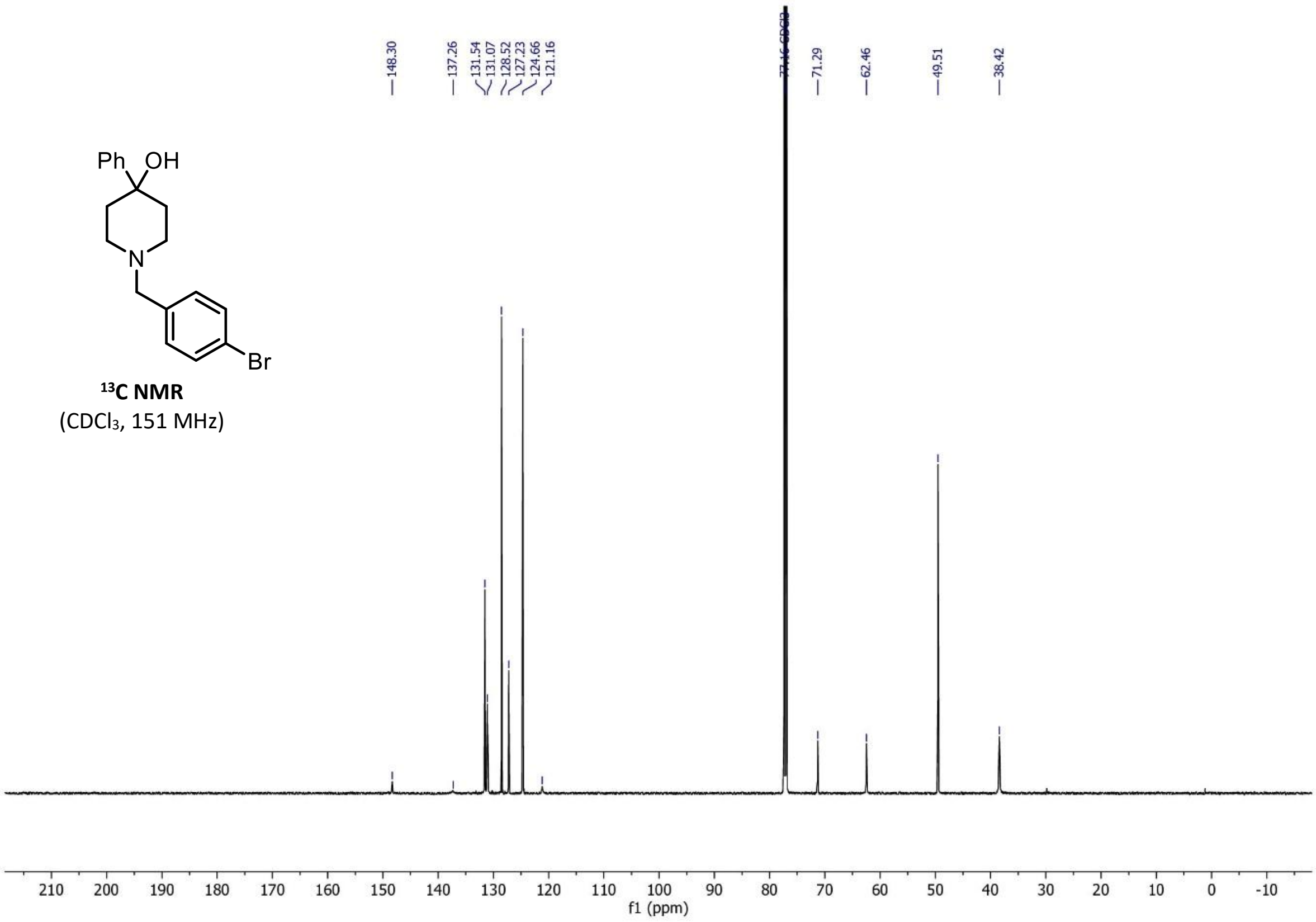

^{13}C NMR
($CDCl_3$, 151 MHz)

(3*S*,4*R*)-3-((Benzo[*d*][1,3]dioxol-5-yloxy)methyl)-1-(4-bromobenzyl)-4-(4-fluorophenyl)piperidine (3k)

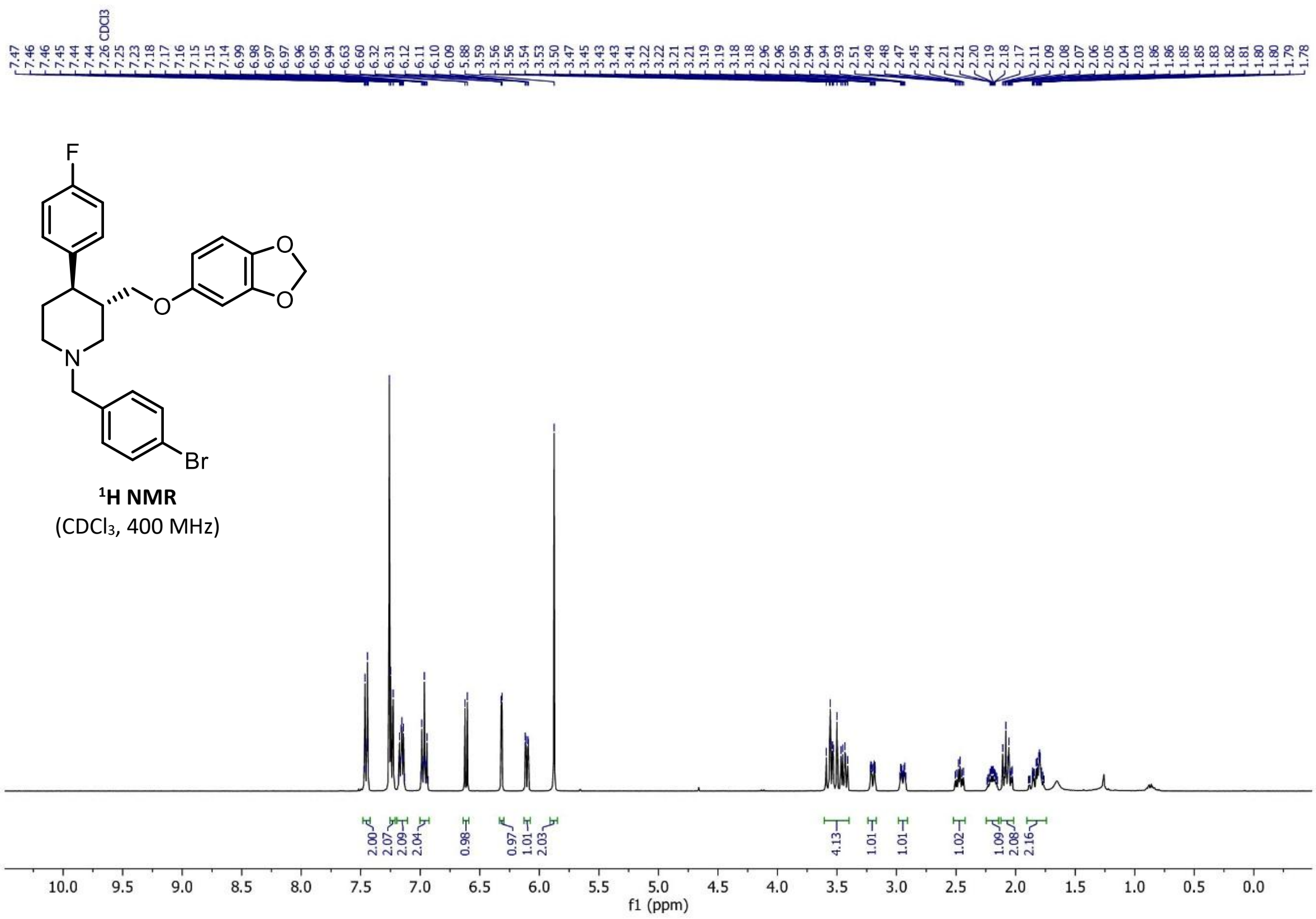

^{1}H NMR
($CDCl_3$, 400 MHz)

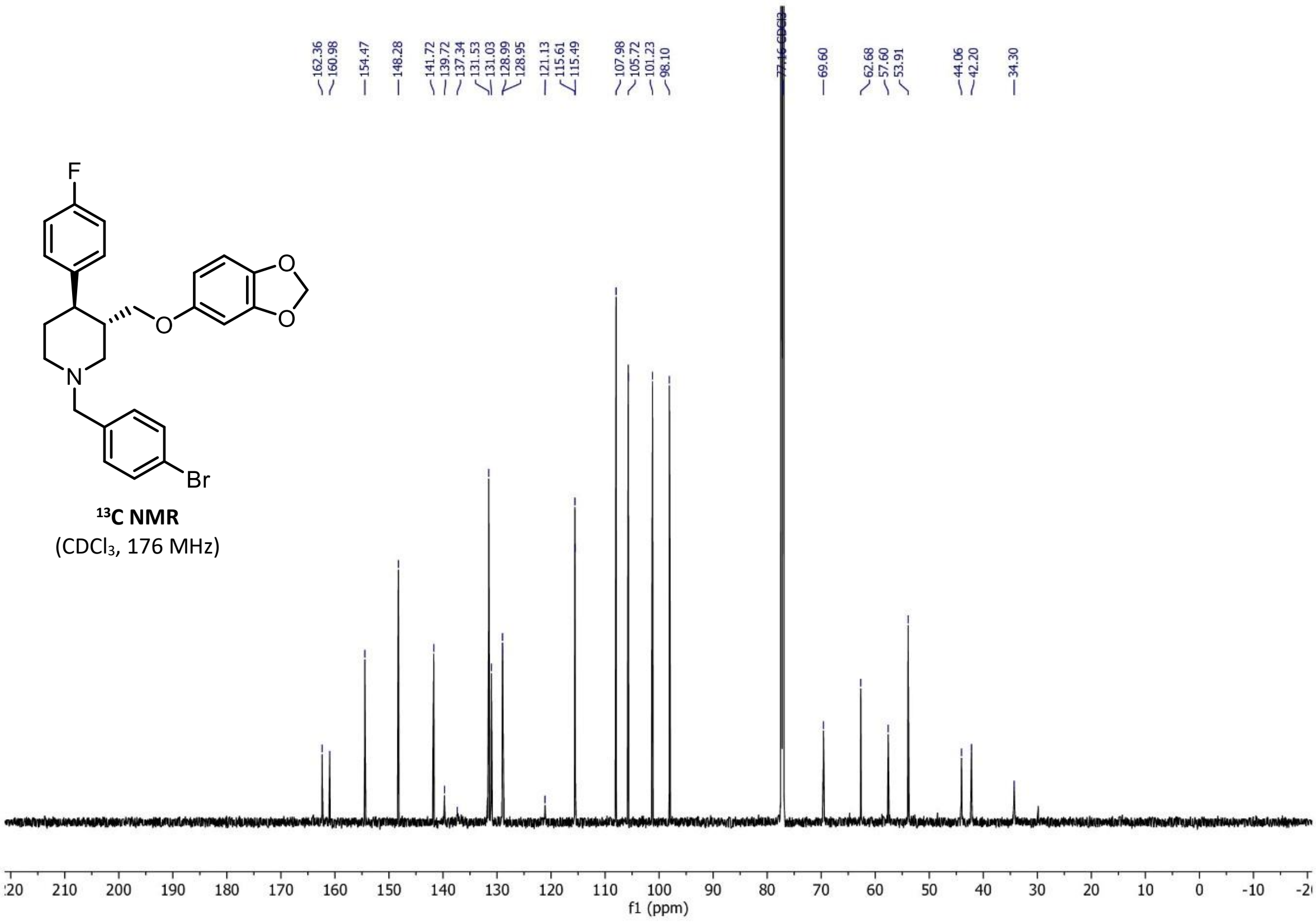

^{13}C NMR
($CDCl_3$, 176 MHz)

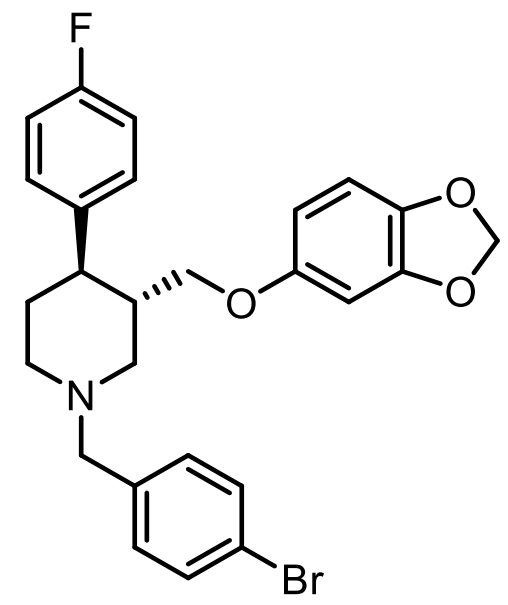

^{19}F NMR
($CDCl_3$, 659 MHz)

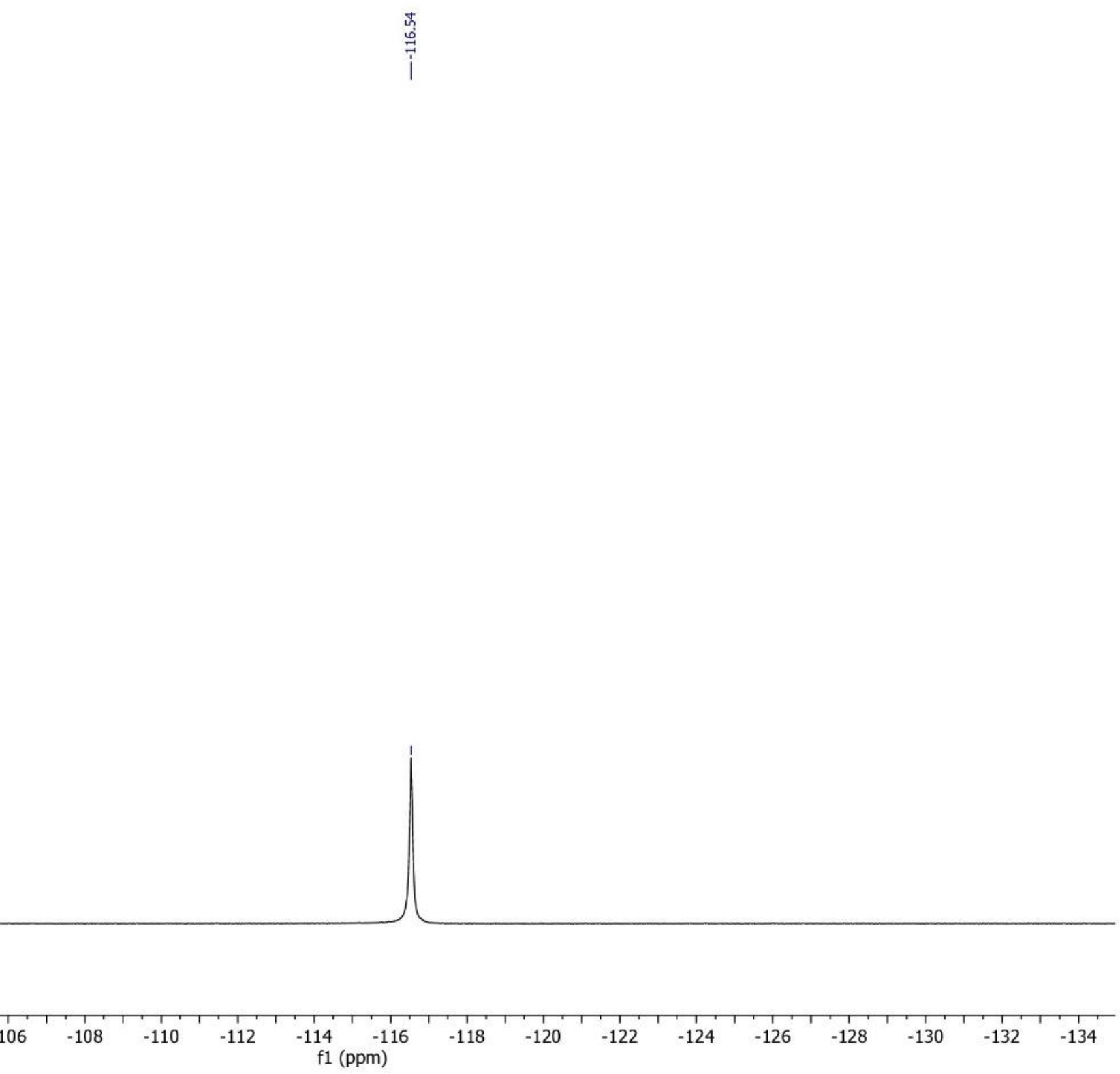

5-((4-(4-Bromobenzyl)-1,4-diazepan-1-yl)sulfonyl)isoquinoline (3l)

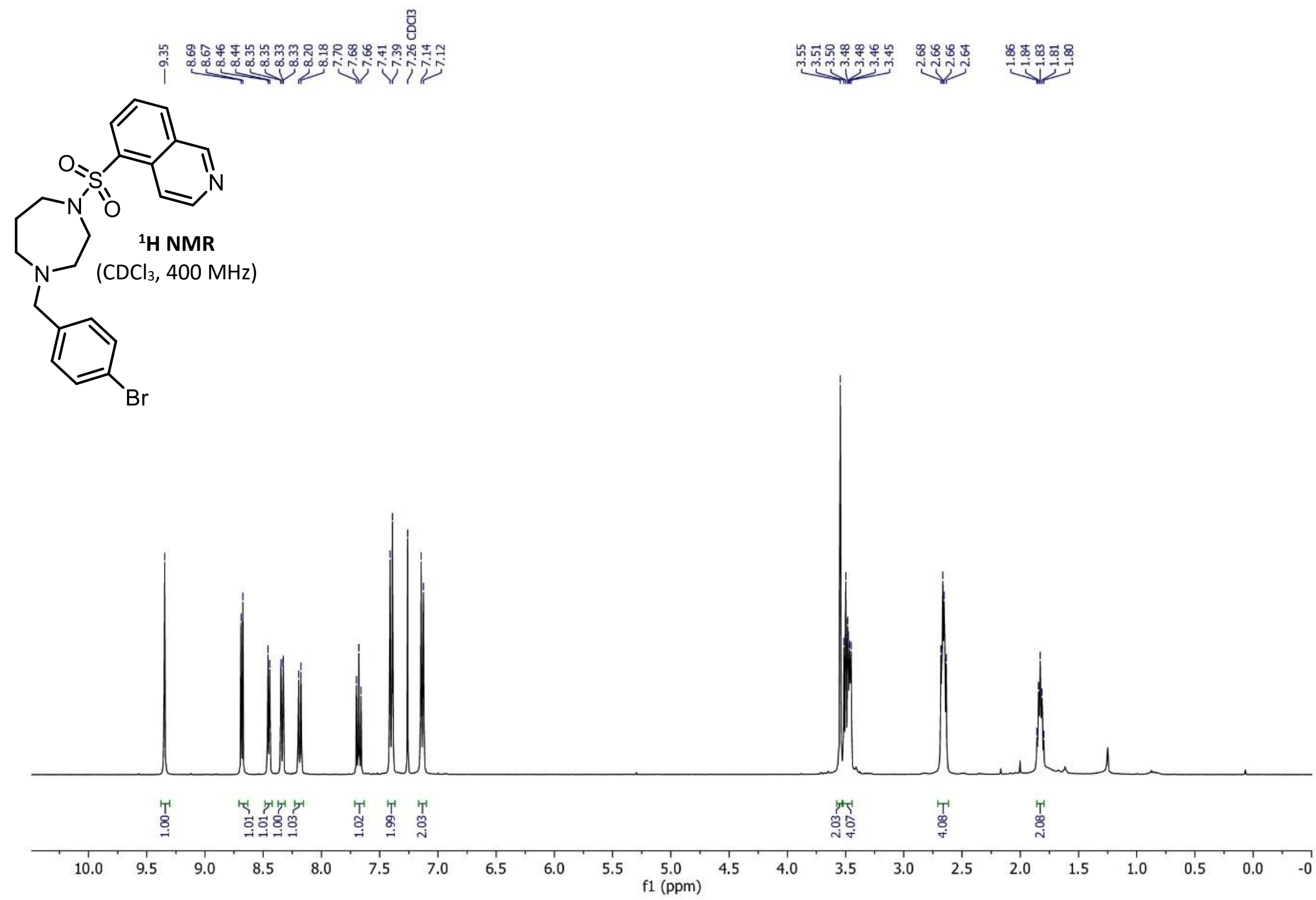

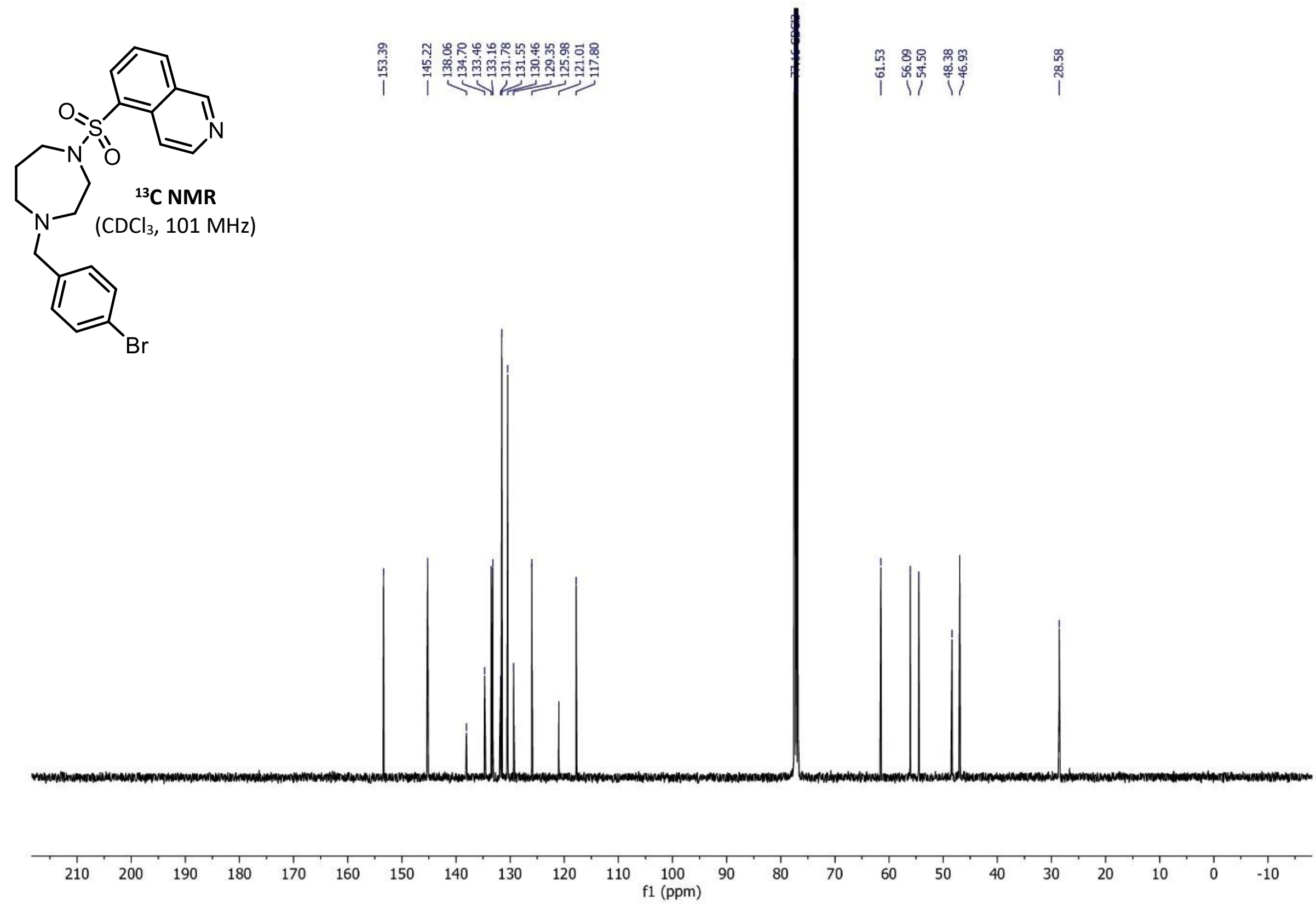

11-(1-(4-Bromobenzyl)piperidin-4-ylidene)-8-chloro-6,11-dihydro-5*H*-benzo[5,6]cyclohepta[1,2-*b*]pyridine (3m)

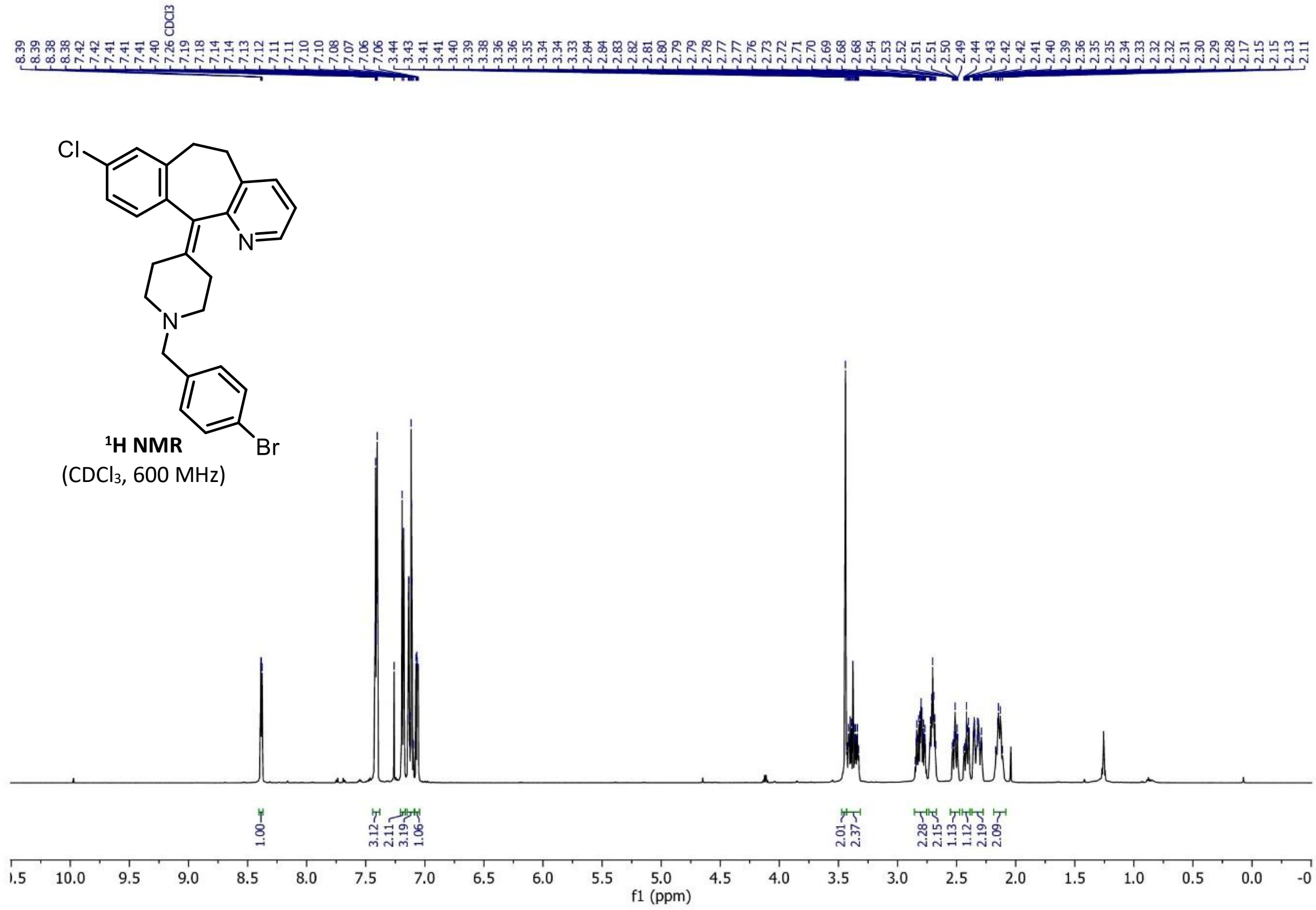

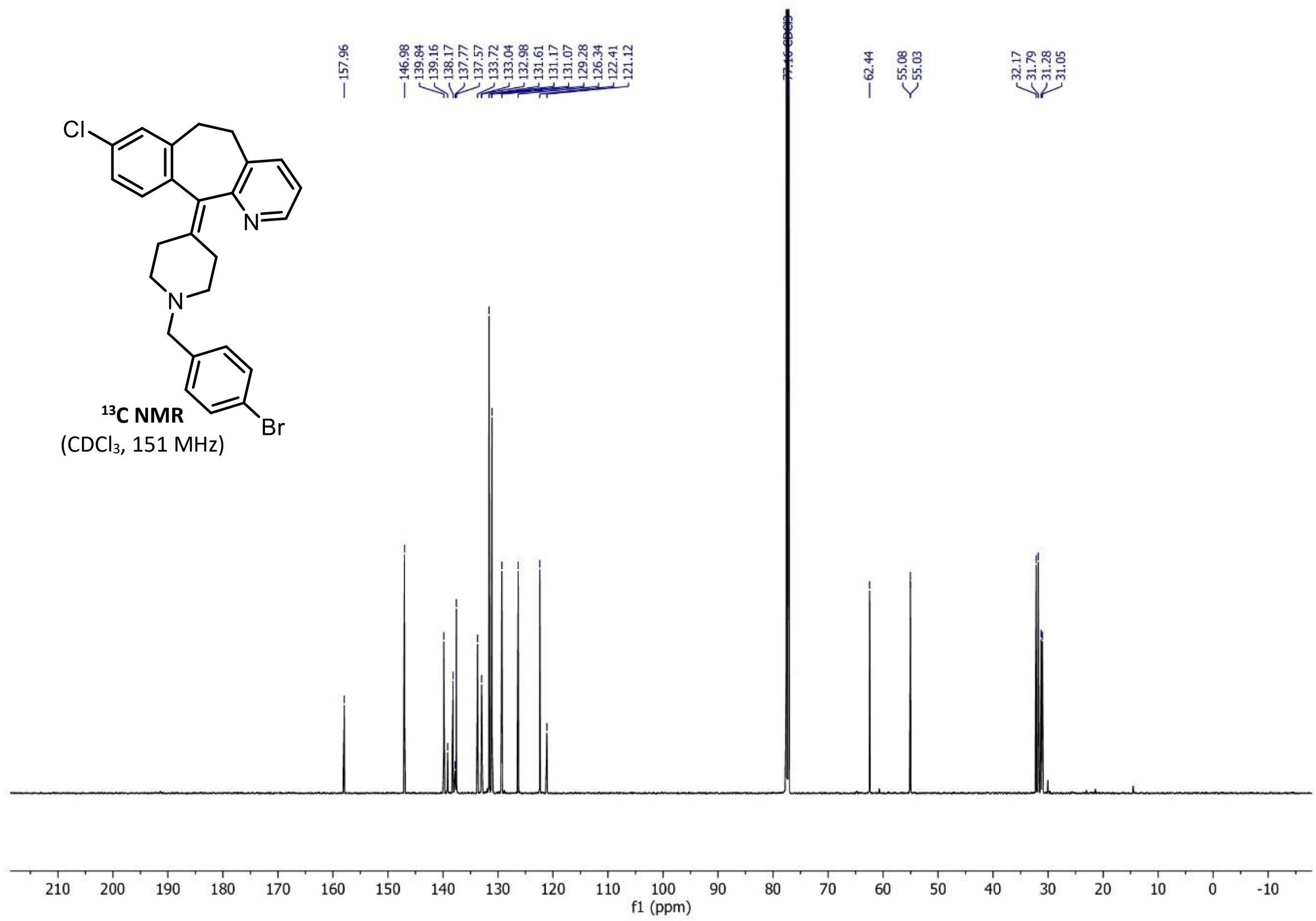

***N*-(4-Bromobenzyl)-*N*-methyl-1-(5-phenyl-1-(pyridin-3-ylsulfonyl)-1*H*-pyrrol-3-yl)methanamine (3n)**

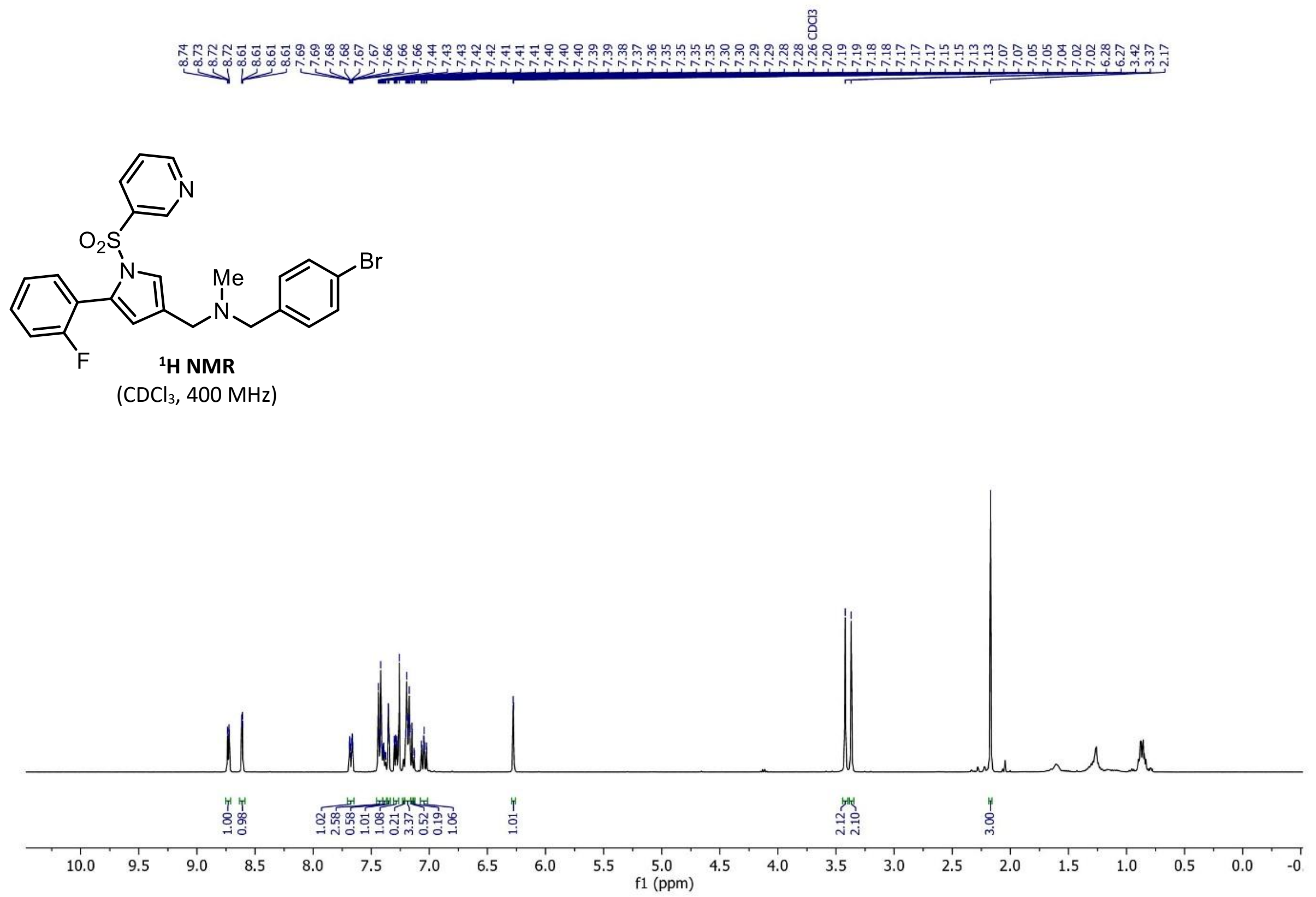

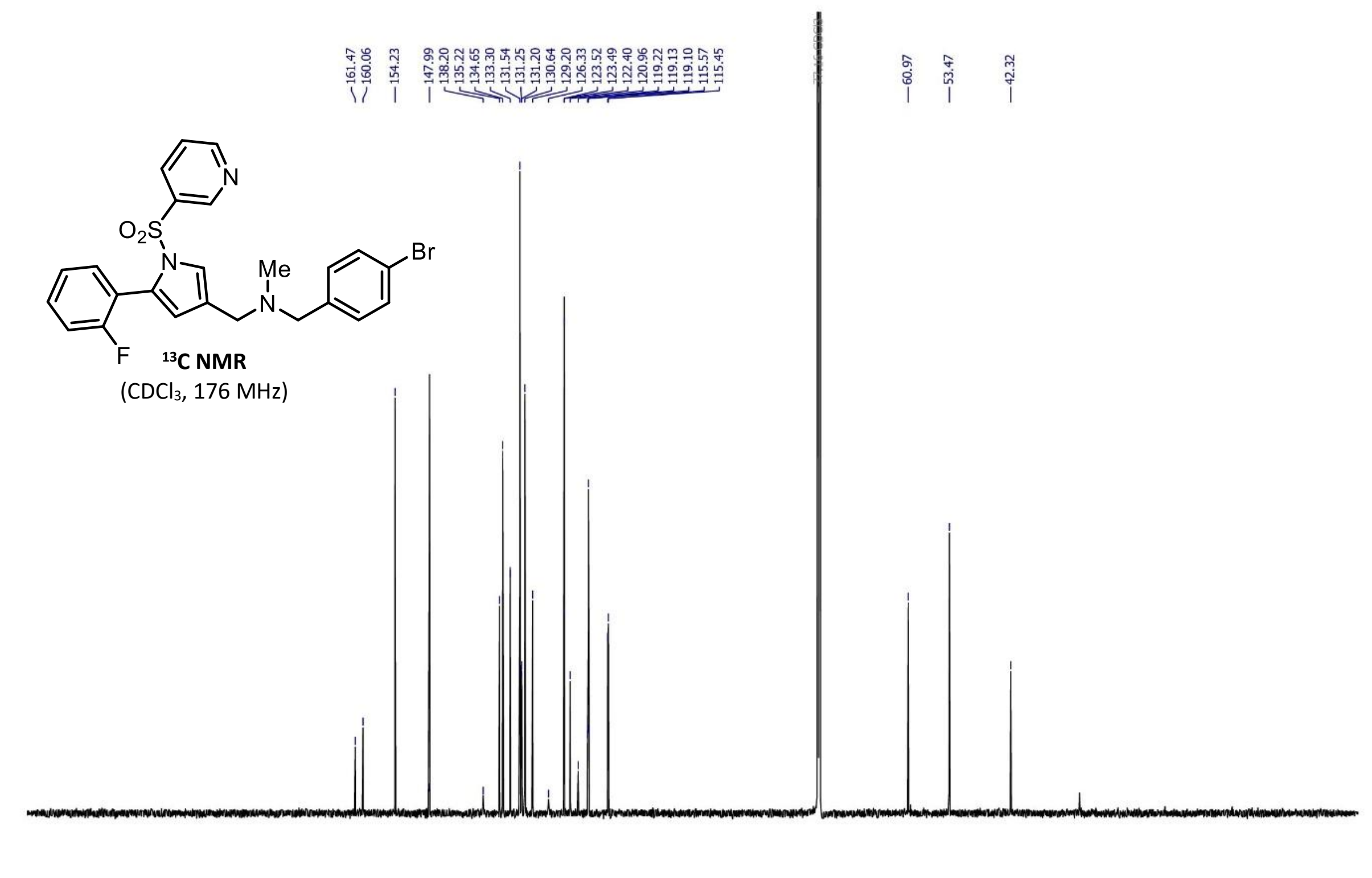

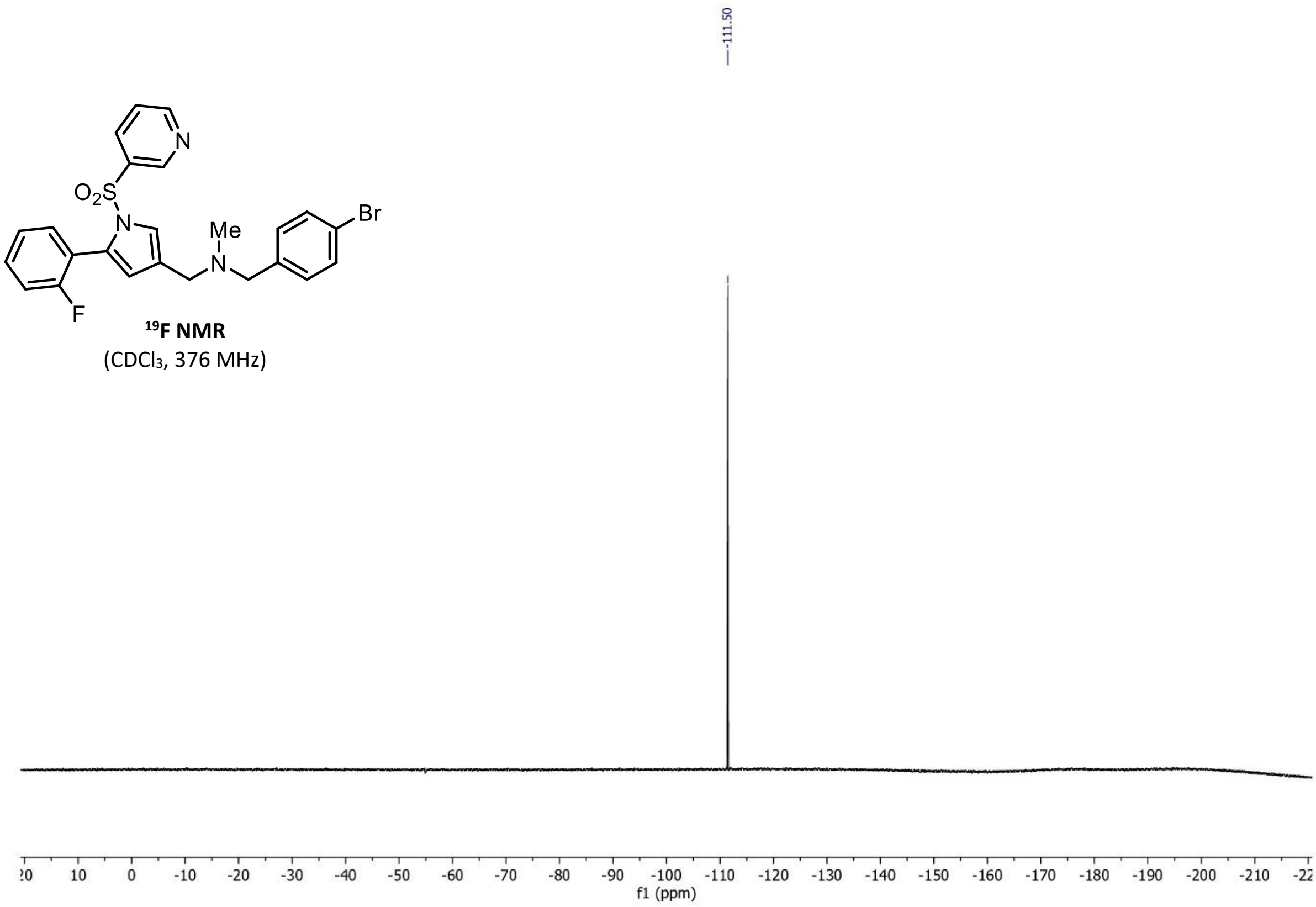

^{19}F NMR
($CDCl_3$, 376 MHz)

1-((2,4,6-Tris(trifluoromethyl)phenyl)sulfonyl)piperidin-4-one (S26)

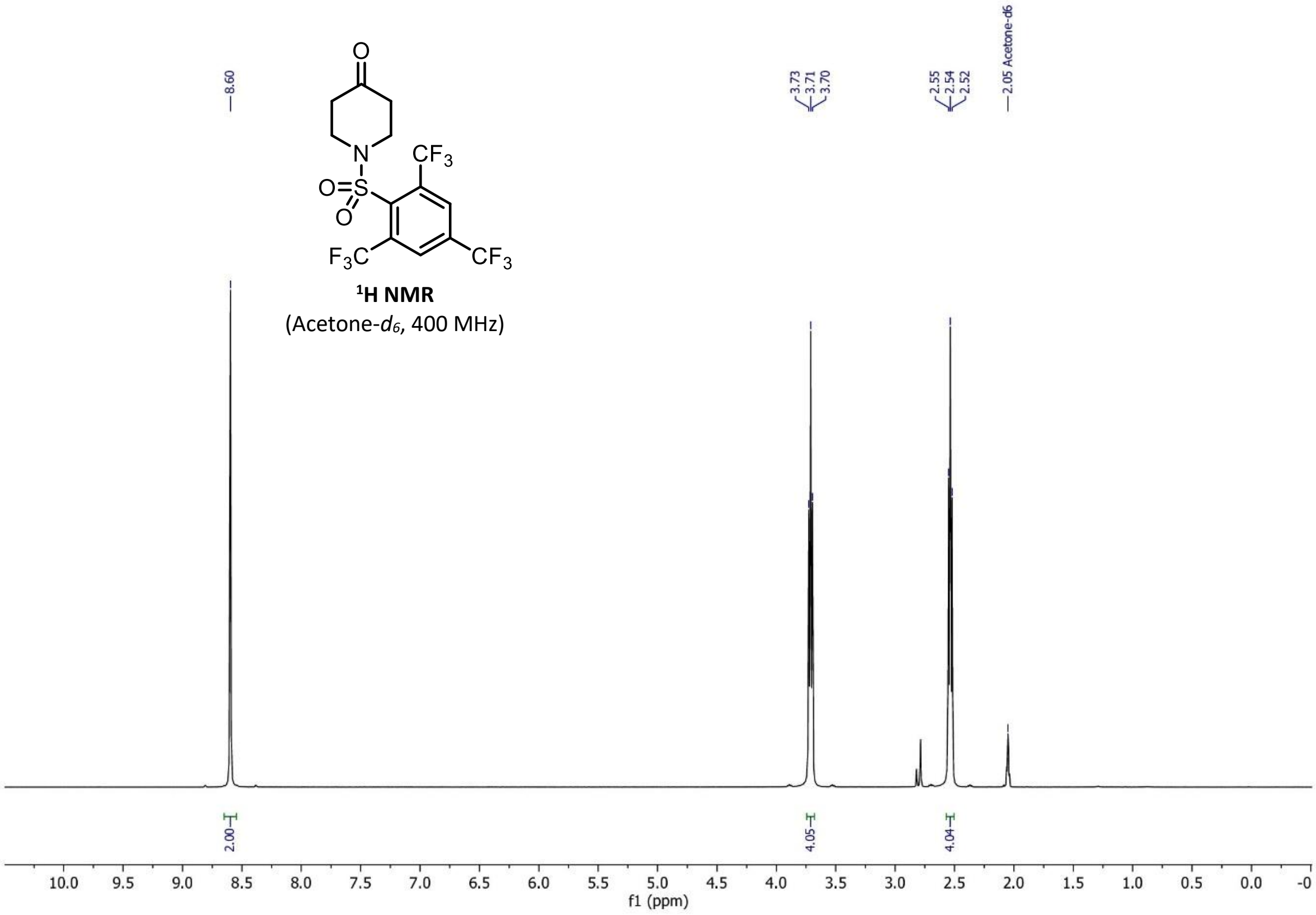

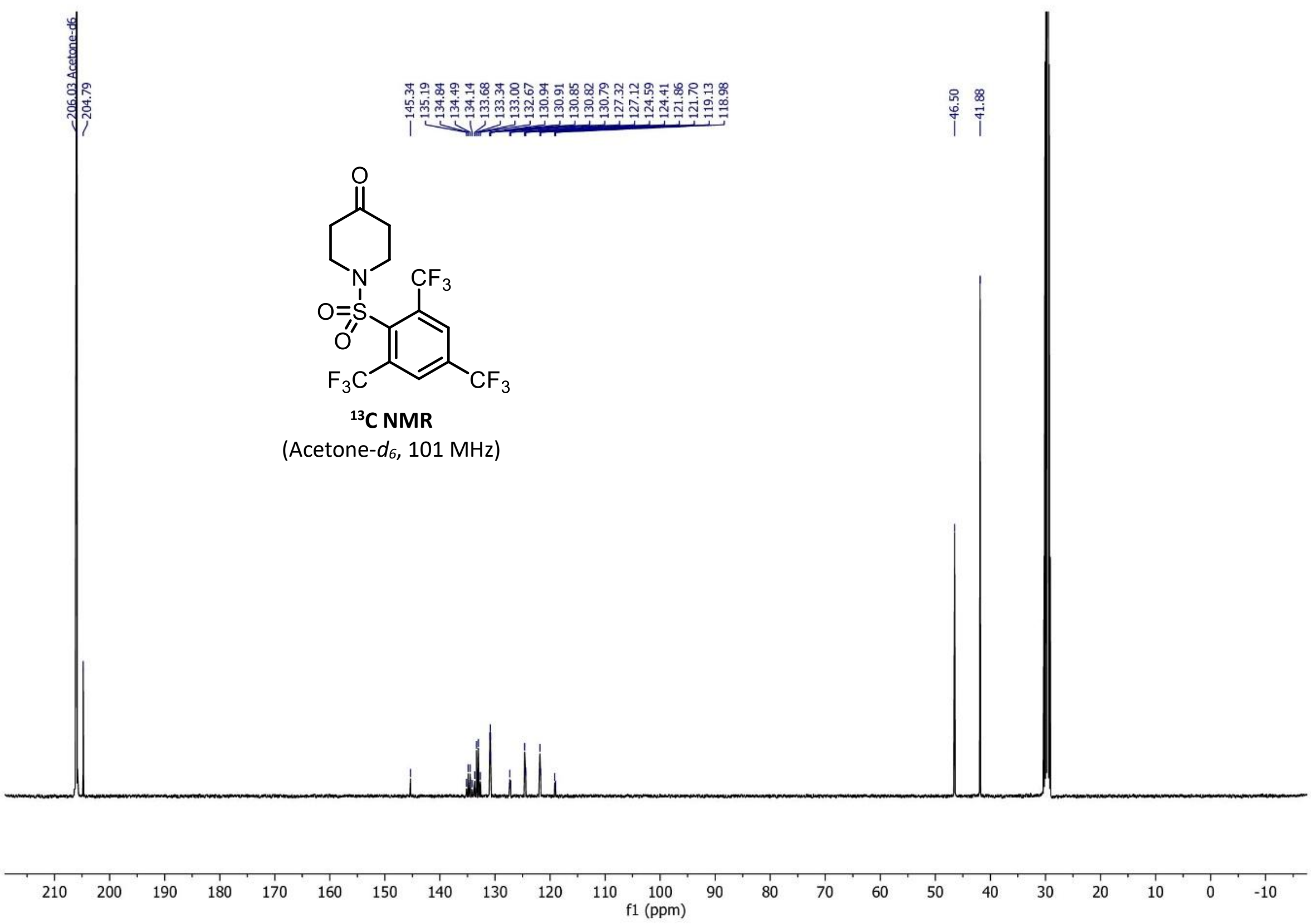

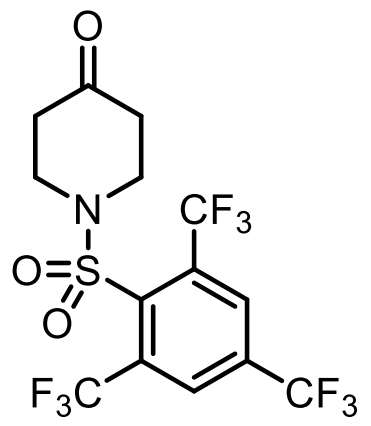

^{19}F NMR
(Acetone-d_6, 376 MHz)

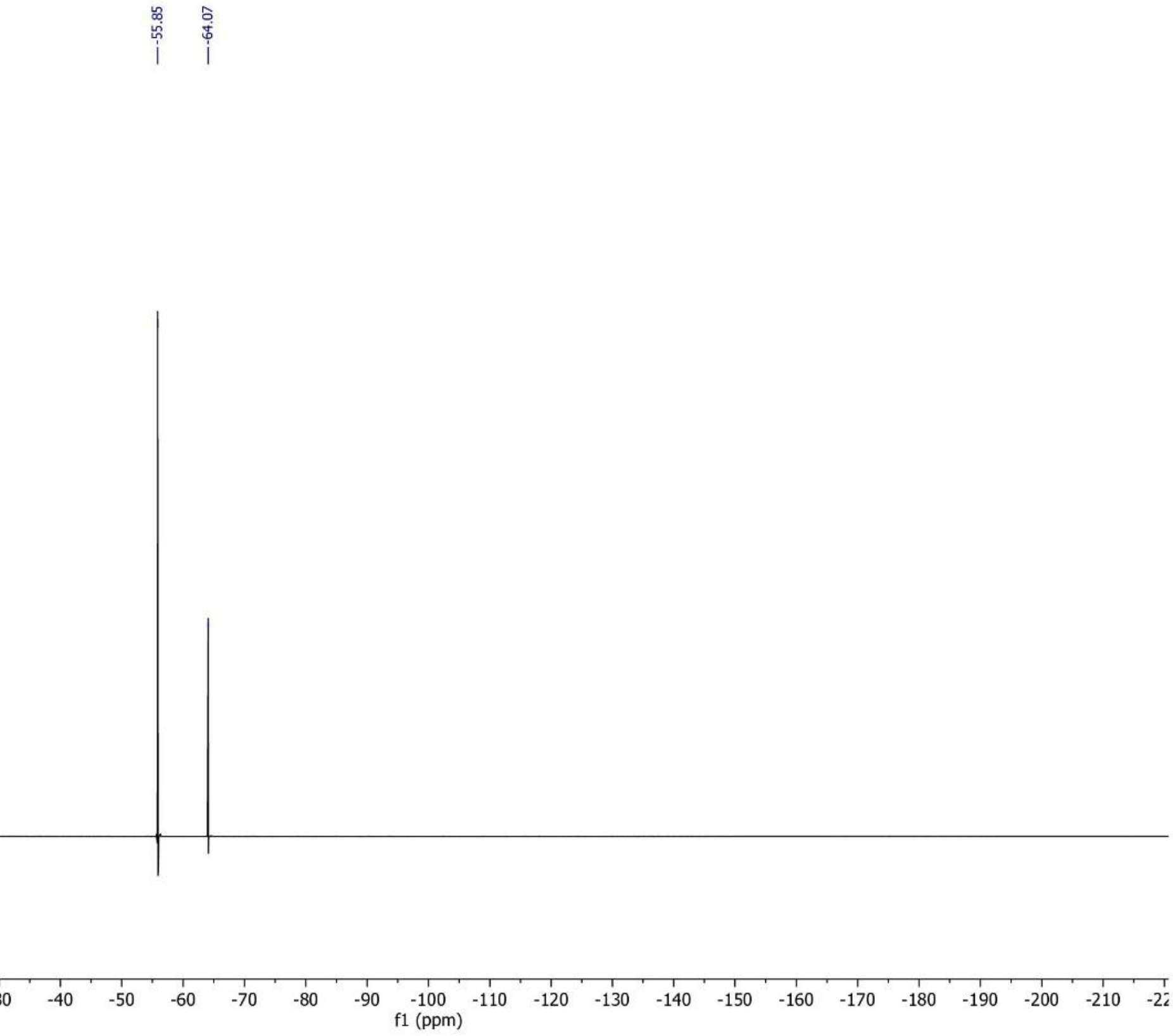

4-(4-Chloro-3-(trifluoromethyl)phenyl)-1-((2,4,6-tris(trifluoromethyl)phenyl)sulfonyl)piperidin-4-ol (5)

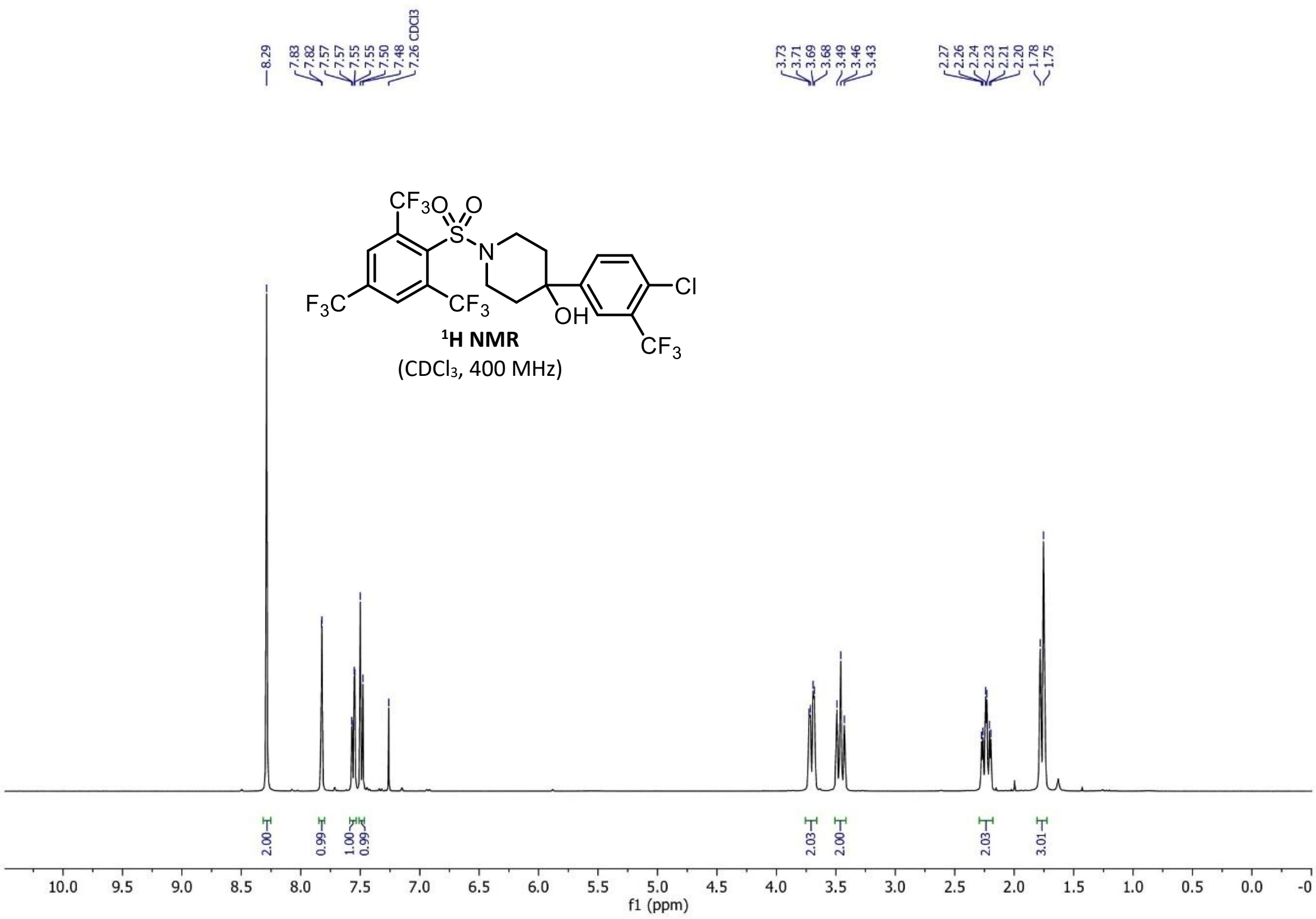

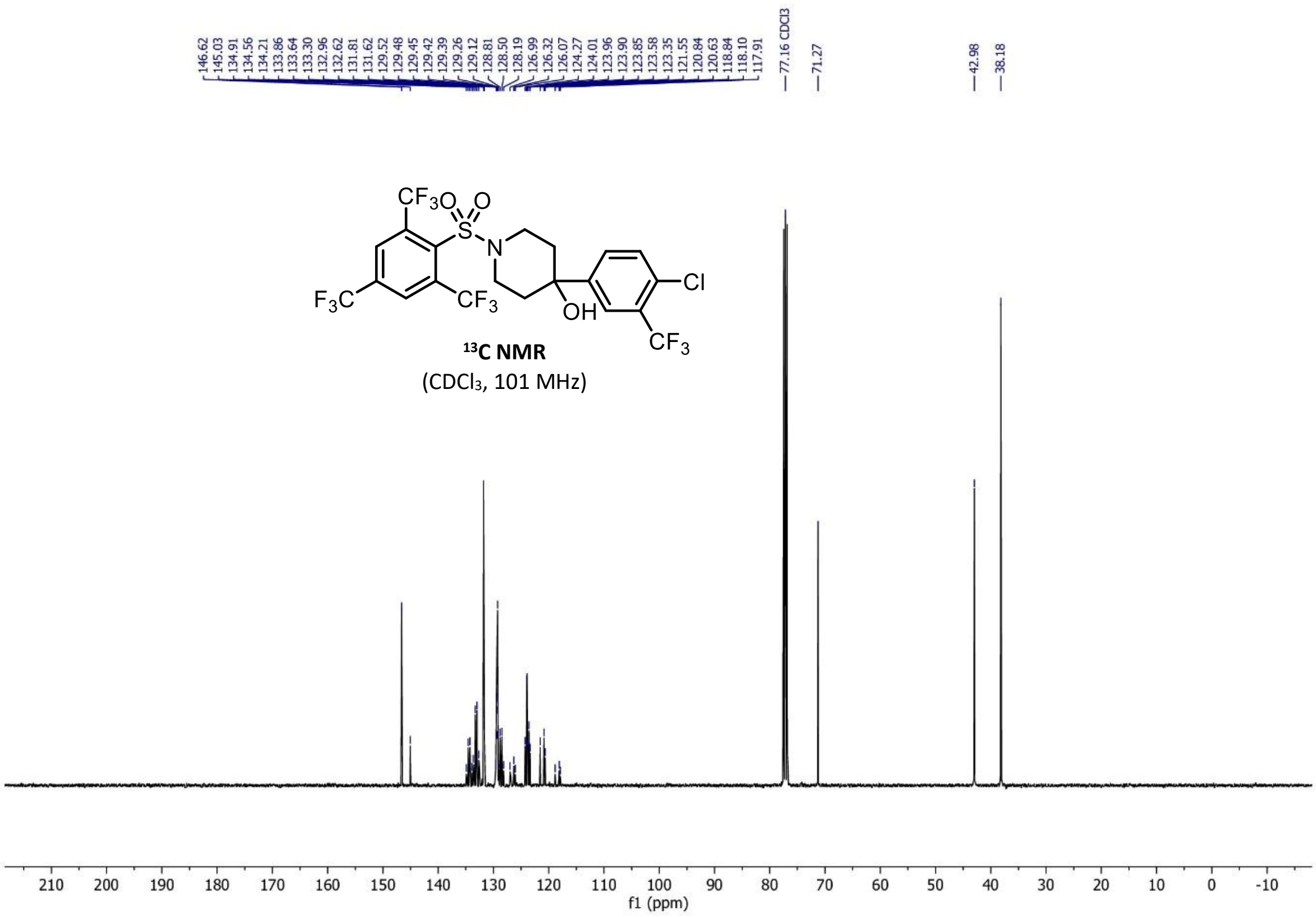

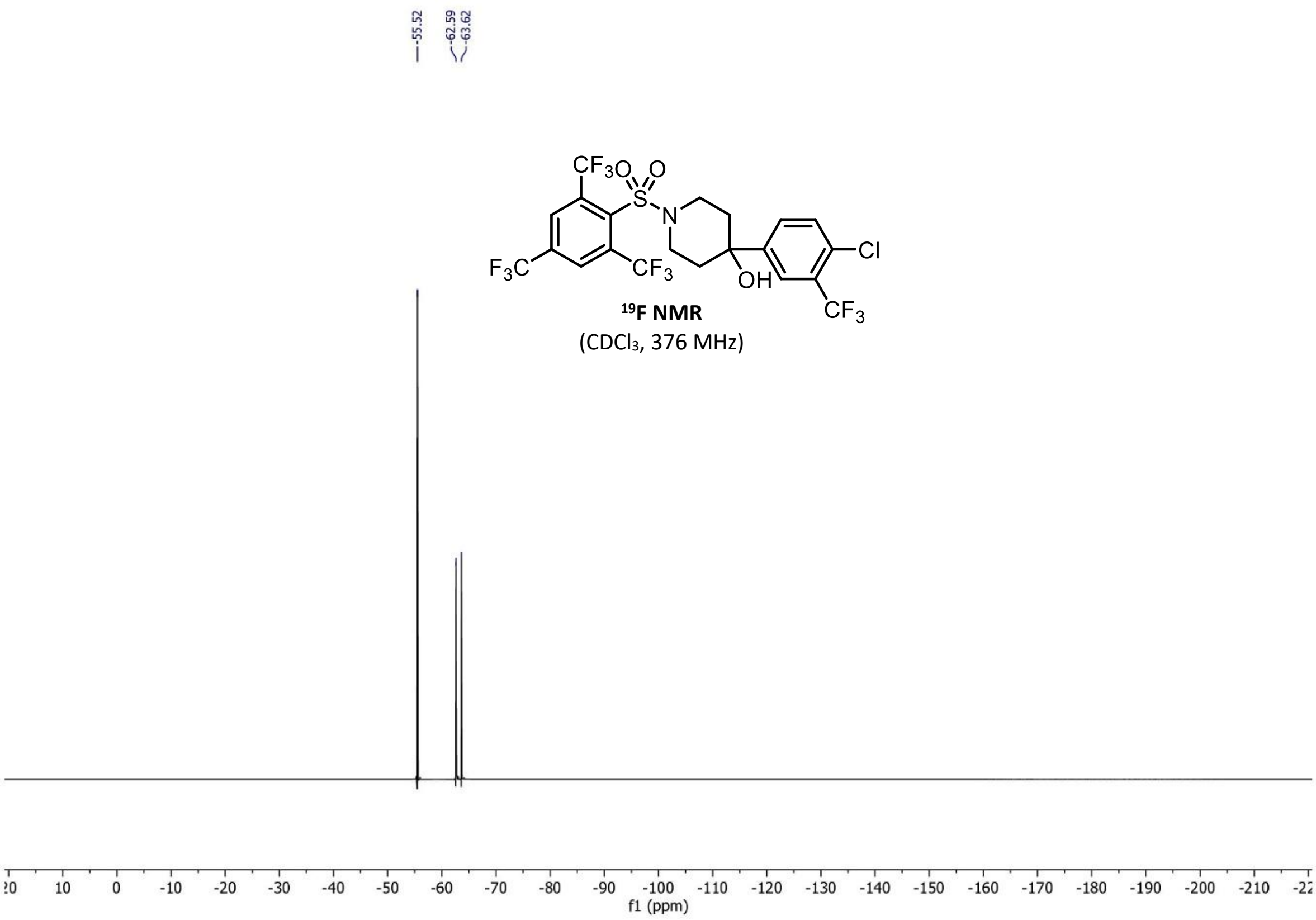

-55.52
-62.59
-63.62
CF3
O
O
S
N
Cl
F3C
CF3
OH
CF3
¹⁹F NMR
(CDCl3, 376 MHz)
f1 (ppm)

1-(4,4-Bis(4-fluorophenyl)butyl)-4-(4-chloro-3-(trifluoromethyl)phenyl)piperidin-4-ol (6-*Penfluridol*)

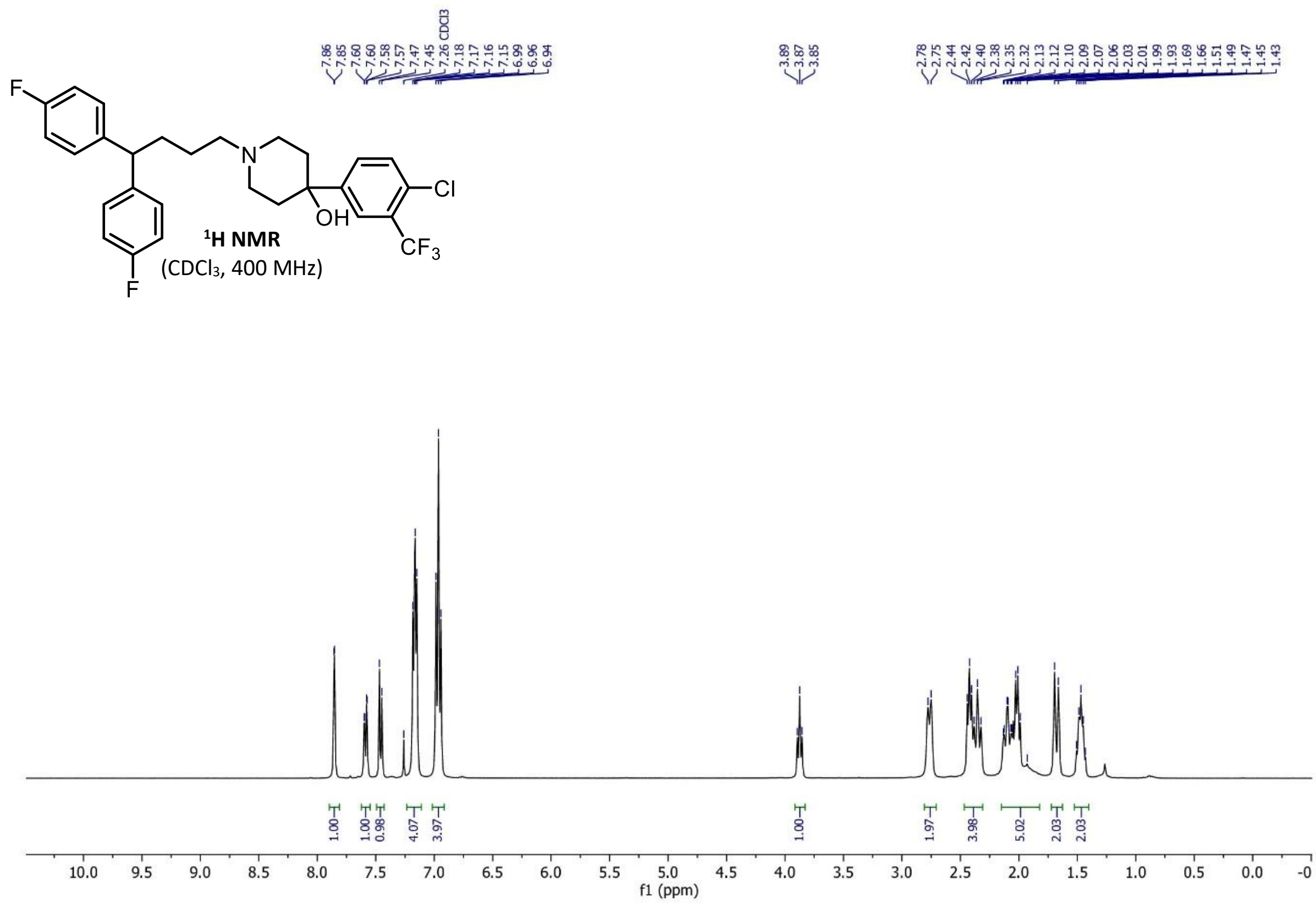

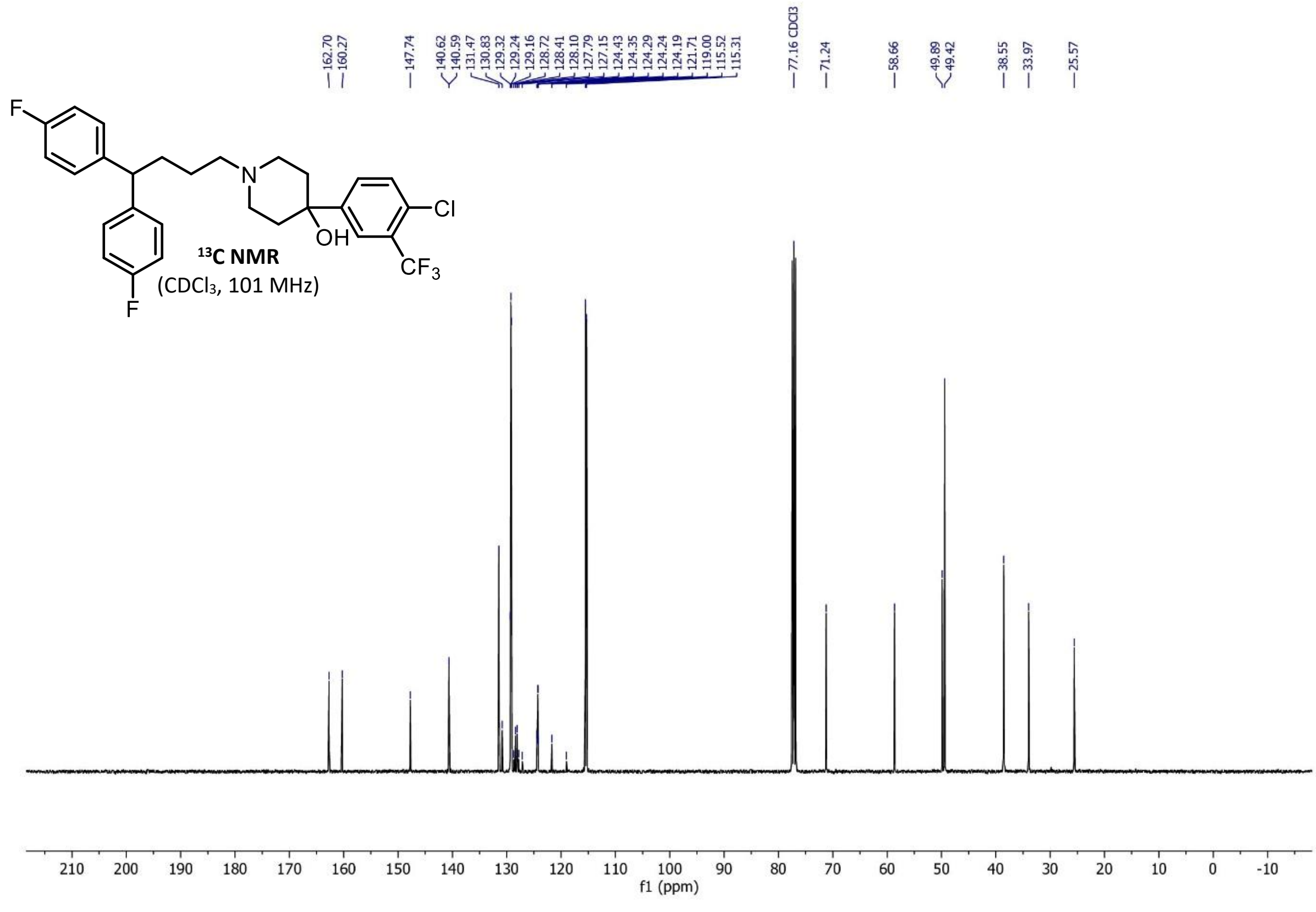

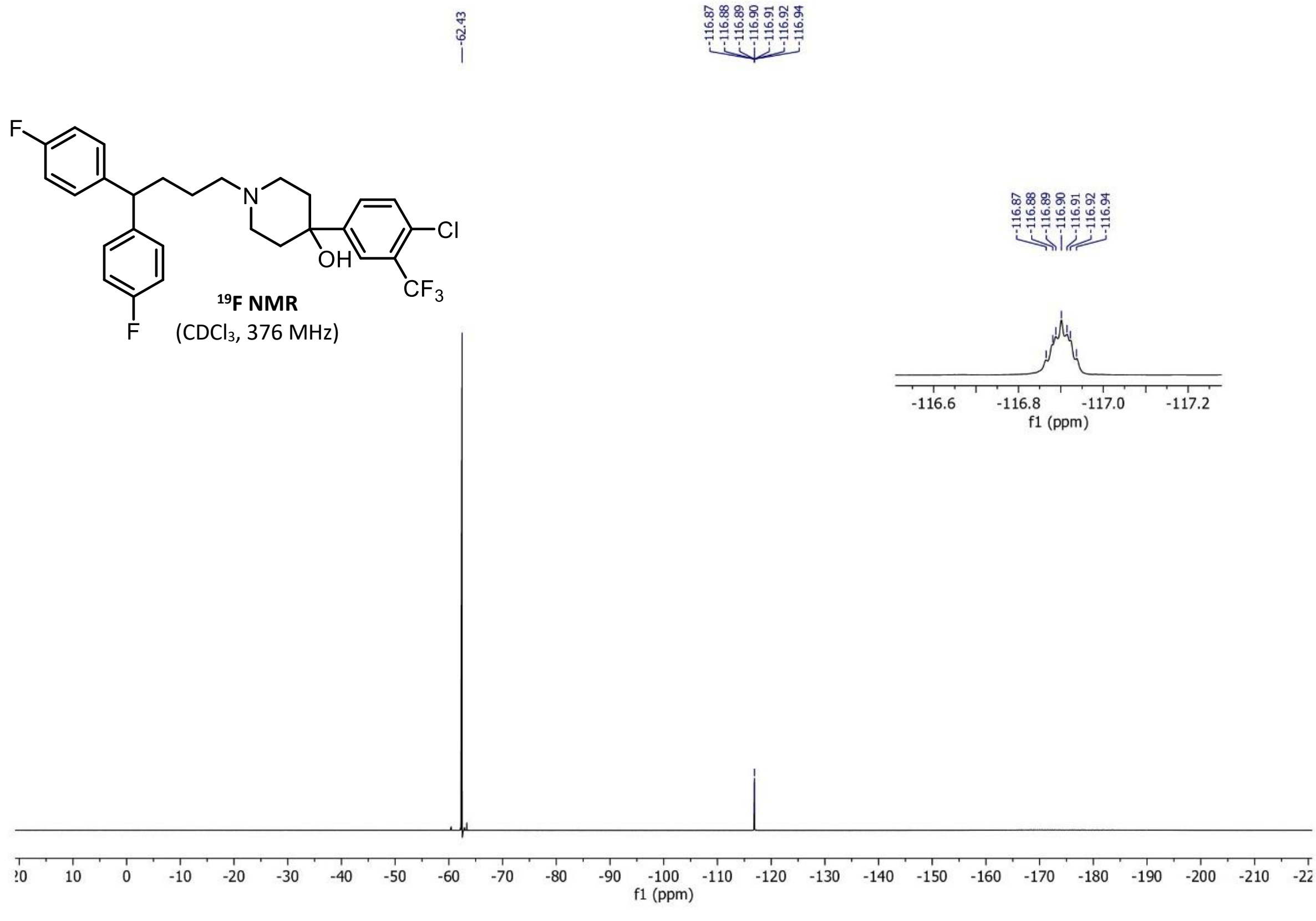
-62.43
-116.87
-116.88
-116.89
-116.90
-116.91
-116.92
-116.94
F
N
OH
Cl
CF3
19F NMR
(CDCl3, 376 MHz)
-116.6
-116.8
-117.0
-117.2
f1 (ppm)
f1 (ppm)

***tert*-Butyl 4-(methyl-d_3)piperazine-1-carboxylate (8)**

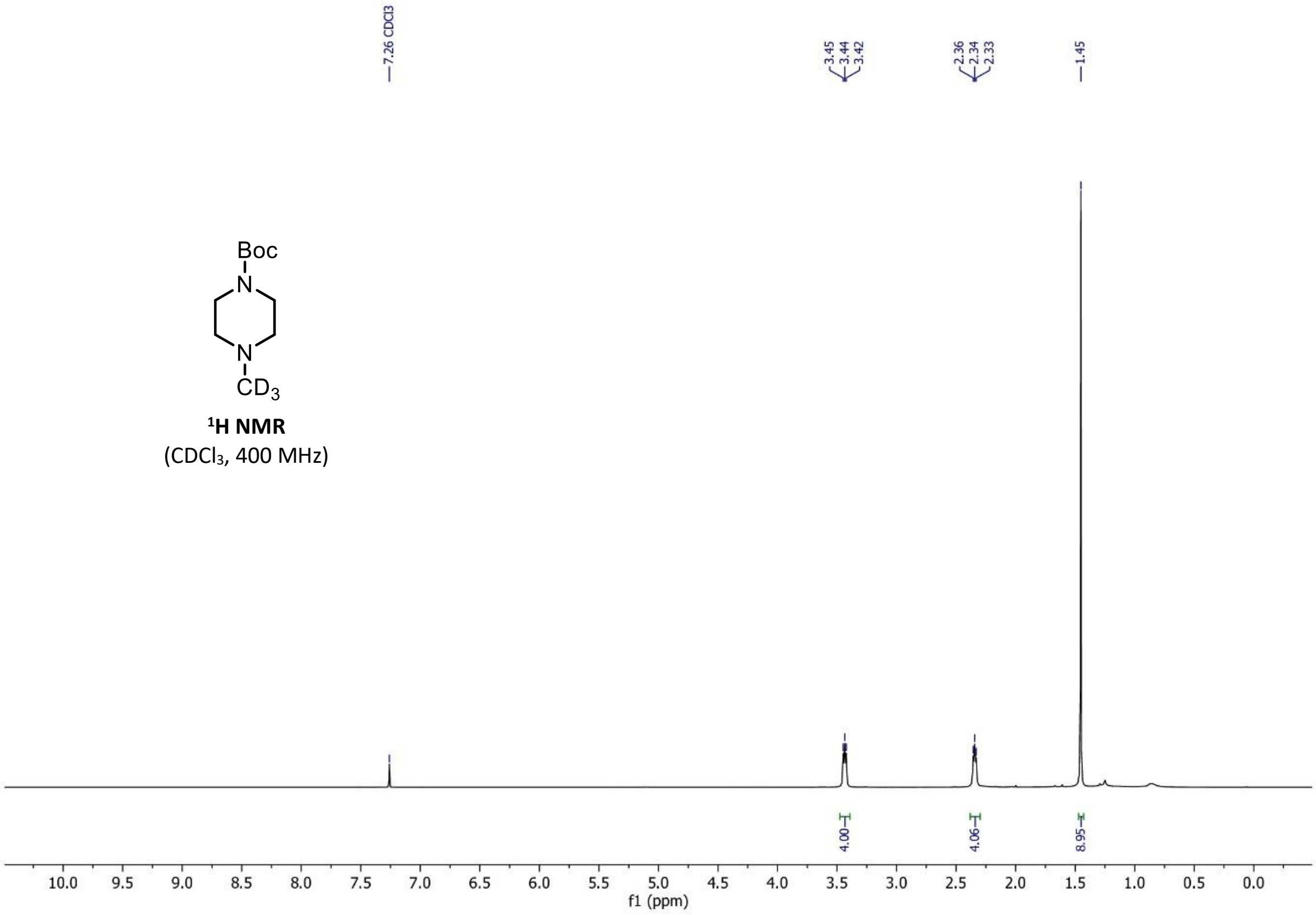

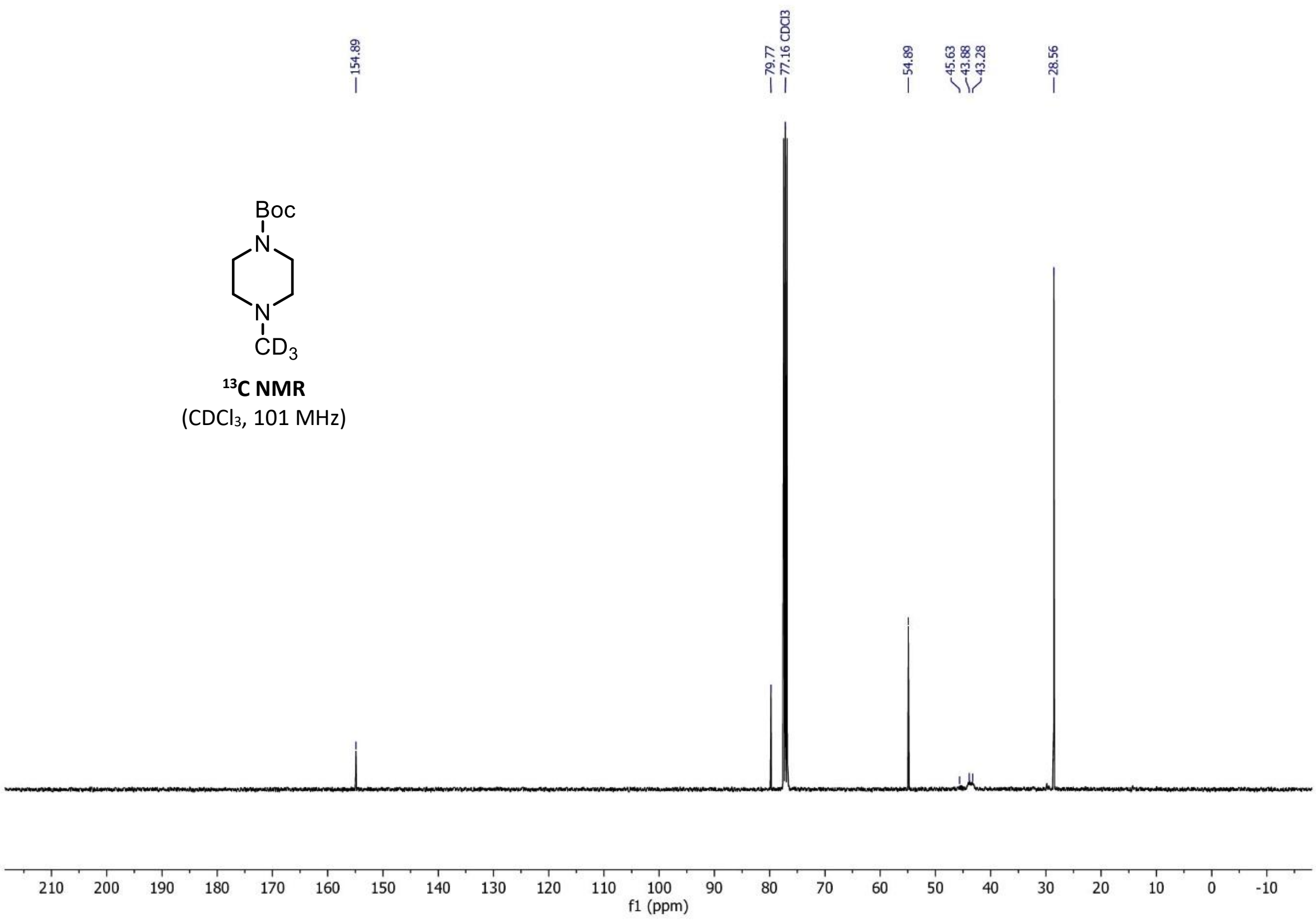

***N*,*N*-Dimethyl-2,4,6-tris(trifluoromethyl)benzenesulfonamide-^{15}*N* (9)**

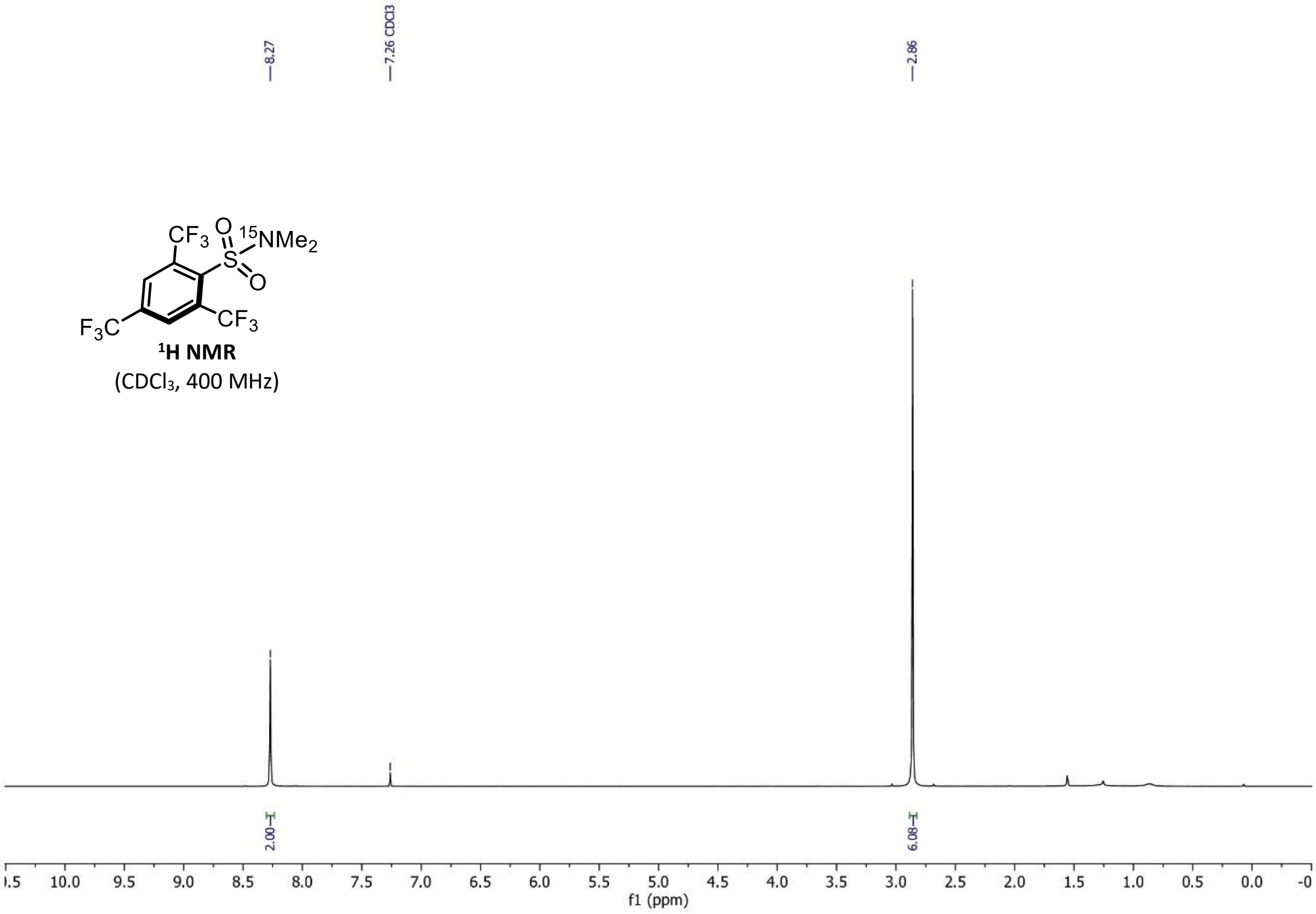

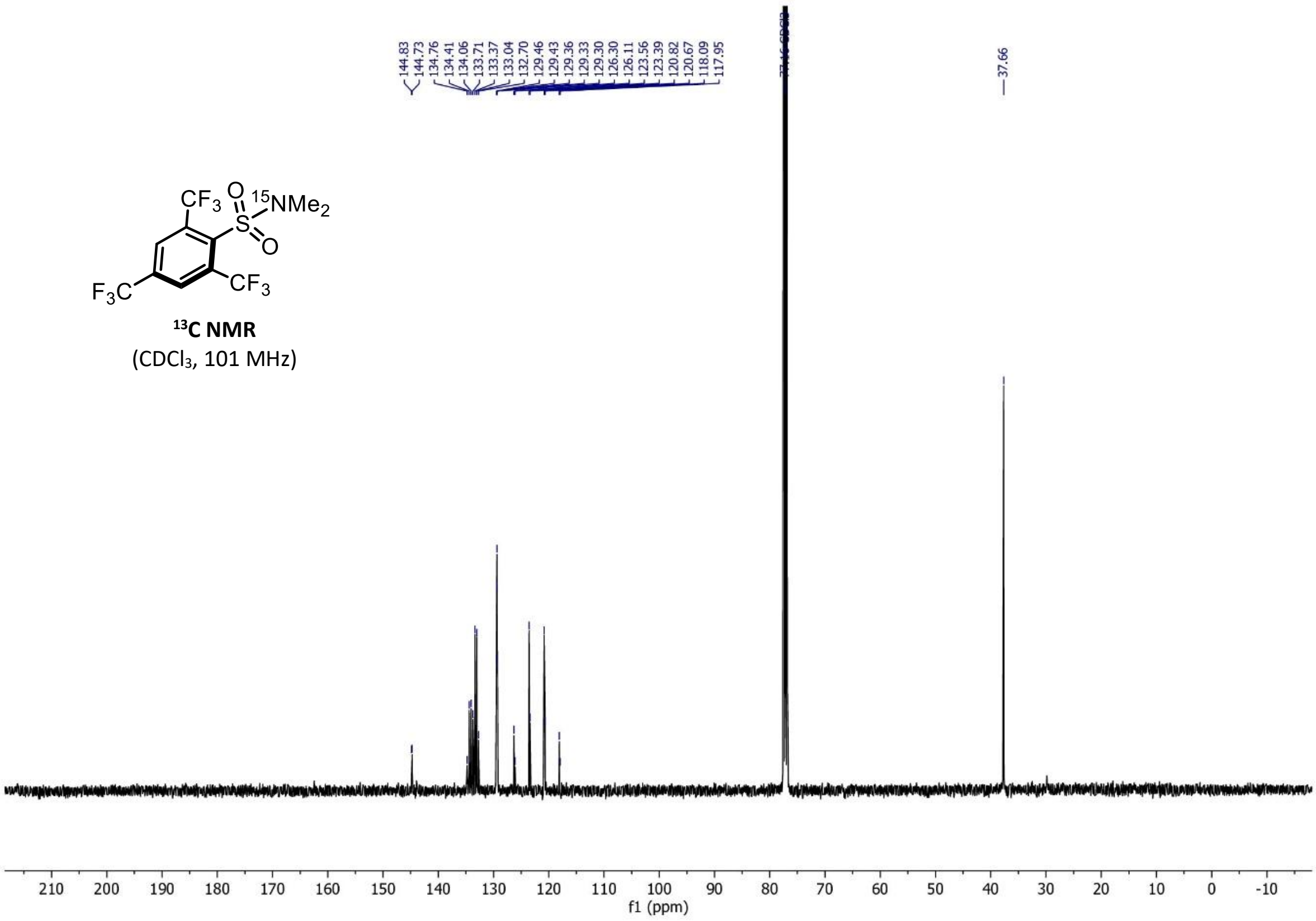

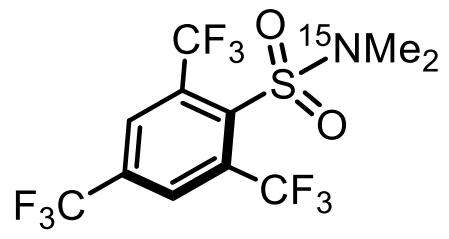

^{19}F NMR
($CDCl_3$, 376 MHz)

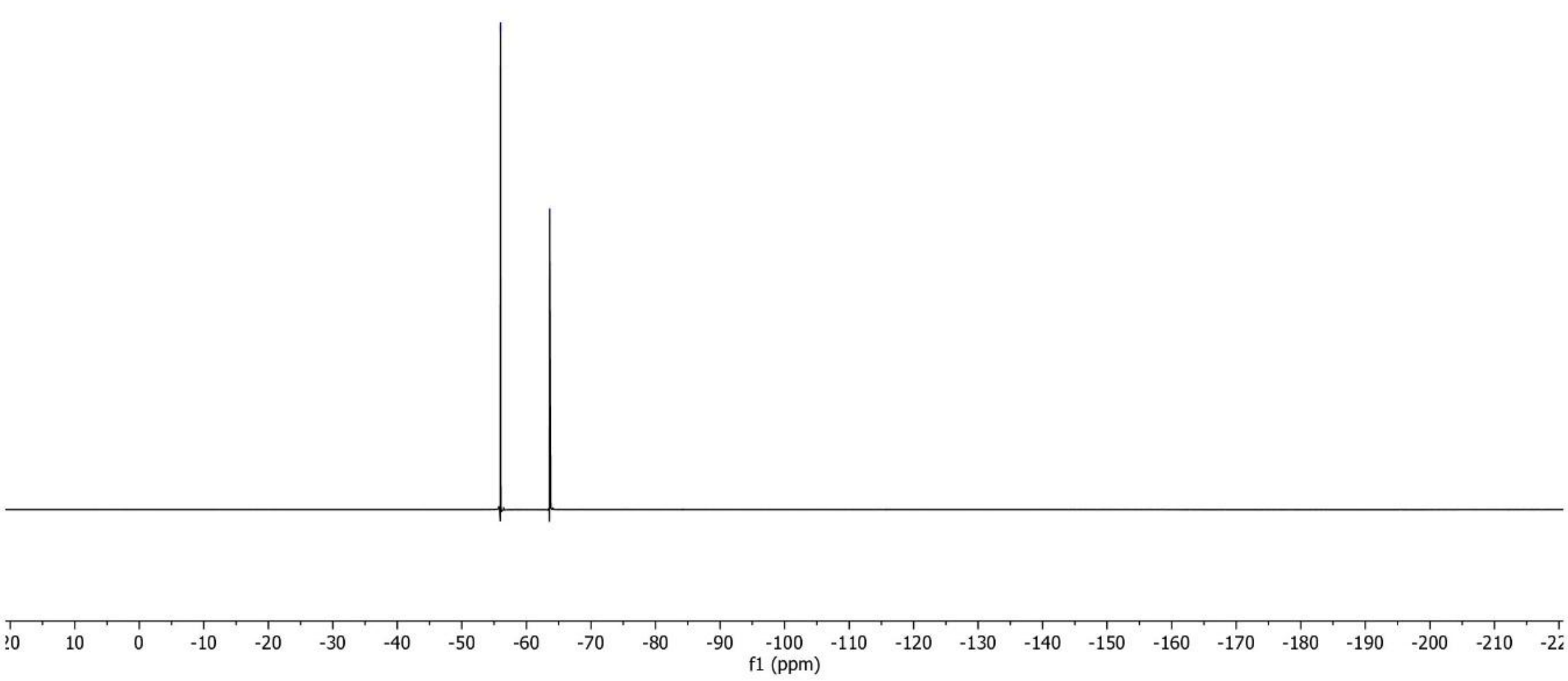

(*S*)-3-(1-(Dimethylamino-^{15}*N*)ethyl)phenyl ethyl(methyl)carbamate (10a)

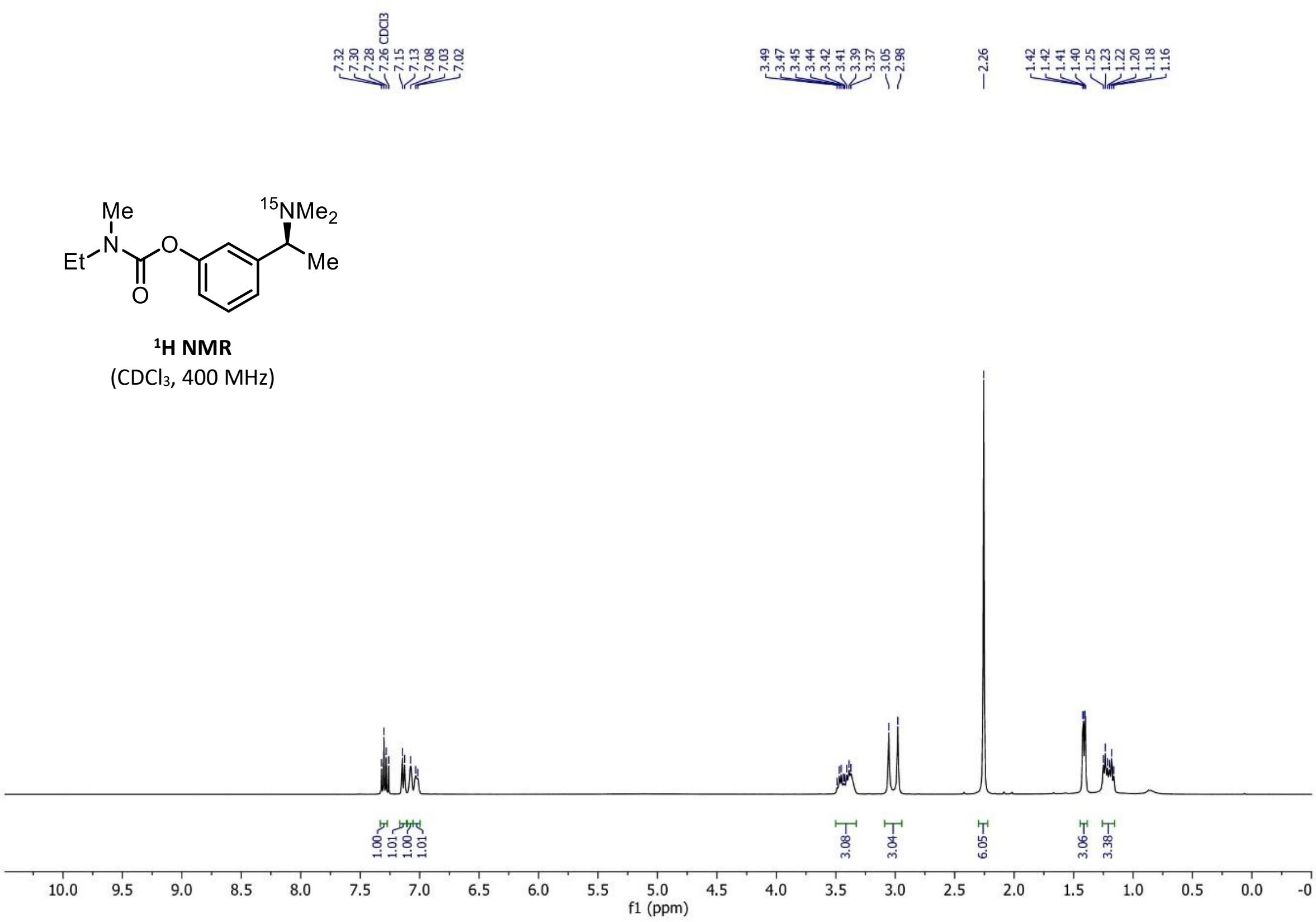

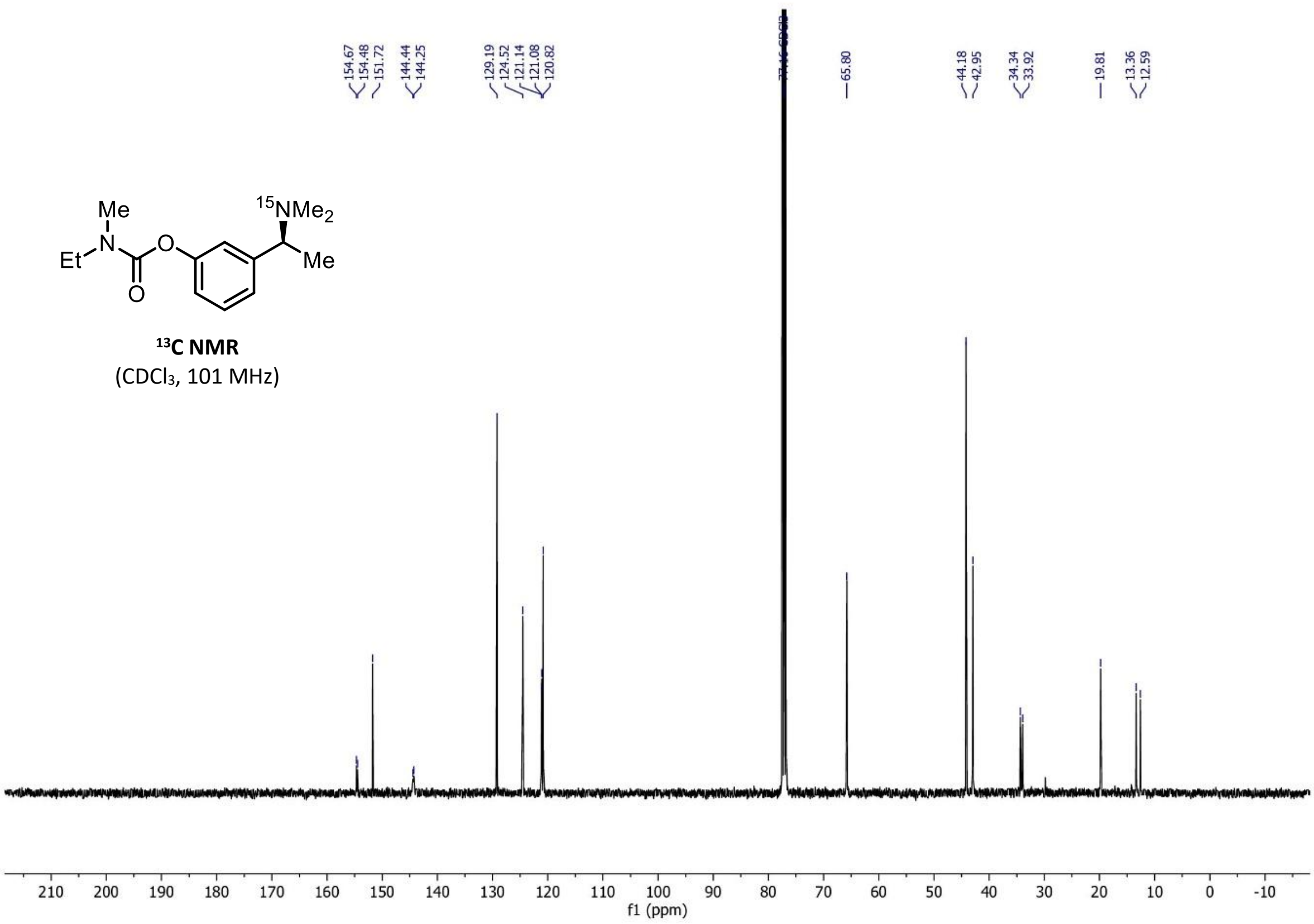

1-(3-(Dimethylamino-^{15}*N*)propyl)-1-(4-fluorophenyl)-1,3-dihydroisobenzofuran-5-carbonitrile (10b)

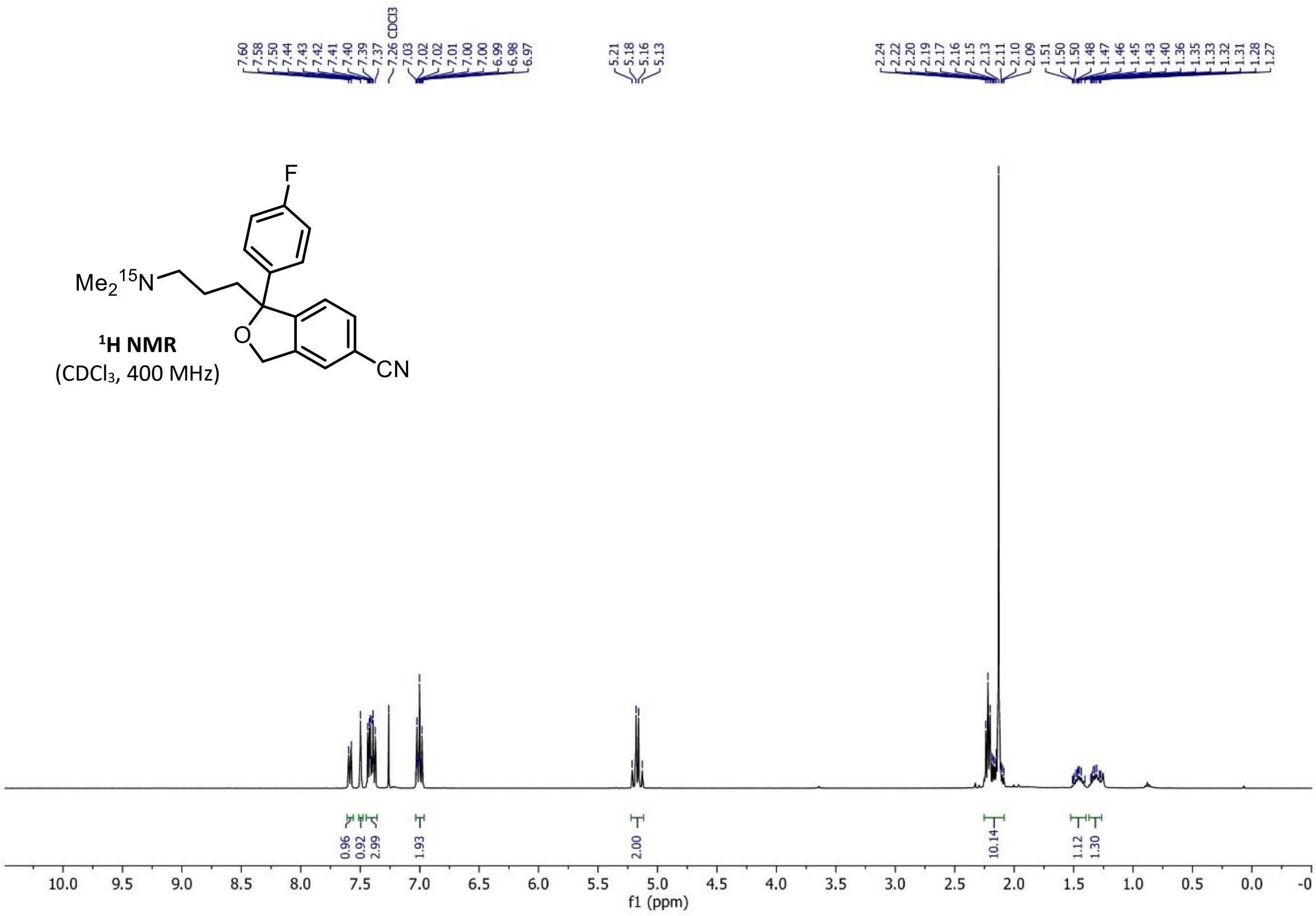

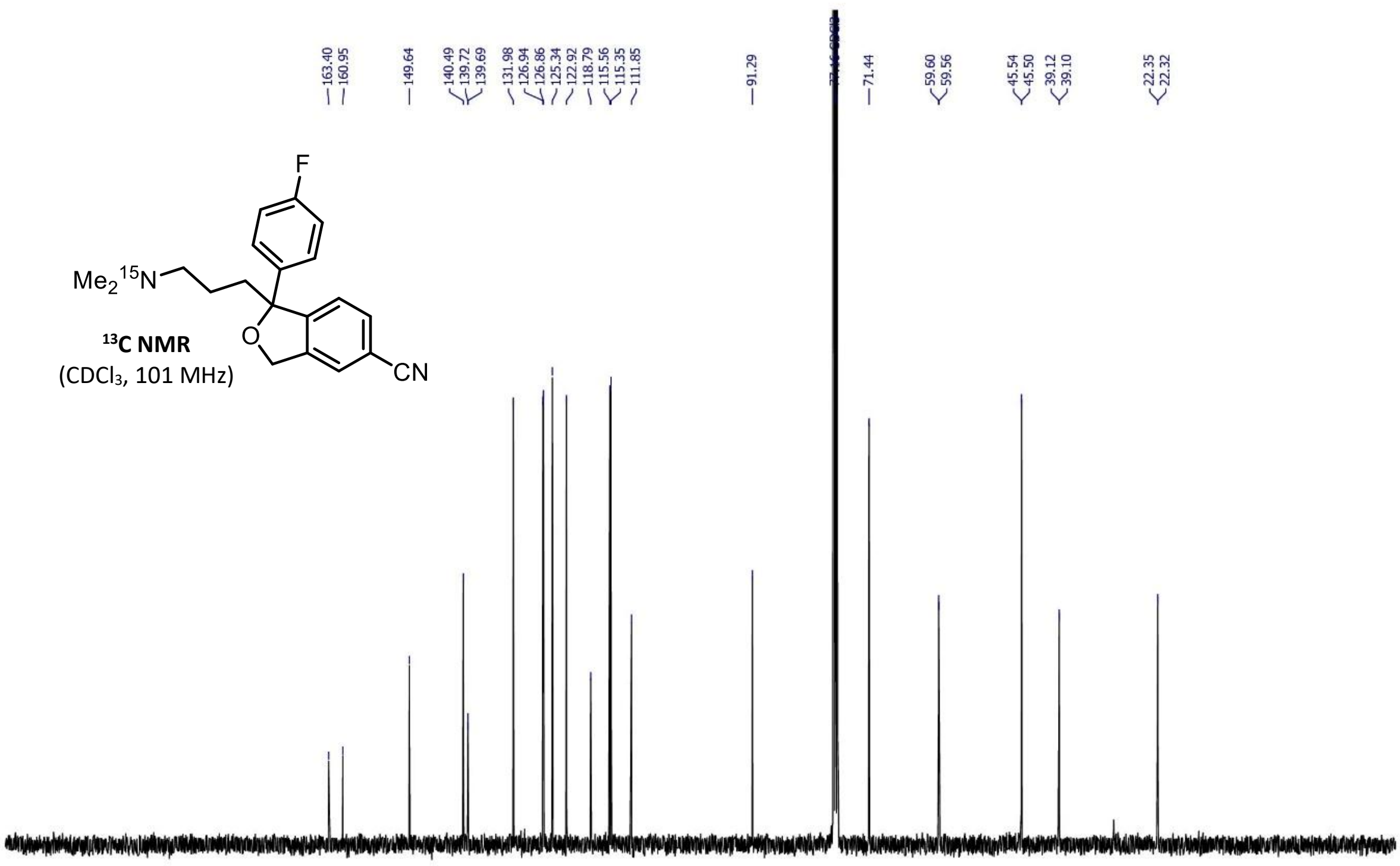

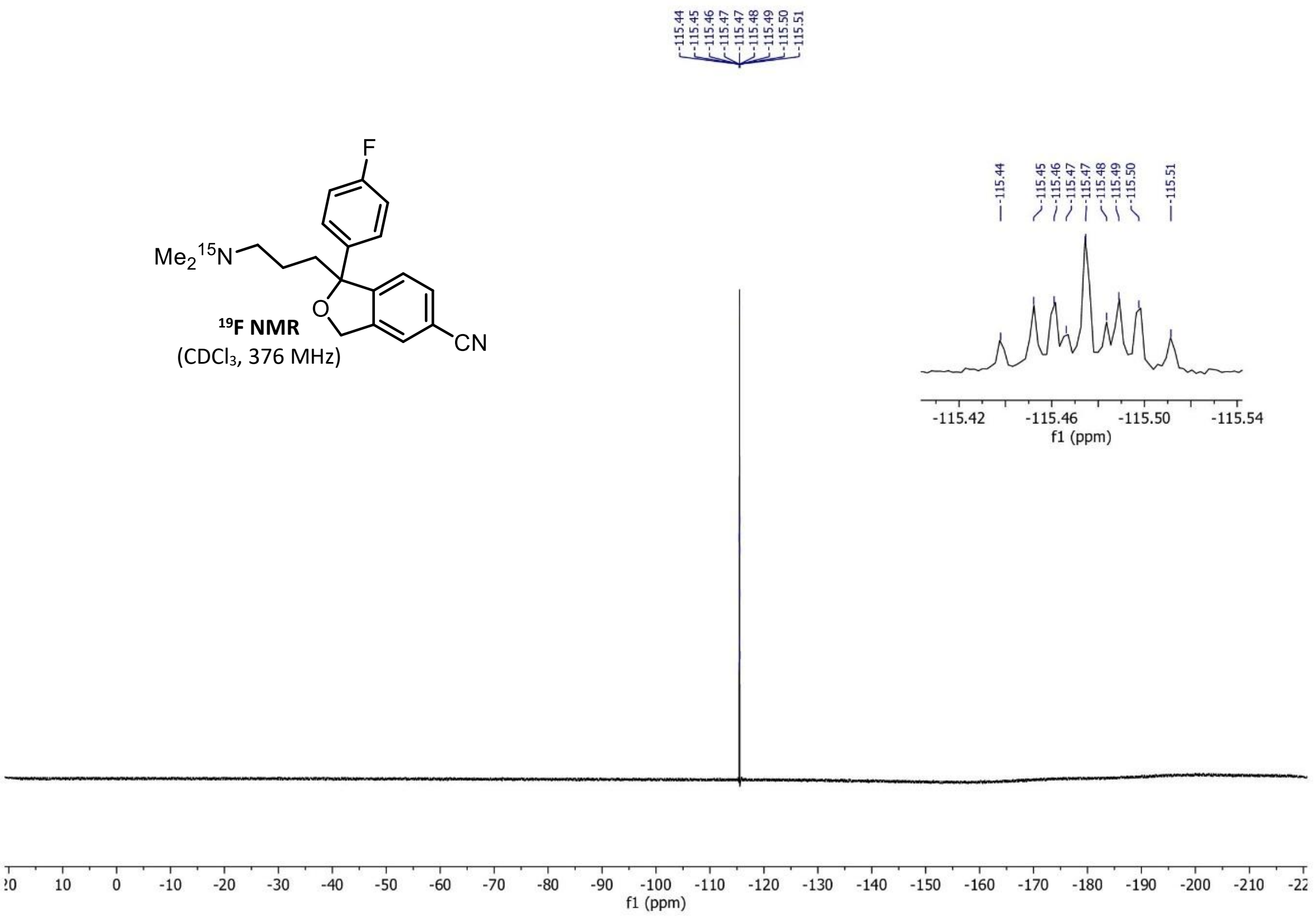

19F NMR
(CDCl3, 376 MHz)

3-(3-Chloro-10,11-dihydro-5*H*-dibenzo[*b*,*f*]azepin-5-yl)-*N*,*N*-dimethylpropan-1-amine-*N*-^{15}*N* (10c)

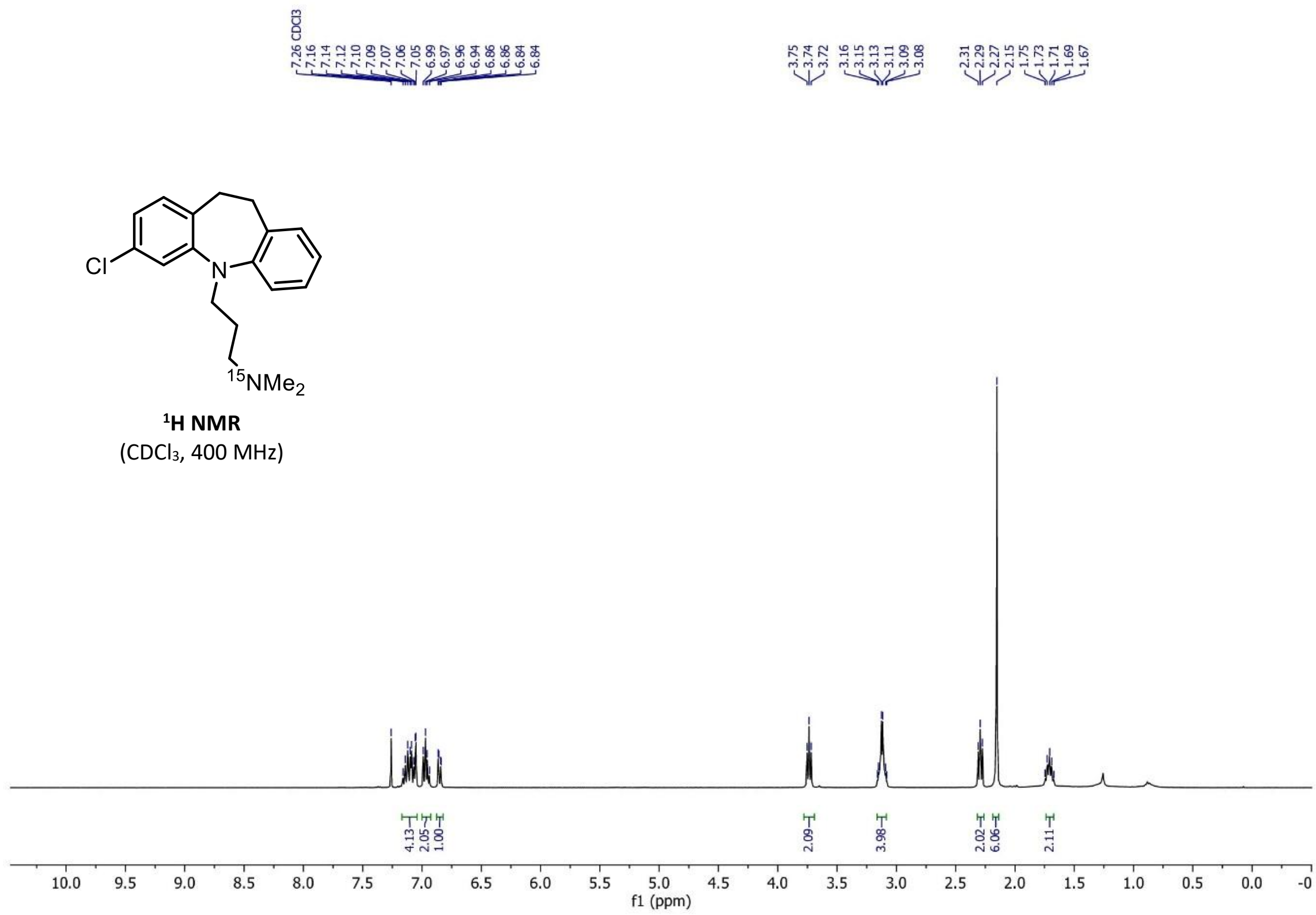

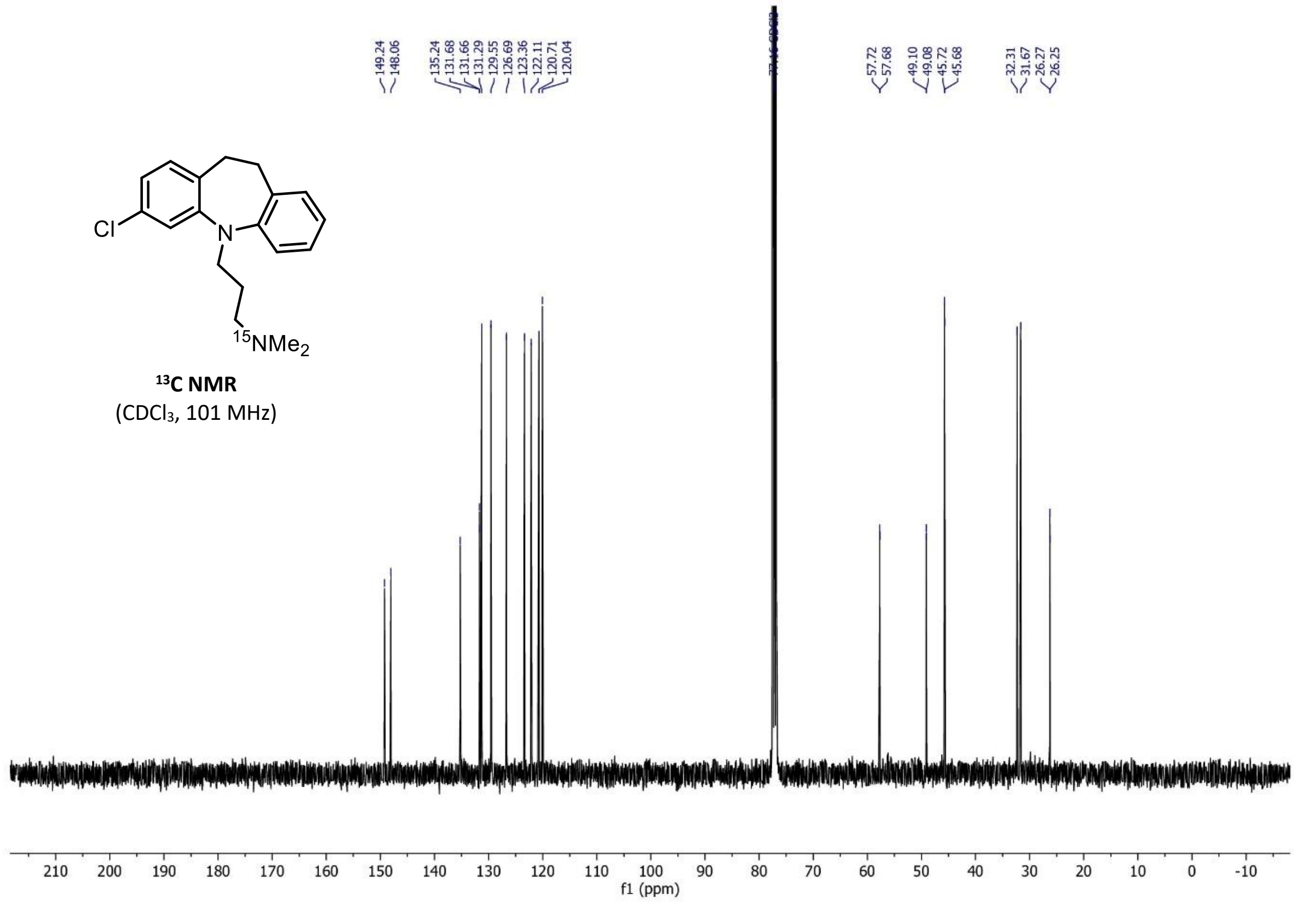

2-(3-Phenylpropoxy)-1,3,5-tris(trifluoromethyl)benzene (11)

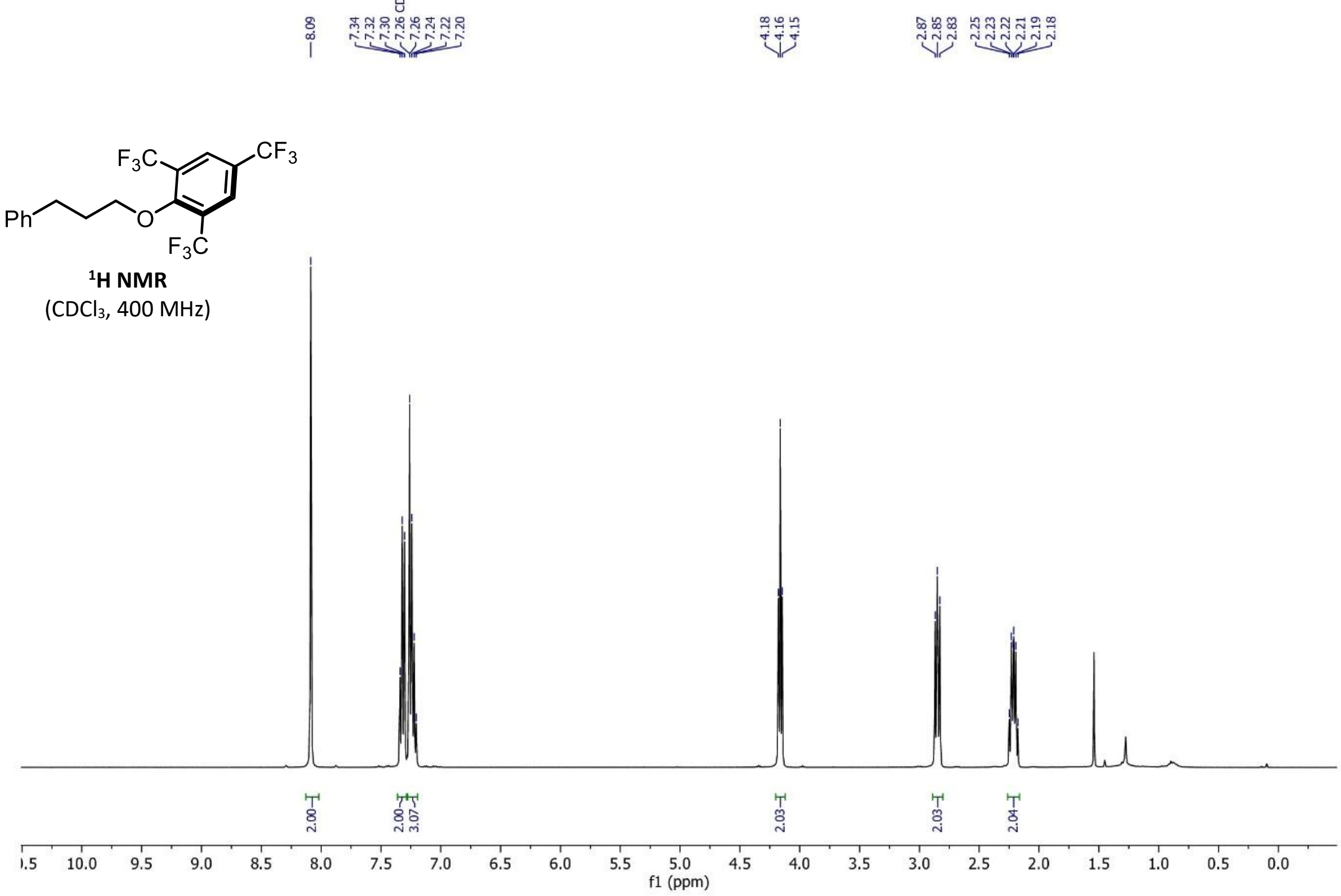

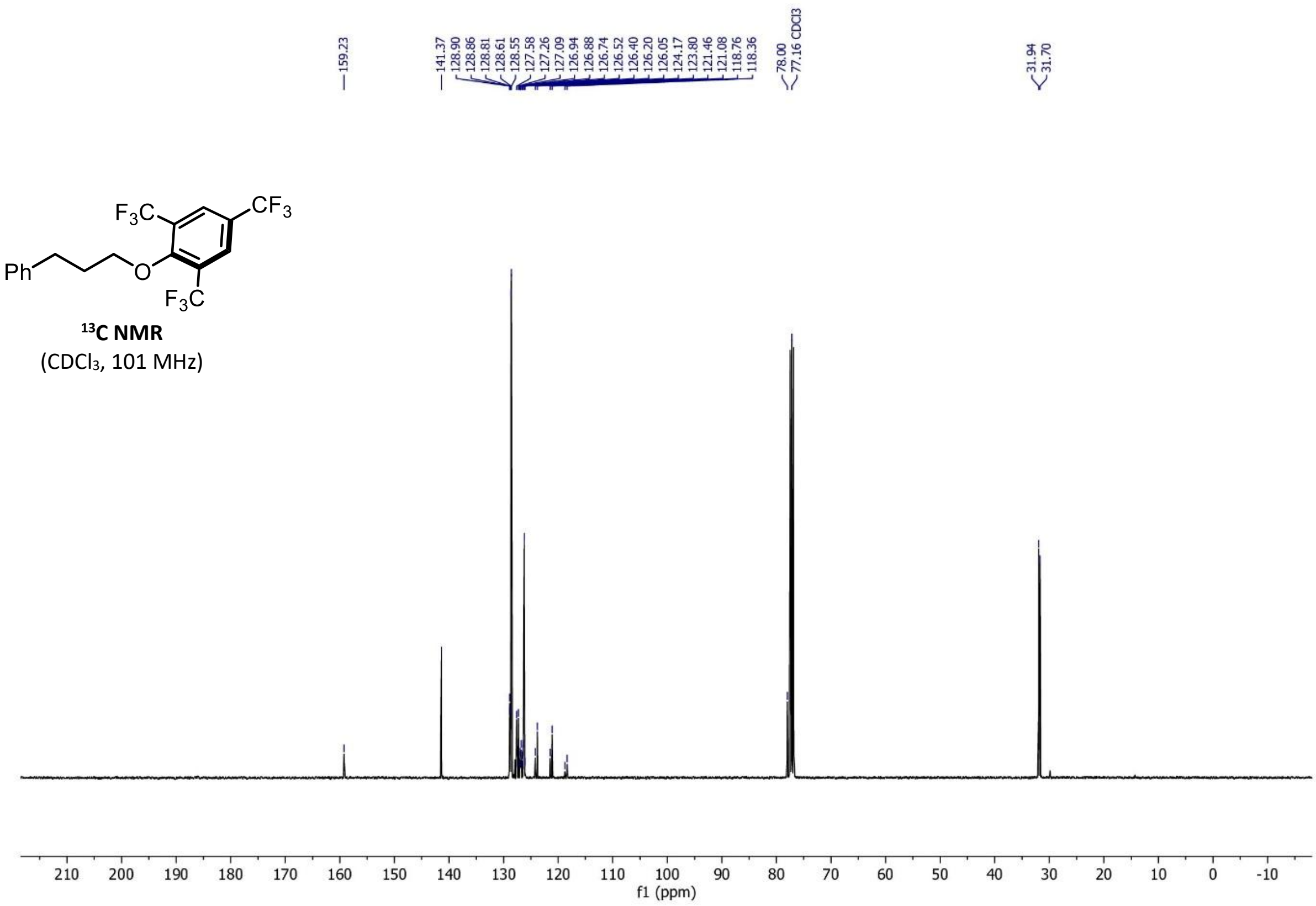

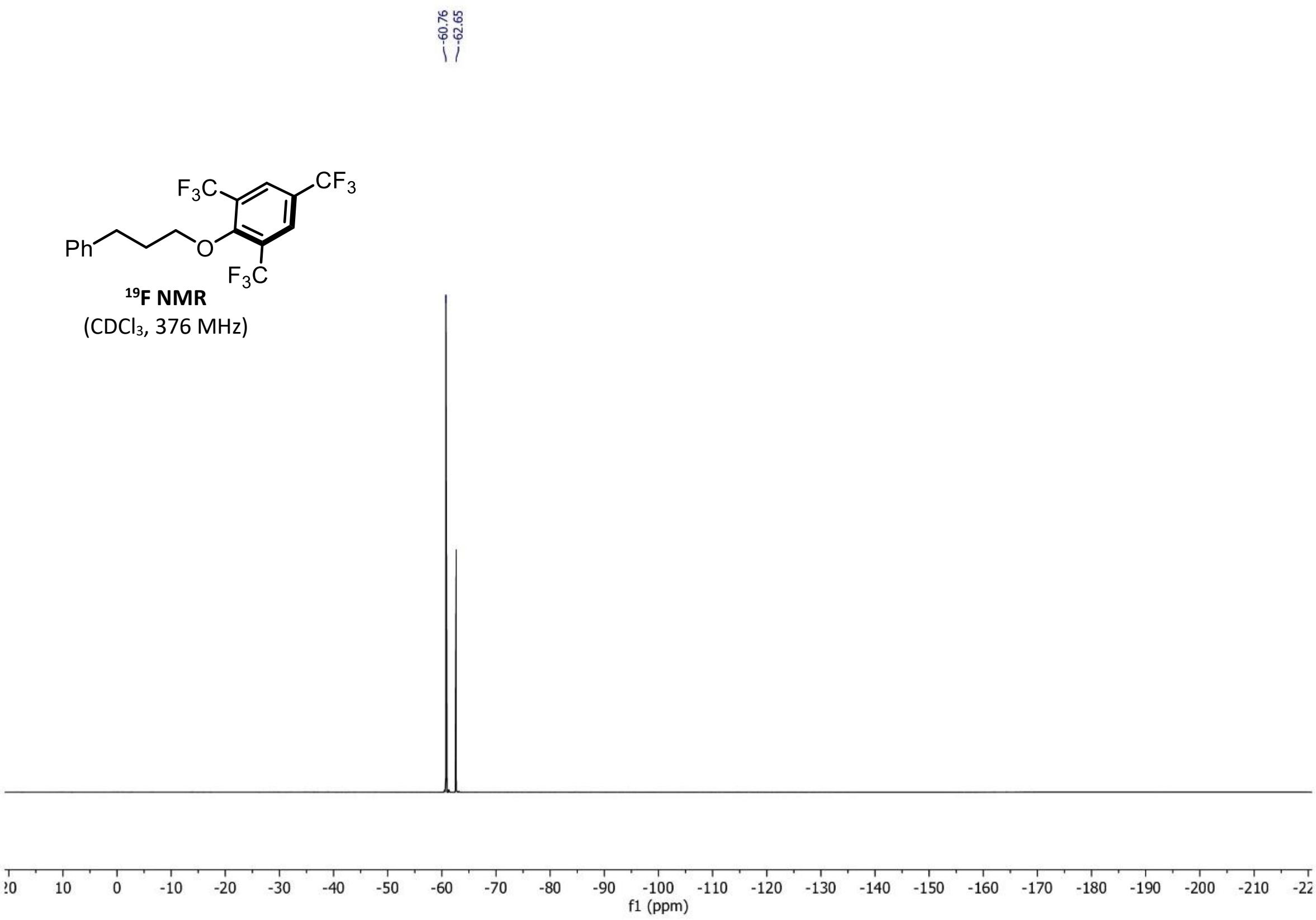
-60.76
-62.65
F_3C
CF_3
Ph
O
F_3C
19F NMR
($CDCl_3$, 376 MHz)
f1 (ppm)

6. References

[31] W. S. Bechara, I. K. Sagamanova, L. Thai-Savard, M. Dauphinais, S. Régnier, C. Noël, S. B. D. Jarvis, A. B. Charette, Universal reagent for mild and stereospecific nucleophilic substitution of alcohols with amines." *Angew. Chem. Int. Ed.* **2025**, *64*, e202420312.

[33] P. Spieß, A. Sirvent, I. Tiefenbrunner, J. Sargueil, A. J. Fernandes, A. Arroyo-Bondía, R. Meyrelles, D. Just, A. Prado-Roller, S. Shaaban, D. Kaiser, N. Maulide, "Nms-Amides: An Amine Protecting Group with Unique Stability and Selectivity." *Chem. Eur. J.* **2023**, *29*, e202301312.

[34] P. Spieß, J. Brześkiewicz, R. Meyrelles, D. Just, N. Maulide, "Deprotective Functionalization: A Direct Conversion of Nms-Amides to Carboxamides Using Carboxylic Acids." *Angew. Chem. Int. Ed.* **2024**, *63*, e202318304.

[41] S. Graßl, Y.-H. Chen, C. Hamze, C. P. Tüllmann, P. Knochel, "Late stage functionalization of secondary amines via a cobalt-catalyzed electrophilic amination of organozinc reagents." *Org. Lett.* **2019**, *21*, 494–497. [50] G. R. Fulmer, A. J. M. Miller, N. H. Sherden, H. E. Gottlieb, A. Nudelman, B. M. Stoltz, J. E. Bercaw, K. I. Goldberg, *Organometallics* **2010**, *29*, 2176–2179.

[44] A. D. Becke, "Density-functional thermochemistry. III. The role of exact exchange." *J. Chem. Phys.* **1993**, *98*, 5648–5652.

[45] S. Grimme, S. Ehrlich, L. Goerigk, "Effect of the damping function in dispersion corrected density functional theory." *J. Comput. Chem.* **2011**, *32*, 1456–1465.

[46] F. Weigend, R. Ahlrichs, "Balanced basis sets of split valence, triple zeta valence and quadruple zeta valence quality for H to Rn: Design and assessment of accuracy."*Phys. Chem. Chem. Phys.* **2005**, *7*, 3297–3305.

[47] C. Lee, W. Yang, R. G. Parr, "Development of the Colle-Salvetti correlation-energy formula into a functional of the electron density." *Phys. Rev. B Condens. Matter* **1988**, *37*, 785–789.

[48] P. J. Stephens, F. J. Devlin, C. F. Chabalowski, M. J. Frisch, "Ab initio calculation of vibrational absorption and circular dichroism spectra using density functional force fields." *J. Phys. Chem.* **1994**, *98*, 11623–11627.

[49] S. H. Vosko, L. Wilk, M. Nusair, "Accurate spin-dependent electron liquid correlation energies for local spin density calculations: a critical analysis." *Can. J. Phys.* **1980**, *58*, 1200–1211.

[50] G. R. Fulmer, A. J. M. Miller, N. H. Sherden, H. E. Gottlieb, A. Nudelman, B. M. Stoltz, J. E. Bercaw, K. I. Goldberg, "NMR chemical shifts of trace impurities: common laboratory solvents, organics, and gases in deuterated solvents relevant to the organometallic chemist." *Organometallics* **2010**, *29*, 2176–2179.

[51] C. Aïssa, A. Fürstner, "A Rhodium-Catalyzed C−H Activation/Cycloisomerization Tandem." *J. Am. Chem. Soc.* **2007**, *129*, 14836–14837.

[52] S. K. Kariofillis, B. J. Shields, M. A. Tekle-Smith, M. J. Zacuto, A. G. Doyle, "Nickel/photoredox-catalyzed methylation of (hetero) aryl chlorides using trimethyl orthoformate as a methyl radical source." *J. Am. Chem. Soc.* **2020**, *142*, 7683–7689.

[53] S.-H. Liu, F. Li, Y.-M. He, Q.-H. Fan, "Manganese (I)-catalyzed enantioselective formal anti-Markovnikov hydroalkoxylation of racemic allylic alcohols: a borrowing hydrogen access." *Org. Lett.* **2025**, *27*, 2139–2145.

[54] R. T. H. Linstadt, C. A. Peterson, D. J. Lippincott, C. I. Jette, B. H. Lipshutz, "Stereoselective silylcupration of conjugated alkynes in water at room temperature." *Angew. Chem. Int. Ed.* **2014**, *53*, 4159–4163.

[55] F. Chen, Y. Zhang, L. Yu, S. Zhu, "Enantioselective NiH/Pmrox-catalyzed 1, 2-reduction of α, β-unsaturated ketones." *Angew. Chem. Int. Ed.* **2017**, *56*, 2022–2025.

[56] N. S Mahajani, R. I. L. Meador, R. T. J. Smith, S. E. Canarelli, A. A. Adhikari, J. P. Shah, C. M. Russo, D. R. Wallach, K. T. Howard, A. M. Millimaci, J. D. Chisholm, "Ester formation via symbiotic activation utilizing trichloroacetimidate electrophiles" *J. Org. Chem.* **2019**, *84*, 7871–7882.

[57] P. H. Huy, I. Filbrich, "A General Catalytic Method for Highly Cost-and Atom-Efficient Nucleophilic Substitutions." *Chem. Eur. J.* **2018**, *24*, 7410–7416.

[58] M. H. Rønnest, F. Nissen, P. J. Pedersen, T. O. Larsen, W. Mier, M. H. Clausen, "A mild method for regioselective labeling of aromatics with radioactive iodine." *European J. Org. Chem.* **2013**, *2013*, 3970–3973.

[59] W. H. Steel, F. Damkaci, R. Nolan, R. A. Walker, "Molecular rulers: New families of molecules for measuring interfacial widths." *J. Am. Chem. Soc.* **2002**, *124*, 4824–4831.

[60] X. Liu, B. Xu, M. Oestreich, "One-Pot Sequential Alcohol Activation and Nickel-Catalyzed Cross-Electrophile Coupling with Chlorosilanes**.**"*Org. Lett.* **2025**, *27*, 3686–3690.

[61] Y. Sasano, N. Kogure, S. Nagasawa, K. Kasabata, Y. Iwabuchi, "2-Azaadamantane N-oxyl (AZADO)/Cu catalysis enables chemoselective aerobic oxidation of alcohols containing electron-rich divalent sulfur functionalities." *Org. Lett.* **2018**, *20*, 6104–6107.

[62] D. Wei, J. Bu, S. Zhang, S. Chen, L. Yue, X. Li, K. Liang, C. Xia, "Light-Driven Stepwise Reduction of Aliphatic Carboxylic Esters to Aldehydes and Alcohols." *Angew. Chem. Int. Ed.* **2025**, *64*, e202420084.

[63] F. Louafi, J. Moreau, S. Shahane, S. Golhen, T. Roisnel, S. Sinbandhit, J.-P. Hurvois, "Electrochemical synthesis and chemistry of chiral 1-cyanotetrahydroisoquinolines. an approach to the asymmetric syntheses of the alkaloid (−)-crispine A and its natural (+)-antipode." *J. Org. Chem.* **2011**, *76*, 9720–9732.

[64] P. Knochel, A. Krasovskiy, "Convenient Titration Method for Organometallic Zinc, Magnesium, and Lanthanide Reagents." *Synthesis (Mass.)* **2006**, *2006*, 0890–0891.

[65] S.-X. Zhang, Y. Ding, J.-J. Wang, C. Shen, X. Zhou, X.-Q. Chu, M. Ma, Z.-L. Shen, "Titanium (IV)-Mediated Ring-Opening/Dehydroxylative Cross-Coupling of Diaryl-Substituted Methanols with Cyclopropanol Derivatives." *J. Org. Chem.* **2021**, *86*, 15753–15760.

[66] X. Bao, Q. Wang, J. Zhu, "Remote C (sp^3)– H Arylation and Vinylation of N-Alkoxypyridinium Salts to δ-Aryl and δ-Vinyl Alcohols." *Chem. Eur. J.* **2019**, *25*, 11630–11634.

[67] D. L. Kane, B. C. Figula, K. Balaraman, J. A. Bertke, C. Wolf, "Cryogenic organometallic carbon–fluoride bond functionalization with broad functional group tolerance." *J. Am. Chem. Soc.* **2025**, *147*, 5764–5774.

[68] S. Grimme, "Exploration of chemical compound, conformer, and reaction space with meta-dynamics simulations based on tight-binding quantum chemical calculations." *J. Chem. Theory Comput.* **2019**, *15*, 2847–2862.

[69] P. Pracht, F. Bohle, S. Grimme, "Automated exploration of the low-energy chemical space with fast quantum chemical methods." *Phys. Chem. Chem. Phys.* **2020**, *22*, 7169–7192.

[70] C. Bannwarth, S. Ehlert, S. Grimme, "GFN2-xTB—An accurate and broadly parametrized self-consistent tight-binding quantum chemical method with multipole electrostatics and density-dependent dispersion contributions." *J. Chem. Theory Comput.* **2019**, *15*, 1652–1671.

[71] G. Luchini, J. Alegre-Requena, I. Funes-Ardoiz, R. Paton, "GoodVibes: automated thermochemistry for heterogeneous computational chemistry data." *F1000Res.* **2020**, 291.

[72] I. Funes-Ardoiz, R. S. Paton, "GoodVibes: GoodVibes v1. 0.1." *Zenodo* **2016**.

[73] E. Cancès, B. Mennucci, J. Tomasi, "A new integral equation formalism for the polarizable continuum model: Theoretical background and applications to isotropic and anisotropic dielectrics." *J. Chem. Phys.* **1997**, *107*, 3032–3041.

[74] A. V. Marenich, C. J. Cramer, D. G. Truhlar, "Universal solvation model based on solute electron density and on a continuum model of the solvent defined by the bulk dielectric constant and atomic surface tensions." *J. Phys. Chem. B* **2009**, *113*, 6378–6396.

[75] E. Engelage, N. Schulz, F. Heinen, S. M. Huber, D. Truhlar, C. Cramer, "Refined SMD parameters for bromine and iodine accurately model halogen-bonding interactions in solution." *Chem. Eur. J.* **2018**, *24*, 15983–15987.

[76] Frisch, M. J.; Trucks, G. W.; Cheeseman, J. R.; Scalmani, G.; Caricato, M.; Hratchian, H. P.; Li, X.; Barone, V.; Bloino, J.; Zheng, G.; Vreven, T.; Montgomery, J. A.; Petersson, G. A.; Scuseria, G. E.; Schlegel, H. B.; Nakatsuji, H.; Izmaylov, A. F.; Martin, R. L.; Sonnenberg, J. L.; Peralta, J. E.; Heyd, J. J.; Brothers, E.; Ogliaro, F.; Bearpark, M.; Robb, M. A.; Mennucci, B.; Kudin, K. N.; Staroverov, V. N.; Kobayashi, R.; Normand, J.; Rendell, A.; Gomperts, R.; Zakrzewski, V. G.; Hada, M.; Ehara, M.; Toyota, K.; Fukuda, R.; Hasegawa, J.; Ishida, M.; Nakajima, T.; Honda, Y.; Kitao, O.; Nakai, H. Gaussian 16, Revision C.01.
